# Chiral Lewis Base‐Catalysed Asymmetric Syntheses of Benzo‐fused ϵ‐Lactones

**DOI:** 10.1002/ejoc.202300704

**Published:** 2023-09-18

**Authors:** Lotte Stockhammer, Maximilian Radetzky, Syeda Sadia Khatoon, Matthias Bechmann, Mario Waser

**Affiliations:** ^1^ Institute of Organic Chemistry Johannes Kepler University Linz Altenbergerstrasse 69 4040 Linz Austria

**Keywords:** asymmetric organocatalysis, cyclizations, lactones, Lewis base catalysis, quinone methides

## Abstract

We herein report a two‐step protocol for the asymmetric synthesis of novel chiral benzofused ϵ‐lactones starting from O‐protected hydroxymethyl‐para‐quinone methides and activated aryl esters. By using chiral isothiourea Lewis base catalysts a broad variety of differently substituted products could be obtained in yields of around 50 % over both steps with high levels of enantioselectivities, albeit low diastereoselectivities only.

## Graphical abstract

An asymmetric protocol for the synthesis of novel benzofused ϵ‐lactones starting from quinone methides and activated acetic acid esters using chiral isothiourea Lewis base catalysts has been developed.

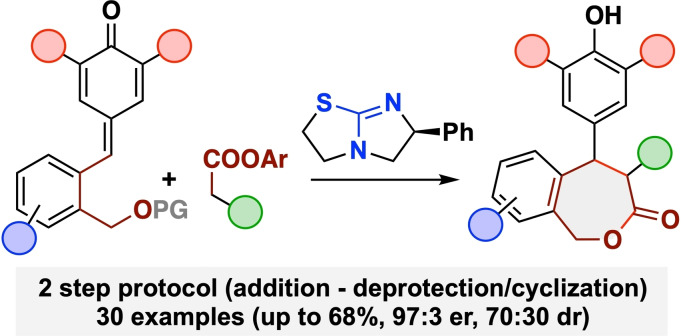

## Introduction

**Scheme 1:**
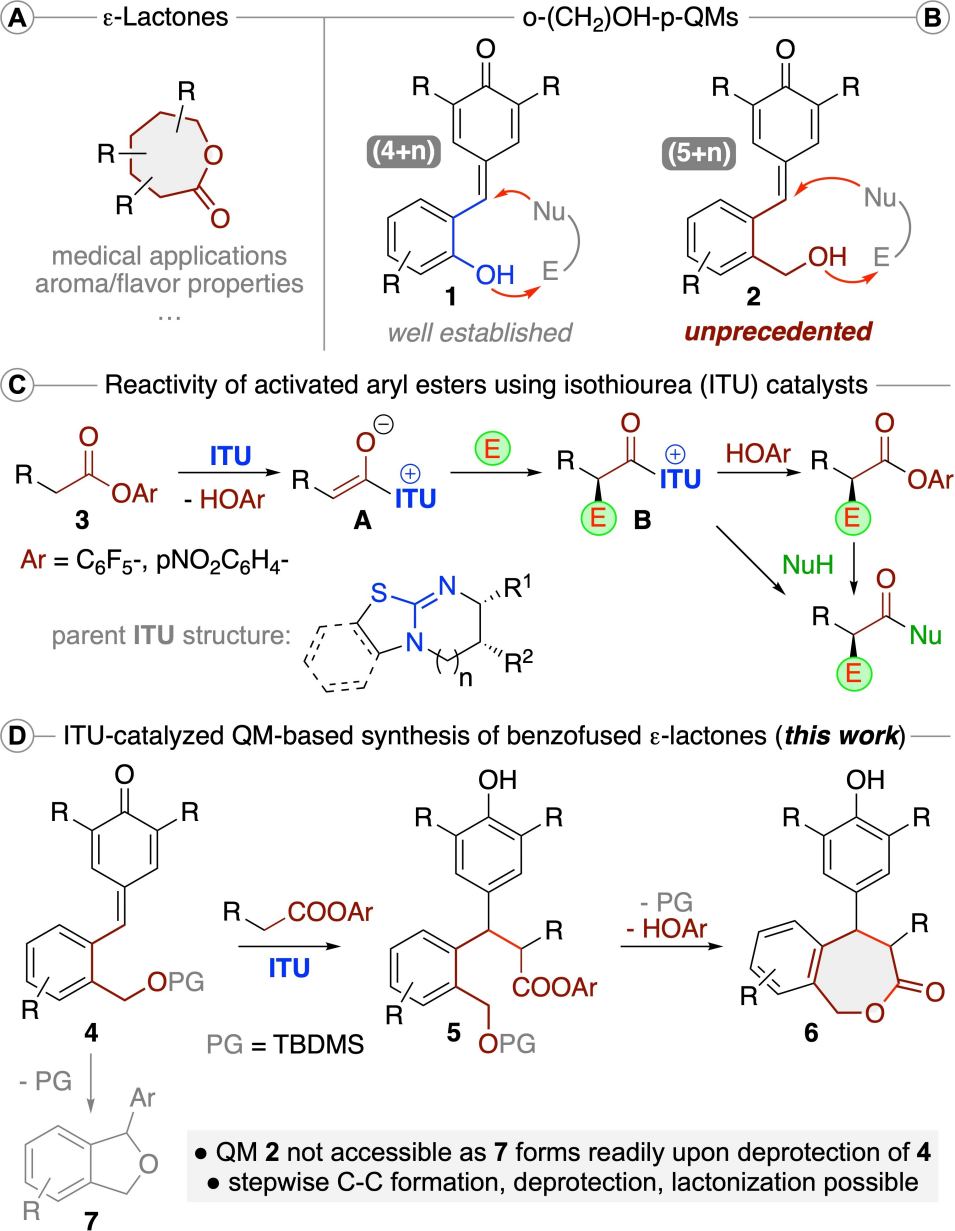
(A) General structure of ϵ‐lactones. (B) OH‐functionalized p‐QMs **1** and **2**. (C) The general reactivity of aryl esters **3** under ITU catalysis. (D) The herein investigated ITU‐catalysed QM **4**‐based syntheses of benzofused

Seven‐membered chiral lactones (ϵ‐lactones) represent an important class of heterocyclic molecules associated with a diverse variety of properties (Scheme 1A).[^1^, ^2^] For example, some naturally occurring ϵ‐lactone derivatives show interesting biological activities that make them promising lead structures for medical applications.^[2]^ In addition, such lactones often represent the core structural motives responsible for flavour and aroma properties of natural products.^[1]^ Considering the value of these compounds, the development of asymmetric strategies that give access to new chemical space by providing novel highly functionalized ϵ‐lactone derivatives thus represents a worthwhile goal.[^3^, ^4^, ^5^, ^6^] Over the course of the last years our group has had a strong focus on the development of asymmetric heterocycle forming reactions.^[7]^ Hereby we have been especially fascinated by the potential of preformed or in situ formed quinone methides (QMs) as building blocks for the synthesis of chiral oxygen‐containing heterocycles.^[8]^ One class of QMs that serves well for cyclization reactions are o‐OH‐functionalized p‐QMs **1** (Scheme 1B), which can serve as versatile 4‐atom building blocks for formal (4+n)‐cyclization reactions when reacted with various dipolar species.[^5c^, ^5d^, ^5e^, ^7c^, ^9^] In contrast, the homologous o‐hydroxymethyl p‐QMs **2** have, to the best of our knowledge, so far not been systematically explored for analogous formal (5+n)‐cyclization reactions.

Chiral Lewis bases,^[10]^ i. e. isothioureas (ITUs), have emerged as powerful nucleophilic organocatalysts for the activation and control of in situ activated carboxylic acids or electron‐deficient aryl esters **3** (Scheme 1C).[^11^, ^12^, ^13^, ^14^] Upon displacement of the OAr group by the ITU and subsequent α‐deprotonation, chiral C1 ammonium enolates **A** are formed, which then add to different electrophiles in an enantioselective manner giving the catalyst‐bound products **B**. These species can then react with nucleophiles (like the initially cleaved‐off HOAr^[11c]^ group or an external nucleophile) either in a cyclization approach or in a bimolecular manner by releasing the catalyst again.^[14]^ Our group has recently contributed to the field of ITU catalysis^[15]^ and we were now wondering if it is possible to utilize this powerful catalysis concept for the hitherto unprecedented synthesis of novel benzofused ϵ‐lactones **6** (Scheme 1D). We initially thought of accessing p‐QMs **2** by deprotecting the easily available p‐QMs **4**,^[16]^ but soon realized that the deprotection of **4** directly leads to the formation of the dihydroisobenzofuran derivatives **7** by immediate 1,6‐addition of the free OH group to the p‐QM moiety. Gratifyingly, we were able to overcome this inherent limitation by developing a stepwise approach which we wish to outline in this contribution. As summarized in Scheme 1D, we utilized O‐protected p‐QMs **4** for an ITU‐catalysed reaction with arylesters **3** first (giving products **5**),^[17]^ followed by deprotection and cyclization to the benzofused ϵ‐lactones **6** in a second step.

## Results and Discussion

We started our investigations by optimizing the addition of the nitrophenol ester **3 a** to the silyl‐protected p‐QM **4 a** using the established catalysts **ITU1‐6** (Table 1 gives an overview of the most significant results obtained in this screening). First experiments with achiral **ITU1** proved the feasibility of this transformation by giving the addition product **5 a** as a mixture of diastereoisomers in moderate yield (entry 1). Interestingly, the reaction itself was found to be relatively slow and required three days to achieve a reasonable conversion of starting material **3 a**. Notable amounts of free p‐nitrophenol were formed hereby, which could be traced back to a background hydrolysis of **3 a** under the reaction conditions (product **5 a** was found to be more stable towards ester hydrolysis). Attempts to improve conversion rate and yield by using one reaction partner in access were not successful as the conversion increased only slightly but isolation by silica gel column‐ or thin layer‐chromatography was much more tedious in those cases. Therefore, we used **3 a** and **4 a** in an equimolar manner throughout the whole optimization process (we retested alternative ratios later under the optimized asymmetric conditions as well but without any benefit). Gratifyingly, product **5 a** could be deprotected – cyclized in a reliable and high yielding manner by using Bu_4_NBr_3_ in MeOH for the desilylation followed by immediate DMAP‐mediated cyclization. The targeted ϵ‐lactone **6 a** was hereby obtained almost quantitatively (based on **5 a**) and with full retention of the diastereomeric ratio observed for **5 a**. Assignment of the relative configurations of both diastereomers (*cis*=*unlike*; *trans*=*like*) was possible by comparison of the experimental NMR results (chemical shifts and ^3^J_HH_ coupling) with predicted data of DFT‐optimized structures of the single diastereoisomers.^[16]^

**Table 1:** Optimization of the stepwise synthesis of the ϵ‐lactone **6 a**.^[a]^

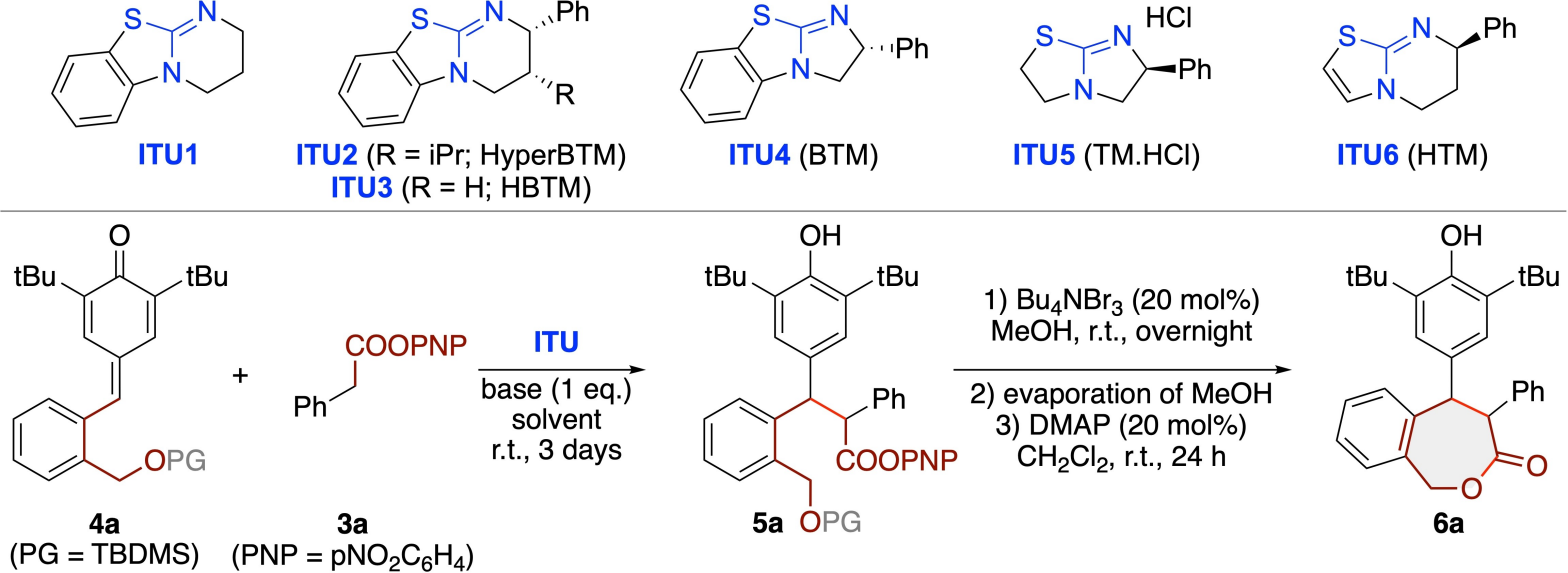											
Entry	ITU [mol%]	Base	Solvent (step 1)	Conv. (**3 a**)^[b]^ [%]	**5 a**			**6 a**			
					yield^[c]^ [%]	ArOH^[d]^ [%]	dr^[b]^	yield^[e]^ [%]	*cis* : *trans* ^[b,f]^	er (*cis*)^[g]^	er (*trans*)^[g]^
1	**ITU1** (10)	DIPEA	MeCN	80	45	15	75 : 25	40	75 : 25	–	–
2	**ITU2** (10)	DIPEA	MeCN	20	n. d.	n. d.	n. d.	–	–	–	–
3	**ITU2** (20)	DIPEA	DMF	65	35	30	75 : 25	34	75 : 25	45 : 55	46 : 54
4	**ITU3** (20)	DIPEA	DMF	65	40	25	70 : 30	38	70 : 30	35 : 65	n. d.
5	**ITU4** (20)	DIPEA	DMF	75	40	25	60 : 40	35	60 : 40	25 : 75	15 : 85
6	**ITU5** (20)	DIPEA	DMF	>95	40	25	50 : 50	28	50 : 50	87 : 13	96 : 4
7	**ITU5** (20)	DIPEA	MeCN	65	40	15	50 : 50	29	50 : 50	92 : 8	97 : 3
8	**ITU6** (20)	DIPEA	MeCN	65	53	10	65 : 45	45	65 : 45	88 : 12	88 : 12
9	**ITU5** (20)	DIPEA	EtOAc	n. r.	–	–	–	–	–	–	–
10	**ITU5** (20)	DIPEA	MeCN/DMF^[h]^	80	55	15	50 : 50	51	50 : 50	92 : 8	97 : 3
11	**ITU5** (20)	DIPEA (dry)	MeCN (dry)^[i]^	65	36	20	50 : 50	35	50 : 50	94 : 6	97 : 3
12	**ITU5** (20)	Et_3_N	MeCN	65	38	10	50 : 50	31	50 : 50	95 : 5	97 : 3
13	**ITU5** (20)	Cs_2_CO_3_	MeCN	>95	40	5	50 : 50	34	50 : 50	82 : 18	82 : 18
14	**ITU5** (20)	2,6‐Lutidine	MeCN	n. r.	–	–	–	–	–	–	–
15	**ITU5** (20)	proton sponge	MeCN	60	36	10	50 : 50	34	50 : 50	96 : 4	98 : 2
16	**ITU5** (20)	TMP	MeCN	80	57	10	50 : 50	51 (50)^[k]^	50 : 50 (50 : 50)^[k]^	93 : 7 (93 : 7)^[k]^	97 : 3 (96 : 4)^[k]^
17	**ITU5** (20)	TMP	MeCN/DMF^[h]^	90	56	35	50 : 50	53	50 : 50	90 : 10	95 : 5
18	**ITU5** (20)	TMP	MeCN^[j]^	n. r.	–	–	–	–	–	–	–

[a] All reactions were carried out by stirring 0.1 mmol **4 a**, 0.1 mmol **3 a**, 0.1 mmol base (DIPEA=diisopropylethylamine, TMP=2,2,6,6‐tetramethylpiperidine) and the indicated catalyst in the given solvent (0.1 M) for 3 days unless otherwise stated. [b] Judged by ^1^H NMR of the crude product. [c] Isolated yields of the mixtures of diastereoisomers. [d] p‐Nitrophenol formed by background hydrolysis of starting material **3 a** (judged by ^1^H NMR of the crude product). [e] Isolated yield over both steps. [f] Relative configuration assigned based on NOE correlations. [g] Determined by HPLC using a chiral stationary phase. [h] Different ratios were tested where a MeCN/DMF ratio of 9/1 proofed to be most suitable. [i] Dry MeCN was freshly degassed prior to use. [j] Run at −40 °C. [k] 1 mmol scale.

Screening chiral catalysts next revealed a strong effect of catalyst structure, catalyst loading, and solvent on the reaction performance. Using 10 mol% of Hyper‐BTM (**ITU2**) in MeCN first gave almost no product **5 a** (entry 2). In contrast, with 20 mol% catalyst loading in DMF a reasonable conversion of **3 a** (65 %) was possible, but significant amounts of nitrophenol were formed as well (entry 3). While attempts to determine the enantiomeric ratio on this stage failed, both diastereomers of the cyclic product **6 a** could be readily analysed by HPLC using a chiral stationary phase. While **ITU2** gave almost racemic product only (favouring the cis isomer), **ITU3‐5** showed higher enantioselectivities, but lower diastereoselectivities (entries 4–6). Tetramisole (**ITU5**) was found to be best‐suited, giving **6 a** in a combined yield of 28 % with reasonable levels of enantioselectivities in DMF. As this reaction however delivered significant amounts of free nitrophenol and other side products as well, we next tested alternative solvents as well as the less‐commonly used homotetramisole **ITU6** ^[13b]^ (entries 7–11). While **ITU6** did not allow for any improvement (entry 8), the use of MeCN as the solvent resulted in slower but cleaner addition reactions and higher enantioselectivities. Interestingly, a mixture of MeCN/DMF (9/1) was beneficial compared to MeCN alone when using DIPEA as a base (compare entries 7 and 10). Finally, we also tested different bases (entries 12–15). We initially used pure MeCN as the solvent for this evaluation, as this allows for an operationally simpler direct solvent evaporation after the first addition step. This screening allowed us to identify 2,2,6,6‐tetramethylpiperidine (TMP) as the best‐suited base, which delivered the cyclic product **6 a** in more or less the same yield, er, and dr in MeCN (entry 16) as DIPEA in a MeCN/DMF mixture (entry 10). Surprisingly, employing TMP in a MeCN/DMF mixture turned out to be not beneficial (entry 17) and considering the fact that the reaction does not proceed at lower temperature (entry 18) we thus identified the conditions outlined in entry 16 as the best‐suited ones to access the ϵ‐lactone **6 a** in a reasonable overall two‐step yield of 51 % with high levels of enantioselectivities but unfortunately no preference for either diastereoisomer. Interestingly, cyclization towards the trans isomer was found to be faster than towards the cis, however this aspect could not be utilized in a practically useful manner as separation of trans‐**6 a** from a mixture of cis‐**6 a** and intermediate O‐deprotected **5 a** was found to be difficult. It should be noted that rather low diastereoselectivities have recently also been observed for ITU‐catalysed additions of esters **3** to classical p‐QMs,^[17a]^ thus representing a more general limitation when this covalent activation principle is used for QM addition reactions. The dr itself remained constant over the progress of the reaction and unfortunately attempts to improve the dr by means of a base‐mediated epimerization of either intermediate **5 a** or a post‐cyclization epimerization of product **6 a** failed. Also the use of well‐established pentafluorophenol esters instead of p‐nitrophenol ones did not allow for any improved stereoselectivities or reaction rates.

**Scheme 2:**
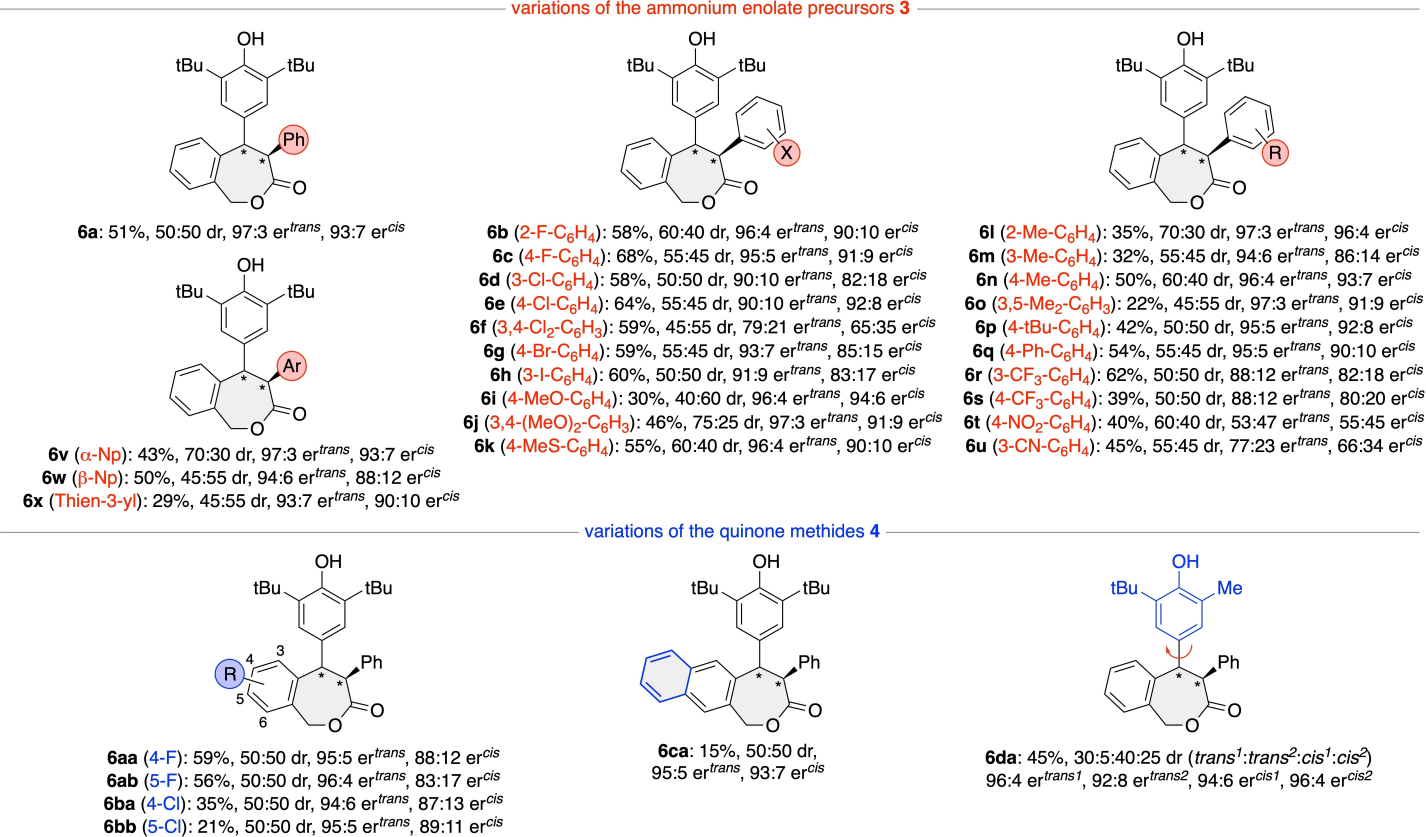
Asymmetric application scope (conditions as outlined in Table 1, entry 16).

With reliable and scalable conditions for the asymmetric two‐step synthesis of the parent ϵ‐lactone **6 a** at hand (entry 16, Table 1), we next put our attention on the generality of this process by testing a variety of differently substituted arylesters **3** and p‐QMs **4** (Scheme 2). In general, different esters **3** were reasonably well‐tolerated, giving products **6 a**‐**x** in moderate to good yields and high enantioselectivities (apart from very electron‐deficient esters as outlined for products **6 t** and **6 u**), but with low diastereoselectivities only. In those cases where reduced yields were obtained (like products **6 i**, **6 o**, and **6 x**), the first step (1,6‐addition reaction) was found to be generally slower and accompanied by a more pronounced hydrolysis of the starting esters **3** (giving the free arylacetic acid derivatives and nitrophenol), while the cyclization step performed well in all cases. Attempts to expand the application scope by using α‐alkyl acetic acid derivatives, instead of the herein used α‐aryl acetic acid derivatives **3**, failed, which is unfortunately a well‐documented limitation in C1 ammonium enolate chemistry.[^11^, ^14^, ^15^, ^17^]

Halogen‐substituted p‐QMs were also tolerated, as outlined for the syntheses of products **6 aa**–**bb**, albeit the yields dropped significantly for the Cl‐containing **6 ba** and **6 bb**, mainly because the ITU‐catalysed addition reaction was again pretty slow and accompanied by pronounced ester **3 a** hydrolysis. Unfortunately, the same limitation was observed for the naphthyl‐based product **6 ca** as well. Interesting results were obtained when using a p‐QM with a dis‐symmetrically substituted phenol part as the corresponding product **6 da** contains an additional axis of chirality, which could be controlled to some extent (interestingly hereby the *cis* diastereoisomers were favoured over the *trans*). Generally we isolated a mixture of both diastereoisomers by classical silica gel column chromatography but for further analysis the single diastereoisomers could either be separated by repeated column or thin layer chromatography or by preparative HPLC.

Concerning the absolute configuration of products **6** in general, it has to be pointed out that we were not able to obtain any crystals suited for X‐Ray diffraction analysis. However, it is a well‐documented fact that reactions of esters **3** with chiral ITUs usually proceed via a very defined chiral C1 ammonium enolate **A** (Scheme 1C), where the absolute configuration of the used catalyst allows for a good prediction of the sense of induction when reacted with an electrophile.[^11^, ^14^, ^15^, ^17^] Thus, and based on previous observations when using (*S*)‐**ITU5** with esters **3** and classical pQMs,^[17]^ it can be rationalized that the stereogenic centre alpha to the carbonyl group is controlled by the configuration of the ITU catalyst and should therefore be (S)‐configurated for both diastereoisomers in our case (as illustrated in Scheme 2).

## Conclusions

A two‐step protocol for the asymmetric syntheses of chiral ϵ‐lactones starting from O‐protected hydroxymethyl‐para‐quinone methides and activated aryl esters using chiral Lewis base catalysts was developed. The stereochemistry is controlled by using chiral isothioureas (ITUs) in the first 1,6‐addition step of the esters to the p‐QMs, allowing for satisfying levels of enantioselectivities for both diastereoisomers (which were formed in a roughly 1 : 1 ratio in most cases). These intermediate products were then successfully deprotected and cyclized to the final products, thus providing an organocatalytic entry to a diverse assembly of novel benzofused ϵ‐lactones.

## Supporting Information

Experimental procedures and analytical details of all new compounds are provided in the online Supporting Information. Additional references were cited within the Supporting Information.[^18^, ^19^]

## Conflict of interest

The authors declare no conflict of interest.

1

## Supporting information

As a service to our authors and readers, this journal provides supporting information supplied by the authors. Such materials are peer reviewed and may be re‐organized for online delivery, but are not copy‐edited or typeset. Technical support issues arising from supporting information (other than missing files) should be addressed to the authors.

Supporting Information

## Data Availability

The data that support the findings of this study are available in the supplementary material of this article.
