# Supplementary material for: Chiral Lewis Base‐Catalysed Asymmetric Syntheses of Benzo‐fused ϵ‐Lactones
Source: European J Org Chem. 2023 Sep 18;26(39):e202300704. doi: 10.1002/ejoc.202300704 (PMC11005097; doi:10.1002/ejoc.202300704)
Supplement: Supplementary file 1 — Supporting Information [file EJOC-26-0-s001.pdf]

European Journal of Organic Chemistry

Supporting Information

Chiral Lewis Base-Catalysed Asymmetric Syntheses of Benzo-fused ε-Lactones

Lotte Stockhammer, Maximilian Radetzky, Syeda Sadia Khatoon, Matthias Bechmann, and Mario Waser*

Contents

1. General Information 2

2. Synthesis of *p*-Nitrophenyl esters (General procedure A) 3

3. Synthesis of Quinone Methides 3

3.1 Overview 3

3.2 General Procedure B for the Synthesis of Diols 4

3.3 General Procedure C for the Monoprotection of Diols 4

3.4 General Procedure D for the Synthesis of Aldehydes 5

3.5 General Procedure E for the Synthesis of Quinone Methides 5

3.6 Deprotection of 4a and Subsequent Intramolecular Cyclization (Formation of 7a) 6

4. Starting Materials: Product Characterization 7

4.1 Esters 7

4.2 Diols 15

4.3 Monoprotected Diols 16

4.4 Aldehydes 19

4.5 Quinone Methides 22

5. Synthesis of 7-Membered Lactones 26

5.1 General Information 26

5.2 General Procedure F for the Synthesis of 7-Membered Lactones 26

5.3 Characterization of 7-Membered Lactones 27

6. Computational Details and Relative Configuration 88

6.1 Determination of the Relative Configuration of the Cyclic Products 88

6.2 Computational Details 91

7. References 96

8. Appendix: NMR Spectra and HPLC Chromatograms 98

8.1 NMR Spectra for Unknown Starting Materials 98

8.2 NMR Spectra for the Alkylation and Cyclization Products 133

8.3 HPLC Chromatograms for the Cyclization Products 230

1. General Information

NMR spectra were recorded on a Bruker Avance III 300 MHz spectrometer with a broad band observe probe and a sample changer for 16 samples, a Bruker Avance DRX 500 MHz spectrometer or a Bruker Avance III 700 MHz spectrometer with an Ascend magnet and TCI cryoprobe, which are property to the Austro Czech NMR Research Center "RERI uasb". All NMR spectra were referenced on the solvent residual peak ($CDCl_3$: δ 7.26 ppm for ^1H NMR and δ 77.16 ppm for ^{13}C NMR). NMR data are reported as follows: chemical shift (δ ppm), multiplicity (s = singlet, d = doublet, t = triplet, q = quartet, m = multiplet, br = broad), coupling constants (Hz) and integrals.

High resolution mass spectra were obtained using an Agilent QTOF 6520 with ESI source. Optical rotations were measured on a Schmidt+Haensch Unipol L 100 polarimeter ($[\alpha]_D$ values are listed in $deg \cdot cm^3 \cdot g^{-1} \cdot dm^{-1}$; concentration c is given in g/100 mL).

Preparative column chromatography was carried out using Davisil LC 60A 70– 200 MICRON silica gel. Thin layer chromatography was performed on Macherey-Nagel pre-coated TLC plates (silica gel, 60 F254, 0.20 mm, ALUGRAM® Xtra SIL). TLC plates were visualized under 254 nm UV lamp.

Enantiomeric ratios (e.r.) were determined by HPLC analysis using a Dionex Summit HPLC system with a CHIRAL ART Amylose-SA (4.6 mm × 250 mm, 5 µm) or a CHIRAL ART Cellulose-SB (4.6 mm × 250 mm, 5 µm) chiral stationary phase. Semipreparative HPLC was carried out using a Thermo Scientific Dionex Ultimate 3000 system with variable wavelength detection and a Grace Alltima Silica 10µm 250x10 mm column.

Dry solvents were taken from an mBRAUN SPS solvent purifier. All reactions were run under an Argon atmosphere unless otherwise stated. All chemicals were purchased from commercial suppliers and used without further purification unless otherwise stated.

Catalysts used were either commercially available (HBTM from Sigma Aldrich, TM HCl from Fluorochem, HyperBTM from Apollo) or synthesized according to known procedures (HTM according to V. B. Birmans protocol [1] and Okamotos's catalyst (DHPB) according to A.D. Smiths protocol [2]).

2. Synthesis of *p*-Nitrophenyl esters (General procedure A)

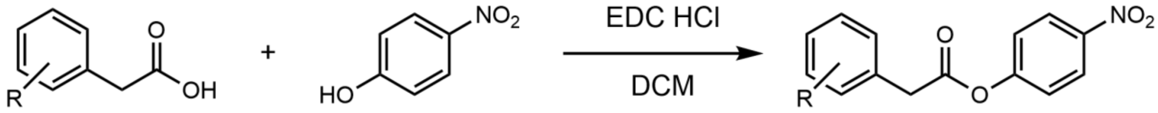

In analogy to a known procedure [3], the respective phenylacetic acid (1 eq) and EDC · HCl (1.3 eq) were dissolved in DCM (0.5 mol L^{-1}) and stirred at r.t. until everything was dissolved and the solution turned clear. Then, *p*-nitrophenol (1.5 eq) was added in one portion and the mixture was stirred at r.t. overnight. The reaction was quenched by the addition of water, the phases were separated, and the aqueous phase was extracted with DCM two more times. The combined organic phases were dried with Na_2SO_4, filtered, and concentrated to give *p*-nitrophenyl esters **3**. The crude products were purified by column chromatography on silica.

3. Synthesis of Quinone Methides

3.1 Overview

Quinone Methides were synthesized from commercially available phthalic acid anhydrides or phthalides following the 4-step procedure depicted below:

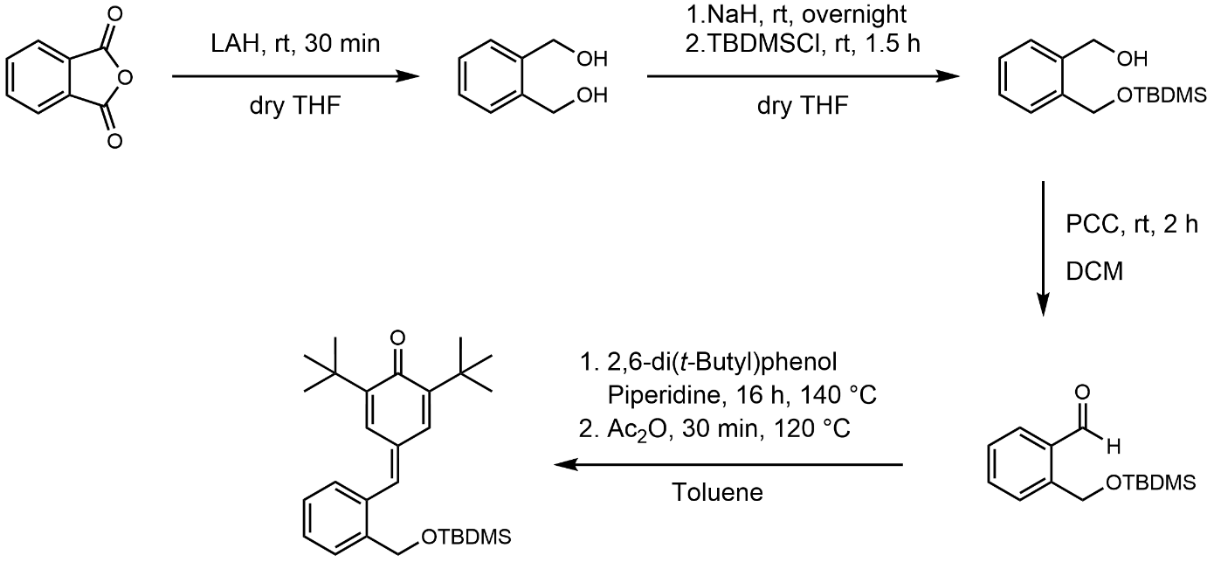

3.2 General Procedure B for the Synthesis of Diols

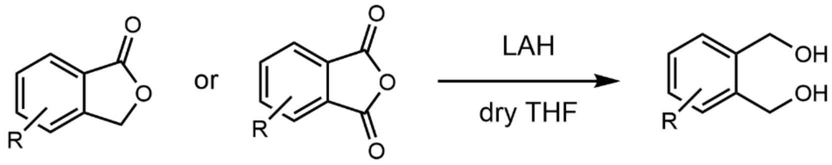

With sight adaptions to literature [4], the corresponding phthalide or phthalic acid (1 eq) was dissolved in anhydrous THF (0.75 mol L^{-1}). and cooled to 0 °C. LAH (1.5 eq in case of phthalides and 2 eq in case of anhydrides) was added portionwise over a period of 30 min. After complete addition, the mixture was stirred at r.t. for 2 h after which it was cooled to 0 °C again and a Fieser workup was performed. The mixture was diluted with Et_2O and water (1 mL per g of LAH) was added dropwise cautiously, followed by 15% NaOH (1 mL per g LAH) and water again (3 mL per g of LAH). Na_2SO_4 was added and the mixture was further stirred at r.t. for 15 min. It was filtered over a thoroughly packed pad of Celite, thoroughly washed with Et_2O, and the filtrate was concentrated. The obtained diols **4**$^{\mathbf{Diol}}$ were used in the next step without further purification.

3.3 General Procedure C for the Monoprotection of Diols

Remark: In principle, all diols were monoprotected using the same procedure. For the unsubstituted diol and symmetrically substituted diols, exclusively the monoprotected product was formed. For asymmetrically substituted diols however, both possible monoprotection products were obtained in a roughly 50:50 ratio. The products were separable by column chromatography upon loss of material in mixed fractions.

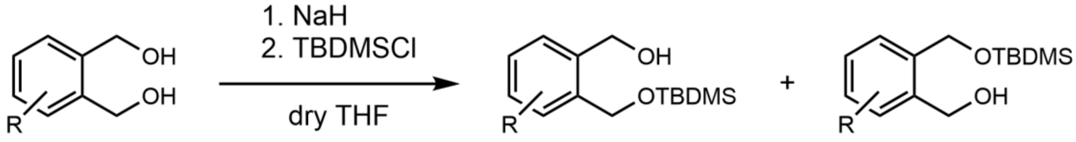

In analogy to a known procedure [5], NaH (90%, 1 eq) was suspended in anhydrous THF (0.15 mol L^{-1}). To this suspension, a solution of the respective diol (1 eq) in anhydrous THF (0.5 mol L^{-1}) was added dropwise over a period of 30 min. The mixture was stirred at r.t. overnight. It was cooled to 0 °C and TBDMSCl (1 eq) was added in one portion. The mixture was stirred at r.t. for 1.5 h and then quenched by the addition of a saturated NH_4Cl solution. It was extracted with Et_2O trice. The combined organic phases were washed with brine, dried with Na_2SO_4, filtered and concentrated. The obtained monoprotected diols **4**$^{\mathbf{prot.diol}}$ were used in the next step without further purification (symmetrical diols) or purified/separated by column chromatography (asymmetric diols) as stated for the respective compounds.

3.4 General Procedure D for the Synthesis of Aldehydes

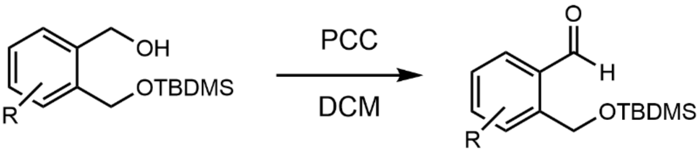

In analogy to literature [6], the corresponding monoprotected diol (1 eq) was dissolved in DCM (0.4 mol L^{-1}) and PCC (2 eq) was added portionwise over a period of 15 min. The resulting dark suspension was stirred for 2 h at r.t. and then filtered over a silica plug and washed thoroughly with DCM. The filtrate was concentrated on the rotary evaporator to give the protected aldehydes **4**$^{\mathbf{Aldehyde}}$ which were used without further purification.

In case of bad shim in crude NMR spectra (caused by remaining chromium species) and/or apparent presence of such in the concentrated filtrate, it was redissolved in DCM and extracted with a dil. aqueous solution of ascorbic acid until no greenish coloring of the aqueous phase was observable anymore.

3.5 General Procedure E for the Synthesis of Quinone Methides

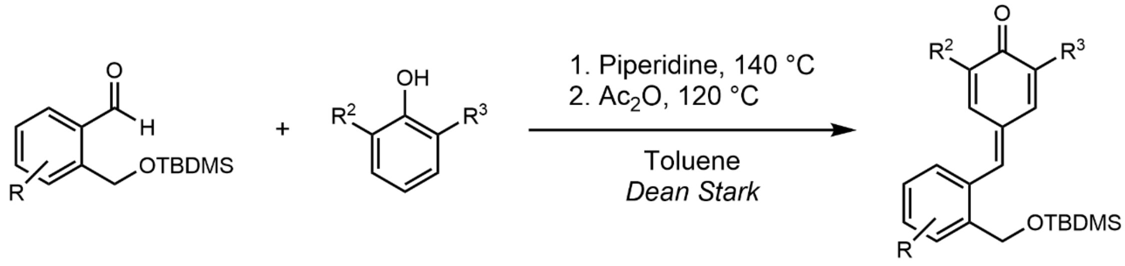

In analogy to the synthesis of standard quinone methides [7], the corresponding aldehyde (1 eq) and phenol (1.1 eq) were dissolved in toluene (0.32 mol L^{-1}) in a *Dean-Stark-Apparatus* and heated to 140 °C. Then, a solution of piperidine (2 eq) in toluene (0.9 mol L^{-1}) was added dropwise over a period of 30 min. After complete addition, the mixture was stirred at 140 °C overnight. It was cooled to 120 °C and Ac_2O (2 eq) was added dropwise and further stirred at 120 °C for 30 min. The mixture was poured on ice/water and the phases were separated. The aqueous phase was extracted with EtOAc trice. The combined organic layers were washed with brine, dried with Na_2SO_4 and concentrated. The crude quinone methides **4** were purified by column chromatography on silica with the given eluent and recrystallized from the given solvents as given for the respective compounds.

3.6 Deprotection of 4a and Subsequent Intramolecular Cyclization (Formation of 7a)

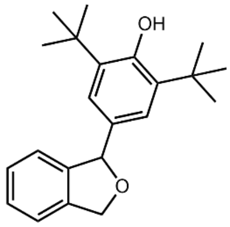

7a

As stated in the main manuscript, the deprotection of quinone methide **4a** did not lead to the formation of the free *o*-(CH_2)OH-*p*-QM **2a**. Instead, the free hydroxyl group immediately underwent 1,6-addition to the quinone methide moiety. Hence, rearomatization and the formation of dihydroisobenzofuran derivative **7a** was observable. The procedure for the synthesis of **7a** shall still be given here:

According to the literature procedure for the synthesis of *o*-OH-*p*-QMs [8], TBDMS protected quinone methide **4a** (0.44 g, 1.0 mmol) was dissolved in 10 mL anhydrous THF and cooled to 0 °C. To this solution, TBAF (1.0 M solution in THF, 1.1 mL, 1.1 mmol, 1.1 eq) was added dropwise via syringe. The mixture was stirred at 0 °C for 15 min after which it was quenched by the addition of 10 mL of an aq. conc. NH_4Cl solution. The phases were separated, and the aqueous phase was extracted with Et_2O three times. The combined organic phases were washed with brine, dried with Na_2SO_4, filtered, and concentrated on the rotary evaporator. The crude was purified by column chromatography on silica (heptanes/EtOAc 20/1) to give **7a** as a yellowish solid.

^{1}H-NMR (300 MHz, $CDCl_3$, 298 K) δ / ppm = 7.32-7.23 (m, 3 H, Ar-**H**), 7.11 (d, 2 H, Ar-**H**), 7.11-7.10 (m, 1 H, Ar-**H**), 6.13 (s, 1 H, -C**H**), 5.31 (dd, J_1 = 2.5 Hz, J_2 = 12.2 Hz, 1 H, -C**H₂**), 5.22 (s, 1 H, -O**H**), 5.17 (dd, J_1 = 1.9 Hz, J_2 = 12.2 Hz, 1 H, -C**H₂**), 1.42 (s, 18 H, -C**H₃**).

^{13}C-NMR (75 MHz, $CDCl_3$, 298 K) δ / ppm = 154.0 (1 C, $\mathbf{C_{Ar}}$), 142.1 (1 C, $\mathbf{C_{Ar}}$), 139.8 (1 C, $\mathbf{C_{Ar}}$), 136.0 (2 C, $\mathbf{C_{Ar}}$), 132.4 (1 C, $\mathbf{C_{Ar}}$), 127.5 (1 C, $\mathbf{C_{Ar}}$), 127.4 (1 C, $\mathbf{C_{Ar}}$), 124.3 (2 C, $\mathbf{C_{Ar}}$), 122.7 (1 C, $\mathbf{C_{Ar}}$), 121.0 (1 C, $\mathbf{C_{Ar}}$), 86.9 (1 C, -**C**H), 72.9 (1 C, -**C**H_2), 34.4 (2 C, -**C**(CH_3)$_3$), 30.4 (6 C, -C(**C**H_3)$_3$).

HRMS (ESI-TOF): *m/z:* $[M+H]^+$ calcd for $C_{22}H_{29}O_2^+$: 325.2162, found 325.2168.

Melting point: 110.0-115.6 °C

4. Starting Materials: Product Characterization

4.1 Esters

4-Nitrophenyl 2-phenylacetat (3a)

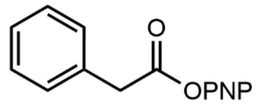

3a

Ester **3a** was synthesized according to general procedure **A** and obtained in a yield of 70% as a white solid after column chromatography on deactivated silica (DCM as eluent). Spectral data were in accordance with literature [9].

^{1}H-NMR (300 MHz, $CDCl_3$, 298 K) *δ* / ppm = 8.25 (d, *J* = 9.2 Hz, 2 H, Ar-**H**), 7.42-7.31 (m, 5 H, Ar-**H**), 7.27 (d, *J* = 9.2 Hz, 2 H, Ar-**H**), 3.92 (s, 2 H, -C**H**$_2$).

4-Nitrophenyl 2-(2-fluorophenyl)acetate (3b)

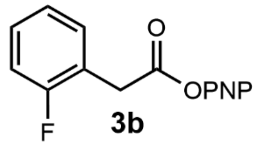

3b

Ester **3b** was synthesized according to general procedure **A** and obtained in a yield of 60% as a white solid after column chromatography on deactivated silica (DCM as eluent).

^{1}H-NMR (300 MHz, $CDCl_3$, 298 K) *δ* / ppm = 8.18 (d, *J* = 9.2 Hz, 2 H, Ar-**H**), 7.29-7.18 (m, 2 H, Ar-**H**), 7.22 (d, *J* = 9.2 Hz, 2 H, Ar-**H**), 7.12-7.02 (m, 2 H, Ar-**H**), 3.88 (s, 2 H, -C**H**$_2$).

^{19}F-NMR (282 MHz, CDCl3, 298 K) *δ* / ppm = -62.7 (3 F, Ar-**F**).

^{13}C-NMR (75 MHz, $CDCl_3$, 298 K) *δ* / ppm = 168.4 (1 C, -**C**OOR), 161.2 (d, *J* = 246.7 Hz, 1 C, -**C**$_{Ar}$-F), 155.5 (1 C, **C**$_{Ar}$), 145.5 (1 C, **C**$_{Ar}$), 131.5 (d, *J* = 3.8 Hz, 1 C, **C**$_{Ar}$), 129.9 (d, *J* = 8.2 Hz, 1 C, **C**$_{Ar}$), 125.3 (2 C, **C**$_{Ar}$), 124.5 (d, *J* = 4.1 Hz, 1 C, **C**$_{Ar}$), 122.5 (2 C, **C**$_{Ar}$), 120.3 (d, *J* = 15.9 Hz, 1 C, **C**$_{Ar}$), 115.7 (d, *J* = 21.5 Hz, 1 C, **C**$_{Ar}$), 34.9 (d, *J* = 3.5 Hz, 1 C, -**C**H_2).

HRMS (ESI-TOF): *m/z:* $[M+Na]^+$ calcd for $C_{14}H_{10}FNNaO_4^+$: 298.0486, found 298.0490.

Melting point: 57.7-59.7 °C

4-Nitrophenyl 2-(4-fluorophenyl)acetate (3c)

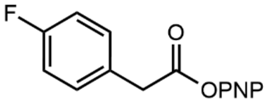

3c

Ester **3c** was synthesized according to general procedure **A** and obtained in a yield of 67% as an off-white solid after column chromatography on deactivated silica (DCM as eluent). Spectral data were in accordance with literature [3].

^{1}H-NMR (300 MHz, $CDCl_3$, 298 K) *δ* / ppm = 8.26 (d, *J* = 9.2 Hz, 2 H, Ar-**H**), 7.35 (dd, J_1 = 5.3 Hz, J_2 = 8.7 Hz, 2 H, Ar-**H**), 7.26 (d, *J* = 9.2 Hz, 2 H, Ar-**H**), 7.08 (t, *J* = 8.7 Hz, 2 H, Ar-**H**), 3.88 (s, 2 H, -C**H**$_2$).

^{19}F-NMR (282 MHz, CDCl3, 298 K) *δ* / ppm = -114.6 (3 F, Ar-**F**).

4-Nitrophenyl 2-(3-chlorophenyl)acetate (3d)

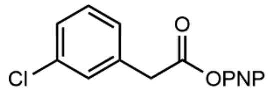

3d

Ester **3d** was synthesized according to general procedure **A** and obtained in a yield of 56% as a white solid after column chromatography on deactivated silica (DCM as eluent).

^{1}H-NMR (300 MHz, $CDCl_3$, 298 K) *δ* / ppm = 8.26 (d, *J* = 9.2 Hz, 2 H, Ar-**H**), 7.38 (br. s, 1 H, Ar-**H**), 7.33-7.31 (m, 2 H, Ar-**H**), 7.28-7.24 (m, 1 H, Ar-**H**), 7.27 (d, *J* = 9.2 Hz, 2 H, Ar-**H**), 3.88 (s, 2 H, -C**H₂**).

^{13}C-NMR (75 MHz, $CDCl_3$, 298 K) *δ* / ppm = 168.6 (1 C, -**C**OOR), 155.3 (1 C, $\mathbf{C_{Ar}}$), 145.6 (1 C, $\mathbf{C_{Ar}}$), 134.8 (1 C, $\mathbf{C_{Ar}}$), 134.5 (1 C, $\mathbf{C_{Ar}}$), 130.2 (1 C, $\mathbf{C_{Ar}}$), 129.6 (1 C, $\mathbf{C_{Ar}}$), 128.1 (1 C, $\mathbf{C_{Ar}}$), 127.6 (1 C, $\mathbf{C_{Ar}}$), 125.3 (2 C, $\mathbf{C_{Ar}}$), 122.4 (2 C, $\mathbf{C_{Ar}}$), 40.9 (1 C, -**C**H_2).

HRMS (ESI-TOF): *m/z:* $[M+Na]^+$ calcd for $C_{14}H_{10}ClNNaO_4^+$: 314.0191, found 314.0196.

Melting point: 42.5-45.6 °C

4-Nitrophenyl 2-(4-chlorophenyl)acetate (3e)

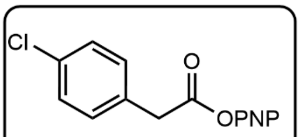

3e

Ester **3e** was synthesized according to general procedure **A** and obtained in a yield of 67% as an off-white solid after column chromatography on deactivated silica (DCM as eluent). Spectral data were in accordance with literature [3].

^{1}H-NMR (300 MHz, $CDCl_3$, 298 K) *δ* / ppm = 8.26 (d, *J* = 9.2 Hz, 2 H, Ar-**H**), 7.36 (d, *J* = 8.6 Hz, 2 H, Ar-**H**), 7.31 (d, *J* = 8.6 Hz, 2 H, Ar-**H**), 7.26 (d, *J* = 9.2 Hz 2 H, Ar-**H**), 3.97 (s, 2 H, -C$\mathbf{H_2}$).

4-Nitrophenyl 2-(3,4-dichlorophenyl)acetate (3f)

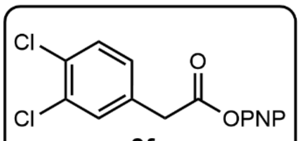

3f

Ester **3f** was synthesized according to general procedure **A** and obtained in a yield of 49% as a slightly yellow solid after column chromatography on deactivated silica (DCM as eluent).

^{1}H-NMR (300 MHz, $CDCl_3$, 298 K) *δ* / ppm = 8.27 (d, *J* = 9.2 Hz, 2 H, Ar-**H**), 7.49-7.45 (m, 2 H, Ar-**H**), 7.29-7.20 (m, 1 H, Ar-H), 7.27 (d, *J* = 9.2 Hz, 2 H, Ar-**H**), 3.87 (s, 2 H, Ar-**H**).

^{13}C-NMR (75 MHz, $CDCl_3$, 298 K) *δ* / ppm = 168.3 (1 C, -**C**OOR), 155.2 (1 C, $\mathbf{C_{Ar}}$), 145.7 (1 C, $\mathbf{C_{Ar}}$), 133.1 (1 C, $\mathbf{C_{Ar}}$), 132.7 (1 C, $\mathbf{C_{Ar}}$), 133.2 (1 C, $\mathbf{C_{Ar}}$), 131.5 (1 C, $\mathbf{C_{Ar}}$), 130.9 (1 C, $\mathbf{C_{Ar}}$), 128.9 (1 C, $\mathbf{C_{Ar}}$), 125.4 (2 C, $\mathbf{C_{Ar}}$), 122.4 (2 C, $\mathbf{C_{Ar}}$), 40.3 (1 C, -**C**H_2).

HRMS (ESI-TOF): *m/z: :* [M+Na]$^+$ calcd for $C_{14}H_9Cl_2NNaO_4^+$: 347.9801, found 347.9804.

Melting point: 94.5-97.6 °C

4-Nitrophenyl 2-(4-bromophenyl)acetate (3g)

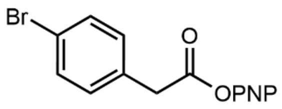

3g

Ester **3g** was synthesized according to general procedure **A** and obtained in a yield of 70% as an off-white solid after column chromatography on deactivated silica (DCM as eluent). Spectral data were in accordance with literature [3].

^{1}H-NMR (300 MHz, $CDCl_3$, 298 K) δ / ppm = 8.25 (d, J = 9.2 Hz, 2 H, Ar-**H**), 7.51 (d, J = 8.4 Hz, 2 H, Ar-**H**), 7.23-7.26 (m, 4 H, Ar-**H**), 3.85 (s, 2 H, -C**H₂**).

4-Nitrophenyl 2-(3-iodophenyl)acetate (3h)

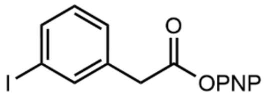

3h

Ester **3h** was synthesized according to general procedure **A** and obtained in a yield of 64% as a white solid after column chromatography on deactivated silica (DCM as eluent).

^{1}H-NMR (300 MHz, $CDCl_3$, 298 K) δ / ppm = 8.26 (d, J = 9.2 Hz, 2 H, Ar-**H**), 7.74 (s, 1 H, Ar-**H**), 7.68 (d, J = 7.9 Hz, 1 H, Ar-**H**), 7.35 (d, J = 7.9 Hz, 1 H, Ar-**H**), 7.27 (d, J = 9.2 Hz, 2 H, Ar-**H**), 7.13 (t, J = 7.8 Hz, 1 H, Ar-**H**), 3.85 (s, 2 H, -C**H**$_2$).

^{13}C-NMR (75 MHz, $CDCl_3$, 298 K) δ / ppm = 168.6 (1 C, -**C**OOR), 155.3 (1 C, $\mathbf{C_{Ar}}$), 145.6 (1 C, $\mathbf{C_{Ar}}$), 138.4 (1 C, $\mathbf{C_{Ar}}$), 136.9 (1 C, $\mathbf{C_{Ar}}$), 134.9 (1 C, $\mathbf{C_{Ar}}$), 130.6 (1 C, $\mathbf{C_{Ar}}$), 128.7 (1 C, $\mathbf{C_{Ar}}$), 125.3 (2 C, $\mathbf{C_{Ar}}$), 122.4 (2 C, $\mathbf{C_{Ar}}$), 95.0 (1 C, $\mathbf{C_{Ar}}$-I), 40.7 (1 C, -**C**H_2).

HRMS (ESI-TOF): *m/z:* [M+Na]$^+$ calcd for $C_{14}H_{10}INNaO_4^+$: 405.9547, found 405.9549.

Melting point: 52.0-54.3 °C

4-Nitrophenyl 2-(4-methoxyphenyl)acetate (3i)

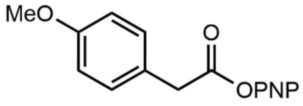

3i

Ester **3i** was synthesized according to general procedure **A** and obtained in a yield of 61% as an off-white solid after column chromatography on deactivated silica (DCM as eluent). Spectral data were in accordance with literature [10].

^{1}H-NMR (300 MHz, $CDCl_3$, 298 K) δ / ppm = 8.25 (d, J = 9.1 Hz, 2 H, Ar-**H**), 7.29 (d, J = 8.7 Hz, 2 H, Ar-**H**), 7.25 (d, J = 9.1 Hz, 2 H, Ar-**H**), 6.91 (d, J = 8.7 Hz, 2 H, Ar-**H**), 3.83 (s, 2 H, -C**H**$_2$), 3.82 (3 H, -OC**H**$_3$).

4-Nitrophenyl 2-(3,4-dimethoxyphenyl)acetate (3j)

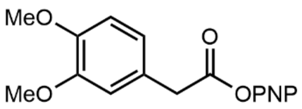

3j

Ester **3j** was synthesized according to general procedure **A** and obtained in a yield of 70% as an off-white solid after column chromatography on deactivated silica (DCM as eluent). Spectral data were in accordance with literature [3].

^{1}H-NMR (300 MHz, $CDCl_3$, 298 K) δ / ppm = 8.26 (d, J = 9.2 Hz, 2 H, Ar-**H**), 7.26 (d, J = 9.2 Hz, 2 H, Ar-**H**), 6.93-6.86 (m, 3 H, Ar-**H**), 3.90 (s, 3 H, -OC**H₃**), 3.89 (s, 3 H, -OC**H₃**), 3.84 (s, 2 H, -C**H₂**).

4-Nitrophenyl 2-(4-(methylthio)phenyl)acetate (3k)

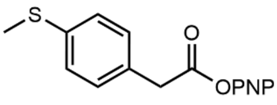

3k

Ester **3k** was synthesized according to general procedure **A** and obtained in a yield of 59% as a white solid after column chromatography on deactivated silica (DCM as eluent).

^{1}H-NMR (300 MHz, $CDCl_3$, 298 K) δ / ppm = 8.25 (d, J = 9.2 Hz, 2 H, Ar-**H**), 7.31-7.24 (m, 6 H, Ar-**H**), 3.85 (s, 2 H, -C**H₂**), 2.50 (s, 3 H, -C**H₃**).

^{13}C-NMR (75 MHz, $CDCl_3$, 298 K) δ / ppm = 169.1 (1 C, -**C**OOR), 155.5 (1 C, $\mathbf{C_{Ar}}$), 145.5 (1 C, $\mathbf{C_{Ar}}$), 138.3 (1 C, $\mathbf{C_{Ar}}$), 129.9 (2 C, $\mathbf{C_{Ar}}$), 129.4 (1 C, $\mathbf{C_{Ar}}$), 127.1 (2 C, $\mathbf{C_{Ar}}$), 125.3 (2 C, $\mathbf{C_{Ar}}$), 122.5 (2 C, $\mathbf{C_{Ar}}$), 40.9 (1 C, -**C**H_2), 15.9 (1 C, -S**C**H_3).

HRMS (ESI-TOF): *m/z:* $[M+Na]^+$ calcd for $C_{15}H_{13}NNaO_4S$: 326.0457, found 326.0457.

Melting point: 110.0-112.0 °C

4-Nitrophenyl 2-(*o*-tolyl)acetate (3l)

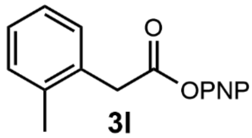

3l

Ester **3l** was synthesized according to general procedure **A** and obtained in a yield of 65% as a white solid after column chromatography on deactivated silica (DCM as eluent). Spectral data were in accordance with literature [10].

^{1}H-NMR (300 MHz, $CDCl_3$, 298 K) δ / ppm = 8.25 (d, J = 9.2 Hz, 2 H, Ar-**H**), 7.20-7.31 (m, 6 H, Ar-**H**), 3.92 (s, 2 H, -C**H₂**), 2.41 (s, 3 H, -C**H₃**).

4-Nitrophenyl 2-(*m*-tolyl)acetate (3m)

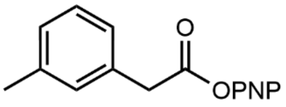

3m

Ester **3m** was synthesized according to general procedure **A** and obtained in a yield of 65% as a colorless oil after column chromatography on deactivated silica (DCM as eluent). Spectral data were in accordance with literature [10].

^{1}**H-NMR** (300 MHz, $CDCl_3$, 298 K) *δ* / ppm = 8.25 (d, *J* = 9.2 Hz, 2 H, Ar-**H**), 7.31-7.25 (m, 1 H, Ar-**H**), 7.27 (d, *J* = 9.2 Hz, 2 H, Ar-**H**), 7.19-7.14 (m, 3 H, Ar-**H**), 3.87 (s, 2 H, -C**H$_2$**), 2.38 (s, 3 H, -C**H$_3$**).

4-Nitrophenyl 2-(*p*-tolyl)acetate (3n)

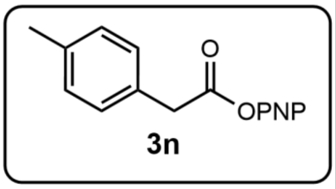

Ester **3n** was synthesized according to general procedure **A** and obtained in a yield of 63% as a white solid after column chromatography on deactivated silica (DCM as eluent). Spectral data were in accordance with literature [10].

^{1}**H-NMR** (300 MHz, $CDCl_3$, 298 K) *δ* / ppm = 8.25 (d, *J* = 9.2 Hz, 2 H, Ar-**H**), 7.28-7.23 (m, 2 H, Ar-**H**), 7.26 (d, *J* = 9.2 Hz, 2 H, Ar-**H**), 7.21-7.18 (m, 2 H, Ar-**H**), 3.86 (s, 2 H, -C**H$_2$**), 2.36 (s, 3 H, -C**H$_3$**).

4-Nitrophenyl 2-(3,5-dimethylphenyl)acetate (3o)

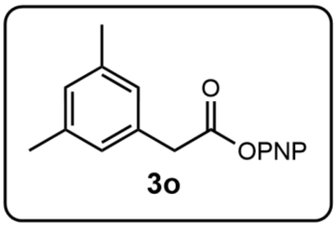

Ester **3o** was synthesized according to general procedure **A** and obtained in a yield of 81% as a white solid after column chromatography on deactivated silica (DCM as eluent).

^{1}**H-NMR** (300 MHz, $CDCl_3$, 298 K) *δ* / ppm = 8.25 (d, *J* = 9.2 Hz, 2 H, Ar-**H**), 7.27 (d, *J* = 9.2 Hz), 6.99 (s, 2 H, Ar-**H**), 6.97 (s, 1 H, Ar-**H**), 3.82 (s, 2 H, -C**H$_2$**, 2.34 (s, 6 H, -C**H$_3$**).

^{13}**C-NMR** (75 MHz, $CDCl_3$, 298 K) *δ* / ppm = 169.4 (1 C, -**C**OOR), 155.6 (1 C, **C$_{Ar}$**), 145.4 (1 C, **C$_{Ar}$**), 138.6 (2 C, **C$_{Ar}$**), 132.5 (1 C, **C$_{Ar}$**), 129.4 (1 C, **C$_{Ar}$**), 127.2 (2 C, **C$_{Ar}$**), 125.3 (2 C, **C$_{Ar}$**), 122.5 (2 C, **C$_{Ar}$**), 42.3 (1 C, -**C**H$_2$), 21.4 (2 C, -**C**H$_3$).

HRMS (ESI-TOF): *m/z:* $[M+NH_4]^+$ calcd for $C_{16}H_{19}N_2O_4^+$: 303.1339, found 303.1346.

Melting point: 51.6-52.3 °C

4-Nitrophenyl 2-(4-(tert-butyl)phenyl)acetate (3p)

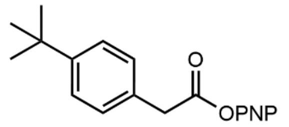

3p

Ester **3p** was synthesized according to general procedure **A** and obtained in a yield of 57% as a white solid after column chromatography on deactivated silica (DCM as eluent).

^{1}H-NMR (300 MHz, CDCl$_3$, 298 K) δ / ppm = 8.25 (d, J = 9.2 Hz, 2 H, Ar-**H**), 7.41 (d, J = 8.4 Hz, 2 H, Ar-**H**), 7.30 (d, J = 8.4 Hz, 2 H, Ar-**H**), 7.27 (d, J = 9.2 Hz, 2 H, Ar-**H**), 3.87 (s, 2 H, -C**H$_2$**), 1.33 (s, 9 H, -C**H$_3$**).

^{13}C-NMR (75 MHz, CDCl$_3$, 298 K) δ / ppm = 169.4 (1 C, -**C**OOR), 155.6 (1 C, **C$_{Ar}$**), 150.8 (1 C, **C$_{Ar}$**), 145.5 (1 C, **C$_{Ar}$**), 129.7 (1 C, **C$_{Ar}$**), 129.1 (2 C, **C$_{Ar}$**), 126.0 (2 C, **C$_{Ar}$**), 125.3 (2 C, **C$_{Ar}$**), 122.5 (2 C, **C$_{Ar}$**), 40.9 (1 C, -**C**H$_2$), 34.7 (1 C, -**C**(CH$_3$)$_3$), 31.4 (3 C, -C(**C**H$_3$)$_3$).

HRMS (ESI-TOF): *m/z:* $[M+NH_4]^+$ calcd for $C_{18}H_{23}N_2O_4^+$: 331.1652, found 331.1653.

Melting point: 65.5-67.0 °C

4-Nitrophenyl 2-([1,1'-biphenyl]-4-yl)acetate (3q)

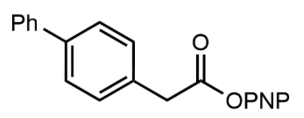

3q

Ester **3q** was synthesized according to general procedure **A** and obtained in a yield of 63% as a yellow solid after column chromatography on deactivated silica (DCM as eluent). Spectral data were in accordance with literature [10].

^{1}H-NMR (300 MHz, CDCl$_3$, 298 K) δ / ppm = 8.26 (d, J = 9.2 Hz, 2 H, Ar-**H**), 7.63-7.59 (m, 4 H, Ar-**H**), 7.48-7.43 (m, 4 H, Ar-**H**), 7.39-7.34 (m, 1 H, Ar-**H**), 7.29 (d, J = 9.2 Hz, 2 H, Ar-**H**), 3.95 (s, 2 H, -C**H$_2$**).

4-Nitrophenyl 2-(3-(trifluoromethyl)phenyl)acetate (3r)

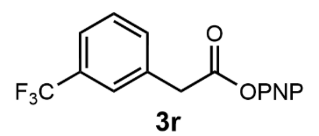

3r

Ester **3r** was synthesized according to general procedure **A** and obtained in a yield of 65% as a white solid after column chromatography on deactivated silica (DCM as eluent).

^{1}H-NMR (300 MHz, CDCl$_3$, 298 K) δ / ppm = 8.28 (d, J = 9.2 Hz, 2 H, Ar-**H**), 7.66-7.51 (m, 4 H, Ar-**H**), 7.29 (d, J = 9.2 Hz, 2 H, Ar-**H**), 3.99 (s, 2 H, -C**H$_2$**).

^{19}F-NMR (282 MHz, CDCl3, 298 K) δ / ppm = -62.7 (3 F, -C**F$_3$**).

^{13}C-NMR (75 MHz, CDCl$_3$, 298 K) δ / ppm = 168.5 (1 C, -**C**OOR), 155.3 (1 C, **C$_{Ar}$**), 145.6 (1 C, **C$_{Ar}$**), 132.9 (1 C, **C$_{Ar}$**), 132.9 (1 C, **C$_{Ar}$**), 131.4 (q, J = 32.5, 1 C, -**C**F$_3$), 129.5 (1 C, **C$_{Ar}$**),

HRMS (ESI-TOF): *m/z:* $[M+Na]^+$ calcd for $C_{15}H_{10}F_3NNaO_4^+$: 348.0454, found 348.0452.

Melting point: 61.1-65.0 °C

4-Nitrophenyl 2-(4-(trifluoromethyl)phenyl)acetate (3s)

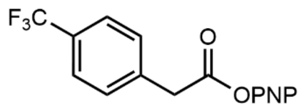

3s

Ester **3s** was synthesized according to general procedure **A** and obtained in a yield of 55% as a white solid after column chromatography on deactivated silica (DCM as eluent). Spectral data were in accordance with literature [10].

^{1}H-NMR (300 MHz, $CDCl_3$, 298 K) *δ* / ppm = 8.27 (d, *J* = 9.1 Hz, 2 H, Ar-**H**), 7.66 (d, *J* = 8.1 Hz, 2 H, Ar-**H**), 7.51 (d, *J* = 8.1 Hz, 2 H, Ar-**H**), 7.27 (d, *J* = 9.1 Hz, 2 H, Ar-**H**), 3.97 (s, 2 H, -C**H_2**).

^{19}F-NMR (282 MHz, CDCl3, 298 K) *δ* / ppm = -62.7 (3 F, -C**F_3**).

4-Nitrophenyl 2-(4-nitrophenyl)acetate (3t)

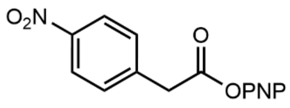

3t

Ester **3t** was synthesized according to general procedure **A** and obtained in a yield of 19% as an orange, not perfectly pure solid after column chromatography on deactivated silica (DCM as eluent). Due to degradation on the column, the ester was not further purified and used as obtained after the first column.

^{1}H-NMR (300 MHz, $CDCl_3$, 298 K) *δ* / ppm = 8.28 (d, *J* = 9.2 Hz, 2 H, Ar-**H**), 8.26 (d, *J* = 8.7 Hz, 2 H, Ar-**H**), 7.57 (d, *J* = 8.7 Hz, 2 H, Ar-**H**), 7.28 (d, *J* = 9.2 Hz, 2 H, Ar-**H**), 4.03 (s, 2 H, -C**H_2**).

^{13}C-NMR (75 MHz, $CDCl_3$, 298 K) *δ* / ppm = 168.0 (1 C, -**C**OOR), 155.1 (1 C, **C_{Ar}**), 147.8 (1 C, **C_{Ar}**), 143.7 (1 C, **C_{Ar}**), 139.9 (1 C, **C_{Ar}**), 130.5 (2 C, **C_{Ar}**), 125.5 (2 C, **C_{Ar}**), 124.2 (2 C, **C_{Ar}**), 122.4 (2 C, **C_{Ar}**), 41.0 (1 C, -**C**H_2).

HRMS (ESI-TOF): *m/z: :* $[M+Na]^+$ calcd for $C_{14}H_{10}N_2NaO_6^+$: 325.0431, found 325.0512.

Melting point: 116.2-118.1 °C

4-Nitrophenyl 2-(3-cyanophenyl)acetate (3u)

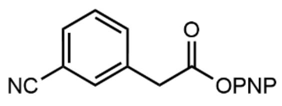

3u

Ester **3u** was synthesized according to general procedure **A** and obtained in a yield of 28% as a slightly yellow solid after column chromatography on deactivated silica (DCM as eluent).

^{1}H-NMR (300 MHz, $CDCl_3$, 298 K) *δ* / ppm = 8.28 (d, *J* = 9.2 Hz, 2 H, Ar-**H**), 7.69-7.62 (m, 3 H, Ar-**H**), 7.52 (t, *J* = 7.7 Hz, 1 H, Ar-**H**), 7.28 (d, *J* = 9.2 Hz, 2 H, Ar-**H**), 3.96 (s, 2 H, -C**H_2**).

^{13}C-NMR (75 MHz, $CDCl_3$, 298 K) *δ* / ppm = 168.2 (1 C, -**C**OOR), 155.1 (1 C, $\mathbf{C_{Ar}}$), 145.7 (1 C, $\mathbf{C_{Ar}}$), 134.2 (1 C, $\mathbf{C_{Ar}}$), 134.0 (1 C, $\mathbf{C_{Ar}}$), 133.1 (1 C, $\mathbf{C_{Ar}}$), 131.6 (1 C, $\mathbf{C_{Ar}}$), 129.9 (1 C, $\mathbf{C_{Ar}}$), 125.4 (2 C, $\mathbf{C_{Ar}}$), 122.4 (2 C, $\mathbf{C_{Ar}}$), 118.5 (1 C, -**C**N), 113.3 (1 C, $\mathbf{C_{Ar}}$), 40.7 (1 C, -**C**H_2).

HRMS (ESI-TOF): *m/z:* $[M+Na]^+$ calcd for $C_{15}H_{10}N_2NaO_4^+$: 305.0533, found 305.0537.

Melting point: 103.5-105.6 °C

4-Nitrophenyl 2-(naphthalen-1-yl)acetate (3v)

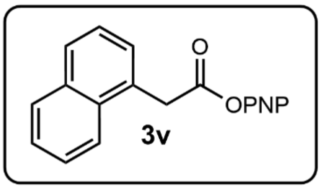

Ester **3v** was synthesized according to general procedure **A** and obtained in a yield of 78% as a white solid after column chromatography on deactivated silica (DCM as eluent). Spectral data were in accordance with literature [10].

^{1}H-NMR (300 MHz, $CDCl_3$, 298 K) *δ* / ppm = 8.21 (d, *J* = 9.2 Hz, 2 H, Ar-**H**), 8.07 (d, *J* = 8.3 Hz, 2 H, Ar-**H**), 7.92 (d, *J* = 8.1 Hz, 1 H, Ar-**H**), 7.86 (d, *J* = 8.1 Hz, 1 H, Ar-**H**), 7.63-7.46 (m, 4 H, Ar-**H**), 7.20 (d, *J* = 9.2 Hz, 2 H, Ar-**H**), 4.35 (s, 2 H, -C$\mathbf{H_2}$).

4-Nitrophenyl 2-(naphthalen-2-yl)acetate (3w)

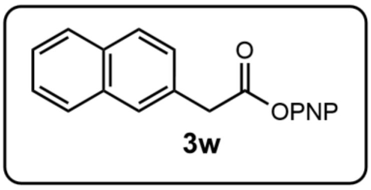

Ester **3w** was synthesized according to general procedure **A** and obtained in a yield of 69% as a white solid after column chromatography on deactivated silica (DCM as eluent). Spectral data were in accordance with literature [10].

^{1}H-NMR (300 MHz, $CDCl_3$, 298 K) *δ* / ppm = 8.25 (d, *J* = 9.2 Hz, 2 H, Ar-**H**), 7.89-7.84 (m, 4 H, Ar-**H**), 7.52-7.49 (m, 3 H, Ar-**H**), 7.26 (d, *J* = 9.2 Hz, 2 H, Ar-**H**), 4.07 (s, 2 H, -C$\mathbf{H_2}$).

4-Nitrophenyl 2-(thiophen-3-yl)acetate (3x)

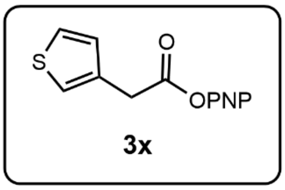

Ester **3x** was synthesized according to general procedure **A** and obtained in a yield of 47% as a slightly yellow solid after column chromatography on deactivated silica (DCM as eluent). Spectral data were in accordance with literature [3].

^{1}H-NMR (300 MHz, $CDCl_3$, 298 K) *δ* / ppm = 8.26 (d, *J* = 9.2 Hz, 2 H, Ar-**H**), 7.36 (dd, J_1 = 3.0 Hz, J_2 = 4.9 Hz, 1 H, Ar-**H**), 7.31-7.25 (m, 1 H, Ar-**H**), 7.28 (d, *J* = 9.2 Hz, 2 H, Ar-**H**), 7.13 (dd, J_1 = 1.3 Hz, J_2 = 4.9 Hz, 1 H, Ar-**H**), 3.95 (s, 2 H, -C$\mathbf{H_2}$).

4.2 Diols

1,2-Phenylenedimethanol (4^{diol})

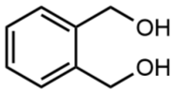

4^{diol}

Diol **4^{diol}** was synthesized from the phthalic anhydride according to general procedure **B** and obtained in a yield of 96% as a white solid. Spectral data were in accordance with literature [11].

^{1}H-NMR (300 MHz, $CDCl_3$, 298 K) *δ* / ppm = 7.31 (m (app. s), 4 H, Ar-**H**), 4.66 (s, 4 H, -C**H$_2$**), 3.50 (br. s, 2 H, -O**H**).

(4-Fluoro-1,2-phenylene)dimethanol (4a^{diol})

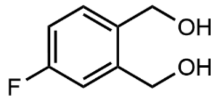

4a^{diol}

Diol **4a^{diol}** was synthesized from the phthalic anhydride according to general procedure **B** and obtained in a yield of 90% as a colorless oil. Spectral data were in accordance with literature [12].

^{1}H-NMR (300 MHz, $CDCl_3$, 298 K) *δ* / ppm = 7.30 (dd, J_1 = 5.6 Hz, J_2 = 8.4 Hz, 1 H, Ar-**H**), 7.08 (dd, J_1 = 2.7 Hz, J_2 = 9.3 Hz, 1 H, Ar-**H**), 7.00 (td, J_1 = 2.7 Hz, J_2 = 8.4 Hz, 1 H, Ar-**H**), 4.64 (s, 2 H, -C**H$_2$**OH), 4.62 (s, 2 H, -C**H$_2$**OH), 3.65 (br. s, 2H, -O**H**).

(4-Chloro-1,2-phenylene)dimethanol (4b^{diol})

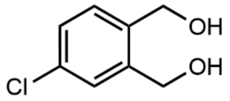

4b^{diol}

Diol **4b^{diol}** was synthesized from the phthalic anhydride according to general procedure **B** and obtained in a yield of 89% as a colorless oil. Spectral data were in accordance with literature [13].

^{1}H-NMR (300 MHz, $CDCl_3$, 298 K) *δ* / ppm = 7.31 (s, 1 H, Ar-**H**), 7.26-7.25 (m, 2 H, Ar-**H**), 4.61 (s, 4H, -C**H$_2$**), 3.50 (br., 2 H, -O**H**).

Naphthalene-2,3-diyldimethanol (4c^{diol})

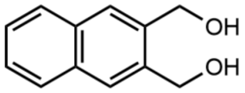

4c^{diol}

Diol **4c^{diol}** was synthesized from the phthalic anhydride according to general procedure **B** and obtained in a yield of 69% as a white solid. Spectral data were in accordance with literature [14].

^{1}H-NMR (300 MHz, $CDCl_3$, 298 K) *δ* / ppm = 7.85-7.82 (m, 4 H, Ar-**H**), 7.55-7.45 (m, 2 H, Ar-**H**), 4.92 (s, 4 H, -C**H$_2$**OH), 1.92 (br. s, 2 H, -O**H**).

4.3 Monoprotected Diols

(2-(((tert-butyldimethylsilyl)oxy)methyl)phenyl)methanol 4a$^{prot. diol}$

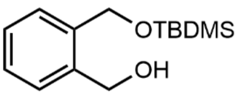

4a$^{prot. diol}$

Monoprotected diol **4a$^{prot. diol}$** was synthesized from the corresponding diol according to general procedure **C** and obtained in quantitative yield as a colourless oil. Spectral data was in accordance with literature [5].

^{1}H-NMR (300 MHz, $CDCl_3$, 298 K) *δ* / ppm = 7.38-7.31 (m, 4 H, Ar-**H**), 4.83 (s, 2 H, -C**H$_2$**OTBDMS), 4.69 (2 H, -C**H$_2$**OH), 3.35 (br. s, 1 H, -O**H**), 0.95 (s, 9 H, -SiC(C**H$_3$**)$_3$, 0.15 (s, 6 H,- SiC**H$_3$**).

4- and 5-F monoprotected phthalic alcohols

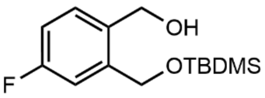

4ab$^{prot diol}$

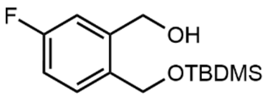

4aa$^{prot diol}$

Diols **4aa$^{prot. diol}$** and **4ab$^{prot. diol}$** were synthesized from the corresponding diol according to general procedure **C** and obtained in a 50:50 mixture of both isomers (combined yield of 95%). The two compounds could be separated by column chromatography on silica using a slow gradient from heptanes/EtOAc 10/1 to 5/1 to 2/1. The pure 5-F (**4aa$^{prot. diol}$**) compound eluted first, followed by several mixed fractions and the pure 4-F (**4ab$^{prot. diol}$**) compound. Both compounds were isolated as colorless oils. The structures were assigned by looking at HSQC, HMBC and NOESY correlations.

(2-(((*t*-Butyldimethylsilyl)oxy)methyl)-4-fluorophenyl)methanol (4ab$^{prot. diol}$)

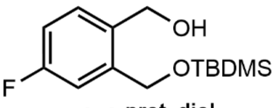

4ab$^{prot. diol}$

^{1}H-NMR (300 MHz, $CDCl_3$, 298 K) *δ* / ppm = 7.32 (dd, J_1 = 5.6 Hz, J_2 = 8.3 Hz, 1 H, Ar-**H**), 7.09 (dd, J_1 = 2.8 Hz, J_2 = 9.6 Hz, 1 H, Ar-**H**), 6.96 (td, J_1 = 2.8 Hz, J_2 = 8.3 Hz, 1 H, Ar-**H**), 4.78 (s, 2 H, -C**H$_2$**OTBMDS), 4.63 (d, *J* = 6.2 Hz, 2 H, -C**H$_2$**OH), 2.75 (t, *J* = 6.2 Hz, 1 H, -O**H**), 0.93 (s, 9 H, -SiC(C**H$_3$**)$_3$), 0.13 (s, 6 H, -SiC**H$_3$**).

^{13}C-NMR (75 MHz, $CDCl_3$, 298 K) *δ* / ppm = 162.5 (d, *J* = 246.3 Hz, 1 C, **C$_{Ar}$**-F), 141.6 (d, *J* = 7.1 Hz, 1 C, **C$_{Ar}$**), 135.0 (d, *J* = 3.3 Hz, 1 C, **C$_{Ar}$**), 131.0 (d, *J* = 8.2 Hz, 1 C, **C$_{Ar}$**), 115.5 (d, *J* = 22.1 Hz, 1 C, **C$_{Ar}$**), 114.4 (d, *J* = 20.9 Hz, 1 C, **C$_{Ar}$**), 63.8 (s, 1 C, -**C**H_2OTBDMS), 63.1 (s, 1 C, -**C**H_2OH), 26.0 (3 C, -SiC(**C**H_3)$_3$), 18.4 (1 C, -Si**C**(CH_3)$_3$), -5.1 (2 C, -Si**C**H_3).

^{19}F-NMR (282 MHz, CDCl3, 298 K) *δ* / ppm = -114.3 (1 F, Ar-**F**).

HRMS (ESI-TOF): *m/z:* $[M+Na]^+$ calcd for $C_{14}H_{23}FNaO_2Si^+$: 293.1344, found 293.1348.

(2-(((*t*-Butyldimethylsilyl)oxy)methyl)-5-fluorophenyl)methanol (4aa$^{prot. diol}$)

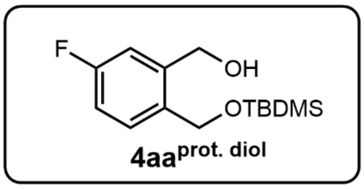

^{1}H-NMR (300 MHz, $CDCl_3$, 298 K) *δ* / ppm = 7.27 (dd, J_1 = 5.7 Hz, J_2 = 8.4 Hz, 1 H, Ar-**H**), 7.11 (dd, J_1 = 2.7 Hz, J_2 = 9.3 Hz, 1 H, Ar-**H**), 6.95 (td, J_1 = 2.7 Hz, J_2 = 8.4 Hz, 1 H, Ar-**H**), 4.74 (s, 2 H, -C**H$_2$**OTBDMS), 4.65 (d, *J* = 6.4 Hz, 2 H, -C**H$_2$**OH), 3.12 (t, *J* = 6.4 Hz, 1 H, -O**H**), 0.91 (s, 9 H, -SiC(C**H$_3$**)$_3$), 0.12 (s, 6 H, -SiC**H$_3$**).

^{13}C-NMR (75 MHz, $CDCl_3$, 298 K) *δ* / ppm = 162.6 (d, *J* = 246.6 H, **C$_{Ar}$**-F), 142.4 (d, *J* = 7.4 Hz, 1 C, **C$_{Ar}$**), 134.4 (d, *J* = 3.3 Hz, 1 C, **C$_{Ar}$**), 130.5 (d, *J* = 8.1 Hz, 1 C, **C$_{Ar}$**), 116.2 (d, *J* = 21.7 Hz, 1 C, **C$_{Ar}$**), 114.3 (d, *J* = 20.9 Hz, 1 C, **C$_{Ar}$**), 64.1 (1 C, -**C**H$_2$OTBDMS), 63.5 (d, *J* = 1.1 Hz, 1 C, -**C**H$_2$OH), 26.0 (3 C, -SiC(**C**H$_3$)$_3$), 18.4 (1 C, -Si**C**(CH$_3$)$_3$), -5.1 (2 C, -Si**C**H$_3$).

^{19}F-NMR (282 MHz, CDCl3, 298 K) *δ* / ppm = -114.3 (1 F, Ar-**F**).

HRMS (ESI-TOF): *m/z:* $[M+Na]^+$ calcd for $C_{14}H_{23}FNaO_2Si^+$: 293.1344, found 293.1343.

4- and 5-Cl monoprotected phthalic alcohols

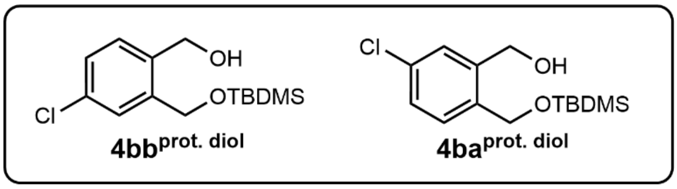

Diols **4ba$^{prot. diol}$** and **4bb$^{prot. diol}$** were synthesized from the corresponding diol according to general procedure **C** and obtained in a 50:50 mixture of both isomers (combined yield of 94%). The two compounds could be separated by column chromatography on silica using a slow gradient from heptanes/EtOAc 20/1 to 5/1. The pure 5-Cl **4ba$^{prot. diol}$** compound eluted first, followed by several mixed fractions and the pure 4-Cl **4bb$^{prot. diol}$** compound. Both compounds were isolated as colorless oils. The structures were assigned by looking at HSQC, HMBC and NOESY correlations.

(2-(((*t*-Butyldimethylsilyl)oxy)methyl)-4-chlorophenyl)methanol (4bb$^{\text{prot. diol}}$)

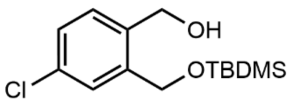

4bb$^{\text{prot. diol}}$

^{1}H-NMR (300 MHz, $CDCl_3$, 298 K) *δ* / ppm = 7.32-7.24 (m, 3 H, Ar-**H**), 4.73 (s, 2 H, -C**H_2**OTBDMS), 4.60 (s, 2 H, -C**H_2**OH), 2.92 (br. s, 1 H, -O**H**), 0.90 (s, 9 H, -SiC(C**H_3**)$_3$), 0.11 (s, 6 H, -SiC**H_3**).

^{13}C-NMR (75 MHz, $CDCl_3$, 298 K) *δ* / ppm = 140.8 (1 C, **C_{Ar}**), 137.8 (1 C, **C_{Ar}**), 133.7 (1 C, **C_{Ar}**),, 130.5 (1 C, **C_{Ar}**),, 128.4 (1 C, **C_{Ar}**),, 128.0 (1 H, **C_{Ar}**),, 63.9 (1 C, -**C**H_2OTBDMS), 63.1 (1 C, -**C**H_2OH), 26.0 (3 C, -SiC(**C**H_3)$_3$), 18.4 (1 C, -Si**C**(CH_3)$_3$), -5.2 (2 C, -Si**C**H_3).

HRMS (ESI-TOF): *m/z:* $[M+Na]^+$ calcd for $C_{14}H_{23}ClNaO_2Si^+$: 309.1048, found 309.1046.

(2-(((*t*-Butyldimethylsilyl)oxy)methyl)-5-chlorophenyl)methanol (4ba$^{\text{prot. diol}}$)

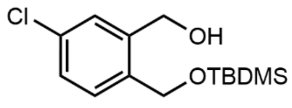

4ba$^{\text{prot. diol}}$

^{1}H-NMR (300 MHz, $CDCl_3$, 298 K) *δ* / ppm = 7.38 (s, 1 H, Ar-**H**), 7.26-7.25 (m, 2 H, Ar-**H**), 4.74 (s, 2 H, -C**H_2**OTBDMS), 4.64 (d, *J* = 5.3 Hz, 2 H, -C**H_2**OH), 3.01 (br. t, *J* = 6.0 Hz, 1 H, -O**H**), 0.91 (s, 9 H, -SiC(C**H_3**)$_3$), 0.12 (s, 6 H, -SiC**H_3**).

^{13}C-NMR (75 MHz, $CDCl_3$, 298 K) *δ* / ppm = 141.5 (1 C, **C_{Ar}**), 137.2 (1 C, **C_{Ar}**), 133.9 (1 C, **C_{Ar}**), 130.0 (1 C, **C_{Ar}**), 129.2(1 C, **C_{Ar}**), 127.9 (1 C, **C_{Ar}**), 64.0 (1 C, -**C**H_2OTBDMS), 63.4 (1 C, -**C**H_2OH), 26.0 (3 C, -SiC(**C**H_3)$_3$), 18.4 (1 C, -Si**C**(CH_3)$_3$), -5.1 (2 C, -Si**C**H_3).

HRMS (ESI-TOF): *m/z:* $[M+Na]^+$ calcd for $C_{14}H_{23}ClNaO_2Si^+$: 309.1048, found 309.1057.

(3-(((*t*-Butyldimethylsilyl)oxy)methyl)naphthalen-2-yl)methanol (4ca$^{\text{prot. diol}}$)

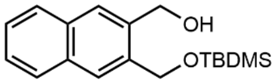

4ca$^{\text{prot. diol}}$

Monoprotected diol **4ca$^{\text{prot. diol}}$** was synthesized from the corresponding diol according to general procedure **C** and obtained in a yield of 89% as a colourless oil.

^{1}H-NMR (300 MHz, $CDCl_3$, 298 K) *δ* / ppm = 7.84-7.76 (m, 4 H, Ar-**H**), 7.49-7.46 (m, 2 H, Ar-**H**), 4.96 (s, 2 H, -C**H_2**OTBDMS), 4.84 (s, 2 H, -C**H_2**OH), 3.39 (br. s, 1 H, -O**H**), 0.93 (s, 9 H, -SiC(C**H_3**)$_3$), 0.16 (s, 6 H, -SiC**H_3**).

^{13}C-NMR (75 MHz, $CDCl_3$, 298 K) *δ* / ppm = 137.77 (1 C, **C_{Ar}**), 136.7 (1 C, **C_{Ar}**), 133.3 (1 C, **C_{Ar}**), 133.0 (1 C, **C_{Ar}**), 128.5 (1 C, **C_{Ar}**), 127.9 (1 C, **C_{Ar}**), 127.8 (1 C, **C_{Ar}**), 127.7 (1 C, **C_{Ar}**), 126.4 (1 C, **C_{Ar}**), 126.4 (1 C, **C_{Ar}**), 65.3 (1 C, -**C**H_2OTBDMS), 64.4 (1 C, -**C**H_2OH), 26.0 (3 C, -SiC(**C**H_3)$_3$), 18.5 (1 C, -Si**C**(CH_3)$_3$), -5.0 (2 C, -Si**C**H_3).

HRMS (ESI-TOF): *m/z:* $[M+Na]^+$ calcd for $C_{18}H_{26}NaO_2Si$: 325.1594, found 325.1590.

4.4 Aldehydes

2-(((tert-butyldimethylsilyl)oxy)methyl)benzaldehyde (4a^{Aldehyde})

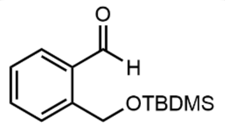

4a^{Aldehyde}

Aldehyde **4a^{Aldehyde}** was synthesized according to general procedure **D** and obtained in a yield of 90% as a yellow oil. Spectral data was in accordance with literature [5].

^{1}H-NMR (300 MHz, $CDCl_3$, 298 K) *δ* / ppm = 10.12 (1 H, -C**H**O), 7.82 (dd, J_1 = 1.2 Hz, J_2 = 7.6 Hz, 1 H, Ar-**H**), 7.78 (d, *J* = 8.0 Hz, 1 H, Ar-**H**), 7.62 (td, J_1 = 1.2 Hz, J_2 = 7.6 Hz, 1 H, Ar-**H**), 7.45 (t, *J* = 7.4 Hz, 1 H, Ar-**H**), 5.15 (s, 2 H, -C**H$_2$**), 0.96 (s, 9 H, -SiC(C**H$_3$**)$_3$), 0.13 (s, 6 H, -SiC**H$_3$**).

2-((*t*-Butyldimethylsilyl)oxy)methyl)-5-fluorobenzaldehyde (4aa^{Aldehyde})

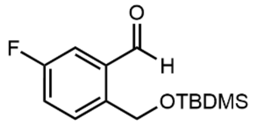

4aa^{Aldehyde}

Aldehyde **4aa^{Aldehyde}** was synthesized according to general procedure **D** and obtained in a yield of 95% as an orange oil.

^{1}H-NMR (300 MHz, $CDCl_3$, 298 K) *δ* / ppm = 10.17 (s, 1 H, -C**H**O), 7.70 (dd, J_1 = 5.3 Hz, J_2 = 8.2 Hz, 1 H, Ar-**H**), 7.52 (dd, J_1 = 2.1 Hz, J_2 = 8.5 Hz, 1 H, Ar-**H**), 7.30 (dt, J_1 = 2.0 Hz, J_2 = 8.2 Hz, 1 H, Ar-**H**), 5.08 (s, 2 H, -C**H$_2$**), 0.94 (s, 9 H, -SiC(C**H$_3$**)$_3$), 0.12 (s, 6 H, -SiC**H$_3$**).

^{13}C-NMR (75 MHz, $CDCl_3$, 298 K) *δ* / ppm = 191.7 (d, *J* = 2.0 Hz, 1 C, -**C**HO), 162.0 (d, *J* = 257.5 Hz, 1 C, **C$_{Ar}$**-F), 140.0 (d, *J* = 3.3 Hz, 1 C, **C$_{Ar}$**), 134.4 (d, *J* = 5.6 Hz, 1 C, **C$_{Ar}$**), 129.3 (d, *J* = 7.2 Hz, 1 C, **C$_{Ar}$**), 120.8 (d, *J* = 21.0 Hz, 1 C, **C$_{Ar}$**), 118.5 (d, *J* = 22.0 Hz, 1 C, **C$_{Ar}$**), 62.6 (1 C, -**C**H_2), 26.0 (3 C, -SiC(**C**H_3)$_3$), 18.4 (1 C, -Si**C**(CH_3)$_3$), -5.2 (2 C, -Si**C**H_3).

^{19}F-NMR (282 MHz, CDCl3, 298 K) *δ* / ppm = -115.1 (1 F, Ar-**F**).

HRMS (ESI-TOF): *m/z:* $[M+H]^+$ calcd for $C_{14}H_{22}FO_2Si$: 269.1368, found 269.1370.

2-((*t*-Butyldimethylsilyl)oxy)methyl)-4-fluorobenzaldehyde (4ab^{Aldehyde})

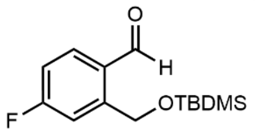

4ab^{Aldehyde}

Aldehyde **4ab^{Aldehyde}** was synthesized according to general procedure **D** and obtained in a yield of 82% as a slightly yellow oil.

^{1}H-NMR (300 MHz, $CDCl_3$, 298 K) *δ* / ppm = 10.05 (s, 1 H, -C**H**O), 7.81 (dd, J_1 = 5.7 Hz, J_2 = 8.4 Hz, 1 H, Ar-**H**), 7.53 (dd, J_1 = 2.1 Hz, J_2 = 10.4 Hz, 1 H, Ar-**H**), 7.09 (dt, J_1 = 2.5 Hz, J_2 = 8.1 Hz, 1 H, Ar-**H**), 5.14 (s, 2 H, -C**H$_2$**), 0.96 (s, 9 H, -SiC(C**H$_3$**)$_3$), 0.13 (s, 6 H, -SiC**H$_3$**).

^{13}C-NMR (75 MHz, $CDCl_3$, 298 K) *δ* / ppm = 191.8 (1 C, -**C**HO), 166.6 (d, *J* = 256.3 Hz, 1 C, **C$_{Ar}$**-F), 148.7 (d, *J* = 9.0 Hz, 1 C, **C$_{Ar}$**), 136.9 (d, *J* = 10.0 Hz, 1 C, **C$_{Ar}$**), 129.1 (d, *J* = 2.7 Hz, 1 C, **C$_{Ar}$**), 114.2 (d,

J = 19.4 Hz, 1 C, $\mathbf{C_{Ar}}$), 113.9 (d, *J* = 17.7 Hz, 1 C, $\mathbf{C_{Ar}}$), 62.6 (1 C, -**C**H_2), 26.1 (3 C, -SiC(**C**$H_3)_3$), 18.5 (1 C, -Si**C**($CH_3)_3$), -5.2 (2 C, -Si**C**H_3).

^{19}F-NMR (282 MHz, CDCl3, 298 K) *δ* / ppm = -115.2 (1 F, Ar-**F**).

HRMS (ESI-TOF): *m/z:* $[M+H]^+$ calcd for $C_{14}H_{22}FO_2Si$: 269.1368, found 269.1373.

2-(((*t*-Butyldimethylsilyl)oxy)methyl)-5-chlorobenzaldehyde (4ba^{Aldehyde})

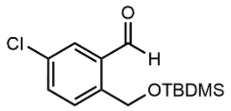

4ba^{Aldehyde}

Aldehyde **4ba^{Aldehyd}** was synthesized according to general procedure **D** and obtained in a yield of 88% as a yellow oil.

^{1}H-NMR (300 MHz, $CDCl_3$, 298 K) *δ* / ppm = 10.12 (s, 1 H, -C**H**O), 7.79 (d, *J* = 2.2 Hz, 1 H, Ar-**H**), 7.71 (d, *J* = 8.3 Hz, 1 H, Ar-**H**), 7.57 (dd, J_1 = 2.2 Hz, J_2 = 8.3 Hz, 1 H, Ar-**H**), 5.09 (s, 2 H, -C$\mathbf{H_2}$), 0.95 (s, 9 H, -SiC(C$\mathbf{H_3}$)$_3$), 0.12 (s, 6 H, -SiC$\mathbf{H_3}$).

^{13}C-NMR (75 MHz, $CDCl_3$, 298 K) *δ* / ppm = 191.9 (1 C, -**C**HO), 142.7 (1 C, $\mathbf{C_{Ar}}$), 134.0 (1 C, $\mathbf{C_{Ar}}$), 133.9 (1 C, $\mathbf{C_{Ar}}$), 133.3 (1 C, $\mathbf{C_{Ar}}$), 132.6 (1 C, $\mathbf{C_{Ar}}$), 128.7 (1 C, $\mathbf{C_{Ar}}$), 62.6 (1 C, -**C**H_2), 26.0 (3 C, -SiC(**C**$H_3)_3$), 18.5 (1 C, -Si**C**($CH_3)_3$), -5.2 (2 C, -Si**C**H_3).

HRMS (ESI-TOF): *m/z:* $[M+H]^+$ calcd for $C_{14}H_{22}ClO_2Si$: 285.1072, found 285.1074.

2-((*t*-Butyldimethylsilyl)oxy)methyl)-4-chlorobenzaldehyde (4bb^{Aldehyde})

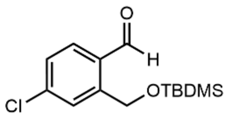

4bb^{Aldehyde}

Aldehyde **4bb^{Aldehyde}** was synthesized according to general procedure **D** and obtained in a yield of 81% as a yellow oil.

^{1}H-NMR (300 MHz, $CDCl_3$, 298 K) *δ* / ppm = 10.09 (1 H, -C**H**O), 7.78 (d, *J* = 2.0 Hz, 1 H, Ar-**H**), 7.74 (d, *J* = 8.1 Hz, 1 H, Ar-**H**), 7.41 (dd, J_1 = 2.0 Hz, J_2 = 8.1 Hz, 1 H, Ar-**H**), 5.11 (s, 2 H, -C$\mathbf{H_2}$), 0.96 (s, 9 H, -SiC(C$\mathbf{H_3}$)$_3$), 0.14 (s, 6 H, -SiC$\mathbf{H_3}$).

^{13}C-NMR (75 MHz, $CDCl_3$, 298 K) *δ* / ppm = 192.1 (1 C, -**C**HO), 146.4 (1 C, $\mathbf{C_{Ar}}$), 140.9 (1 C, $\mathbf{C_{Ar}}$), 135.0 (1 C, $\mathbf{C_{Ar}}$), 131.0 (1 C, $\mathbf{C_{Ar}}$), 127.3 (1 C, $\mathbf{C_{Ar}}$), 127.2 (1 C, $\mathbf{C_{Ar}}$), 62.6 (1 C, -**C**H_2), 26.1 (3 C, -SiC(**C**$H_3)_3$), 18.5 (1 C, -Si**C**($CH_3)_3$), -5.2 (2 C, -Si**C**H_3).

HRMS (ESI-TOF): *m/z:* $[M+H]^+$ calcd for $C_{14}H_{22}ClO_2Si$: 285.1072, found 285.1075.

3-(((tert-butyldimethylsilyl)oxy)methyl)-2-naphthaldehyde (4ca^{Aldehyde})

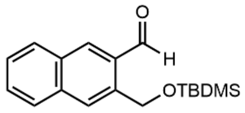

4ca^{Aldehyde}

Aldehyde **4ca^{Aldehyde}** was synthesized according to general procedure **D** and obtained in a yield of 70% as a slightly yellow oil.

^{1}H-NMR (300 MHz, $CDCl_3$, 298 K) *δ* / ppm = 10.26 (s, 1 H, -C**H**O), 8.33 (s, 1 H, Ar-**H**), 8.15 (s, 1 H, Ar-**H**), 8.00 (d, *J* = 8.1 Hz, 1 H, Ar-**H**), 7.91 (d, *J* = 8.1 Hz, 1H, Ar-**H**), 7.64 (t, *J* = 7.0 Hz, 1 H, Ar-**H**), 7.55 (t, *J* = 8.1 Hz, 1 H, Ar-**H**), 5.27 (s, 1 H, -C$\mathbf{H_2}$OTBDMS), 1.00 (s, 9 H, -SiC(C$\mathbf{H_3}$)$_3$), 0.17 (s, 6 H, -SiC$\mathbf{H_3}$).

^{13}C-NMR (75 MHz, $CDCl_3$, 298 K) *δ* / ppm = 193.5 (1 C, -**C**HO), 139.1 (1 C, $\mathbf{C_{Ar}}$), 137.5 (1 C, $\mathbf{C_{Ar}}$), 136.0 (1 C, $\mathbf{C_{Ar}}$), 131.8 (1 C, $\mathbf{C_{Ar}}$), 131.6 (1 C, $\mathbf{C_{Ar}}$), 129.5 (1 C, $\mathbf{C_{Ar}}$), 129.2 (1 C, $\mathbf{C_{Ar}}$), 128.1 (1 C, $\mathbf{C_{Ar}}$), 126.7 (1 C, $\mathbf{C_{Ar}}$), 125.8 (1 C, $\mathbf{C_{Ar}}$), 63.3 (1 C, -**C**H_2), 26.2 (3 C, -SiC(**C**H_3)$_3$), 18.6 (1 C, -Si**C**(CH_3)$_3$), -5.1 (2 C, -Si**C**H_3).

HRMS (ESI-TOF): *m/z:* $[M+H]^+$ calcd for $C_{18}H_{25}O_2Si$: 301.1618, found 301.1615.

4.5 Quinone Methides

Unsubstituted Quinone Methide (4a)

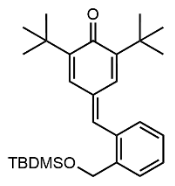

4a

Quinone methide **4a** was synthesized according to general procedure **E** and obtained as a yellow solid in a yield of 75% after column chromatography on silica (using a gradient from heptanes over heptanes/EtOAc 100/1 to 80/1 and 50/1).

^{1}H-NMR (300 MHz, $CDCl_3$, 298 K) *δ* / ppm = 7.55-7.52 (m, 1 H, Ar-**H**), 7.43-7.28 (m, 5 H, Ar-**H**, -C**H**), 7.04 (d, *J* = 2.3 Hz, 1 H, -C**H**), 4.77 (s, 2 H, -C**H**$_2$), 1.35 (s, 9 H, -CC**H**$_3$), 1.26 (s, 9 H, -CC**H**$_3$), 0.92 (s, 9 H, -SiC(C**H**$_3$)$_3$), 0.10 (s, 6 H, -SiC**H**$_3$).

^{13}C-NMR (75 MHz, $CDCl_3$, 298 K) *δ* / ppm = 186.7 (1 C, -**C**=O), 149.3 (1 C, -**C**C(CH_3)$_3$), 147.9 (1 C, -**C**C(CH_3)$_3$), 140.8 (1 C, **C**$_{Ar}$), 140.6 (1 C, **C**$_{Ar}$), 135.0 (1 C, -**C**H), 133.7 (1 C, -**C**H), 132.4 (1 C, -**C**=CH), 130.9 (1 C, **C**$_{Ar}$), 129.2 (1 C, **C**$_{Ar}$), 128.4 (1 C, **C**$_{Ar}$), 127.4 (1 C, **C**$_{Ar}$), 127.1 (1 C, -C=**C**H), 63.6 (1 C, -**C**H_2), 35.5 (1 C, 1 C, -**C**(CH_3)$_3$), 35.1 (1 C, -**C**(CH_3)$_3$), 29.6 (6 C, -**C**H_3), 26.0 (3 C, -SiC(**C**H_3)$_3$), 18.4 (1 C, -Si**C**(CH_3)$_3$), -5.2 (2 C, -Si**C**H_3).

HRMS (ESI-TOF): *m/z:* $[M+H]^+$ calcd for $C_{28}H_{43}O_2Si^+$: 439.3027, found 439.3030.

Melting point: 85.0-88.0 °C

5-Fluoro Quinone Methide (4aa)

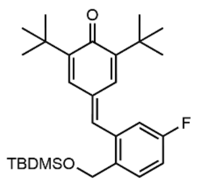

4aa

Quinone methide **4aa** was synthesized according to general procedure **E** and obtained as a yellow solid in a yield of 46% after column chromatography on silica (using a gradient from heptanes over heptanes/EtOAc 100/1 to 80/1 and 50/1).

^{1}H-NMR (300 MHz, $CDCl_3$, 298 K) *δ* / ppm = 7.48 (dd, J_1 = 5.8 Hz, J_2 = 8.4 Hz, 1 H, Ar-**H**), 7.26-7.23 (m, 2 H, Ar-**H**, -C**H**), 7.11-7.00 (3 H, Ar-**H**, -C**H**), 4.70 (s, 2 H, -C**H**$_2$), 1.34 (s, 9 H, -C**H**$_3$), 1.26 (s, 9 H, -C**H**$_3$), 0.91 (s, 9 H, -SiC(C**H**$_3$)$_3$), 0.09 (6 H, -SiC**H**$_3$).

^{13}C-NMR (75 MHz, $CDCl_3$, 298 K) *δ* / ppm = 186.7 (1 C, -**C**=O), 161.7 (d, *J* = 246.1 Hz, 1 C, **C**$_{Ar}$-F), 149.9 (1 C, -**C**C(CH_3)$_3$), 148.4 (1 C, -**C**C(CH_3)$_3$), 138.8 (1 C, -**C**=CH), 136.4 (d, *J* = 3.0 Hz, 1 C, **C**$_{Ar}$), 135.5 (d, *J* = 7.9 Hz, 1 C, **C**$_{Ar}$), 134.7 (1 C, -**C**H), 133.1 (1 C, -**C**H), 129.3 (d, *J* = 8.3 Hz, 1 C, **C**$_{Ar}$), 127.7 (1 C, -**C**H), 117.5 (d, *J* = 22.6 Hz, 1 C, **C**$_{Ar}$), 115.6 (d, *J* = 20.9 Hz, 1 C, **C**$_{Ar}$), 63.1 (1 C, -**C**H_2), 35.6 (1 C, -**C**(CH_3)$_3$), 35.2 (1 C, -**C**(CH_3)$_3$), 29.6 (6 C, -C(**C**H_3)$_3$), 26.0 (3 C, -SiC(**C**H_3)$_3$), 18.4 (1 C, -Si**C**(CH_3)$_3$), -5.1 (2 C, -Si**C**H_3).

^{19}F-NMR (282 MHz, CDCl3, 298 K) *δ* / ppm = -115.52 (1 F, Ar-**F**).

HRMS (ESI-TOF): *m/z:* $[M+H]^+$ calcd for $C_{28}H_{42}O_2FSi^+$: 457.2933, found 457.2936.

Melting point: 99.0-101.9 °C

4-Fluoro Quinone Methide (4ab)

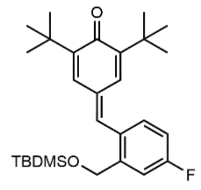

4ab

Quinone methide **4ab** was synthesized according to general procedure **E** and obtained as a yellow solid in a yield of 68% after column chromatography on silica (using a gradient from heptanes over heptanes/EtOAc 100/1 to 80/1 and 50/1).

^{1}H-NMR (300 MHz, $CDCl_3$, 298 K) δ / ppm = 7.33-7.23 (m, 3 H, Ar-**H**, -C**H**), 7.14 (s, 1 H, -C**H**), 7-06-6.99 (m, 2 H, Ar-**H**, -C**H**), 4.73 (s, 2 H, -C**H$_2$**), 1.34 (s, 9 H, -C**H$_3$**), 1.26 (s, 9 H, -C**H$_3$**), 0.94 (s, 9 H, -SiC(C**H$_3$**)$_3$), 0.12 (s, 6 H, -SiC**H$_3$**).

^{13}C-NMR (75 MHz, $CDCl_3$, 298 K) δ / ppm = 186.7 (1 C, -**C**=O), 163.6 (d, *J* = 249.7 Hz, 1 C, -**C$_{Ar}$**-F), 149.6 (1 C, -**C**C(CH_3)$_3$), 148.1 (1 C, -**C**C(CH_3)$_3$), 143.7 (d, *J* = 7.5 Hz, 1 C, **C$_{Ar}$**), 138.8 (1 C, -**C**=CH), 134.7 (1 C, -**C**H), 132.7 (1 C, -**C**H), 132.6 (d, *J* = 8.2 Hz, 1 C, **C$_{Ar}$**), 129.0 (d, *J* = 3.3 Hz, 1 C, **C$_{Ar}$**), 128.0 (1 C, -**C**H), 114.2 (d, *J* = 23.0 Hz, 1 C, **C$_{Ar}$**), 113.8 (d, *J* = 21.6 Hz, 1 C, **C$_{Ar}$**), 62.8 (1 C, -**C**H$_2$), 35.5 (1 C, -**C**(CH_3)$_3$), 35.2 (1 C, -**C**(CH_3)$_3$), 29.6 (6 C, -C(**C**H$_3$)$_3$), 26.0 (3 C, -SiC(**C**H$_3$)$_3$), 18.5 (1 C, -Si**C**(CH_3)$_3$), -5.2 (2 C, -Si**C**H$_3$).

^{19}F-NMR (282 MHz, CDCl3, 298 K) δ / ppm = -111.0 (1 F, Ar-**F**).

HRMS (ESI-TOF): *m/z:* $[M+H]^+$ calcd for $C_{28}H_{42}O_2FSi^+$: 457.2933, found 457.2935.

Melting point: 98.5-100.0 °C

5-Chloro Quinone Methide (4ba)

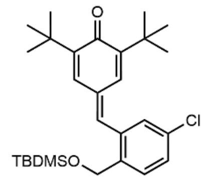

4ba

Quinone methide **4ba** was synthesized according to general procedure **E** and obtained as a yellow solid in a yield of 67% after column chromatography on silica (using a gradient from heptanes over heptanes/EtOAc 100/1 to 80/1 and 50/1) and recrystallization from *n*-hexane.

^{1}H-NMR (300 MHz, $CDCl_3$, 298 K) δ / ppm = 7.47 (d, *J* = 8.2 Hz, 1 H, Ar-**H**), 7.36 (dd, J_1 = 2.1 H, J_2 = 8.2 Hz, 1 H, Ar-**H**), 7.29 (d, *J* = 1.9 Hz, 1 H, Ar-**H**), 7.24 (d, *J* = 2.2 Hz, 1 H, -**C**H), 7.19 (s, 1 H, -C**H**), 7.01 (d, *J* = 2.2 Hz, 1 H, -C**H**), 4.70 (s, 2 H, -C**H$_2$**), 1.34 (s, 9 H, -C**H$_3$**), 1.27 (s, 9 H, -C**H$_3$**), 0.92 (s, 9 H, -SiC(C**H$_3$**)$_3$), 0.10 (s, 6 H, -SiC**H$_3$**)$_3$).

^{13}C-NMR (75 MHz, $CDCl_3$, 298 K) δ / ppm = 186.7 (1 C, -**C**=O), 149.7 (1 C, -**C**C(CH_3)$_3$), 148.3 (1 C, -**C**C(CH_3)$_3$), 142.5 (1 C, **C$_{Ar}$**), 138.7 (1 C, -**C**H), 135.4 (1 C, -**C**=CH), 134.6 (1 C, -**C**H), 132.9 (1 C, **C$_{Ar}$**), 132.0 (1 C, **C$_{Ar}$**), 131.7 (1 C, **C$_{Ar}$**), 127.9 (1 C, **C$_{Ar}$**), 127.4 (1 C, **C$_{Ar}$**), 1227.1 (1 C, -**C**H), 62.9 (1 C, -**C**H$_2$), 35.6 (1 C, -**C**(CH_3)$_3$), 35.2 (1 C, -**C**(CH_3)$_3$), 29.6 (6 C, -C(**C**H$_3$)$_3$), 26.0 (3 C, -SiC(**C**H$_3$)$_3$), 18.5 (1 C, -Si**C**(CH_3)$_3$), -5.2 (1 C, -Si**C**H$_3$).

HRMS (ESI-TOF): *m/z:* $[M+H]^+$ calcd for $C_{28}H_{42}O_2ClSi^+$: 473.2637, found 473.2640.

Melting point: 132.2-135.6 °C

4-Chloro Quinone Methide (4bb)

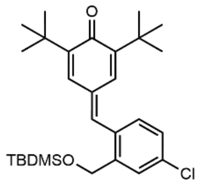

4bb

Quinone methide **4bb** was synthesized according to general procedure **E** and obtained as a yellow solid in a yield of 71% after column chromatography on silica (using a gradient from heptanes over heptanes/EtOAc 100/1 to 80/1 and 50/1) and recrystallization from *n*-hexane.

^{1}H-NMR (300 MHz, $CDCl_3$, 298 K) δ / ppm = 7.55 (d, J = 2.2 Hz, 1 H, -C**H**), 7.32 (dd, J_1 = 2.0 Hz, J_2 = 8.1 Hz, 1 H, Ar-**H**), 7.22-7.20 (m, 2 H, Ar-**H**), 7.16 (s, 1 H, -C**H**), 7.02 (d, J = 2.2 Hz, 1 H, -C**H**), 4.72 (s, 2 H, -**C**H_2), 1.34 (s, 9 H, -C$\mathbf{H_3}$), 1.25 (s, 9 H, -**C**H_3), 0.94 (s, 9 H, -SiC(C$\mathbf{H_3}$)$_3$), 0.12 (s, 6 H, -SiC$\mathbf{H_3}$).

^{13}C-NMR (75 MHz, $CDCl_3$, 298 K) δ / ppm = 186.7 (1 C, -**C**=O), 149.7 (1 C, -**C**C(CH_3)$_3$), 148.3 (1 C, -**C**C(CH_3)$_3$), 142.5 (1 C, $\mathbf{C_{Ar}}$), 138.7 (1 C, -**C**H), 135.4 (1 C, -**C**=CH), 134.6 (1 C, -**C**H), 132.9 (1 C, $\mathbf{C_{Ar}}$), 132.0 (1 C, $\mathbf{C_{Ar}}$), 131.7 (1 C, $\mathbf{C_{Ar}}$), 127.9 (1 C, $\mathbf{C_{Ar}}$), 127.4 (1 C, $\mathbf{C_{Ar}}$), 127.1 (1 C, -**C**H), 62.9 (1 C, -**C**H_2), 35.6 (1 C, -**C**(CH_3)$_3$), 35.2 (1 C, -**C**(CH_3)$_3$), 29.6 (6 C, -C(**C**H_3)$_3$), 26.0 (3 C, -SiC(**C**H_3)$_3$), 18.5 (1 C, -Si**C**(CH_3)$_3$), -5.2 (2 C, -Si**C**H_3).

HRMS (ESI-TOF): *m/z:* $[M+H]^+$ calcd for $C_{28}H_{42}O_2ClSi^+$: 473.2637, found 473.2639.

Melting point: 129.3-130.1 °C

2-Naphthyl Quinone Methide (4ca)

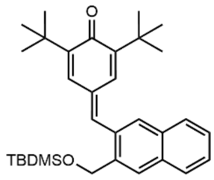

4da

Quinone methide **4ca** was synthesized according to general procedure **E** and obtained as a yellow solid in a yield of 31% after column chromatography on silica (yield after two columns, using a gradient from heptanes over heptanes/EtOAc 100/1 to 80/1 and 50/1).

^{1}H-NMR (300 MHz, $CDCl_3$, 298 K) δ / ppm = 7.92 (s, 1 H, Ar-**H**), 7.89-7.84 (m, 2 H, Ar-**H**), 7.79 (s, 1 H, Ar-**H**), 7.57-7.52 (m, 2 H, Ar-**H**), 7.49 (s, 1 H, -C**H**), 7.45 (d, J = 2.3 Hz, 1 H, -C**H**), 7.11 (d, J = 2.3 Hz, 1 H, -C**H**), 4.91 (s, 2 H, -C$\mathbf{H_2}$), 1.37 (s, 9 H, -C$\mathbf{H_3}$), 1.27 (s, 9 H, -C$\mathbf{H_3}$), 0.95 (s, 9 H, -SiC(C$\mathbf{H_3}$)$_3$), 0.14 (s, 6 H, -SiC$\mathbf{H_3}$).

^{13}C-NMR (75 MHz, $CDCl_3$, 298 K) δ / ppm = 186.9 (1 C, -**C**=O), 149.5 (1 C, -**C**C(CH_3)$_3$), 148.0 (1 C, -**C**C(CH_3)$_3$), 140.9 (1 C, $\mathbf{C_{Ar}}$), 137.8 (1 C, $\mathbf{C_{Ar}}$), 135.0 (1 C, -**C**H), 133.5 (1 C, -**C**=CH), 132.8 (1 C, $\mathbf{C_{Ar}}$), 132.5 (1 C, $\mathbf{C_{Ar}}$), 132.4 (1 C, $\mathbf{C_{Ar}}$), 131.3 (1 C, -**C**H), 128.5 (1 C, $\mathbf{C_{Ar}}$), 128.2 (1 C, $\mathbf{C_{Ar}}$), 127.9 (1 C, $\mathbf{C_{Ar}}$), 127.3 (1 C, $\mathbf{C_{Ar}}$), 126.6 (1 C, -C=**C**H), 126.4 (1 C, $\mathbf{C_{Ar}}$), 64.2 (1 C, -**C**H_2), 35.5 (1 C, -**C**(CH_3)$_3$), 35.2 (1 C, -**C**(CH_3)$_3$), 29.7 (3 C, -C(**C**H_3)$_3$), 29.7 (3 C, -C(**C**H_3)$_3$), 26.0 (3 C, -SiC(**C**H_3)$_3$), 18.5 (1 C, -Si**C**(CH_3)$_3$), -5.1 (2 C, -Si**C**H_3).

HRMS (ESI-TOF): *m/z:* $[M+H]^+$ calcd for $C_{32}H_{45}O_2Si^+$: 489.3183, found 489.3182.

Melting point: 123.8-125.6 °C

Unsymmetrically Substituted Quinone Methide (4da)

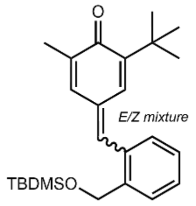

4da

Quinone methide **4da** was synthesized according to general procedure **E** and obtained as a yellow oil in a yield of 12% (roughly 50:50 mixture of *E/Z* isomers) after column chromatography on silica (yield after two columns, using a gradient from heptanes over heptanes/EtOAc 100/1 to 80/1 and 50/1).

^{1}H-NMR (300 MHz, $CDCl_3$, 298 K) δ / ppm = 7.55-7.51 (m, 2 x 1 H, Ar-**H**), 7.44-7.26 (m, 2 x 5 H, Ar-**H**+-C**H**), 7.10-7.06 (m, 2 x 1 H, -C**H**), 4.74 (s, 2 x 2 H, -C**H₂**), 2.06 (d, *J* = 1.0 Hz, 3 H, -C**H₃**), 2.00 (d, *J* = 1.0 Hz, 3 H, -C**H₃**), 1.35 (s, 9 H, -C(C**H₃**)₃), 1.26 (s, 9 H, -C(C**H₃**)₃), 0.92 (s, 9 H, -SiC(C**H₃**)₃), 0.92 (s, 9 H, -SiC(C**H₃**)₃), 0.10 (s, 3 H, -SiC**H₃**), 0.09 (s, 3 H, -SiC**H₃**).

^{13}C-NMR (75 MHz, $CDCl_3$, 298 K) δ / ppm = 187.1 (1 C, -**C**OOR), 187.0 (1 C, -**C**OOR), *Signals for aromatic and alkene carbons (2 x 12 C, **C**$_{Ar}$, -**C**H, -**C**=CH):* 147.9, 146.5, 141.2, 140.7, 140.7, 140.6, 139.1, 137.5, 137.1, 136.6, 133.6, 133.5, 132.4, 131.0, 131.0, 130.7, 129.8, 129.4, 129.3, 127.5, 127.4, 127.2, 127.1, 126.6, 63.6 (1 C, -**C**H₂), 63.5 (1 C, -**C**H₂), 35.3 (1 C, -**C**(CH₃)₃), 35.0 (1 C, -**C**(CH₃)₃), 29.4 (2 x 3 C, -C(**C**H₃)₃), 36.0 (2 x 3 C, -SiC(**C**H₃), 18.5 (1 C, -Si**C**(CH₃)₃), 18.5 (1 C, -Si**C**(CH₃)₃), 17.1 (1 C, -**C**H₃), 16.6 (1 C, -**C**H₃), -5.1 (2 x 1 C, -Si**C**H₃), -5.2 (2 x 1 C, -Si**C**H₃).

HRMS (ESI-TOF): *m/z:* $[M+H]^+$ calcd for $C_{25}H_{37}O_2Si^+$: 397.2557, found 397.2559.

5. Synthesis of 7-Membered Lactones

5.1 General Information

In general, the synthesis of 7-membered lactones involved three separate steps: Alkylation, deprotection and cyclization. After the alkylation step, products were roughly purified by a quick column chromatographic step (deactivated silica, heptanes/EtOAc gradient) or preparative TLC (heptanes/EtOAc 5/1) in case of poor conversion (<50%). This was mainly done to remove the base, catalyst, and free phenol, giving products **5** in significant purity for the deprotection/cyclization step. The deprotection and cyclization were done in the same flask with a change of solvent in between (no MeOH should be present during the cyclization step anymore due to partial formation of the methyl ester instead of the cyclic product). The products were obtained as a mixture of diastereomers in any case. Diastereomeric ratio and conversion were determined by looking at crude ^{1}H- and ^{19}F-NMR spectra. NMR and HRMS data are given for both the alkylation step and the final cyclic products. Enantiomeric ratios were determined by HPLC analysis of the final cyclic products. HPLC analysis and measurements of optical rotation were done with the mixture of diastereomers unless otherwise stated. In most cases, the diastereomers were separable by semi preparative HPLC. In those cases, NMR data for both diastereomers of the final 7-membered rings are given separately, for the other cases NMR data is reported for the mixture.

Racemic samples were obtained by performing the reaction using Okamoto's catalyst (DHPB). This usually led to a better *dr* compared to TM · HCl. This is in accordance with observations made during the optimization process (also see main manuscript). Also, when using DHPB, the *cis* diastereomer was the major product, while TM · HCl gave the *trans* as major product.

5.2 General Procedure F for the Synthesis of 7-Membered Lactones

Adapting literature procedures [15,16], the respective ester **3** (0.1 mmol, 1 eq) and TM · HCl (**ITU5**, 4.8 mg, 20 mol%) were dissolved in a Schlenk flask under Ar in MeCN (1 mL, 0.1 mol L^{-1}) at r.t. Then, tetramethylpiperidine (TMP, 17 µL, 0.1 mmol, 1 eq) and the respective quinone methide **4** (0.1 mmol, 1 eq) were added. The mixture was stirred at r.t. for 64 h after which the solvents were evaporated. The crude was submitted to a quick column (deactivated silica, heptanes followed by heptanes/EtOAc 50/1; 10/1 and 5/1) or preparative TLC (heptanes/EtOAc 5/1, only if conversion was low). The products **5** from the chromatography step were dissolved in 2 mL MeOH in a round bottom flask and $(n\text{-Bu})_4NBr_3$ (4.8 mg, 20mol% with respect to the initial 0.1 mmol scale) was added. The mixture was stirred for 16 h after which MeOH was evaporated. The residues were taken up in 2 mL DCM and DMAP (2.4 mg, 20mol% with respect to the initial 0.1 mmol scale) was added. The mixture was stirred for 24 h at r.t. after which it was filtered over a deactivated silica plug. The reaction flask and silica were washed with 15 mL DCM trice. The filtrate was concentrated to give the cyclic products in sufficient purity.

5.3 Characterization of 7-Membered Lactones

Unsubstituted benzo[c]oxepinone derivative (6a)

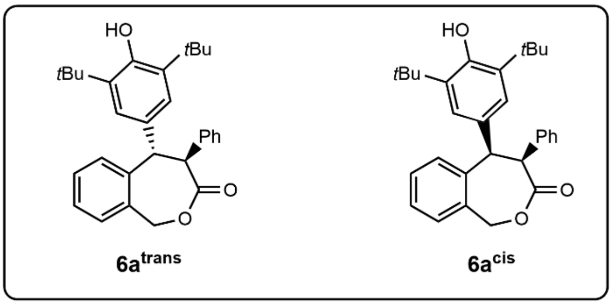

7-membered ring **6a** was synthesized according to general procedure **F** and obtained as a colorless residue in a yield of 51% (57% for the alkylation step) as a mixture of diastereomers (*dr* 50:50; er_{trans} 97:3; er_{cis} 93:7).

Data for the alkylation product (mixture of diastereomers) 5a:

*As the diastereomers were not separated on this stage, the NMR data is reported for the mixture. Often, there is a lot of overlap between the signals and a clear assignment is not possible for both diastereomers. Therefore, mainly the signals for the major diastereomer (unlike in case of Okamoto's catalyst, like in case of TM HCl [**ul-5** give **cis-6** products; **l-5** give **trans-6** in the cyclization) and selected signals for the minor diastereomer (vice versa) are given. Also, as those are not the final products of interest, spectra are not always perfectly clean, and the interpretation thereof is a bit rougher. All of the above also applies to all other alkylation products (vide infra).*

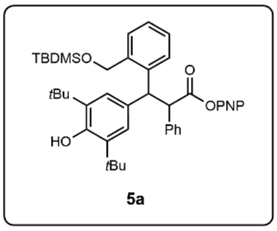

^{1}H-NMR (300 MHz, $CDCl_3$, 298 K) δ / ppm = *Signals for the unlike diastereomer:* 8.10 (d, *J* = 9.1 Hz, 2 H, Ar-**H**), 7.49-7.10 (m, 11 H, Ar-**H**), 6.61 (d, *J* = 9.1 Hz, 2 H, Ar-**H**), 5.16 (1 H, -O**H**), 4.98 (d, *J* = 12.1 Hz, 1 H, -C**H**$_2$), 4.78 (d, *J* = 13.1 Hz, 1 H, -C**H**), 4.75 (d, *J* = 12.1 Hz, 1 H, -C**H**$_2$), 4.62 (d, *J* = 13.1 Hz, 1 H, -C**H**), 1.40 (s, 18 H, -C**H**$_3$), 0.95 (s, 9 H, -SiC(C**H**$_3$), 0.10 (s, 3 H, -SiC**H**$_3$), 0.07 (s, 3 H, -SiC**H**$_3$). *Selected signals for the like diastereomer:* 8.12 (d, *J* = 9.1 Hz, 2 H, Ar-**H**), 6.78 (d, *J* = 9.1 Hz, 2 H, Ar-**H**), 4.92 (s, 1 H, -O**H**), 4.90 (d, *J* = 13.6 Hz, 1 H, -C**H**), 4.65 (d, *J* = 12.0 Hz, 1 H, -C**H**$_2$), 4.60 (d, *J* = 13.6 Hz, 1 H, -C**H**), 4.46 (d, *J* = 12.0 Hz, 1 H, -C**H**$_2$), 1.21 (s, 18 H, -C**H**$_3$), 0.85 (s, 9 H, -SiC(C**H**$_3$), -0.06 (s, 3 H, -SiC**H**$_3$), -0.07 (s, 3 H, -SiC**H**$_3$).

^{13}C-NMR (75 MHz, $CDCl_3$, 298 K) δ / ppm = *Signals for the unlike diastereomer:* 171.2 (1 C, -**C**OOR), 155.5 (1 C, **C**$_{Ar}$), 152.9 (1 C, **C**$_{Ar}$), 145.5 (1 C, **C**$_{Ar}$), 139.0 (1 C, **C**$_{Ar}$), 137.9 (1 C, **C**$_{Ar}$), 136.1 (2 C, **C**$_{Ar}$), 136.0 (1 C, **C**$_{Ar}$), 131.8 (1 C, **C**$_{Ar}$), 128.8 (2 C, **C**$_{Ar}$), 128.7 (2 C, **C**$_{Ar}$), 127.9 (1 C, **C**$_{Ar}$), 127.2 (1 C, **C**$_{Ar}$), 127.0 (1 C, **C**$_{Ar}$), 126.5 (1 C, **C**$_{Ar}$), 126.4 (1 C, **C**$_{Ar}$), 125.5 (2 C, **C**$_{Ar}$), 125.1 (2 C, **C**$_{Ar}$), 122.5 (2 C, **C**$_{Ar}$), 63.1 (1 C, -**C**H$_2$), 57.8 (1 C, -**C**H), 48.6 (1 C, -**C**H), 34.5 (2 C, -**C**(CH$_3$)$_3$), 30.4 (6 C, -C(**C**H$_3$)$_3$), 26.1 (3 C, -SiC(**C**H$_3$)$_3$), 18.6 (1 C, -Si**C**(CH$_3$)$_3$), -5.1 (1 C, -Si**C**H$_3$), -5.2 (1 C, -Si**C**H$_3$). *Selected signals for the like diastereomer:* 170.8 (1 C, -**C**OOR), 139.6 (1 C, **C**$_{Ar}$), 139.2 (1 C, **C**$_{Ar}$), 136.6 (1 C, **C**$_{Ar}$), 135.3 (2 C, **C**$_{Ar}$),

129.9 (1 C, **C$_{Ar}$**), 128.9 (2 C, **C$_{Ar}$**), 128.7 (2 C, **C$_{Ar}$**), 127.8 (1 C, **C$_{Ar}$**), 126.9 (1 C, **C$_{Ar}$**), 125.4 (2 C, **C$_{Ar}$**), 125.2 (2 C, **C$_{Ar}$**), 62.8 (1 C, -**C**H_2), 58.3 (1 C, -**C**H), 50.7 (1 C, -**C**H), 34.2 (2 C, -**C**$(CH_3)_3$), 30.2 (6 C, -C(**C**$H_3)_3$), 26.0 (3 C, -SiC(**C**$H_3)_3$), 18.5 (1 C, -Si**C**$(CH_3)_3$), -5.2 (1 C, -Si**C**H_3), -5.4 (1 C, -Si**C**H_3).

HRMS (ESI-TOF): *m/z:* $[M+H]^+$ calcd for $C_{42}H_{53}NNaO_6Si^+$: 718.3534, found 718.3537.

Data for the cyclic product 6a:

HPLC (YMC-SB, *n*-hexane/IPA = 10/1, flow = 1.0 mL min^{-1}, T_{Column} = 10 °C, *l* = 240 nm): t_r*(trans):* 15.4 min (major), 35.2 min (minor); t_r*(cis):* 13.7 min (major), 19.5 min (minor).

α_D^{20}(c = 1, $CHCl_3$): -59.5 (mixture of diastereomers).

HRMS (ESI-TOF): *m/z:* $[M+NH_4]^+$ calcd for $C_{30}H_{38}NO_3^+$: 460.2846, found 460.2850.

NMR data for the cis diastereomer:

^{1}H-NMR (500 MHz, $CDCl_3$, 298 K) δ / ppm = 7.27-7.22 (6 H, Ar-**H**), 7.08-7.06 (m, 1 H, Ar-**H**), 7.02-7.00 (m, 2 H, Ar-**H**), 6.56 (s, 2 H, Ar-**H**), 5.91 (d, *J* = 15.1 Hz, 1 H, -C**H$_2$**), 5.22 (d, *J* = 15.1 Hz, 1 H, -C**H$_2$**), 5.12 (s, 1 H, -O**H**), 4.95 (d, *J* = 3.4 Hz, 1 H, -C**H**), 4.32 (d, *J* = 3.4 Hz, 1 H, -C**H**), 1.31 (s, 18 H, -C**H$_3$**).

^{13}C-NMR (125 MHz, $CDCl_3$, 298 K) δ / ppm = 172.1 (1 C, -**C**OOR), 153.2 (1 C, **C$_{Ar}$**), 141.2 (1 C, **C$_{Ar}$**), 136.8 (1 C, **C$_{Ar}$**), 135.1 (2 C, **C$_{Ar}$**), 132.6 (1 C, **C$_{Ar}$**), 132.4 (1 C, **C$_{Ar}$**), 130.9 (1 C, **C$_{Ar}$**), 130.9 (2 C, **C$_{Ar}$**), 129.0 (1 C, **C$_{Ar}$**), 128.1 (1 C, **C$_{Ar}$**), 127.7 (2 C, **C$_{Ar}$**), 127.6 (1 C, **C$_{Ar}$**), 127.2 (2 C, **C$_{Ar}$**), 126.6 (1 C, **C$_{Ar}$**), 70.6 (1 C, -**C**H_2), 55.2 (1 C, -C**H**), 50.2 (1 C, -**C**H), 34.4 (2 C, -**C**$(CH_3)_3$), 30.3 (6 C, -**C**H_3).

NMR data for the trans diastereomer:

^{1}H-NMR (500 MHz, $CDCl_3$, 298 K) δ / ppm = 7.28-7.21 (m, 6 H, Ar-**H**), 7.18-7.16 (m, 2 H, Ar-**H**), 7.13-7.11 (m, 1 H, Ar-**H**), 6.46 (s, 2 H, Ar-**H**), 5.83 (d, *J* = 13.7 Hz, 1 H, -C**H$_2$**), 5.06 (d, *J* = 13.7 Hz, 1 H, -C**H$_2$**), 4.99 (s, 1 H, -O**H**), 4.56 (d, *J* = 10.2 Hz, 1 H, -C**H**), 4.49 (d, *J* = 10.2 Hz, 1 H, -C**H**), 1.22 (s, 18 H, -C**H$_3$**).

^{13}C-NMR (125 MHz, $CDCl_3$, 298 K) δ / ppm = 172.5 (1 C, -**C**OOR), 152.4 (1 C, **C$_{Ar}$**), 141.0 (1 C, **C$_{Ar}$**), 137.7 (1 C, **C$_{Ar}$**), 135.7 (2 C, **C$_{Ar}$**), 134.5 (1 C, **C$_{Ar}$**), 133.6 (1 C, **C$_{Ar}$**), 132.4 (1 C, **C$_{Ar}$**), 129.7 (1 C, **C$_{Ar}$**), 129.6 (2 C, **C$_{Ar}$**), 129.4 (1 C, **C$_{Ar}$**), 128.3 (2 C, **C$_{Ar}$**), 127.5 (1 C, **C$_{Ar}$**), 126.9 (1 C, **C$_{Ar}$**), 125.0 (2 C, **C$_{Ar}$**), 70.25 (1 C, -**C**H_2), 56.1 (1 C, -**C**H), 54.0 (1 C, -**C**H), 34.2 (2 C, -**C**$(CH_3)_3$), 30.2 (6 C, -**C**H_3).

The experiment was also repeated on a 1 mmol scale, delivering the final cyclic product in a yield of 50% (*dr* 50:50, er_{trans} 96:4, er_{cis} 93:7) as an off-white solid (melting point 77.2-79.3 °C).

2-Fluorophenyl benzo[c]oxepinone derivative (6b)

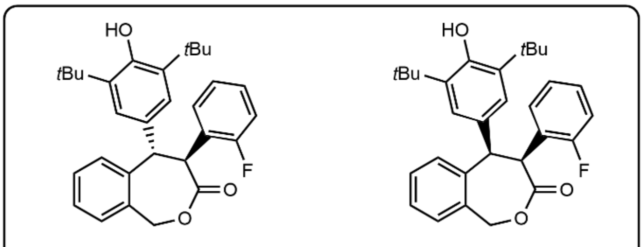

$\mathbf{6b^{trans}}$ $\mathbf{6b^{cis}}$

7-membered ring **6b** was synthesized according to general procedure **F** and obtained as a colorless residue in a yield of 58% (63% for the alkylation step) as a mixture of diastereomers (*dr* 60:40; er_{trans} 96:4; er_{cis} 90:10).

Data for the alkylation product (mixture of diastereomers) 5b:

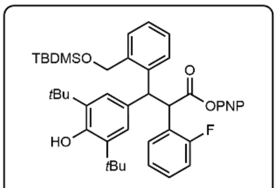

5b

^{1}H-NMR (500 MHz, $CDCl_3$, 298 K) *δ* / ppm = *Signals for the unlike diastereomer:* 8.10 (d, *J* = 9.0 Hz, 2 H, Ar-**H**), 7.71-7.30 (m, 4 H, Ar-**H**), 7.22 (s, 2 H, Ar-**H**), 7.19-6.98 (m, 4 H, Ar-**H**), 6.61 (d, *J* = 9.0 Hz, 2 H, Ar-**H**), 5.17 (d, *J* = 12.2 Hz, 1 H, -C**H₂**), 5.17 (s, 1 H, -O**H**), 5.02 (d, *J* = 12.2 Hz, 1 H, -C**H₂**), 4.94 (d, *J* = 13.0 Hz, 1 H, -C**H**), 4.59 (d, *J* = 13.0 Hz, 1 H, -C**H**), 1.39 (s, 18 H, -C**H₃**), 0.96 (s, 9 H, -SiC(C**H₃**)₃), 0.12 (s, 3 H, -SiC**H₃**), 0.08 (s, 3 H, -SiC**H₃**). *Selected signals for the like diastereomer:* 8.13 (d, *J* = 9.0 Hz, 2 H, Ar-**H**), 7.79 (d, *J* = 8.1 Hz, 1 H, Ar-**H**), 6.86-6.82 (m, 1 H, Ar-**H**), 6.80 (d, *J* = 9.0 Hz, 2 H, Ar-**H**), 6.67 (s, 2 H, Ar-**H**), 5.06 (d, *J* = 12.0 Hz, 1 H, -C**H₂**), 4.93 (s, 1 H, -O**H**), 4.91 (d, *J* = 13.6 Hz, 1 H, -C**H**), 4.65 (d, *J* = 12.0 Hz, 1 H, -C**H₂**), 4.64 (d, *J* = 13.6 Hz, 1 H, -C**H**), 1.24 (s, 18 H, -C**H₃**), 0.87 (s, 9 H, -SiC(C**H₃**)₃), -0.03 (s, 3 H, -SiC**H₃**), -0.04 (s, 3 H, -SiC**H₃**).

^{19}F-NMR (470 MHz, $CDCl_3$, 298 K) *δ* / ppm = -117.0 (1 F, Ar-**F**, *like*), -117.4 (1 F, Ar-**F**, *unlike*).

^{13}C-NMR (125 MHz, $CDCl_3$, 298 K) *δ* / ppm = *Signals for the cis diastereomer:* 170.9 (1 C, -**C**OOR), 160.8 (d, *J* = 245.9 Hz, 1 C, $\mathbf{C_{Ar}}$-F), 155.3 (1 C, $\mathbf{C_{Ar}}$), 153.0 (1 C, $\mathbf{C_{Ar}}$), 145.5 (1 C, $\mathbf{C_{Ar}}$), 140.8 (1 C, $\mathbf{C_{Ar}}$), 138.9 (1 C, $\mathbf{C_{Ar}}$), 137.8 (1 C, $\mathbf{C_{Ar}}$), 136.1 (2 C, $\mathbf{C_{Ar}}$), 131.1 (1 C, $\mathbf{C_{Ar}}$), 129.6 (d, *J* = 3.4 Hz, 1 C, $\mathbf{C_{Ar}}$), 129.4 (d, *J* = 8.3 Hz, 1 C, $\mathbf{C_{Ar}}$), 127.4 (1 C, $\mathbf{C_{Ar}}$), 127.2 (1 C, $\mathbf{C_{Ar}}$), 127.0 (d, *J* = 12.3 Hz, 1 C, $\mathbf{C_{Ar}}$), 126.7 (1 C, $\mathbf{C_{Ar}}$), 125.8 (2 C, $\mathbf{C_{Ar}}$), 125.0 (2 C, $\mathbf{C_{Ar}}$), 124.7 (d, *J* = 3.0 Hz, 1 C, $\mathbf{C_{Ar}}$), 122.5 (2 C, $\mathbf{C_{Ar}}$), 115.4 (d, *J* = 22.6 Hz, 1 C, $\mathbf{C_{Ar}}$), 63.2 (1 C, -**C**H₂), 50.0 (1 C, -**C**H), 48.3 (1 C, -**C**H), 34.5 (2 C, -**C**(CH₃)₃), 30.4 (6 C, -C(**C**H₃)₃), 26.1 (3 C, -SiC(**C**H₃)₃), 18.6 (1 C, -Si**C**(CH₃)₃), -5.1 (1 C, -Si**C**H₃), -5.2 (1 C, -Si**C**H₃). *Selected signals for the trans diastereomer:* -170.3 (1 C, -**C**OOR), 155.4 (1 C, $\mathbf{C_{Ar}}$), 152.4 (1 C, $\mathbf{C_{Ar}}$), 140.6 (1 C, $\mathbf{C_{Ar}}$), 139.6 (1 C, $\mathbf{C_{Ar}}$), 138.9 (1 C, $\mathbf{C_{Ar}}$), 135.3 (2 C, $\mathbf{C_{Ar}}$), 130.9 (1 C, $\mathbf{C_{Ar}}$), 129.0 (d, *J* = 3.3 Hz, 1 C, $\mathbf{C_{Ar}}$), 128.4 (1 C, $\mathbf{C_{Ar}}$), 125.6 (2 C, $\mathbf{C_{Ar}}$), 125.2 (2 C, $\mathbf{C_{Ar}}$), 122.5 (2 C, $\mathbf{C_{Ar}}$), 115.5 (d, *J* = 22.7 Hz, 1 C, $\mathbf{C_{Ar}}$), 62.7 (1 C, -**C**H₂), 48.8 (1 C, -**C**H), 48.7 (1 C, -**C**H), 34.2 (2 C, -**C**(CH₃)₃), 30.2 (6 C, -C(**C**H₃)₃), 26.1 (3 C, -SiC(**C**H₃)₃), 18.5 (1 C, -Si**C**(CH₃)₃), -5.2 (1 C, -Si**C**H₃), -5.3 (1 C, -Si**C**H₃).

HRMS (ESI-TOF): *m/z:* $[M+Na]^+$ calcd for $C_{42}H_{52}FNNaO_6Si^+$: 736.3440, found 736.3446.

Data for the cyclic product 6b:

HPLC (YMC-SB, *n*-hexane/IPA = 10/1, flow = 1.0 mL min^{-1}, T_{Column} = 10 °C, *l* = 240 nm): t_r*(trans):* 5.98 min (major), 6.54 min (minor); t_r*(cis):* 10.97 min (major), 23.04 min (minor).

α_D^{20}(c = 1, $CHCl_3$): -33.5 (mixture of diastereomers).

HRMS (ESI-TOF): *m/z:* $[M+NH_4]^+$ calcd for $C_{30}H_{37}FNO_3^+$: 478.2752, found 478.2754.

NMR data for the cis diastereomer:

^{1}H-NMR (500 MHz, $CDCl_3$, 298 K) *δ* / ppm = 7.24-7.22 (m, 4 H, Ar-**H**), 7.08-7.05 (m, 2 H, Ar-**H**), 6.95-6.87 (m, 2 H, Ar-**H**), 6.54 (s, 2 H, Ar-**H**), 5.95 (d, *J* = 15.1 Hz, 1 H, -C**H$_2$**), 5.49 (d, *J* = 3.3 Hz, 1 H, -C**H**), 5.21 (d, *J* = 15.1 Hz, 1 H, -C**H$_2$**), 5.13 (s, 1 H, -O**H**), 4.28 (d, *J* = 3.3 Hz, 1 H, -C**H**), 1.30 (s, 18 H, -C**H$_3$**).

^{19}F-NMR (470 MHz, $CDCl_3$, 298 K) *δ* / ppm = -119.7 (1 F, Ar-**F**).

^{13}C-NMR (125 MHz, $CDCl_3$, 298 K) *δ* / ppm = 171.7 (1 C, -**C**OOR), 160.1 (d, *J* = 244.2 Hz, 1 C, **C$_{Ar}$**-F), 153.2 (1 C, **C$_{Ar}$**), 140.9 (1 C, **C$_{Ar}$**), 135.2 (2 C, **C$_{Ar}$**), 133.9 (d, *J* = 2.7 Hz, 1 C, **C$_{Ar}$**), 132. 5 (1 C, **C$_{Ar}$**), 132.4 (1 C, **C$_{Ar}$**), 130.9 (1 C, **C$_{Ar}$**), 129.0 (1 C, **C$_{Ar}$**), 128.9 (d, *J* = 8.0 Hz, 1 C, **C$_{Ar}$**), 128.1 (1 C, **C$_{Ar}$**), 127.1 (2 C, **C$_{Ar}$**), 126.6 (1 C, **C$_{Ar}$**), 123.7 (d, *J* = 11.3 Hz, 1 C, **C$_{Ar}$**), 123.0 (d, *J* = 2.5 Hz, 1 C, **C$_{Ar}$**), 114.5 (d, *J* = 24.9 Hz, 1 C, **C$_{Ar}$**), 70.8 (1 C, -**C**H$_2$), 53.8 (1 C, -**C**H), 40.2 (d, *J* = 3.9 Hz, 1 C, -**C**H), 34.4 (2 C, -**C**(CH_3)$_3$), 30.3 (6 C, -**C**H$_3$).

NMR data for the trans diastereomer:

^{1}H-NMR (500 MHz, $CDCl_3$, 298 K) *δ* / ppm = 7.56 (t, *J* = 7.2 Hz, 1 H, Ar-**H**), 7.30-7.26 (m, 1 H, Ar-**H**), 7.24-7.14 (m, 4 H, Ar-**H**), 7.10 (d, *J* = 7.4 Hz, 1 H, Ar-**H**), 6.84-6.80 (m, 1 H, Ar-**H**), 6.51 (s, 2 H, Ar-**H**), 6.05 (d, *J* = 13.7 Hz, 1 H, -C**H$_2$**), 5.10 (d, *J* = 13.7 Hz, 1 H, -C**H$_2$**), 5.03 (d, *J* = 11.5 Hz, 1 H, -C**H**), 4.98 (s, 1 H, -O**H**), 4.55 (d, *J* = 11.5 Hz, 1 H, -C**H**), 1.22 (s, 18 H, -C**H$_3$**).

^{19}F-NMR (470 MHz, $CDCl_3$, 298 K) *δ* / ppm = -117.7 (1 F, Ar-**F**).

^{13}C-NMR (125 MHz, $CDCl_3$, 298 K) *δ* / ppm = 171.8 (1 C, -**C**OOR), 162.7 (d, *J* = 294.4 Hz, 1 C, **C$_{Ar}$**-F), 152.4 (1 C, **C$_{Ar}$**), 141.0 (1 C, **C$_{Ar}$**), 135.6 (2 C, **C$_{Ar}$**), 134.0 (1 C, **C$_{Ar}$**), 133.3 (1 C, **C$_{Ar}$**), 132.4 (1 C, **C$_{Ar}$**), 131.8 (d, *J* = 3.6 Hz, 1 C, **C$_{Ar}$**), 129. 7 (1 C, **C$_{Ar}$**), 129.6 (1 C, **C$_{Ar}$**), 129.0 (d, *J* = 9.1 Hz, 1 C, **C$_{Ar}$**), 126.9 (1 C, **C$_{Ar}$**), 125.1 (d, *J* = 12.7 Hz, 1 C, **C$_{Ar}$**), 124.9 (2 C, **C$_{Ar}$**), 124.0 (d, *J* = 3.4 Hz, 1 C, **C$_{Ar}$**), 115.0 (d, *J* = 22.8 Hz, 1 C, **C$_{Ar}$**), 70.5 (1 C, -**C**H$_2$), 53.5 (1 C, -**C**H), 46.8 (1 C, -**C**H), 34.2 (2 C, -**C**CH_3)$_3$), 30.2 (6 C, -**C**H$_3$).

4-Fluorophenyl benzo[c]oxepinone derivative (6c)

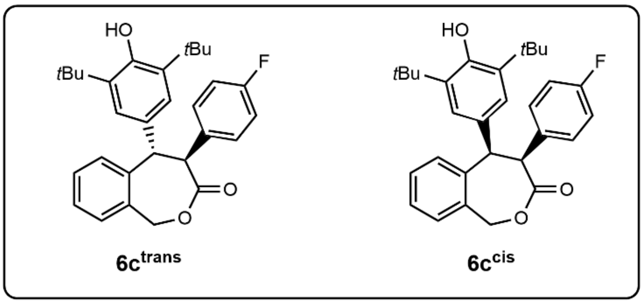

7-membered ring **6c** was synthesized according to general procedure **F** and obtained as a colorless residue in a yield of 68% (68% for the alkylation step) as a mixture of diastereomers (*dr* 55:45; er_{trans} 95:5; er_{cis} 91:9).

Data for the alkylation product (mixture of diastereomers) 5c:

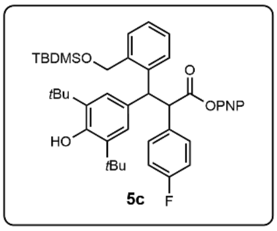

^{1}H-NMR (500 MHz, $CDCl_3$, 298 K) δ / ppm = *Signals for the unlike diastereomer:* 8.11 (d, *J* = 9.1 Hz, 2 H, Ar-**H**), 7.48-7.30 (m, 4 H, Ar-**H**), 7.24 (s, 2 H, Ar-**H**), 7.20-7.69 (m, 4 H, Ar-**H**), 6.61 (d, *J* = 9.1 Hz, 2 H, Ar-**H**), 5.17 (s, 2 H, -O**H**), 4.99 (d, *J* = 12.2 Hz, 1 H, -C**H₂**), 4.81 (d, *J* = 13.0 Hz, 1 H, -C**H**), 4.75 (d, *J* = 12.2 Hz, 1 H, -C**H₂**), 4.60 (d, *J* = 13.0 Hz, 1 H, -C**H**), 1.40 (s, 18 H, -C**H₃**), 0.97 (9 H, -SiC(C**H₃**)₃), 0.12 (s, 3 H, -SiC**H₃**), 0.09 (s, 3 H, -SiC**H₃**). *Selected signals for the like diastereomer:* 8.13 (d, *J* = 9.1 Hz, 2 H, Ar-**H**), 7.72 (d, *J* = 7.2 Hz, 1 H, Ar-**H**), 7.54 (d, *J* = 7.7 Hz, 1 H, Ar-**H**), 6.77 (d, *J* = 9.1 Hz, 2 H, Ar-**H**), 6.58 (s, 2 H, Ar-**H**), 5.00 (s, 1 H, -O**H**), 4.90 (d, *J* = 13.6 Hz, 1 H, -C**H**), 4.62 (d, *J* = 11.9 Hz, 1 H, -C**H₂**), 4.60 (d, *J* = 13.6 Hz,1 H, -C**H**), 4.46 (d, *J* = 11.9 Hz, 1 H, -C**H₂**), 1.24 (s, 18 H, -C**H₃**), 0.86 (s, 9 H, -SiC(C**H₃**)₃), -0.05 (s, 3 H, -SiC**H₃**), -0.06 (s, 3 H, -SiC**H₃**).

^{19}F-NMR (470 MHz, $CDCl_3$, 298 K) δ / ppm = -114.3 (1 F, Ar-**F**, *unlike*), -114.7 (1 F, Ar-**F**, *like*).

^{13}C-NMR (125 MHz, $CDCl_3$, 298 K) δ / ppm = *Signals for the unlike diastereomer:* 171.1 (1 C, -**C**OOR), 162.3 (1 C, $\mathbf{C_{Ar}}$-F), 155.3 (1 C, $\mathbf{C_{Ar}}$), 152.9 (1 C, $\mathbf{C_{Ar}}$), 145.5 (1 C, $\mathbf{C_{Ar}}$), 139.0 (1 C, $\mathbf{C_{Ar}}$), 137.9 (1 C, $\mathbf{C_{Ar}}$), 136.1 (2 C, $\mathbf{C_{Ar}}$), 131.8 (d, *J* = 3.2 Hz, 1 C, $\mathbf{C_{Ar}}$), 131.6 (1 C, $\mathbf{C_{Ar}}$), 130.4 (d, *J* = 8.1 Hz, 1 C, $\mathbf{C_{Ar}}$), 127.6 (1 C, $\mathbf{C_{Ar}}$), 127.1 (1 C, $\mathbf{C_{Ar}}$), 126.6 (1 C, $\mathbf{C_{Ar}}$), 126.3 (1 C, $\mathbf{C_{Ar}}$), 125.5 (2 C, $\mathbf{C_{Ar}}$), 125.3 (1 C, $\mathbf{C_{Ar}}$), 125.2 (1 C, $\mathbf{C_{Ar}}$), 125.1 (2 C, $\mathbf{C_{Ar}}$), 122.5 (2 C, $\mathbf{C_{Ar}}$), 115.7 (d, *J* = 21.7 Hz, 1 C, $\mathbf{C_{Ar}}$), 63.2 (1 C, -**C**H₂), 56.9 (1 C, -**C**H), 48.7 (1 C, -**C**H), 34.5 (2 C, -**C**(CH₃)₃), 30.4 (6 C, -C(**C**H₃)₃), 26.1 (3 C, -SiC(**C**H₃)₃), 18.6 (1 C, -Si**C**(CH₃)₃), -5.1 (1 C, -Si**C**H₃), -5.2 (1 C, -Si**C**H₃). *Selected signals for the like diastereomer:* 170.7 (1 C, -**C**OOR), 162.5 (d, *J* = 146.5 Hz, 1 C, $\mathbf{C_{Ar}}$-F), 152.4 (1 C, $\mathbf{C_{Ar}}$), 149.3 (1 C, $\mathbf{C_{Ar}}$), 147.9 (1 C, $\mathbf{C_{Ar}}$), 140.8 (1 C, $\mathbf{C_{Ar}}$), 139.6 (1 C, $\mathbf{C_{Ar}}$), 135.5 (2 C, $\mathbf{C_{Ar}}$), 132.4 (d, *J* = 4.5 Hz, 1 C, $\mathbf{C_{Ar}}$), 115.5 (d, *J* = 21.8 Hz, 1 H, $\mathbf{C_{Ar}}$), 62.8 (1 C, -**C**H₂), 57.4 (1 C, -**C**H), 50.9 (1 C, -**C**H), 34.2 (2 C, -**C**(CH₃)₃), 30.2 (6 C, -C(**C**H₃)₃), 26.0 (3 C, -SiC(**C**H₃)₃), 18.5 (1 C, -Si**C**(CH₃)₃), -5.2 (1 C, -Si**C**H₃), -5.4 (1 C, -Si**C**H₃).

HRMS (ESI-TOF): *m/z:* $[M+Na]^+$ calcd for $C_{42}H_{52}FNNaO_6Si^+$: 736.3440, found 736.3442.

Data for the cyclic product 6c:

HPLC (YMC-SB, *n*-hexane/IPA = 10/1, flow = 1.0 mL min^{-1}, T_{Column} = 10 °C, *l* = 240 nm): t_r*(trans):* 16.27 min (major), 25.46 min (minor); t_r*(cis):* 14.76 min (major), 40.88 min (minor).

α_D^{20}(c = 1, $CHCl_3$): -38.3 (mixture of diastereomers).

HRMS (ESI-TOF): *m/z:* $[M+NH_4]^+$ calcd for $C_{30}H_{37}FNO_3^+$: 478.2752, found 478.2756.

NMR data for the cis diastereomer:

^{1}H-NMR (500 MHz, $CDCl_3$, 298 K) *δ* / ppm = 7.24-7.22 (m, 3 H, Ar-**H**), 7.08-7.06 (m, 1 H, Ar-**H**), 6.98-6.91 (m, 4 H, Ar-**H**), 6.56 (s, 2 H, Ar-**H**), 5.90 (d, *J* = 15.1 Hz, 1 H, -C**H₂**), 5.22 (d, *J* = 15.1 Hz, 1 H, -C**H₂**), 5.15 (s, 1 H, -O**H**), 4.95 (d, *J* = 3.4 Hz, 1 H, -C**H**), 4.27 (d, *J* = 3.4 Hz, 1 H, -C**H**), 1.32 (s, 18 H, -C**H₃**).

^{19}F-NMR (470 MHz, $CDCl_3$, 298 K) *δ* / ppm = -115.1 (1 F, Ar-**F**).

^{13}C-NMR (125 MHz, $CDCl_3$, 298 K) *δ* / ppm = 172.1 (1 C, -**C**OOR), 162.3 (d, *J* = 246.1 Hz. 1 C, **C**$_{\mathbf{Ar}}$-F), 153.2 (1 C, **C**$_{\mathbf{Ar}}$), 141.0 (1 C, **C**$_{\mathbf{Ar}}$), 135.2 (2 C, **C**$_{\mathbf{Ar}}$), 132.6 (d, *J* = 2.4 Hz, 1 C, **C**$_{\mathbf{Ar}}$), 132.5 (1 C, **C**$_{\mathbf{Ar}}$), 132.4 (1 C, **C**$_{\mathbf{Ar}}$), 132.4 (d, *J* = 3.6 Hz, 1 C, **C**$_{\mathbf{Ar}}$), 130.7 (1 C, **C**$_{\mathbf{Ar}}$), 129.1 (2 C, **C**$_{\mathbf{Ar}}$), 128.1 (1 C, **C**$_{\mathbf{Ar}}$), 127.1 (2 C, **C**$_{\mathbf{Ar}}$), 126.7 (1 C, **C**$_{\mathbf{Ar}}$), 114.5 (1 C, **C**$_{\mathbf{Ar}}$), 114.4 (1 C, **C**$_{\mathbf{Ar}}$), 70.7 (1 C, -**C**H_2), 55.2 (1 C, -**C**H), 49.4 (1 C, -**C**H), 34.4 (2 C, -**C**$(CH_3)_3$), 30.3 (6 C, -**C**H_3).

NMR data for the trans diastereomer:

^{1}H-NMR (500 MHz, $CDCl_3$, 298 K) *δ* / ppm = 7.28-7.21 (m, 3 H, Ar-**H**), 7.14-7.09 (m, 3 H, Ar-**H**), 6.97-6.93 (m, 2 H, Ar-**H**), 6.43 (s, 2 H, Ar-**H**), 5.92 (d, *J* = 13.7 Hz, 1 H, -C**H₂**), 5.06 (d, *J* = 13.7 Hz, 1 H, -C**H₂**), 5.01 (s, 1 H, -O**H**), 4.50 (d, *J* = 10.8 Hz, 1 H, -C**H**), 4.46 (d, *J* = 10.8 Hz, 1 H, -C**H**), 1.23 (s, 18 H, -C**H₃**).

^{19}F-NMR (470 MHz, $CDCl_3$, 298 K) *δ* / ppm = -115.5 (1 F, Ar-**F**).

^{13}C-NMR (125 MHz, $CDCl_3$, 298 K) *δ* / ppm = 172.5 (1 C, -**C**OOR), 162.2 (d, *J* = 247.3 Hz, 1 C, **C**$_{\mathbf{Ar}}$-F), 152.5 (1 C, **C**$_{\mathbf{Ar}}$), 140.8 (1 C, **C**$_{\mathbf{Ar}}$), 135.7 (2 C, **C**$_{\mathbf{Ar}}$), 134.4 (1 C, **C**$_{\mathbf{Ar}}$), 133.6 (d, *J* = 3.5 Hz, 1 C, **C**$_{\mathbf{Ar}}$), 133.4 (1 C, **C**$_{\mathbf{Ar}}$), 132.5 (1 C, **C**$_{\mathbf{Ar}}$), 131.4 (1 C, **C**$_{\mathbf{Ar}}$), 131.4 (1 C, **C**$_{\mathbf{Ar}}$), 129.8 (1 C, **C**$_{\mathbf{Ar}}$), 129.5 (1 C, **C**$_{\mathbf{Ar}}$), 127.0 (1 C, **C**$_{\mathbf{Ar}}$), 124.9 (2 C, **C**$_{\mathbf{Ar}}$), 115.2 (1 C, **C**$_{\mathbf{Ar}}$), 115.0 (1 C, **C**$_{\mathbf{Ar}}$), 70.3 (1 C, -**C**H_2), 54.9 (1 C, -**C**H), 54.6 (1 C, -**C**H), 34.3 (2 C, -**C**$CH_3)_3$), 30.2 (6 C, -**C**H_3).

3-Chlorophenyl benzo[c]oxepinone derivative (6d)

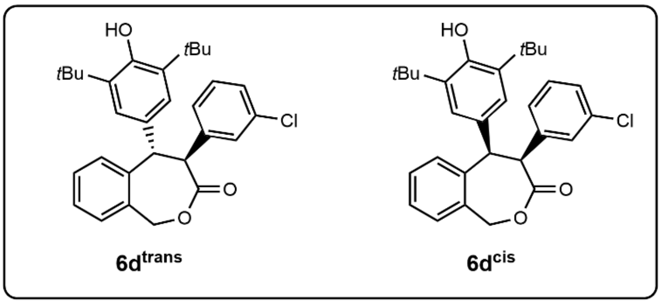

7-membered ring **6d** was synthesized according to general procedure **F** and obtained as a colorless residue in a yield of 58% (72% for the alkylation step) as a mixture of diastereomers (*dr* 50:50; er_{trans} 90:10; er_{cis} 82:18).

Data for the alkylation product (mixture of diastereomers) 5d:

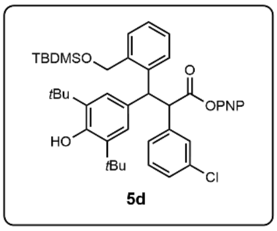

^{1}H-NMR (500 MHz, $CDCl_3$, 298 K) δ / ppm = *Signals for the unlike diastereomer:* 8.11 (d, J = 9.1 Hz, 2 H, Ar-**H**), 7.50-7.32 (m, 4 H, Ar-**H**), 7.22 (s, 2 H, Ar-**H**), 7.21-7.10 (m, 4 H, Ar-**H**), 6.62 (d, J = 9.1 Hz, 2 H, Ar-**H**), 5.17 (s, 1 H, -O**H**), 4.96 (d, J = 12.2 Hz, 1 H, -C**H₂**), 4.79 (d, J = 13.1 Hz, 1 H -C**H**), 4.73 (d, J = 12.2 Hz, 1 H, -C**H₂**), 4.62 (d, J = 13.1 Hz, 1 H, -C**H**), 1.39 (s, 18 H, -C**H₃**), 0.96 (s, 9 H, -SiC(C**H₃**), 0.11 (s, 3 H, -SiC**H₃**), 0.09 (s, 3 H, -SiC**H₃**). *Selected signals for the like diastereomer:* 8.13 (d, J = 9.1 Hz, 2 H, Ar-**H**), 7.71 (d, J = 7.2 Hz, 1 H, Ar-**H**), 7.53 (d, J = 8.2 Hz, 1 H, Ar-**H**), 6.79 (d, J = 9.1 Hz, 2 H, Ar-**H**), 6.60 (s, 2 H, Ar-**H**), 4.97 (s, 1 H, -O**H**), 4.89 (d, J = 13.5 Hz, 1 H, -C**H**), 4.64 (d, J = 11.6 Hz, 1 H, -C**H₂**), 4.59 (d, J = 13.5 Hz, 1 H, -C**H**), 4.45 (d, J = 11.8 Hz, 1 H, -C**H₂**), 1.25 (s, 18 H, -C**H₃**), 0.86 (s, 9 H, -SiC(C**H₃**)₃), -0.03 (s, 3 H, -SiC**H₃**), -0.05 (s, 3 H, -SiC**H₃**).

^{13}C-NMR (125 MHz, $CDCl_3$, 298 K) δ / ppm = *Signals for the unlike diastereomer:* 170.7 (1 C, -**C**OOR), 155.3 (1 C, $\mathbf{C_{Ar}}$), 153.0 (1 C, $\mathbf{C_{Ar}}$), 145.6 (1 C, $\mathbf{C_{Ar}}$), 139.0 (1 C, $\mathbf{C_{Ar}}$), 138.0 (1 C, $\mathbf{C_{Ar}}$), 137.5 (1 C, $\mathbf{C_{Ar}}$), 136.1 (2 C, $\mathbf{C_{Ar}}$), 134.5 (1 C, $\mathbf{C_{Ar}}$), 131.4 (1 C, $\mathbf{C_{Ar}}$), 130.0 (1 C, $\mathbf{C_{Ar}}$), 128.9 (1 C, $\mathbf{C_{Ar}}$), 128.2 (1 C, $\mathbf{C_{Ar}}$), 127.4 (1 C, $\mathbf{C_{Ar}}$), 127.1 (1 C, $\mathbf{C_{Ar}}$), 127.0 (1 C, $\mathbf{C_{Ar}}$), 126.7 (1 C, $\mathbf{C_{Ar}}$), 126.2 (1 C, $\mathbf{C_{Ar}}$), 125.5 (2 C, $\mathbf{C_{Ar}}$), 125.1 (2 C, $\mathbf{C_{Ar}}$), 122.5 (2 C, $\mathbf{C_{Ar}}$), 63.1 (1 C, -**C**H_2), 57.4 (1 C, -C**H₂**), 48.6 (1 C, -**C**H_2), 34.5 (2 C, -**C**$(CH_3)_3$), 30.4 (6 C, -C(**C**$H_3)_3$), 26.2 (3 C, -SiC(**C**$H_3)_3$), 18.6 (1 C, -Si**C**$(CH_3)_3$), -5.1 (1 C, -Si**C**H_3), -5.2 (1 C, -Si**C**H_3). *Selected signals for the like diastereomer:* 170.2 (1 C, -**C**OOR), 155.3 (1 C, $\mathbf{C_{Ar}}$), 152.5 (1 C, $\mathbf{C_{Ar}}$), 145.6 (1 C, $\mathbf{C_{Ar}}$), 139.7 (1 C, $\mathbf{C_{Ar}}$), 138.9 (1 C, $\mathbf{C_{Ar}}$), 138.7 (1 C, $\mathbf{C_{Ar}}$), 135.6 (2 C, $\mathbf{C_{Ar}}$), 129.8 (1 C, $\mathbf{C_{Ar}}$), 129.6 (1 C, $\mathbf{C_{Ar}}$), 129.1 (1 C, $\mathbf{C_{Ar}}$), 127.9 (1 C, $\mathbf{C_{Ar}}$), 127.6 (1 C, $\mathbf{C_{Ar}}$), 126.8 (1 C, $\mathbf{C_{Ar}}$), 125.3 (2 C, $\mathbf{C_{Ar}}$), 125.2 (2 C, $\mathbf{C_{Ar}}$), 122.5 (2 C, $\mathbf{C_{Ar}}$), 62.9 (1 C, -**C**H_2), 57.8 (1 C, -**C**H), 50.8 (1 C, -**C**H), 34.2 (2 C, -**C**$(CH_3)_3$), 30.2 (6 C, -C(**C**$H_3)_3$), 26.0 (3 C, -SiC(**C**$H_3)_3$), 18.5 (1 C, -Si**C**$(CH_3)_3$), -5.2 (1 C, -Si**C**H_3), -5.3 (1 C, -Si**C**H_3).

HRMS (ESI-TOF): *m/z:* $[M+Na]^+$ calcd for $C_{42}H_{52}ClNNaO_6Si^+$: 752.3145, found 752.3149.

Data for the cyclic product 6d:

HPLC (YMC-SB, *n*-hexane/IPA = 10/1, flow = 1.0 mL min^{-1}, T_{Column} = 10 °C, *l* = 220 nm)): t_r*(trans):* 13.94 min (major), 30.75 min (minor); t_r*(cis):* 17.99 min (major), 27.52 min (minor).

$\boldsymbol{\alpha_D^{20}}$(c = 1, $CHCl_3$): -31.0 (mixture of diastereomers).

HRMS (ESI-TOF): *m/z:* $[M+NH_4]^+$ calcd for $C_{30}H_{37}ClNO_3^+$: 494.2456, found 494.2457.

NMR data for the cis diastereomer:

^{1}H-NMR (500 MHz, $CDCl_3$, 298 K) *δ* / ppm = 7.27 (br. app. s, 1 H, Ar-**H**), 7.24-7.23 (m, 3 H, Ar-**H**), 7.18 (t, *J* = 7.9 Hz, 1 H, Ar-**H**), 7.07-7.05 (m, 2 H, Ar-**H**), 6.91 (d, *J* = 8.1 Hz, 1 H, Ar-**H**), 6.57 (s, 2 H, Ar-**H**), 5.89 (d, *J* = 15.1 Hz, 1 H, -C**H₂**), 5.22 (d, *J* = 15.1 Hz, 1 H, -C**H₂**), 5.16 (s, 1 H, -O**H**), 4.92 (d, *J* = 3.3 Hz, 1 H, -C**H**), 4.30 (d, *J* = 3.3 Hz, 1 H, -C**H**), 1.32 (s, 18 H, -C**H₃**).

^{13}C-NMR (125 MHz, $CDCl_3$, 298 K) *δ* / ppm = 171.6 (1 C, -**C**OOR), 153.3 (1 C, $\mathbf{C_{Ar}}$), 140.9 (1 C, $\mathbf{C_{Ar}}$), 138.7 (1 C, $\mathbf{C_{Ar}}$), 135.4 (2 C, $\mathbf{C_{Ar}}$), 133.4 (1 C, $\mathbf{C_{Ar}}$), 132.3 (2 C, $\mathbf{C_{Ar}}$), 131.0 (1 C, $\mathbf{C_{Ar}}$), 130.5 (1 C, $\mathbf{C_{Ar}}$), 129.1 (1 C, $\mathbf{C_{Ar}}$), 129.0 (1 C, $\mathbf{C_{Ar}}$), 128.9 (1 C, $\mathbf{C_{Ar}}$), 128.1 (1 C, $\mathbf{C_{Ar}}$), 127.8 (1 C, $\mathbf{C_{Ar}}$), 127.0 (2 C, $\mathbf{C_{Ar}}$), 126.7 (1 C, $\mathbf{C_{Ar}}$), 70.7 (1 C, -**C**H_2), 54.9 (1 C, -**C**H), 49.9 (1 C, -**C**H), 34.4 (2 C, -**C**$(CH_3)_3$), 30.3 (6 C, -**C**H_3).

NMR data for the trans diastereomer:

^{1}H-NMR (500 MHz, $CDCl_3$, 298 K) *δ* / ppm = 7.29-7.26 (m, 2 H, Ar-**H**), 7.23-7.18 (m, 3 H, Ar-**H**), 7.11-7.10 (m, 3 H, Ar-**H**), 6.44 (s, 2 H, Ar-**H**), 5.90 (d, *J* = 13.8 Hz, 1 H, -C**H₂**), 5.07 (d, *J* = 13.8 Hz, 1 H, -C**H₂**), 5.02 (s, 1 H, -O**H**), 4.48 (app. s, 2 H, -C**H**), 1.23 (s, 18 H, -C**H₃**).

^{13}C-NMR (125 MHz, $CDCl_3$, 298 K) *δ* / ppm = 172.0 (1 C, -**C**OOR), 152.5 (1 C, $\mathbf{C_{Ar}}$), 140.7 (1 C, $\mathbf{C_{Ar}}$), 139.8 (1 C, $\mathbf{C_{Ar}}$), 135.8 (2 C, $\mathbf{C_{Ar}}$), 134.1 (1 C, $\mathbf{C_{Ar}}$), 133.9 (1 C, $\mathbf{C_{Ar}}$), 133.3 (1 C, $\mathbf{C_{Ar}}$), 132.5 (1 C, $\mathbf{C_{Ar}}$), 130.2 (1 C, $\mathbf{C_{Ar}}$), 129.8 (1 C, $\mathbf{C_{Ar}}$), 129.6 (1 C, $\mathbf{C_{Ar}}$), 129.4 (1 C, $\mathbf{C_{Ar}}$), 128.1 (1 C, $\mathbf{C_{Ar}}$), 127.6 (1 C, $\mathbf{C_{Ar}}$), 127.0 (1 C, $\mathbf{C_{Ar}}$), 124.9 (2 C, $\mathbf{C_{Ar}}$), 70.4 (1 C, -**C**H_2), 55.3 (1 C, -**C**H), 54.4 (1 C, -**C**H), 34.3 (2 C, -**C**$CH_3)_3$, 30.2 (6 C, -C**H₃**).

4-Chlorophenyl benzo[c]oxepinone derivative (6e)

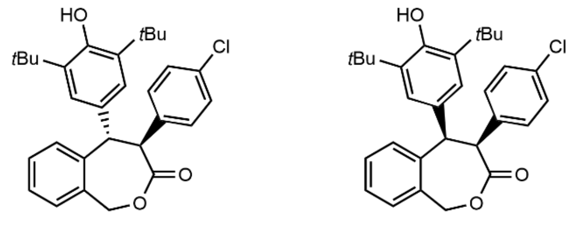

$\mathbf{6e^{trans}}$ $\mathbf{6e^{cis}}$

7-membered ring 6ewas synthesized according to general procedure **F** and obtained as a colorless residue in a yield of 64% (72% for the alkylation step) as a mixture of diastereomers (*dr* 55:45; er_{trans} 90:10; er_{cis} 92:8).

Data for the alkylation product (mixture of diastereomers) 5e:

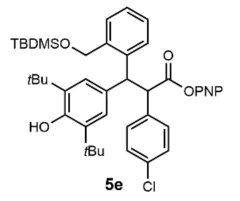

^{1}H-NMR (300 MHz, $CDCl_3$, 298 K) *δ* / ppm = *Signals for the unlike diastereomer:* 8.11 (d, *J* = 9.1 Hz, 2 H, Ar-**H**), 7.45-7.29 (m, 4 H, Ar-**H**), 7.23 (s, 2 H, Ar-**H**), 7.21-7.06 (4 H, Ar-**H**), 6.61 (d, *J* = 9.1 Hz, 2 H, Ar-**H**), 5.18 (s. 1 H, -O**H**), 5.01 (d, *J* = 12.2 Hz, 1 H, -C$\mathbf{H_2}$), 4.82 (d, *J* = 14.0 Hz, 1 H, -C**H**), 4.74 (d, *J* = 12.2 Hz, 1 H, -C$\mathbf{H_2}$), 4.60 (d, *J* = 13.0 Hz, 1 H, -C**H**), 1.40 (s, 18 H, -C$\mathbf{H_3}$), 0.97 (s, 9 H, -SiC(C$\mathbf{H_3}$)$_3$), 0.13 (s, 3 H, -SiC$\mathbf{H_3}$), 0.10 (s, 3 H, -SiC$\mathbf{H_3}$). *Selected signals for the like diastereomer:* 8.13 (d, *J* = 9.1 Hz, 2 H, Ar-**H**), 7.72 (d, *J* = 8.5 Hz, 1 H, Ar-**H**), 7.54 (d, *J* = 7.0 Hz, 1 H, Ar-**H**), 6.78 (d, *J* = 9.1 Hz, 2 H, Ar-**H**), 6.57 (s, 2 H, Ar-**H**), 4.97 (s, 1 H, -O**H**), 4.89 (d, *J* = 13.5 Hz, 1 H, -C**H**), 4.61 (d, *J* = 11.8 Hz, 1 H, -C**H**), 4.59 (d, *J* = 13.5 Hz, 1 H, -C**H**), 4.45 (d, *J* = 11.8 Hz, 1 H, -C$\mathbf{H_2}$), 1.25 (s, 18 H, -C$\mathbf{H_3}$), 0.86 (s, 9 H, -SiC(C$\mathbf{H_3}$)$_3$), -0.05 (s, 3 H, -SiC$\mathbf{H_3}$), -0.06 (s, 3 H, -SiC$\mathbf{H_3}$).

^{13}C-NMR (75 MHz, $CDCl_3$, 298 K) *δ* / ppm = *Signals for the unlike diastereomer:* 170.9 (1 C, -**C**OOR), 155.3 (1 C, $\mathbf{C_{Ar}}$), 153.0 (1 C, $\mathbf{C_{Ar}}$), 145.5 (1 C, $\mathbf{C_{Ar}}$), 139.0 (1 C, $\mathbf{C_{Ar}}$), 137.8 (1 C, $\mathbf{C_{Ar}}$), 136.1 (2 C, $\mathbf{C_{Ar}}$), 134.6 (1 C, $\mathbf{C_{Ar}}$), 133.8 (1 C, $\mathbf{C_{Ar}}$), 131.5 (1 C, $\mathbf{C_{Ar}}$), 130.1 (2 C, $\mathbf{C_{Ar}}$), 129.0 (2 C, $\mathbf{C_{Ar}}$), 127.7 (1 C, $\mathbf{C_{Ar}}$), 127.2 (1 C, $\mathbf{C_{Ar}}$), 126.7 (1 C, $\mathbf{C_{Ar}}$), 126.3 (1 C, $\mathbf{C_{Ar}}$), 125.5 (2 C, $\mathbf{C_{Ar}}$), 125.1 (2 C, $\mathbf{C_{Ar}}$), 122.5 (2 C, $\mathbf{C_{Ar}}$), 63.3 (1 C, -**C**H_2), 57.1 (1 C, -**C**H), 48.5 (1 C, -**C**H), 34.5 (2 C, -**C**(CH_3)$_3$), 30.4 (6 C, -C(**C**H_3)$_3$), 26.1 (3 C, -SiC(**C**H_3)$_3$), 18.6 (1 C, -Si**C**(CH_3)$_3$), -5.1 (1 C, -Si**C**H_3), -5.2 (1 C, -Si**C**H_3). *Selected signals for the like diastereomer:* 170.4 (1 C, -**C**OOR), 152.4 (1 C, $\mathbf{C_{Ar}}$), 139.6 (1 C, $\mathbf{C_{Ar}}$), 138.9 (1 C, $\mathbf{C_{Ar}}$), 135.6 (2 C, $\mathbf{C_{Ar}}$), 135.2 (1 C, $\mathbf{C_{Ar}}$), 133.9 (1 C, $\mathbf{C_{Ar}}$), 128.7 (2 C, $\mathbf{C_{Ar}}$), 127.5 (1 C, $\mathbf{C_{Ar}}$), 127.1 (1 C, $\mathbf{C_{Ar}}$), 127.0 (1 C, $\mathbf{C_{Ar}}$), 125.3 (2 C, $\mathbf{C_{Ar}}$), 125.2 (2 C, $\mathbf{C_{Ar}}$), 122.5 (2 C, $\mathbf{C_{Ar}}$), 62.8 (1 C, -**C**H_2), 57.5 (1 C, -**C**H), 50.9 (1 C, -**C**H), 34.2 (2 C, -**C**(CH_3)$_3$), 30.2 (6 C, -C(**C**H_3)$_3$), 26.0 (3 C, -SiC(**C**H_3)$_3$), 18.5 (1 C, -Si**C**(CH_3)$_3$), -5.2 (1 C, -Si**C**H_3), -5.4 (1 C, -Si**C**H_3).

HRMS (ESI-TOF): *m/z:* $[M+Na]^+$ calcd for $C_{42}H_{52}ClNNaO_6Si^+$: 752.3145, found 752.3147.

Data for the cyclic product 6e:

HPLC (YMC-SB, *n*-hexane/IPA = 10/1, flow = 1.0 mL min^{-1}, T_{Column} = 10 °C, *l* = 240 nm) $t_r(trans)$: 15.55 min (major), 27.14 min (minor); $t_r(cis)$: 14.54 min (major), 41.89 min (minor).

α_D^{20}(c = 1, $CHCl_3$): -37.9 (mixture of diastereomers).

HRMS (ESI-TOF): *m/z:* $[M+NH_4]^+$ calcd for $C_{30}H_{37}ClNO_3^+$: 494.2456, found 494.2456.

NMR data for the cis diastereomer:

^{1}H-NMR (500 MHz, $CDCl_3$, 298 K) *δ* / ppm = 7.24-7.22 (m, 3 H, Ar-**H**), 7.21 (d, *J* = 8.5 Hz, 2 H, Ar-**H**), 7.07-7.06 (m, 1 H, Ar-**H**), 6.94 (d, *J* = 8.5 Hz, 2 H Ar-**H**), 6.55 (s, 2 H, Ar-**H**), 5.90 (d, *J* = 15.1 Hz, 1 H, -C**H$_2$**), 5.21 (d, *J* = 15.1 Hz, 1 H, -C**H$_2$**), 5.14 (s, 1 H, -O**H**), 4.93 (d, *J* = 3.2 Hz, 1 H, -C**H**), 4.27 (d, *J* = 3.2 Hz, 1 H, -C**H**), 1.32 (s, 18 H, -C**H$_3$**).

^{13}C-NMR (125 MHz, $CDCl_3$, 298 K) *δ* / ppm = 171.8 (1 C, -**C**OOR), 153.3 (1 C, **C$_{Ar}$**), 140.9 (1 C, **C$_{Ar}$**), 135.4 (1 C, **C$_{Ar}$**), 135.3 (2 C, **C$_{Ar}$**), 133.5 (1 C, **C$_{Ar}$**), 132.4 (1 C, **C$_{Ar}$**), 132.4 (1 C, **C$_{Ar}$**), 132.2 (2 C, **C$_{Ar}$**), 130.6 (1 C, **C$_{Ar}$**), 129.1 (1 C, **C$_{Ar}$**), 128.1 (1 C, **C$_{Ar}$**), 127.8 (2 C, **C$_{Ar}$**), 127.1 (1 C, **C$_{Ar}$**), 126.7 (2 C, **C$_{Ar}$**), 70.7 (1 C, -**C**H$_2$), 55.1 (1 C, -**C**H), 49.6 (1 C, -**C**H), 34.4 (2 C, -**C**CH$_3$), 30.3 (6 C, -**C**H$_3$).

NMR data for the trans diastereomer:

^{1}H-NMR (500 MHz, $CDCl_3$, 298 K) *δ* / ppm = 7.28-7.20 (m, 5 H, Ar-**H**), 7.10-7.08 (m, 3 H, Ar-**H**), 6.42 (s, 2 H, Ar-**H**), 5.91 (d, *J* = 13.7 Hz, 1 H, -C**H$_2$**), 5.06 (d, *J* = 13.7 Hz, 1 H, -C**H$_2$**), 5.01 (s, 1 H, -O**H**), 4.50 (d, *J* = 10.8 Hz, 1 H, -C**H**), 4.46 (d, *J* = 10.8 Hz, 1 H, -C**H**), 1.23 (s, 18 H, -C**H$_3$**).

^{13}C-NMR (125 MHz, $CDCl_3$, 298 K) *δ* / ppm = 172.2 (1 C, -**C**OOR), 152.5 (1 C, **C$_{Ar}$**), 140.7 (1 C, **C$_{Ar}$**), 136.4 (1 C, **C$_{Ar}$**), 135.8 (2 C, **C$_{Ar}$**), 134.3 (1 C, **C$_{Ar}$**), 133.4 (1 C, **C$_{Ar}$**), 133.4 (1 C, **C$_{Ar}$**), 132.5 (1 C, **C$_{Ar}$**), 131.2 (2 C, **C$_{Ar}$**), 129.7 (1 C, **C$_{Ar}$**), 129.5 (1 C, **C$_{Ar}$**), 128.3 (2 C, **C$_{Ar}$**), 127.0 (1 C, **C$_{Ar}$**), 124.9 (2 C, **C$_{Ar}$**), 70.4 (1 C, -**C**H$_2$), 55.0 (1 C, -**C**H), 54.5 (1 C, -**C**H), 34.3 (2 C, -**C**CH$_3$), 30.2 (6 C, -**C**H$_3$).

3,4-Dichlorophenyl benzo[c]oxepinone derivative (6f)

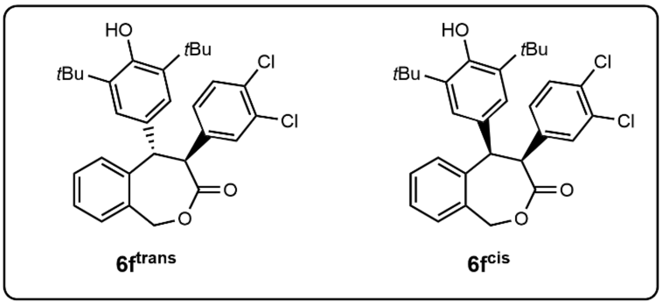

7-membered ring **6f** was synthesized according to general procedure **F** and obtained as a colorless residue in a yield of 59% (71% for the alkylation step) as a mixture of diastereomers (*dr* 45:55; $er_{trans'}$ 79:21; er_{cis} 65:35).

Data for the alkylation product (mixture of diastereomers) 5f:

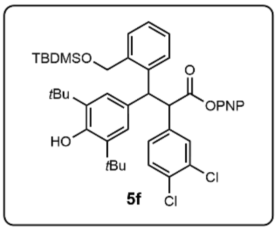

^{1}H-NMR (500 MHz, $CDCl_3$, 298 K) *δ* / ppm = *Signals for the unlike diastereomer:* 8.11 (d, *J* = 9.0 Hz, 2 H, Ar-**H**), 7.60 (d, *J* = 2.0 Hz, 1 H, Ar-**H**), 7.40-7.28 (m, 4 H, Ar-**H**), 7.21 (s, 2 H, Ar-**H**), 7.14 (t, *J* = 7.3 Hz, 1 H, Ar-**H**), 7.06 (dd, J_1 = 2.0 Hz, J_2 = 8.3 Hz, 1 H, Ar-**H**), 6.61 (d, *J* = 9.0 Hz, 2 H, Ar-**H**), 5.18 (s, 1 H, -O**H**), 4.96 (d, *J* = 12.2 Hz, 1 H, -C**H₂**), 4.81 (d, *J* = 13.0 Hz, 1 H, -C**H**), 4.71 (d, *J* = 12.2 Hz, 1 H, -C**H₂**), 4.60 (d, *J* = 13.0 Hz, 1 H, -C**H**), 1.39 (s, 18 H, -C**H₃**), 0.96 (s, 9 H, -SiC(C**H₃**)₃), 0.12 (s, 3 H, -SiC**H₃**), 0.09 (s, 3 H, -SiC**H₃**). *Selected signals for the like diastereomer:* 8.14 (d, *J* = 9.0 Hz, 2 H, Ar-**H**), 7.70 (d, *J* = 7.8 Hz, 1 H, Ar-**H**), 7.52 (d, *J* = 7.3 Hz, 1 H, Ar-**H**), 7.24 (d, *J* = 2.0 Hz, 1 H, Ar-**H**), 6.79 (d, *J* = 9.0 Hz, 2 H, Ar-**H**), 6.59 (s, 2 H, Ar-**H**), 5.00 (s, 1 H, -O**H**), 4.87 (d, *J* = 13.4 Hz, 1 H, -C**H**), 4.61 (d, *J* = 11.9 Hz, 1 H, -C**H₂**), 4.57 (d, *J* = 13.4 Hz, 1 H, -C**H**), 4.43 (d, *J* = 11.9 Hz, 1 H, -C**H₂**), 1.26 (s, 18 H, -C**H₃**), 0.86 (s, 9 H, -SiC(C**H₃**)₃), -0.03 (s, 3 H, -SiC**H₃**), -0.06 (s, 3 H, -SiC**H₃**).

^{13}C-NMR (125 MHz, $CDCl_3$, 298 K) *δ* / ppm = *Signals for the unlike diastereomer:* 170.0 (1 C, -**C**OOR), 155.1 (1 C, C_{Ar}), 153.1 (1 C, C_{Ar}), 145.6 (1 C, C_{Ar}), 139.7 (1 C, C_{Ar}), 139.0 (1 C, C_{Ar}), 137.5 (1 C, C_{Ar}), 136.3 (1 C, C_{Ar}), 136.2 (2 C, C_{Ar}), 132.8 (1 C, C_{Ar}), 132.2 (1 C, C_{Ar}), 131.1 (1 C, C_{Ar}), 130.7 (1 C, C_{Ar}), 130.7 (1 C, C_{Ar}), 128.2 (1 C, C_{Ar}), 127.8 (1 C, C_{Ar}), 127.3 (1 C, C_{Ar}), 126.9 (1 C, C_{Ar}), 125.3 (2 C, C_{Ar}), 125.1 (2 C, C_{Ar}), 122.5 (2 C, C_{Ar}), 63.3 (1 C, -**C**H₂), 56.8 (1 C, -**C**H), 48.6 (1 C, -**C**H), 34.5 (2 C, -**C**(CH₃)₃), 30.4 (6 C, -C(**C**H₃)₃), 26.2 (3 C, -SiC(**C**H₃)₃), 18.7 (1 C, -Si**C**(CH₃)₃), -5.1 (1 C, -Si**C**H₃), -5.2 (1 C, -Si**C**H₃). *Selected signals for the like diastereomer:* 170.5 (1 C, -**C**OOR), 155.2 (1 C, C_{Ar}), 152.6 (1 C, C_{Ar}), 145.7 (1 C, C_{Ar}), 138.6 (1 C, C_{Ar}), 136.9 (1 C, C_{Ar}), 135.8 (2 C, C_{Ar}), 132.7 (1 C, C_{Ar}), 132.1 (1 C, C_{Ar}), 130.9 (1 C, C_{Ar}), 130.4 (1 C, C_{Ar}), 129.4 (1 C, C_{Ar}), 127.9 (1 C, C_{Ar}), 127.8 (1 C, C_{Ar}), 127.3 (1 C, C_{Ar}), 127.1 (1 C, C_{Ar}), 125.5 (2 C, C_{Ar}), 62.9 (1 C, -**C**H₂), 57.2 (1 C, -**C**H), 51.0 (1 C, -**C**H), 34.2 (2 C, -**C**(CH₃)₃), 30.2 (6 C, -C(**C**H₃)₃), 26.0 (3 C, -SiC(**C**H₃)₃), 18.5 (1 C, -Si**C**(CH₃)₃), -5.2 (1 C, -Si**C**H₃), -5.3 (1 C, -Si**C**H₃).

HRMS (ESI-TOF): *m/z:* $[M+Na]^+$ calcd for $C_{42}H_{51}Cl_2NNaO_6Si^+$: 786.2755, found 786.2760.

Data for the cyclic product 6f:

HPLC (YMC-SB, *n*-hexane/IPA = 4/1, flow = 1.0 mL min^{-1}, T_{Column} = 10 °C, *l* = 240 nm) t_r*(trans):* 10.93 min (major), 20.15 min (minor); t_r*(cis):* 15.81 min (major), 36.83 min (minor).

$\boldsymbol{\alpha_D^{20}}$(c = 1, $CHCl_3$): 1.4 (mixture of diastereomers).

HRMS (ESI-TOF): *m/z:* $[M+NH_4]^+$ calcd for $C_{30}H_{37}Cl_2NO_3^+$: 586.1813, found 586.1814.

NMR data for the cis diastereomer:

^{1}H-NMR (500 MHz, $CDCl_3$, 298 K) *δ* / ppm = 7.31 (d, *J* = 8.4 Hz, 1 H, Ar-**H**), 7.24-7.23 (m, 3 H, Ar-**H**), 7.14 (d, *J* = 2.0 Hz, 1 H, Ar-**H**), 7.06-7.05 (m, 1 H, Ar-**H**), 6.85 (dd, J_1 = 2.0 Hz, J_2 = 8.4 Hz, 1 H, Ar-**H**), 6.57 (s, 2 H, Ar-**H**), 5.88 (d, *J* = 15.1 Hz, 1 H, -C$\mathbf{H_2}$), 5.23 (d, *J* = 15.1 Hz, 1 H, -C$\mathbf{H_2}$), 5.17 (s, 1 H, -O**H**), 4.91 (d, *J* = 3.3 Hz, 1 H, -C**H**), 4.27 (d, *J* = 3.3 Hz, 1 H, -C**H**), 1.32 (s, 18 H, -C$\mathbf{H_3}$).

^{13}C-NMR (125 MHz, $CDCl_3$, 298 K) *δ* / ppm = 171.4 (1 C, -**C**OOR), 153.4 (1 C, $\mathbf{C_{Ar}}$), 140.6 (1 C, $\mathbf{C_{Ar}}$), 137.0 (1 C, $\mathbf{C_{Ar}}$), 135.5 (2 C, $\mathbf{C_{Ar}}$), 132.8 (1 C, $\mathbf{C_{Ar}}$), 132.3 (1 C, $\mathbf{C_{Ar}}$), 132.2 (1 C, $\mathbf{C_{Ar}}$), 131.8 (1 C, $\mathbf{C_{Ar}}$), 131.6 (1 C, $\mathbf{C_{Ar}}$), 130.3 (1 C, $\mathbf{C_{Ar}}$), 130.1 (1 C, $\mathbf{C_{Ar}}$), 129.5 (1 C, $\mathbf{C_{Ar}}$), 129.2 (1 C, $\mathbf{C_{Ar}}$), 128.2 (1 C, $\mathbf{C_{Ar}}$), 127.0 (2 C, $\mathbf{C_{Ar}}$), 126.8 (1 C, $\mathbf{C_{Ar}}$), 70.7 (1 C, -**C**H_2), 54.8 (1 C, -**C**H), 49.4 (1 C, -**C**H), 34.4 (2 C, -**C**$(CH_3)_3$), 30.3 (6 C, -C(**C**$H_3)_3$).

NMR data for the trans diastereomer:

^{1}H-NMR (500 MHz, $CDCl_3$, 298 K) *δ* / ppm = 7.33 (d, *J* = 8.3 Hz, 1 H, Ar-**H**), 7.29-7.22 (m, 3 H, Ar-**H**), 7.17 (d, *J* = 2.0 Hz, 1 H, Ar-**H**), 7.09 (d, *J* = 7.7 Hz, 1 H, Ar-**H**), 7.06 (dd, J_1 = 2.0 Hz, J_2 = 8.3 Hz, 1 H, Ar-**H**), 6.42 (s, 2 H, Ar-**H**), 5.95 (d, *J* = 13.8 Hz, 1 H, -C$\mathbf{H_2}$), 5.07 (d, *J* = 13.8 Hz, 1 H, -C$\mathbf{H_2}$), 5.04 (s, 1 H, -O**H**), 4.50 (d, *J* = 11.2 Hz, 1 H, -C**H**), 4.42 (d, *J* = 11.2 Hz, 1 H, Ar-**H**), 1.24 (s, 18 H, -C$\mathbf{H_3}$).

^{13}C-NMR (125 MHz, $CDCl_3$, 298 K) *δ* / ppm = 171.5 (1 C, -**C**OOR), 152.6 (1 C, $\mathbf{C_{Ar}}$), 140.4 (1 C, $\mathbf{C_{Ar}}$), 138.1 (1 C, $\mathbf{C_{Ar}}$), 136.0 (2 C, $\mathbf{C_{Ar}}$), 134.0 (1 C, $\mathbf{C_{Ar}}$), 133.1 (1 C, $\mathbf{C_{Ar}}$), 132.5 (1 C, $\mathbf{C_{Ar}}$), 132.0 (2 C, $\mathbf{C_{Ar}}$), 131.5 (1 C, $\mathbf{C_{Ar}}$), 130.0 (1 C, $\mathbf{C_{Ar}}$), 129.8 (1 C, $\mathbf{C_{Ar}}$), 129.6 (1 C, $\mathbf{C_{Ar}}$), 129.3 (1 C, $\mathbf{C_{Ar}}$), 127.1 (1 C, $\mathbf{C_{Ar}}$), 124.9 (2 C, $\mathbf{C_{Ar}}$), 70.4 (1 C, -**C**H_2), 54.6 (1 C, -**C**H), 54.3 (1 C, -**C**H), 34.2 (2 C, -**C**$(CH_3)_3$), 30.2 (6 C, -C(**C**$H_3)_3$).

4-Bromophenyl benzo[c]oxepinone derivative (6g)

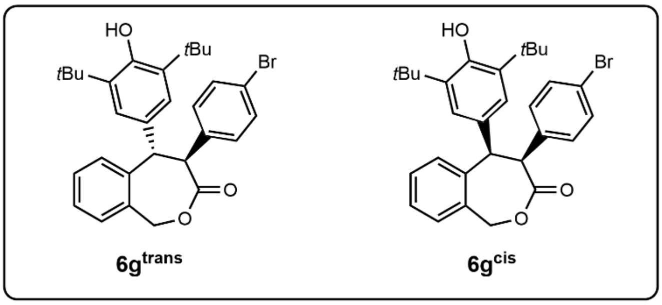

7-membered ring **6g** was synthesized according to general procedure **F** and obtained as a colorless residue in a yield of 59% (72% for the alkylation step) as a mixture of diastereomers (*dr* 55:45; er_{trans} 93:7; er_{cis} 85:15).

Data for the alkylation product (mixture of diastereomers) 5g:

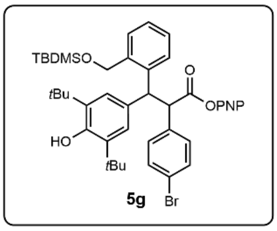

^{1}H-NMR (500 MHz, $CDCl_3$, 298 K) *δ* / ppm = *Signals for the unlike diastereomer:* 8.10 (d, *J* = 9.1 Hz, 2 H, Ar-**H**), 7.40-7.29 (m, 6 H, Ar-**H**), 7.22 (s, 2 H, Ar-**H**), 7.18-7.06 (m, 2 H, Ar-**H**), 6.60 (d, *J* = 9.1 Hz, 2 H, Ar-**H**), 5.17 (s, 1 H, -O**H**), 4.99 (d, *J* = 12.2 Hz, 1 H, -C**H₂**), 4.81 (d, *J* = 13.0 Hz, 1 H, -C**H**), 4.72 (d, *J* = 12.2 Hz, 1 H, -C**H₂**), 4.59 (d, *J* = 13.0 Hz, 1 H, -C**H**), 1.39 (s, 18 H, -C**H₃**), 0.96 (s, 9 H, -SiC(C**H₃**)₃), 0.12 (s, 3 H, -SiC**H₃**), 0.09 (s, 3 H, -SiC**H₃**). *Selected signals for the like diastereomer:* 8.13 (d, *J* = 9.1 Hz, 2 H, Ar-**H**), 7.71 (d, *J* = 7.7 Hz, 1 H, Ar-**H**), 7.53 (d, *J* = 7.6 Hz, 1 H, Ar-**H**), 6.78 (d, *J* = 9.1 Hz, 2 H, Ar-**H**), 6.55 (s, 2 H, Ar-**H**), 4.97 (s, 1 H, -O**H**), 4,87 (d, *J* = 13.5 Hz, 1 H, -C**H**), 4.60 (d, *J* = 11.8 Hz, 1 H, -C**H₂**), 4.58 (d, *J* = 13.5 Hz, 1 H, -C**H**), 4.43 (d, *J* = 11.8 Hz, 1 H, -C**H₂**), 1.24 (s, 18 H, -C**H₃**), 0.85 (s, 9 H, -SiC(C**H₃**)₃), -0.06 (s, 3 H, -SiC**H₃**), -0.07 (s, 3 H, -SiC**H₃**).

^{13}C-NMR (125 MHz, $CDCl_3$, 298 K) *δ* / ppm = *Signals for the unlike diastereomer:* 170.8 (1 C, -**C**OOR), 155.3 (1 C, $\mathbf{C_{Ar}}$), 153.0 (1 C, $\mathbf{C_{Ar}}$), 145.6 (1 C, $\mathbf{C_{Ar}}$), 139.0 (1 C, $\mathbf{C_{Ar}}$), 137.8 (1 C, $\mathbf{C_{Ar}}$), 136. 1 (2 C, $\mathbf{C_{Ar}}$), 135.1 (1 C, $\mathbf{C_{Ar}}$), 131.9 (2 C, $\mathbf{C_{Ar}}$), 130.5 (2 C, $\mathbf{C_{Ar}}$), 127.7 (1 C, $\mathbf{C_{Ar}}$), 127.2 (1 C, $\mathbf{C_{Ar}}$), 126.7 (1 C, $\mathbf{C_{Ar}}$), 126.3 (1 C, $\mathbf{C_{Ar}}$), 125.5 (2 C, $\mathbf{C_{Ar}}$), 125.2 (1 C, $\mathbf{C_{Ar}}$), 125.1 (2 C, $\mathbf{C_{Ar}}$), 122.5 (2 C, $\mathbf{C_{Ar}}$), 122.0 (1 C, $\mathbf{C_{Ar}}$), 63.3 (1 C, -**C**H₂), 57.2 (1 C, -**C**H), 48.4 (1 C, -**C**H), 34.5 (2 C, -**C**(CH₃)₃), 30.4 (6 C, -C(**C**H₃)₃), 26.1 (3 C, -SiC(**C**H₃)₃), 18.6 (1 C, -Si**C**(CH₃)₃), -5.1 (1 C, -Si**C**H₃), -5.2 (1 C, -Si**C**H₃). *Selected signals for the like diastereomer:* 170.4 (1 C, -**C**OOR), 152.5 (1 C, $\mathbf{C_{Ar}}$), 139.7 (1 C, $\mathbf{C_{Ar}}$), 138.8 (1 C, $\mathbf{C_{Ar}}$), 135.8 (1 C, $\mathbf{C_{Ar}}$), 135.6 (2 C, $\mathbf{C_{Ar}}$), 131.7 (2 C, $\mathbf{C_{Ar}}$), 131.4 (2 C, $\mathbf{C_{Ar}}$), 127.5 (1 C, $\mathbf{C_{Ar}}$), 127.2 (1 C, $\mathbf{C_{Ar}}$), 127.0 (1 C, $\mathbf{C_{Ar}}$), 125.3 (2 C, $\mathbf{C_{Ar}}$), 62.8 (1 C, -**C**H₂), 57.6 (1 C, -**C**H), 50.9 (1 C, -**C**H), 34.2 (2 C, -**C**(CH₃)₃), 30.2 (6 C, -C(**C**H₃)₃), 26.0 (3 C, -SiC(**C**H₃)₃), 18.5 (1 C, -Si**C**(CH₃)₃), -5.2 (1 C, -Si**C**H₃), -5.4 (1 C, -Si**C**H₃).

HRMS (ESI-TOF): *m/z:* $[M+Na]^+$ calcd for $C_{42}H_{52}BrNNaO_6Si^+$: 796.2639, found 796.2641.

Data for the cyclic product 6g:

HPLC (YMC-SB, *n*-hexane/IPA = 10/1, flow = 1.0 mL min^{-1}, T_{Column} = 10 °C, *l* = 240 nm) $t_r(trans)$: 16.20 min (major), 33.50 min (minor); $t_r(cis)$: 15.72 min (major), 42.18 min (minor).

α_D^{20}(c = 1, $CHCl_3$): -33.5 (mixture of diastereomers).

HRMS (ESI-TOF): *m/z:* $[M+NH_4]^+$ calcd for $C_{30}H_{37}BrNO_3^+$: 538.1952, found 538.1951.

NMR data for the cis diastereomer:

^{1}H-NMR (500 MHz, $CDCl_3$, 298 K) δ / ppm = 7.36 (d, *J* = 8.5 Hz, 2 H, Ar-**H**), 7.24-.23 (m, 3 H, Ar-**H**), 7.07-7.05 (m, 1 H, Ar-**H**), 6.88 (d, *J* = 8.5 Hz, 2 H, Ar-**H**), 6.55 (s, 2 H, Ar-**H**), 5.89 (d, *J* = 15.1 Hz, 1 H, -C**H₂**), 5.21 (d, *J* = 15.1 Hz, 1 H, -C**H₂**), 5.14 (s, 1 H, -O**H**), 4.91 (d, *J* = 3.4 Hz, 1 H, -C**H**), 4.27 (d, *J* = 3.4 Hz, 1 H, -C**H**), 1.32 (s, 18 H, -C**H₃**).

^{13}C-NMR (125 MHz, $CDCl_3$, 298 K) δ / ppm = 171.8 (1 C, -**C**OOR), 153.3 (1 C, $\mathbf{C_{Ar}}$), 140.9 (1 C, $\mathbf{C_{Ar}}$), 135.9 (1 C, $\mathbf{C_{Ar}}$), 135.3 (2 C, $\mathbf{C_{Ar}}$), 132.6 (2 C, $\mathbf{C_{Ar}}$), 132.4 (1 C, $\mathbf{C_{Ar}}$), 132.4 (1 C, $\mathbf{C_{Ar}}$), 130.8 (2 C, $\mathbf{C_{Ar}}$), 130.6 (1 C, $\mathbf{C_{Ar}}$), 129.1 (1 C, $\mathbf{C_{Ar}}$), 128.2 (1 C, $\mathbf{C_{Ar}}$), 127.1 (2 C, $\mathbf{C_{Ar}}$), 126.7 (1 C, $\mathbf{C_{Ar}}$), 121.7 (1 C, $\mathbf{C_{Ar}}$), 70.7 (1 C, -**C**H_2), 55.0 (1 C, -**C**H), 49.7 (1 C, -**C**H), 34.4 (2 C, -**C**$(CH_3)_3$), 30.3 (6 C, -C(**C**$H_3)_3$).

NMR data for the trans diastereomer:

^{1}H-NMR (500 MHz, $CDCl_3$, 298 K) δ / ppm = 7.37 (d, *J* = 8.5 Hz, 2 H, Ar-**H**), 7.28-7.27 (m, 1 H, Ar-**H**), 7.24-7.21 (m, 2 H, Ar-**H**), 7.10-7-08 (m, 1 H, Ar-**H**), 7.03 (d, *J* = 8.5 Hz, 2 H, Ar-**H**), 6.42 (s, 2 H, Ar-**H**), 5.91 (d, *J* = 13.7 Hz, 1 H, -C**H₂**), 5.05 (d, *J* = 13.7 Hz, 1 H, -C**H₂**), 5.01 (s, 1 H, -O**H**), 4.48 (d, *J* = 10.9 Hz, 1 H, -C**H**), 4.45 (d, *J* = 10.9 Hz, 1 H, -C**H**), 1.23 (s, 18 H, -C**H₃**).

^{13}C-NMR (125 MHz, $CDCl_3$, 298 K) δ / ppm = 172.1 (1 C, -**C**OOR), 152.5 (1 C, $\mathbf{C_{Ar}}$), 140.7 (1 C, $\mathbf{C_{Ar}}$), 136.9 (1 C, $\mathbf{C_{Ar}}$), 135.8 (2 C, $\mathbf{C_{Ar}}$), 134.3 (1 C, $\mathbf{C_{Ar}}$), 133.3 (1 C, $\mathbf{C_{Ar}}$), 132.5 (1 C, $\mathbf{C_{Ar}}$), 131.6 (2 C, $\mathbf{C_{Ar}}$), 131.3 (2 C, $\mathbf{C_{Ar}}$), 129.8 (1 C, $\mathbf{C_{Ar}}$), 129.6 (1 C, $\mathbf{C_{Ar}}$), 127.0 (1 C, $\mathbf{C_{Ar}}$), 124.9 (2 C, $\mathbf{C_{Ar}}$), 121.6 (1 C, $\mathbf{C_{Ar}}$), 70.4 (1 C, -**C**H_2), 55.1 (1 C, -**C**H), 54.5 (1 C, **C**H), 34.3 (2 C, -**C**$(CH_3)_3$), 30.2 (6 C, -**C**H_3).

3-Iodophenyl benzo[c]oxepinone derivative 6h

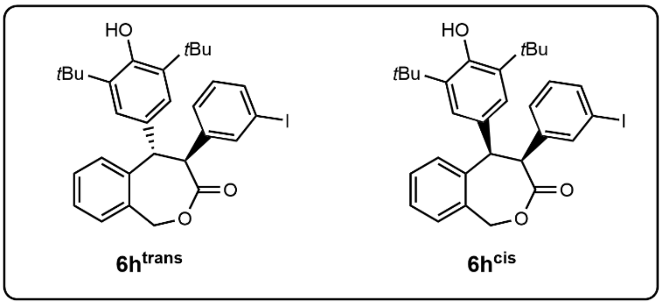

7-membered ring **6h** was synthesized according to general procedure **F** and obtained as a colorless residue in a yield of 60% (64% for the alkylation step) as a mixture of diastereomers (*dr* 50:50; er_{trans} 91:9; er_{cis} 83:17).

Data for the alkylation product (mixture of diastereomers) 5h:

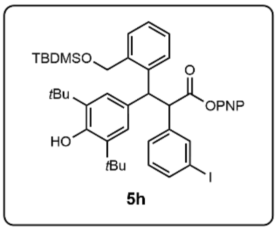

^{1}H-NMR (500 MHz, $CDCl_3$, 298 K) *δ* / ppm = *Signals for the unlike diastereomer:* 8.11 (d, *J* = 9.1 Hz, 2 H, Ar-**H**), 7.84 (s, 1 H, Ar-**H**), 7.54-7.33 (m, 4 H, Ar-**H**), 7.24-7.19 (m, 1 H, Ar-**H**), 7.23 (s, 2 H, Ar-**H**), 7.14 (t, *J* = 7.6 Hz, 1 H, Ar-**H**), 6.96 (t, *J* = 7.9 Hz, 1 H, Ar-**H**), 6.62 (d, *J* = 9.1 Hz, 2 H, Ar-**H**), 5.18 (s, 1 H, -O**H**), 4.92 (d, *J* = 12.2 Hz, 1 H, -C**H₂**), 4.77 (d, *J* = 13.1 Hz, 1 H, -C**H**), 4.69 (d, *J* = 12.2 Hz, 1 H, -C**H₂**), 4.64 (d, *J* = 13.1 Hz, 1 H, -C**H**), 1.40 (s, 18 H, -C**H₃**), 0.96 (s, 9 H, -SiC(C**H₃**)₃), 0.12 (s, 3 H, -SiC**H₃**), 0.10 (s, 3 H, -SiC(C**H₃**)₃). *Selected signals for the like diastereomer:* 8.13 (d, *J* = 9.1 Hz, 2 H, Ar-**H**), 7.71 (d, *J* = 7.6 Hz, 1 H, Ar-**H**), 7.58 (d, *J* = 7.9 Hz, 1 H, Ar-**H**), 7.01 (t, *J* = 7.8 Hz, 1 H, Ar-**H**), 6.80 (d, *J* = 9.1 Hz, 2 H, Ar-**H**), 6.60 (s, 2 H, Ar-**H**), 4.99 (1 H, -O**H**), 4.89 (d, *J* = 13.0 Hz, 1 H, -C**H**), 4.63 (d, *J* = 11.9 Hz, 1 H, -C**H₂**), 4.59 (d, *J* = 13.9 Hz, 1 H, -C**H**), 4.41 (d, *J* = 11.9 Hz, 1 H, -C**H₂**), 1.27 (s, 18 H, -C**H₃**), 0.87 (s, 9 H, -SiC(C**H₃**)₃), -0.02 (s, 3 H, -SiC**H₃**), -0.05 (s, 3 H, -SiC**H₃**).

^{13}C-NMR (125 MHz, $CDCl_3$, 298 K) *δ* / ppm = *Signals for the unlike diastereomer:* 170.7 (1 C, -**C**OOR), 155.3 (1 C, C_{Ar}), 153.0 (1 C, C_{Ar}), 145.5 (1 C, C_{Ar}), 139.0 (1 C, C_{Ar}), 138.3 (1 C, C_{Ar}), 137.7 (1 C, C_{Ar}), 137.4 (1 C, C_{Ar}), 137.0 (1 C, C_{Ar}), 136.1 (2 C, C_{Ar}), 131.4 (1 C, C_{Ar}), 130.5 (1 C, C_{Ar}), 128.0 (1 C, C_{Ar}), 127.3 (1 C, C_{Ar}), 127.1 (1 C, C_{Ar}), 126.7 (1 C, C_{Ar}), 126.3 (1 C, C_{Ar}), 125.5 (2 C, C_{Ar}), 125.1 (2 C, C_{Ar}), 122.5 (2 C, C_{Ar}), 94.4 (1 C, C_{Ar}), 63.0 (1 C, -**C**H₂), 57.2 (1 C, -**C**H), 48.7 (1 C, -**C**H), 34.5 (2 C, -**C**(CH₃)₃), 30.4 (6 C, -C(**C**H₃)₃), 26.2 (3 C, -SiC(**C**H₃)₃), 18.6 (1 C, -Si**C**(CH₃)₃), -5.1 (1 C, -Si**C**H₃), -5.1 (1 C, -Si**C**H₃). *Selected signals for the like diastereomer:* 170.2 (1 C, -**C**OOR), 155.3 (1 C, C_{Ar}), 152.5 (1 C, C_{Ar}), 139.7 (1 C, C_{Ar}), 138.9 (1 C, C_{Ar}), 138.8 (1 C, C_{Ar}), 138.1 (1 C, C_{Ar}), 136.7 (1 C, C_{Ar}), 135.7 (2 C, C_{Ar}), 130.3 (1 C, C_{Ar}), 129.6 (1 C, C_{Ar}), 127.7 (1 C, C_{Ar}), 127.6 (1 C, C_{Ar}), 125.3 (2 C, C_{Ar}), 125.2 (2 C, C_{Ar}), 94.3 (1 C, C_{Ar}), 62.9 (1 C, -**C**H₂), 57.6 (1 C, -**C**H), 50.9 (1 C, -**C**H), 34.2 (2 C, -**C**(CH₃)₃), 30.3 (6 C, -C(**C**H₃)₃), 26.1 (3 C, -SiC(**C**H₃)₃), 18.5 (1 C, -Si**C**(CH₃)₃), -5.2 (1 C, -Si**C**H₃), -5.3 (1 C, -Si**C**H₃).

HRMS (ESI-TOF): *m/z:* $[M+Na]^+$ calcd for $C_{42}H_{52}INNaO_6Si^+$: 844.2501, found 844.2502.

Data for the cyclic product 6h:

HPLC (YMC-SB, *n*-hexane/IPA = 10/1, flow = 1.0 mL min^{-1}, T_{Column} = 10 °C, *l* = 240 nm) t_r*(trans):* 13.78 min (major), 47.60 min (minor); t_r*(cis):* 16.98 min (major), 29.15 min (minor).

α_D^{20}(c = 1, $CHCl_3$): -26.7 (mixture of diastereomers).

HRMS (ESI-TOF): *m/z:* $[M+NH_4]^+$ calcd for $C_{30}H_{37}INO_3^+$: 586.1813, found 586.1814.

NMR data for the cis diastereomer:

^{1}H-NMR (500 MHz, $CDCl_3$, 298 K) δ / ppm = 7.62 (dt, J_1 = 1.6 Hz, J_2 = 7.3 Hz, 1 H, Ar-**H**), 7.46-7.45 (m, 1 H, Ar-**H**), 7.23-7.22 (m, 3 H, Ar-**H**), 7.06-7.04 (m, 1 H, Ar-**H**), 6.98-6.93 (m, 2 H, Ar-**H**), 6.58 (s, 2 H, Ar-**H**), 5.88 (d, *J* = 15.1 Hz, 1 H, -C**H₂**), 5.22 (d, *J* = 15.1 Hz, 1 H, -C**H**), 5.15 (s, 1 H, -O**H**), 4.87 (d, *J* = 3.3 Hz, 1 H, -C**H**), 4.30 (d, *J* = 3.3 Hz, 1 H, -C**H**), 1.33 (s, 18 H, -C**H₃**).

^{13}C-NMR (125 MHz, $CDCl_3$, 298 K) δ / ppm = 171.6 (1 C, -**C**OOR), 153.3 (1 C, $\mathbf{C_{Ar}}$), 140.9 (1 C, $\mathbf{C_{Ar}}$), 139.3 (1 C, $\mathbf{C_{Ar}}$), 139.2 (1 C, $\mathbf{C_{Ar}}$), 136.7 (1 C, $\mathbf{C_{Ar}}$), 135.4 (2 C, $\mathbf{C_{Ar}}$), 132.3 (1 C, $\mathbf{C_{Ar}}$), 132.3 (1 C, $\mathbf{C_{Ar}}$), 130.6 (1 C, $\mathbf{C_{Ar}}$), 130.4 (1 C, $\mathbf{C_{Ar}}$), 129.4 (1 C, $\mathbf{C_{Ar}}$), 129.1 (1 C, $\mathbf{C_{Ar}}$), 128.1 (1 C, $\mathbf{C_{Ar}}$), 127.0 (2 C, $\mathbf{C_{Ar}}$), 126.7 (1 C, $\mathbf{C_{Ar}}$), 93.5 (1 C, $\mathbf{C_{Ar}}$-I), 70.7 (1 C, -**C**H_2), 54.9 (1 C, -**C**H), 49.7 (1 C, -**C**H), 34.4 (2 C, -**C**$(CH_3)_3$), 30.4 (6 C, -C(**C**$H_3)_3$).

NMR data for the trans diastereomer:

^{1}H-NMR (500 MHz, $CDCl_3$, 298 K) δ / ppm = 7.56-7.54 (m, 1 H, Ar-**H**), 7.39 (t, *J* = 1.6 Hz, 1 H, Ar-**H**), 7.29-7.22 (m, 4 H, Ar-**H**), 7.11-7.09 (m, 1 H, Ar-**H**), 7.02 (t, *J* = 7.8 Hz, 1 H, Ar-**H**), 6.44 (s, 2 H, Ar-**H**), 5.92 (d, *J* = 13.7 Hz, 1 H, -C**H₂**), 5.06 (d, *J* = 13.7 Hz, 1 H, -C**H**), 5.02 (s, 1 H, -O**H**), 4.46 (app. s, 2 H, 2x -C**H**), 1.25 (s, 18 H, -C**H₃**).

^{13}C-NMR (125 MHz, $CDCl_3$, 298 K) δ / ppm = 171.9 (1 C, -**C**OOR), 152.6 (1 C, $\mathbf{C_{Ar}}$), 140.7 (1 C, $\mathbf{C_{Ar}}$), 140.2 (1 C, $\mathbf{C_{Ar}}$), 138.9 (1 C, $\mathbf{C_{Ar}}$), 136.4 (1 C, $\mathbf{C_{Ar}}$), 135.9 (2 C, $\mathbf{C_{Ar}}$), 134.1 (1 C, $\mathbf{C_{Ar}}$), 133.3 (1 C, $\mathbf{C_{Ar}}$), 132.5 (1 C, $\mathbf{C_{Ar}}$), 130.0 (1 C, $\mathbf{C_{Ar}}$), 129.8 (1 C, $\mathbf{C_{Ar}}$), 129.6 (1 C, $\mathbf{C_{Ar}}$), 129.1 (1 C, $\mathbf{C_{Ar}}$), 127.0 (1 C, $\mathbf{C_{Ar}}$), 124.9 (2 C, $\mathbf{C_{Ar}}$), 93.8 (1 C, $\mathbf{C_{Ar}}$-I), 70.4 (1 C, -**C**H_2), 55.1 (1 C, -**C**H), 54.4 (1 C, -**C**H), 34.3 (2 C, -**C**$(CH_3)_3$), 30.3 (6 C, -C(**C**$H_3)_3$).

4-Methoxyphenyl benzo[c]oxepinone derivative (6i)

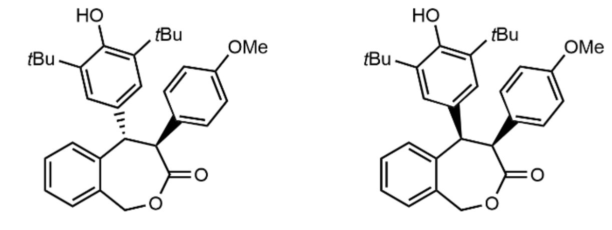

6i^{trans} **6i**^{cis}

7-membered ring **6i** was synthesized according to general procedure **F** and obtained as a colorless residue in a yield of 30% (50% for the alkylation step) as a mixture of diastereomers (*dr* 40:60; er_{trans} 96:4; er_{cis} 94:6).

Data for the alkylation product (mixture of diastereomers) 5i:

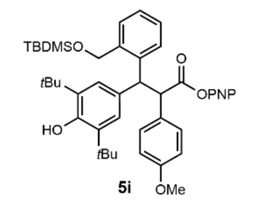

5i

^{1}H-NMR (500 MHz, $CDCl_3$, 298 K) δ / ppm = *Signals for the unlike diastereomer:* 8.10 (d, J = 9.2 Hz, 2 H, Ar-**H**), 7.39 (d, J = 8.7 Hz, 2 H, Ar-**H**), 7.34-7.32 (m, 2 H, Ar-**H**), 7.24 (s, 2 H, Ar-**H**), 7.20-7.17 (m, 1 H, Ar-**H**), 7.14-7.10 (m, 1 H, Ar-**H**), 6.77 (d, J = 8.7 Hz, 2 H, Ar-**H**), 6.61 (d, J = 9.2 Hz, 2 H, Ar-**H**), 5.17 (s, 1 H, -O**H**), 4.93 (d, J = 12.2 Hz, 1 H, -C$\mathbf{H_2}$), 4.79 (d, J = 13.1 Hz, 1 H, -C**H**), 4.71 (d, J = 12.2 Hz, 1 H, -C$\mathbf{H_2}$), 4.65 (d, J = 13.1 Hz, 1 H, -C**H**), 3.73 (s, 3 H, -OC$\mathbf{H_3}$), 1.40 (s, 18 H, -C$\mathbf{H_3}$), 0.96 (s, 9 H, -SiC(C$\mathbf{H_3}$)$_3$), 0.11 (s, 3 H, -SiC$\mathbf{H_3}$), 0.08 (s, 3 H, -SiC$\mathbf{H_3}$). *Selected signals for the like diastereomer:* 8.13 (d, J = 9.2 Hz, 2 H, Ar-**H**), 7.72 (d, J = 7.4 Hz, 1 H, Ar-**H**), 7.55 (d, J = 7.5 Hz, 1 H, Ar-**H**), 7.41 (d, J = 8.7 Hz, 2 H, Ar-**H**), 6.80 (d, J = 8.7 Hz, 2 H, Ar-**H**), 6.78 (d, J = 9.2 Hz, 2 H, Ar-**H**), 6.57 (s, 2 H, Ar-**H**), 4.95 (s, 1 H, -O**H**), 4.90 (d, J = 13.8 Hz, 1 H, -C**H**), 4.61 (d, J = 13.8 Hz, 1 H, -C**H**), 4.59 (d, J = 11.7 Hz, 1 H, -C$\mathbf{H_2}$), 4.41 (d, J = 11.7 Hz, 1 H, -C$\mathbf{H_2}$), 3.78 (s, 3 H, -OC$\mathbf{H_3}$), 1.24 (s, 18 H, -C$\mathbf{H_3}$), 0.85 (s, 9 H, -SiC(C$\mathbf{H_3}$)$_3$), -0.06 (s, 3 H, -SiC$\mathbf{H_3}$), -0.07 (s, 3 H, -SiC$\mathbf{H_3}$).

^{13}C-NMR (125 MHz, $CDCl_3$, 298 K) δ / ppm = *Signals for the unlike diastereomer:* 171.4 (1 C, -**C**OOR), 159.1 (1 C, $\mathbf{C_{Ar}}$), 155.5 (1 C, $\mathbf{C_{Ar}}$), 152.8 (1 C, $\mathbf{C_{Ar}}$), 145.4 (1 C, $\mathbf{C_{Ar}}$), 139.0 (1 C, $\mathbf{C_{Ar}}$), 138.0 (1 C, $\mathbf{C_{Ar}}$), 136.0 (2 C, $\mathbf{C_{Ar}}$), 131.9 (1 C, $\mathbf{C_{Ar}}$), 129.8 (2 C, $\mathbf{C_{Ar}}$), 127.9 (1 C, $\mathbf{C_{Ar}}$), 127.0 (1 C, $\mathbf{C_{Ar}}$), 127.0 (1 C, $\mathbf{C_{Ar}}$), 126.4 (1 C, $\mathbf{C_{Ar}}$), 126.4 (1 C, $\mathbf{C_{Ar}}$), 125.5 (2 C, $\mathbf{C_{Ar}}$), 125.0 (2 C, $\mathbf{C_{Ar}}$), 122.5 (2 C, $\mathbf{C_{Ar}}$), 114.1 (2 C, $\mathbf{C_{Ar}}$), 63.0 (1 C, -**C**H_2), 56.9 (1 C, -C**H**), 55.2 (1 C, -O**C**H_3), 48.6 (1 C, -**C**H), 34.4 (2 C, -C(**C**H_3)$_3$), 30.4 (6 C, -C(**C**H_3)$_3$), 26.1 (3 C, -SiC(**C**H_3)$_3$), 18.5 (1 C, -Si**C**(CH_3)$_3$), -5.2 (1 C, -Si**C**H_3), -5.2 (1 C, -Si**C**H_3)$_3$). *Selected signals for the like diastereomer:* 171.0 (1 C, -**C**OOR), 159.3 (1 C, $\mathbf{C_{Ar}}$), 152.2 (1 C, $\mathbf{C_{Ar}}$), 140.8 (1 C, $\mathbf{C_{Ar}}$), 139.6 (1 C, $\mathbf{C_{Ar}}$), 139.2 (1 C, $\mathbf{C_{Ar}}$), 139.0 (1 C, $\mathbf{C_{Ar}}$), 135.2 (2 C, $\mathbf{C_{Ar}}$), 129.9 (2 C, $\mathbf{C_{Ar}}$), 125.4 (2 C, $\mathbf{C_{Ar}}$), 125.1 (2 C, $\mathbf{C_{Ar}}$), 122.5 (2 C, $\mathbf{C_{Ar}}$), 114.0 (2 C, $\mathbf{C_{Ar}}$), 62.7 (1 C, -**C**H_2), 57.3 (1 C, -**C**H), 55.4 (1 C, -O**C**H_3), 50.8 (1 C, -**C**H), 34.2 (2 C, -**C**(CH_3)$_3$), 30.2 (6 C, -C(**C**H_3)$_3$), 26.0 (3 C, -SiC(**C**H_3)$_3$), 18.5 (1 C, -Si**C**(CH_3)$_3$), -5.3 (1 C, -Si**C**H_3), -5.4 (1 C, -Si**C**H_3).

HRMS (ESI-TOF): *m/z:* $[M+Na]^+$ calcd for $C_{43}H_{55}NNaO_7Si^+$: 748.3640, found 748.3644.

Data for the cyclic product 6i:

HPLC (YMC-SB, *n*-hexane/IPA = 4/1, flow = 1.0 mL min^{-1}, T_{Column} = 10 °C, *l* = 240 nm) t_r*(trans):* 12.78 min (major), 32.13 min (minor); t_r*(cis):* 11.59 min (major), 20.62 min (minor).

α_D^{20}(c = 1, $CHCl_3$): -40.8 (mixture of diastereomers).

HRMS (ESI-TOF): *m/z:* $[M+NH_4]^+$ calcd for $C_{31}H_{40}NO_4^+$: 490.2952, found 490.2951.

NMR data for the cis diastereomer:

^{1}H-NMR (500 MHz, $CDCl_3$, 298 K) *δ* / ppm = 7.23-7.22 (m, 3 H, Ar-**H**), 7.07-7.05 (m, 1 H, Ar-**H**), 6.92 (d, *J* = 8.8 Hz, 2 H, Ar-**H**), 6.77 (d, *J* = 8.8 Hz, 2 H, Ar-**H**), 6.58 (s, 2 H, Ar-**H**), 5.89 (d, *J* = 15.1 Hz, 1 H, -C**H$_2$**), 5.20 (d, *J* = 15.1 Hz, 1 H, -C**H$_2$**), 5.12 (s, 1 H, -O**H**), 4.91 (d, *J* = 3.4 Hz, 1 H, -C**H**), 4.29 (d, *J* = 3.4 Hz, 1 H, -C**H**), 3.79 (s, 3 H, -OC**H$_3$**), 1.32 (s, 18 H, -C**H$_3$**).

^{13}C-NMR (125 MHz, $CDCl_3$, 298 K) *δ* / ppm = 172.5 (1 C, -**C**OOR), 159.0 (1 C, **C$_{Ar}$**), 153.2 (1 C, **C$_{Ar}$**), 141.3 (1 C, **C$_{Ar}$**), 135.1 (2 C, **C$_{Ar}$**), 132.6 (1 C, **C$_{Ar}$**), 132.4 (1 C, **C$_{Ar}$**), 131.9 (2 C, **C$_{Ar}$**), 131.1 (1 C, **C$_{Ar}$**), 129.1 (1 C, **C$_{Ar}$**), 129.0 (1 C, **C$_{Ar}$**), 128.1 (1 C, **C$_{Ar}$**), 127.2 (2 C, **C$_{Ar}$**), 126.6 (1 C, **C$_{Ar}$**), 113.1 (2 C, **C$_{Ar}$**), 70.6 (1 C, -**C**H$_2$), 55.4 (1 C, -**C**H), 55.3 (1 C, -O**C**H$_3$), 49.5 (1 C, -**C**H), 34.4 (2 C, -**C**(CH$_3$)$_3$), 30.4 (6 C, -C(**C**H$_3$)$_3$).

NMR data for the trans diastereomer:

^{1}H-NMR (500 MHz, $CDCl_3$, 298 K) *δ* / ppm = 7.27-7.19 (m, 3 H, Ar-**H**), 7.12-7.10 (m, 1 H, Ar-**H**), 7.08 (d, *J* = 8.8 Hz, 2 H, Ar-**H**), 6.80 (d, *J* = 8.8 Hz, 2 H, Ar-**H**), 6.46 (s, 2 H, Ar-**H**), 5.82 (d, *J* = 13.6 Hz, 1 H, -C**H$_2$**), 5.04 (d, *J* = 13.6 Hz, 1 H, -C**H$_2$**), 4.99 (s, 1 H, -O**H**), 4.50 (d, *J* = 10.2 Hz, 1 H, -C**H**), 4.44 (d, *J* = 10.2 Hz, 1 H, -C**H**), 3.77 (s, 3 H, -OC**H$_3$**), 1.23 (18 H, -C**H$_3$**).

^{13}C-NMR (125 MHz, $CDCl_3$, 298 K) *δ* / ppm = 172.8 (1 C, -**C**OOR), 158.9 (1 C, **C$_{Ar}$**), 152.4 (1 C, **C$_{Ar}$**), 141.0 (1 C, **C$_{Ar}$**), 135.6 (2 C, **C$_{Ar}$**), 134.6 (1 C, **C$_{Ar}$**), 133.6 (1 C, **C$_{Ar}$**), 132.4 (1 C, **C$_{Ar}$**), 130.5 (2 C, **C$_{Ar}$**), 129.8 (1 C, **C$_{Ar}$**), 129.6 (1 C, **C$_{Ar}$**), 129.3 (1 C, **C$_{Ar}$**), 126.8 (1 C, **C$_{Ar}$**), 125.0 (2 C, **C$_{Ar}$**), 113.8 (2 C, **C$_{Ar}$**), 70.2 (1 C, -**C**H$_2$), 55.4 (1 C, -O**C**H$_3$), 55.2 (1 C, -**C**H), 54.2 (1 C, -**C**H), 34.2 (2 C, -**C**(CH$_3$)$_3$), 30.2 (6 C, -C(**C**H$_3$)$_3$).

3,4-Dimethoxyphenyl benzo[c]oxepinone derivative (6j)

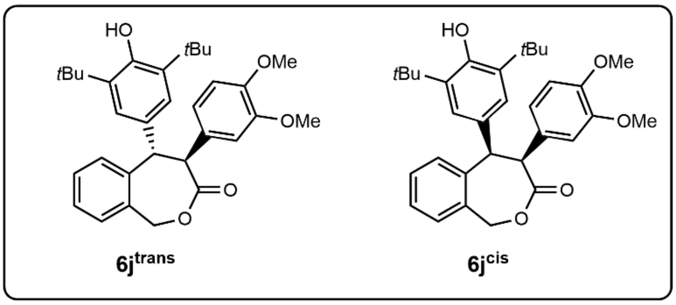

7-membered ring **6j** was synthesized according to general procedure **F** and obtained as a colorless residue in a yield of 46% (47% for the alkylation step) as a mixture of diastereomers (*dr* 75:25; er_{trans} 97:3; er_{cis} 91:9).

Data for the alkylation product (mixture of diastereomers) 5j:

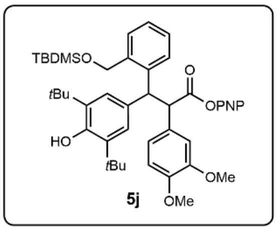

^{1}H-NMR (500 MHz, $CDCl_3$, 298 K) δ / ppm = *Signals for the unlike diastereomer:* 8.11 (d, *J* = 9.2 Hz, 2 H, Ar-**H**), 7.43-7.42 (m, 1 H, Ar-**H**), 7.36-7.32 (m, 1 H, Ar-**H**), 7.24 (s, 2 H, Ar-**H**), 7.19 (t, *J* = 7.8 Hz, 1 H, Ar-**H**), 7.13 (t, *J* = 7.4 Hz, 1 H, Ar-**H**), 7.03 (dd, J_1 = 2.0 Hz, J_2 = 8.4 Hz, 1 H, Ar-**H**), 6.83 (m, 1 H, Ar-**H**), 6.73 (d, *J* = 8.4 Hz, 1 H, Ar-**H**), 6.63 (d, *J* = 9.2 Hz, 2 H, Ar-**H**), 5.17 (s, 1 H, -O**H**), 4.80 (d, *J* = 12.2 Hz, 1 H, -C**H$_2$**), 4.72-4.66 (m, 3 H, -C**H**, -C**H$_2$**), 3.81 (s, 3 H, -OC**H$_3$**), 3.78 (s, 3 H, -OC**H$_3$**), 1.40 (18 H, -C**H$_3$**), 0.94 (9 H, -SiC(C**H$_3$**)$_3$), 0.07 (s, 3 H, -SiC**H$_3$**)$_3$), 0.05 (s, 3 H, -SiC**H$_3$**)$_3$). *Selected signals for the like diastereomer:* 8.14 (d, *J* = 9.2 Hz, 2 H, Ar-**H**), 7.72 (d, *J* = 7.2 Hz, 1 H, Ar-**H**), 7.54 (d, *J* = 7.4 Hz, 1 H, Ar-**H**), 7.41-7.38 (m, 1 H, Ar-**H**), 7.34-7.32 (m, 1 H, Ar-**H**), 6.93 (dd, J_1 = 2.0 Hz, J_2 = 8.3 Hz, 1 H, Ar-**H**), 6.83-6.80 (m, 1 H, Ar-**H**), 6.82 (d, *J* = 9.2 Hz, 2 H, Ar-**H**), 6.57 (s, 2 H, Ar-**H**), 6.34 (d, *J* = 2.0 Hz, 1 H, Ar-**H**), 4.98 (s, 1 H, -O**H**), 4.88 (d, *J* = 13.6 Hz, 1 H, -C**H**), 4.58 (d, *J* = 13.6 Hz, 1 H, -C**H**), 4.54 (d, *J* = 11.7 Hz, 1 H, -C**H$_2$**), 4.39 (d, *J* = 11.7 Hz, 1 H, -C**H$_2$**), 3.86 (s, 3 H, -OC**H$_3$**), 3.64 (s, 3 H, -OC**H$_3$**), 1.23 (s, 18 H, -C**H$_3$**), 0.84 (s, -SiC(C**H$_3$**)$_3$), -0.07 (s, 3 H, -SiC**H$_3$**), -0.08 (s, 3 H, -SiC**H$_3$**).

^{13}C-NMR (125 MHz, $CDCl_3$, 298 K) δ / ppm = *Signals for the unlike diastereomer:* 171.3 (1 C, -**C**OOR), 155.4 (1 C, **C_{Ar}**), 152.8 (1 C, **C_{Ar}**), 148.8 (1 C, **C_{Ar}**), 148.5 (1 C, **C_{Ar}**), 145.4 (1 C, **C_{Ar}**), 139.0 (1 C, **C_{Ar}**), 137.8 (1 C, **C_{Ar}**), 136.0 (2 C, **C_{Ar}**), 131.9 (1 C, **C_{Ar}**), 128.2 (1 C, **C_{Ar}**), 126.8 (1 C, **C_{Ar}**), 126.6 (1 C, **C_{Ar}**), 126.5 (1 C, **C_{Ar}**), 126.4 (1 C, **C_{Ar}**), 125.3 (2 C, **C_{Ar}**), 125.1 (2 C, **C_{Ar}**), 122.5 (2 C, **C_{Ar}**), 120.8 (1 C, **C_{Ar}**), 111.7 (1 C, **C_{Ar}**), 111.0 (1 C, **C_{Ar}**), 62.7 (1 C, -**C**H$_2$), 57.2 (1 C, -**C**H), 55.9 (1 C, -O**C**H$_3$), 55.8 (1 C, -O**C**H$_3$), 48.9 (1 C,-**C**H), 34.5 (2 C, -**C**(CH$_3$)$_3$), 30.4 (6 C, -C(**C**H$_3$)$_3$), 26.1 (3 C, -SiC(**C**H$_3$)$_3$), 18.6 (1 C, -Si**C**(CH$_3$)$_3$), -5.2 (1 C, -Si**C**H$_3$), -5.2 (1 C, -Si**C**H$_3$). *Selected signals for the like diastereomer:* 170.8 (1 C, -**C**OOR), 152.3 (1 C, **C_{Ar}**), 148.7 (1 C, **C_{Ar}**), 148.6 (1 C, **C_{Ar}**), 139.6 (1 C, **C_{Ar}**), 139.1 (1 C, **C_{Ar}**), 135.4 (2 C, **C_{Ar}**), 130.1 (1 C, **C_{Ar}**), 129.0 (1 C, **C_{Ar}**), 127.4 (1 C, **C_{Ar}**), 127.0 (1 C, **C_{Ar}**), 126.9 (1 C, **C_{Ar}**), 125.4 (2 C, **C_{Ar}**), 125.2 (2 C, **C_{Ar}**), 120.0 (1 C, **C_{Ar}**), 112.6 (1 C, **C_{Ar}**), 111.2 (1 C, **C_{Ar}**), 62.8 (1 C,-**C**H$_2$),

57.5 (1 C, -**C**H), 56.0 (1 C, -O**C**H_3), 55.7 (1 C, -O**C**H_3), 51.2 (1 C, -**C**H), 34.2 (2 C, -**C**(CH_3)$_3$), 30.2 (6 C, -C(**C**H_3)$_3$), 26.0 (3 C, -SiC(**C**H_3)$_3$), 18.5 (1 C, -Si**C**(CH_3)$_3$), -5.3 (1 C, -Si**C**H_3), -5.4 (1 C, -Si**C**H_3).

HRMS (ESI-TOF): *m/z:* $[M+NH_4]^+$ calcd for $C_{44}H_{61}N_2O_8Si^+$: 773.4192, found 773.4195.

Data for the cyclic product 6j:

HPLC (YMC-SA, *n*-hexane/IPA = 4/1, flow = 1.0 mL min^{-1}, T_{Column} = 10 °C, *l* = 220 nm) t_r*(trans):* 10.02 min (major), 31.09 min (minor); t_r*(cis):* 8.50 min (major), 22.12 min (minor).

α_D^{20}(c = 1, $CHCl_3$): -6.1 (mixture of diastereomers).

HRMS (ESI-TOF): *m/z*: $[M+NH_4]^+$ calcd for $C_{32}H_{42}NO_5^+$: 520.3057, found 520.3061.

NMR data for the cis diastereomer:

^{1}H-NMR (500 MHz, $CDCl_3$, 298 K) *δ* / ppm = 7.23-7.20 (m, 3 H, Ar-**H**), 7.07-7.06 (m, 1 H, Ar-**H**), 6.75 (d, *J* = 8.3 Hz, 1 H, Ar-**H**), 6.63 (dd, J_1 = 2.1 Hz, J_2 = 8.3 Hz, 1 H, Ar-**H**), 6.60 (s, 2 H, Ar-**H**), 6.43 (d, *J* = 2.1 Hz, 1 H, Ar-**H**), 5.91 (d, *J* = 15.0 Hz, 1 H, -C**H₂**), 5.19 (d, *J* = 15.0 Hz, 1 H, -C**H**), 5.13 (s, 1 H, -O**H**), 4.90 (d, *J* = 3.8 Hz, 1 H, -C**H**), 4.31 (d, *J* = 3.8 Hz, 1 H, -C**H**), 3.86 (s, 3 H, -OC**H₃**), 3.60 (s, 3 H, -OC**H₃**), 1.32 (s, 18 H, -C**H₃**).

^{13}C-NMR (125 MHz, $CDCl_3$, 298 K) *δ* / ppm = 172.5 (1 C, -**C**OOR), 153.1 (1 C, **C_{Ar}**), 148.3 (1 C, **C_{Ar}**), 147.9 (1 C, **C_{Ar}**), 141.2 (1 C, **C_{Ar}**), 135.4 (2 C, **C_{Ar}**), 132.6 (1 C, **C_{Ar}**), 132.4 (1 C, **C_{Ar}**), 131.5 (1 C, **C_{Ar}**), 129.6 (1 C, **C_{Ar}**), 129.0 (1 C, **C_{Ar}**), 128.2 (1 C, **C_{Ar}**), 127.1 (2 C, **C_{Ar}**), 126.5 (1 C, **C_{Ar}**), 122.3 (1 C, **C_{Ar}**), 114.3 (1 C, **C_{Ar}**), 110.3 (1 C, **C_{Ar}**), 70.6 (1 C, -**C**H_2), 56.1 (1 C, -**C**H), 55.6 (1 C, -O**C**H_3), 55.6 (1 C, -O**C**H_3), 49.3 (1 C, -C**H**), 34.4 (2 C, -**C**(CH_3)$_3$), 30.3 (6 C, -C(**C**H_3)$_3$).

NMR data for the trans diastereomer:

^{1}H-NMR (500 MHz, $CDCl_3$, 298 K) *δ* / ppm = 7.27-7.21 (m, 3 H, Ar-**H**), 7.12 (d, *J* = 7.4 Hz, 1 H, Ar-**H**), 6.79-6.75 (m, 2 H, Ar-**H**), 6.59 (d, *J* = 2.0 Hz, 1 H, Ar-**H**), 6.51 (s, 2 H, Ar-**H**), 5.78 (d, *J* = 13.7 Hz, 1 H, -C**H₂**), 5.06 (d, *J* = 13.7 Hz, 1 H, -C**H**), 5.01 (s, 1 H, -O**H**), 4.50 (d, *J* = 10.1 Hz, 1 H, -C**H**), 4.45 (d, *J* = 10.1 Hz, 1 H, -C**H**), 3.84 (s, 3 H, -OC**H₃**), 3.73 (s, 3 H, -OC**H₃**), 1.25 (s, 18 H, -C**H₃**).

^{13}C-NMR (125 MHz, $CDCl_3$, 298 K) *δ* / ppm = 172.6 (1 C, -**C**OOR), 152.3 (1 C, **C_{Ar}**), 148.6 (1 C, **C_{Ar}**), 148.2 (1 C, **C_{Ar}**), 140.8 (1 C, **C_{Ar}**), 135.6 (2 C, **C_{Ar}**),134.4 (1 C, **C_{Ar}**), 133.5 (1 C, **C_{Ar}**), 132.3 (1 C, **C_{Ar}**), 130.0 (1 C, **C_{Ar}**), 129.5 (1 C, **C_{Ar}**), 129.2 (1 C, **C_{Ar}**), 126.8 (1 C, **C_{Ar}**), 124.9 (2 C, **C_{Ar}**), 121.4 (1 C, **C_{Ar}**), 112.6 (1 C, **C_{Ar}**), 110.9 (1 C, **C_{Ar}**), 70.1 (1 C, -**C**H_2), 55.9 (1 C, -O**C**H_3), 55.8 (1 C, -O**C**H_3), 55.4 (1 C, -**C**H), 54.0 (1 C, -**C**H), 34.2 (2 C, -**C**(CH_3)$_3$), 30.1 (6 C, -C(**C**(CH_3)$_3$).

4-Methylthiophenyl benzo[c]oxepinone derivative (6k)

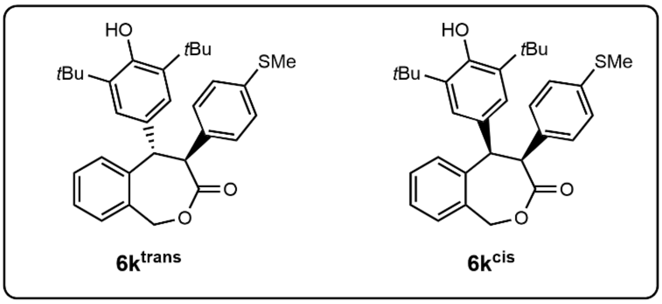

7-membered ring **6k** was synthesized according to general procedure **F** and obtained as a colorless residue in a yield of 55% (57% for the alkylation step) as a mixture of diastereomers (*dr* 40:60; er_{trans} 96:4; er_{cis} 90:10).

Data for the alkylation product (mixture of diastereomers) 5k:

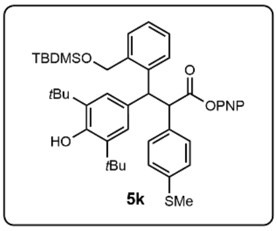

^{1}H-NMR (500 MHz, $CDCl_3$, 298 K) δ / ppm = *Signals for the unlike diastereomer:* 8.10 (d, *J* = 9.2 Hz, 2 H, Ar-**H**), 7.40-7.31 (m, 2 H, Ar-**H**), 7.40 (d, *J* = 8.4 Hz, 2 H, Ar-**H**), 7.23 (s, 2 H, Ar-**H**), 7.19-7.11 (m, 2 H, Ar-**H**), 7.11 (d, *J* = 8.4 Hz, 2 H, Ar-**H**), 6.60 (d, *J* = 9.2 Hz, 2 H, Ar-**H**), 5.17 (s, 1 H, -O**H**), 4.96 (d, *J* = 12.2 Hz, 1 H, -C**H₂**), 4.81 (d, *J* = 13.3 Hz, 1 H, -C**H**), 4.72 (d, *J* =12.2 Hz, 1 H, -C**H₂**), 4.63 (d, *J* = 13.1 Hz, 1 H, -C**H**), 2.41 (s, 3 H, -SC**H₃**), 1.39 (s, 18 H, -C**H₃**), 0.96 (s, 9 H, -SiC(C**H₃**)₃), 0.11 (s, 3 H, -SiC**H₃**), 0.08 (s, 3 H, -SiC**H₃**). *Selected signals for the like diastereomer:* 8.13 (d, *J* = 9.2 Hz, 2 H, Ar-**H**), 7.71 (d, *J* = 7.5 Hz, 1 H, Ar-**H**), 7.54 (d, *J* = 7.6 Hz, 1 H, Ar-**H**), 6.77 (d, *J* = 9.2 Hz, 2 H, Ar-**H**), 6.56 (s, 2 H, Ar-**H**), 4.96 (s, 1 H, -O**H**), 4.89 (d, *J* = 13.6 Hz, 1 H, -C**H**), 4.60 (d, *J* = 11.8 Hz, 1 H, -C**H₂**), 4.59 (d, *J* = 13.6 Hz, 1 H, -C**H**), 4.41 (d, *J* = 11.8 Hz, 1 H, -C**H₂**), 2.46 (s, 3 H, -SC**H₃**), 1.23 (s, 18 H, -C**H₃**), 0.84 (s, 9 H, -SiC(C**H₃**)₃), -0.07 (s, 3 H, -SiC**H₃**), -0.07 (s, 3 H, -SiC**H₃**).

^{13}C-NMR (125 MHz, $CDCl_3$, 298 K) δ / ppm = *Signals for the unlike diastereomer:* 171.2 (1 C, -**C**OOR), 155.4 (1 C, C_{Ar}), 152.9 (1 C, C_{Ar}), 145.4 (1 C, C_{Ar}), 139.0 (1 C, C_{Ar}), 138.2 (1 C, C_{Ar}), 137.8 (1 C, C_{Ar}), 136.0 (2 C, C_{Ar}), 132.6 (1 C, C_{Ar}), 131.7 (1 C, C_{Ar}), 129.2 (2 C, C_{Ar}), 127.2 (1 C, C_{Ar}), 127.1 (1 C, C_{Ar}), 126.6 (1 C, C_{Ar}), 126.5 (2 C, C_{Ar}), 126.3 (1 C, C_{Ar}), 125.5 (2 C, C_{Ar}), 125.1 (2 C, C_{Ar}), 122.5 (2 C, C_{Ar}), 63.1 (1 H, -**C**H_2), 57.2 (1 C, -**C**H), 48.4 (1 C, -**C**H), 34.5 (2 C, -**C**$(CH_3)_3$), 30.4 (6 C, -C(**C**$H_3)_3$), 26.1 (3 C, -SiC(**C**$H_3)_3$), 18.6 (1 C, -Si**C**$(CH_3)_3$), 15.5 (1 C, -S**C**H_3), -5.2 (1 C, -Si**C**H_3), -5.2 (1 C, -Si**C**H_3). *Selected signals for the like diastereomer:* 170.7 (1 C, -**C**OOR), 152.3 (1 C, C_{Ar}), 139.6 (1 C, C_{Ar}), 139.0 (1 C, C_{Ar}), 138.3 (1 C, C_{Ar}), 135.3 (2 C, C_{Ar}), 133.3 (1 C, C_{Ar}), 129.8 (1 C, C_{Ar}), 129.2 (2 C, C_{Ar}), 127.4 (1 C, C_{Ar}), 127.0 (1 C, C_{Ar}), 126.9 (1 C, C_{Ar}), 126.6 (2 C, C_{Ar}), 125.4 (2 C, C_{Ar}), 125.2 (2 C, C_{Ar}), 122.5 (2 C, C_{Ar}), 62.8 (1 C, -**C**H_2), 57.6 (1 C, -**C**H), 50.8 (1 C, -**C**H), 34.2 (2 C, -**C**$(CH_3)_3$), 30.2 (6 C, -C(**C**$H_3)_3$), 26.0 (3 C, -SiC(**C**$H_3)_3$), 18.5 (1 C, -Si**C**$(CH_3)_3$), 15.8 (1 C, -S**C**H_3), -5.3 (1 C, -Si**C**H_3), -5.4 (1 C, -Si**C**H_3).

HRMS (ESI-TOF): *m/z:* $[M+NH_4]^+$ calcd for $C_{43}H_{59}N_2O_6SSi^+$: 659.3858, found 659.3860.

Data for the cyclic product 6k:

HPLC (YMC-SB, *n*-hexane/IPA = 10/1, flow = 1.0 mL min^{-1}, T_{Column} = 10 °C, *l* = 220 nm) t_r*(trans):* 11.78 min (major), 24.72 min (minor); t_r*(cis):* 11.32 min (major), 19.23 min (minor).

α_D^{20}(c = 1, $CHCl_3$): -33.1 (mixture of diastereomers).

HRMS (ESI-TOF): *m/z:* $[M+NH_4]^+$ calcd for $C_{31}H_{40}NO_3S^+$: 506.2723, found 506.2725.

NMR data for the cis diastereomer:

^{1}H-NMR (500 MHz, $CDCl_3$, 298 K) *δ* / ppm = 7.24-7.22 (m, 3 H, Ar-**H**), 7.13 (d, *J* = 8.4 Hz, 2 H, Ar-**H**), 7.08-7.05 (m, 1 H, Ar-**H**), 6.92 (d, *J* = 8.4 Hz, 2 H, Ar-**H**), 6.56 (s, 2 H, Ar-**H**), 5.90 (d, *J* = 15.1 Hz, 1 H, -C**H₂**), 5.21 (d, *J* = 15.1 Hz, 1 H, -C**H₂**), 5.13 (s, 1 H, -O**H**), 4.91 (d, *J* = 3.4 Hz, 1 H, -C**H**), 4.23 (d, *J* = 3.4 Hz, 1 H, -C**H**), 2.46 (s, 3 H, -SC**H₃**), 1.31 (s, 18 H, -C**H₃**).

^{13}C-NMR (125 MHz, $CDCl_3$, 298 K) *δ* / ppm = 172.1 (1 C, -**C**OOR), 153.2 (1 C, $\mathbf{C_{Ar}}$), 141.1 (1 C, $\mathbf{C_{Ar}}$), 137.6 (1 C, $\mathbf{C_{Ar}}$), 135.2 (2 C, $\mathbf{C_{Ar}}$), 133.8 (1 C, $\mathbf{C_{Ar}}$), 132.5 (1 C, $\mathbf{C_{Ar}}$), 132.4 (1 C, $\mathbf{C_{Ar}}$), 131.3 (2 C, $\mathbf{C_{Ar}}$), 130.9 (1 C, $\mathbf{C_{Ar}}$), 129.0 (1 C, $\mathbf{C_{Ar}}$), 128.1 (1 C, $\mathbf{C_{Ar}}$), 127.2 (2 C, $\mathbf{C_{Ar}}$), 126.6 (1 C, $\mathbf{C_{Ar}}$), 126.1 (2 C, $\mathbf{C_{Ar}}$), 70.6 (1 C, -**C**H_2), 55.2 (1 C, -**C**H), 49.8 (1 C, -**C**H), 34.4 (2 C, -**C**(CH_3)$_3$), 30.3 (6 C, -C(**C**H_3)$_3$), 16.2 (1 C, -S**C**H_3).

NMR data for the trans diastereomer:

^{1}H-NMR (500 MHz, $CDCl_3$, 298 K) *δ* / ppm = 7.27-7.20 (m, 3 H, Ar-**H**), 7.15 (d, *J* = 8.4 Hz, 2 H, Ar-**H**), 7.12-7.09 (m, 1 H, Ar-**H**), 7.08 (d, *J* = 8.4 Hz, 2 H, Ar-**H**), 6.44 (s, 2 H, Ar-**H**), 5.85 (d, *J* = 13.7 Hz, 1 H, -C**H₂**), 5.05 (d, *J* = 13.7 Hz, 1 H, -C**H₂**), 5.00 (s, 1 H, -O**H**), 4.50 (d, *J* = 10.4 Hz, 1 H, -C**H**), 4.45 (d, *J* = 10.4 Hz, 1 H, -C**H**), 2.45 (s, 3 H, -SC**H₃**), 1.23 (s, 18 H, -C**H₃**).

^{13}C-NMR (125 MHz, $CDCl_3$, 298 K) *δ* / ppm = 172.5 (1 C, -**C**OOR), 152.5 (1 C, $\mathbf{C_{Ar}}$), 140.9 (1 C, $\mathbf{C_{Ar}}$), 137.7 (1 C, $\mathbf{C_{Ar}}$), 135.7 (2 C, $\mathbf{C_{Ar}}$), 134.7 (1 C, $\mathbf{C_{Ar}}$), 134.5 (1 C, $\mathbf{C_{Ar}}$), 133.5 (1 C, $\mathbf{C_{Ar}}$), 132.5 (1 C, $\mathbf{C_{Ar}}$), 130.1 (2 C, $\mathbf{C_{Ar}}$), 129.7 (1 C, $\mathbf{C_{Ar}}$), 129.4 (1 C, $\mathbf{C_{Ar}}$), 126.9 (1 C, $\mathbf{C_{Ar}}$), 126.7 (2 C, $\mathbf{C_{Ar}}$), 125.0 (2 C, $\mathbf{C_{Ar}}$), 70.3 (1 C, -**C**H_2), 55.4 (1 C, -**C**H), 54.2 (1 C, -**C**H), 34.3 (2 C, -**C**(CH_3)$_3$), 30.2 (6 C, -C(**C**H_3)$_3$), 16.1 (1 C, -S**C**H_3).

2-Methylphenyl benzo[c]oxepinone derivative (6l)

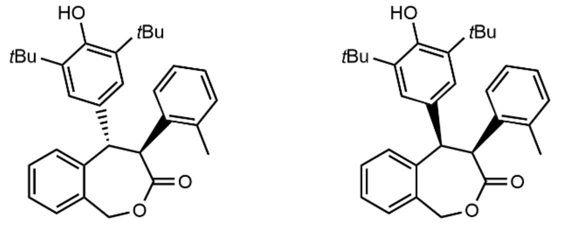

$\mathbf{6l^{trans}}$ $\mathbf{6l^{cis}}$

7-membered ring **6l** was synthesized according to general procedure **F** and obtained as a colorless residue in a yield of 35% (43% for the alkylation step) as a mixture of diastereomers (*dr* 70:30; er_{trans} 97:3; er_{cis} 96:4).

Data for the alkylation product (mixture of diastereomers) 5l:

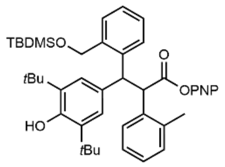

5l

Here, also the racemic product was formed with an dr of 50:50 only. Therefore, assignment of the signals to the cis/trans diastereomer was not possible unambiguously. Selected multiplets were assigned according to similarity with other entries to the substrate scope.

^{1}H-NMR (500 MHz, $CDCl_3$, 298 K) δ / ppm = *Selected signals for the unlike diastereomer:* 8.10 (d, *J* = 9.1 Hz, 2 H, Ar-**H**), 7.23 (s, 2 H, Ar-**H**), 6.63 (d, *J* = 9.1 Hz, 2 H, Ar-**H**), 5.16 (s, 1 H, -O**H**), 5.13 (d, *J* = 12.1 Hz, 1 H, -C**H$_2$**), 4.99 (d, *J* = 12.1 Hz, 1 H, -C**H$_2$**), 4.86 (d, *J* = 13.2 Hz, 1 H, -C**H**), 4.63 (d, *J* = 13.2 Hz, 1 H, -C**H**), 1.40 (s, 18 H, -C**H$_3$**), 0.97 (s, 9 H, -SiC(C**H$_3$**)$_3$), 0.12 (s, 3 H, -SiC**H$_3$**), 0.09 (s, 3 H, -SiC**H$_3$**). *Selected Signals for the like diastereomer:* 8.12 (d, *J* = 9.1 Hz, 2 H, Ar-**H**), 6.80 (d, *J* = 9.1 Hz, 2 H, Ar-**H**), 6.54 (s, 2 H, Ar-**H**), 4.94 (s, 1 H, -O**H**), 4.88 (d, *J* = 13.5 Hz, 1 H, -C**H**), 4.83 (d, *J* = 11.9 Hz, 1 H, -C**H$_2$**), 4.66 (d, *J* = 11.9 Hz, 1 H, -C**H$_2$**), 4.56 (d, *J* = 13.5 Hz, 1 H, -C**H**), 1.20 (s, 18 H, -C**H$_3$**), 0.84 (9 H, -SiC(C**H$_3$**)$_3$), -0.09 (s, 3 H, -SiC**H$_3$**), -0.09 (s, 3 H, -SiC**H$_3$**). *Signals for both diastereomers:* 7.81 (d, *J* = 8.3 Hz, 1 H, Ar-**H**), 7.77 (d, *J* = 7.6 Hz, 1 H, Ar-**H**), 7.64-7.62 (m, 1 H, Ar-**H**), 7.55 (d, *J* = 7.4 Hz, 1 H, Ar-**H**), 7.42 (t, *J* = 7.4 Hz, 1 H, Ar-**H**), 7.36-7.30 (m, 3 H, Ar-**H**), 7.25-7.23 (m, 1 H, Ar-**H**), 7.18-7.17 (m, 1 H, Ar-**H**), 7.15-7.08 (m, 5 H, Ar-**H**), 6.98 (d, *J* = 7.5 Hz, 1 H, Ar-**H**), 2.53 (s, 3 H, -C**H$_3$**), 1.83 (s, 3 H, -C**H$_3$**).

^{13}C-NMR (125 MHz, $CDCl_3$, 298 K) δ / ppm = *Selected signals for the unlike diastereomer:* 171.1 (1 C, -**C**OOR), 63.2 (1 C, -**C**H$_2$), 52.3 (1 C, -**C**H), 48.2 (1 C, -**C**H), 34.5 (2 C, -**C**(CH$_3$)$_3$), 30.4 (6 C, -C(**C**H$_3$)$_3$), 26.2 (3 C, -SiC(**C**H$_3$)$_3$), 20.2 (1 C, -**C**H$_3$), 18.6 (1 C, -Si**C**(CH$_3$)$_3$), -5.1 (1 C, -Si**C**H$_3$), -5.2 (1 C, -Si**C**H$_3$). *Selected Signals for the like diastereomer:* 170.9 (1 C, -**C**OOR), 62.9 (1 C, -**C**H$_2$), 53.3 (1 C, -**C**H), 50.8 (1 C, -**C**H), 34.2 (2 C, -**C**(CH$_3$)$_3$), 30.1 (6 C, -C(**C**H$_3$)$_3$), 26.1 (3 C, -SiC(**C**H$_3$)$_3$), 19.3 (1 C, -**C**H$_3$), 18.5 (1 C, -Si**C**(CH$_3$)$_3$), -5.2 (1 C, -Si**C**H$_3$), -5.4 (1 C, -Si**C**H$_3$). *Signals for both diastereomers:* 155.5 (1 C, **C$_{Ar}$**), 155.5 (1 C, **C$_{Ar}$**), 152.9 (1 C, **C$_{Ar}$**), 152.4 (1 C, **C$_{Ar}$**), 145.4 (2 C, **C$_{Ar}$**), 139.7 (1 C, **C$_{Ar}$**), 139.4 (1 C, **C$_{Ar}$**), 139.0 (1 C, **C$_{Ar}$**), 138.1 (1 C, **C$_{Ar}$**), 137.9 (1 C, **C$_{Ar}$**), 136.6 (1 C, **C$_{Ar}$**), 136.0 (2 C, **C$_{Ar}$**),

135.3 (1 C, **C**$_{Ar}$), 135.1 (2 C, **C**$_{Ar}$), 134.4 (1 C, **C**$_{Ar}$), 131.9 (1 C, **C**$_{Ar}$), 130.6 (1 C, **C**$_{Ar}$), 130.6 (1 C, **C**$_{Ar}$), 129.5 (1 C, **C**$_{Ar}$), 127.9 (1 C, **C**$_{Ar}$), 127.7 (1 C, **C**$_{Ar}$), 127.7 (1 C, **C**$_{Ar}$), 127.6 (1 C, **C**$_{Ar}$), 127.3 (1 C, **C**$_{Ar}$), 127.3 (1 C, **C**$_{Ar}$), 127.0 (1 C, **C**$_{Ar}$), 127.0 (1 C, **C**$_{Ar}$), 126.8 (1 C, **C**$_{Ar}$), 126.8 (1 C, **C**$_{Ar}$), 126.6 (1 C, **C**$_{Ar}$), 126.5 (1 C, **C**$_{Ar}$), 126.0 (1 C, **C**$_{Ar}$), 125.7 (2 C, **C**$_{Ar}$), 125.6 (2 C, **C**$_{Ar}$), 125.3 (1 C, **C**$_{Ar}$), 125.1 (2 C, **C**$_{Ar}$), 125.0 (2 C, **C**$_{Ar}$), 122.5 (4 C, **C**$_{Ar}$).

HRMS (ESI-TOF): *m/z:* $[M+H]^+$ calcd for $C_{43}H_{56}NO_6Si^+$: 710.3871, found 710.3876.

Data for the cyclic product 6l:

HPLC (YMC-SB, *n*-hexane/IPA = 10/1, flow = 1.0 mL min^{-1}, T_{Column} = 10 °C, *l* = 240 nm) *t$_r$(trans):* 13.23 min (major), 33.89 min (minor); *t$_r$(cis):* 7.83 min (major), 13.04 min (minor).

$\boldsymbol{\alpha_D^{20}}$(c = 1, $CHCl_3$): -40.7 (mixture of diastereomers).

HRMS (ESI-TOF): *m/z*: $[M+NH_4]^+$ calcd for $C_{31}H_{40}NO_3^+$: 474.3003, found 474.3004.

NMR data for the cis diastereomer:

^{1}H-NMR (500 MHz, $CDCl_3$, 298 K) *δ* / ppm = 7.24-7.22 (m, 3 H, Ar-**H**), 7.17-7.16 (m, 2 H, Ar-**H**), 7.09-7.07 (m, 1 H, Ar-**H**), 7.03-6.99 (m, 1 H, Ar-**H**), 6.86 (d, *J* = 7.7 Hz, 1 H, Ar-**H**), 6.55 (s, 2 H, Ar-**H**), 5.91 (d, *J* = 15.1 Hz, 1 H, -C**H$_2$**), 5.26 (d, *J* = 3.1 Hz, 1 H, -C**H**), 5.24 (d, *J* = 15.1 Hz, 1 H, -C**H$_2$**), 5.11 (s, 1 H, -O**H**), 4.21 (d, *J* = 3.1 Hz, 1 H, -C**H**), 2.37 (s, 3 H, -C**H$_3$**), 1.30 (s, 18 H, -C**H$_3$**).

^{13}C-NMR (125 MHz, $CDCl_3$, 298 K) *δ* / ppm = 172.1 (1 C, -**C**OOR), 153.0 (1 C, **C**$_{Ar}$), 141.2 (1 C, **C**$_{Ar}$), 135.0 (2 C, **C**$_{Ar}$), 134.6 (1 C, **C**$_{Ar}$), 133.6 (1 C, **C**$_{Ar}$), 132.4 (1 C, **C**$_{Ar}$), 132.2 (1 C, **C**$_{Ar}$), 130.9 (1 C, **C**$_{Ar}$), 130.0 (1 C, **C**$_{Ar}$), 129.5 (1 C, **C**$_{Ar}$), 128.9 (1 C, **C**$_{Ar}$), 127.9 (1 C, **C**$_{Ar}$), 127.1 (2 C, **C**$_{Ar}$), 126.5 (1 C, **C**$_{Ar}$), 125.0 (1 C, **C**$_{Ar}$), 124.8 (1 C, **C**$_{Ar}$), 70.6 (1 C, -**C**H$_2$), 53.5 (1 C, -**C**H), 44.1 (1 C, -**C**H), 34.2 (2 C, -**C**(CH$_3$)$_3$), 30.2 (6 C, -C(**C**H$_3$)$_3$).

NMR data for the trans diastereomer:

^{1}H-NMR (500 MHz, $CDCl_3$, 298 K) *δ* / ppm = 7.71 (d, *J* = 7.8 Hz, 1 H, Ar-**H**), 7.33-7.30 (m, 2 H, Ar-**H**), 7.29-7.26 (m, 1 H, Ar-**H**), 7.24-7.21 (m, 1 H, Ar-**H**), 7.15-7.11 (m, 2 H, Ar-**H**), 6.94 (d, *J* = 7.5 Hz, 1 H, Ar-**H**), 6.42 (s, 2 H, Ar-**H**), 5.99 (d, *J* = 13.5 Hz, 1 H, -C**H$_2$**), 5.09 (d, *J* = 13.5 Hz, 1 H, -C**H$_2$**), 5.00 (s, 1 H, -O**H**), 4.81 (d, *J* = 10.8 Hz, 1 H, -C**H**), 4.53 (d, *J* = 10.8 Hz, 1 H, -C**H**), 1.68 (s, 3 H, -C**H$_3$**), 1.20 (s, 18 H, -C**H$_3$**).

^{13}C-NMR (125 MHz, $CDCl_3$, 298 K) *δ* / ppm = 172.3 (1 C, -**C**OOR), 152.5 (1 C, **C**$_{Ar}$), 141.4 (1 C, **C**$_{Ar}$), 136.6 (1 C, **C**$_{Ar}$), 136.4 (1 C, **C**$_{Ar}$), 135.6 (1 C, **C**$_{Ar}$), 134.2 (1 C, **C**$_{Ar}$), 133.7 (1 C, **C**$_{Ar}$), 132.4 (1 C, **C**$_{Ar}$), 130.5 (1 C, **C**$_{Ar}$), 129.9 (1 C, **C**$_{Ar}$), 129.8 (1 C, **C**$_{Ar}$), 129.6 (1 C, **C**$_{Ar}$), 127.3 (1 C, **C**$_{Ar}$), 127.0 (1 C, **C**$_{Ar}$), 126.2 (1 C, **C**$_{Ar}$), 124.9 (2 C, **C**$_{Ar}$), 70.3 (1 C, -**C**H$_2$), 55.2 (1 C, -**C**H), 49.9 (1 C, -**C**H), 34.2 (2 C, -**C**(CH$_3$)$_3$), 30.1 (6 C, -C(**C**H$_3$)$_3$), 9.3 (1 C, -**C**H$_3$).

3-Methylphenyl benzo[c]oxepinone derivative (6m)

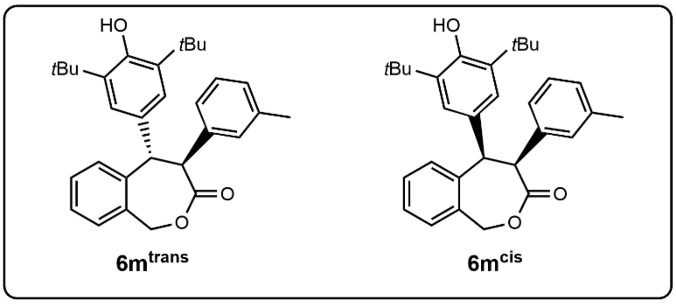

7-membered ring **6m** was synthesized according to general procedure **F** and obtained as a colorless residue in a yield of 32% (42% for the alkylation step) as a mixture of diastereomers (*dr* 55:45; er_{trans} 94:6; er_{cis} 86:14).

Data for the alkylation product (mixture of diastereomers) 5m:

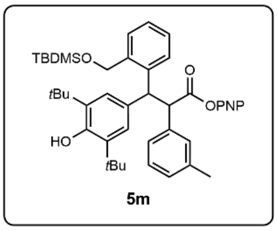

^{1}H-NMR (500 MHz, $CDCl_3$, 298 K) *δ* / ppm = *Signals for the unlike diastereomer:* 8.09 (d, *J* = 9.1 Hz, 2 H, Ar-**H**), 7.41 (d, *J* = 7.8 Hz, 1 H, Ar-**H**), 7.33-7.31 (m, 1 H, Ar-**H**), 7.25-7.22 (m, 2 H, Ar-**H**), 7.22 (s, 2 H, Ar-**H**), 7.18-7.14 (m, 1 H, Ar-**H**), 7.13-7.09 (m, 2 H, Ar-**H**), 6.99 (d, *J* = 7.6 Hz, 1 H, Ar-**H**), 6.61 (d, *J* = 9.1 Hz, 2 H, Ar-**H**), 5.14 (s, 1 H, -O**H**), 4.90 (d, *J* = 12.2 Hz, 1 H, -C**H₂**), 4.74 (d, *J* = 13.3 Hz, 1 H, -C**H**), 4.70 (d, *J* = 12.2 Hz, 1 H, -C**H₂**), 4.65 (d, *J* = 13.3 Hz, 1 H, -C**H**), 2.27 (s, 3 H, -C**H₃**), 1.39 (s, 18 H, -C**H₃**), 0.94 (s, 9 H, -SiC(C**H₃**)₃), 0.08 (s, 3 H, -SiC**H₃**), 0.06 (s, 3 H, -SiC**H₃**). *Selected signals for the like diastereomer:* 8.12 (d, *J* = 9.1 Hz, 2 H, Ar-**H**), 7.71 (d, *J* = 7.7 Hz, 1 H, Ar-**H**), 7.53 (d, *J* = 7.8 Hz, 1 H, Ar-**H**), 7.42-7.37 (m, 1 H, Ar-**H**), 7.33-7.31 (m, 1 H, Ar-**H**), 7.18-7.15 (m, 1 H, Ar-**H**), 7.04 (d, *J* = 7.7 Hz, 2 H, Ar-**H**), 6.91 (s, 1 H, Ar-**H**), 6.79 (d, *J* = 9.1 Hz, 2 H, Ar-**H**), 6.56 (s, 2 H, Ar-**H**), 4.92 (s, 1 H, -O**H**), 4.89 (d, *J* = 13.6 Hz, 1 H, -C**H**), 4.63 (d, *J* = 11.7 Hz, 1 H, -C**H₂**), 4.59 (d, *J* = 13.6 Hz, 1 H, -C**H**), 4.41 (d, *J* = 11.7 1 H, -C**H₂**), 2.25 (s, 3 H, -C**H₃**), 1.22 (s, 18 H, -C**H₃**), 0.85 (s, 9 H, -SiC(C**H₃**), -0.06 (s, 3 H, -SiC**H₃**), -0.07 (s, 3 H, -SiC**H₃**).

^{13}C-NMR (125 MHz, $CDCl_3$, 298 K) *δ* / ppm = *Signals for the unlike diastereomer:* 171.3 (1 C, -**C**OOR), 155.5 (1 C, $\mathbf{C_{Ar}}$), 152.9 (1 C, $\mathbf{C_{Ar}}$), 145.5 (1 C, $\mathbf{C_{Ar}}$), 139.1 (1 C, $\mathbf{C_{Ar}}$), 138.3 (1 C, $\mathbf{C_{Ar}}$), 137.8 (1 C, $\mathbf{C_{Ar}}$), 136.1 (2 C, $\mathbf{C_{Ar}}$), 135.3 (1 C, $\mathbf{C_{Ar}}$), 132.0 (1 C, $\mathbf{C_{Ar}}$), 129.4 (1 C, $\mathbf{C_{Ar}}$), 128.8 (1 C, $\mathbf{C_{Ar}}$), 128.6 (1 C, $\mathbf{C_{Ar}}$), 126.9 (1 C, $\mathbf{C_{Ar}}$), 126.8 (1 C, $\mathbf{C_{Ar}}$), 126.5 (1 C, $\mathbf{C_{Ar}}$), 126.4 (1 C, $\mathbf{C_{Ar}}$), 125.9 (1 C, $\mathbf{C_{Ar}}$), 125.5 (2 C, $\mathbf{C_{Ar}}$), 125.1 (2 C, $\mathbf{C_{Ar}}$), 122.6 (2 C, $\mathbf{C_{Ar}}$), 62.9 (1 C, -**C**H₂), 57.8 (1 C, -**C**H), 48.7 (1 C, -**C**H), 34.5 (2 C, -**C**(CH₃)₃), 30.4 (6 C, -C(**C**H₃)₃), 26.2 (3 C, -SiC(**C**H₃)₃), 21.6 (1 C, -**C**H₃), 18.6 (1 C, -Si**C**(CH₃)₃), -5.1 (1 C, -Si**C**H₃), -5.2 (1 C, -Si**C**H₃). *Selected signals for the like diastereomer:* 170.8 (1 C, -**C**OOR), 152.3 (1 C, $\mathbf{C_{Ar}}$), 136.5 (1 C, $\mathbf{C_{Ar}}$), 135.9 (2 C, $\mathbf{C_{Ar}}$), 129.9 (1 C, $\mathbf{C_{Ar}}$), 128.6 (1 C, $\mathbf{C_{Ar}}$), 128.5 (1 C, $\mathbf{C_{Ar}}$), 127.0 (1 C, $\mathbf{C_{Ar}}$), 127.0 (1 C, $\mathbf{C_{Ar}}$), 125.4 (2 C, $\mathbf{C_{Ar}}$), 125.2 (2 C, $\mathbf{C_{Ar}}$), 62.9 (1 C, -**C**H₂), 58.1 (1 C, -**C**H), 50.8 (1 C, -**C**H), 34.2 (2 C, -**C**(CH₃)₃), 30.2 (6 C, -C(**C**H₃)₃), 26.1 (3 C, -SiC(**C**H₃)₃), 21.4 (1 C, -**C**H₃), 18.5 (1 C, -Si**C**(CH₃)₃), -5.2 (1 C, -Si**C**H₃), -5.3 (1 C, -Si**C**H₃).

HRMS (ESI-TOF): *m/z:* $[M+NH_4]^+$ calcd for $C_{43}H_{59}N_2O_6Si^+$: 727.4137, found 727.4139.

Data for the cyclic product 6m:

HPLC (YMC-SB, *n*-hexane/IPA = 10/1, flow = 1.0 mL min^{-1}, T_{Column} = 10 °C, *l* = 240 nm) t_r*(trans):* 13.07 min (major), 32.32 min (minor); t_r*(cis):* 12.30 min (major), 14.29 min (minor).

α_D^{20}(c = 1, $CHCl_3$): -21.6 (mixture of diastereomers).

HRMS (ESI-TOF): *m/z*: $[M+NH_4]^+$ calcd for $C_{31}H_{40}NO_3^+$: 474.3003, found 474.3001.

NMR data for the cis diastereomer:

^{1}H-NMR (500 MHz, $CDCl_3$, 298 K) *δ* / ppm = 7.23-7.20 (m, 3 H, Ar-**H**), 7.14-7.12 (m, 1 H, Ar-**H**), 7.09-7.04 (m, 2 H, Ar-**H**), 6.89 (br. s, 1 H, Ar-**H**), 6.81 (d, *J* = 7.7 Hz, 1 H, Ar-**H**), 6.60 (s, 2 H, Ar-**H**), 5.90 (d, *J* = 15.2 Hz, 1 H, -C**H₂**), 5.22 (d, *J* = 15.2 Hz, 1 H, -C**H₂**), 5.11 (s, 1 H, -O**H**), 4.91 (d, *J* = 3.3 Hz, 1 H, -C**H**), 4.33 (d, *J* = 3.3 Hz, 1 H, -C**H**), 2.29 (s, 3 H, -C**H₃**), 1.32 (s, 18 H, -C**H₃**).

^{13}C-NMR (125 MHz, $CDCl_3$, 298 K) *δ* / ppm = 172.2 (1 C, -**C**OOR), 153.1 (1 C, $\mathbf{C_{Ar}}$), 141.4 (1 C, $\mathbf{C_{Ar}}$), 137.2 (1 C, $\mathbf{C_{Ar}}$), 136.8 (1 C, $\mathbf{C_{Ar}}$), 135.1 (2 C, $\mathbf{C_{Ar}}$), 132.5 (1 C, $\mathbf{C_{Ar}}$), 132.3 (1 C, $\mathbf{C_{Ar}}$), 131.3 (1 C, $\mathbf{C_{Ar}}$), 131.1 (1 C, $\mathbf{C_{Ar}}$), 129.0 (1 C, $\mathbf{C_{Ar}}$), 128.3 (1 C, $\mathbf{C_{Ar}}$), 128.0 (1 C, $\mathbf{C_{Ar}}$), 127.9 (1 C, $\mathbf{C_{Ar}}$), 127.6 (1 C, $\mathbf{C_{Ar}}$), 127.2 (2 C, $\mathbf{C_{Ar}}$), 126.5 (1 C, $\mathbf{C_{Ar}}$), 70.6 (1 C, -**C**H_2), 55.0 (1 C, -**C**H), 50.2 (1 C, -**C**H), 34.4 (2 C, -**C**$(CH_3)_3$), 30.3 (6 C, -C(**C**$H_3)_3$), 21.7 (1 C, -**C**H_3).

NMR data for the trans diastereomer:

^{1}H-NMR (500 MHz, $CDCl_3$, 298 K) *δ* / ppm = 7.27-7.25 (m, 2 H, Ar-**H**), 7.23-7.19 (m, 1 H, Ar-**H**), 7.17-7.12 (m, 2 H, Ar-**H**), 7.03 (d, *J* = 7.6 Hz, 1 H, Ar-**H**), 7.01 (d, *J* = 7.6 Hz, 1 H, Ar-**H**), 6.94 (br. s, 1 H, Ar-**H**), 6.57 (s, 2 H, Ar-**H**), 5.79 (d, *J* = 13.6 Hz, 1 H, -C**H₂**), 5.06 (d, *J* = 13.6 Hz, 1 H, -C**H₂**), 4.98 (s, 1 H, -O**H**), 4.57 (d, *J* = 10.1 Hz, 1 H, -C**H**), 4.46 (d, *J* = 10.1 Hz, 1 H, -C**H**), 2.27 (s, 3 H, -C**H₃**), 1.23 (s, 18 H, -C**H₃**).

^{13}C-NMR (125 MHz, $CDCl_3$, 298 K) *δ* / ppm = 172.6 (1 C, -**C**OOR), 152.4 (1 C, $\mathbf{C_{Ar}}$), 141.0 (1 C, $\mathbf{C_{Ar}}$), 137.8 (1 C, $\mathbf{C_{Ar}}$), 137.6 (1 C, $\mathbf{C_{Ar}}$), 135.6 (2 C, $\mathbf{C_{Ar}}$), 134.5 (1 C, $\mathbf{C_{Ar}}$), 133.7 (1 C, $\mathbf{C_{Ar}}$), 132.4 (1 C, $\mathbf{C_{Ar}}$), 130.3 (1 C, $\mathbf{C_{Ar}}$), 129.6 (1 C, $\mathbf{C_{Ar}}$), 129.4 (1 C, $\mathbf{C_{Ar}}$), 128.3 (1 C, $\mathbf{C_{Ar}}$), 128.2 (1 C, $\mathbf{C_{Ar}}$), 126.9 (1 C, $\mathbf{C_{Ar}}$), 126.5 (1 C, $\mathbf{C_{Ar}}$), 125.0 (2 C, $\mathbf{C_{Ar}}$), 70.2 (1 C, -**C**H_2), 56.1 (1 C, -**C**H), 53.8 (1 C, -**C**H), 34.3 (2 C, -**C**$(CH_3)_3$), 30.2 (6 C, -C(**C**$H_3)_3$), 21.5 (1 C, -**C**H_3).

4-Methylphenyl benzo[c]oxepinone derivative (6n)

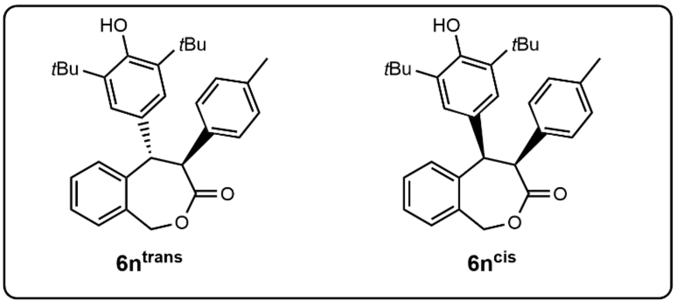

7-membered ring **6n** was synthesized according to general procedure **F** and obtained as a colorless residue in a yield of 50% (54% for the alkylation step) as a mixture of diastereomers (*dr* 60:40; er_{trans} 96:4; er_{cis} 93:7).

Data for the alkylation product (mixture of diastereomers) 5n:

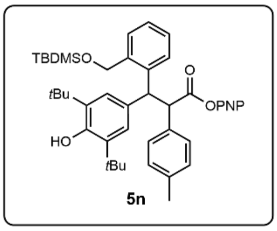

^{1}H-NMR (500 MHz, $CDCl_3$, 298 K) δ / ppm = *Signals for the unlike diastereomer:* 8.09 (d, *J* = 9.0 Hz, 2 H, Ar-**H**), 7.41 (d, *J* = 7.9 Hz, 1 H, Ar-**H**), 7.34 (d, *J* = 8.0 Hz, 2 H, Ar-**H**), 7.32-7.30 (m, 1 H, Ar-**H**), 7.22 (s, 2 H, Ar-**H**), 7.17 (t, *J* = 7.3 Hz, 1 H, Ar-**H**), 7.07-7.06 (m, 1 H, Ar-**H**), 7.03 (d, *J* = 8.0 Hz, 2 H, Ar-**H**), 6.60 (d, *J* = 9.0 Hz, 2 H, Ar-**H**), 5.14 (s, 1 H, -O**H**), 4.93 (d, *J* = 12.2 Hz, 1 H, -C**H₂**), 4.77 (d, *J* = 13.3 Hz, 1 H, -C**H**), 4.71 (d, *J* = 12.2 Hz, 1 H, -C**H₂**), 4.64 (d, *J* = 13.3 Hz, 1 H, -C**H**), 2.25 (s, 3 H, -C**H₃**), 1.39 (s, 18 H, -C**H₃**), 0.95 (s, 9 H, -SiC(C**H₃**)₃), 0.09 (s, 3 H, -SiC**H₃**), 0.07 (s, 3 H, -SiC**H₃**). *Selected signals for the like diastereomer:* 8.11 (d, *J* = 9.1 Hz, 2 H, Ar-**H**), 7.71 (d, *J* = 7.9 Hz, 1 H, Ar-**H**), 7.53 (d, *J* = 8.9 Hz, 1 H, Ar-**H**), 7.38 (d, *J* = 6.9 Hz, 1 H, Ar-**H**), 7.32-7.30 (m, 1 H, Ar-**H**), 7.11 (d, *J* = 7.2 Hz, 2 H, Ar-**H**), 7.09-7.08 (m, 1 H, Ar-**H**), 6.77 (d, *J* = 9.1 Hz, 2 H, Ar-**H**), 6.54 (s, 2 H, Ar-**H**), 4.91 (s, 1 H, -O**H**), 4.88 (d, *J* = 13.6 Hz, 1 H, -C**H**), 4.60 (d, *J* = 11.9 Hz, 1 H, -C**H₂**), 4.58 (d, *J* = 13.6 Hz, 1 H, -C**H**), 4.40 (d, *J* = 11.9 Hz, 1 H, -C**H₂**),

^{13}C-NMR (125 MHz, $CDCl_3$, 298 K) δ / ppm = *Signals for the unlike diastereomer:* 171.3 (1 C, -**C**OOR), 155.6 (1 C, $\mathbf{C_{Ar}}$), 152.9 (1 C, $\mathbf{C_{Ar}}$), 145.5 (1 C, $\mathbf{C_{Ar}}$), 139.1 (1 C, $\mathbf{C_{Ar}}$), 138.0 (1 C, $\mathbf{C_{Ar}}$), 137.6 (1 C, $\mathbf{C_{Ar}}$), 136.1 (2 C, $\mathbf{C_{Ar}}$), 133.0 (1 C, $\mathbf{C_{Ar}}$), 132.0 (1 C, $\mathbf{C_{Ar}}$), 129.5 (2 C, $\mathbf{C_{Ar}}$), 128.6 (2 C, $\mathbf{C_{Ar}}$), 127.0 (1 C, $\mathbf{C_{Ar}}$), 127.0 (1 C, $\mathbf{C_{Ar}}$), 126.5 (1 C, $\mathbf{C_{Ar}}$), 126.4 (1 C, $\mathbf{C_{Ar}}$), 125.5 (2 C, $\mathbf{C_{Ar}}$), 125.1 (2 C, $\mathbf{C_{Ar}}$), 122.6 (2 C, $\mathbf{C_{Ar}}$), 63.0 (1 C, -**C**H₂), 57.5 (1 C, -**C**H), 48.6 (1 C, -**C**H), 34.5 (2 C, -**C**(CH₃)₃), 30.4 (6 C, -C(**C**H₃)₃), 26.2 (3 C, -SiC(**C**H₃)₃), 21.2 (1 C, -**C**H₃), 18.6 (1 C, -Si**C**(CH₃)₃), -5.1 (1 C, -Si**C**H₃), -5.2 (1 C, -Si**C**H₃). *Selected signals for the like diastereomer:* 170.9 (1 C, -**C**OOR), 152.3 (1 C, $\mathbf{C_{Ar}}$), 139.7 (1 C, $\mathbf{C_{Ar}}$), 139.3 (1 C, $\mathbf{C_{Ar}}$), 135.3 (2 C, $\mathbf{C_{Ar}}$), 133.6 (1 C, $\mathbf{C_{Ar}}$), 130.0 (1 C, $\mathbf{C_{Ar}}$), 129.3 (2 C, $\mathbf{C_{Ar}}$), 128.7 (2 C, $\mathbf{C_{Ar}}$), 127.3 (1 C, $\mathbf{C_{Ar}}$), 125.5 (2 C, $\mathbf{C_{Ar}}$), 125.2 (2 C, $\mathbf{C_{Ar}}$), 122.5 (2 C, $\mathbf{C_{Ar}}$), 62.8 (1 C, -**C**H₂), 57.9 (1 C, -**C**H), 50.8 (1 C, -**C**H), 34.2 (2 C, -**C**(CH₃)₃), 30.2 (6 C, -C(**C**H₃)₃), 26.1 (3 C, -SiC(**C**H₃)₃), 21.1 (1 C, -**C**H₃), 18.5 (1 C, -Si**C**(CH₃)₃), -5.2 (1 C, -Si**C**H₃), -5.4 (1 C, -Si**C**H₃).

HRMS (ESI-TOF): *m/z:* $[M+NH_4]^+$ calcd for $C_{43}H_{59}N_2O_6Si^+$: 727.4137, found 727.4140.

Data for the cyclic product 6n:

HPLC (YMC-SB, *n*-hexane/IPA = 10/1, flow = 1.0 mL min^{-1}, T_{Column} = 10 °C, *l* = 240 nm) t_r*(trans):* 12.97 min (major), 27.10 min (minor); t_r*(cis):* 9.69 min (major), 15.90 min (minor).

α_D^{20}(c = 1, $CHCl_3$): -23.6 (mixture of diastereomers).

HRMS (ESI-TOF): *m/z*: $[M+NH_4]^+$ calcd for $C_{31}H_{40}NO_3^+$: 474.3003, found 474.2999.

NMR data for the cis diastereomer:

^{1}H-NMR (500 MHz, $CDCl_3$, 298 K) *δ* / ppm = 7.24-7.20 (m, 3 H, Ar-**H**), 7.07-7.05 (m, 1 H, Ar-**H**), 7.04 (d, *J* = 8.0 Hz, 2 H, Ar-**H**), 6.89 (d, *J* = 8.0 Hz, 2 H, Ar-**H**), 6.57 (s, 2 H, Ar-**H**), 5.90 (d, *J* = 15.1 Hz, 1 H, -C**H₂**), 5.20 (d, *J* = 15.1 Hz, 1 H, -C**H₂**), 5.11 (s, 1 H, -O**H**), 4.91 (d, *J* = 3.2 Hz, 1 H, -C**H**), 4.30 (d, *J* = 3.2 Hz, 1 H, -C**H**), 2.33 (s, 3 H, -C**H₃**), 1.31 (s, 18 H, -C**H₃**).

^{13}C-NMR (125 MHz, $CDCl_3$, 298 K) *δ* / ppm = 172.3 (1 C, -**C**OOR), 153.1 (1 C, $\mathbf{C_{Ar}}$), 141.3 (1 C, $\mathbf{C_{Ar}}$), 137.2 (1 C, $\mathbf{C_{Ar}}$), 135.1 (2 C, $\mathbf{C_{Ar}}$), 133.9 (1 C, $\mathbf{C_{Ar}}$), 132.6 (1 C, $\mathbf{C_{Ar}}$), 132.4 (1 C, $\mathbf{C_{Ar}}$), 131.1 (1 C, $\mathbf{C_{Ar}}$), 130.7 (2 C, $\mathbf{C_{Ar}}$), 129.0 (1 C, $\mathbf{C_{Ar}}$), 128.4 (2 C, $\mathbf{C_{Ar}}$), 128.0 (1 C, $\mathbf{C_{Ar}}$), 127.2 (2 C, $\mathbf{C_{Ar}}$), 126.6 (1 C, $\mathbf{C_{Ar}}$), 70.6 (1 C, -**C**H_2), 55.2 (1 C, -**C**H), 49.9 (1 C, -**C**H), 34.4 (2 C, -**C**$(CH_3)_3$), 30.3 (6 C, -C(**C**$H_3)_3$), 21.2 (1 C, -**C**H_3).

NMR data for the trans diastereomer:

^{1}H-NMR (500 MHz, $CDCl_3$, 298 K) *δ* / ppm = 7.27-7.24 (m, 2 H, Ar-**H**), 7.22-7.19 (m, 1 H, Ar-**H**), 7.13-7.11 (m, 1 H, Ar-**H**), 7.07-7.04 (m, 4 H, Ar-**H**), 6.45 (s, 2 H, Ar-**H**), 5.80 (d, *J* = 13.7 Hz, 1 H, -C**H₂**), 5.04 (d, *J* = 13.7 Hz, 1 H, -C**H₂**), 4.98 (s, 1 H, -O**H**), 4.54 (d, *J* = 10.1 Hz, 1 H, -C**H**), 4.45 (d, *J* = 10.1 Hz, 1 H, -C**H**), 2.30 (s, 3 H, -C**H₃**), 1.22 (s, 18 H, -C**H₃**).

^{13}C-NMR (125 MHz, $CDCl_3$, 298 K) *δ* / ppm = 172.7 (1 C, -**C**OOR), 152.4 (1 C, $\mathbf{C_{Ar}}$), 141.0 (1 C, $\mathbf{C_{Ar}}$), 137.1 (1 C, $\mathbf{C_{Ar}}$), 135.6 (2 C, $\mathbf{C_{Ar}}$), 134.7 (1 C, $\mathbf{C_{Ar}}$), 133.7 (1 C, $\mathbf{C_{Ar}}$), 132.5 (1 C, $\mathbf{C_{Ar}}$), 129.6 (1 C, $\mathbf{C_{Ar}}$), 129.4 (2 C, $\mathbf{C_{Ar}}$), 129.3 (1 C, $\mathbf{C_{Ar}}$), 129.0 (3 C, $\mathbf{C_{Ar}}$), 126.9 (1 C, $\mathbf{C_{Ar}}$), 125.0 (2 C, $\mathbf{C_{Ar}}$), 70.2 (1 C, -**C**H_2), 55.7 (1 C, -**C**H), 54.0 (1 C, -**C**H), 34.3 (2 C, -**C**$(CH_3)_3$), 30.2 (6 C, -C(**C**$H_3)_3$), 21.1 (1 C, -**C**H_3).

3,5-Dimethylphenyl benzo[c]oxepinone derivative (6o)

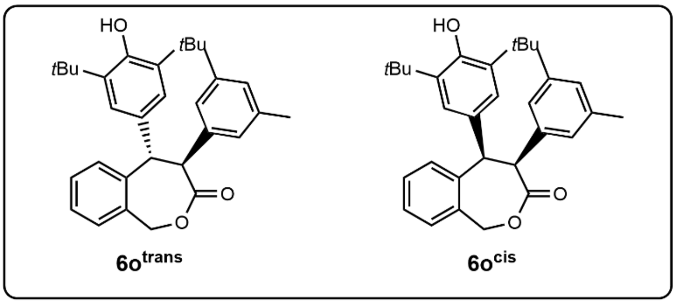

7-membered ring **6o** was synthesized according to general procedure **F** and obtained as a colorless residue in a yield of 22% (24% for the alkylation step) as a mixture of diastereomers (*dr* 45:55; er_{trans} 97:3; er_{cis} 91:9).

Data for the alkylation product (mixture of diastereomers) 5o:

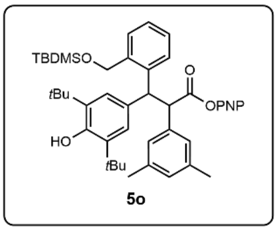

^{1}H-NMR (300 MHz, CDCl$_3$, 298 K) δ / ppm = *Signals for the unlike diastereomer:* 8.11 (d, *J* = 9.1 Hz, 2 H, Ar-**H**), 7.44 (d, *J* = 7.4 Hz, 1 H, Ar-**H**), 7.37 (d, *J* = 7.4 Hz, 1 H, Ar-**H**), 7.24 (s, 2 H, Ar-**H**), 7.20-7.10 (m, 1 H, Ar-**H**), 7.05 (s, 2 H, Ar-**H**), 6.87-6.78 (m, 2 H, Ar-**H**), 6.62 (d, *J* = 9.1 Hz, 2 H, Ar-**H**), 5.16 (s, 1 H, -O**H**), 4.84 (d, *J* = 12.1 Hz, 1 H, -C**H$_2$**), 4.76-4-70 (m, 2 H, -C**H**), 4.68 (d, *J* = 12.1 Hz, 1 H, Ar-**H**), 2.24 (s, 6 H, -C**H$_3$**), 1.40 (s, 18 H, -C(C**H$_3$**)$_3$), 0.96 (s, 9 H, -SiC(C**H$_3$**)$_3$), 0.08 (s, 3 H, -SiC**H$_3$**), 0.07 (s, 3 H, -SiC**H$_3$**). *Selected signals for the like diastereomer:* 8.14 (d, *J* = 9.1 Hz, 2 H, Ar-**H**), 7.73 (d, *J* = 7.2 Hz, 1 H, Ar-**H**), 7.53 (d, *J* = 7.3 Hz, 1 H, Ar-**H**), 7.40-7.33 (m, 2 H, Ar-**H**), 7.20-7.10 (m, 3 H, Ar-**H**), 6.87-6.78 (m, 2 H, Ar-**H**), 6.58 (s, 2 H, Ar-**H**), 4.93 (s, 1 H, -O**H**), 4.91 (d, *J* = 13.5 Hz, 1 H, -C**H**), 4.65 (d, *J* = 11.7 Hz, 1 H, -C**H$_2$**), 4.60 (d, *J* = 13.5 Hz, 1 H, -C**H**), 4.40 (d, *J* = 11.7 Hz, 1 H, -C**H$_2$**), 1.24 s, 18 H, -C(C**H$_3$**)$_3$), 0.88 (s, 9 H, -SiC(C**H$_3$**)$_3$), -0.02 (s, 3 H, -SiC**H$_3$**), -0.05 (s, 3 H, -SiC**H$_3$**).

^{13}C-NMR (75 MHz, CDCl$_3$, 298 K) δ / ppm = *Signals for the unlike diastereomer:* 171.3 (1 C, -**C**OOR), 155.5 (1 C, **C$_{Ar}$**), 152.8 (1 C, **C$_{Ar}$**), 145.4 (1 C, **C$_{Ar}$**), 139.1 (1 C, **C$_{Ar}$**), 138.1 (2 C, **C$_{Ar}$**), 137.6 (1 C, **C$_{Ar}$**), 136.0 (2 C, **C$_{Ar}$**), 135.2 (1 C, **C$_{Ar}$**), 132.0 (1 C, **C$_{Ar}$**), 129.7 (1 C, **C$_{Ar}$**), 126.7 (1 C, **C$_{Ar}$**), 126.6 (1 C, **C$_{Ar}$**), 126.4 (2 C, **C$_{Ar}$**), 126.3 (2 C, **C$_{Ar}$**), 125.5 (2 C, **C$_{Ar}$**), 125.0 (2 C, **C$_{Ar}$**), 122.5 (2 C, **C$_{Ar}$**), 62.7 (1 C, -**C**H$_2$), 57.6 (1 C, -**C**H), 48.7 (1 C, -**C**H), 34.5 (2 C, -**C**(CH$_3$)$_3$), 30.4 (6 C, -C(**C**H$_3$)$_3$), 26.0 (3 C, -SiC(**C**H$_3$)$_3$), 21.3 (2 C, -**C**H$_3$) 18.5 (1 C, -Si**C**(CH$_3$)$_3$), -5.2 (1 C, -Si**C**H$_3$), -5.2 (1 C, -Si**C**H$_3$). *Selected signals for the like diastereomer:* 170.8 (1 C, -**C**OOR), 152.2 (1 C, **C$_{Ar}$**), 139.7 (1 C, **C$_{Ar}$**), 139.4 (1 C, **C$_{Ar}$**), 136.4 (1 C, **C$_{Ar}$**), 135.7 (2 C, **C$_{Ar}$**), 130.2 (1 C, **C$_{Ar}$**), 129.2 (1 C, **C$_{Ar}$**), 127.5 (1 C, **C$_{Ar}$**), 127.0 (1 C, **C$_{Ar}$**), 126.9 (1 C, **C$_{Ar}$**), 125.4 (2 C, **C$_{Ar}$**), 125.1 (2 C, **C$_{Ar}$**), 62.9 (1 C, -**C**H$_2$), 57.9 (1 C, -**C**H), 50.7 (1 C, -**C**H), 34.2 (2 C, -**C**(CH$_3$)$_3$), 30.2 (6 C, -SiC(**C**H$_3$)$_3$), 26.0 (3 C, -SiC(**C**H$_3$)$_3$), 21.3 (2 C, -**C**H$_3$), 18.5 (1 C, -Si**C**(CH$_3$)$_3$), -5.4 (1 C, -Si**C**H$_3$).

HRMS (ESI-TOF): *m/z:* [M+Na]$^+$ calcd for $C_{44}H_{57}NNaO_6Si^+$: 746.3847, found 746.3851.

Data for the cyclic product 6o:

HPLC (YMC-SB, *n*-hexane/IPA = 4/1, flow = 1.0 mL min^{-1}, T_{Column} = 10 °C, *l* = 240 nm) t_r*(trans):* 6.72 min (major), 7.93 min (minor); t_r*(cis):* 7.35 min (major), 32.70 min (minor).

α_D^{20}(c = 1, $CHCl_3$): -39.0 (mixture of diastereomers).

HRMS (ESI-TOF): *m/z*: $[M+NH_4]^+$ calcd for $C_{32}H_{42}NO_3^+$: 488.3159, found 488.3162.

NMR data for the cis diastereomer:

^{1}H-NMR (500 MHz, $CDCl_3$, 298 K) *δ* / ppm = 7.23-7.20 (m, 3 H, Ar-**H**), 7.05-7.03 (m, 1 H, Ar-**H**), 6.91 (s, 1 H, Ar-**H**), 6.69 (s, 2 H, Ar-**H**), 6.65 (s, 2 H, Ar-**H**), 5.89 (d, *J* = 15.2 Hz, 1 H, -C**H₂**), 5.22 (d, *J* = 15.2 Hz, 1 H, -C**H₂**), 5.11 (s, 1 H, -O**H**), 4.87 (d, *J* = 3.1 Hz, 1 H, -C**H**), 4.34 (d, *J* = 3.1 Hz, 1 H, -C**H**), 2.25 (s, 6 H, -**C**H₃), 1.33 (s, 18 H, -C**H₃**).

^{13}C-NMR (125 MHz, $CDCl_3$, 298 K) *δ* / ppm = 172.2 (1 C, -**C**OOR), 153.0 (1 C, $\mathbf{C_{Ar}}$), 141.5 (1 C, $\mathbf{C_{Ar}}$), 137.1 (2 C, $\mathbf{C_{Ar}}$), 136.8 (1 C, $\mathbf{C_{Ar}}$), 135.2 (2 C, $\mathbf{C_{Ar}}$), 132.5 (1 C, $\mathbf{C_{Ar}}$), 132.2 (1 C, $\mathbf{C_{Ar}}$), 131.3 (1 C, $\mathbf{C_{Ar}}$), 129.2 (1 C, $\mathbf{C_{Ar}}$), 129.0 (1 C, $\mathbf{C_{Ar}}$), 128.5 (2 C, $\mathbf{C_{Ar}}$), 128.0 (1 C, $\mathbf{C_{Ar}}$), 127.1 (2 C, $\mathbf{C_{Ar}}$), 126.5 (1 C, $\mathbf{C_{Ar}}$), 70.6 (1 C, -**C**H_2), 54.9 (1 C, -**C**H), 50.2 (1 C, -**C**H), 24.4 (2 C, -**C**$(CH_3)_3$), 30.4 (6 C, -C(**C**$H_3)_3$), 21.6 (2 C, -**C**H_3).

NMR data for the trans diastereomer:

^{1}H-NMR (500 MHz, $CDCl_3$, 298 K) *δ* / ppm = 7.24-7.21 (m, 3 H, Ar-**H**), 7.13 (d, *J* = 7.5 Hz, 1 H, Ar-**H**), 6.85 (s, 1 H, Ar-**H**), 6.77 (s, 2 H, Ar-**H**), 6.48 (s, 2 H, Ar-**H**), 5.76 (d, *J* = 13.7 Hz, 1 H, -C**H₂**), 5.06 (d, *J* = 13.7 Hz, 1 H, -**C**H), 4.98 (s, 1 H, -O**H**), 4.57 (d, *J* = 10.1 Hz, 1 H, -C**H**), 4.42 (d, *J* = 10.1 Hz, 1 H, -C**H**), 2.23 (s, 6 H, -C**H₃**), 1.24 (s, 18 H, -C**H₃**).

^{13}C-NMR (125 MHz, $CDCl_3$, 298 K) *δ* / ppm = 172.7 (1 C, -**C**OOR), 152.4 (1 C, $\mathbf{C_{Ar}}$), 141.0 (1 C, $\mathbf{C_{Ar}}$), 137.7 (2 C, $\mathbf{C_{Ar}}$), 137.5 (1 C, $\mathbf{C_{Ar}}$), 135.6 (2 C, $\mathbf{C_{Ar}}$), 134.6 (1 C, $\mathbf{C_{Ar}}$), 133.7 (1 C, $\mathbf{C_{Ar}}$), 132.4 (1 C, $\mathbf{C_{Ar}}$), 129.6 (1 C, $\mathbf{C_{Ar}}$), 129.3 (1 C, $\mathbf{C_{Ar}}$), 129.1 (1 C, $\mathbf{C_{Ar}}$), 127.3 (2 C, $\mathbf{C_{Ar}}$), 126.9 (1 C, $\mathbf{C_{Ar}}$), 125.0 (2 C, $\mathbf{C_{Ar}}$), 70.2 (1 C, -**C**H_2), 56.0 (1 C, -**C**H), 53.6 (1 C, -**C**H), 34.3 (2 C, -**C**$(CH_3)_3$), 30.2 (6 C, -C(**C**$H_3)_3$), 21.4 (2 C, -**C**H_3).

4-*t*-Butylphenyl benzo[c]oxepinone derivative (6p)

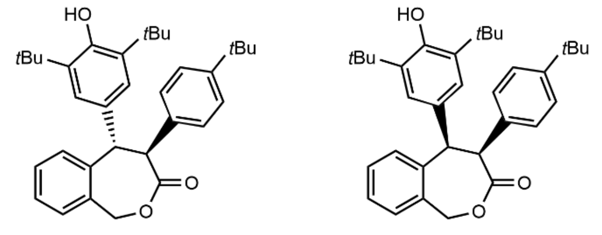

$\mathbf{6p^{trans}}$ $\mathbf{6p^{cis}}$

7-membered ring **6p** was synthesized according to general procedure **F** and obtained as a colorless residue in a yield of 42% (57% for the alkylation step) as a mixture of diastereomers (*dr* 50:50; er_{trans} 95:5; er_{cis} 92:8).

Data for the alkylation product (mixture of diastereomers) 5p:

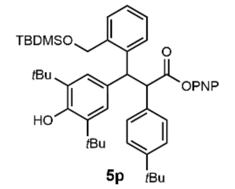

5p

^{1}**H-NMR** (500 MHz, $CDCl_3$, 298 K) δ / ppm = *Signals for the unlike diastereomer:* 8.08 (d, *J* = 9.1 Hz, 2 H, Ar-**H**), 7.44 (d, *J* = 7.3 Hz, 1 H, Ar-**H**), 7.36 (d, *J* = 8.3 Hz, 2 H, Ar-**H**), 7.34-7.32 (m, 1 H, Ar-**H**), 7.23 (d, *J* = 8.3 Hz, 2 H, Ar-**H**), 7.22 (s, 2 H, Ar-**H**), 7.20-7.17 (m, 1 H, Ar-**H**), 7.13-7.10 (m, 1 H, Ar-**H**), 6.58 (d, *J* = 9.1 Hz, 2 H, Ar-**H**), 5.14 (s, 1 H, -O**H**), 4.90 (d, *J* = 12.1 Hz, 1 H, -C**H**$_2$), 4.77 (d, *J* = 13.3 Hz, 1 H, -C**H**), 4.73 (d, *J* = 12.2 Hz, 1 H, -C**H**), 4.63 (d, *J* = 13.3 Hz, 1 H, -C**H**), 1.39 (s, 18 H, -C**H**$_3$), 1.25 (s, 9 H, -C**H**$_3$), 0.95 (s, 9 H, -SiC(C**H**$_3$)$_3$), 0.08 (s, 3 H, -SiC**H**$_3$), 0.06 (s, 3 H, -SiC**H**$_3$). *Selected signals for the like diastereomer:* 8.11 (d, *J* = 9.1 Hz, 2 H, Ar-**H**), 7.72 (d, *J* = 8.2 Hz, 1 H, Ar-**H**), 7.53 (d, *J* = 7.2 Hz, 1 H, Ar-**H**), 7.40-7.32 (m, 2 H, Ar-**H**), 7.29 (d, *J* = 8.3 Hz, 2 H, Ar-**H**), 7.14-7.10 (m, 2 H, Ar-**H**), 6.76 (d, *J* = 9.1 Hz, 2 H, Ar-**H**), 6.53 (s, 2 H, Ar-**H**), 4.92 (s, 1 H, -O**H**), 4.87 (d, *J* = 13.7 Hz, 1 H, -C**H**), 4.61 (d, *J* = 11.8 Hz, 1 H, -C**H**$_2$), 4.56 (d, *J* = 13.7 Hz, 1 H, -C**H**), 4.39 (d, *J* = 11.8 Hz, 1 H, -C**H**), 1.31 (s, 9 H, -C**H**$_3$), 1.20 (18 H, -C**H**$_3$), 0.83 (s, 9 H, -SiC(C**H**$_3$)$_3$), -0.09 (s, 6 H, -SiC**H**$_3$).

^{13}**C-NMR** (75 MHz, $CDCl_3$, 298 K) δ / ppm = *Signals for the unlike diastereomer:* 171.4 (1 C, -**C**OOR), 155.6 (1 C, $\mathbf{C_{Ar}}$), 152.8 (1 C, $\mathbf{C_{Ar}}$), 150.7 (1 C, $\mathbf{C_{Ar}}$), 145.4 (1 C, $\mathbf{C_{Ar}}$), 139.1 (1 C, $\mathbf{C_{Ar}}$), 138.0 (1 C, $\mathbf{C_{Ar}}$), 136.0 (2 C, $\mathbf{C_{Ar}}$), 132.8 (1 C, $\mathbf{C_{Ar}}$), 132.0 (1 C, $\mathbf{C_{Ar}}$), 128.3 (2 C, $\mathbf{C_{Ar}}$), 127.0 (1 C, $\mathbf{C_{Ar}}$), 126.9 (1 C, $\mathbf{C_{Ar}}$), 126.4 (1 C, $\mathbf{C_{Ar}}$), 126.3 (1 C, $\mathbf{C_{Ar}}$), 125.7 (2 C, $\mathbf{C_{Ar}}$), 125.6 (2 C, $\mathbf{C_{Ar}}$), 122.6 (2 C, $\mathbf{C_{Ar}}$), 62.9 (1 C, -**C**H$_2$), 57.3 (1 C, -**C**H), 48.7 (1 C, -**C**H), 34.6 (1 C, -**C**(CH$_3$)$_3$), 34.5 (2 C, -**C**(CH$_3$)$_3$), 31.4 (3 C, -C(**C**H$_3$)$_3$), 30.4 (6 C, -C(**C**H$_3$)$_3$), 26.2 (3 C, -SiC(**C**H$_3$)$_3$), 18.6 (1 C, -Si**C**(CH$_3$)$_3$), -5.1 (1 C, -Si**C**H$_3$), -5.1 (1 C, -Si**C**H$_3$). *Selected signals for the like diastereomer:* 135.2 (2 C, $\mathbf{C_{Ar}}$), 133.5 (1 C, $\mathbf{C_{Ar}}$), 128.6 (2 C, $\mathbf{C_{Ar}}$), 127.4 (1 C, $\mathbf{C_{Ar}}$), 125.6 (2 C, $\mathbf{C_{Ar}}$), 125.5 (2 C, $\mathbf{C_{Ar}}$), 125.2 (2 C, $\mathbf{C_{Ar}}$), 122.6 (2 C, $\mathbf{C_{Ar}}$), 62.8 (1 C, -**C**H$_2$), 58.1 (1 C, -**C**H), 50.9 (1 C, -**C**H), 34.6 (1 C, -**C**(CH$_3$)$_3$), 34.2 (2 C, -**C**(CH$_3$)$_3$), 31.5 (3 C, -C(**C**H$_3$)$_3$), 30.3 (6 C, -C(**C**H$_3$)$_3$), 26.1 (3 C, -SiC(**C**H$_3$)$_3$), 18.5 (1 C, -Si**C**(CH$_3$)$_3$), -5.2 (1 C, -Si**C**H$_3$), -5.4 (1 C, -Si**C**H$_3$).

HRMS (ESI-TOF): *m/z:* $[M+Na]^+$ calcd for $C_{46}H_{61}NNaO_6Si^+$: 769.4606, found 769.4610.

Data for the cyclic product 6p:

HPLC (YMC-SB, *n*-hexane/IPA = 10/1, flow = 1.0 mL min^{-1}, T_{Column} = 10 °C, *l* = 240 nm) t_r*(trans):* 8.65 min (major), 19.42 min (minor); t_r*(cis):* 6.87 min (major), 9.01 min (minor).

α_D^{20}(c = 1, $CHCl_3$): -44.3 (mixture of diastereomers).

HRMS (ESI-TOF): *m/z*: $[M+NH_4]^+$ calcd for $C_{34}H_{46}NO_3^+$: 516.3472, found 516.3466.

Unfortunately, the diastereomers were not separable. NMR data is given for the mixture of diastereomers (obtained from the asymmetric sample, cis:trans = 50:50). Assignment of the signals to the cis/trans diastereomer was not possible unambiguously. Selected multiplets were assigned according to similarity with other entries to the substrate scope.

^{1}H-NMR (300 MHz, $CDCl_3$, 298 K) *δ* / ppm = *Selected signals for the cis diastereomer:* 6.52 (s, 2 H, Ar-**H**), 5.90 (d, *J* = 15.0 Hz, 1 H, -C**H$_2$**), 5.19 (d, *J* = 15.0 Hz, 1 H, -C**H$_2$**), 5.11 (s, 1 H, -O**H**), 4.93 (d, *J* = 3.4 Hz, 1 H, -C**H**), 4.30 (d, *J* = 3.4 Hz, 1 H, -C**H**). *Selected signals for the trans diastereomer:* 6.42 (s, 2 H, Ar-**H**), 5.85 (d, *J* = 13.6 Hz, 1 H, -C**H$_2$**), 5.05 (d, *J* = 13.6 Hz, 1 H, -C**H$_2$**), 4.97 (s, 1 H, -O**H**), 4.55 (d, *J* = 10.2 Hz, 1 H, -C**H**), 4.43 (d, *J* = 10.2 Hz, 1 H, -C**H**). *Signals for both diastereomers:* 7.31-6.91 (m, 16 H, 2 x 8 Ar-**H**), 1.31 (App. s, 27 H, 3 x -C(C**H$_3$**)$_3$), 1.29 (s, 9 H, -C(C**H$_3$**)$_3$), 1.21 (s, 18 H, 2 x -C(C**H$_3$**)$_3$).

^{13}C-NMR (75 MHz, $CDCl_3$, 298 K) *δ* / ppm = *Selected signals for the cis diastereomer:* 172.3 (1 C, -**C**OOR), 70.6 (1 C, -**C**H$_2$), 55.3 (1 C, -**C**H), 49.7 (1 C, -**C**H), 34.6 (1 C, -**C**(CH$_3$)$_3$), 34.4 (3 C, -C(**C**H$_3$)$_3$), 31.5 (2 C, -**C**(CH$_3$)$_3$), 30.3 (6 C, -C(**C**H$_3$)$_3$). *Selected signals for the trans diastereomer:* 172.7 (1 C, -**C**OOR), 70.2 (1 C, -**C**H$_2$), 55.8 (1 C, -**C**H), 54.2 (1 C, -**C**H), 34.6 (1 C, -**C**(CH$_3$)$_3$), 34.3 (3 C, -C(**C**H$_3$)$_3$), 31.5 (2 C, -**C**(CH$_3$)$_3$), 30.2 (6 C, -C(**C**H$_3$)$_3$). *Signals for both diastereomers:* 153.1 (1 C, **C$_{Ar}$**), 152.3 (1 C, **C$_{Ar}$**), 150.3 (1 C, **C$_{Ar}$**), 150.2 (1 C, **C$_{Ar}$**), 141.2 (1 C, **C$_{Ar}$**), 141.1 (1 C, **C$_{Ar}$**), 135.5 (2 C, **C$_{Ar}$**), 135.0 (2 C, **C$_{Ar}$**), 134.7 (1 C, **C$_{Ar}$**), 134.6 (1 C, **C$_{Ar}$**), 133.8 (1 C, **C$_{Ar}$**), 133.7 (1 C, **C$_{Ar}$**), 132.7 (1 C, **C$_{Ar}$**), 132.5 (1 C, **C$_{Ar}$**), 132.4 (1 C, **C$_{Ar}$**), 131.1 (1 C, **C$_{Ar}$**), 130.4 (2 C, **C$_{Ar}$**), 129.7 (1 C, **C$_{Ar}$**), 129.4 (1 C, **C$_{Ar}$**), 129.3 (2 C, **C$_{Ar}$**), 129.0 (1 C, **C$_{Ar}$**), 128.1 (1 C, **C$_{Ar}$**), 127.3 (2 C, **C$_{Ar}$**), 126.9 (1 C, **C$_{Ar}$**), 126.5 (1 C, **C$_{Ar}$**), 125.3 (2 C, **C$_{Ar}$**), 125.0 (2 C, **C$_{Ar}$**), 124.6 (2 C, **C$_{Ar}$**),

***p*-Phenyl benzo[c]oxepinone derivative (6q)**

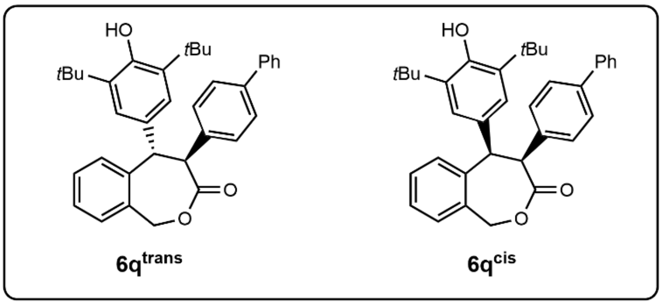

7-membered ring **6q** was synthesized according to general procedure **F** and obtained as a colorless residue in a yield of 54% (58% for the alkylation step) as a mixture of diastereomers (*dr* 55:45; er_{trans} 95:5; er_{cis} 90:10).

Data for the alkylation product (mixture of diastereomers) 5q:

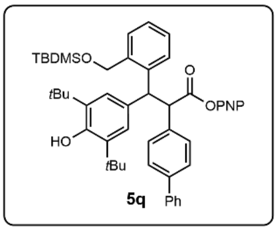

^{1}H-NMR (300 MHz, $CDCl_3$, 298 K) δ / ppm = *Signals for the unlike diastereomer:* 8.12 (d, *J* = 9.1 Hz, 2 H, Ar-**H**), 7.57-7.31 (11 H, Ar-**H**), 7.27 (s, 2 H, Ar-**H**), 7.20 (dt, J_1 = 1.4 Hz, J_2 = 7.4 Hz, 1 H, Ar-**H**), 7.12 (dt, J_1 = 1.0 Hz, J_2 = 7.4 Hz, 1 H, Ar-**H**), 6.63 (d, *J* = 9.1 Hz, 2 H, Ar-**H**), 5.17 (s, 1 H, -O**H**), 5.04 (d, *J* = 12.2 Hz, 1 H, -C**H₂**), 4.85 (d, *J* = 13.2 Hz, 1 H, -C**H**), 4.82 (d, *J* = 12.2 Hz, 1 H, -C**H₂**), 4.66 (d, *J* = 13.2 Hz, 1 H, -C**H**), 1.41 (s, 18 H, -C**H₃**), 0.97 (s, 9 H, -SiC(C**H₃**)₃), 0.11 (s, 3 H, -SiC**H₃**), 0.09 (s, 3 H, -SiC**H₃**). *Selected signals for the like diastereomer:* 8.14 (d, *J* = 9.1 Hz, 2 H, Ar-**H**), 6.81 (d, *J* = 9.1 Hz, 2 H, Ar-**H**), 6.61 (s, 2 H, Ar-**H**), 4.94 (s, 1 H, -O**H**), 4.92 (d, *J* = 13.6 Hz, 1 H, -C**H**), 4.70 (d, *J* = 11.8 Hz, 1 H, -C**H₂**), 4.62 (d, *J* = 13.6 Hz, 1 H, -C**H**), 4.51 (d, *J* = 11.8 Hz, 1 H, -C**H₂**), 1.22 (s, 18 H, -C**H₃**), 0.86 (s, 9 H, -SiC(C**H₃**)₃), -0.05 (s, 3 H, -SiC**H₃**), -0.06 (s, 3 H, -SiC**H₃**).

^{13}C-NMR (75 MHz, $CDCl_3$, 298 K) δ / ppm = *Signals for the unlike diastereomer:* 171.2 (1 C, -**C**OOR), 155.5 (1 C, $\mathbf{C_{Ar}}$), 152.9 (1 C, $\mathbf{C_{Ar}}$), 145.5 (1 C, $\mathbf{C_{Ar}}$), 140.6 (1 C, $\mathbf{C_{Ar}}$), 140.5 (1 C, $\mathbf{C_{Ar}}$), 139.0 (1 C, $\mathbf{C_{Ar}}$), 137.9 (1 C, $\mathbf{C_{Ar}}$), 136.1 (2 C, $\mathbf{C_{Ar}}$), 135.4 (1 C, $\mathbf{C_{Ar}}$), 131.7 (1 C, $\mathbf{C_{Ar}}$), 129.2 (2 C, $\mathbf{C_{Ar}}$), 128.8 (2 C, $\mathbf{C_{Ar}}$), 127.5 (1 C, $\mathbf{C_{Ar}}$), 127.4 (2 C, $\mathbf{C_{Ar}}$), 127.2 (1 C, $\mathbf{C_{Ar}}$), 127.2 (1 C, $\mathbf{C_{Ar}}$), 127.1 (2 C, $\mathbf{C_{Ar}}$), 126.6 (1 C, $\mathbf{C_{Ar}}$), 126.3 (1 C, $\mathbf{C_{Ar}}$), 125.5 (2 C, $\mathbf{C_{Ar}}$), 125.0 (2 C, $\mathbf{C_{Ar}}$), 122.5 (2 C, $\mathbf{C_{Ar}}$), 63.1 (1 C, -**C**H₂), 57.5 (1 C, -**C**H), 48.6 (1 C, -**C**H), 34.5 (2 C, -**C**(CH₃)₃), 30.4 (6 C, -C(**C**H₃)₃), 26.1 (3 C, -SiC(**C**H₃)₃), 18.6 (1 C, -Si**C**(CH₃)₃), -5.1 (1 C, -Si**C**H₃), -5.2 (1 C, -Si**C**H₃). *Selected signals for the like diastereomer:* 170.7 (1 C, -**C**OOR), 152.3 (1 C, $\mathbf{C_{Ar}}$), 140.9 (1 C, $\mathbf{C_{Ar}}$), 140.8 (1 C, $\mathbf{C_{Ar}}$), 139.7 (1 C, $\mathbf{C_{Ar}}$), 139.2 (1 C, $\mathbf{C_{Ar}}$), 135.7 (1 C, $\mathbf{C_{Ar}}$), 135.4 (2 C, $\mathbf{C_{Ar}}$), 129.3 (2 C, $\mathbf{C_{Ar}}$), 128.9 (2 C, $\mathbf{C_{Ar}}$), 125.5 (2 C, $\mathbf{C_{Ar}}$), 125.2 (2 C, $\mathbf{C_{Ar}}$), 62.8 (1 C, 1 C -**C**H₂), 58.0 (1 C, -**C**H), 50.8 (1 C, -**C**H), 34.2 (2 C, -**C**(CH₃)₃), 30.2 (6 C, -C(**C**H₃)₃), 26.0 (3 C, -SiC(**C**H₃)₃), 18.5 (1 C, -Si**C**(CH₃)₃), -5.2 (s, 1 C, -Si**C**H₃), -5.4 (1 C, -Si**C**H₃).

HRMS (ESI-TOF): *m/z:* $[M+NH_4]^+$ calcd for $C_{48}H_{61}N_2O_6Si^+$: 789.4293, found 789.4299.

Data for the cyclic product 6q:

HPLC (YMC-SB, *n*-hexane/IPA = 4/1, flow = 1.0 mL min^{-1}, T_{Column} = 10 °C, *l* = 240 nm)) t_r*(trans):* 11.65 min (major), 16.80 min (minor); t_r*(cis):* 14.14 min (major), 18.48 min (minor).

α_D^{20}(c = 1, $CHCl_3$): -23.6 (mixture of diastereomers).

HRMS (ESI-TOF): *m/z*: $[M+NH_4]^+$ calcd for $C_{34}H_{40}NO_3^+$: 536.3159, found 536.3155.

NMR data for the cis diastereomer:

^{1}H-NMR (500 MHz, $CDCl_3$, 298 K) *δ* / ppm = 7.59-7.57 (m, 2 H, Ar-**H**), 7.48-7.43 (m, 4 H, Ar-**H**), 7.37-7.35 (m, 1 H, Ar-**H**), 7.26-7.24 (m, 3 H, Ar-**H**), 7.10-7.09 (m, 3 H, Ar-**H**), 6.62 (s, 2 H, Ar-**H**), 5.94 (d, *J* = 15.2 Hz, 1 H, -C**H$_2$**), 5.23 (d, *J* = 15.2 Hz, 1 H, -C**H$_2$**), 5.14 (s, 1 H, -O**H**), 5.00 (d, *J* = 3.3 Hz, 1 H, -C**H**), 4.37 (d, *J* = 3.3 Hz, 1 H, -C**H**), 1.33 (s, 18 H, -C**H$_3$**).

^{13}C-NMR (125 MHz, $CDCl_3$, 298 K) *δ* / ppm = 172.2 (1 C, -**C**OOR), 153.2 (1 C, **C$_{Ar}$**), 141.2 (1 C, **C$_{Ar}$**), 141.1 (1 C, **C$_{Ar}$**), 140.4 (1 C, **C$_{Ar}$**), 135.9 (1 C, **C$_{Ar}$**), 135.2 (2 C, **C$_{Ar}$**), 132.6 (1 C, **C$_{Ar}$**), 132.4 (2 C, **C$_{Ar}$**), 131.2 (2 C, **C$_{Ar}$**), 130.9 (1 C, **C$_{Ar}$**), 129.0 (1 C, **C$_{Ar}$**), 128.9 (2 C, **C$_{Ar}$**), 128.1 (1 C, **C$_{Ar}$**), 127.4 (1 C, **C$_{Ar}$**), 127.2 (3 C, **C$_{Ar}$**), 126.6 (1 C, **C$_{Ar}$**), 126.4 (2 C, **C$_{Ar}$**), 70.7 (1 C, -**C**H_2), 55.2 (1 C, -**C**H), 50.0 (1 Cm -**C**H), 34.4 (2 C, -**C**$(CH_3)_3$), 30.3 (6 C, -C(**C**$H_3)_3$).

NMR data for the trans diastereomer:

^{1}H-NMR (500 MHz, $CDCl_3$, 298 K) *δ* / ppm = 7.55-7.53 (m, 2 H, Ar-**H**), 7.48 (d, *J* = 8.3 Hz, 2 H, Ar-**H**), 7.45-7.42 (m, 2 H, Ar-**H**), 7.36-7.32 (m, 1 H, Ar-**H**), 7.29-7.28 (m, 2 H, Ar-**H**), 7.25-7.21 (m, 3 H, Ar-**H**), 7.15-7.13 (m, 1 H, Ar-**H**), 6.48 (s, 2 H, Ar-**H**), 5.90 (d, *J* = 13.7 Hz, 1 H, -C**H$_2$**), 5.08 (d, *J* = 13.7 Hz, 1 H, -C**H**), 4.99 (s, 1 H, -O**H**), 4.60 (d, *J* = 10.4 Hz, 1 H, -C**H**), 4.54 (d, *J* = 10.4 Hz, 1 H, -C**H**), 1.21 (s, 18 H, -C**H$_3$**).

^{13}C-NMR (125 MHz, $CDCl_3$, 298 K) *δ* / ppm = 172.5 (1 C, -**C**OOR), 152.5 (1 C, **C$_{Ar}$**), 141.1 (1 C, **C$_{Ar}$**), 140.9 (1 C, **C$_{Ar}$**), 140.5 (1 C, **C$_{Ar}$**), 136.8 (1 C, **C$_{Ar}$**), 135.7 (2 C, **C$_{Ar}$**), 134.5 (1 C, **C$_{Ar}$**), 133.6 (1 C, **C$_{Ar}$**), 132.5 (1 C, **C$_{Ar}$**), 130.1 (2 C, **C$_{Ar}$**), 129.7 (1 C, **C$_{Ar}$**), 129.5 (1 C, **C$_{Ar}$**), 128.9 (2 C, **C$_{Ar}$**),127.4 (1 C, **C$_{Ar}$**), 127.3 (2 C, **C$_{Ar}$**), 127.1 (2 C, **C$_{Ar}$**), 127.0 (1 C, **C$_{Ar}$**), 125.0 (2 C, **C$_{Ar}$**), 70.3 (1 C, -**C**H_2), 55.7 (1 C, -**C**H), 54.2 (1 C, -**C**H), 34.3 (2 C, -**C**$(CH_3)_3$), 30.2 (6 C, -C(**C**$H_3)_3$).

3-Trifluoromethylphenyl benzo[c]oxepinone derivative (6r)

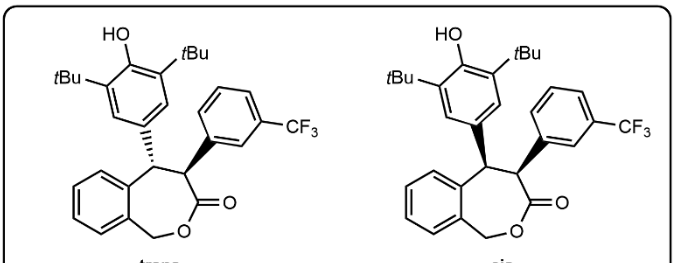

6r^{trans} **6r**^{cis}

7-membered ring **6r** was synthesized according to general procedure **F** and obtained as a colorless residue in a yield of 62% (67% for the alkylation step) as a mixture of diastereomers (*dr* 50:50; er_{trans} 88:12; er_{cis} 82:18).

Data for the alkylation product (mixture of diastereomers) 5r:

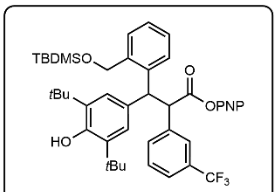

5r

^{1}H-NMR (300 MHz, $CDCl_3$, 298 K) *δ* / ppm = *Signals for the unlike diastereomer:* 8.06 (d, *J* = 9.1 Hz, 2 H, Ar-**H**), 7.71-7.63 (m, 1 H, Ar-**H**), 7.49-7.24 (m, 5 H, Ar-**H**), 7.20 (s, 2 H, Ar-**H**), 7.16-7.04 (m, 2 H, Ar-**H**), 6.56 (d, *J* = 9.1 Hz, 2 H, Ar-**H**), 5.13 (s, 1 H, -O**H**), 4.92 (d, *J* = 12.1 Hz, 1 H, -C**H$_2$**), 4.77 (d, *J* = 12.1 Hz, 1 H, -C**H$_2$**), 4.69 (d, *J* = 13.1 Hz, 1 H, -C**H**), 4.57 (d, *J* = 13.1 Hz, 1 H, -C**H**), 1.35 (s, 18 H, -C**H$_3$**), 0.90 (s, 9 H, -SiC(C**H$_3$**)$_3$), 0.05 (s, 3 H, -SiC**H$_3$**), 0.03 (s, 3 H, -SiC**H$_3$**). *Selected signals for the like diastereomer:* 8.08 (d, *J* = 9.1 Hz, 2 H, Ar-**H**), 7.71-7.63 (m, 3 H, Ar-**H**), 7.49-7.24 (m, 5 H, Ar-**H**), 6.73 (d, *J* = 9.1 Hz, 2 H, Ar-**H**), 6.53 (s, 2 H, -Ar-**H**), 4.91 (s, 1 H, -O**H**), 4.85 (d, *J* = 13.4 Hz, 1 H, -C**H**), 4.63 (d, *J* = 11.9 Hz, 1 H, -C**H$_2$**), 4.59-4.54 (m, 1 H, -C**H**), 4.52 (d, *J* = 11.9 Hz, 1 H, -**C**H$_2$), 1.17 (s, 18 H, -C**H$_3$**), 0.81 (s, 9 H, -SiC(C**H$_3$**)$_3$), -0.07 (s, 3 H, -SiC**H$_3$**), -0.10 (s, 3 H, -SiC**H$_3$**).

^{19}F-NMR (282 MHz, $CDCl_3$, 298 K) *δ* / ppm = -62.6 (3 F, -C**F$_3$**, *both diastereomers*).

^{13}C-NMR (75 MHz, $CDCl_3$, 298 K) *δ* / ppm = *Signals for the unlike diastereomer:* 170.7 (1 C, -**C**OOR), 155.2 (1 C, **C$_{Ar}$**), 153.0 (1 C, **C$_{Ar}$**), 145.6 (1 C, **C$_{Ar}$**), 139.0 (1 C, **C$_{Ar}$**), 137.3 (1 C, **C$_{Ar}$**), 137.1 (1 C, **C$_{Ar}$**), 136.2 (2 C, **C$_{Ar}$**), 132.0 (1 C, **C$_{Ar}$**), 131.2 (1 C, **C$_{Ar}$**), 129.3 (1 C, **C$_{Ar}$**), 127.4 (1 C, **C$_{Ar}$**), 127.1 (1 C, **C$_{Ar}$**), 126.8 (1 C, **C$_{Ar}$**), 126.2 (1 C, **C$_{Ar}$**), 125.8-125.6 (m, 1 C, **C$_{Ar}$**), 125.5 (2 C, **C$_{Ar}$**), 125.2 (1 C, **C$_{Ar}$**), 125.1 (2 C, **C$_{Ar}$**), 124.8-124.7 (1 C, **C$_{Ar}$**), 122.4 (2 C, **C$_{Ar}$**), 63.0 (1 C, -**C**H$_2$), 57.4 (1 C, -**C**H), 48.9 (1 C, -**C**H), 34.5 (2 C, -**C**(CH$_3$)$_3$), 30.4 (6 C, -C(**C**H$_3$)$_3$), 26.1 (3 C, -SiC(**C**H$_3$)$_3$), 18.6 (1 C, -Si**C**(CH$_3$)$_3$), -5.2 (1 C, -Si**C**H$_3$), -5.3 (1 C, -Si**C**H$_3$). *Selected signals for the like diastereomer:* 170.2 (1 C, -**C**OOR), 155.2 (1 C, **C$_{Ar}$**), 152.5 (1 C, **C$_{Ar}$**), 139.7 (1 C, **C$_{Ar}$**), 138.8 (1 C, **C$_{Ar}$**), 137.8 (1 C, **C$_{Ar}$**), 135.8 (2 C, **C$_{Ar}$**), 131.7 (1 C, **C$_{Ar}$**), 129.4 (1 C, **C$_{Ar}$**), 129.1 (1 C, **C$_{Ar}$**), 127.8 (1 C, **C$_{Ar}$**), 127.2 (1 C, **C$_{Ar}$**), 127.1 (1 C, **C$_{Ar}$**), 126.0-125.9 (m, 1 C, **C$_{Ar}$**), 124.5-124.4 (m, 1 C, **C$_{Ar}$**), 62.9 (1 C, -**C**H$_2$), 57.9 (1 C, -**C**H), 51.1 (1 C, -**C**H), 34.2 (2 C, -**C**(CH$_3$)$_3$), 30.1 (6 C, -C(**C**H$_3$)$_3$), 26.0 (3 C, -SiC(**C**H$_3$)$_3$), 18.5 (1 C, -Si**C**(CH$_3$)$_3$), -5.2 (1 C, -Si**C**H$_3$), -5.4 (1 C, -Si**C**H$_3$).

HRMS (ESI-TOF): *m/z:* $[M+Na]^+$ calcd for $C_{43}H_{52}F_3NNaO_6Si^+$: 786.3408, found 786.3411.

Data for the cyclic product 6r:

HPLC (YMC-SB, *n*-hexane/IPA = 4/1, flow = 1.0 mL min^{-1}, T_{Column} = 10 °C, *l* = 240 nm) t_r*(trans):* 7.30 min (major), 12.21 min (minor); t_r*(cis):* 9.38 min (major), 16.63 min (minor).

α_D^{20}(c = 1, $CHCl_3$): -37.1 (mixture of diastereomers).

HRMS (ESI-TOF): *m/z*: $[M+NH_4]^+$ calcd for $C_{31}H_{37}F_3NO_3^+$: 528.2720, found 528.2724.

Unfortunately, the diastereomers were not separable. NMR data is given for the mixture of diastereomers (obtained from the racemic sample, cis:trans = 70:30):

^{1}H-NMR (500 MHz, $CDCl_3$, 298 K) *δ* / ppm = *Signals for the cis diastereomer:* 6.57 (s, 2 H, Ar-**H**), 5.92 (d, *J* = 15.1 Hz, 1 H, -C**H₂**), 5.23 (d, *J* = 15.1 Hz, 1 H, -C**H₂**), 5.16 (s, 1 H, -O**H**), 5.02 (d, *J* = 3.3 Hz, 1 H, -C**H**), 4.31 (d, *J* = 3.3 Hz, 1 H, -C**H**), 1.31 (s, 18 H, -C**H₃**). *Signals for the trans diastereomer:* 6.42 (s, 2 H, Ar-**H**), 6.01 (d, *J* = 13.8 Hz, 1 H, -C**H₂**), 5.09 (d, *J* = 13.8 Hz, 1 H, -C**H₂**), 5.00 (s, 1 H, -O**H**), 4.65 (d, *J* = 11.3 Hz, 1 H, -C**H**), 4.48 (d, *J* = 11.3 Hz, 1 H, -C**H**), 1.21 (s, 18 H, -C**H₃**, *trans*). *Signals for both diastereomers:* 7.54-7.05 (m, 2 x 8 H, Ar-**H**, *cis* + *trans*).

^{13}C-NMR (125 MHz, $CDCl_3$, 298 K) *δ* / ppm =

Signals for the cis diastereomer: 171.7 (1 C, -**C**OOR), 153.4 (1 C, **C_{Ar}**), 140.8 (1 C, **C_{Ar}**), 137.8 (1 C, **C_{Ar}**), 135.6 (2 C, **C_{Ar}**), 134.7 (1 C, **C_{Ar}**), 132.3 (1 C, **C_{Ar}**), 132.3 (1 C, **C_{Ar}**), 132.5 (1 C, **C_{Ar}**), 130.5 (1 C, **C_{Ar}**), 129.8 (1 C, **C_{Ar}**), 129.1 (1 C, **C_{Ar}**), 128.2 (1 C, **C_{Ar}**), 128.0 (1 C, **C_{Ar}**), 127.0 (2 C, **C_{Ar}**), 124.8 (1 C, **C_{Ar}**), 124.5 (1 C, **C_{Ar}**), 70.7 (1 C, -**C**H_2), 54.9 (1 C, -**C**H), 50.0 (1 C, -**C**H), 34.4 (2 C, -**C**$(CH_3)_3$), 30.3 (6 C, -C(**C**$H_3)_3$). *Signals for the trans diastereomer:* 172.0 (1 C, -**C**OOR), 152.6 (1 C, **C_{Ar}**), 140.6 (1 C, **C_{Ar}**), 138.9 (1 C, **C_{Ar}**), 136.0 (2 C, **C_{Ar}**), 134.1 (1 C, **C_{Ar}**), 133.4 (1 C, **C_{Ar}**), 133.1 (1 C, **C_{Ar}**), 130.3 (1 C, **C_{Ar}**), 130.0 (1 C, **C_{Ar}**), 129.7 (1 C, **C_{Ar}**), 128.6 (1 C, **C_{Ar}**), 127.3 (1 C, **C_{Ar}**), 127.0 (1 C, **C_{Ar}**), 126.8 (2 C, **C_{Ar}**), 125.3 (1 C, **C_{Ar}**), 124.2 (1 C, **C_{Ar}**), 70.4 (1 C, -**C**H_2), 55.0 (1 C, -**C**H), 54.7 (1 C, -**C**H), 34.2 (2 C, -**C**$(CH_3)_3$), 30.1 (6 C, -C(**C**$H_3)_3$).

4-Trifluoromethylphenyl benzo[c]oxepinone derivative (6s)

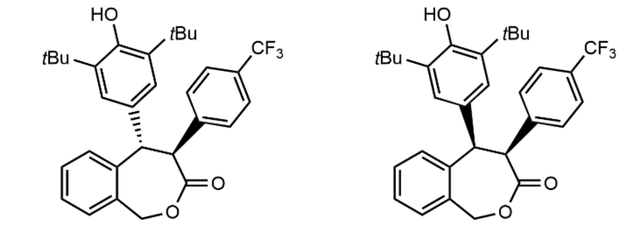

$6s^{trans}$ $6s^{cis}$

7-membered ring **6s** was synthesized according to general procedure **F** and obtained as a colorless residue in a yield of 39% (43% for the alkylation step) as a mixture of diastereomers (*dr* 50:50; er_{trans} 88:12; er_{cis} 80:20).

Data for the alkylation product (mixture of diastereomers) 5s:

This alkylation product could never be isolated in pure form and the NMR spectra still contain impurities. Interestingly enough, when using Okamoto's catalyst, the trans isomer was isolated as mayor product (usually Okamoto's catalyst gives the cis).

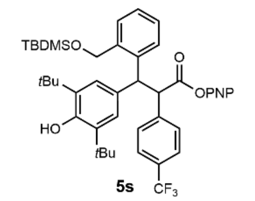

^{1}H-NMR (300 MHz, $CDCl_3$, 298 K) δ / ppm = *Selected Signals for the unlike diastereomer:* 8.10 (d, J = 9.1 Hz, 2 H, Ar-**H**), 7.23 (s, 2 H, Ar-**H**), 6.60 (d, J = 9.1 Hz, 2 H, Ar-**H**), 5.18 (s, 1 H, -O**H**), 5.06 (d, J = 12.2 Hz, 1 H, -C**H$_2$**), 1.39 (s, 18 H, -C**H$_3$**), 0.92 (s, 9 H, -SiC(C**H$_3$**)$_3$), 0.11 (s, 3 H, -SiC**H$_3$**), 0.09 (s, 3 H, -SiC**H$_3$**). *Selected signals for the like diastereomer:* 8.13 (d, J = 9.1 Hz, 2 H, Ar-**H**), 7.73 (d, J = 6.8 Hz, 1 H, Ar-**H**), 7.63 (d, J = 8.2 Hz, 1 H, Ar-**H**), 6.78 (d, 9.1 Hz, 2 H, Ar-**H**), 6.53 (s, 2 H, Ar-**H**), 4.97 (s, 1 H, -O**H**), 1.21 (s, 18 H, -C**H$_3$**), 0.85 (9 H, -SiC(C**H$_3$**)$_3$), -0.06 (s, 3 H, -SiC**H$_3$**), -0.07 (s, 3 H, -SiC**H$_3$**). *Signals for both diastereomers:* 7.54-7.04 (m, *cis*: 8 x Ar-**H**; *trans* 6 x Ar-**H**), 4.89-4.75 (m, *cis:* 1 x -C**H$_2$**, 1 x -C**H**; *trans*: 1 x -C**H**), 4.67-4.51 (m, *cis:* 1 x -C**H**; *trans*: 2 x -C**H$_2$**, 1 x -C**H**).

^{19}F-NMR (282 MHz, $CDCl_3$, 298 K) δ / ppm = -62.6 (3 F, -C**F$_3$**, *both diastereomers*).

^{13}C-NMR (75 MHz, $CDCl_3$, 298 K) δ / ppm = *Selected Signals for the unlike diastereomer:* 170.7 (1 C, -**C**OOR), 63.6 (1 C, -**C**H$_2$), 57.5 (1 C, -**C**H), 48.5 (1 C, -**C**H), 34.5 (2 C, -**C**(CH$_3$)$_3$), 30.4 (6 C, -C(**C**H$_3$)$_3$), 26.1 (3 C, -SiC(**C**H$_3$)$_3$), 18.6 (1 C, -Si**C**(CH$_3$)$_3$), -5.1 (1 C, -Si**C**H$_3$), -5.2 (1 C, -Si**C**H$_3$). *Selected signals for the like diastereomer:* 170.2 (1 C, -**C**OOR), 155.2 (1 C, **C$_{Ar}$**), 152.5 (1 C, **C$_{Ar}$**), 149.3 (1 C, **C$_{Ar}$**), 147.9 (1 C, **C$_{Ar}$**), 145.6 (1 C, **C$_{Ar}$**), 140.8 (1 C, **C$_{Ar}$**), 139.7 (1 C, **C$_{Ar}$**), 138.7 (1 C, **C$_{Ar}$**), 135.7 (2 C, **C$_{Ar}$**), 131.0 (1 C, **C$_{Ar}$**), 129.3 (2 C, **C$_{Ar}$**), 127.7 (1 C, **C$_{Ar}$**), 127.3 (1 C, **C$_{Ar}$**), 127.0 (1 C, **C$_{Ar}$**), 125.5 (2 C, **C$_{Ar}$**), 125.3 (2 C, **C$_{Ar}$**), 125.3 (2 C, **C$_{Ar}$**), 122.5 (2 C, **C$_{Ar}$**), 62.8 (1 C, -**C**H$_2$), 58.0 (1 C, -**C**H), 51.1 (1 C, -**C**H), 34.2 (2 C, -**C**(CH$_3$)$_3$), 30.1 (6 C, -C(**C**H$_3$)$_3$), 26.0 (3 C, -SiC(**C**H$_3$)$_3$), 18.5 (1 C, -Si**C**(CH$_3$)$_3$), -5.2 (1 C, -Si**C**H$_3$), -5.4 (1 C, -Si**C**H$_3$).

HRMS (ESI-TOF): *m/z:* $[M+Na]^+$ calcd for $C_{43}H_{52}F_3NNaO_6Si^+$: 786.3408, found 786.3406.

Data for the cyclic product 6s:

HPLC (YMC-SB, *n*-hexane/IPA = 10/1, flow = 1.0 mL min^{-1}, T_{Column} = 10 °C, *l* = 240 nm) t_r*(trans):* 12.21 min (major), 23.58 min (minor); t_r*(cis):* 11.78 min (major), 27.47 min (minor).

α_D^{20}(c = 1, $CHCl_3$): -18.5 (mixture of diastereomers).

HRMS (ESI-TOF): *m/z*: $[M+NH_4]^+$ calcd for $C_{31}H_{37}F_3NO_3^+$: 528.2720, found 528.2727.

NMR data for the cis diastereomer:

^{1}H-NMR (500 MHz, $CDCl_3$, 298 K) *δ* / ppm = 7.49 (d, *J* = 8.2 Hz, 2 H, Ar-**H**), 7.25-7.22 (m, 3 H, Ar-**H**), 7.13 (d, *J* = 8.2 Hz, 2 H, Ar-**H**), 7.07-7.05 (m, 1 H, Ar-**H**), 6.51 (s, 2 H, Ar-**H**), 5.92 (d, *J* = 15.1 Hz, 1 H, -C**H$_2$**), 5.23 (d, *J* = 15.1 Hz, 1 H, -C**H$_2$**), 5.16 (s, 1 H, -O**H**), 5.02 (d, *J* = 3.6 Hz, 1 H, -C**H**), 4.28 (d, *J* = 3.6 Hz, 1 H, -C**H**), 1.30 (s, 18 H, -C**H$_3$**).

^{19}F-NMR (470 MHz, $CDCl_3$, 298 K) *δ* / ppm = -62.7 (3 F, -C**F$_3$**).

^{13}C-NMR (125 MHz, $CDCl_3$, 298 K) *δ* / ppm = 171.5 (1 C, -**C**OOR), 153.3 (1 C, **C$_{Ar}$**), 140.8 (1 C, **C$_{Ar}$**), 140.7 (1 C, **C$_{Ar}$**), 135.4 (2 C, **C$_{Ar}$**), 132.3 (1 C, **C$_{Ar}$**), 132.3 (1 C, **C$_{Ar}$**), 131.2 (2 C, **C$_{Ar}$**), 130.4 (1 C, **C$_{Ar}$**), 129.6 (1 C, **C$_{Ar}$**), 129.1 (1 C, **C$_{Ar}$**), 128.2 (1 C, **C$_{Ar}$**), 127.0 (2 C, **C$_{Ar}$**), 126.8 (1 C, **C$_{Ar}$**), 124.5 (1 C, **C$_{Ar}$**), 124.5 (1 C, **C$_{Ar}$**), 70.7 (1 C, -**C**H$_2$), 55.0 (1 C, -**C**H), 50.0 (1 C, -**C**H), 34.3 (2 C, -**C**(CH_3)$_3$), 30.2 (6 C, -C(**C**H$_3$)$_3$).

NMR data for the trans diastereomer:

^{1}H-NMR (500 MHz, $CDCl_3$, 298 K) *δ* / ppm = 7.52 (d, *J* = 8.1 Hz, 2 H, Ar-**H**), 7.30-7.21 (m, 5 H, Ar-**H**), 7.09 (d, *J* = 8.0 Hz, 1 H, Ar-**H**), 6.39 (s, 2 H, Ar-**H**), 5.98 (d, *J* = 13.7 Hz, 1 H, -C**H$_2$**), 5.08 (d, *J* = 13.7 Hz, 1 H, -C**H$_2$**), 5.01 (s, 2 H, -O**H**), 4.59 (d, *J* = 11.0 Hz, 1 H, -C**H**), 4.50 (d, *J* = 11.0 Hz, 1 H, -C**H**), 1.20 (s, 18 H, -C**H$_3$**).

^{19}F-NMR (470 MHz, $CDCl_3$, 298 K) *δ* / ppm = -62.6 (3 F, -C**F$_3$**).

^{13}C-NMR (125 MHz, $CDCl_3$, 298 K) *δ* / ppm = 171.8 (1 C, -**C**OOR), 152.6 (1 C, **C$_{Ar}$**), 142.0 (1 C, **C$_{Ar}$**), 140.6 (1 C, **C$_{Ar}$**), 135.9 (2 C, **C$_{Ar}$**), 134.1 (1 C, **C$_{Ar}$**), 133.2 (1 C, **C$_{Ar}$**), 132.5 (2 C, **C$_{Ar}$**), 130.5 (2 C, **C$_{Ar}$**), 129.8 (1 C, **C$_{Ar}$**), 129.6 (1 C, **C$_{Ar}$**), 127.1 (1 C, **C$_{Ar}$**), 125.1 (1 C, **C$_{Ar}$**), 125.0 (1 C, **C$_{Ar}$**), 124.9 (2 C, **C$_{Ar}$**), 70.4 (1 C, -**C**H$_2$), 55.4 (1 C, -**C**H), 54.7 (1 C, -**C**H), 34.2 (2 C, -**C**(CH_3)$_3$), 30.1 (6 C, -C(**C**H$_3$)$_3$).

4-Nitrophenyl benzo[c]oxepinone derivative (6t)

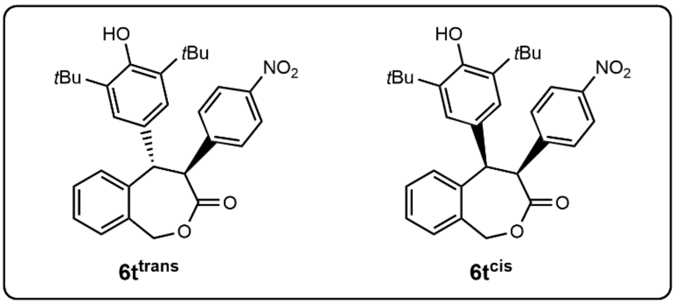

7-membered ring **6t** was synthesized according to general procedure **F** and obtained as a colorless residue in a yield of 40% (46% for the alkylation step) as a mixture of diastereomers (*dr* 60:40; er_{trans} 53:47; er_{cis} 55:45).

Data for the alkylation product (mixture of diastereomers) 5t:

This alkylation product could never be isolated in pure form and the NMR spectra still contain impurities. Interestingly enough, when using Okamoto's catalyst, the trans isomer was isolated as mayor product (usually Okamoto's catalyst gives the cis).

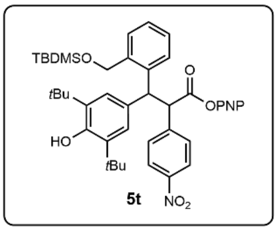

^{1}H-NMR (500 MHz, $CDCl_3$, 298 K) δ / ppm = *Selected signals for the unlike diastereomer:* 8.11 (d, *J* = 9.1 Hz, 2 H, Ar-**H**), 8.10 (d, *J* = 9.1 Hz, 2 H, Ar-**H**), 7.72 (d, *J* = 9.1 Hz, 2 H, Ar-**H**), 7.43-7.34 (m, 3 H, Ar-**H**), 7.24 (s, 2 H, Ar-**H**), 7.13-7.10 (m, 1 H, Ar-**H**), 6.60 (d, *J* = 9.1 Hz, 1 H, Ar-**H**), 5.21 (s, 1 H, -O**H**), 5.14 (d, *J* = 12.2 Hz, 1 H, -C**H₂**), 4.89 (d, *J* = 12.2 Hz, 1 H, -C**H₂**), 4.85 (d, *J* = 12.8 Hz, 1 H, -C**H**), 4.55 (d, *J* = 12.8 Hz, 1 H, -C**H**), 1.39 (s, 18 H, -C**H₃**), 0.97 (s, 9 H, -SiC(C**H₃**)₃), 0.14 (s, 3 H, -SiC**H₃**), 0.11 (s, 3 H, -SiC**H₃**). *Signals for the like diastereomer:* 8.24 (d, *J* = 9.1 Hz, 2 H, Ar-**H**), 8.13 (d, *J* = 9.1 Hz, 2 H, Ar-**H**), 7.74-7.72 (m, 1 H, Ar-**H**), 7.54 (d, *J* = 7.5 Hz, 1 H, Ar-**H**), 7.43-7.34 (m, 1 H, Ar-**H**), 7.40 (d, *J* = 9.1 Hz, 2 H, Ar-**H**), 7.18 (t, *J* = 8.0 Hz, 1 H, Ar-**H**), 6.76 (d, *J* = 9.1 Hz, 2 H, Ar-**H**), 6.58 (s, 2 H, Ar-**H**), 5.00 (s, 2 H, Ar-**H**), 4.88 (d, *J* = 13.5 Hz, 1 H, -C**H**), 4.70 (d, *J* = 11.9 Hz, 1 H, -C**H₂**), 4.63 (d, *J* = 11.9 Hz, -C**H₂**), 4.59 (d, *J* = 13.5 Hz, 1 H, -C**H**), 1.21 (s, 18 H, -C**H₃**), 0.86 (s, 9 H, -SiC(C**H₃**)₃), -0.04 (s, 3 H, -SiC**H₃**), -0.06 (s, 3 H, -SiC**H₃**).

^{13}C-NMR (125 MHz, $CDCl_3$, 298 K) δ / ppm = *Selected signals for the unlike diastereomer:* 170.2 (1 C, -**C**OOR), 153.1 (1 C, $\mathbf{C_{Ar}}$), 143.4 (1 C, $\mathbf{C_{Ar}}$), 136.2 (2 C, $\mathbf{C_{Ar}}$), 130.8 (1 C, $\mathbf{C_{Ar}}$), 129.9 (2 C, $\mathbf{C_{Ar}}$), 128.3 (1 C, $\mathbf{C_{Ar}}$), 127.4 (1 C, $\mathbf{C_{Ar}}$), 127.0 (1 C, $\mathbf{C_{Ar}}$), 126.1 (1 C, $\mathbf{C_{Ar}}$), 125.4 (2 C, $\mathbf{C_{Ar}}$), 123.9 (2 C, $\mathbf{C_{Ar}}$), 122.4 (2 C, $\mathbf{C_{Ar}}$), 63.5 (1 C, -**C**H₂), 57.5 (1 C, -**C**H), 48.6 (1 C, -**C**H), 34.5 (2 C, -**C**(CH₃)₃), 30.3 (6 C, -C(**C**H₃)₃), 26.1 (3 C, -SiC(**C**H₃)₃), 18.6 (1 C, -Si**C**(CH₃)₃), -5.1 (1 C, -Si**C**H₃), -5.2 (1 C, -Si**C**H₃). *Signals for the like diastereomer:* 169.7 (1 C, -**C**OOR), 155.0 (1 C, $\mathbf{C_{Ar}}$), 152.6 (1 C, $\mathbf{C_{Ar}}$), 147.5 (1 C, $\mathbf{C_{Ar}}$), 145.6 (1 C, $\mathbf{C_{Ar}}$), 144.1 (1 C, $\mathbf{C_{Ar}}$), 139.5 (1 C, $\mathbf{C_{Ar}}$), 138.4 (1 C, $\mathbf{C_{Ar}}$), 137.5 (1 C, $\mathbf{C_{Ar}}$), 135.8 (2 C, $\mathbf{C_{Ar}}$), 129.7 (2 C, $\mathbf{C_{Ar}}$), 129.0 (1 C, $\mathbf{C_{Ar}}$), 127.7 (1 C, $\mathbf{C_{Ar}}$), 127.4 (1 C, $\mathbf{C_{Ar}}$), 127.1 (1 C, $\mathbf{C_{Ar}}$), 125.3 (2 C, $\mathbf{C_{Ar}}$), 125.1 (2 C, $\mathbf{C_{Ar}}$), 123.6 (2 C, $\mathbf{C_{Ar}}$), 122.3 (2 C, $\mathbf{C_{Ar}}$), 62.8 (1 C, -**C**H₂), 57.8 (1 C, -**C**H), 51.0

(1 C, -**C**H), 34.2 (2 C, -**C**(CH_3)$_3$), 30.1 (6 C, -C(**C**H_3)$_3$), 26.0 (3 C, -SiC(**C**H_3)$_3$), 18.5 (1 C, -Si**C**(CH_3)$_3$), -5.3 (1 C, -Si**C**H_3), -5.4 (1 C, -Si**C**H_3).

HRMS (ESI-TOF):): *m/z:* $[M+Na]^+$ calcd for $C_{42}H_{52}N_2NaO_8Si^+$: 763.3385, found 763.3383.

Data for the cyclic product 6t:

HPLC (YMC-SB, *n*-hexane/IPA = 2/1, flow = 1.0 mL min^{-1}, T_{Column} = 10 °C, *l* = 240 nm) *t_r(trans):* 13.30 min (major), 14.38 min (minor); *t_r(cis):* 19.63 min (major), 93.12 min (minor).

α_D^{20}(c = 1, $CHCl_3$): 11.6 (mixture of diastereomers).

HRMS (ESI-TOF): *m/z*: $[M+NH_4]^+$ calcd for $C_{30}H_{37}N_2O5_3^+$: 505.2697, found 505.2695.

NMR data for the cis diastereomer:

^{1}H-NMR (500 MHz, $CDCl_3$, 298 K) *δ* / ppm = 8.09 (d, *J* = 8.9 Hz, 2 H, Ar-**H**), 7.28-7.25 (m, 3 H, Ar-**H**), 7.18 (d, *J* = 8.9 Hz, 2 H, Ar-**H**), 7.08-7.06 (m, 1 H, Ar-**H**), 6.53 (s, 2 H, Ar-**H**), 5.93 (d, *J* = 15.2 Hz, 1 H, -C**H$_2$**), 5.25 (d, *J* = 15.2 Hz, 2 H, -C**H$_2$**), 5.19 (s, 1 H, -O**H**), 5.08 (d, *J* = 3.3 Hz, 1 H, -C**H**), 4.29 (d, *J* = 3.3 Hz, 1 H, -C**H**), 1.31 (s, 18 H, -C**H$_3$**).

^{13}C-NMR (125 MHz, $CDCl_3$, 298 K) *δ* / ppm = 171.1 (1 C, -**C**OOR), 153.5 (1 C, **C$_{Ar}$**), 152.8 (1 C, **C$_{Ar}$**), 147.3 (1 C, **C$_{Ar}$**), 144.1 (1 C, **C$_{Ar}$**), 135.6 (2 C, **C$_{Ar}$**), 132.3 (1 C, **C$_{Ar}$**), 132.1 (1 C, **C$_{Ar}$**), 131.9 (2 C, **C$_{Ar}$**), 131.0 (1 C, **C$_{Ar}$**), 130.1 (1 C, **C$_{Ar}$**), 129.3 (1 C, **C$_{Ar}$**), 128.3 (1 C, **C$_{Ar}$**), 127.0 (2 C, **C$_{Ar}$**), 122.7 (2 C, **C$_{Ar}$**), 70.7 (1 C, -**C**H_2), 54.8 (1 C, -**C**H), 50.1 (1 C, -**C**H), 34.4 (2 C, -**C**(CH_3)$_3$), 30.3 (6 C, -C(**C**H_3)$_3$).

NMR data for the trans diastereomer:

^{1}H-NMR (500 MHz, $CDCl_3$, 298 K) *δ* / ppm = 8.11 (d, *J* = 8.8 Hz, 2 H, Ar-**H**), 7.34 (d, *J* = 8.8 Hz, 2 H, Ar-**H**), 7.31-7.22 (m, 3 H, Ar-**H**), 7.09 (d, *J* = 7.9 Hz, 1 H, Ar-**H**), 6.42 (s, 2 H, Ar-**H**), 5.99 (d, *J* = 13.8 Hz, 1 H, -C**H$_2$**), 5.09 (d, *J* = 13.8 Hz, 1 H, -C**H$_2$**), 5.03 (s, 1 H, -O**H**), 4.70 (d, *J* = 11.1 Hz, 1 H, -C**H**), 4.51 (d, *J* = 11.1 Hz, 1 H, -C**H**), 1.20 (s, 18 H, -C**H$_3$**).

^{13}C-NMR (125 MHz, $CDCl_3$, 298 K) *δ* / ppm = -171.3 (1 C, -**C**OOR), 152.7 (1 C, **C$_{Ar}$**), 147.2 (1 C, **C$_{Ar}$**), 145.4 (1 C, **C$_{Ar}$**), 140.4 (1 C, **C$_{Ar}$**), 136.1 (2 C, **C$_{Ar}$**), 133.7 (1 C, **C$_{Ar}$**), 133.0 (1 C, **C$_{Ar}$**), 132.4 (1 C, **C$_{Ar}$**), 131.1 (2 C, **C$_{Ar}$**), 129.9 (1 C, **C$_{Ar}$**), 129.7 (1 C, **C$_{Ar}$**), 127.2 (1 C, **C$_{Ar}$**), 124.8 (2 C, **C$_{Ar}$**), 123.2 (2 C, **C$_{Ar}$**), 70.5 (1 C, -**C**H_2), 55.1 (1 C, -**C**H), 54.6 (1 C, -**C**H), 34.2 (2 C, -**C**(CH_3)$_3$), 30.2 (6 C, -(**C**H_3)$_3$).

3-Cyanophenyl benzo[c]oxepinone derivative (6u)

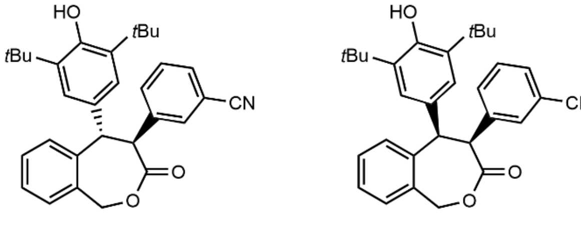

6u^{trans} **6u^{cis}**

7-membered ring **6u** was synthesized according to general procedure **F** and obtained as a colorless residue in a yield of 45% (58% for the alkylation step) as a mixture of diastereomers (*dr* 55:45; *er$_{trans'}$* 77:23; *er$_{cis}$* 66:34).

Data for the alkylation product (mixture of diastereomers) 5u:

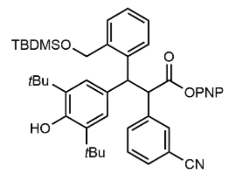

5u

Here, also the racemic product was formed with an dr of 50:50 only. Therefore, assignment of the signals to the unlike/like diastereomer was not possible unambiguously. Selected multiplets were assigned according to similarity with other entries to the substrate scope.

^{1}H-NMR (500 MHz, CDCl$_3$, 298 K) δ / ppm = *Signals for the unlike diastereomer:* 8.12 (d, *J* = 9.1 Hz, 2 H, Ar-**H**), 7.80-7.29 (m, 6 H, Ar-**H**), 7.23 (s, 2 H, Ar-**H**), 7.22-7.12 (m, 2 H, Ar-**H**), 6.59 (d, *J* = 9.1 Hz, 2 H, Ar-**H**), 5.21 (s, 1 H, -O**H**), 5.03 (d, *J* = 12.2 Hz, 1 H, -C**H$_2$**), 4.81 (d, *J* = 12.9 Hz, 1 H, -C**H**), 4.80 (d, *J* = 12.2 Hz, 1 H, -C**H$_2$**), 4.57 (d, *J* = 12.9 Hz, 1H, -C**H**), 1.39 (s, 18 H, -C**H$_3$**), 0.97 (s, 9 H, -SiC(C**H$_3$**)$_3$), 0.14 (s, 3 H, -SiC**H$_3$**), 0.11 (s, 1 H, -SiC**H$_3$**). *Selected signals for the like diastereomer:* 8.14 (d, *J* = 9.1 Hz, 2 H, Ar-**H**), 6.78 (d, *J* = 9.1 Hz, 2 H, Ar-**H**), 6.58 (s, 2 H, Ar-**H**), 5.02 (s, 1 H, -O**H**), 4.89 (d, *J* = 13.5 Hz, 1 H, -C**H**), 4.64 (d, *J* = 11.9 Hz, 1 H, -C**H$_2$**) 4.59 (d, *J* = 13.5 Hz, 1 H, -C**H**), 4.53 (d, *J* = 11.9 Hz, 1 H, -C**H$_2$**), 1.24 (s, 18 H, -C**H$_3$**), 0.87 (s, 9 H, -SiC(C**H$_3$**)$_3$), -0.03 (s, 3 H, -SiC**H$_3$**), -0.05 (s, 3 H, -SiC**H$_3$**).

^{13}C-NMR (125 MHz, CDCl$_3$, 298 K) δ / ppm = *Selected signals for the unlike diastereomer:* 170.5 (1 C, -**C**OOR), 63.3 (1 C, -**C**H$_2$), 57.1 (1 C, -**C**H), 48.7 (1 C, -**C**H), 34.5 (2 C, -**C**(CH$_3$)$_3$), 30.3 (6 C, -C(**C**H$_3$)$_3$), 26.1 (3 C, -SiC(**C**H$_3$)$_3$), 18.6 (1 C, -Si**C**(CH$_3$)$_3$), -5.1 (1 C, -Si**C**H$_3$), -5.2 (1 C, -Si**C**H$_3$). *Selected signals for the like diastereomer:* 169.9 (1 C, -**C**OOR), 62.8 (1 C, -**C**H$_2$), 57.1 (1 C, -**C**H), 51.0 (1 C, -**C**H), 34.2 (2 C, -**C**(CH$_3$)$_3$), 30.2 (6 C, -C(**C**H$_3$)$_3$), 26.0 (3 C, -SiC(**C**H$_3$)$_3$), 18.5 (1 C, -Si**C**(CH$_3$)$_3$), -5.2 (1 C, -Si**C**H$_3$), -5.4 (1 C, -Si**C**H$_3$). *Aromatic and -CN Signals for both diastereomers:* 155.0 (1 C, **C$_{Ar}$**), 155.0 (1 C, **C$_{Ar}$**), 153.1 (1 C, **C$_{Ar}$**), 152.6 (1 C, **C$_{Ar}$**), 145.6 (1 C, **C$_{Ar}$**), 145.6 (1 C, **C$_{Ar}$**), 139.6 (1 C, **C$_{Ar}$**), 138.9 (1 C, **C$_{Ar}$**), 138.4 (1 C, **C$_{Ar}$**), 138.4 (1 C, **C$_{Ar}$**), 137.7 (1 C, **C$_{Ar}$**), 137.3 (1 C, **C$_{Ar}$**), 136.2 (2 C, **C$_{Ar}$**), 135.8 (2 C, **C$_{Ar}$**), 133.2 (1 C, **C$_{Ar}$**), 133.1 (1 C, **C$_{Ar}$**), 132.4 (1 C, **C$_{Ar}$**), 131.7 (1 C, **C$_{Ar}$**), 131.3 (1 C, **C$_{Ar}$**), 130.8 (1 C, **C$_{Ar}$**), 129.6 (1 C, **C$_{Ar}$**), 129.4 (1 C, **C$_{Ar}$**), 129.0 (1 C, **C$_{Ar}$**), 128.0 (1 C, **C$_{Ar}$**), 127.7 (1 C, **C$_{Ar}$**), 127.4 (1 C, **C$_{Ar}$**), 127.3 (1 C, **C$_{Ar}$**), 127.1 (1 C, **C$_{Ar}$**), 126.9 (1 C, **C$_{Ar}$**), 126.1 (1 C, **C$_{Ar}$**), 125.5 (2 C, **C$_{Ar}$**), 125.2 (2 C, **C$_{Ar}$**),

125.2 (2 C, **C**$_{Ar}$), 125.1 (2 C, **C**$_{Ar}$), 122.4 (2 C, **C**$_{Ar}$), 122.4 (2 C, **C**$_{Ar}$), 118.5 (1 C, **C**$_{Ar}$), 118.3 (1 C, **C**$_{Ar}$), 112.8 (1 C, -**C**N), 112.7 (1 C, -**C**N).

HRMS (ESI-TOF): *m/z:* $[M+Na]^+$ calcd for $C_{43}H_{52}N_2NaO_6Si^+$: 743.3487, found 743.3487.

Data for the cyclic product 6u:

HPLC (YMC-SB, *n*-hexane/IPA = 2/1, flow = 1.0 mL min^{-1}, T_{Column} = 10 °C, *l* = 240 nm) t_r*(trans):* 9.05 min (major), 11.90 min (minor); t_r*(cis):* 38.66 min (major), 50.19 min (minor).

α_D^{20}(c = 1, $CHCl_3$): -6.1 (mixture of diastereomers).

HRMS (ESI-TOF): *m/z*: $[M+NH_4]^+$ calcd for $C_{31}H_{37}N_2O_3^+$: 485.2799, found 485.2796.

NMR data for the cis diastereomer:

^{1}H-NMR (500 MHz, $CDCl_3$, 298 K) δ / ppm = 7.58 (dt, J_1 = 1.3 Hz, J_2 = 7.4 Hz, 1 H, Ar-**H**), 7.36-7.33 (m, 2 H, Ar-**H**), 7.25-7.21 (m, 4 H, Ar-**H**), 7.06-7.04 (m, 1 H, Ar-**H**), 6.53 (s, 2 H, Ar-**H**), 5.91 (d, *J* = 15.1 Hz, 1 H, -C**H**$_2$), 5.24 (d, *J* = 15.1 Hz, 1 H, -C**H**$_2$), 5.19 (s, 1 H, -O**H**), 4.99 (d, *J* = 3.4 Hz, 1 H, -C**H**), 4.27 (d, *J* = 3.4 Hz, 1 H, -C**H**), 1.32 (s, 18 H, -C**H**$_3$).

^{13}C-NMR (125 MHz, $CDCl_3$, 298 K) δ / ppm = 171.3 (1 C, -**C**OOR), 153.5 (1 C, **C**$_{Ar}$), 140.5 (1 C, **C**$_{Ar}$), 138.4 (1 C, **C**$_{Ar}$), 135.7 (2 C, **C**$_{Ar}$), 135.5 (1 C, **C**$_{Ar}$), 134.5 (1 C, **C**$_{Ar}$), 132.3 (1 C, **C**$_{Ar}$), 132.2 (1 C, **C**$_{Ar}$), 131.3 (1 C, **C**$_{Ar}$), 130.1 (1 C, **C**$_{Ar}$), 129.2 (1 C, **C**$_{Ar}$), 128.4 (1 C, **C**$_{Ar}$), 128.2 (1 C, **C**$_{Ar}$), 126.9 (2 C, **C**$_{Ar}$), 126.9 (1 C, **C**$_{Ar}$), 118.9 (1 C, -**C**N), 111.9 (1 C, **C**$_{Ar}$), 70.7 (1 C, -**C**H$_2$), 54.9 (1 C, -**C**H), 49.9 (1 C, -**C**H), 34.4 (2 C, -**C**($CH_3)_3$), 30.3 (6 C, -C(**C**$H_3)_3$).

NMR data for the trans diastereomer:

^{1}H-NMR (500 MHz, $CDCl_3$, 298 K) δ / ppm = 7.53 (dt, J_1 = 1.4 Hz, J_2 = 7.7 Hz, 1 H, Ar-**H**), 7.49 (dt, J_1 = 1.3 Hz, J_2 = 7.6 Hz, 1 H, Ar-**H**), 7.40-7.37 (m, 2 H, Ar-**H**), 7.31-7.22 (m, 3 H, Ar-**H**), 7.08 (dd, *J* = 1.1 Hz, J_2 = 7.6 Hz, 1 H, Ar-**H**), 6.40 (s, 2 H, Ar-**H**), 6.00 (d, *J* = 13.7 Hz, 1 H, -C**H**$_2$), 5.09 (d, *J* = 13.7 Hz, 1 H, -C**H**$_2$), 5.03 (s, 1 H, -O**H**), 4.58 (d, *J* = 11.2 Hz, 1 H, -C**H**), 4.44 (d, *J* = 11.2 Hz, 1 H, -C**H**), 1.22 (s, 18 H, -C**H**$_3$).

^{13}C-NMR (125 MHz, $CDCl_3$, 298 K) δ / ppm = 171.6 (1 C, -**C**OOR), 152.7 (1 C, **C**$_{Ar}$), 140.4 (1 C, **C**$_{Ar}$), 139.6 (1 C, **C**$_{Ar}$), 136.1 (2 C, **C**$_{Ar}$), 134.7 (1 C, **C**$_{Ar}$), 133.8 (1 C, **C**$_{Ar}$), 133.8 (1 C, **C**$_{Ar}$), 133.1 (1 C, **C**$_{Ar}$), 132.5 (1 C, **C**$_{Ar}$), 131.0 (1 C, **C**$_{Ar}$), 129.9 (1 C, **C**$_{Ar}$), 129.7 (1 C, **C**$_{Ar}$), 129.0 (1 C, **C**$_{Ar}$), 127.2 (1 C, **C**$_{Ar}$), 124.8 (2 C, **C**$_{Ar}$), 118.6 (1 C, -**C**N), 112.2 (1 C, **C**$_{Ar}$), 70.5 (1 C, -**C**H$_2$), 54.9 (1 C, -**C**H), 54.8 (1 C, -**C**H), 34.3 (2 C, -**C**($CH_3)_3$), 30.2 (6 C, -C(**C**$H_3)_3$).

α-Naphthyl benzo[c]oxepinone derivative (6v)

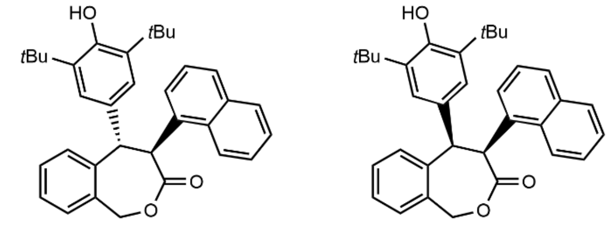

6v^{trans} **6v**^{cis}

7-membered ring **6v** was synthesized according to general procedure **F** and obtained as a colorless residue in a yield of 43% (48% for the alkylation step) as a mixture of diastereomers (*dr* 70:30; *er*$_{trans}$ 97:3; *er*$_{cis}$ 93:7).

Data for the alkylation product (mixture of diastereomers) 5v:

Here, also the racemic product was formed with an dr of 50:50 only. Therefore, assignment of the signals to the unlike/like diastereomer was not possible unambiguously. Selected multiplets were assigned according to similarity with other entries to the substrate scope.

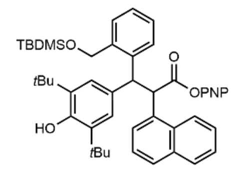

5v

^{1}H-NMR (500 MHz, CDCl$_3$, 298 K) *δ* / ppm = *Selected Signals for the unlike diastereomer:* 8-06 (d, *J* = 9.0 Hz, 2 H, Ar-**H**), 7.27 (s, 2 H, Ar-**H**), 6.57 (d, *J* = 9.0 Hz, 2 H, Ar-**H**), 5.63 (d, *J* = 12.0 Hz, 1 H, -C**H$_2$**), 5.38 (d, *J* = 12.0 Hz, -C**H$_2$**), 5.21 (s, 1 H, -O**H**), 5.04 (d, *J* = 13.0 Hz, 1 H, -C**H**), 4.59 (d, *J* = 13.0 Hz, 1 H, -C**H**), 1.42 (s, 18 H, -C**H$_3$**), 0.99 (s, 9 H, -SiC(C**H$_3$**)$_3$), 0.17 (s, 3 H, -SiC**H$_3$**), 0.12 (s, 3 H, -SiC**H$_3$**). *Selected signals for the like diastereomer:* 8.12 (d, *J* = 9.0 Hz, 2 H, Ar-**H**), 6.83 (d, *J* = 9.0 Hz, 2 H, Ar-**H**), 6.46 (s, 2 H, Ar-**H**), 5.47 (d, *J* = 11.6 Hz, 1 H, -C**H$_2$**), 4.91 (d, *J* = 13.6 Hz, 1 H, -C**H**), 4.77 (d, *J* = 11.6 Hz, 1 H, -C**H$_2$**), 4.69 (s, 1 H, -O**H**), 4.50 (d, *J* = 13.6 Hz, 1 H, -C**H**), 1.03 (s, 18 H, -C**H$_3$**), 0.84 (s, 9 H, -SiC(C**H$_3$**)$_3$), -0.09 (s, 3 H, -SiC**H$_3$**), -0.10 (s, 3 H, -SiC**H$_3$**). *Signals for both diastereomers:* 8.52 (d, *J* = 8.4 Hz, 1 H, Ar-**H**), 7.91-7.86 (m, 2 H, Ar-**H**), 7.80-7.71 (m, 4 H, Ar-**H**), 7.68-7.64 (m, 1 H, Ar-**H**), 7.58-7.52 (m, 3 H, Ar-**H**), 7.48-7.45 (m, 1 H, Ar-**H**), 7.38-7.31 (m, 4 H, Ar-**H**), 7.24-7.21 (m, 1 H, Ar-**H**), 7.18 (d, *J* = 7.6 Hz, 1 H, Ar-**H**), 7.05 (t, *J* = 7.3 Hz, 1 H, Ar-**H**), 7.95 (t, *J* = 7.3 Hz, 1 H, Ar-**H**),

^{13}C-NMR (125 MHz, CDCl$_3$, 298 K) *δ* / ppm = *Selected Signals for the unlike diastereomer:* 171.2 (1 C, -**C**OOR), 63.5 (1 C, -**C**H$_2$), 34.5 (2 C, -**C**(CH$_3$)$_3$), 30.4 (6 C, -C(**C**H$_3$)$_3$), 26.2 (3 C, -SiC(**C**H$_3$)$_3$), 18.7 (1 C, -Si**C**(CH$_3$)$_3$), -5.1 (1 C, -Si**C**H$_3$), -5.1 (1 C, -Si**C**H$_3$). *Selected signals for the like diastereomer:* 171.0 (1 C, -**C**OOR), 62.8 (1 C, -**C**H$_2$), 33.9 (2 C, -**C**(CH$_3$)$_3$), 29.9 (6 C, -C(**C**H$_3$)$_3$), 26.1 (3 C, -SiC(**C**H$_3$)$_3$), 18.5 (1 C, -Si**C**(CH$_3$)$_3$), -5.2 (1 C, -Si**C**H$_3$), -5.4 (1 C, -Si**C**H$_3$). *Signals for both diastereomers:* 155.5 (1 C, **C$_{Ar}$**), 155.4 (1 C, **C$_{Ar}$**), 153.0 (1 C, **C$_{Ar}$**), 152.1 (1 C, **C$_{Ar}$**), 145.4 (1 C, **C$_{Ar}$**), 145.4(1 C, **C$_{Ar}$**), 139.7 (1 C, **C$_{Ar}$**), 139.4 (1 C, **C$_{Ar}$**), 138.8 (1 C, **C$_{Ar}$**), 138.3 (1 C, **C$_{Ar}$**), 136.1 (2 C, **C$_{Ar}$**), 134.9 (2 C, **C$_{Ar}$**), 134.0 (1 C, **C$_{Ar}$**), 133.9 (1 C, **C$_{Ar}$**), 133.8 (1 C, **C$_{Ar}$**), 133.4 (1 C, **C$_{Ar}$**), 132.3 (1 C, **C$_{Ar}$**), 132.2 (1 C, **C$_{Ar}$**), 131.8 (1 C,

C$_{Ar}$), 131.4 (1 C, **C$_{Ar}$**), 129.4 (1 C, **C$_{Ar}$**), 129.4 (1 C, **C$_{Ar}$**), 128.6 (1 C, **C$_{Ar}$**), 128.6 (1 C, **C$_{Ar}$**), 128.4 (1 C, **C$_{Ar}$**), 128.2 (1 C, **C$_{Ar}$**), 127.7 (1 C, **C$_{Ar}$**), 127.5 (1 C, **C$_{Ar}$**), 127.2 (1 C, **C$_{Ar}$**), 127.0 (1 C, **C$_{Ar}$**), 126.9 (1 C, **C$_{Ar}$**), 126.8 (1 C, **C$_{Ar}$**), 126.7 (1 C, **C$_{Ar}$**), 126.5 (1 C, **C$_{Ar}$**), 126.1 (1 C, **C$_{Ar}$**), 126.0 (2 C, **C$_{Ar}$**), 125.9 (1 C, **C$_{Ar}$**), 125.7 (1 C, **C$_{Ar}$**), 125.5 (1 C, **C$_{Ar}$**), 125.5 (1 C, **C$_{Ar}$**), 125.4 (1 C, **C$_{Ar}$**), 125.3 (2 C, **C$_{Ar}$**), 125.2 (2 C, **C$_{Ar}$**), 125.0 (2 C, **C$_{Ar}$**), 122.8 (1 C, **C$_{Ar}$**), 122.6 (1 C, **C$_{Ar}$**), 122.6 (2 C, **C$_{Ar}$**), 122.5 (2 C, **C$_{Ar}$**), 52.4 (1 C, -**C**H), 51.5 (1 C, -**C**H), 47.7 (1 C, -**C**H).

HRMS (ESI-TOF): *m/z:* $[M+Na]^+$ calcd for $C_{46}H_{55}NNaO_6Si^+$: 768.3691, found 768.3693.

Data for the cyclic product 6v:

HPLC (YMC-SA, *n*-hexane/IPA = 4/1, flow = 1.0 mL min^{-1}, T_{Column} = 10 °C, *l* = 240 nm) t_r*(trans):* 9.69 min (major), 58.98 min (minor); t_r*(cis):* 8.97 min (major), 34.40 min (minor).

α_D^{20}(c = 1, $CHCl_3$): -35.3 (mixture of diastereomers).

HRMS (ESI-TOF): *m/z*: $[M+NH_4]^+$ calcd for $C_{34}H_{40}NO_3^+$: 510.3003, found 510.3006.

Unfortunately, the diastereomers were not separable. NMR data is given for the mixture of diastereomers (obtained from the racemic sample, cis:trans = 60:40*):*

^{1}H-NMR (500 MHz, $CDCl_3$, 298 K) *δ* / ppm = *Data for the cis diastereomer:* 7.99 (d, *J* = 8.7 Hz, 1 H, Ar-**H**), 7-94-7.92 (m, 1 H, Ar-**H**), 7.83-7.77 (m, 2 H, Ar-**H**), 7.54-7.49 (m, 2 H, Ar-**H**), 7.37-7.28 (m, 4 H, Ar-**H**), 7.08-7.06 (m, 1 H, Ar-**H**), 6.47 (s, 2 H, Ar-**H**), 6.10 (d, *J* = 15.3 Hz, 1 H, -C**H$_2$**), 5.94 (d, *J* = 2.7 Hz, 1 H, -C**H**), 5.33 (d, *J* = 15.3 Hz, 1 H, -C**H$_2$**), 5.12 (s, 1 H, -O**H**), 4.39 (d, *J* = 2.7 Hz, 1 H, -C**H**), 1.29 (s, 18 H, -C**H$_3$**). *Data for the trans diastereomer:* 7.79-7.77 (m, 1 H, Ar-**H**), 7.61-7.60 (br., 1 H, Ar-**H**), 7.54-7.49 (m, 1 H, Ar-**H**), 7.36-7.27 (m, 5 H, Ar-**H**), 7.25-7.23 (m, 1 H, Ar-**H**), 7.16-7.14 (m, 1 H, Ar-**H**), 7.08-7.06 (m, 1 H, Ar-**H**), 6.43 (s, 2 H, Ar-**H**), 5.97 (d, *J* = 13.6 Hz, 1 H, -C**H$_2$**), 5.37-5.34 (br., 1 H, -C**H**), 5.18 (d, *J* = 13.6 Hz, 1 H, -C**H$_2$**), 4.86 (s, 1 H, -O**H**), 4.80 (br. d, *J* = 9.2 Hz, 1 H, -C**H**), 1.10 (s, 18 H, -C**H$_3$**).

^{13}C-NMR (125 MHz, $CDCl_3$, 298 K) *δ* / ppm = *Data for the cis diastereomer:* 171.9 (1 C, -**C**OOR), 153.1 (1 C, **C$_{Ar}$**), 141.0 (1 C, **C$_{Ar}$**), 135.0 (2 C, **C$_{Ar}$**), 134.2 (1 C, **C$_{Ar}$**), 134.0 (1 C, **C$_{Ar}$**), 132.5 (1 C, **C$_{Ar}$**), 132.3 (1 C, **C$_{Ar}$**), 130.6 (1 C, **C$_{Ar}$**), 129.9 (2 C, **C$_{Ar}$**), 129.1 (1 C, **C$_{Ar}$**), 128.3 (1 C, **C$_{Ar}$**), 128.1 (1 C, **C$_{Ar}$**), 128.0 (1 C, **C$_{Ar}$**), 127.2 (2 C, **C$_{Ar}$**), 127.2 (1 C, **C$_{Ar}$**), 126.7 (1 C, **C$_{Ar}$**), 125.2 (1 C, **C$_{Ar}$**), 124.9 (1 C, **C$_{Ar}$**), 124.7 (1 C, **C$_{Ar}$**), 70.9 (1 C, -**C**H$_2$), 53.8 (1 C, -**C**H), 42.7 (1 C, -**C**H), 34.4 (2 C, -**C**(CH$_3$)$_3$), 30.3 (6 C, -C(**C**H$_3$)$_3$). *Data for the trans diastereomer:* 172.2 (1 C, -**C**OOR), 152.4 (1 C, **C$_{Ar}$**), 141.3 (1 C, **C$_{Ar}$**), 135.5 (2 C, **C$_{Ar}$**), 134.4 (1 C, **C$_{Ar}$**), 133.9 (1 C, **C$_{Ar}$**), 132.8 (1 C, **C$_{Ar}$**), 132.0 (1 C, **C$_{Ar}$**), 131.2 (1 C, **C$_{Ar}$**), 131.2 (1 C, **C$_{Ar}$**), 131.1 (1 C, **C$_{Ar}$**), 129.6 (2 C, **C$_{Ar}$**), 129.0 (1 C, **C$_{Ar}$**), 128.3 (1 C, **C$_{Ar}$**), 127.1 (1 C, **C$_{Ar}$**), 126.8 (2 C, **C$_{Ar}$**), 126.0 (1 C, **C$_{Ar}$**), 125.3 (1 C, **C$_{Ar}$**), 125.3 (1 C, **C$_{Ar}$**), 121.2 (1 C, **C$_{Ar}$**), 70.4 (1 C, -**C**H$_2$), 53.8 (2 H, -C**H**), 34.1 (2 C, -**C**(CH$_3$)$_3$), 30.0 (6 C, -C(**C**H$_3$)$_3$).

β-Naphthyl benzo[c]oxepinone derivative (6w)

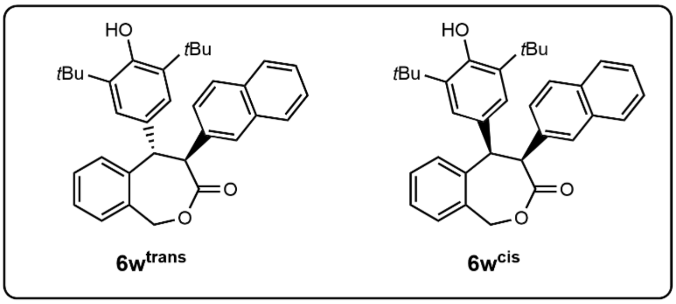

7-membered ring **6w** was synthesized according to general procedure **F** and obtained as a colorless residue in a yield of 50% (51% for the alkylation step) as a mixture of diastereomers (*dr* 45:55; er_{trans} 94:6; er_{cis} 88:12).

Data for the alkylation product (mixture of diastereomers) 5w:

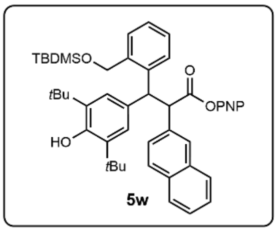

^{1}H-NMR (500 MHz, $CDCl_3$, 298 K) *δ* / ppm = *Signals for the unlike diastereomer:* 8.11 (d, *J* = 9.1 Hz, 2 H, Ar-**H**), 7.95 (s, 1 H, Ar-**H**), 7.82-7.73 (m, 4 H, Ar-**H**), 7.64 (dd, J_1 = 1.6 Hz, J_2 = 8.6 Hz, 1 H, Ar-**H**), 7.51 (d, *J* = 7.7 Hz, 1 H, Ar-**H**), 7.47-7.43 (m, 2 H, Ar-**H**), 7.31 (s, 2 H, Ar-**H**), 7.16 (t, *J* = 7.4 Hz, 1 H, Ar-**H**), 7.08 (t, *J* = 7.4 Hz, 1 H, Ar-**H**), 6.63 (d, *J* = 9.1 Hz, 2 H, Ar-**H**), 5.20 (s, 1 H, -O**H**), 5.12 (d, *J* = 12.2 Hz, 1 H, -C**H₂**), 4.96 (d, *J* = 12.2 Hz, 1 H, -C**H₂**), 4.82 (d, *J* = 13.3 Hz, 1 H, -C**H**), 4.69 (d, *J* = 13.3 Hz, 1 H, -C**H**), 1.43 (s, 18 H, -C**H₃**), 0.96 (s, 9 H, -SiC(C**H₃**)₃), 0.07 (s, 3 H, -SiC**H₃**), 0.07 (s, 3 H, -SiC**H₃**). *Selected signals for the like diastereomer:* 8.14 (d, *J* = 9.1 Hz, 2 H, Ar-**H**), 7.82-7.73 (m, 2 H, Ar-**H**), 7.58-7.56 (m, 2 H, Ar-**H**), 7.42-7.37 (m, 2 H, Ar-**H**), 7.29 (s, 1 H, Ar-**H**), 6.82 (d, *J* = 9.1 Hz, 2 H, Ar-**H**), 6.55 (s, 2 H, Ar-**H**), 4.95 (d, *J* = 13.6 Hz, 1 H, -C**H**), 4.87 (1 H, -O**H**), 4.77 (d, *J* = 11.8 Hz, 1 H, -C**H₂**), 4.66 (d, *J* = 11.8 Hz, 1 H, -C**H₂**), 4.63 (d, *J* = 13.6 Hz, 1 H, -C**H**), 1.09 (s, 18 H, -C**H₃**), 0.88 (s, 9 H, -SiC(C**H₃**)₃), -0.03 (s, 3 H, -SiC**H₃**), -0.04 (s, 3 H, -SiC**H₃**).

^{13}C-NMR (125 MHz, $CDCl_3$, 298 K) *δ* / ppm = *Signals for the unlike diastereomer:* 171.2 (1 C, -**C**OOR), 155.4 (1 C, $\mathbf{C_{Ar}}$), 152.9 (1 C, $\mathbf{C_{Ar}}$), 145.4 (1 C, $\mathbf{C_{Ar}}$), 139.0 (1 C, $\mathbf{C_{Ar}}$), 137.7 (1 C, $\mathbf{C_{Ar}}$), 136.0 (2 C, $\mathbf{C_{Ar}}$), 133.4 (1 C, $\mathbf{C_{Ar}}$), 133.3 (1 C, $\mathbf{C_{Ar}}$), 132.9 (1 C, $\mathbf{C_{Ar}}$), 131.8 (1 C, $\mathbf{C_{Ar}}$), 128.5 (1 C, $\mathbf{C_{Ar}}$), 128.1 (1 C, $\mathbf{C_{Ar}}$), 127.9 (1 C, $\mathbf{C_{Ar}}$), 127.7 (1 C, $\mathbf{C_{Ar}}$), 127.0 (2 C, $\mathbf{C_{Ar}}$), 126.5 (1 C, $\mathbf{C_{Ar}}$), 126.4 (1 C, $\mathbf{C_{Ar}}$), 126.3 (1 C, $\mathbf{C_{Ar}}$), 126.2 (1 C, $\mathbf{C_{Ar}}$), 126.1 (1 C, $\mathbf{C_{Ar}}$), 125.5 (2 C, $\mathbf{C_{Ar}}$), 125.0 (2 C, $\mathbf{C_{Ar}}$), 122.5 (2 C, $\mathbf{C_{Ar}}$), 63.0 (1 C, -**C**H₂), 57.9 (1 C, -**C**H), 48.5 (1 C, -**C**H), 34.5 (2 C, -**C**(CH₃)₃), 30.4 (6 C, -C(**C**H₃)₃), 26.1 (3 C, -SiC(**C**H₃)₃), 18.6 (1 C, -Si**C**(CH₃)₃), -5.2 (1 C, -Si**C**H₃), -5.3 (1 C, -Si**C**H₃). *Selected signals for the like diastereomer:* 170.8 (1 C, -**C**OOR), 155.4 (1 C, $\mathbf{C_{Ar}}$), 152.2 (1 C, $\mathbf{C_{Ar}}$), 139.7 (1 C, $\mathbf{C_{Ar}}$), 139.1 (1 C, $\mathbf{C_{Ar}}$), 135.3 (2 C, $\mathbf{C_{Ar}}$), 133.9 (1 C, $\mathbf{C_{Ar}}$), 129.8 (1 C, $\mathbf{C_{Ar}}$), 128.3 (1 C, $\mathbf{C_{Ar}}$), 128.2 (1 C, $\mathbf{C_{Ar}}$), 127.8 (1 C, $\mathbf{C_{Ar}}$), 127.5 (1 C, $\mathbf{C_{Ar}}$), 127.5 (1 C, $\mathbf{C_{Ar}}$), 127.0 (1 C, $\mathbf{C_{Ar}}$), 126.1 (1 C, $\mathbf{C_{Ar}}$), 125.3 (2 C, $\mathbf{C_{Ar}}$), 125.2 (2 C, $\mathbf{C_{Ar}}$), 62.8 (1 C, -**C**H₂), 58.1 (1 C, -**C**H), 50.7 (1 C, -**C**H), 34.0 (2 C, -**C**(CH₃)₃), 30.0 (6 C, -C(**C**H₃)₃), 26.0 (3 C, -SiC(**C**H₃)₃), 18.5 (1 C, -Si**C**(CH₃)₃), -5.3 (1 C, -Si**C**H₃), -5.4 (1 C, -Si**C**H₃).

HRMS (ESI-TOF): *m/z:* $[M+Na]^+$ calcd for $C_{46}H_{55}NNaO_6Si^+$: 768.3691, found 768.3689.

Data for the cyclic product 6w:

HPLC (YMC-SB, *n*-hexane/IPA = 10/1, flow = 1.0 mL min^{-1}, T_{Column} = 10 °C, *l* = 240 nm) t_r*(trans):* 14.95 min (major), 72.06 min (minor); t_r*(cis):* 17.46 min (major), 30.89 min (minor).

α_D^{20}(c = 1, $CHCl_3$): -45.5 (mixture of diastereomers).

HRMS (ESI-TOF): *m/z*: $[M+NH_4]^+$ calcd for $C_{34}H_{40}NO_3^+$: 510.3003, found 510.3008.

NMR data for the cis diastereomer:

^{1}H-NMR (500 MHz, $CDCl_3$, 298 K) *δ* / ppm = 7.81 (d, *J* = 7.4 Hz, 1 H, Ar-**H**), 7.72 (d, *J* = 10.0 Hz, 1 H, Ar-**H**), 7.70 (d, *J* = 11.6 Hz, 1 H, Ar-**H**), 7.60 (s, 1 H, Ar-**H**), 7.46-7.43 (m, 2 H, Ar-**H**), 7.27-7.22 (m, 3 H, Ar-**H**), 7.09-7.06 (m, 2 H, Ar-**H**), 6.57 (s, 2 H, Ar-**H**), 5.97 (d, *J* = 15.2 Hz, 1 H, -C**H$_2$**), 5.26 (d, *J* = 15.2 Hz, 1 H, -C**H$_2$**), 5.14 (s, 1 H, -O**H**), 5.13 (d, *J* = 3.3 Hz, 1 H, -C**H**), 4.40 (d, *J* = 3.3 Hz, 1 H, -C**H**), 1.27 (s, 18 H, -C**H$_3$**).

^{13}C-NMR (125 MHz, $CDCl_3$, 298 K) *δ* / ppm = 172.2 (1 C, -**C**OOR), 153.2 (1 C, **C$_{Ar}$**), 141.2 (1 C, **C$_{Ar}$**), 135.3 (2 C, **C$_{Ar}$**), 134.5 (1 C, **C$_{Ar}$**), 133.0 (1 C, **C$_{Ar}$**), 132.9 (1 C, **C$_{Ar}$**), 132.6 (1 C, **C$_{Ar}$**), 132.4 (1 C, **C$_{Ar}$**), 131.0 (1 C, **C$_{Ar}$**), 129.8 (1 C, **C$_{Ar}$**), 129.1 (1 C, **C$_{Ar}$**), 128.9 (1 C, **C$_{Ar}$**), 128.3 (1 C, **C$_{Ar}$**), 128.1 (1 C, **C$_{Ar}$**), 127.6 (1 C, **C$_{Ar}$**), 127.2 (2 C, **C$_{Ar}$**), 127.0 (1 C, **C$_{Ar}$**), 126.7 (1 C, **C$_{Ar}$**), 126.1 (1 C, **C$_{Ar}$**), 125.8 (1 C, **C$_{Ar}$**), 70.7 (1 C, -**C**H$_2$), 55.1 (1 C, -**C**H), 50.4 (1 C, -**C**H), 34.4 (2 C, -**C**(CH$_3$)$_3$), 30.3 (6 C, -C(**C**H$_3$)$_3$).

NMR data for the trans diastereomer:

^{1}H-NMR (500 MHz, $CDCl_3$, 298 K) *δ* / ppm = 7.83 (t, *J* = 9.3 Hz, 1 H, Ar-**H**), 7.65 (d, *J* = 7.8 Hz, 1 H, Ar-**H**), 7.58 (d, *J* = 8.5 Hz, 1 H, Ar-**H**), 7.47-7.43 (m, 2 H, Ar-**H**), 7.39 (s, 1 H, Ar-**H**), 7.32-7.25 (m, 3 H, Ar-**H**), 7.17 (d, *J* = 7.2 Hz, 1 H, Ar-**H**), 6.44 (s, 2 H, Ar-**H**), 5.91 (d, *J* = 13.4 Hz, 1 H, -C**H$_2$**), 5.12 (d, *J* = 13.4 Hz, 1 H, -C**H$_2$**), 4.94 (s, 1 H, -O**H**), 4.69 (app. s, 2 H, -C**H**), 1.11 (s, 18 H, -C**H$_3$**).

^{13}C-NMR (125 MHz, $CDCl_3$, 298 K) *δ* / ppm = 172.6 (1 C, -**C**OOR), 152.4 (1 C, **C$_{Ar}$**), 140.9 (1 C, **C$_{Ar}$**), 135.6 (2 C, **C$_{Ar}$**), 135.1 (1 C, **C$_{Ar}$**), 134.4 (1 C, **C$_{Ar}$**), 133.6 (1 C, **C$_{Ar}$**), 133.2 (1 C, **C$_{Ar}$**), 132.5 (1 C, **C$_{Ar}$**), 132.3 (1 C, **C$_{Ar}$**), 129.6 (1 C, **C$_{Ar}$**), 129.4 (1 C, **C$_{Ar}$**), 128.7 (1 C, **C$_{Ar}$**), 128.0 (1 C, **C$_{Ar}$**), 127.8 (1 C, **C$_{Ar}$**), 127.6 (1 C, **C$_{Ar}$**), 127.2 (1 C, **C$_{Ar}$**), 126.9 (1 C, **C$_{Ar}$**), 126.0 (2 C, **C$_{Ar}$**), 124.9 (2 C, **C$_{Ar}$**), 70.3 (1 C, -**C**H$_2$), 56.1 (1 C, -**C**H), 53.8 (1 C, -**C**H), 34.1 (2 C, -**C**(CH$_3$)$_3$), 30.0 (6 C, -C(**C**H$_3$)$_3$).

Thiophen-3-yl benzo[c]oxepinone derivative (6x)

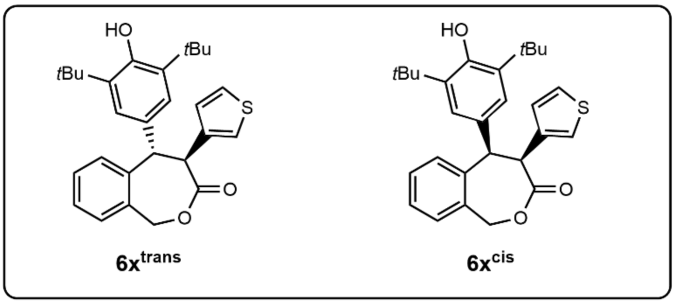

7-membered ring **6x** was synthesized according to general procedure **F** and obtained as a colorless residue in a yield of 29% (30% for the alkylation step) as a mixture of diastereomers (*dr* 45:55; er_{trans} 93:7; er_{cis} 90:10).

Data for the alkylation product (mixture of diastereomers) 5x:

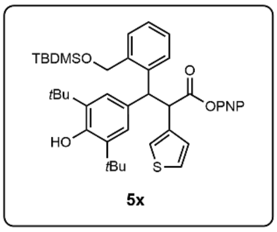

^{1}H-NMR (300 MHz, $CDCl_3$, 298 K) *δ* / ppm = *Signals for the unlike diastereomer:* 8.12 (d, *J* = 9.1 Hz, 2 H, Ar-**H**), 7.47 (d, *J* = 7.7 Hz, 1 H, Ar-**H**), 7.35-7.33 (m, 1 H, Ar-**H**), 7.27-7.26 (m, 1 H, Ar-**H**), 7.24-7.21 (m, 1 H, Ar-**H**), 7.24 (s, 2 H, Ar-**H**), 7.18-7.14 (m, 2 H, Ar-**H**), 7.11 (dd, J_1 = 1.0 Hz, J_2 = 5.0 Hz, 1 H, Ar-**H**), 6.61 (d, *J* = 9.1 Hz, 2 H, Ar-**H**), 5.18 (s, 1 H, -O**H**), 4.92 (d, *J* = 12.2 Hz, 1 H, -C**H₂**), 4.89 (d, *J* = 12.2 Hz, 1 H, -C**H₂**), 4.77 (d, *J* = 13.1 Hz, 1 H, -C**H**), 4.63 (d, *J* = 13.1 Hz, 1 H, -C**H**), 1.40 (s, 18 H, -C**H₃**), 0.96 (s, 9 H, -SiC(C**H₃**), 0.12 (s, 3 H, -SiC**H₃**), 0.09 (s, 3 H, -SiC**H₃**). *Selected signals for the like diastereomer:* 8.13 (d, *J* = 9.1 Hz, 2 H, Ar-**H**), 7.72 (d, *J* = 7.5 Hz, 1 H, Ar-**H**), 7.54 (d, *J* = 7.4 Hz, 1 H, -C**H**), 7.40 (t, *J* = 7.5 Hz, 1 H, Ar-**H**), 7.35-7.33 (m, 1 H, Ar-**H**), 7.27-7.26 (m, 1 H, Ar-**H**), 6.99 (dd, J_1 = 1.0 Hz, J_2 = 2.9 Hz, 1 H, Ar-**H**), 6.94 (dd, J_1 = 1.0 Hz, J_2 = 5.0 Hz, 1 H, Ar-**H**), 6.76 (d, *J* = 9.1 Hz, 1 H, Ar-**H**), 6.64 (s, 2 H, Ar-**H**), 5.00 (s, 1 H, -O**H**), 4.88 (d, *J* = 13.6 Hz, 1 H, -C**H**), 4.63-4.61 (m, 2 H, -C**H**), 4.58 (d, *J* = 13.6 Hz, 1 H, -C**H**), 1.27 (s, 18 H, -C**H₃**), 0.85 (s, 9 H, -SiC(C**H₃**), -0.06 (s, 3 H, -SiC**H₃**), -0.07 (s, 3 H, -SiC**H₃**).

^{13}C-NMR (125 MHz, $CDCl_3$, 298 K) *δ* / ppm = *Signals for the unlike diastereomer:* 170.8 (1 C, -**C**OOR), 155.4 (1 C, C_{Ar}), 152.9 (1 C, C_{Ar}), 145.4 (1 C, C_{Ar}), 138.9 (1 C, C_{Ar}), 138.3 (1 C, C_{Ar}), 136.2 (1 C, C_{Ar}), 136.0 (2 C, C_{Ar}), 131.6 (1 C, C_{Ar}), 127.5 (1 C, C_{Ar}), 127.4 (1 C, C_{Ar}), 127.2 (1 C, C_{Ar}), 126.6 (1 C, C_{Ar}), 126.1 (1 C, C_{Ar}), 125.8 (1 C, C_{Ar}), 125.4 (2 C, C_{Ar}), 125.1 (2 C, C_{Ar}), 123.8 (1 C, C_{Ar}), 122.5 (2 C, C_{Ar}), 63.1 (1 C, -**C**H_2), 53.2 (1 C, -**C**H), 49.1 (1 C, -**C**H), 34.4 (2 C, -**C**$(CH_3)_3$), 30.3 (6 C, -C(**C**$H_3)_3$), 26.1 (3 C, -SiC(**C**$H_3)_3$), 18.6 (1 C, -Si**C**$(CH_3)_3$), -5.1 (1 C, -Si**C**H_3), -5.2 (1 C, -Si**C**H_3). *Selected signals for the like diastereomer:* 170.6 (1 C, -**C**OOR), 152.4 (1 C, C_{Ar}), 139.6 (1 C, C_{Ar}), 138.8 (1 C, C_{Ar}), 136.6 (1 C, C_{Ar}), 135.4 (2 C, C_{Ar}), 130.0 (1 C, C_{Ar}), 127.6 (1 C, C_{Ar}), 127.0 (1 C, C_{Ar}), 126.9 (1 C, C_{Ar}), 125.7 (1 C, C_{Ar}), 125.2 (2 C, C_{Ar}), 125.2 (2 C, C_{Ar}), 123.5 (1 C, C_{Ar}), 62.7 (1 C, -**C**H_2), 53.7 (1 C, -**C**H), 50.8 (1 C, -**C**H), 34.2 (2 C, -**C**$(CH_3)_3$), 30.2 (6 C, -C(**C**$H_3)_3$), 26.0 (3 C, -SiC(**C**$H_3)_3$), 18.5 (1 C, -Si**C**$(CH_3)_3$), -5.3 (1 C, -Si**C**H_3), -5.4 (1 C, -Si**C**H_3).

HRMS (ESI-TOF): *m/z:* $[M+Na]^+$ calcd for $C_{40}H_{51}NNaO_6SSi^+$: 724.3099, found 724.3097.

Data for the cyclic product 6x:

HPLC (YMC-SB, *n*-hexane/IPA = 4/1, flow = 1.0 mL min^{-1}, T_{Column} = 10 °C, *l* = 240 nm) t_r*(trans):* 10.21 min (major), 16.67 min (minor); t_r*(cis):* 11.24 min (major), 14.31 min (minor).

α_D^{20}(c = 1, $CHCl_3$): -61.1 (mixture of diastereomers).

HRMS (ESI-TOF): *m/z*: $[M+NH_4]^+$ calcd for $C_{28}H_{36}NO_3S^+$: 466.2410, found 466.2418.

NMR data for the cis diastereomer:

^{1}H-NMR (500 MHz, $CDCl_3$, 298 K) *δ* / ppm = 7.25-7.20 (m, 4 H, Ar-**H**), 7.11-7.08 (m, 2 H, Ar-**H**), 6.79 (dd, J_1 = 0-8 Hz, J_2 = 5.0 Hz, 1 H, Ar-**H**), 6.51 (s, 2 H, Ar-**H**), 5.86 (d, *J* = 15.0 Hz, 1 H, -C**H₂**), 5.19 (d, *J* = 3.2 Hz, 1 H, -C**H**), 5.18 (d, *J* = 15.0 Hz, 1 H, -C**H₂**), 5.10 (s, 1 H, -O**H**), 4.36 (d, *J* = 3.2 Hz, 1 H, -C**H**), 1.30 (s, 18 H, -C**H₃**).

^{13}C-NMR (125 MHz, $CDCl_3$, 298 K) *δ* / ppm = 171.8 (1 C, -**C**OOR), 153.1 (1 C, $\mathbf{C_{Ar}}$), 140.7 (1 C, $\mathbf{C_{Ar}}$), 136.8 (1 C, $\mathbf{C_{Ar}}$), 135.0 (2 C, $\mathbf{C_{Ar}}$), 132.7 (1 C, $\mathbf{C_{Ar}}$), 132.4 (1 C, $\mathbf{C_{Ar}}$), 31.0 (1 C, $\mathbf{C_{Ar}}$), 129.3 (1 C, $\mathbf{C_{Ar}}$), 129.0 (1 C, $\mathbf{C_{Ar}}$), 128.1 (1 C, $\mathbf{C_{Ar}}$), 126.9 (2 C, $\mathbf{C_{Ar}}$), 126.7 (1 C, $\mathbf{C_{Ar}}$), 124.8 (1 C, $\mathbf{C_{Ar}}$), 124.0 (1 C, $\mathbf{C_{Ar}}$), 70.6 (1 C, -**C**H_2), 54.9 (1 C, -**C**H), 46.0 (1 C, -**C**H), 34.4 (2 C, -**C**$(CH_3)_3$), 30.3 (6 C, -C(**C**$H_3)_3$).

NMR data for the trans diastereomer:

^{1}H-NMR (500 MHz, $CDCl_3$, 298 K) *δ* / ppm = 7.27-7.20 (m, 4 H, Ar-**H**), 7.11-7.09 (m, 2 H, Ar-**H**), 6.85 (dd, J_1 = 1.0 Hz, J_2 = 3.0 Hz, 1 H, Ar-**H**), 6.51 (s, 2 H, Ar-**H**), 5.78 (d, *J* = 13.7 Hz, 1 H, -C**H₂**), 5.04 (d, *J* = 13.7 Hz, 1 H, -C**H₂**), 5.02 (s, 1 H, -O**H**), 4.66 (d, *J* = 10.0 Hz, 1 H, -C**H**), 4.49 (d, *J* = 10.0 Hz, 1 H, -C**H**), 1.27 (s, 18 H, -C**H₃**).

^{13}C-NMR (125 MHz, $CDCl_3$, 298 K) *δ* / ppm = 172.3 (1 C, -**C**OOR), 152.5 (1 C, $\mathbf{C_{Ar}}$), 140.7 (1 C, $\mathbf{C_{Ar}}$), 137.8 (1 C, $\mathbf{C_{Ar}}$), 135.8 (2 C, $\mathbf{C_{Ar}}$), 134.7 (1 C, $\mathbf{C_{Ar}}$), 133.5 (1 C, $\mathbf{C_{Ar}}$), 132.6 (1 C, $\mathbf{C_{Ar}}$), 129.6 (1 C, $\mathbf{C_{Ar}}$), 129.3 (1 C, $\mathbf{C_{Ar}}$), 128.2 (1 C, $\mathbf{C_{Ar}}$), 126.9 (1 C, $\mathbf{C_{Ar}}$), 125.4 (1 C, $\mathbf{C_{Ar}}$), 124.9 (2 C, $\mathbf{C_{Ar}}$), 123.3 (1 C, $\mathbf{C_{Ar}}$), 70.3 (1 C, -**C**H_2), 53.8 (1 C, -**C**H), 51.6 (1 C, -**C**H), 34.3 (2 C, -**C**$(CH_3)_3$), 30.3 (6 C, -C(**C**$H_3)_3$).

7-Fluoro benzo[c]oxepinone derivative (6aa)

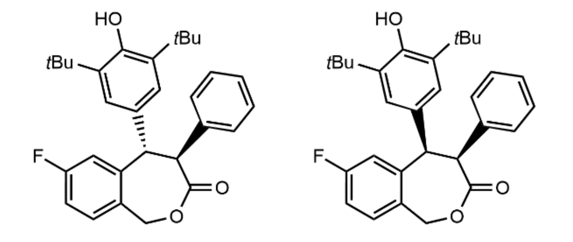

6aa^{trans} **6aa**^{cis}

7-membered ring **6aa** was synthesized according to general procedure **F** and obtained as a colorless residue in a yield of 59% (60% for the alkylation step) as a mixture of diastereomers (*dr* 50:50; er_{trans} 95:5; er_{cis} 88:12).

Data for the alkylation product (mixture of diastereomers) 5aa:

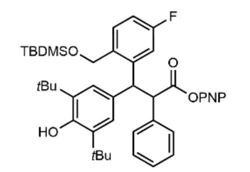

5aa

^{1}H-NMR (500 MHz, $CDCl_3$, 298 K) *δ* / ppm = *Signals for the unlike diastereomer:* 8.10 (d, *J* = 9.1 Hz, 2 H, Ar-**H**), 7.49-7.47 (m, 2 H, Ar-**H**), 7.28-7.25 (m, 3 H, Ar-**H**), 7.23 (s, 2 H, Ar-**H**), 7.19-7.17 (m, 1 H, Ar-**H**), 7.11 (dd, J_1 = 2.5 Hz, J_2 = 10.4 Hz, 1 H, Ar-**H**), 6.79 (dt, J_1 =2.5 Hz, J_2 = 8.4 Hz, 1 H, Ar-**H**), 6.62 (d, *J* = 9.1 Hz, 2 H, Ar-**H**), 5.20 (s, 1 H, -O**H**), 5.00 (d, *J* = 12.2 Hz, 1 H, -C$\mathbf{H_2}$), 4.71 (d, *J* = 13.1 Hz, 1 H, -C**H**), 4.66 (d, *J* = 12.2 Hz, 1 H, -C$\mathbf{H_2}$), 4.57 (d, *J* = 13.1 Hz, 1 H, -C**H**), 1.41 (s, 18 H, -C$\mathbf{H_3}$), 0.95 (s, 9 H, -SiC(C$\mathbf{H_3}$)$_3$), 0.10 (s, 3 H, -Si**C**H_3), 0.08 (s, 3 H, -SiC$\mathbf{H_3}$). *Selected signals for the like diastereomer:* 8.16 (d, *J* = 9.1 Hz, 2 H, Ar-**H**), 7.49-7.47 (m, 2 H, Ar-**H**), 7.39-7.37 (m, 2 H, Ar-**H**), 7.28-7.25 (m, 2 H, Ar-**H**), 7.25-7.20 (m, 1 H, Ar-**H**), 7.02 (dt, J_1 = 2.5 Hz, J_2 = 8.4 Hz, 1 H, Ar-**H**), 6.91 (d, *J* = 9.1 Hz, 2 H, Ar-**H**), 6.54 (s, 2 H, Ar-**H**), 4.96 (s, 1 H, -O**H**), 4.83 (d, *J* = 13.4 Hz, 1 H, -C**H**), 4.62 (d, *J* = 12.0 Hz, 1 H, -C$\mathbf{H_2}$), 4.53 (d, *J* = 13.4 Hz, 1 H, -C**H**), 4.39 (d, *J* = 12.0 Hz, 1 H, -C$\mathbf{H_2}$), 1.23 (s, 18 H, -C$\mathbf{H_3}$), 0.95 (s, 9 H, -SiC(C$\mathbf{H_3}$)$_3$), -0.06 (s, 3 H, -SiC$\mathbf{H_3}$), -0.07 (s, 3 H, -SiC$\mathbf{H_3}$).

^{13}C-NMR (125 MHz, $CDCl_3$, 298 K) *δ* / ppm = *Signals for the unlike diastereomer:* 170.9 (1 C, -**C**OOR), 162.0 (d, *J* = 243.9 Hz, 1 C, $\mathbf{C_{Ar}}$-F), 155.4 (1 C, $\mathbf{C_{Ar}}$), 153.1 (1 C, $\mathbf{C_{Ar}}$), 145.5 (1 C, $\mathbf{C_{Ar}}$), 140.4 (d, *J* = 6.5 Hz, 1 C, $\mathbf{C_{Ar}}$), 136.2 (2 C, $\mathbf{C_{Ar}}$), 135.6 (1 C, $\mathbf{C_{Ar}}$), 134.8 (d, *J* = 2.7 Hz, 1 C, $\mathbf{C_{Ar}}$), 131.2 (1 C, $\mathbf{C_{Ar}}$), 129.0 (d, *J* = 8.2 Hz, 1 C, Ar-**H**), 128.9 (2 C, $\mathbf{C_{Ar}}$), 128.7 (2 C, $\mathbf{C_{Ar}}$), 128.1 (1 C, $\mathbf{C_{Ar}}$), 125. 4 (2 C, $\mathbf{C_{Ar}}$), 125.1 (2 C, $\mathbf{C_{Ar}}$), 122.5 (2 C, $\mathbf{C_{Ar}}$), 113.6 (d, *J* = 22.5 Hz, 1 C, $\mathbf{C_{Ar}}$), 113.1 (d, *J* = 20.8 Hz, 1 C, $\mathbf{C_{Ar}}$), 62.6 (1 C, -**C**H_2), 57.7 (1 C, -**C**H), 48.4 (1 C, -**C**H), 34.5 (2 C, -**C**$(CH_3)_3$), 30.4 (6 C, -C(**C**H_3)$_3$), 26.1 (3 C, -SiC(**C**H_3)$_3$), 18.6 (1 C, -Si**C**$(CH_3)_3$), -5.2 (1 C, -Si**C**H_3), -5.2 (1 C, -Si**C**H_3). *Selected signals for the like diastereomer:* 170.6 (1 C, -**C**OOR), 162.1 (d, *J* = 244.6 Hz, 1 H, $\mathbf{C_{Ar}}$-F), 155.3 (1 C, $\mathbf{C_{Ar}}$), 152.5 (1 C, $\mathbf{C_{Ar}}$), 145.6 (1 C, $\mathbf{C_{Ar}}$), 141.5 (d, *J* = 6.5 Hz, 1 C, $\mathbf{C_{Ar}}$), 136.2 (1 C, $\mathbf{C_{Ar}}$), 135.5 (2 C, $\mathbf{C_{Ar}}$), 130.2 (1 C, $\mathbf{C_{Ar}}$), 129.4 (1 C, $\mathbf{C_{Ar}}$), 129.2 (1 C, $\mathbf{C_{Ar}}$), 128.8 (2 C, $\mathbf{C_{Ar}}$), 127.9 (1 C, $\mathbf{C_{Ar}}$), 125.3 (2 C, $\mathbf{C_{Ar}}$), 125.2 (2 C, $\mathbf{C_{Ar}}$), 122.5 (2 C, $\mathbf{C_{Ar}}$), 113.3 (d, *J* = 20.7 Hz, 1 C, $\mathbf{C_{Ar}}$), 112.5 (d, *J* = 22.7 Hz, 1 C, $\mathbf{C_{Ar}}$), 62.3 (1 C, -**C**H_2),

57.9 (1 C, -**C**H), 50.5 (1 C, -**C**H), 34.2 (3 C, -**C**(CH_3)$_3$), 30.2 (6 C, -C(**C**H_3)$_3$), 26.0 (3 C, -SiC(**C**H_3)$_3$), 18.5 (1 C, -Si**C**(CH_3)$_3$), -5.3 (1 C, -Si**C**H_3), -5.4 (1 C, -Si**C**H_3).

HRMS (ESI-TOF):): *m/z:* $[M+Na]^+$ calcd for $C_{42}H_{52}FNNaO_6Si^+$: 736.3440, found 736.3437.

Data for the cyclic product 6aa:

HPLC (YMC-SB, *n*-hexane/IPA = 10/1, flow = 1.0 mL min^{-1}, T_{Column} = 10 °C, *l* = 240 nm) t_r*(trans):* 18.69 min (major), 34.13 min (minor); t_r*(cis):* 12.40 min (major), 16.39 min (minor).

α_D^{20}(c = 1, $CHCl_3$): -45.6 (mixture of diastereomers).

HRMS (ESI-TOF): *m/z*: $[M+NH_4]^+$ calcd for $C_{30}H_{37}FNO_3^+$: 478.2752, found 478.2756.

NMR data for the cis diastereomer:

^{1}H-NMR (500 MHz, $CDCl_3$, 298 K) *δ* / ppm = 7.29-7.26 (m, 1 H, Ar-**H**), 7.25-7.22 (m, 2 H, Ar-**H**), 7.04 (dd, J_1 = 5.5 Hz, J_2 = 8.4 Hz, 1 H, Ar-**H**), 7.00-6.99 (m, 2 H, Ar-**H**), 6.97-6.91 (m, 2 H, Ar-**H**), 6.54 (s, 2 H, Ar-**H**), 5.87 (d, *J* = 15.3 Hz, 1 H, -C**H$_2$**), 5.15 (d, *J* = 15.3 Hz, 1 H, -C**H$_2$**), 5.14 (s, 1 H, -O**H**), 4.91 (d, *J* = 3.3 Hz, 1 H, -C**H**), 4.28 (d, *J* = 3.3 Hz. 1 H, -C**H**), 1.31 (s, 18 H, -C**H$_3$**).

^{19}F-NMR (282 MHz, $CDCl_3$, 298 K) *δ* / ppm = -116.0 (1 F, Ar-**F**).

^{13}C-NMR (125 MHz, $CDCl_3$, 298 K) *δ* / ppm = 171.7 (1 C, -**C**OOR), 160.8 (d, *J* = 246.5 Hz, 1 C, **C$_{Ar}$**-F), 153.3 (1 C, **C$_{Ar}$**), 137.0 (d, *J* = 3.3 Hz, 1 C, **C$_{Ar}$**), 136.6 (1 C, **C$_{Ar}$**), 135.3 (2 C, **C$_{Ar}$**), 134.4 (d, *J* = 6.4 Hz, 1 C, **C$_{Ar}$**), 134.3 (d, *J* = 8.0 Hz, 1 C, **C$_{Ar}$**), 130.9 (2 C, **C$_{Ar}$**), 130.6 (1 C, **C$_{Ar}$**), 127.8 (2 C, **C$_{Ar}$**), 127.7 (1 C, **C$_{Ar}$**), 127.1 (2 C, **C$_{Ar}$**), 116.2 (d, *J* = 21.0 Hz, 1 C, **C$_{Ar}$**), 114.4 (d, *J* = 21.8 Hz, 1 C, **C$_{Ar}$**), 69.9 (1 C, -**C**H_2), 54.6 (1 C, -**C**H), 50.2 (1 C, -**C**H), 34.4 (1 C, -**C**(CH_3)$_3$), 30.3 (6 C, -C(**C**H_3)$_3$).

NMR data for the trans diastereomer (contains significant amounts of the cis diastereomer):

^{1}H-NMR (500 MHz, $CDCl_3$, 298 K) *δ* / ppm = 7.25-6.89 (m, 7 H, Ar-**H**), 6.45 (s, 2 H, Ar-**H**), 5.77 (d, *J* = 13.8 Hz, 1 H, -C**H$_2$**), 5.01 (s, 1 H, -O**H**), 4.99 (d, *J* = 13.8 Hz, 1 H, -C**H$_2$**), 4.54 (d, *J* = 10.1 Hz, 1 H, -C**H**), 4.48 (d, *J* = 10.1 Hz, 1 H, -C**H**), 1.23 (s, 18 H, -C**H$_3$**).

^{19}F-NMR (282 MHz, $CDCl_3$, 298 K) *δ* / ppm = -115.5 (1 F, Ar-**F**).

^{13}C-NMR (125 MHz, $CDCl_3$, 298 K) *δ* / ppm = *Only unambiguously assignable aliphatic signals given due to impossible isolation as a pure diastereomer* 69.5 (1 C, -**C**H_2), 55.9 (1 C, -**C**H), 53.3 (1 C, -**C**H), 34.3 (2 C, -**C**(CH_3)$_3$), 30.2 (6 C, -C(**C**H_3)$_3$).

8-Fluoro benzo[c]oxepinone derivative (6ab)

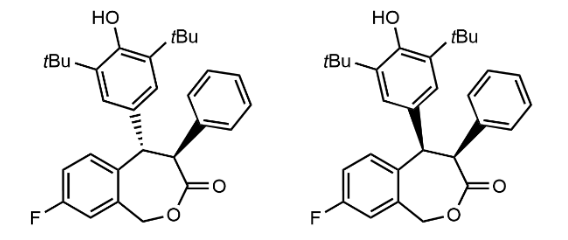

6ab^{trans} **6ab**^{cis}

7-membered ring **6ab** was synthesized according to general procedure **F** and obtained as a colorless residue in a yield of 56% (60% for the alkylation step) as a mixture of diastereomers (*dr* 50:50; $er_{trans'}$ 95:5; er_{cis} 92:8).

Data for the alkylation product (mixture of diastereomers) 5ab:

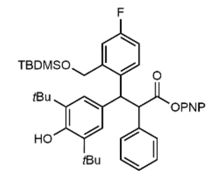

5ab

^{1}H-NMR (500 MHz, CDCl$_3$, 298 K) δ / ppm = *Signals for the unlike diastereomer:* 8.14 (d, J = 9.1 Hz, 2 H, Ar-**H**), 7.48 (d, J = 7.2 Hz, 1 H, Ar-**H**), 7.40-7.29 (m, 5 H, Ar-**H**), 7.24 (s, 2 H, Ar-**H**), 7.13-7.11 (m,1 H, Ar-**H**), 6.91-6.87 (m, 1 H, Ar-**H**), 6.66 (d, J = 9.1 Hz, 2 H, Ar-**H**), 5.23 (s, 1 H, -O**H**), 4.85 (d, J = 12.2 Hz, 1 H, -C**H$_2$**), 4.75 (d, J = 13.7 Hz, 1 H, -C**H**), 4.73 (d, J = 12.2 Hz, 1 H, -C**H$_2$**), 4.67 (d, J = 13.7 Hz, 1 H, -C**H**), 1.45 (s, 18 H, -C**H$_3$**), 0.98 (s, 9 H, -SiC(C**H$_3$**)$_3$), 0.12 (s, 3 H, -SiC**H$_3$**), 0.10 (s, 3 H, -SiC**H$_3$**). *Selected signals for the like diastereomer:* 8.20 (d, J = 9.1 Hz, 2 H, Ar-**H**), 7.77-7-71 (m, 2 H, Ar-**H**), 7.40-7.29 (m, 4 H, Ar-**H**), 7.24-7.21 (m, 1 H, Ar-**H**), 7.13-7.11 (m, 1 H, Ar-**H**), 6.90 (d, J = 9.1 Hz, 2 H, Ar-**H**), 6.56 (s, 2 H, Ar-**H**), 4.99 (s, 1 H, -O**H**), 4.94 (d, J = 14.3 Hz, 1 H, -C**H**), 4.60 (d, J = 14.3 Hz, 1 H, -C**H**), 4.53 (d, J = 11.9 Hz, 1 H, -C**H**), 4.47 (d, J = 11.9 Hz, 1 H, -C**H**), 1.26 (s, 18 H, -C**H$_3$**), 0.90 (s, 9 H, -SiC(C**H$_3$**)$_3$), -0.02 (s, 3 H, -SiC**H$_3$**), -0.02 (s, 3 H, -SiC**H$_3$**).

^{13}C-NMR (125 MHz, CDCl$_3$, 298 K) δ / ppm = *Signals for the unlike diastereomer:* 171.0 (1 C, -**C**OOR), 161.7 (d, J = 242.9 Hz, 1 C, **C$_{Ar}$**-F), 155.4 (1 C, **C$_{Ar}$**), 153.0 (1 C, **C$_{Ar}$**), 145.5 (1 C, **C$_{Ar}$**), 141.9 (d, J =7.1 Hz, 1 C, **C$_{Ar}$**), 136.2 (2 C, **C$_{Ar}$**), 135.8 (1 C, **C$_{Ar}$**), 133.1 (d, J = 3.2 Hz, 1 C, **C$_{Ar}$**), 131.5 (1 C, **C$_{Ar}$**), 128.9 (2 C, **C$_{Ar}$**), 128.6 (2 C, **C$_{Ar}$**), 128.1 (1 C, **C$_{Ar}$**), 127.9 (d, J = 7.7 Hz, 1 C, **C$_{Ar}$**), 125.3 (2 C, **C$_{Ar}$**), 125.1 (2 C, **C$_{Ar}$**), 122.5 (2 C, **C$_{Ar}$**), 113.6 (d, J = 22.9 Hz, 1 C, **C$_{Ar}$**), 113.4 (d, J = 21.6 Hz, 1 C, **C$_{Ar}$**), 62.2 (1 C, -**C**H$_2$), 57.8 (1 C, -**C**H), 48.4 (1 C, -**C**H), 34.5 (2 C, -**C**(CH$_3$)$_3$), 30.4 (6 C, -C(**C**H$_3$)$_3$), 26.1 (3 C, -SiC(**C**H$_3$)$_3$), 18.6 (1 C, -Si**C**(CH$_3$)$_3$), -5.3 (2 C, -Si**C**H$_3$). *Selected signals for the like diastereomer:* 170.7 (1 C, -**C**OOR), 152.4 (1 C, **C$_{Ar}$**), 142.7 (d, J = 7.3 Hz, 1 C, **C$_{Ar}$**), 136.3 (1 C, **C$_{Ar}$**), 135.5 (2 C, **C$_{Ar}$**), 134.5 (d, J = 4.0 Hz, 1 C, **C$_{Ar}$**), 128.8 (2 C, **C$_{Ar}$**), 128.7 (2 C, **C$_{Ar}$**), 125.3 (2 C, **C$_{Ar}$**), 125.3 (2 C, **C$_{Ar}$**), 122.4 (2 C, **C$_{Ar}$**), 114.1 (d, J = 22.9 Hz, 1 C, **C$_{Ar}$**), 113.0 (d, J = 21.5 Hz, 1 C, **C$_{Ar}$**), 62.0 (1 C, -**C**H$_2$), 58.1 (1 C, -**C**H), 50.3 (1 C, -**C**H), 34.2 (2 C, -**C**(CH$_3$)$_3$), 30.2 (6 C, -C(**C**H$_3$)$_3$), 26.0 (3 C, -SiC(**C**H$_3$)$_3$), 18.5 (1 C, -Si**C**(CH$_3$)$_3$), -5.3 (1 C, -Si**C**H$_3$), -5.5 (1 C, -Si**C**H$_3$).

HRMS (ESI-TOF):): *m/z:* [M+Na]$^+$ calcd for $C_{42}H_{52}FNNaO_6Si^+$: 736.3440, found 436.3438.

Data for the cyclic product 6ab:

HPLC (YMC-SA, *n*-hexane/IPA = 10/1, flow = 1.0 mL min^{-1}, T_{Column} = 10 °C, *l* = 220 nm)) t_r*(trans):* 12.27 min (major), 39.22 min (minor); t_r*(cis):* 12.90 min (major), 49.95 min (minor).

α_D^{20}(c = 1, $CHCl_3$): -56.7 (mixture of diastereomers).

HRMS (ESI-TOF): *m/z*: $[M+NH_4]^+$ calcd for $C_{30}H_{37}FNO_3^+$: 478.2752, found 478.2753.

NMR data for the cis diastereomer:

^{1}H-NMR (500 MHz, $CDCl_3$, 298 K) *δ* / ppm = 7.28-7.21 (m, 4 H, Ar-**H**), 7.00-6.98 (m, 2 H, Ar-**H**), 6.92 (dt, J_1 = 2.5 Hz, J_2 = 8.2 Hz, 1H, Ar-**H**), 6.76 (dd, J_1 = 2.5 Hz, J_2 = 9.7 Hz, 1 H, Ar-**H**), 6.54 (s, 2 H, Ar-**H**), 5.86 (d, *J* = 15.1 Hz, 1 H, -C**H$_2$**), 5.20 (d, *J* = 15.1 Hz, 1 H, -C**H$_2$**), 5.15 (s, 1 H, -O**H**), 4.93 (d, *J* = 3.4 Hz, 1 H, -C**H**), 4.26 (d, *J* = 3.4 Hz, 1 H, -C**H**), 1.32 (s, 18 H, -C**H$_3$**).

^{19}F-NMR (282 MHz, $CDCl_3$, 298 K) *δ* / ppm = -113.1 (1 F, Ar-**F**).

^{13}C-NMR (125 MHz, $CDCl_3$, 298 K) *δ* / ppm = 171.7 (1 C, -**C**OOR), 162.7 (d, *J* = 147.9 Hz, 1 C, **C$_{Ar}$**), 153.3 (1 C, **C$_{Ar}$**), 143.7 (d, *J* = 7.2Hz, 1 C, **C$_{Ar}$**), 136.4 (1 C, **C$_{Ar}$**), 135.3 (2 C, **C$_{Ar}$**), 130.8 (2 C, **C$_{Ar}$**), 130.3 (1 C, **C$_{Ar}$**), 130.0 (d, *J* = 8.3 Hz, 1 C, **C$_{Ar}$**), 128.6 (d, *J* = 2.9 Hz, 1 C, **C$_{Ar}$**), 127.7 (2 C, **C$_{Ar}$**), 127.7 (1 C, **C$_{Ar}$**), 127.1 (2 C, **C$_{Ar}$**), 118.7 (d, *J* = 21.5 Hz, 1 C, **C$_{Ar}$**), 114.0 (d, *J* = 21.6 Hz, 1 C, **C$_{Ar}$**), 69.8 (1 C, -**C**H$_2$), 55.1 (1 C, -**C**H), -50.0 (1 C, -**C**H), 34.4 (2 C, -**C**(CH_3)$_3$), 30.3 (6 C, -C(**C**H$_3$)$_3$).

NMR data for the trans diastereomer (contains significant amounts of the cis diastereomer):

^{1}H-NMR (500 MHz, $CDCl_3$, 298 K) *δ* / ppm = 7.28-7.21 (m, 4 H, Ar-**H**), 7.16-7.14 (m, 2 H, Ar-**H**), 6.93-6.90 (m, 1 H, Ar-**H**), 6.82 (dd, J_1 = 2.6 Hz, J_2 = 9.7 Hz, 1 H, Ar-**H**), 6.46 (s, 2 H, Ar-**H**), 5-79 (d, *J* = 13.8 Hz, 1 H, -C**H$_2$**), 5.05 (d, *J* = 13.8 Hz, 1 H, -C**H**), 5.02 (s, 1 H, -O**H**), 4.50 (App. s, 2 H, -C**H**), 1.23 (s, 18 H, -C**H$_3$**).

^{19}F-NMR (282 MHz, $CDCl_3$, 298 K) *δ* / ppm = -111.9 (1 F, Ar-**F**).

^{13}C-NMR (125 MHz, $CDCl_3$, 298 K) *δ* / ppm = *Only unambiguously assignable aliphatic signals given due to impossible isolation as a pure diastereomer:* 172.3 (1 C, -**C**OOR), 69.4 (1 C, -**C**H$_2$), 55.9 (1 C, -**C**H), 54.1 (1 C, -**C**H), 34.3 (2 C, -**C**(CH_3)$_3$), 30.2 (6 C, -C(**C**H$_3$)$_3$).

7-Chloro benzo[c]oxepinone derivative (6ba)

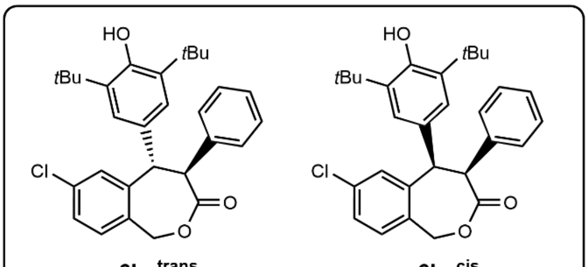

$\mathbf{6ba^{trans}}$ $\mathbf{6ba^{cis}}$

7-membered ring **6ba** was synthesized according to general procedure **F** and obtained as a colorless residue in a yield of 35% (36% for the alkylation step) as a mixture of diastereomers (*dr* 50:50; er_{trans} 93:7; er_{cis} 85:15).

Data for the alkylation product (mixture of diastereomers) 5ba:

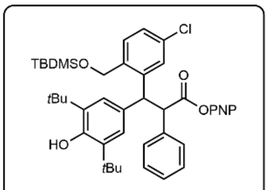

5ba

^{1}H-NMR (500 MHz, $CDCl_3$, 298 K) δ / ppm = *Signals for the unlike diastereomer:* 8.11 (d, *J* = 9.2 Hz, 2 H, Ar-**H**), 7.50-7.47 (m, 2 H, Ar-**H**), 7.40 (d, *J* = 2.0 Hz, 1 H, Ar-**H**), 7.32-7.23 (m, 3 H, Ar-**H**), 7.23 (s, 2 H, Ar-**H**), 7.18-7.17 (m, 1 H, Ar-**H**), 7.08 (dd, J_1 = 2.0 Hz, J_2 = 8.3 Hz, 1 H, Ar-**H**), 6.62 (d, *J* = 9.2 Hz, 2 H, Ar-**H**), 5.21 (s, 1 H, -O**H**), 4.92 (d, *J* = 12.2 Hz, 1 H, -C$\mathbf{H_2}$), 4.69 (d, *J* = 12.2 Hz, 1 H, -C$\mathbf{H_2}$), 4.68 (d, *J* = 13.3 Hz, 1 H, -C**H**), 4.60 (d, *J* = 13.3 Hz, 1 H, -C**H**), 1.42 (s, 18 H, -C$\mathbf{H_3}$), 0.94 (s, 9 H, -SiC(C$\mathbf{H_3}$)$_3$), 0.09 (s, 3 H, -SiC$\mathbf{H_3}$), 0.07 (s, 3 H, -SiC$\mathbf{H_3}$). *Selected signals for the like diastereomer:* 8.18 (d, *J* = 9.2 Hz, 2 H, Ar-**H**), 7.74 (d, *J* = 2.0 Hz, 1 H, Ar-**H**), 7.50-7.47 (m, 1 H, Ar-**H**), 7.32-7.23 (m, 5 H, Ar-**H**), 7.18-7.17 (m, 1 H, Ar-**H**), 6.96 (d, *J* = 9.2 Hz, 2 H, Ar-**H**), 6.52 (s, 2 H, Ar-**H**), 4.97 (s, 1 H, -O**H**), 4.83 (d, *J* = 13.8 Hz, 1 H, -C**H**), 4.54 (d, *J* = 11.9 Hz, 1 H, -C**H**$_2$), 4.52 (d, *J* = 13.8 Hz, 1 H, -C**H**), 4.42 (d, *J* = 11.9 Hz, 1 H, -C$\mathbf{H_2}$), 1.23 (s, 18 H, -C$\mathbf{H_3}$), 0.84 (s, 9 H, -SiC(C$\mathbf{H_3}$)$_3$), -0.08 (s, 3 H, -SiC$\mathbf{H_3}$), -0.09 (s, 3 H, -SiC$\mathbf{H_3}$).

^{13}C-NMR (125 MHz, $CDCl_3$, 298 K) δ / ppm = *Signals for the unlike diastereomer:* 170.9 (1 C, -**C**OOR), 155.4 (1 C, $\mathbf{C_{Ar}}$), 153.1 (1 C, $\mathbf{C_{Ar}}$), 145.5 (1 C, $\mathbf{C_{Ar}}$), 139.8 (1 C, $\mathbf{C_{Ar}}$), 137.6 (1 C, $\mathbf{C_{Ar}}$), 136.3 (1 C, $\mathbf{C_{Ar}}$), 135.5 (2 C, $\mathbf{C_{Ar}}$), 132.7 (1 C, $\mathbf{C_{Ar}}$), 131.1 (1 C, $\mathbf{C_{Ar}}$), 128.9 (2 C, $\mathbf{C_{Ar}}$), 128.7 (2 C, $\mathbf{C_{Ar}}$), 128.6 (1 C, $\mathbf{C_{Ar}}$), 128.2 (1 C, $\mathbf{C_{Ar}}$), 126.8 (1 C, $\mathbf{C_{Ar}}$), 126.6 (1 C, $\mathbf{C_{Ar}}$), 125.4 (2 C, $\mathbf{C_{Ar}}$), 125.1 (2 C, $\mathbf{C_{Ar}}$), 122.5 (2 C, $\mathbf{C_{Ar}}$), 62.5 (1 C, -**C**OOR), 57.6 (1 C, -**C**H), 48.7 (1 C, -**C**H), 34.5 (2 C, -**C**(CH_3)$_3$), 30.4 (6 C, -C(**C**H_3)$_3$), 26.1 (3 C, -Si**C**(CH_3)$_3$), 18.6 (1 C, -Si**C**(CH_3)$_3$), -5.2 (1 C, -Si**C**H_3), -5.2 (1 C, -Si**C**H_3). *Selected signals for the like diastereomer:* 170.7 (1 C, -**C**OOR), 155.3 (1 C, $\mathbf{C_{Ar}}$), 152.5 (1 C, $\mathbf{C_{Ar}}$), 145.6 (1 C, $\mathbf{C_{Ar}}$), 141.1 (1 C, $\mathbf{C_{Ar}}$), 138.4 (1 C, $\mathbf{C_{Ar}}$), 136.2 (1 C, $\mathbf{C_{Ar}}$), 135.5 (2 C, $\mathbf{C_{Ar}}$), 132.6 (1 C, $\mathbf{C_{Ar}}$), 129.6 (1 C, $\mathbf{C_{Ar}}$), 129.0 (1 C, $\mathbf{C_{Ar}}$), 128.8 (2 C, $\mathbf{C_{Ar}}$), 128.7 (2 C, $\mathbf{C_{Ar}}$), 128.0 (1 C, $\mathbf{C_{Ar}}$), 126.9 (1 C, $\mathbf{C_{Ar}}$), 125.4 (2 C, $\mathbf{C_{Ar}}$), 125.3 (2 C, $\mathbf{C_{Ar}}$), 122.6 (2 C, $\mathbf{C_{Ar}}$), 62.2 (1 C, -**C**H_2), 57.7 (1 C, -**C**H), 50.7 (1 C, -**C**H), 34.2 (2 C, -**C**(CH_3)$_3$), 30.2 (6 C, -C(**C**H_3)$_3$), 26.0 (3 C, -SiC(**C**H_3)$_3$), 18.5 (1 C, -Si**C**H_3)$_3$), -5.3 (1 C, -Si**C**H_3), -5.4 (1 C, -Si**C**H_3).

HRMS (ESI-TOF):): *m/z:* [M+Na]$^+$ calcd for $C_{42}H_{52}ClNNaO_6Si^+$: 752.3145, found 752.3135.

Data for the cyclic product 6ba:

HPLC (YMC-SB, *n*-hexane/IPA = 10/1, flow = 1.0 mL min^{-1}, T_{Column} = 10 °C, *l* = 240 nm) t_r*(trans):* 19.21 min (major), 40.41 min (minor); t_r*(cis):* 13.11 min (major), 16.55 min (minor).

α_D^{20}(c = 1, $CHCl_3$): -52.0 (mixture of diastereomers).

HRMS (ESI-TOF): *m/z*: $[M+NH_4]^+$ calcd for $C_{30}H_{37}ClNO_3^+$: 494.2456, found 494.2453.

NMR data for the cis diastereomer:

^{1}H-NMR (500 MHz, $CDCl_3$, 298 K) *δ* / ppm = 7.28-7.26 (m, 1 H, Ar-**H**), 7.25-7.22 (m, 2 H, Ar-**H**), 7.21-7.17 (m, 2 H, Ar-**H**), 7.05 (d, *J* = 1.8 Hz, 1 H, Ar-**H**), 6.99-6.97 (m, 2 H, Ar-**H**), 6.55 (s, 2 H, Ar-**H**), 5.84 (d, *J* = 15.2 Hz, 1 H, -C**H₂**), 5.19 (d, *J* = 15.2 Hz, 1 H, -C**H₂**), 5.16 (s, 1 H, -O**H**), 4.89 (md, *J* = 3.3 Hz, 1 H, -C**H**), 4.24 (d, *J* = 3.3 Hz, 1 H, -C**H**), 1.33 (s, 18 H, -C**H₃**).

^{13}C-NMR (125 MHz, $CDCl_3$, 298 K) *δ* / ppm = 171.7 (1 C, -**C**OOR), 153.4 (1 C, **C$_{Ar}$**), 143.1 (1 C, **C$_{Ar}$**), 136.4 (1 C, **C$_{Ar}$**), 135.3 (2 C, **C$_{Ar}$**), 134.6 (1 C, **C$_{Ar}$**), 132.0 (1 C, **C$_{Ar}$**), 131.2 (1 C, **C$_{Ar}$**), 130.8 (2 C, **C$_{Ar}$**), 130.0 (1 C, **C$_{Ar}$**), 129.5 (1 C, **C$_{Ar}$**), 127.8 (2 C, **C$_{Ar}$**), 127.7 (1 C, **C$_{Ar}$**), 127.1 (2 C, **C$_{Ar}$**), 127.0 (1 C, **C$_{Ar}$**), 69.9 (1 C, -**C**H_2), 55.0 (1 C, -C**H**), -50.1 (1C, -**C**H), 34.4 (2 C, -**C**$(CH_3)_3$), 30.3 (6 C, -C(**C**$H_3)_3$).

NMR data for the trans diastereomer:

^{1}H-NMR (500 MHz, $CDCl_3$, 298 K) *δ* / ppm = 7.29-7.25 (m, 3 H, Ar-**H**), 7.21-7.20 (m, 2 H, Ar-**H**), 7.15-7.13 (m, 3 H, Ar-**H**), 6.45 (s, 2 H, Ar-**H**), 5.77 (d, *J* = 13.8 Hz, 1 H, -C**H₂**), 5.03 (s, 1 H, -O**H**), 5.03 (d, *J* = 13.8 Hz, 1 H, -C**H₂**), 4.50 (d, *J* = 10.1 Hz, 1 H, -C**H**), 4.46 (d, *J* = 10.1 Hz, 1 H, -C**H**), 1.24 (s, 18 H, -C**H₃**).

^{13}C-NMR (125 MHz, $CDCl_3$, 298 K) *δ* / ppm = 172.0 (1 C, -**C**OOR), 152.7 (1 C, **C$_{Ar}$**), 142.9 (1 C, **C$_{Ar}$**), 137.3 (1 C, **C$_{Ar}$**), 135.9 (2 C, **C$_{Ar}$**), 135.4 (1 C, **C$_{Ar}$**), 133.6 (1 C, **C$_{Ar}$**), 132.3 (1 C, **C$_{Ar}$**), 132.2 (1 C, **C$_{Ar}$**), 130.8 (1 C, **C$_{Ar}$**), 129.5 (2 C, **C$_{Ar}$**), 128.4 (2 C, **C$_{Ar}$**), 127.7 (1 C, **C$_{Ar}$**), 127.3 (1 C, **C$_{Ar}$**), 124.9 (2 C, **C$_{Ar}$**), 69.4 (1 C, -**C**H_2), 56.0 (1 C, -**C**H), 53.9 (1 C, -**C**H), 34.3 (2 C, -**C**$(CH_3)_3$), 30.2 (6 C, -C(**C**$H_3)_3$).

8-Chloro benzo[c]oxepinone derivative (6bb)

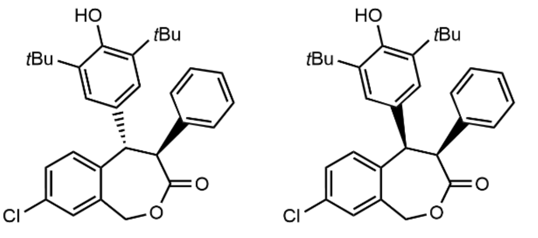

$\mathbf{6bb}^{trans}$ $\mathbf{6bb}^{cis}$

7-membered ring **6bb** was synthesized according to general procedure **F** and obtained as a colorless residue in a yield of 21% (23% for the alkylation step) as a mixture of diastereomers (*dr* 50:50; $er_{trans'}$ 95:5; er_{cis} 89:11).

Data for the alkylation product (mixture of diastereomers) 5ab:

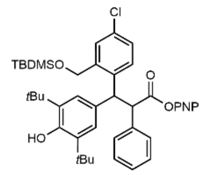

5bb

^{1}H-NMR (500 MHz, $CDCl_3$, 298 K) δ / ppm = *Signals for the unlike diastereomer:* 8.14 (d, *J* = 9.0 Hz, 2 H, Ar-**H**), 7.49 (d, *J* = 7.6Hz, 2 H, Ar-**H**), 7.38-7.26 (m, 4 H, Ar-**H**), 7.23 (s, 2 H, Ar-**H**), 7.20-7.17 (m, 2 H, Ar-**H**), 6.65 (d, *J* = 9.0 Hz, 2 H, Ar-**H**), 5.23 (s, 1 H, -O**H**), 4.91 (d, *J* =12.4 Hz, 1 H, -C**H₂**), 4.76 (d, *J* = 13.6 Hz, 1 H, -C**H**), 4.73 (d, *J* = 12.4 Hz, 1 H, -C**H₂**), 4.64 (d, *J* = 13.6 Hz, 1 H, -C**H**), 1.44 (s, 18 H, -C**H₃**), 0.99 (s, 9 H, -SiC(C**H₃**)₃), 0.13 (s, 3 H, -SiC**H₃**), 0.11 (s, 3 H, -SiC**H₃**). *Selected signals for the like diastereomer:* 8.20 (d, *J* = 9.0 Hz, 2 H, Ar-**H**), 7.70 (d, *J* = 8.3 Hz, 1 H, Ar-**H**), 7.60 (App. s, 1 H, Ar-**H**), 7.43-7.41 (m, 1 H, Ar-**H**), 7.32-7.17 (m, 5 H, Ar-**H**), 6.91 (d, *J* = 9.0 Hz, 2 H, Ar-**H**), 6.55 (s, 2 H, Ar-**H**), 5.00 (s, 1 H, -O**H**), 4.92 (d, *J* = 14.30 Hz, 1 H, -C**H**), 4.58 (d, *J* = 14.4 Hz, 1 H, -C**H**), 4.55 (d, *J* = 11.8 Hz, 1 H, -C**H₂**), 4.46 (d, *J* = 11.8 Hz, 1 H, -C**H₂**), 1.26 (s, 18 H, -C**H₃**), 0.89 (s, 9 H, -SiC(C**H₃**)₃), -0.01 (s, 3 H, -SiC**H₃**), -0.02 (s, 3 H, -SiC**H₃**).

^{13}C-NMR (125 MHz, $CDCl_3$, 298 K) δ / ppm = *Signals for the unlike diastereomer:* 171.0 (1 C, -**C**OOR), 155.4 (1 C, $\mathbf{C_{Ar}}$), 153.0 (1 C, $\mathbf{C_{Ar}}$), 145.5 (1 C, $\mathbf{C_{Ar}}$), 141.2 (1 C, $\mathbf{C_{Ar}}$), 136.2 (2 C, $\mathbf{C_{Ar}}$), 135.7 (1 C, $\mathbf{C_{Ar}}$), 135.5 (1 C, $\mathbf{C_{Ar}}$), 132.4 (1 C, $\mathbf{C_{Ar}}$), 131.2 (1 C, $\mathbf{C_{Ar}}$), 128.9 (2 C, $\mathbf{C_{Ar}}$), 128.7 (2 C, $\mathbf{C_{Ar}}$), 128.1 (1 C, $\mathbf{C_{Ar}}$), 127.8 (1 C, $\mathbf{C_{Ar}}$), 127.1 (1 C, $\mathbf{C_{Ar}}$), 126.9 (1 C, $\mathbf{C_{Ar}}$), 125.4 (2 C, $\mathbf{C_{Ar}}$), 125.1 (2 C, $\mathbf{C_{Ar}}$), 122.5 (2 C, $\mathbf{C_{Ar}}$), 62.54 (1 C, -**C**H₂), 57.6 (1 C, -**C**H), 48.3 (1 C, -**C**H), 34.5 (2 C, -**C**(CH₃)₃), 30.4 (6 C, -C(**C**H₃)₃), 26.1 (3 C, -SiC(**C**H₃)₃), 18.6 (1 C, -Si**C**(CH₃)₃), -5.2 (1 C, -Si**C**H₃), -5.2 (1 C, -Si**C**H₃). *Selected signals for the like diastereomer:* 170.6 (1 C, -**C**OOR), 155.3 (1 C, $\mathbf{C_{Ar}}$), 152.5 (1 C, $\mathbf{C_{Ar}}$), 145.6 (1 C, $\mathbf{C_{Ar}}$), 141.9 (1 C, $\mathbf{C_{Ar}}$), 137.5 (1 C, $\mathbf{C_{Ar}}$), 136.2 (2 C, $\mathbf{C_{Ar}}$), 133.1 (1 C, $\mathbf{C_{Ar}}$), 129.2 (1 C, $\mathbf{C_{Ar}}$), 128.8 (2 C, $\mathbf{C_{Ar}}$), 128.7 (2 C, $\mathbf{C_{Ar}}$), 127.9 (1 C, $\mathbf{C_{Ar}}$), 127.3 (1 C, $\mathbf{C_{Ar}}$), 126.6 (1 C, $\mathbf{C_{Ar}}$), 126.3 (1 C, $\mathbf{C_{Ar}}$), 125.3 (2 C, $\mathbf{C_{Ar}}$), 125.3 (2 C, $\mathbf{C_{Ar}}$), 122.4 (2 C, $\mathbf{C_{Ar}}$), 62.1 (1 C, -**C**H₂), 57.9 (1 C, -**C**H), 50.3 (1 C, -**C**H), 34.2 (2 C, -**C**(CH₃)₃), 30.2 (6 C, -C(**C**H₃)₃), 26.0 (3 C, -SiC(**C**H₃)₃), 18.5 (1 C, -Si**C**(CH₃)₃), -5.3 (1 C, -Si**C**H₃), -5.4 (1 C, -Si**C**H₃).

HRMS (ESI-TOF):): *m/z:* $[M+Na]^+$ calcd for $C_{42}H_{52}ClNNaO_6Si^+$: 752.3145, found 752.3131.

Data for the cyclic product 6bb:

HPLC (YMC-SB, *n*-hexane/IPA = 10/1, flow = 1.0 mL min^{-1}, T_{Column} = 10 °C, *l* = 240 nm) t_r*(trans):* 15.78 min (major), 37.09 min (minor); t_r*(cis):* 14.38 min (major), 21.08 min (minor).

α_D^{20}(c = 1, $CHCl_3$): -27.3 (mixture of diastereomers).

HRMS (ESI-TOF): *m/z*: $[M+NH_4]^+$ calcd for $C_{30}H_{37}ClNO_3^+$: 494.2456, found 494.2459.

Unfortunately, the diastereomers were not separable. NMR data is given for the mixture of diastereomers (obtained from the racemic sample, cis:trans = 70:30*):*

^{1}H-NMR (300 MHz, $CDCl_3$, 298 K) *δ* / ppm = *Signals for the cis diastereomer:* 7.28-7.98 (m, 8 H, Ar-**H**), 6.53 (s, 2 H, Ar-**H**), 5.86 (d, *J* = 15.3 Hz, 1 H, -C**H₂**), 5.15 (s, 1 H, -O**H**), 5.15 (d, *J* = 15.3 Hz, 1 H, -C**H₂**), 4.89 (d, *J* = 3.3 Hz, 1 H, -C**H**), 4.27 (d, *J* = 3.3 Hz, 1 H, -C**H**), 1.31 (s, 18 H, -C**H₃**). *Signals for the trans diastereomer:* 7.28-7.98 (m, 8 H, Ar-**H**), 6.46 (s, 2 H, Ar-**H**), 5.76 (d, *J* = 13.8 Hz, 1 H, -C**H₂**), 5.01 (s, 1 H, -O**H**), 4.99 (d, *J* = 13.8 Hz, 1 H, -C**H₂**), 4.53 (d, *J* = 10.2 Hz, 1 H, -C**H**), 4.48 (d, *J* = 10.2 Hz, 1 H, -C**H**), 1.23 (s, 18 H, -C**H₃**).

^{13}C-NMR (75 MHz, $CDCl_3$, 298 K) *δ* / ppm = *Signals for the cis diastereomer:* 171.6 (1 C, -**C**OOR), 153.4 (1 C, $\mathbf{C_{Ar}}$), 139.8 (1 C, $\mathbf{C_{Ar}}$), 136.5 (1 C, $\mathbf{C_{Ar}}$), 135.3 (2 C, $\mathbf{C_{Ar}}$), 134.3 (1 C, $\mathbf{C_{Ar}}$), 133.9 (1 C, $\mathbf{C_{Ar}}$), 132.1 (1 C, $\mathbf{C_{Ar}}$), 130.8 (2 C, $\mathbf{C_{Ar}}$), 129.4 (1 C, $\mathbf{C_{Ar}}$), 129.1 (1 C, $\mathbf{C_{Ar}}$), 127.8 (3 C, $\mathbf{C_{Ar}}$), 127.7 (1 C, $\mathbf{C_{Ar}}$), 127.1 (2 C, $\mathbf{C_{Ar}}$), 69.9 (1 C, -**C**H_2), 54.7 (1 C, -**C**H), 50.1 (1 C, -**C**H), 34.4 (2 C, -**C**$(CH_3)_3$), 30.3 (6 C, -C(**C**$H_3)_3$). *Signals for the trans diastereomer:* 172.0 (1 C, -**C**OOR), 152.6 (1 C, $\mathbf{C_{Ar}}$), 139.6 (1 C, $\mathbf{C_{Ar}}$), 137.3 (1 C, $\mathbf{C_{Ar}}$), 135.9 (2 C, $\mathbf{C_{Ar}}$), 135.2 (1 C, $\mathbf{C_{Ar}}$), 134.1 (1 C, $\mathbf{C_{Ar}}$), 134.0 (1 C, $\mathbf{C_{Ar}}$), 132.4 (1 C, $\mathbf{C_{Ar}}$), 130.4 (2 C, $\mathbf{C_{Ar}}$), 129.7 (1 C, $\mathbf{C_{Ar}}$), 128.4 (1 C, $\mathbf{C_{Ar}}$), 127.7 (2 C, $\mathbf{C_{Ar}}$), 127.6 (1 C, $\mathbf{C_{Ar}}$), 124.9 (2 C, $\mathbf{C_{Ar}}$), 69.4 (1 C, -**C**H_2), 55.8 (1 C, -**C**H), 53.4 (1 C, -**C**H), 34.3 (2 C, -**C**$(CH_3)_3$), 30.2 (6 C, -C(**C**$H_3)_3$).

Naphtho[c]oxepinone derivative (6ca)

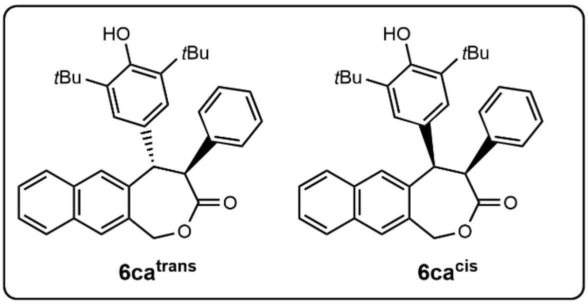

7-membered ring **6ca** was synthesized according to general procedure **F** and obtained as a colorless residue in a yield of 15% (20% for the alkylation step) as a mixture of diastereomers (*dr* 50:50; $er_{trans'}$ 93:7 er_{cis} 95:5).

Data for the alkylation product (mixture of diastereomers) 5ca:

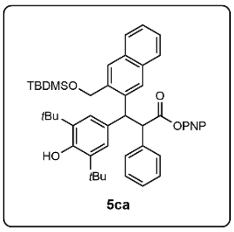

^{1}H-NMR (500 MHz, $CDCl_3$, 298 K) *δ* / ppm = *Signals for the unlike diastereomer:* 8.12 (d, *J* = 9.1 Hz, 2 H, Ar-**H**), 7.89-7.87 (m, 1 H, Ar-**H**), 7.85 (s, 1 H, Ar-**H**), 7.80-7.72 (m, 2 H, Ar-**H**), 7.58-7.51 (m, 3 H, Ar-**H**), 7.43-7.38 (m, 1 H, Ar-**H**) 7.31 (s, 2 H, Ar-**H**), 7.29-7.14 (m, 3 H, Ar-**H**), 6.64 (d, *J* =9.1 Hz, 2 H, Ar-**H**), 5.19 (d, *J* = 12.2 Hz, 1 H, -C**H**$_2$), 5.18 (s, 1 H, -O**H**), 5.03-4.92 (m, 2 H, 1 x -C**H**, 1 x -C**H**$_2$), 4.76 (d, *J* = 13.4 Hz, 1 H, -C**H**), 1.39 (s, 18 H, -C**H**$_3$), 1.00 (s, 9 H, -SiC(C**H**$_3$)$_3$), 0.16 (s, 3 H, -SiC**H**$_3$), 0.12 (s, 3 H, -SiC**H**$_3$). *Selected signals for the like diastereomer:* 8.19 (s, 1 H, Ar-**H**), 8.05 (d, *J* = 9.1 Hz, 2 H, Ar-**H**), 7.87 (s, 1 H, Ar-**H**), 7.95-9.94 (m, 1 H, Ar-**H**), 7.80-7.72 (m, 2 H, Ar-**H**), 7.58-7.51 (m, 1 H, Ar-**H**), 7.43-7.38 (m, 2 H, Ar-**H**), 7.29-7.14 (m, 3 H, Ar-**H**), 6.78 (d, *J* = 9.1 Hz, 2 H, Ar-**H**), 6.60 (s, 2 H, Ar-**H**), 5.03-4.92 (m, 2 H, 1 x -C**H**, 1 x -O**H**), 4.78 (d, *J* = 11.9 Hz, 1 H, -C**H**$_2$), 4.69 (d, *J* = 13.9 Hz, 1 H, -C**H**), 4.61 (d, *J* = 11.9 Hz, 1 H, -C**H**$_2$), 1.20 (s, 18 H, -C**H**$_3$), 0.90 (s, 9 H, -SiC(C**H**$_3$)$_3$), -0.01 (s, 3 H, -SiC**H**$_3$), -0.03 (s, 3 H, -SiC**H**$_3$).

^{13}C-NMR (125 MHz, $CDCl_3$, 298 K) *δ* / ppm = *Signals for the unlike diastereomer:* 171.2 (1 C, -**C**OOR), 155.5 (1 C, **C**$_{Ar}$), 152.9 (1 C, **C**$_{Ar}$), 145.5 (1 C, **C**$_{Ar}$), 137.6 (1 C, **C**$_{Ar}$), 136.6 (1 C, **C**$_{Ar}$), 166.1 (2 C, **C**$_{Ar}$), 132.1 (1 C, **C**$_{Ar}$), 131.6 (1 C, **C**$_{Ar}$), 129.6 (1 C, **C**$_{Ar}$), 128.8 (2 C, **C**$_{Ar}$), 128.7 (2 C, **C**$_{Ar}$), 127.9 (1 C, **C**$_{Ar}$), 127.9 (1 C, **C**$_{Ar}$), 127.6 (1 C, **C**$_{Ar}$), 127.5 (1 C, **C**$_{Ar}$), 126.1 (1 C, **C**$_{Ar}$), 126.1 (1 C, **C**$_{Ar}$), 125.8 (2 C, **C**$_{Ar}$), 125.7 (2 C, **C**$_{Ar}$), 125.1 (2 C, **C**$_{Ar}$), 122.6 (2 C, **C**$_{Ar}$), 63.6 (1 C, -**C**H$_2$), 58.0 (1 C, -**C**H), 48.6 (1 C, -**C**H), 34.5 (2 C, -**C**(CH$_3$)$_3$), 30.4 (6 C, -C(**C**H$_3$)$_3$), 26.2 (3 C, -SiC(**C**H$_3$)$_3$), 18.7 (1 C, -Si**C**(CH$_3$)$_3$), -5.1 (1 C, -Si**C**H$_3$), -5.1 (1 C, -Si**C**H$_3$). *Selected signals for the like diastereomer:* 170.8 (1 C, -**C**OOR), 155.4 (1 C, **C**$_{Ar}$), 152.4 (1 C, **C**$_{Ar}$), 145.4 (1 C, **C**$_{Ar}$), 135.4 (2 C, **C**$_{Ar}$), 128.9 (2 C, **C**$_{Ar}$), 128.7 (2 C, **C**$_{Ar}$), 127.4 (1 C, **C**$_{Ar}$), 126.0 (1 C, **C**$_{Ar}$), 125.8 (2 C, **C**$_{Ar}$), 125.5 (2 C, **C**$_{Ar}$), 125.2 (2 C, **C**$_{Ar}$), 122.5 (2 C, **C**$_{Ar}$), 63.1 (1 C, -**C**H$_2$), 58.4 (1 C, -**C**H), 51.1 (1 C, -**C**H), 34.2 (2 C, -**C**(CH$_3$)$_3$), 30.2 (6 C, -**C**(CH$_3$)$_3$), 26.1 (3 C, -SiC(**C**H$_3$)$_3$), 18.6 (1 C, -Si**C**(CH$_3$)$_3$), -5.2 (1 C, -Si**C**H$_3$), -5.3 (1 C, -Si**C**H$_3$)$_3$).

HRMS (ESI-TOF):): *m/z:* $[M+Na]^+$ calcd for $C_{46}H_{55}NNaO_6Si^+$: 768.3691, found 768.3696.

Data for the cyclic product 6ca:

HPLC (YMC-SB, *n*-hexane/IPA = 10/1, flow = 1.0 mL min^{-1}, T_{Column} = 10 °C, *l* = 240 nm) t_r*(trans):* 19.84 min (major), 42.74 min (minor); t_r*(cis):* 22.77 min (major), 46.60 min (minor).

α_D^{20}(c = 1, $CHCl_3$): -4.7 (mixture of diastereomers).

HRMS (ESI-TOF): *m/z*: $[M+NH_4]^+$ calcd for $C_{34}H_{40}NO_3^+$: 510.3003, found 510.3000.

Unfortunately, the diastereomers were not separable. NMR data is given for the mixture of diastereomers (obtained from the racemic sample, cis:trans = 70:30*):*

^{1}H-NMR (300 MHz, $CDCl_3$, 298 K) *δ* / ppm = *Signals for the cis diastereomer:* 7.86-7.82 (m, 2 H, Ar-**H**), 7.72-7.65 (m, 1 H, Ar-**H**), 7.69 (s, 1 H, Ar-**H**), 7.49-7.43 (m, 2 H, Ar-**H**), 7.31-7.19 (m, 3 H, Ar-**H**), 7.07-7.04 (m, 2 H, Ar-**H**), 6.64 (s, 2 H, Ar-**H**), 6.04 (d, 15.1 Hz, 1 H, -C**H$_2$**), 5.44 (d, *J* = 15.1 Hz, 1 H, -C**H$_2$**), 5.13 (s, 1 H, -O**H**), 5.04 (d, *J* = 3.2 Hz, 1 H, -C**H**), 4.56 (d, *J* = 3.2 Hz, 1 H, -C**H**), 1.32 (s, 18 H, -C**H$_3$**). Selected s*ignals for the trans diastereomer:* 7.86-7.82 (m, 2 H, Ar-**H**), 7.72-7.65 (m, 2 H, Ar-**H**), 7.49-7.43 (m, 2 H, Ar-**H**), .31-7.19 (m, 5 H, Ar-**H**), 6.56 (s, 2 H, Ar-**H**), 5.94 (d, *J* = 13.4 Hz, 1 H, -C**H$_2$**), 5.31 (d, *J* = 13.4 Hz, 1 H, -C**H$_2$**), 5.02 (s, 1 H, -O**H**), 4.79 (d, *J* = 9.7 Hz, 1 H,-C**H**), 4.56 (d, *J* = 9.7 Hz, 1 H, -C**H**), 1.24 (s, 18 H, -C**H$_3$**).

^{13}C-NMR (75 MHz, $CDCl_3$, 298 K) *δ* / ppm = *Signals for the cis diastereomer:* 172.2 (1 C, -**C**OOR), 153.1 (1 C, **C$_{Ar}$**), 138.8 (1 C, **C$_{Ar}$**), 138.1 (1 C, **C$_{Ar}$**), 136.9 (1 C, **C$_{Ar}$**), 135.2 (2 C, **C$_{Ar}$**), 133.5 (1 C, **C$_{Ar}$**), 131.8 (1 C, **C$_{Ar}$**), 131.7 (1 C, **C$_{Ar}$**), 131.3 (1 C, **C$_{Ar}$**), 130.9 (2 C, **C$_{Ar}$**), 129.6 (1 C, **C$_{Ar}$**), 128.4 (1 C, **C$_{Ar}$**), 127.8 (2 C, **C$_{Ar}$**), 127.5 (1 C, **C$_{Ar}$**), 127.1 (2 C, **C$_{Ar}$**), 126.7 (1 C, **C$_{Ar}$**), 126.4 (1 C, **C$_{Ar}$**), 124.8 (1 C, **C$_{Ar}$**), 70.9 (1 C, -**C**H$_2$), 55.3 (1 C, -**C**H), 50.5 (1 C, -**C**H), 34.4 (2 C, -**C**(CH$_3$)$_3$), 30.3 (1 C, -C(**C**H$_3$)$_3$). *Selected signals for the trans diastereomer:* 172.5 (1 C, -**C**OOR), 152.4 (1 C, **C$_{Ar}$**), 138.1 (1 C, **C$_{Ar}$**), 135.8 (2 C, **C$_{Ar}$**), 134.9 (1 C, **C$_{Ar}$**), 133.9 (1 C, **C$_{Ar}$**), 132.0 (1 C, **C$_{Ar}$**), 132.0 (1 C, **C$_{Ar}$**), 131.3 (1 C, **C$_{Ar}$**), 131.1 (2 C, **C$_{Ar}$**), 128.9 (1 C, **C$_{Ar}$**), 127.7 (2 C, **C$_{Ar}$**), 127.6 (2 C, **C$_{Ar}$**), 127.5 (1 C, **C$_{Ar}$**), 126.9 (1 C, **C$_{Ar}$**), 126.5 (1 C, **C$_{Ar}$**), 70.6 (1 C, -**C**H$_2$), 56.8 (1 C, -**C**H), 54.2 (1 C, -**C**H), 34.3 (2 C, -**C**(CH$_3$)$_3$), 30.2 (1 C, -C(**C**H$_3$)$_3$).

5-(3-(tert-butyl)-4-hydroxy-5-methylphenyl)-4-phenyl-4,5-dihydrobenzo[c]oxepin-3(1H)-one (6da)

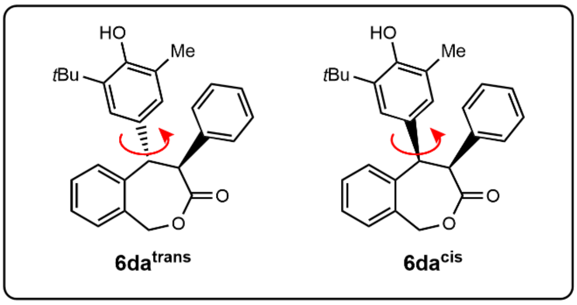

7-membered ring **6da** was synthesized according to general procedure **F** and obtained as a colorless residue in a yield of 45% (49% for the alkylation step) as a mixture of atropisomers (*dr* cis^1:cis^2:trans^1:trans^2 40:25:30:5; $er_{cis}{}^1$ 94:6, $er_{cis}{}^2$ 96:4, $er_{trans}{}^1$ 96:4, $er_{trans}{}^2$ 92:8).

Data for the alkylation product (mixture of diastereomers) 5da:

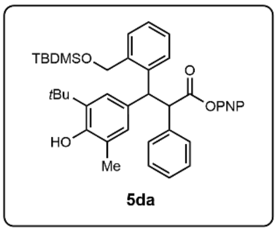

The integrals never really fitted to two pairs of diastereomers, probably already here the presence of atropisomers might be an issue.

^{1}H-NMR (300 MHz, $CDCl_3$, 298 K) δ / ppm = *Signals for the unlike diastereomer:* 8.13 (d, J = 9.1 Hz, 2 H, Ar-**H**), 7.46-7.03 (m, 11 H, Ar-**H**), 6.75 (d, J = 9.1 Hz, 2 H, Ar-**H**), 5.09-3.92 (m, 5 H, -C**H**, -C**H₂**, -O**H**), 2.19 (s, 3 H, -C**H₃**), 1.40 (s, 9 H, -C(C**H₃**)₃), 0.96 (s, 9 H, -SiC(C**H₃**)₃), 0.11 (s, 3 H, -SiC**H₃**), 0.10 (s, 3 H, -SiC**H₃**). Selected s*ignals for the like diastereomer:* 8.12 (d, J = 9.1 Hz, 2 H, Ar-**H**), 7.73-7.71 (m, 1 H, Ar-**H**), 7.54-7.50 (m, 1 H, Ar-**H**), 7.46-7.03 (m, 7 H, Ar-**H**), 6.66 (d, J = 9.1 Hz, 2 H, Ar-**H**), 6.60 (d, J = 2.0 Hz, 1 H, Ar-**H**), 6.45 (d, J = 2.0 Hz, 1 H, Ar-**H**), 5.09-3.92 (m, 5 H, -C**H**, -C**H₂**, -O**H**), 1.92 (s, 1 H, -C**H₃**), 1.22 (s, 9 H, -C(C**H₃**)₃), 0.88 (s, 9 H, -SiC(C**H₃**)₃), 0.02 (s, 3 H, -SiC**H₃**), -0.02 (s, 3 H, -SiC**H₃**).

^{13}C-NMR (125 MHz, $CDCl_3$, 298 K) δ / ppm = *Signals for the unlike diastereomer:* 171.1 (1 C, -**C**OOR), 155.5 (1 C, **C$_{Ar}$**), 151.8 (1 C, **C$_{Ar}$**), 145.5 (1 C, **C$_{Ar}$**), 138.9 (1 C, **C$_{Ar}$**), 138.3 (1 C, **C$_{Ar}$**), 136.0 (1 C, **C$_{Ar}$**), 135.5 (1 C, **C$_{Ar}$**), 132.7 (1 C, **C$_{Ar}$**), 129.9 (1 C, **C$_{Ar}$**), 128.9 (1 C, **C$_{Ar}$**), 128.8 (4 C, **C$_{Ar}$**), 127.9 (1 C, **C$_{Ar}$**), 127.5 (1 C, **C$_{Ar}$**), 127.2 (1 C, **C$_{Ar}$**), 126.8 (1 C, **C$_{Ar}$**), 126.5 (1 C, **C$_{Ar}$**), 125.5 (1 C, **C$_{Ar}$**), 125.1 (2 C, **C$_{Ar}$**), 122.5 (2 C, **C$_{Ar}$**), 63.3 (1 C, -**C**H₂), 57.8 (1 C, -**C**H), 47.9 (1 C, -**C**H), 34.7 (1 C, -**C**(CH₃)₃), 29.9 (3 C, -C(**C**H₃)₃), 26.1 (3 C, -SiC(**C**H₃)₃), 18.6 (1 C, -Si**C**(CH₃)₃), 16.3 (1 C, -**C**H₃), -5.1 (1 C, -Si**C**H₃), -5.1 (1 C, -Si**C**H₃). Selected s*ignals for the like diastereomer:* 170.8 (1 C, -**C**OOR), 155.2 (1 C, **C$_{Ar}$**), 151.2 (1 C, **C$_{Ar}$**), 145.7 (1 C, **C$_{Ar}$**), 139.5 (1 C, **C$_{Ar}$**), 139.5 (1 C, **C$_{Ar}$**), 136.5 (1 C, **C$_{Ar}$**), 135.8 (1 C, **C$_{Ar}$**), 133.1 (1 C, **C$_{Ar}$**), 130.7 (1 C, **C$_{Ar}$**), 123.6 (2 C, **C$_{Ar}$**), 122.9 (2 C, **C$_{Ar}$**), 62.8 (1 C, -**C**H₂), 58.1 (1 C, -**C**H), 50.1 (1 C, -**C**H), 34.3 (1 C, -**C**(CH₃)₃), 29.6 (3 C, -C(**C**H₃)₃), 26.1 (3 C, -SiC(**C**H₃)₃), 18.5 (1 C, -Si**C**(CH₃)₃), 16.2 (1 C, -**C**H₃), -5.3 (1 C, -Si**C**H₃), -5.3 (1 C, -Si**C**H₃).

HRMS (ESI-TOF):): *m/z:* $[M+Na]^+$ calcd for $C_{39}H_{47}NNaO_6Si^+$: 676.3065, found 676.3055.

Data for the cyclic product 6da:

HPLC:

<u>*Data for the cis Atropisomers*</u> (YMC-SB, *n*-hexane/IPA = 10/1, flow = 1.0 mL min^{-1}, T_{Column} = 10 °C, *l* = 220 nm): *t_r(major atropisomer):* 27.25 min (major), 31.29 min (minor); *t_r(minor atropisomer):* 81.43 min (major), 87.23 min (minor).

<u>*Data for the trans Atropisomers*</u> (YMC-SB, *n*-hexane/IPA = 4/1, flow = 1.0 mL min^{-1}, T_{Column} = 10 °C, *l* = 220 nm): *t_r(major atropisomer):* 12.79 min (major), 24.89 min (minor); *t_r(minor atropisomer):* 30.26 min (major), 46.00 min (minor).

HRMS (ESI-TOF): *m/z*: $[M+NH_4]^+$ calcd for $C_{27}H_{28}O_3$: 418.2377, found 418.2379.

NMR Data for the cis Atropisomers:

^{1}H-NMR (700 MHz, $CDCl_3$, 298 K) *δ* / ppm = *Signals for the major atropisomer:* 7.31-7.22 (m, 5 H, Ar-**H**), 7.06-7.01 (m, 4 H, Ar-**H**), 6.54-6.44 (m, 2 H, Ar-**H**), 5.92 (d, *J* = 15.1 Hz, 1 H, -C**H₂**), 5.22 (d, *J* = 15.2 Hz, 1 H, -C**H₂**), 4.95 (d, *J* = 3.3 Hz, 1 H, -C**H**), 4.71 (s, 1 H, -O**H**), 4.32 (d, *J* = 3.3 Hz, 1 H, -C**H**), 2.11 (s, 3 H, -C**H₃**), 1.28 (s, 9 H, -C(C**H₃**)₃). *Signals for the minor atropisomer:* 7.39 (d, *J* = 7.5 Hz, 2 H, Ar-**H**), 7.36 (t, *J* = 7.5 Hz, 2 H, Ar-**H**), 7.31-7.22 (m, 4 H, Ar-**H**), 7.06-7.01 (m, 1 H, Ar-**H**), 6.54-6.44 (m, 2 H, Ar-**H**), 5.91 (d, *J* = 15.1 Hz, 1 H, -C**H₂**), 5.20 (d, *J* = 15.1 Hz, 1 H, -C**H₂**), 4.97 (d, *J* = 3.3 Hz, 1 H, -C**H**), 4.35 (d, *J* = 3.3 Hz, 1 H, -C**H**), 3.87 (s, 1 H, -O**H**), 1.83 (s, 3 H, -C**H₃**), 1.12 (s, 9 H, -C(C**H₃**)₃).

^{13}C-NMR (175 MHz, $CDCl_3$, 298 K) *δ* / ppm = *Signals for the major atropisomer:* 172.1 (1 C, -**C**OOR), 152.1 (1 C, **C_{Ar}**), 147.7 (1 C, **C_{Ar}**), 141.2 (1 C, **C_{Ar}**), 136.7 (1 C, **C_{Ar}**), 134.3 (1 C, **C_{Ar}**), 132.6 (1 C, **C_{Ar}**), 132.3 (1 C, **C_{Ar}**), 130.9 (2 C, **C_{Ar}**), 129.9 (1 C, **C_{Ar}**), 128.8 (1 C, **C_{Ar}**), 128.3 (1 C, **C_{Ar}**), 128.2 (1 C, **C_{Ar}**), 127.8 (1 C, **C_{Ar}**), 127.7 (2 C, **C_{Ar}**), 127.7 (1 C, **C_{Ar}**), 126.7 (1 C, **C_{Ar}**), 70.7 (1 C, -**C**H_2), 54.7 (1 C, -**C**H), 50.0 (1 C, -**C**H), 34.5 (1 C, -**C**$(CH_3)_3$), 29.7 (3 C, -C(**C**$H_3)_3$), 16.2 (1 C, -**C**H_3). *Selected signals for the minor atropisomer:* 171.9 (1 C, -**C**OOR), 140.4 (1 C, **C_{Ar}**), 137.2 (1 C, **C_{Ar}**), 136.5 (1 C, **C_{Ar}**), 133.2 (1 C, **C_{Ar}**), 131.6 (1 C, **C_{Ar}**), 130.8 (2 C, **C_{Ar}**), 129.6 (1 C, **C_{Ar}**), 129.1 (1 C, **C_{Ar}**), 129.0 (1 C, **C_{Ar}**), 128.2 (1 C, **C_{Ar}**), 127.7 (2 C, **C_{Ar}**), 127.5 (1 C, **C_{Ar}**), 126.9 (1 C, **C_{Ar}**), 123.3 (1 C, **C_{Ar}**), 70.6 (1 C, -**C**H_2), 54.7 (1 C, -**C**H), 49.8 (1 C, -**C**H), 34.5 (1 C, -**C**$(CH_3)_3$), 30.3 (3 C, -(**C**$H_3)_3$), 14.3 (1 C, -**C**H_3).

NMR data for the trans Atropisomers:

^{1}H-NMR (700 MHz, $CDCl_3$, 298 K) δ / ppm = *Signals for the major atropisomer:* 7.36-7.07 (m, 9 H, Ar-**H**), 6.45-6.41 (m, 2 H, Ar-**H**), 5.83 (d, J = 13.8 Hz, 1 H, -C**H$_2$**), 5.05 (d, J = 13.8 Hz, 1 H, -C**H$_2$**), 7.59-7.51 (m, 3 H, 2 x -C**H**; 1 x -O**H**), 2.07 (s, 3 H, -C**H$_3$**), 1.18 (s, 9 H, -C(C**H$_3$**)$_3$). *Selected signals for the minor atropisomer:* 7.36-7.07 (m, 9 H, Ar-**H**), 6.45-6.41 (m, 2 H, Ar-**H**), 5.84 (d, J = 13.7 Hz, 1 H, -C**H$_2$**), 5.04 (d, J = 13.7 Hz, 1 H, -C**H$_2$**), (m, 2 H, 2 x -C**H**), 3.83 (s, 1 H, -O**H**), 1.78 (s, 3 H, -C**H$_3$**), 1.01 (s, 9 H, -C(C**H$_3$**)$_3$).

^{13}C-NMR (175 MHz, $CDCl_3$, 298 K) δ / ppm = *Signals for the major atropisomer:* 172.5 (1 C, -**C**OOR), 151.4 (1 C, **C$_{Ar}$**), 141.0 (1 C, **C$_{Ar}$**), 137.5 (1 C, **C$_{Ar}$**), 135.2 (1 C, **C$_{Ar}$**), 135.1 (1 C, **C$_{Ar}$**), 133.5 (1 C, **C$_{Ar}$**), 132.4 (1 C, **C$_{Ar}$**), 129.7 (1 C, **C$_{Ar}$**), 129.5 (1 C, **C$_{Ar}$**), 129.5 (2 C, **C$_{Ar}$**), 128.4 (2 C, **C$_{Ar}$**), 127.6 (1 C, **C$_{Ar}$**), 127.5 (1 C, **C$_{Ar}$**), 126.6 (1 C, **C$_{Ar}$**), 126.2 (1 C, **C$_{Ar}$**), 123.4 (1 C, **C$_{Ar}$**), 70.3 (1 C, -**C**H$_2$), 55.9 (1 C, -**C**H), 53.4 (1 C, -**C**H), 34.4 (1 C, -**C**(CH$_3$)$_3$), 29.6 (3 C, -C(**C**H$_3$)$_3$), 16.2 (1 C, -**C**H$_3$). *Selected signals for the minor atropisomer:* 172.2 (1 C, -**C**OOR), 140.7 (1 C, **C$_{Ar}$**), 137.2 (1 C, **C$_{Ar}$**), 129.8 (1 C, **C$_{Ar}$**), 128.8 (2 C, **C$_{Ar}$**), 128.4 (2 C, **C$_{Ar}$**), 128.1 (1 C, **C$_{Ar}$**), 127.6 (1 C, **C$_{Ar}$**), 127.2 (1 C, **C$_{Ar}$**), 34.4 (1 C, -**C**(CH$_3$)$_3$), 30.2 (3 C, -C(**C**H$_3$)$_3$), 14.3 (1 C, -**C**H$_3$).

6. Computational Details and Relative Configuration

6.1 Determination of the Relative Configuration of the Cyclic Products

As we were not able to obtain crystals suited for X-Ray diffraction analysis the relative configurations (cis/trans) of products **6** were assigned based on observed $^3J_{HH}$ couplings and comparison of experimental ^1H and ^{13}C NMR spectra with computed spectra of DFT optimized structures.

For the coupling constants we observed $^3J_{HH}$ couplings of approx. 3 Hz for the two vicinal protons for one isomer and larger couplings of approx. 10 Hz for the other. The dihedral angle for cis-**6a** was found to be around 70° (DFT optimized structure) while 170° were measured for trans-**6a**. Based on the well-established relationship between $^3J_{HH}$ couplings and dihedral angles (Karplus equation) [17], these results allowed us to assign cis-**6a** as the isomer with the smaller $^3J_{HH}$ coupling.

Observed $^3J_{HH}$ Couplings:

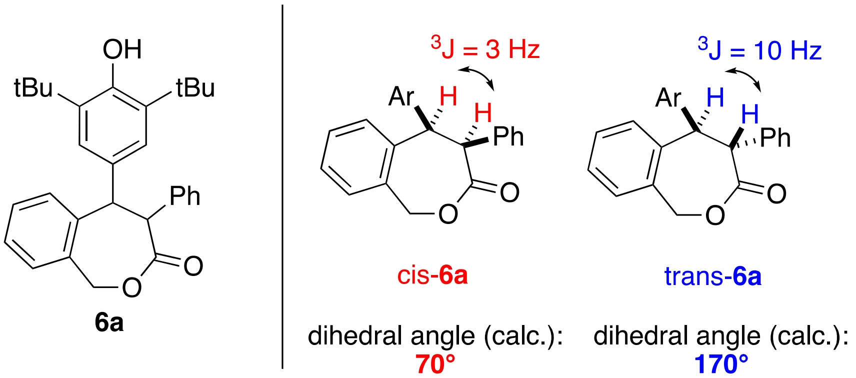

Figure 1: Observed $^3J_{HH}$ couplings and DFT-calculated dihedral angles.

In addition, the experimental NMR spectra were compared with computed spectra and here cis/trans fitted pretty well as well (as expected, deviations were larger in the ^1H NMR spectra while a better fit was obtained for the ^{13}C NMR shifts).

^{1}H-NMR (Experimental):

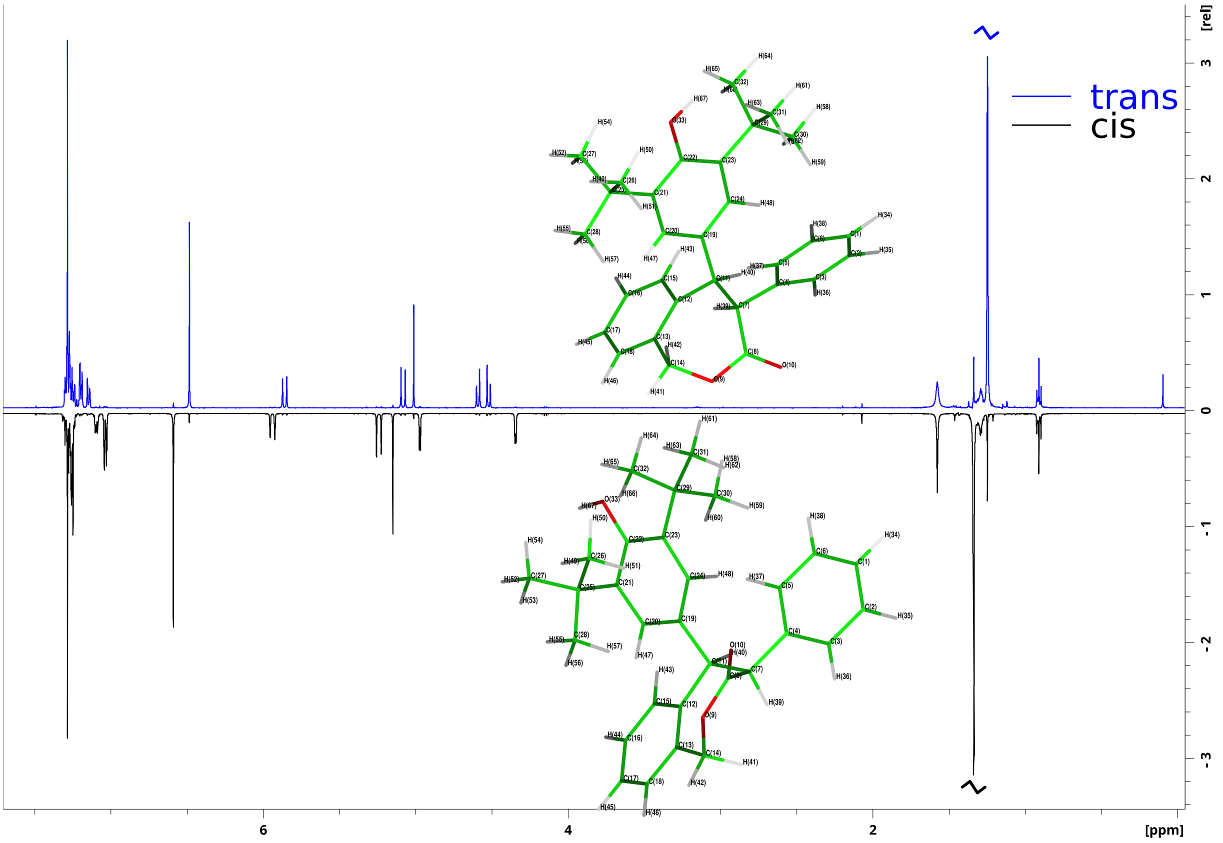

Figure 2: Experimental ^{1}H-NMR spectra for the trans- (blue) and the cis diastereomer (black).

1H-NMR of a DFT optimized structure:

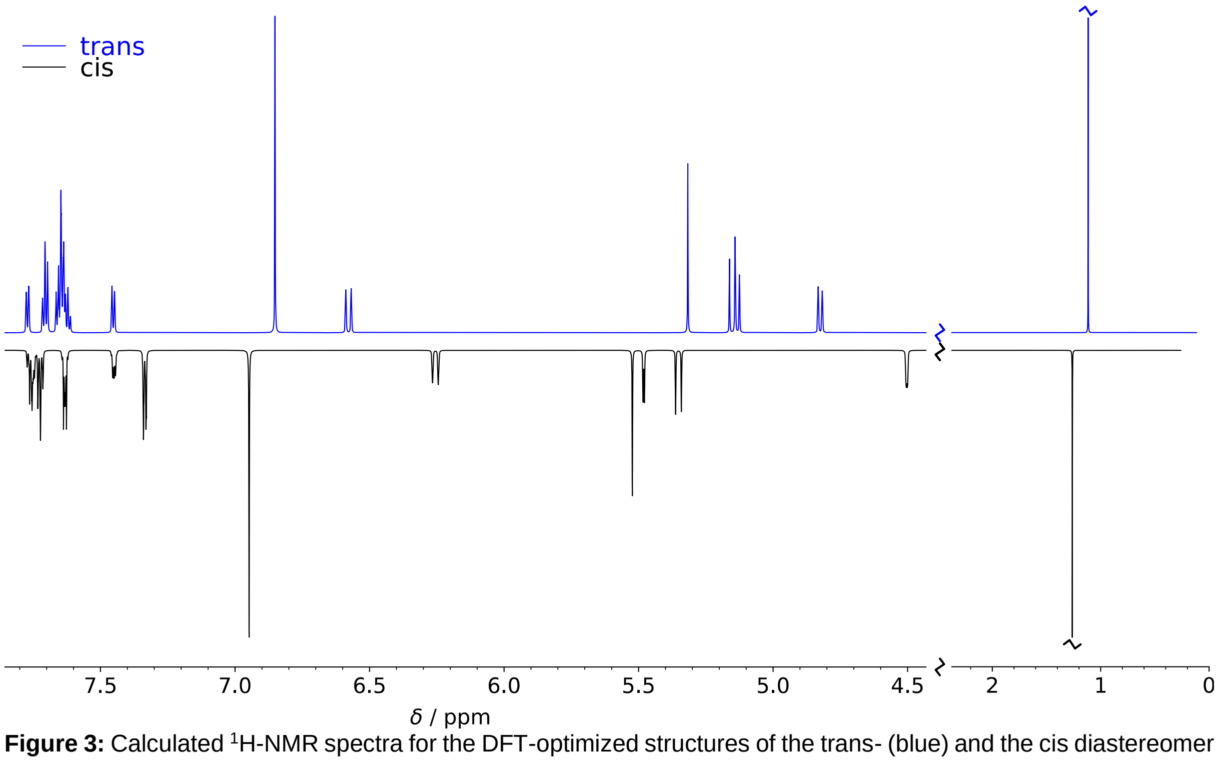

Figure 3: Calculated ^{1}H-NMR spectra for the DFT-optimized structures of the trans- (blue) and the cis diastereomer (black).

^{13}C-NMR (Experimental):

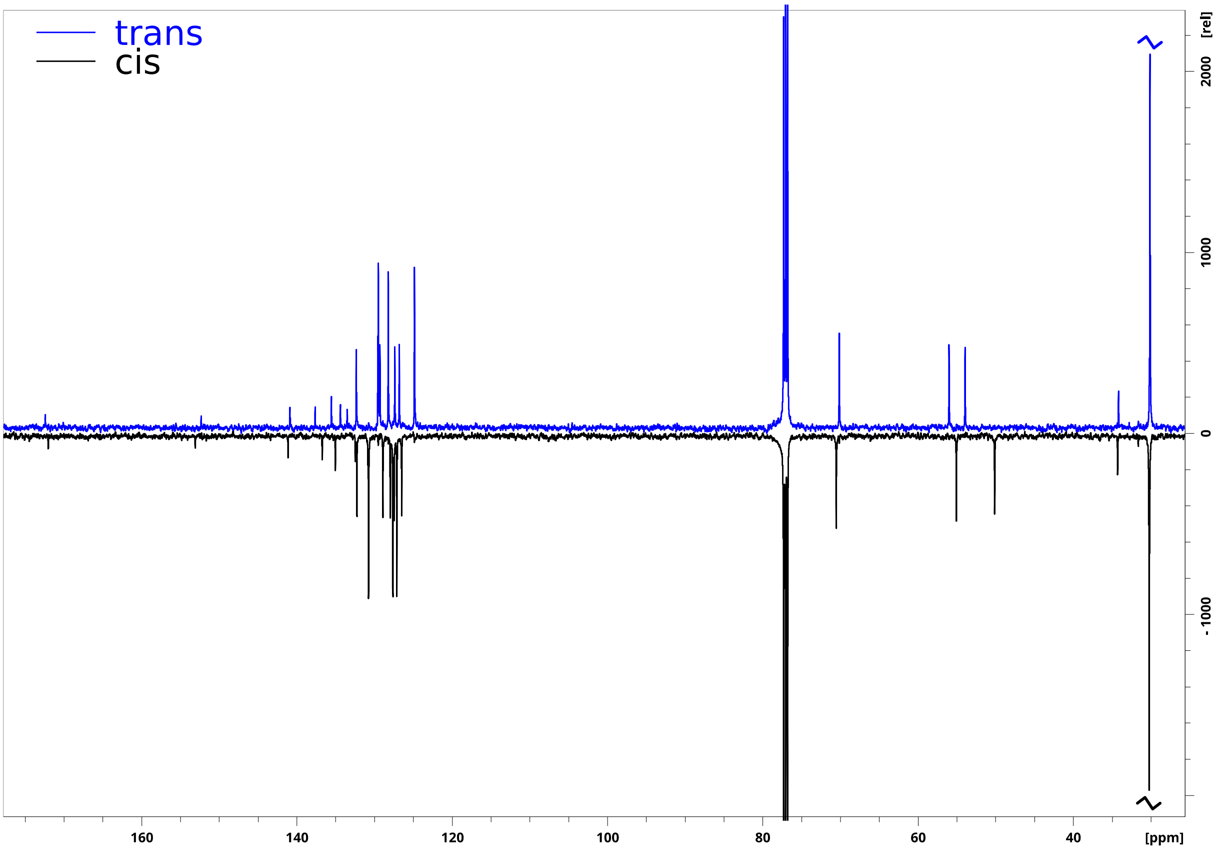

Figure 4: Experimental ^{13}C-NMR spectra for the trans- (blue) and the cis diastereomer (black).

^{13}C-NMR of a DFT optimized structure:

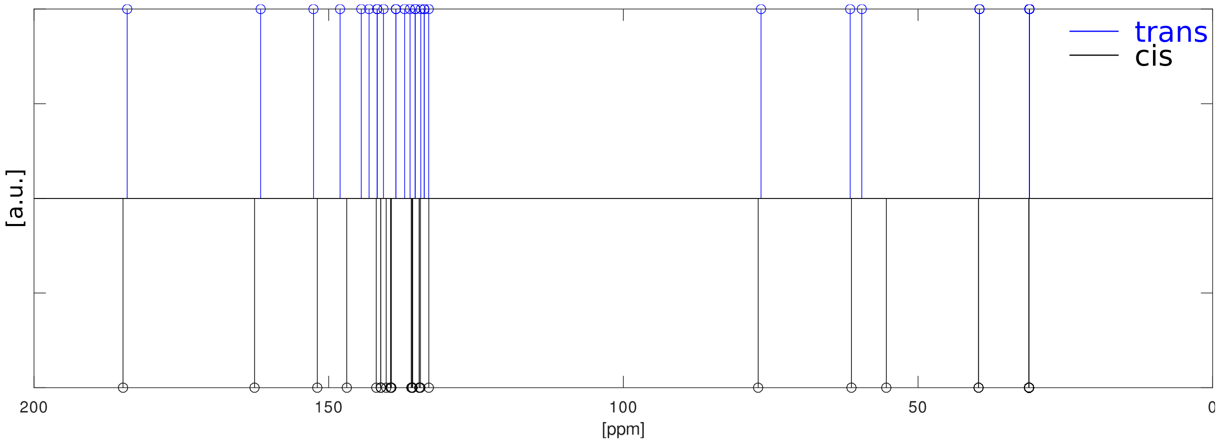

Figure 5: Calculated ^{13}C-NMR spectra for the DFT-optimized structures of the trans- (blue) and the cis diastereomer (black).

6.2 Computational Details

Initial geometries of **trans-6a** and **cis-6a** were generated by hand using the Avogadro software [18]. They are further optimized by using the GFN2-xTB semiempirical electronic structure method [19] and the analytical linearized Poisson-Boltzmann (ALPB) solvation model (chloroform) [20].

Conformer ensembles were obtained at the same level of theory using the utility/driver for the xTB software CREST version 2.12 (conformer-rotamer sampling tool [21,22]. The obtained ensembles for each species were further refined using Grimme's CENSO version 1.2.0 workflow [23]. NMR chemical shieldings are calculated using the DFT software package ORCA version 5.0.4 [24]. NMR chemical shifts and spin-spin couplings are ensemble averaged to construct full NMR spectra [25]. For the detailed parametrization of calculations see Table 1.

Calculated chemical shifts δ_c were generated according to

$$\delta_c = \sigma_{ref} - \sigma_c$$

where σ_{ref}, is the ^{1}H, ^{13}C shielding in tetramethyl silane (TMS), and σ_c in the compound of interest. TMS shieldings are calculated by the same CREST/CENSO workflow as the compound of interest.

Table 1: CREST and CENSO (short notation) parametrisation together with ORCA configuration for NMR shift and spin-spin coupling calculations.

Program	Task	Theory level	Solvation	Sorting threshold
CREST [21,22]	Conformation search	GFN2-xTB	ALPB	6.0 kcal mol^{-1}
CENSO [23]	Ensemble refinement	**Part 0**: b97-d3/def2-SV(P) // GFNn-xTB // CREST structures	ALPB	4.0 kcal mol^{-1}
		**Part 1**: r2scan-3c + SMD[chcl3] + GmRRHO(GFN2[alpb]-bhess) // GFNn-xTB	ALPB	3.5 kcal mol^{-1}
		**Part 2**:r2scan-3c + SMD[chcl3] + GmRRHO(GFN2[alpb]-bhess) // r2scan-3c[SMD]	SMD	2.5 kcal mol^{-1}
ORCA [24]	NMR shielding	pbe0-d4 def2-TZVP	SMD	
	NMR spin-spin	pbe0-d4 def2-TZVP	SMD	

Table 2: Structure of global minimum energy conformer of **trans-6a**, in Cartesian coordinates [angstrom].

C	-1.57083610101741	4.21336200257911	0.96225416171838
C	-0.59393322760829	4.37913766196039	-0.01012996166965
C	0.46888446401896	3.49410908559337	-0.09399587156156
C	0.57093950826863	2.42847030503343	0.79549396864979
C	-0.40875980761718	2.27365463232452	1.76833358552297
C	-1.47370141012573	3.15775668754059	1.85502491021002
C	1.65812548583497	1.39352772562187	0.67860006766785
C	3.05312214576826	1.99100160956883	0.72866028059939
O	4.03466704972830	1.17076883728830	1.15026566392287
O	3.32680315266020	3.11965722299024	0.44457741724287
C	1.44542226717086	0.53490698603994	-0.60153143573807
C	2.53819694314593	-0.45107943192639	-0.96301772330179
C	3.58628925694450	-0.84285882276940	-0.12816619751741
C	3.80702374150457	-0.23342512053400	1.22409101863984
C	2.44633117220343	-1.03008062547832	-2.22740812620150
C	3.34989262162711	-1.98196423830659	-2.65978148800754
C	4.38101053024936	-2.37997059032193	-1.82173710995319
C	4.49478682918647	-1.80204841798046	-0.57151411115784
C	0.11068785391547	-0.16495743671335	-0.47821923596462
C	-0.00636620358848	-1.33004532171149	0.26199074822820
C	-1.22906358305438	-1.93831958887209	0.50416223384093
C	-2.37131294895894	-1.31861463549123	-0.03388708058308
C	-2.29509511039285	-0.13960180205246	-0.79327038943191
C	-1.03315879096262	0.40353169408569	-1.00423759299149
C	-1.31565172175257	-3.22160557867742	1.33202793780997
C	-2.15657021842925	-2.98123266722882	2.59285088620462
C	-1.91997010996060	-4.35796202813602	0.49770380137737
C	0.06467621172501	-3.68760022712154	1.79331842165448
C	-3.54124577696068	0.57120842640315	-1.33347258697023
C	-3.17557793772176	1.84335754994244	-2.09885493301551
C	-4.44082752558616	1.02331264767439	-0.17072195914589
C	-4.31184903893605	-0.31730578994427	-2.32386330360200
O	-3.56748824913841	-1.91617384419840	0.22276029727751
H	-2.40059910093402	4.90652983592396	1.02453669040353
H	-0.65707865829023	5.20662476504437	-0.70602924433099
H	1.23306213724688	3.64139289066542	-0.84484891336521
H	-0.34899311458355	1.43773421899471	2.45591070880361
H	-2.22876536575660	3.01700055850978	2.61856034221430
H	1.54802704348782	0.72401800581177	1.53281297942792

H	1.37475793077669	1.24385219831345	-1.43159964971567
H	4.71923887323715	-0.63430515633287	1.66082098284118
H	2.99270555011614	-0.45591712145822	1.91843078536923
H	1.62895076468433	-0.73252578632370	-2.87444371836366
H	3.24964163228660	-2.41356800518289	-3.64771570700358
H	5.09827485783666	-3.12334055762765	-2.14530427607341
H	5.31101190872736	-2.08943360595592	0.08146753517300
H	0.89463774144460	-1.77943740607093	0.65572000494358
H	-0.93429727390552	1.32301331634007	-1.56168305236194
H	-2.19270111060930	-3.89385637768780	3.19276799370111
H	-3.17544059509366	-2.68957265808998	2.35133899536872
H	-1.70802795787930	-2.19473804721114	3.20375033540721
H	-1.95535673178548	-5.27297116309863	1.09393593957088
H	-1.30392559983950	-4.55363141358338	-0.38252104233983
H	-2.92886454602056	-4.12571482569048	0.16635385947938
H	-0.04857966841854	-4.60564276507793	2.37228428972731
H	0.72727992823034	-3.90512349810009	0.95331156821094
H	0.55131836883487	-2.95170142697140	2.43707975780146
H	-4.09148768366526	2.31775506426523	-2.45567768890827
H	-2.65442349507911	2.56042181331586	-1.46332880461653
H	-2.55215294442177	1.63094838766369	-2.96902554143369
H	-5.32159402628802	1.53777713280655	-0.56107811795810
H	-3.89209977772818	1.71875888529311	0.46620486559261
H	-4.79101159750535	0.21496265254790	0.47318276158872
H	-5.18375482073985	0.22104579535703	-2.70107590173088
H	-4.67292749370933	-1.26363516839917	-1.91559158526263
H	-3.67437053731803	-0.57097413328634	-3.17214778942423
H	-4.26308810947890	-1.42306531188646	-0.21078665648988

Table 3: Structure of global minimum energy conformer of **cis-6a**, in Cartesian coordinates [angstrom].

C	0.24393007703150	1.73047172195220	-4.77331458741668
C	1.48051734099559	1.10671689107445	-4.71415189156076
C	2.03801041751614	0.79834955038299	-3.48296750539434
C	1.37699944668312	1.10191857519005	-2.29604577105224
C	0.13670884288863	1.73164743921565	-2.36578376129395
C	-0.42017378691645	2.04320468498020	-3.59662105131758
C	2.00103161208700	0.64620521126443	-1.00335664642576
C	1.94332513722406	1.66501196827069	0.11776203924854
O	2.74053862825836	1.42404688387430	1.17916981147892
O	1.22701695758392	2.62103629409697	0.15631057078062
C	1.37812692480448	-0.70849169528351	-0.54198010276367
C	2.19387455515270	-1.49300869042874	0.46473736303452
C	3.24500291137905	-0.98822477179811	1.23132085858577
C	3.74776417767915	0.42243973220470	1.12764547633810
C	1.83248787756004	-2.82967351592177	0.63331566546244
C	2.48999067882955	-3.65719988742712	1.52221429981210
C	3.53378234736119	-3.15225317341120	2.28458726206621
C	3.89495487817656	-1.82711281979603	2.13718644388586
C	-0.05320489787979	-0.54338962955460	-0.09547432916538
C	-0.34915455497317	-0.05895443109892	1.16605807882448
C	-1.64780116130968	0.21962298608516	1.56510769922821
C	-2.67772049570794	-0.04031520129537	0.64287818526523
C	-2.42204219014975	-0.55210948477256	-0.63989856428361
C	-1.09558284985188	-0.79298752289444	-0.97180547776287
C	-1.91433372380677	0.82518934310251	2.94736572357268
C	-2.55294267844206	2.21756036549535	2.80769200126532
C	-2.78428076182858	-0.10683641201658	3.80546188531871
C	-0.61457879138006	1.03593512458733	3.72582777535487
C	-3.54065203554245	-0.79810231627313	-1.65392524747780
C	-2.99371047557459	-1.35059822500225	-2.96940142864365
C	-4.24827883748492	0.52322211594640	-1.98138969256925
C	-4.54819515597418	-1.81942227960744	-1.11145086516488
O	-3.97993933286651	0.20066516931754	0.95699114737903
H	-0.19757588655631	1.97564583941296	-5.73126412116977
H	2.01366242213267	0.86161988462164	-5.62433402887632
H	3.00625640171648	0.30997290299085	-3.44370768083294
H	-0.38724409630234	1.98376995246762	-1.45721431429677
H	-1.38421803944312	2.53563989343609	-3.63404457538313
H	3.05167986761619	0.43796453310419	-1.21920213744179

H	1.36017016362670	-1.31350501243252	-1.45282538982630
H	4.36460724075798	0.56615154679606	0.23564968016224
H	4.38242006944891	0.63542942689619	1.98595593729436
H	1.00440936135042	-3.21622697775151	0.05005786100580
H	2.18592410854713	-4.69125495179080	1.62498930491479
H	4.05371573487401	-3.78286603615419	2.99435722201626
H	4.69962612592727	-1.42066711533951	2.73971447817022
H	0.46457494464809	0.12319236767701	1.85209196861436
H	-0.85446960554508	-1.16404635200850	-1.95765576792169
H	-2.71111663579686	2.65238922235077	3.79688368102289
H	-3.51432165053296	2.23221185742976	2.29074284449014
H	-1.88598895201375	2.87406358171427	2.24711351660754
H	-2.95855052472847	0.34549955898649	4.78411433826374
H	-2.27182430984847	-1.05825399837135	3.95658556594753
H	-3.76119131195125	-0.34502469446176	3.38035649543395
H	-0.84976361316112	1.47480262811020	4.69700610041196
H	-0.09236721762295	0.09479000488756	3.90610766904765
H	0.06245342899539	1.71574673453198	3.20666116355820
H	-3.82486780841074	-1.50667136939349	-3.65929908413864
H	-2.29450812992541	-0.65949634256948	-3.44459240052406
H	-2.49363213822097	-2.31152752492475	-2.83153550570128
H	-5.04027897872309	0.34557873236131	-2.71305407740927
H	-3.54159149824655	1.23371636240419	-2.41562874132053
H	-4.69313206624340	0.97484193296059	-1.09814384360935
H	-5.32256671222736	-2.00456343410634	-1.85985293886010
H	-5.02781225202881	-1.47046649931728	-0.20078233489758
H	-4.05284296444210	-2.76909081203984	-0.89817309518671
H	-4.02818655919229	0.55921015706242	1.84245684582546

7. References

[1] V. B. Birman, X. Li, *Organic Letters* **2008**, *10* (*6)*, 1115–1118.

[2] P. A. Woods, L. C. Morrill, T. Lebl, A. M. Z. Slawin, R. A. Bragg, A. D. Smith, *Organic Letters* **2010**, *12* (*11)*, 2660–2663.

[3] C. McLaughlin, A. M. Z. Slawin, A. D. Smith, *Angewandte Chemie (Int. Ed.)* **2019**, *58* (*42)*, 15111–15119.

[4] C.-H. Yang, M. Han, W. Li, N. Zhu, Z. Sun, J. Wang, Z. Yang, Y.-M. Li, *Organic Letters* **2020**, *22* (*13)*, 5090–5093.

[5] K. Chojnacka, S. Santoro, R. Awartani, N. G. J. Richards, F. Himo, A. Aponick, *Organic & Biomolecular Chemistry* **2011**, *9* (*15)*, 5350–5353.

[6] W. K. Anderson, F. R. Kinder, *Journal of Heterocyclic Chemistry* **1990**, *27* (*4)*, 975–979.

[7] K. Zielke, O. Kováč, M. Winter, J. Pospíšil, M. Waser, *Chemistry - a European Journal* **2019**, *25* (*34)*, 8163–8168.

[8] L. Liu, Z. Yuan, R. Pan, Y. Zeng, A. Lin, H. Yao, Y. Huang, *Organic Chemistry Frontiers* **2018**, *5* (*4)*, 623–628.

[9] C. McLaughlin, J. Bitai, L. J. Barber, A. M. Z. Slawin, A. D. Smith, *Chemical Science* **2021**, *12* (*36)*, 12001–12011.

[10] J. N. Arokianathar, A. B. Frost, A. M. Z. Slawin, D. Stead, A. D. Smith, *ACS Catalysis* **2018**, *8* (*2)*, 1153–1160.

[11] O. O. Kovalenko, H. Adolfsson, *Chemistry - a European Journal* **2015**, *21* (*7)*, 2785–2788.

[12] A. C. D'Hollander, N. J. Westwood, *Tetrahedron* **2018**, *74* (*2)*, 224–239.

[13] O. Farooq, *Synthesis* **1994**, *1994* (*10)*, 1035–1036.

[14] F. Xu, L. Peng, K. Shinohara, T. Morita, S. Yoshida, T. Hosoya, A. Orita, J. Otera, *The Journal of Organic Chemistry* **2014**, *79* (*23)*, 11592–11608.

[15] J. N. Arokianathar, W. C. Hartley, C. McLaughlin, M. D. Greenhalgh, D. Stead, S. Ng, A. M. Z. Slawin, A. D. Smith, *Molecules* **2021**, *26* (*21)*, 6333.

[16] L. Stockhammer, R. Craik, U. Monkowius, D. B. Cordes, A. D. Smith, M. Waser, *ChemistryEurope* **2023**, *(1),* e202300015.

[17] M. Karplus, *Journal of the American Chemical Society* **1963**, *85* (*18)*, 2870–2871.

[18] M. D. Hanwell, D. E. Curtis, D. C. Lonie, T. Vandermeersch, E. Zurek, G. R. Hutchison, *Journal of Cheminformatics* **2012**, *4* (*1)*, 17.

[19] C. Bannwarth, E. Caldeweyher, S. Ehlert, A. Hansen, P. Pracht, J. Seibert, S. Spicher, S. Grimme, *WIREs Computational Molecular Science* **2021**, *11* (*2)*.

[20] S. Ehlert, M. Stahn, S. Spicher, S. Grimme, *Journal of Chemical Theory and Computation* **2021**, *17* (*7)*, 4250–4261.

[21] S. Grimme, *Journal of Chemical Theory and Computation* **2019**, *15* (*5)*, 2847–2862.

[22] P. Pracht, F. Bohle, S. Grimme, *Physical Chemistry Chemical Physics : PCCP* **2020**, *22* (*14)*, 7169–7192.

[23] S. Grimme, F. Bohle, A. Hansen, P. Pracht, S. Spicher, M. Stahn, *The Journal of Physical Chemistry. A* **2021**, *125* (*19)*, 4039–4054.

[24] F. Neese, *WIREs Computational Molecular Science* **2022**, *12* (*5)*.

[25] S. Grimme, C. Bannwarth, S. Dohm, A. Hansen, J. Pisarek, P. Pracht, J. Seibert, F. Neese, *Angewandte Chemie (Int. Ed.)* **2017**, *56* (*46)*, 14763–14769.

8. Appendix: NMR Spectra and HPLC Chromatograms

8.1 NMR Spectra for Unknown Starting Materials

4-Nitrophenyl 2-(2-fluorophenyl)acetate (3b)

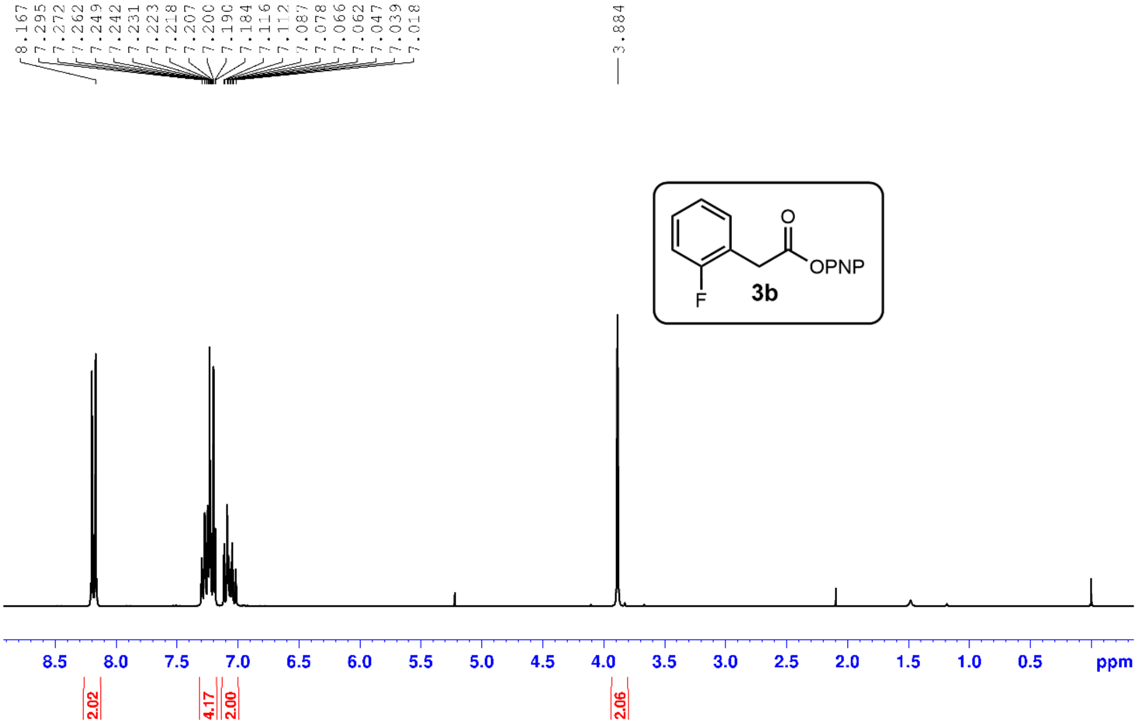

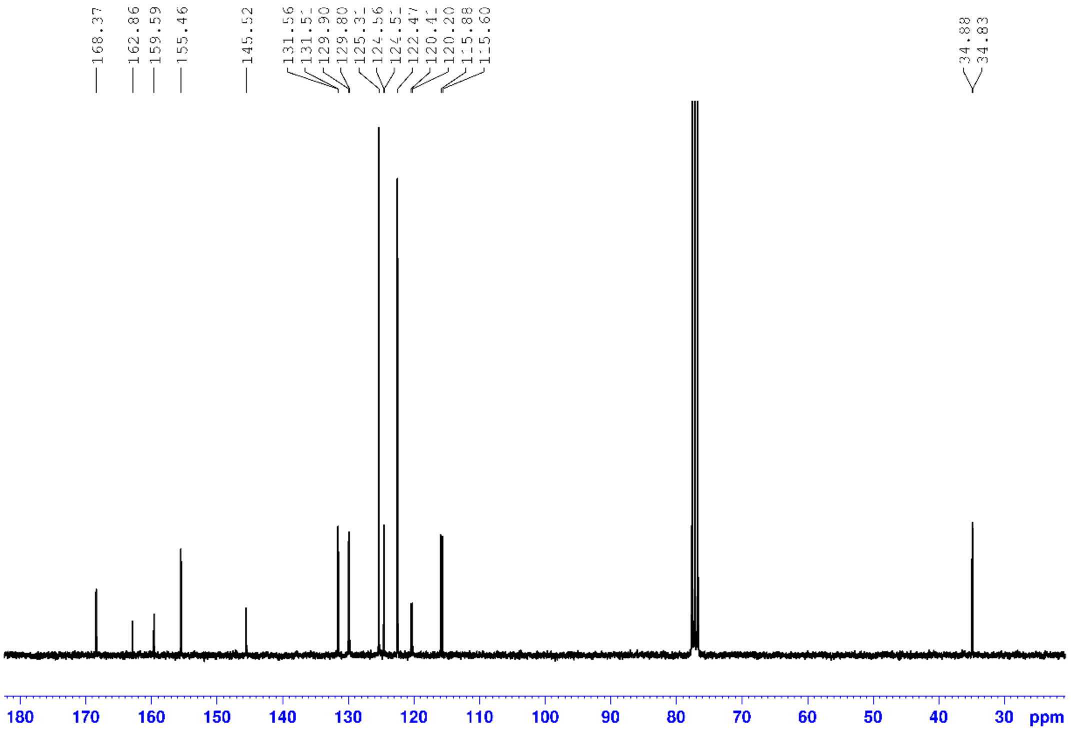

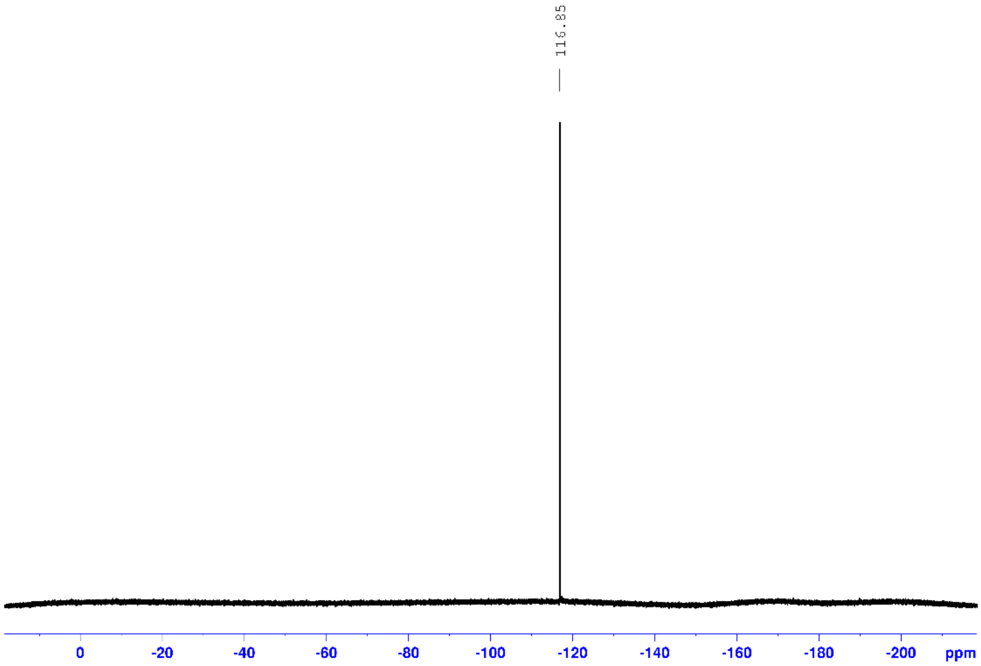
116.85
0
-20
-40
-60
-80
-100
-120
-140
-160
-180
-200
ppm

4-Nitrophenyl 2-(3-chlorophenyl)acetate (3d)

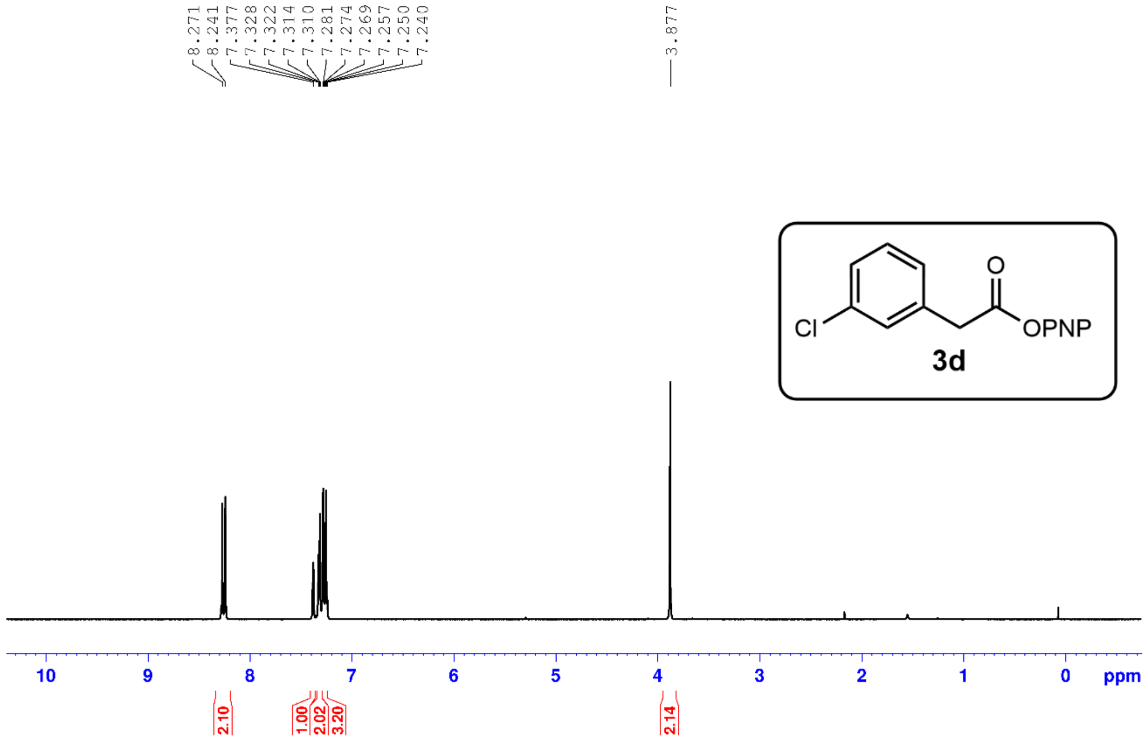

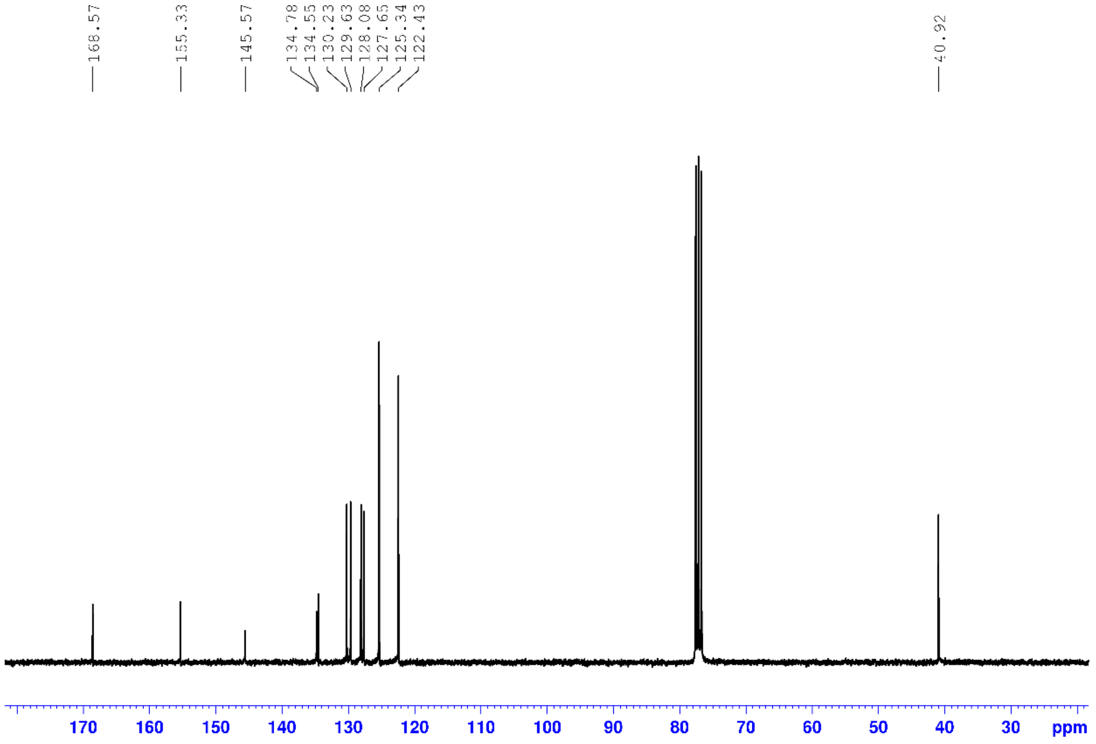

4-Nitrophenyl 2-(3,4-dichlorophenyl)acetate (3f)

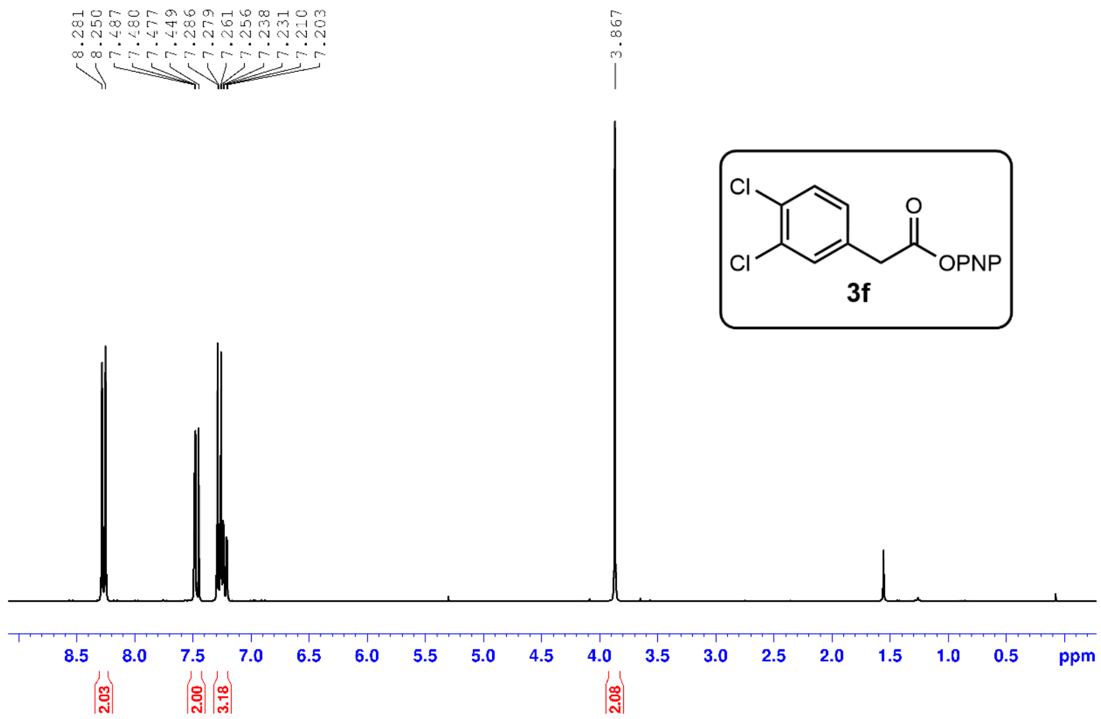

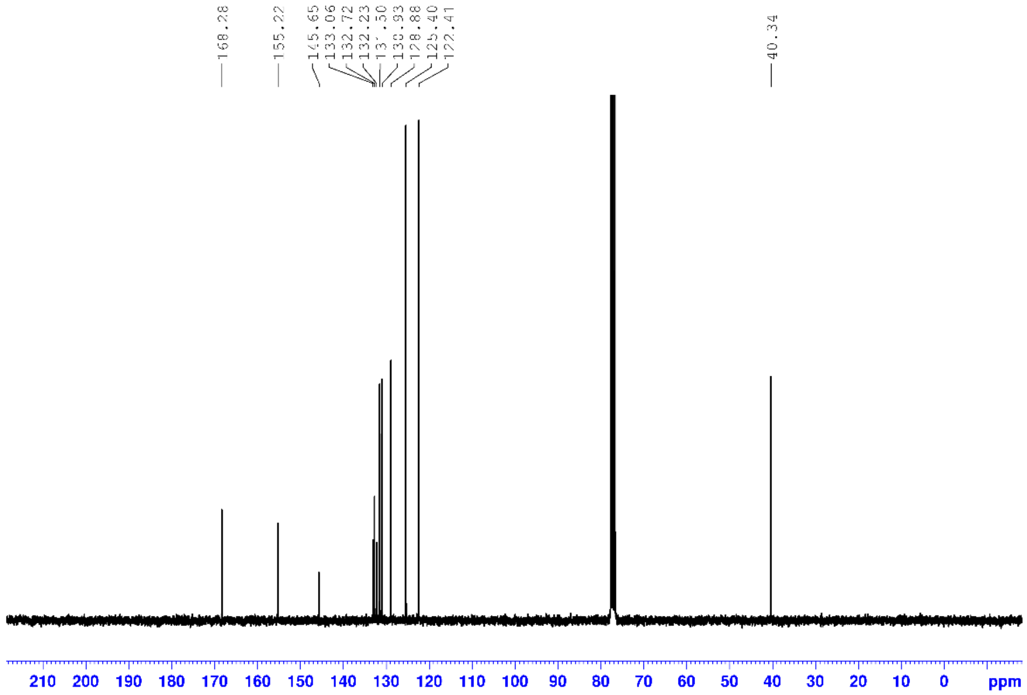

4-Nitrophenyl 2-(3-iodophenyl)acetate 3h

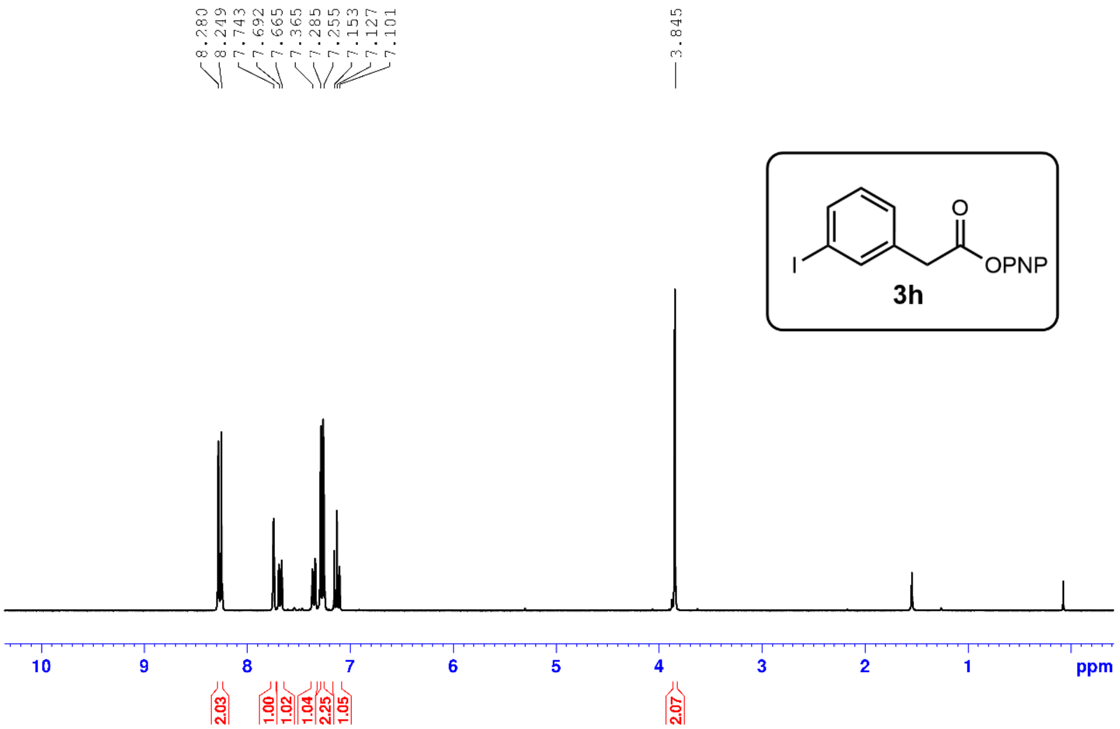

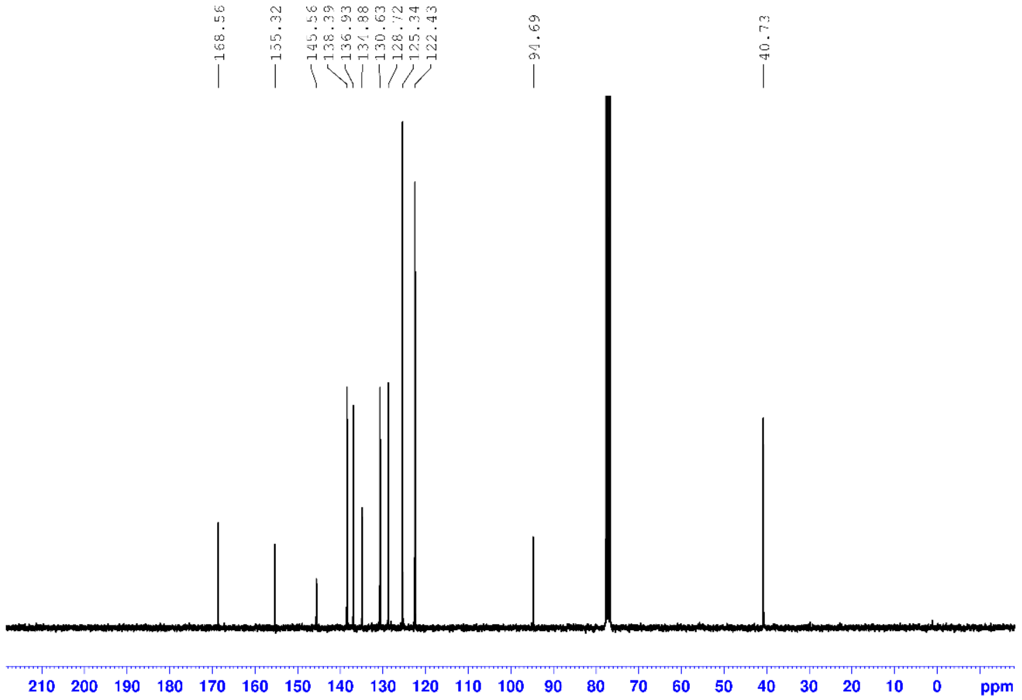

4-Nitrophenyl 2-(4-(methylthio)phenyl)acetate (3k)

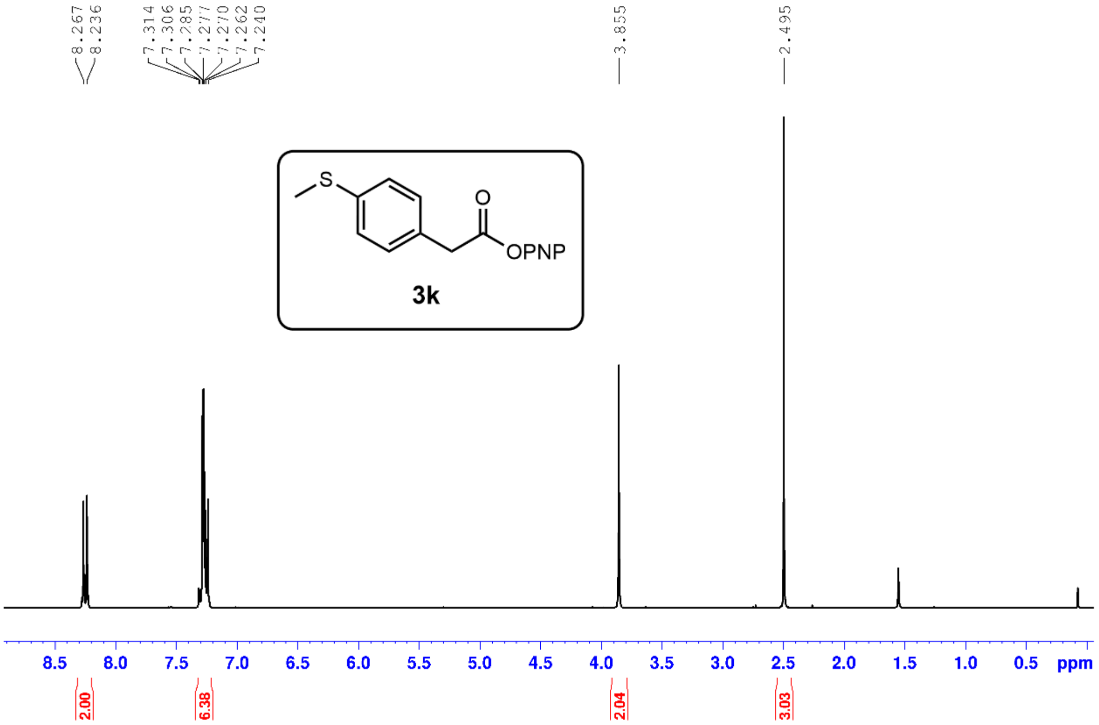

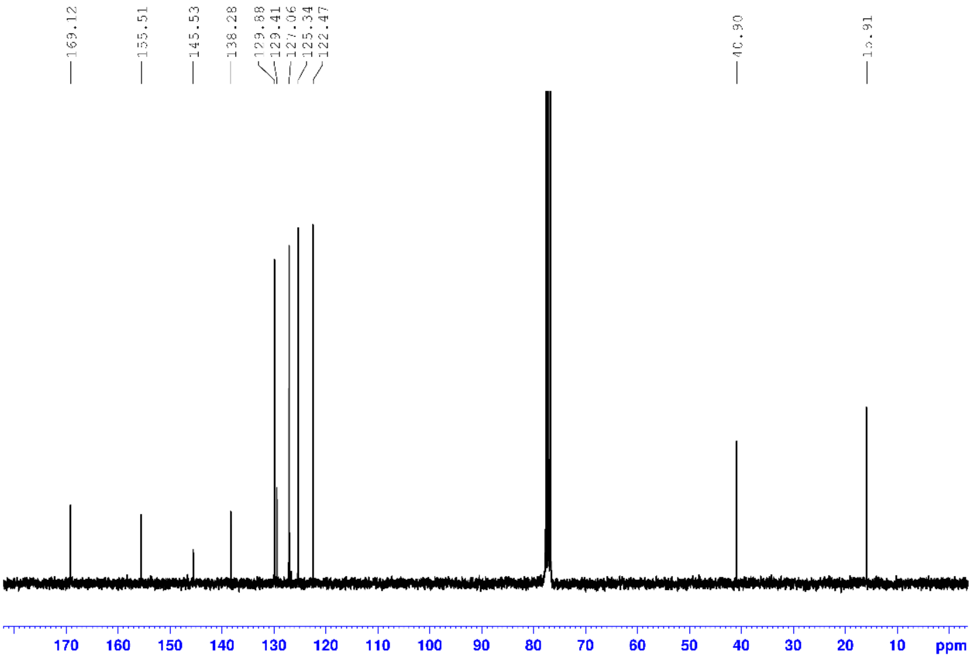

4-Nitrophenyl 2-(3,5-dimethylphenyl)acetate (3o)

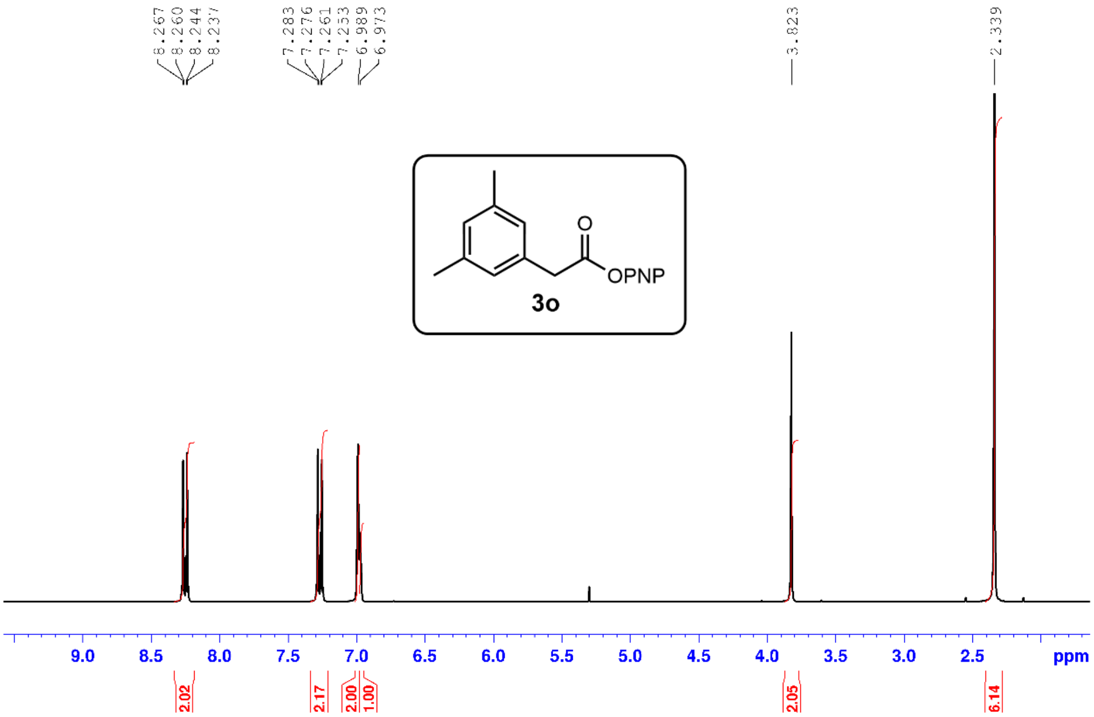

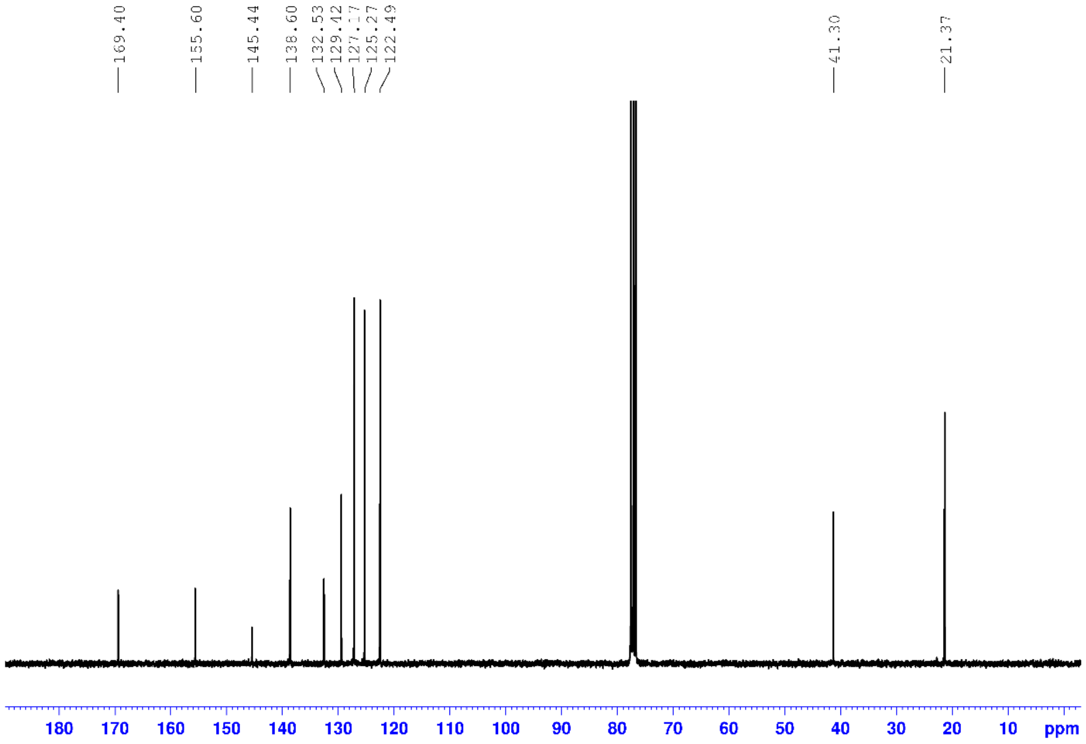

4-Nitrophenyl 2-(4-(tert-butyl)phenyl)acetate (3p)

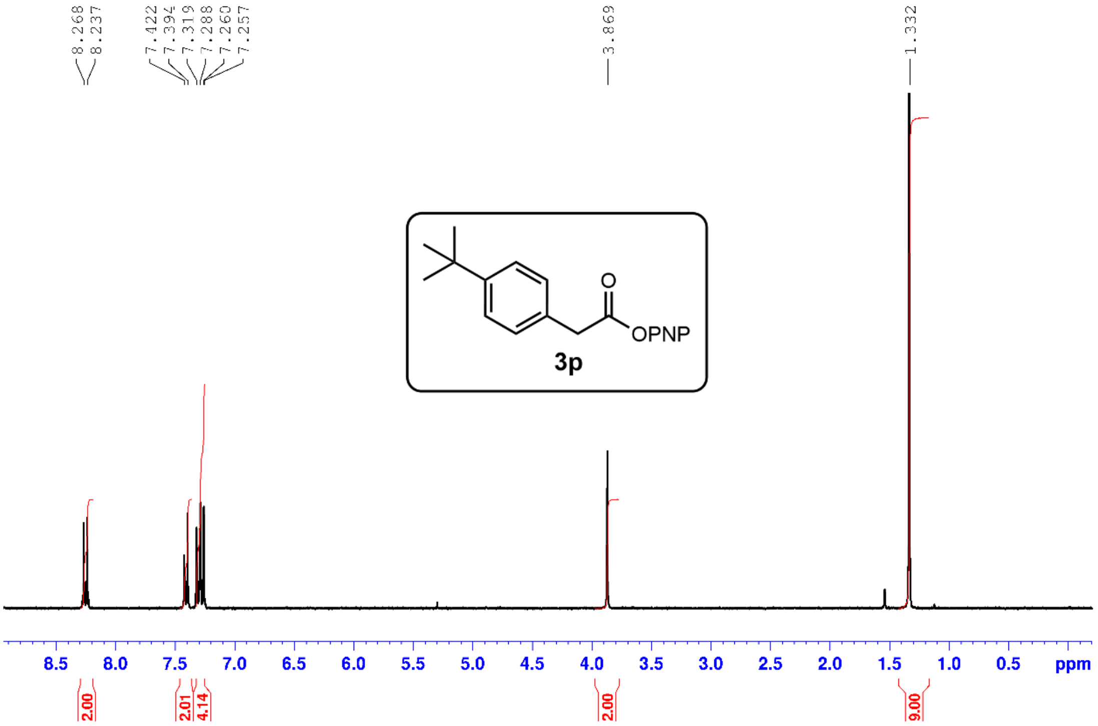

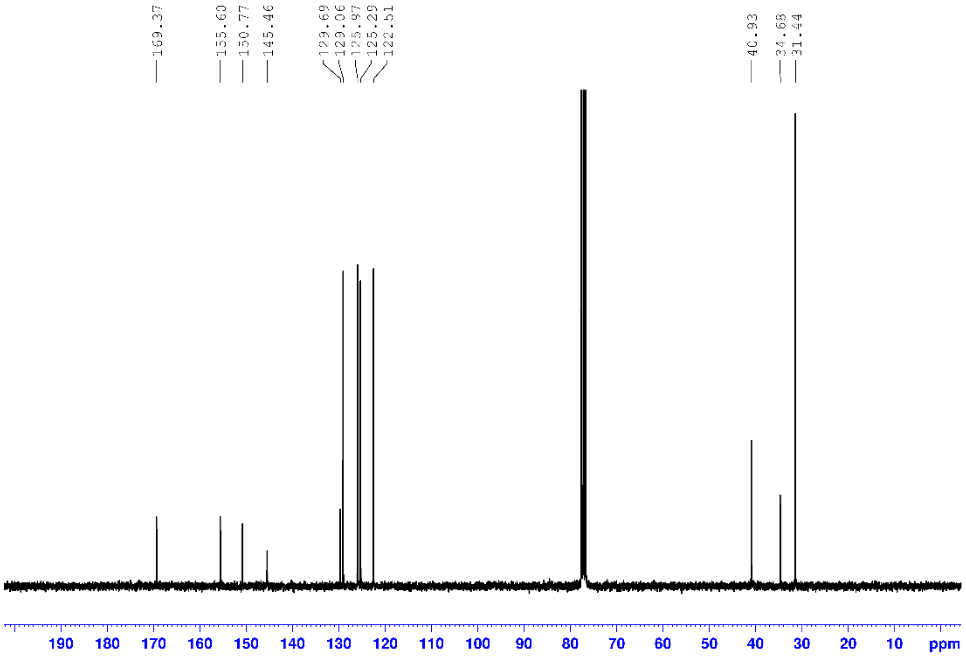

4-Nitrophenyl 2-(3-(trifluoromethyl)phenyl)acetate (3r)

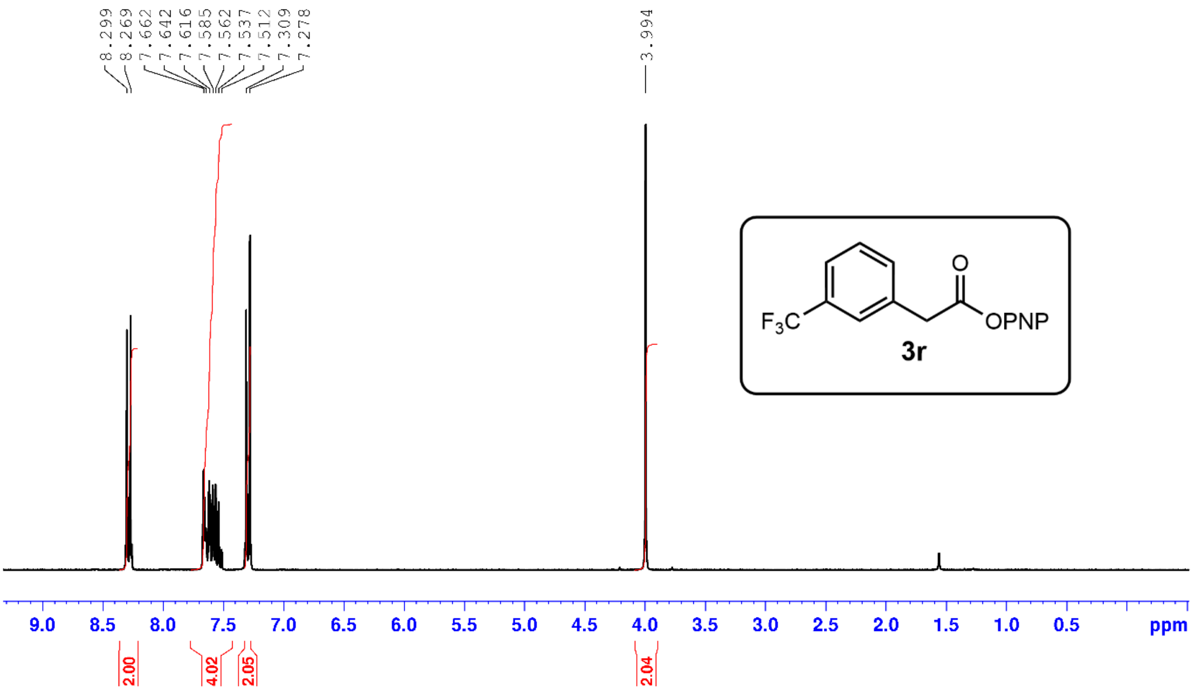

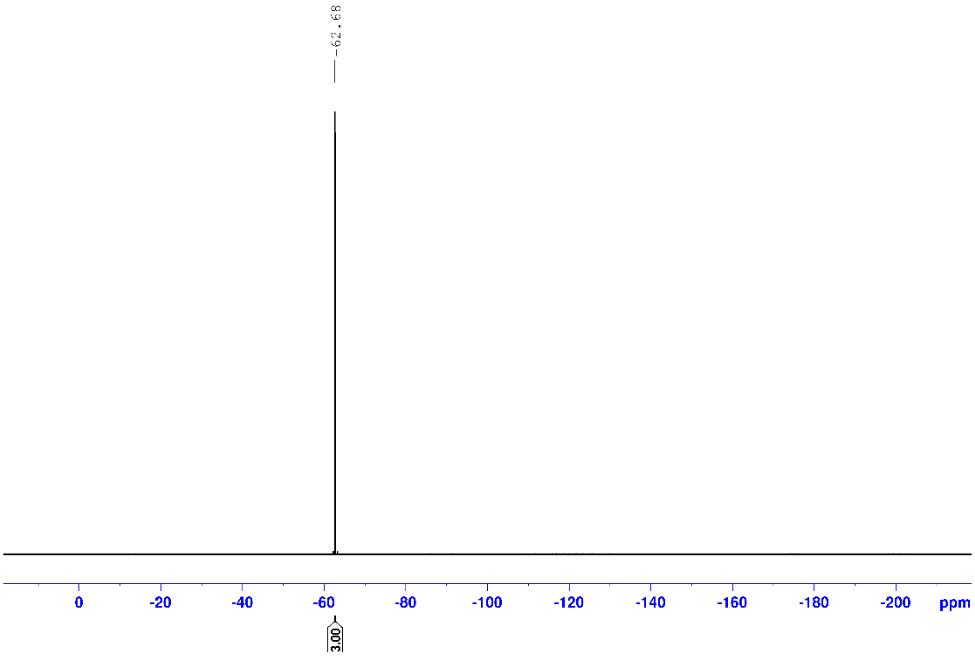

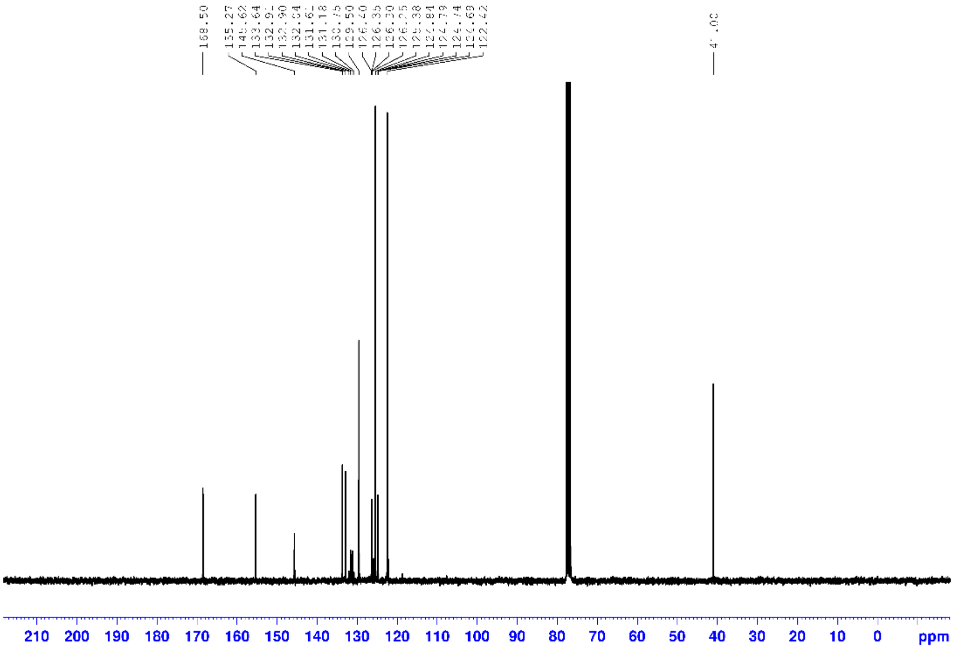
168.50
155.27
133.64
131.18
129.50
126.35
126.30
124.74
124.69
210
200
190
180
170
160
150
140
130
120
110
100
90
80
70
60
50
40
30
20
10
0
ppm

4-Nitrophenyl 2-(4-nitrophenyl)acetate (3t)

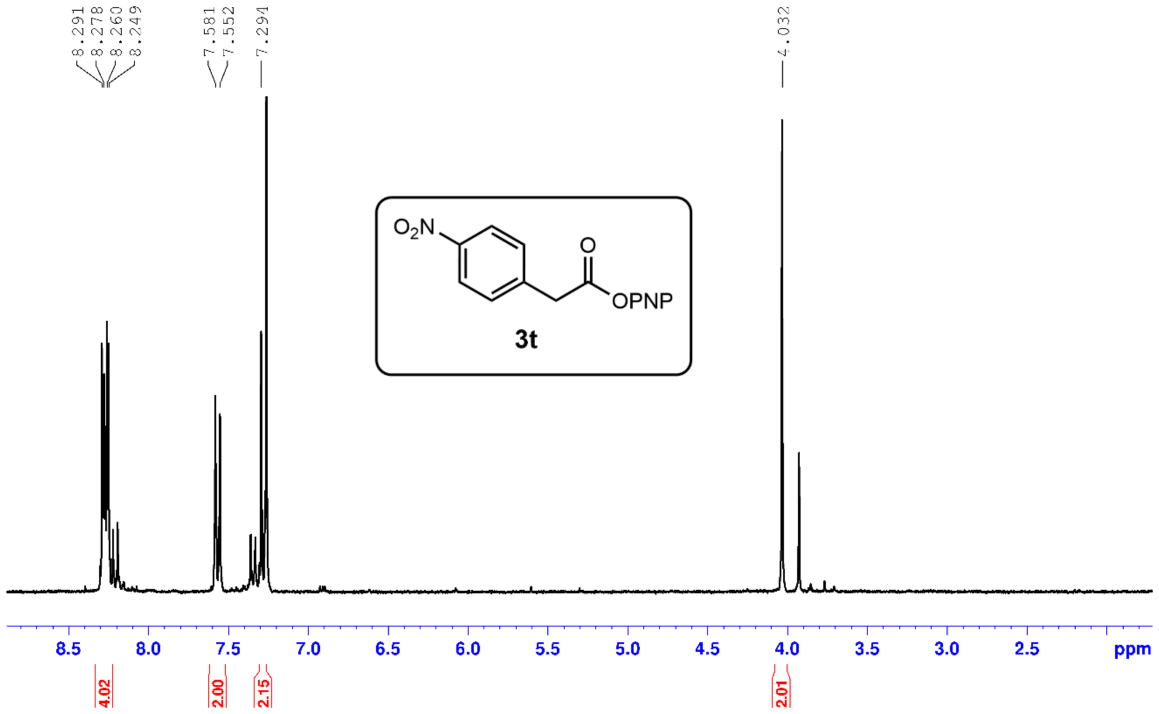

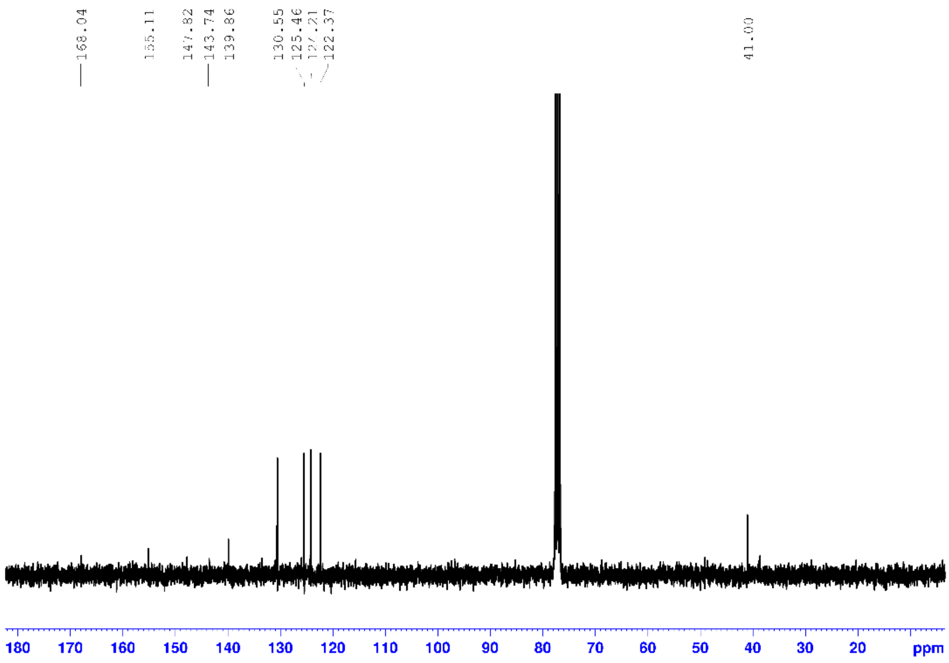

4-Nitrophenyl 2-(3-cyanophenyl)acetate (3u)

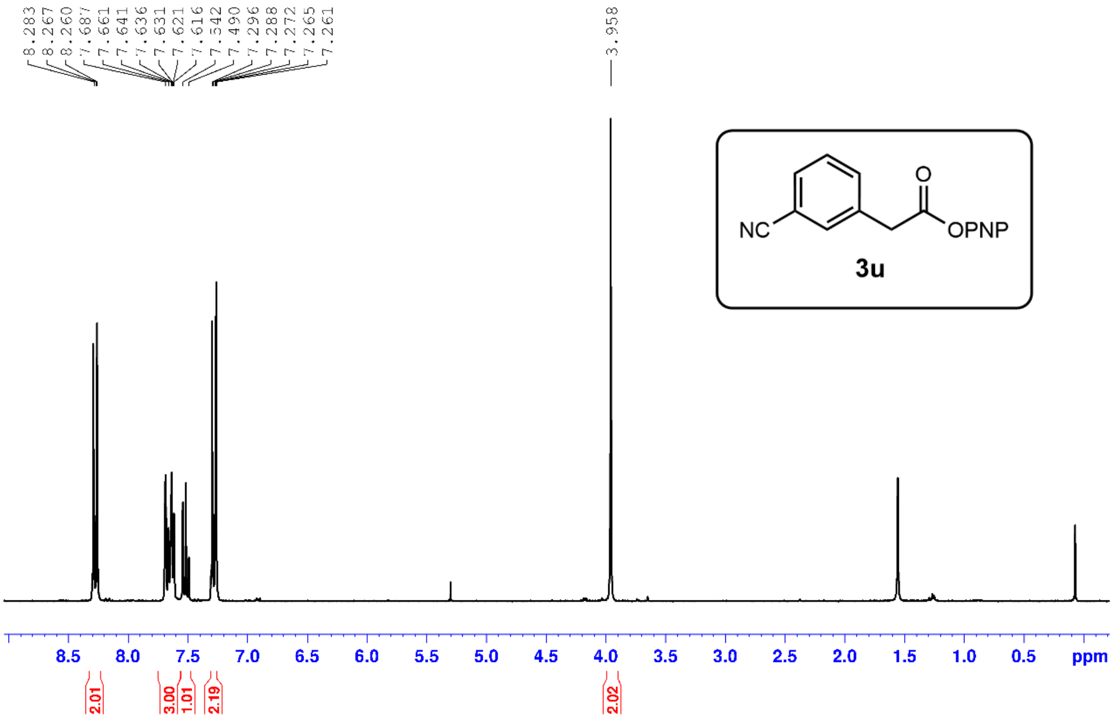

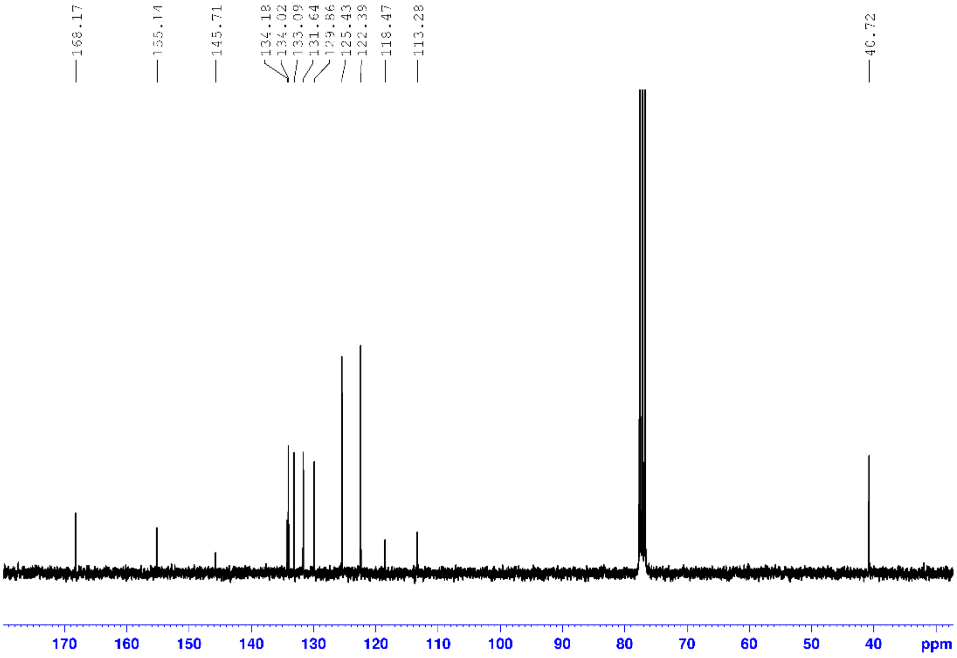

(2-(((*t*-Butyldimethylsilyl)oxy)methyl)-4-fluorophenyl)methanol (4ab$^{prot.\ diol}$)

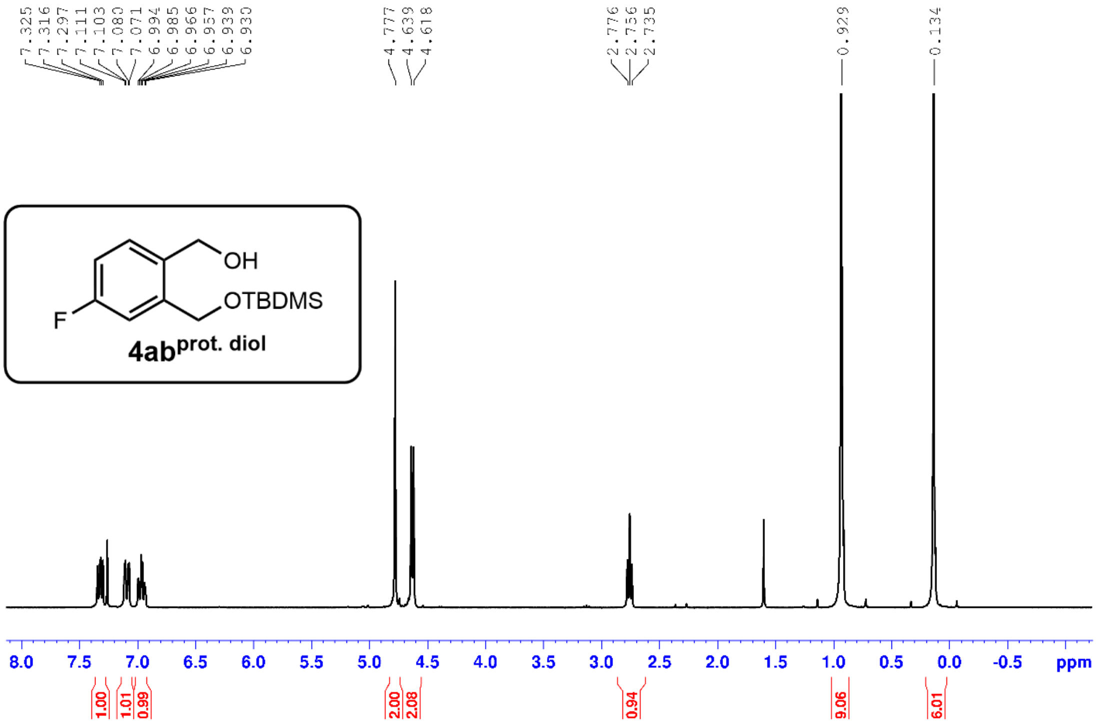

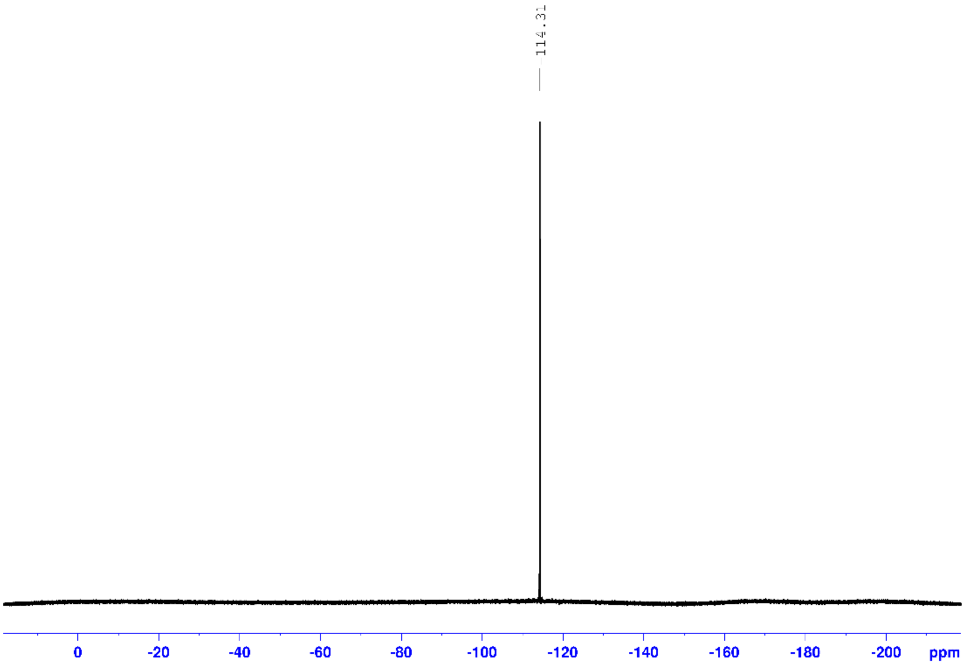

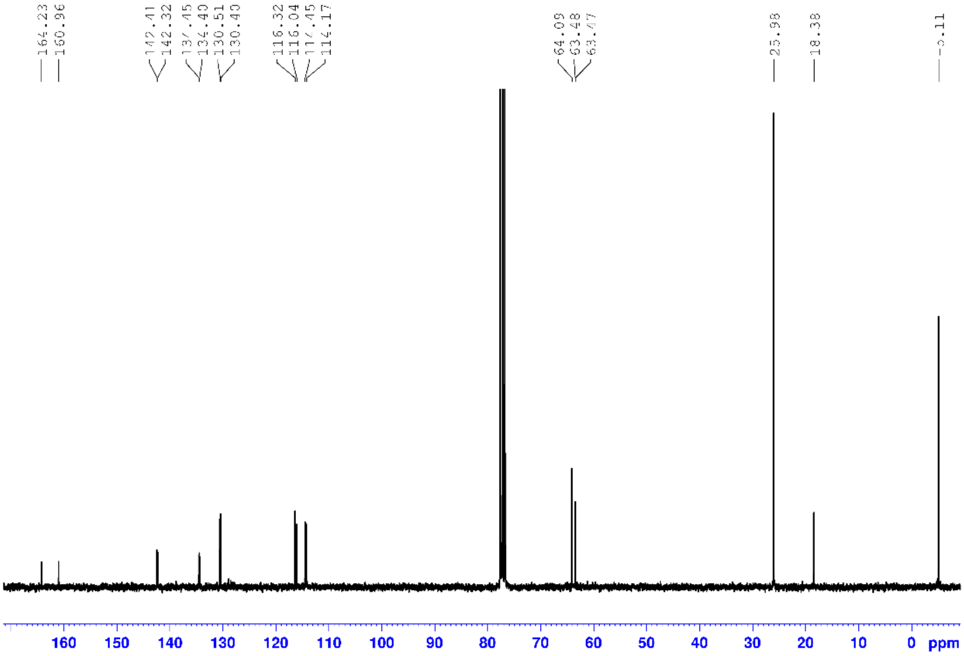
164.23
160.96
142.32
134.40
130.51
130.40
116.32
116.04
114.17
64.09
63.48
25.98
18.38
160
150
140
130
120
110
100
90
80
70
60
50
40
30
20
10
0
ppm

(2-(((*t*-Butyldimethylsilyl)oxy)methyl)-5-fluorophenyl)methanol (4aa$^{prot.\ diol}$)

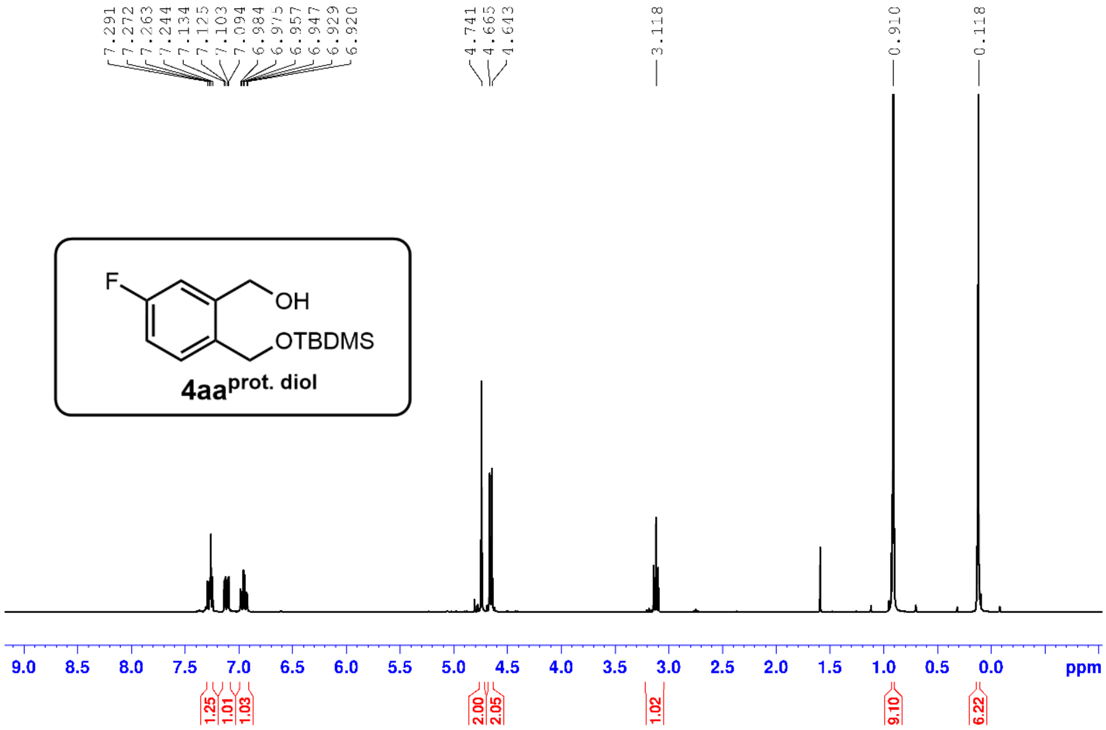

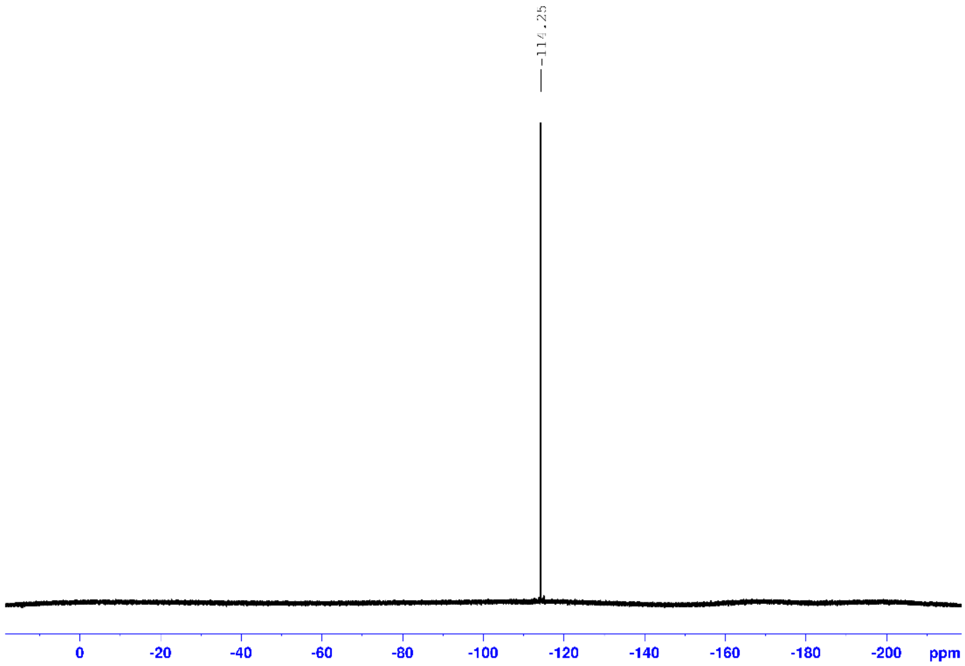

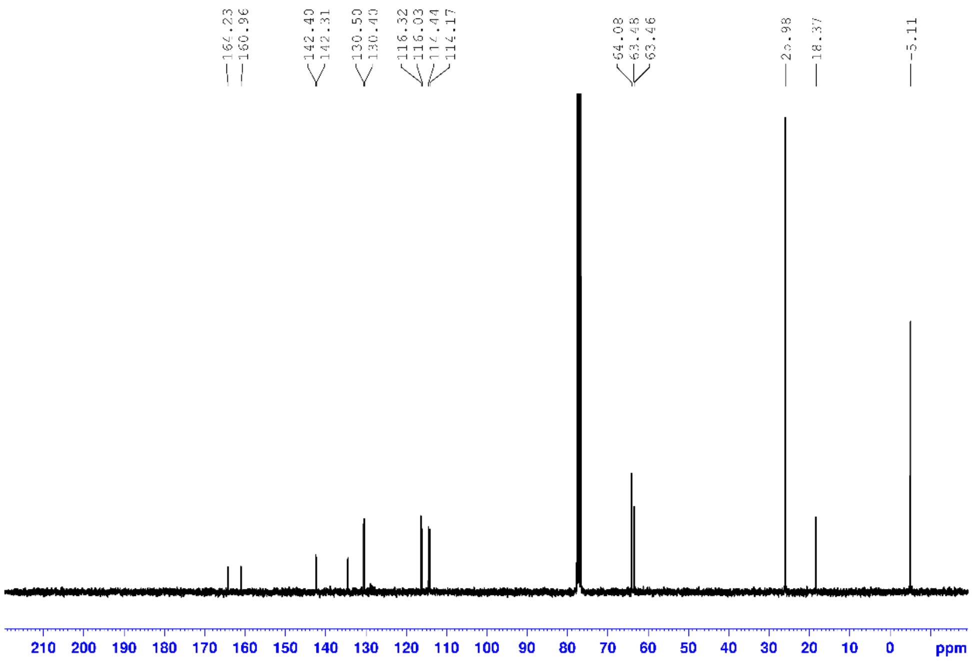
164.23
160.96
142.40
142.31
130.50
130.40
116.32
116.03
114.44
114.17
64.08
63.48
63.46
25.98
18.37
-5.11
210 200 190 180 170 160 150 140 130 120 110 100 90 80 70 60 50 40 30 20 10 0 ppm

(2-(((*t*-Butyldimethylsilyl)oxy)methyl)-4-chlorophenyl)methanol (4bb[prot. diol])

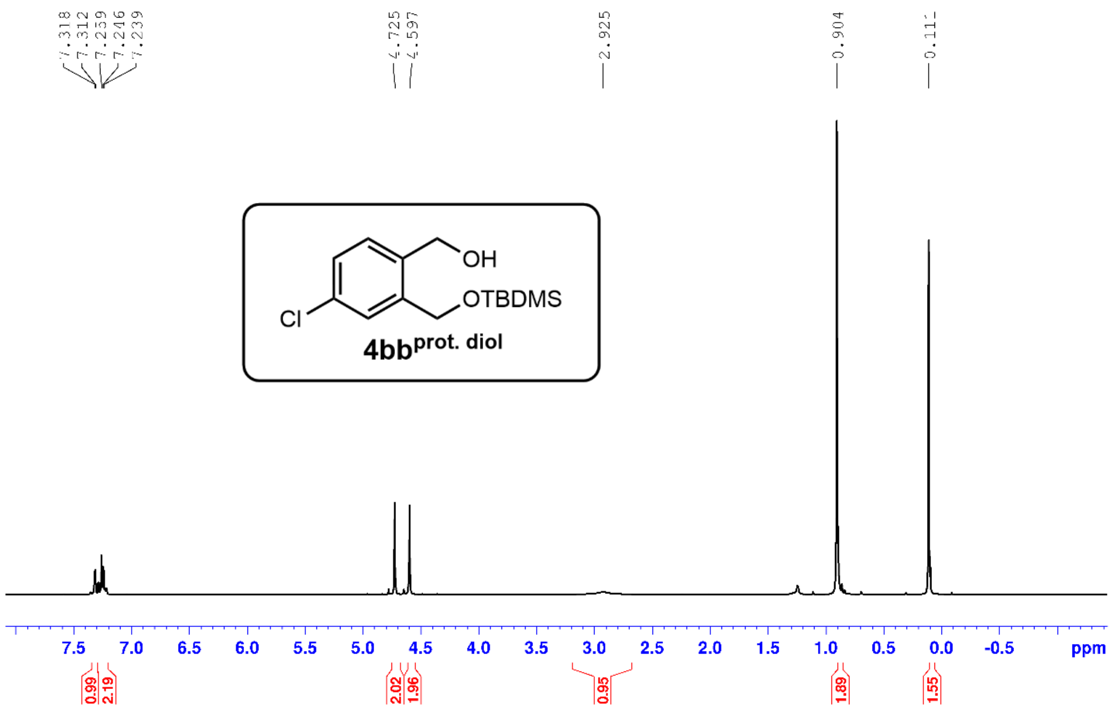

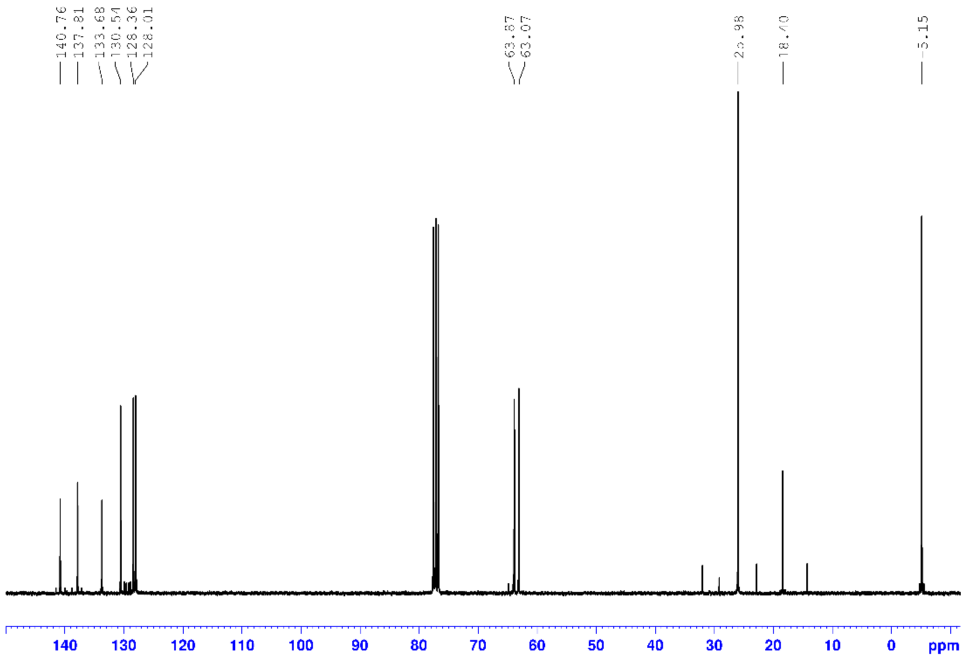

(2-(((*t*-Butyldimethylsilyl)oxy)methyl)-5-chlorophenyl)methanol (4ba$^{\text{prot. diol}}$)

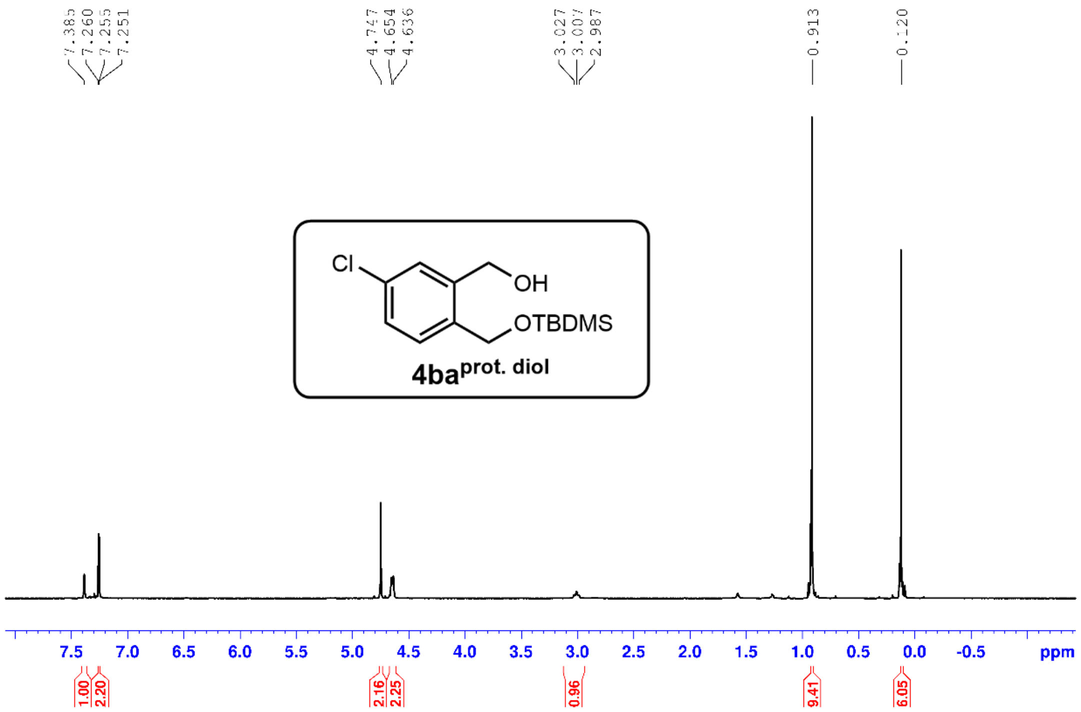

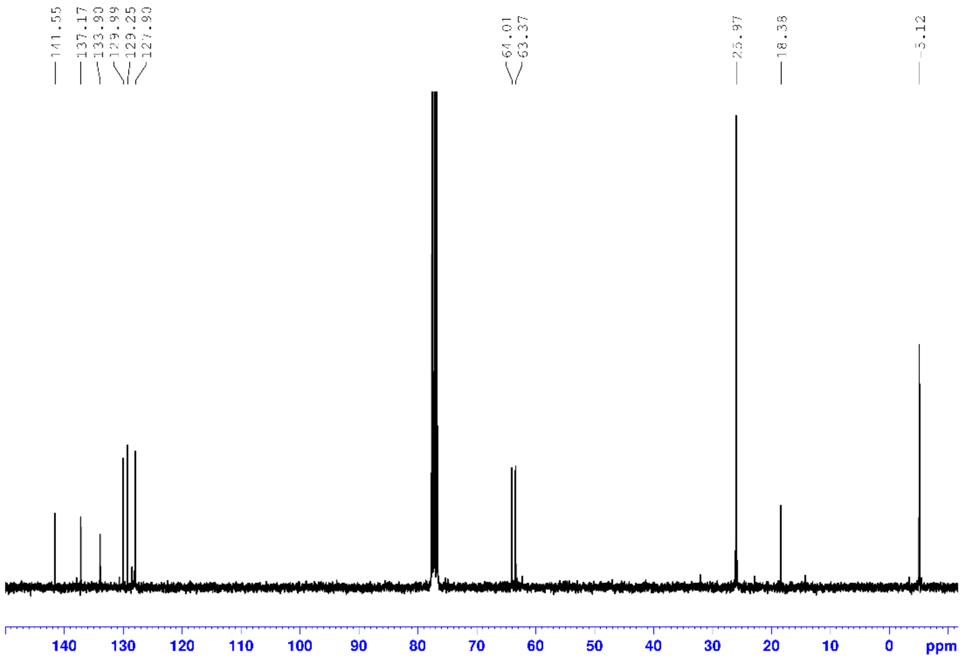

(3-(((*t*-Butyldimethylsilyl)oxy)methyl)naphthalen-2-yl)methanol (4da$^{prot.\ diol}$)

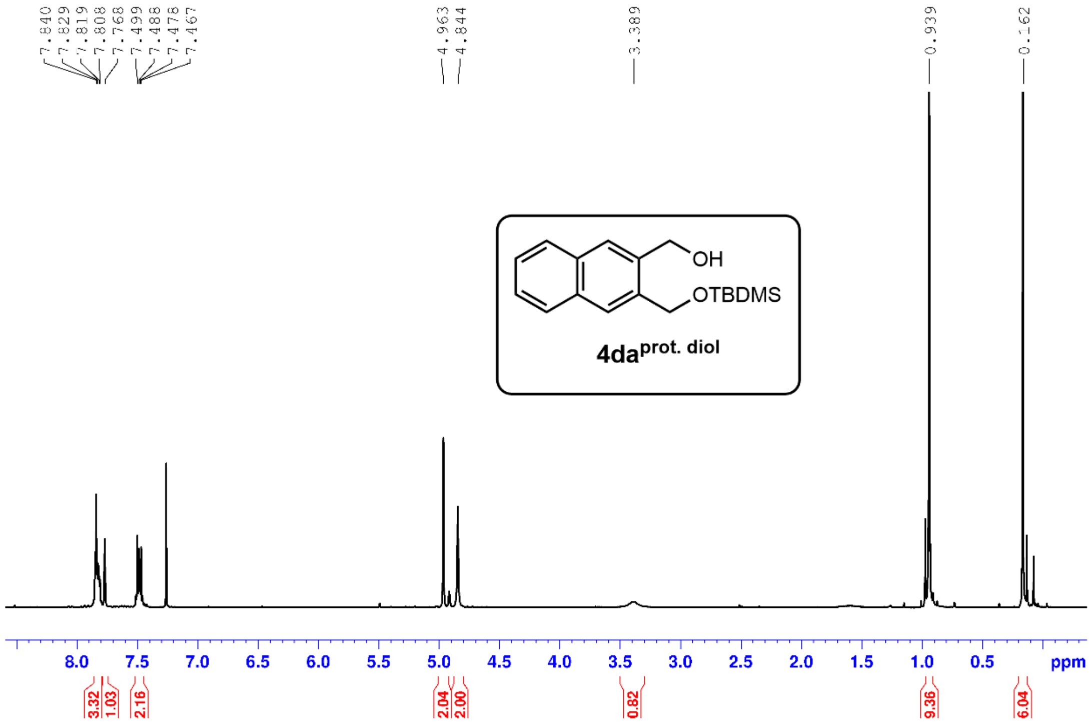

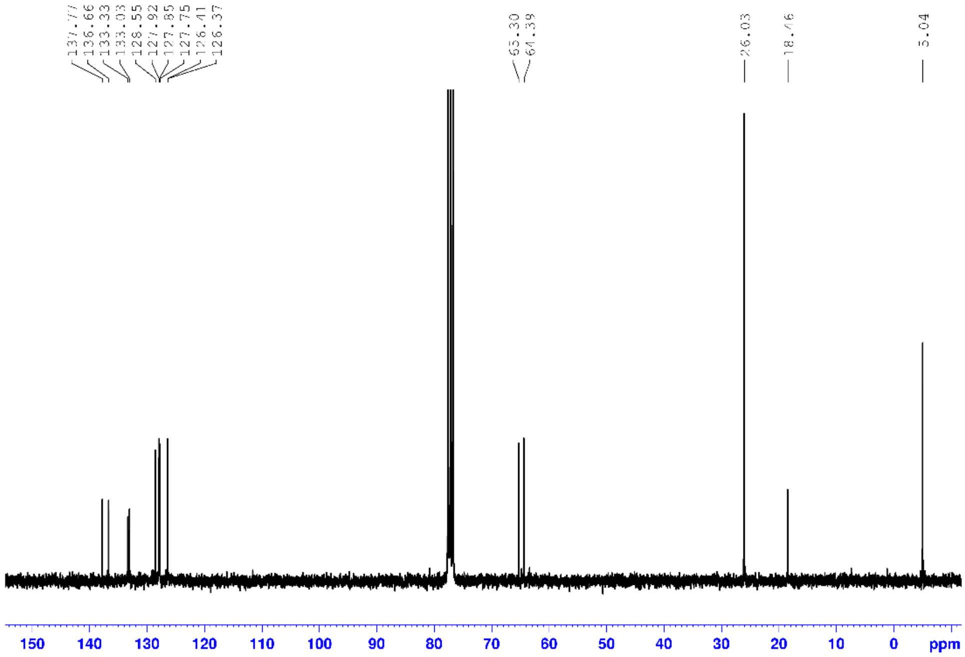

2-((*t*-Butyldimethylsilyl)oxy)methyl)-5-fluorobenzaldehyde (4aa^Aldehyde^)

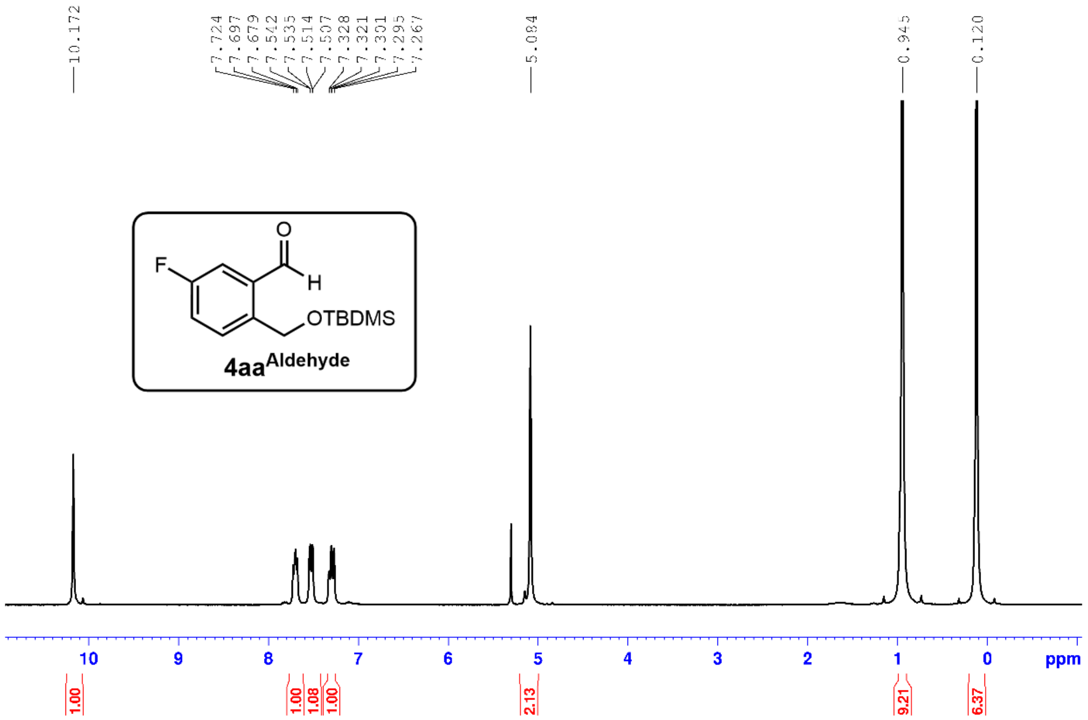

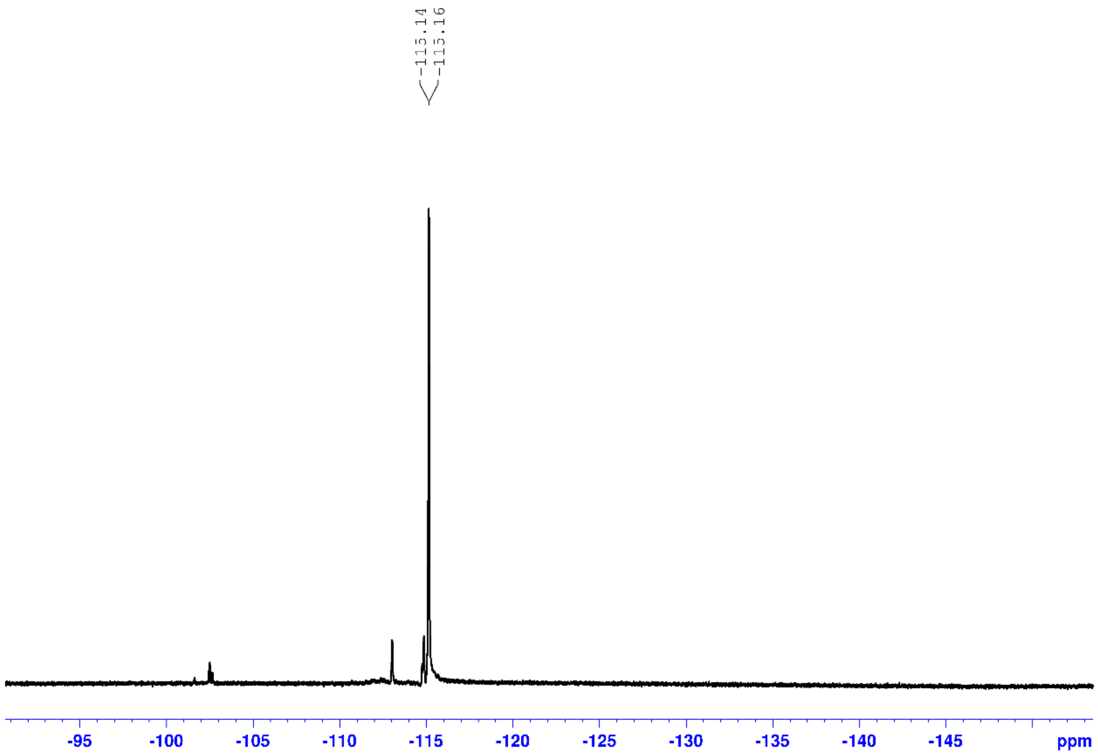

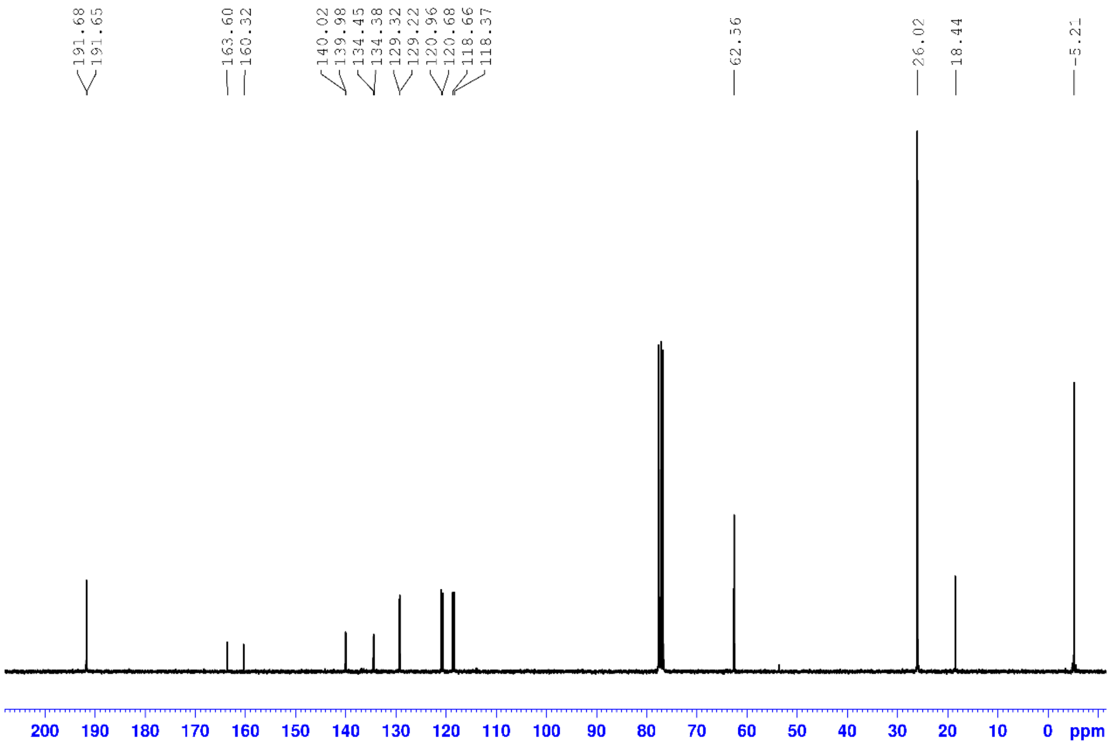

191.68
191.65
163.60
160.32
140.02
139.98
134.45
134.38
129.32
129.22
120.96
120.68
118.66
118.37
62.36
26.02
18.44
-5.21
200
190
180
170
160
150
140
130
120
110
100
90
80
70
60
50
40
30
20
10
0
ppm

2-((*t*-Butyldimethylsilyl)oxy)methyl)-4-fluorobenzaldehyde (4ab[Aldehyde])

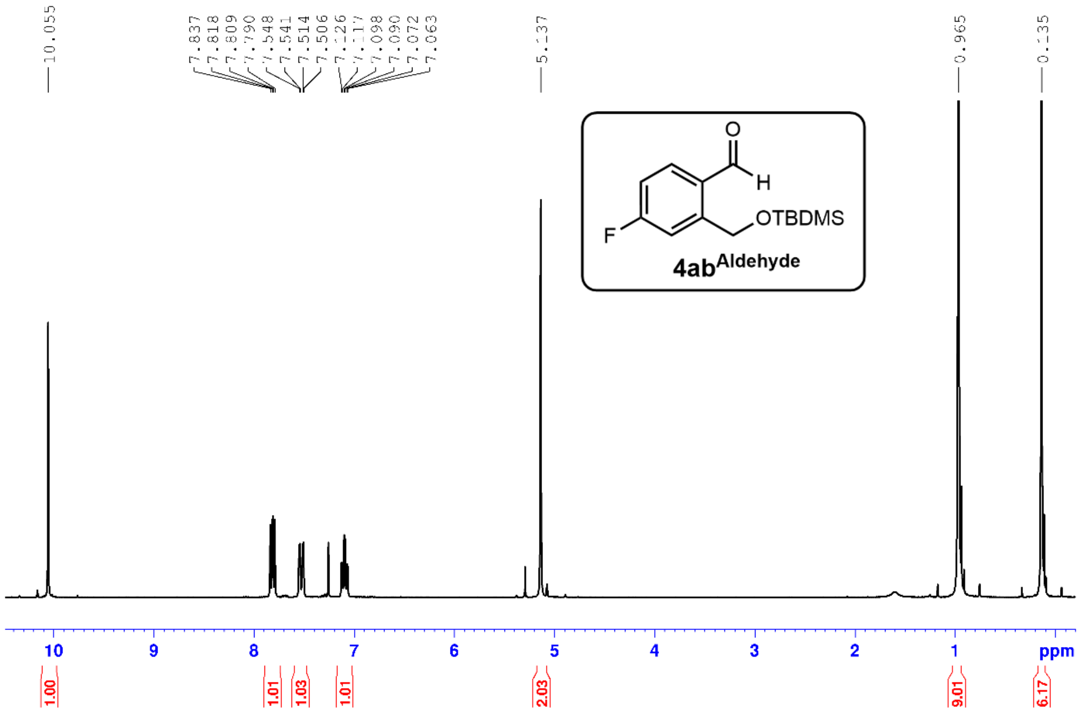

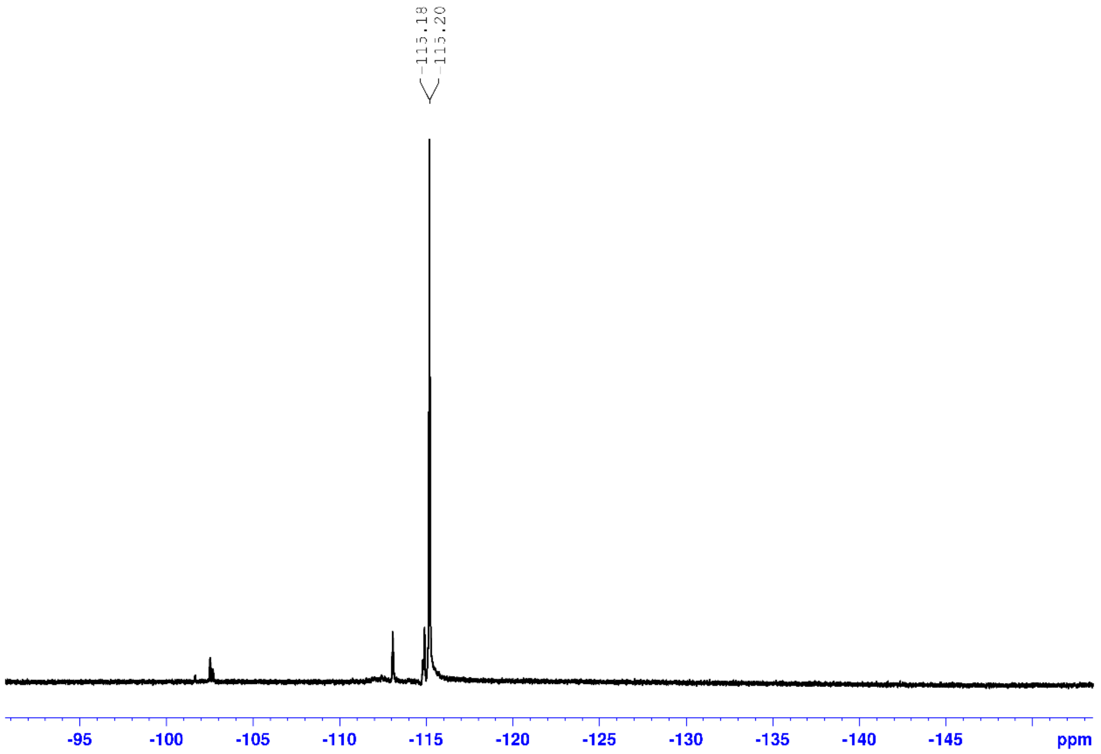

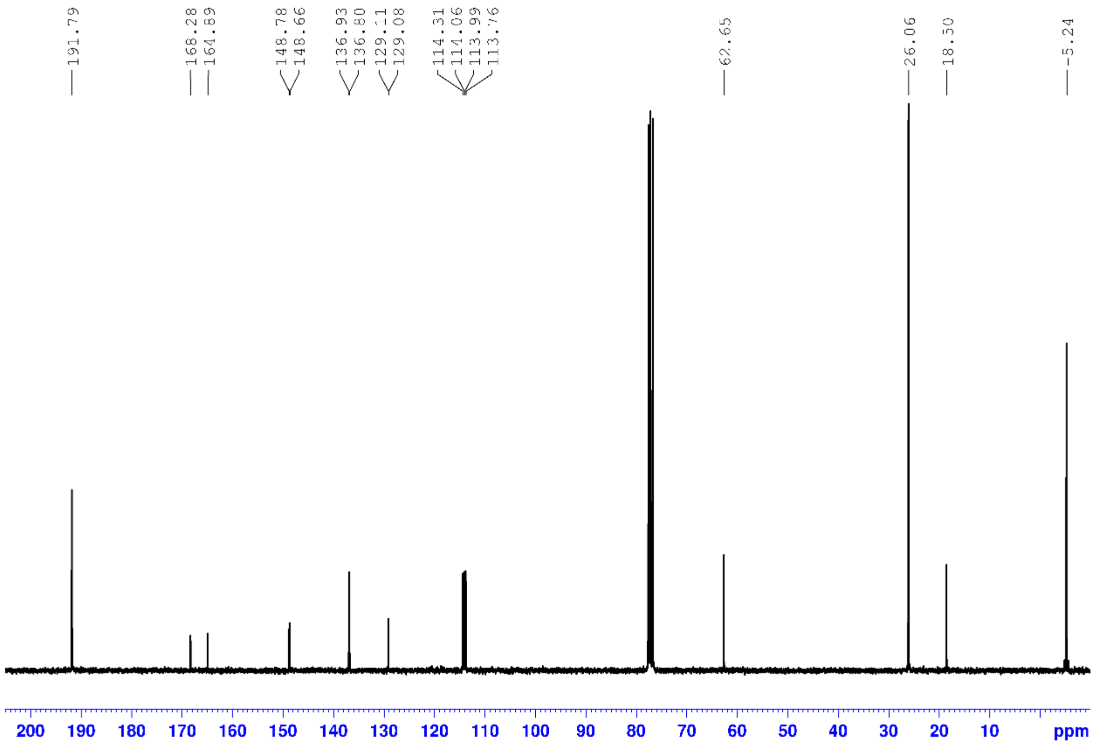
191.79
168.28
164.89
148.78
148.66
136.93
136.80
129.21
129.08
114.31
114.06
113.99
113.76
62.65
26.06
18.30
-5.24
200
190
180
170
160
150
140
130
120
110
100
90
80
70
60
50
40
30
20
10
ppm

2-((*t*-Butyldimethylsilyl)oxy)methyl)-5-chlorobenzaldehyde (4ba^Aldehyde^)

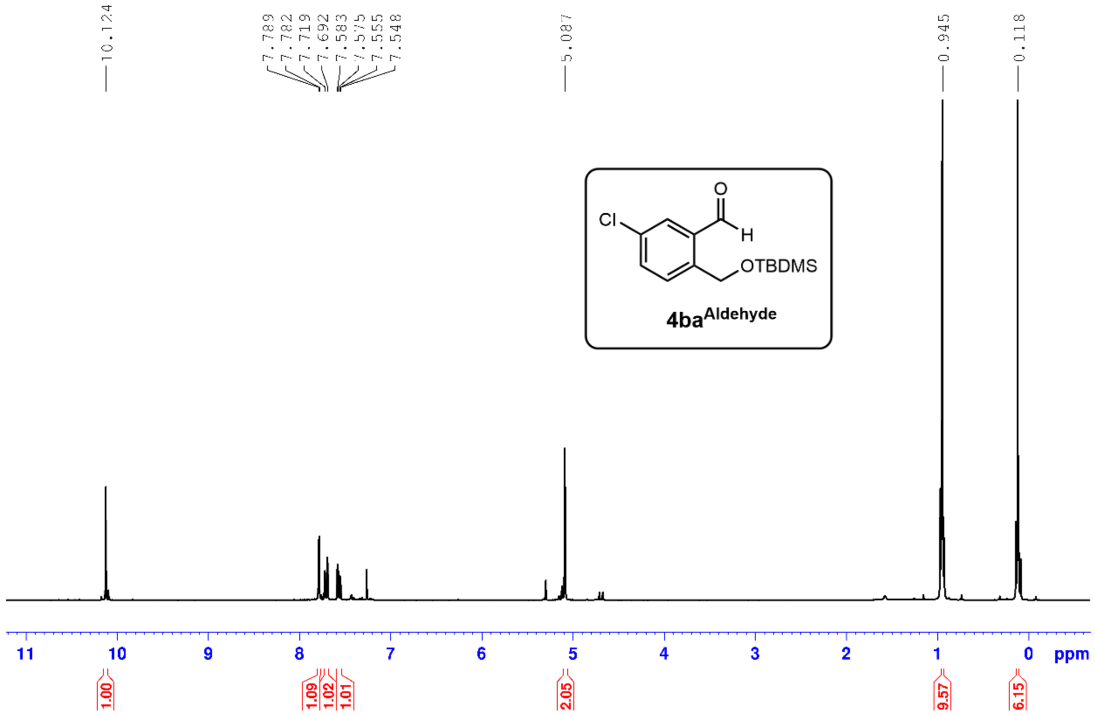

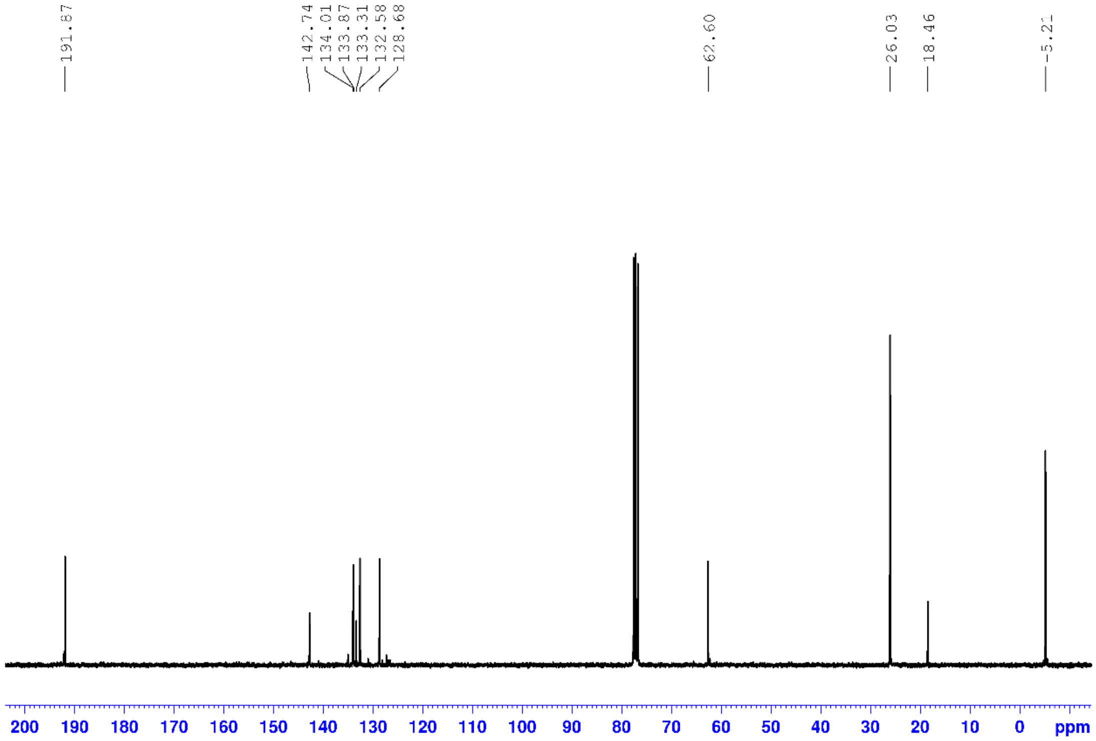

2-((*t*-Butyldimethylsilyl)oxy)methyl)-4-chlorobenzaldehyde (4bb[Aldehyde])

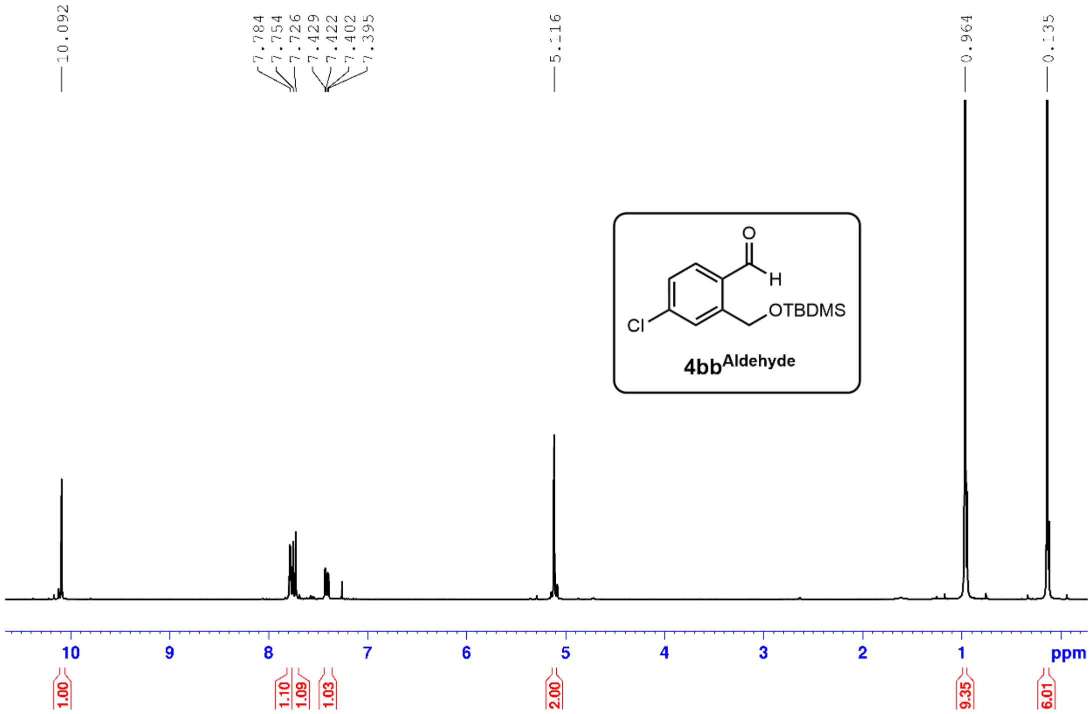

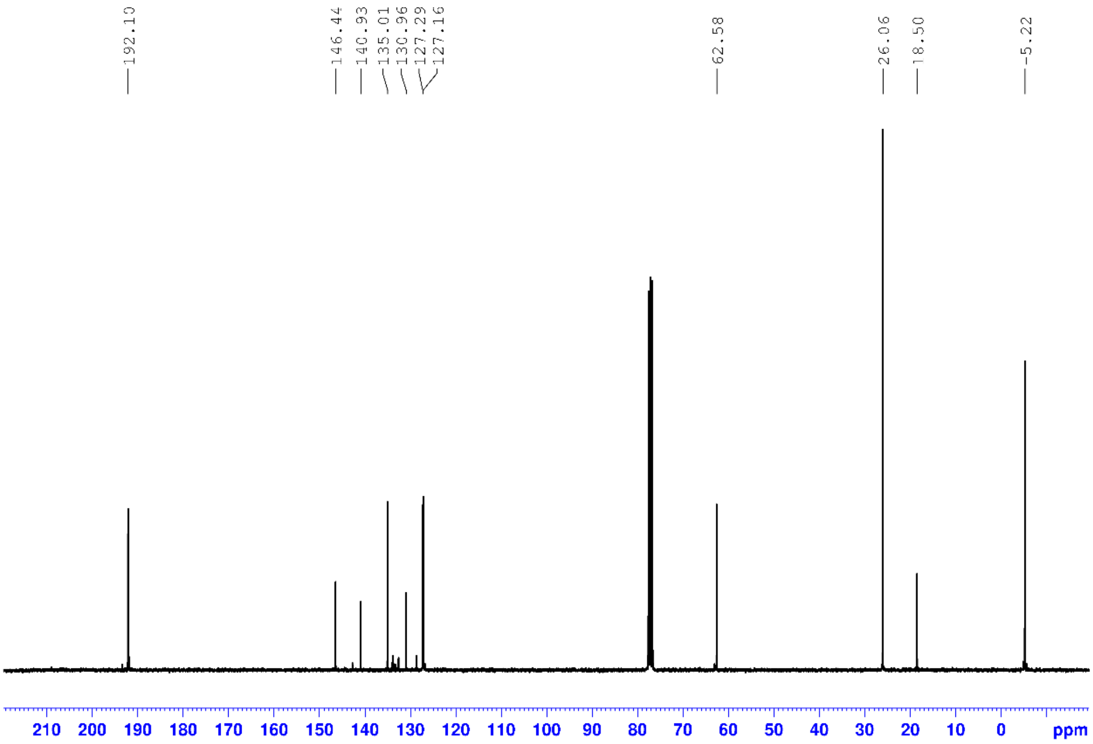

3-(((tert-butyldimethylsilyl)oxy)methyl)-2-naphthaldehyde (4da[Aldehyde])

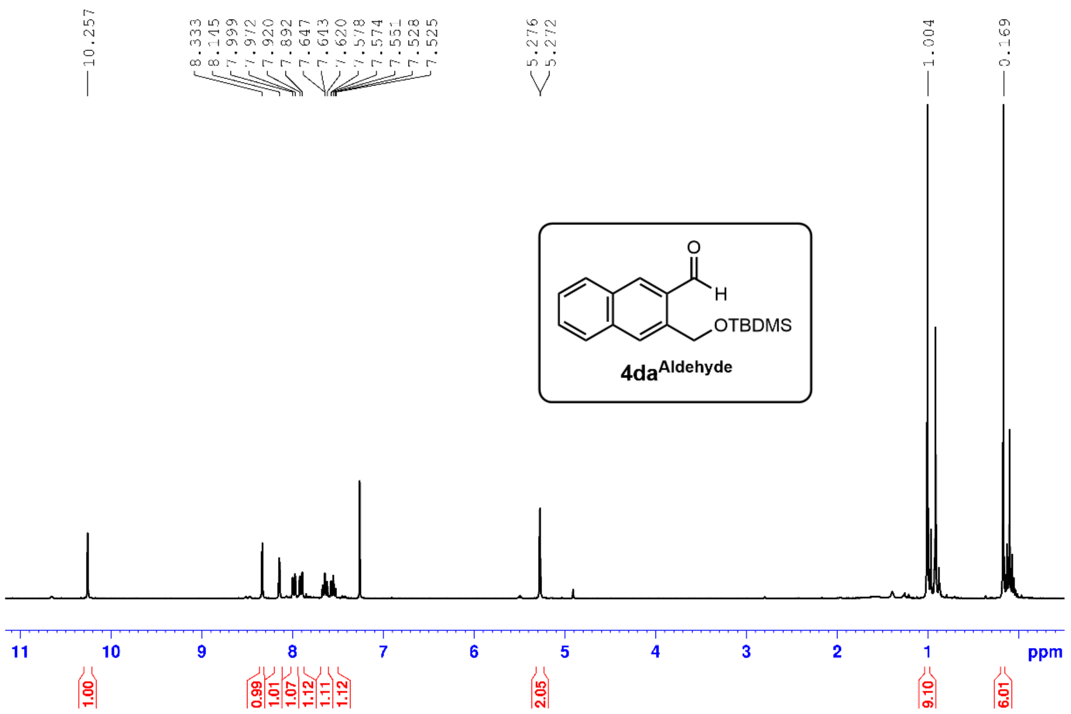

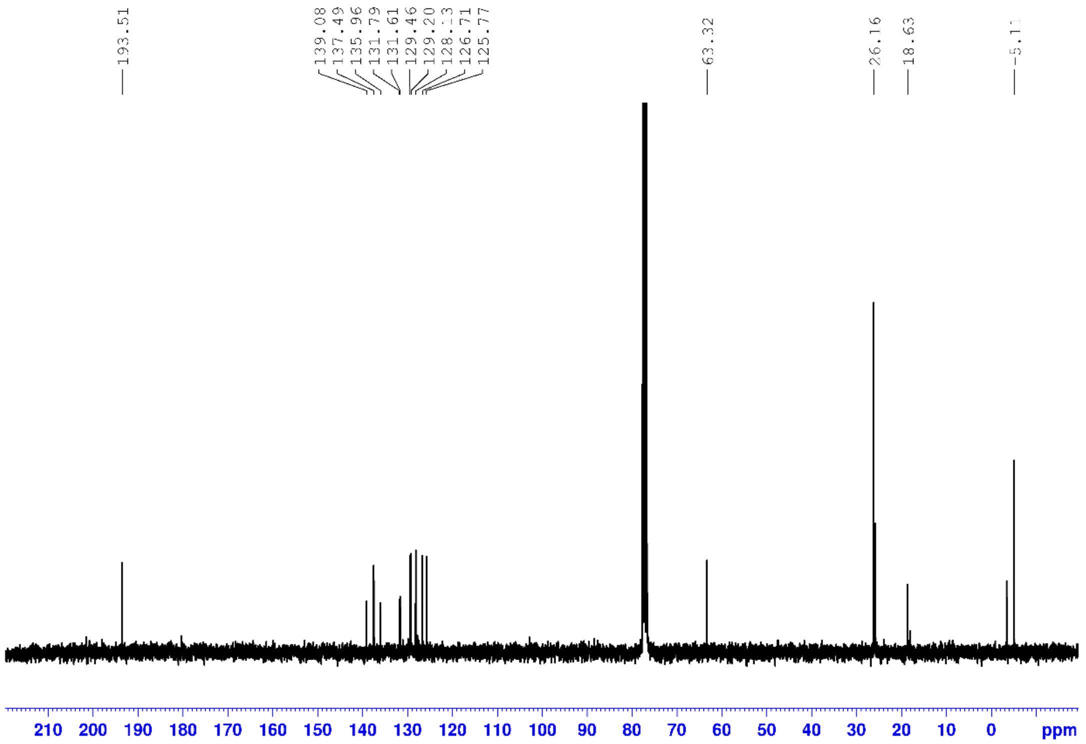

Unsubstituted Quinone Methide (4a)

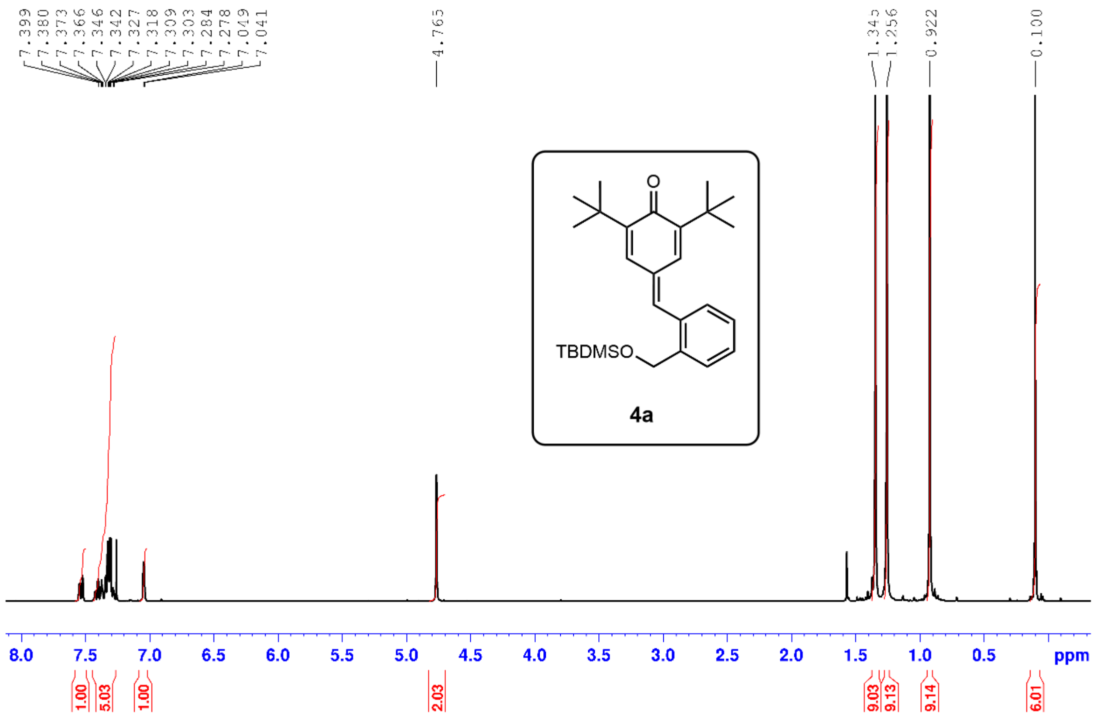

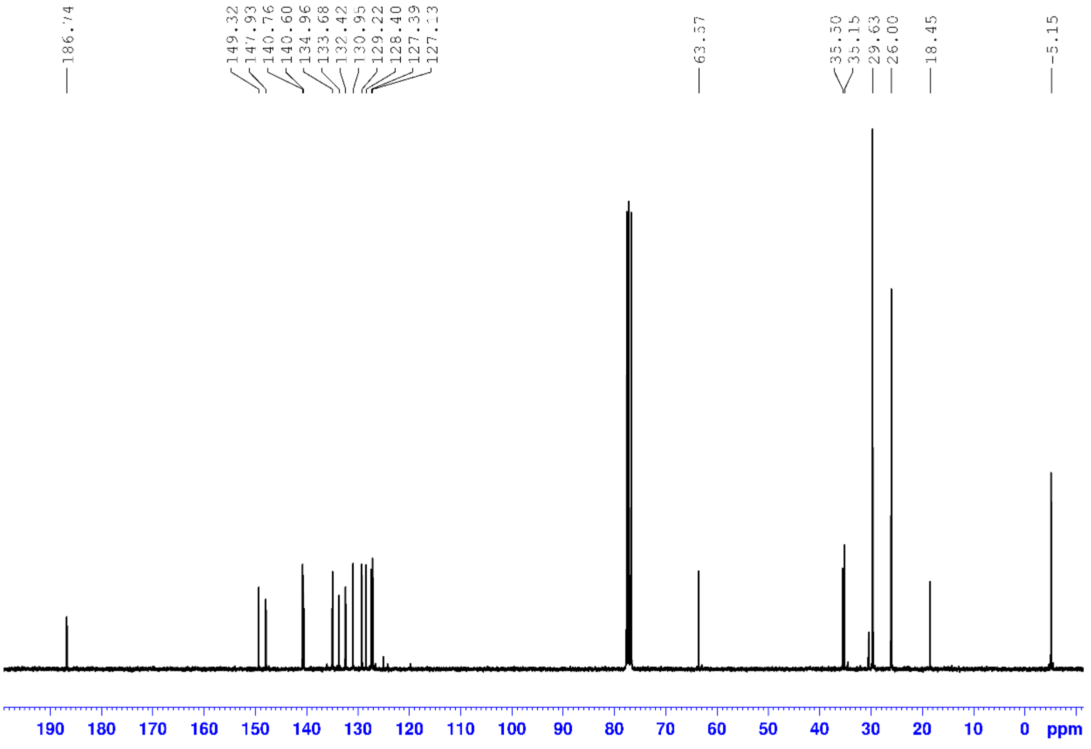

5-Fluoro Quinone Methide (4aa)

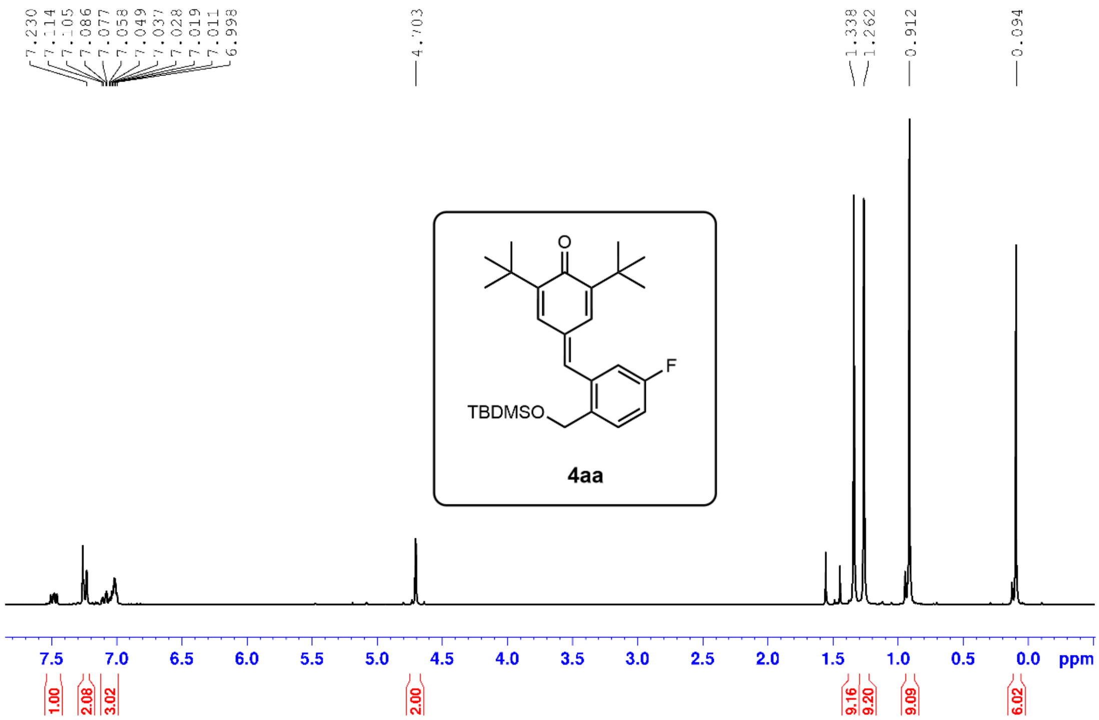

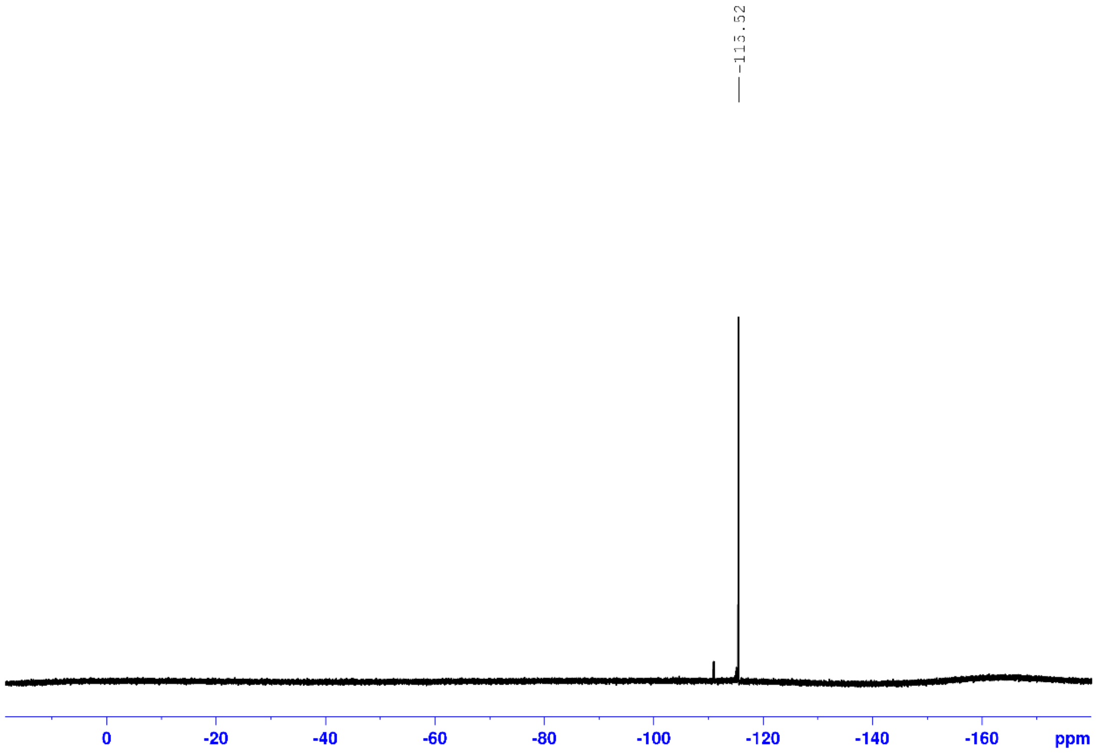

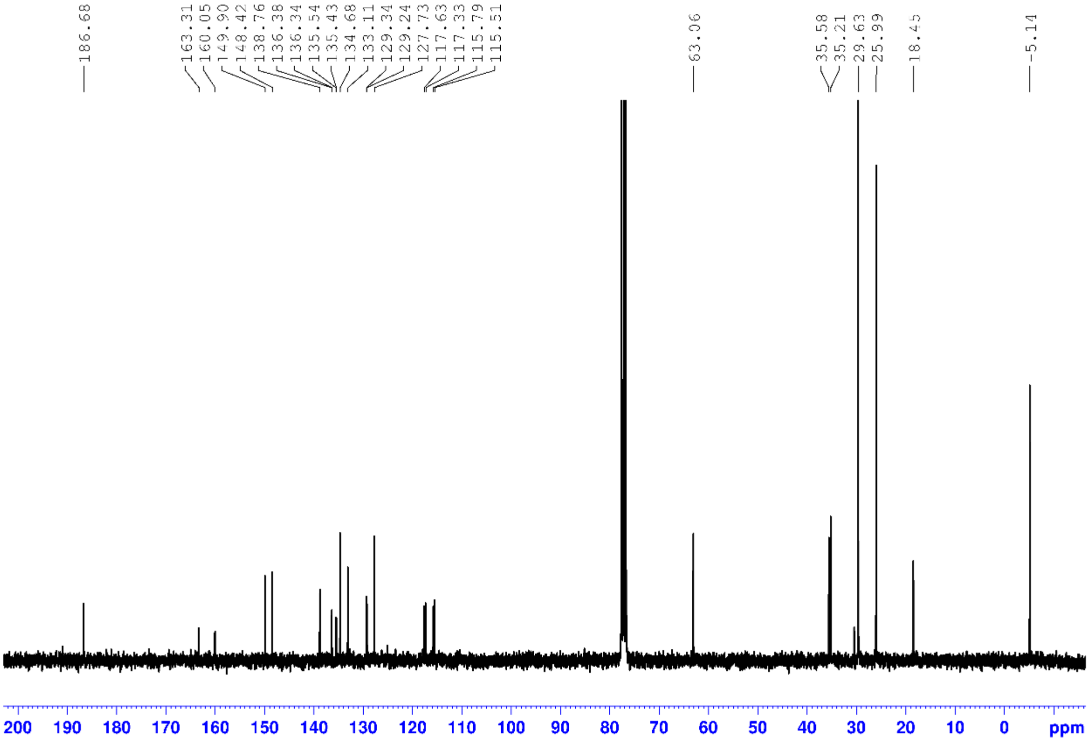
186.68
163.31
160.05
149.90
148.42
138.76
136.38
136.34
135.54
135.43
134.68
133.11
129.34
129.24
127.73
117.63
117.33
115.79
115.51
63.06
35.58
35.21
29.63
25.99
18.45
-5.14
200 190 180 170 160 150 140 130 120 110 100 90 80 70 60 50 40 30 20 10 0 ppm

4-Fluoro Quinone Methide (4ab)

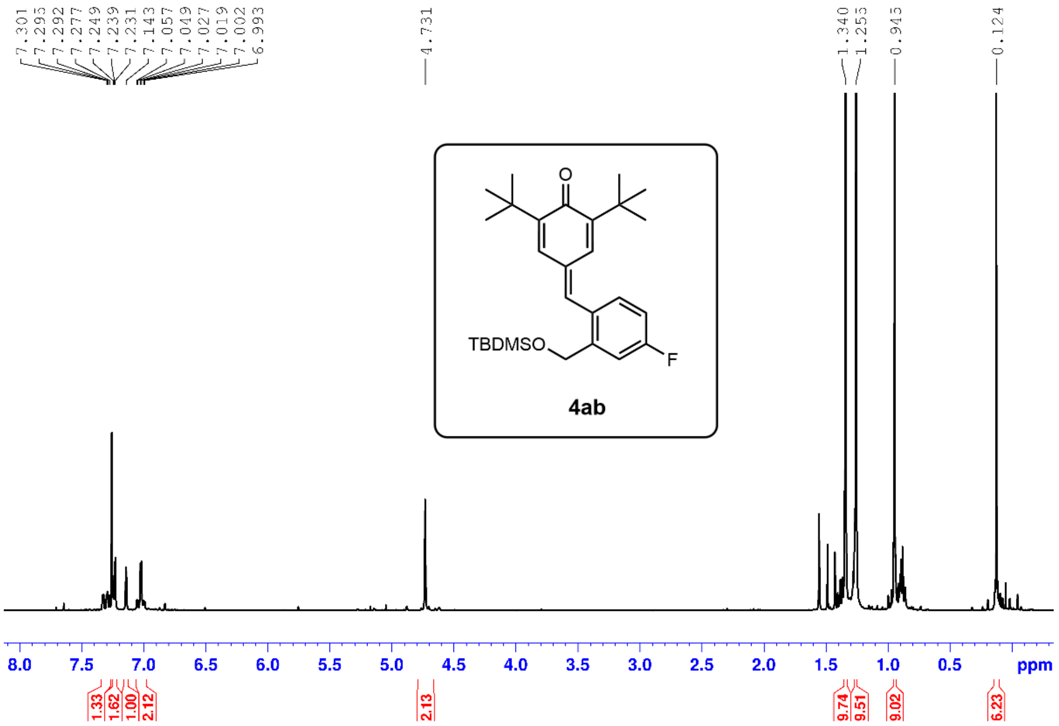

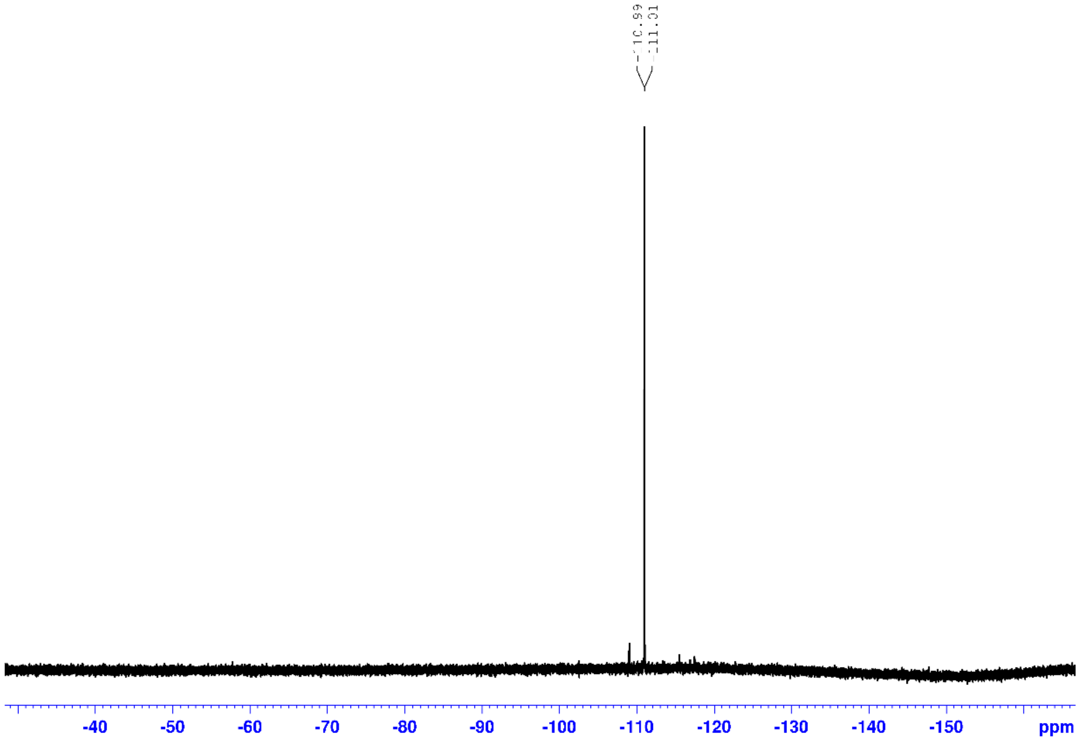

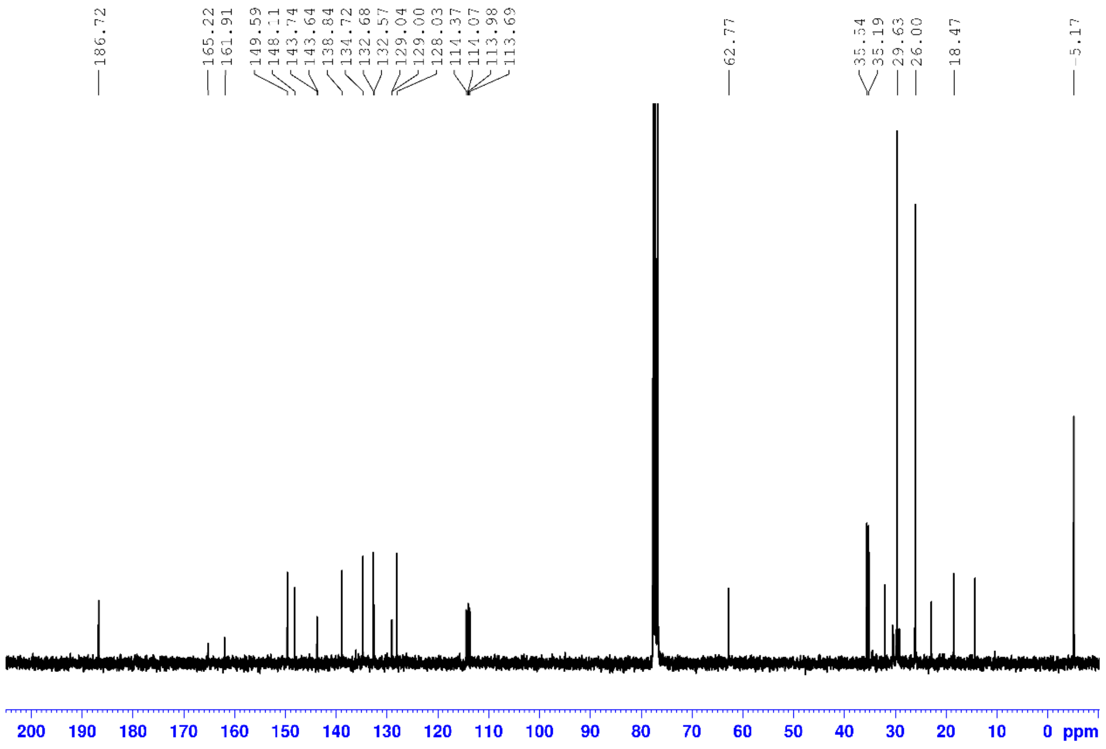
186.72
165.22
161.91
149.59
148.11
143.74
143.64
138.84
134.72
132.68
132.57
129.04
129.00
128.03
114.37
114.07
113.98
113.69
62.77
35.54
35.19
29.63
26.00
18.47
-5.17
200 190 180 170 160 150 140 130 120 110 100 90 80 70 60 50 40 30 20 10 0 ppm

5-Chloro Quinone Methide (4ba)

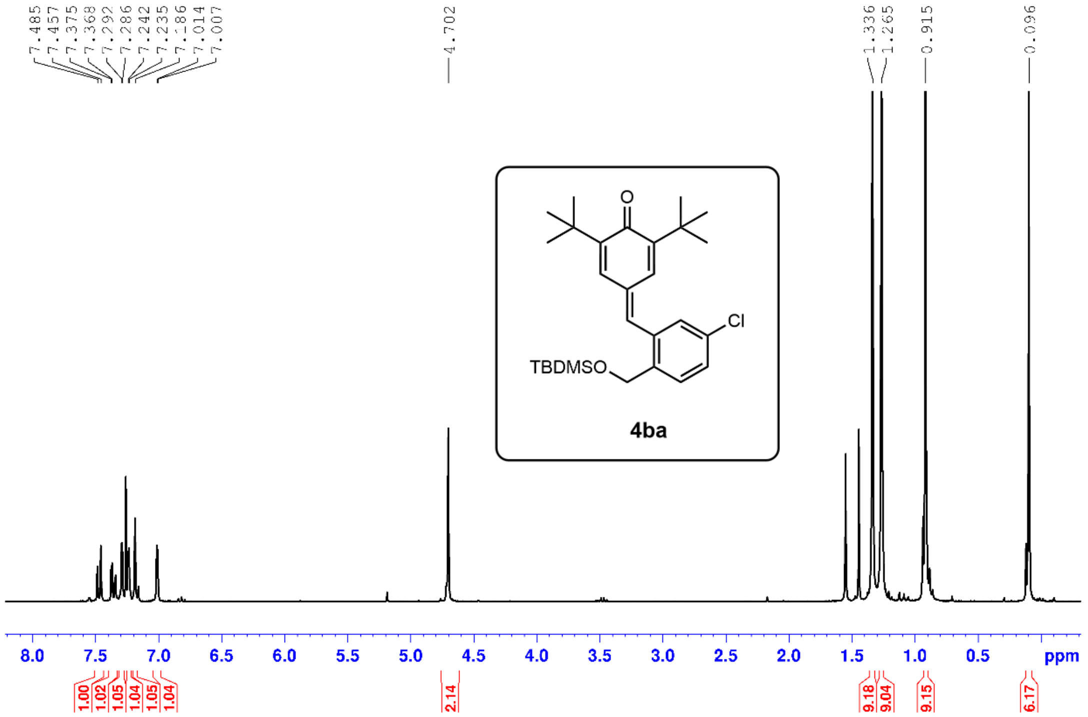

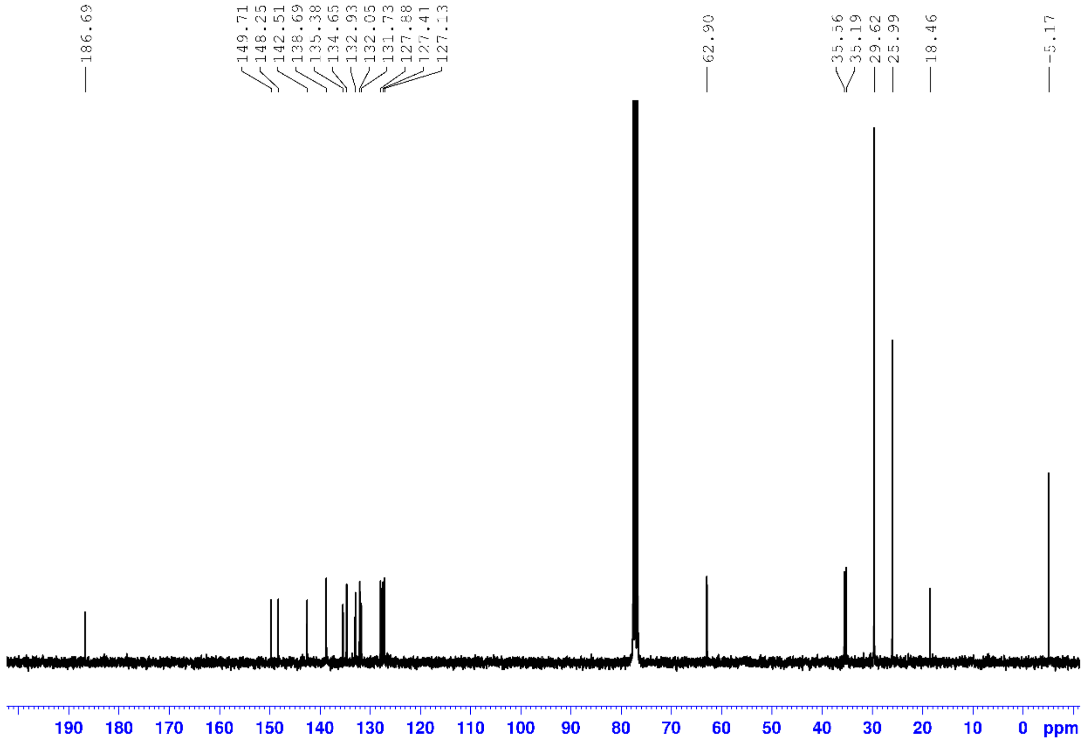

4-Chloro Quinone Methide (4bb)

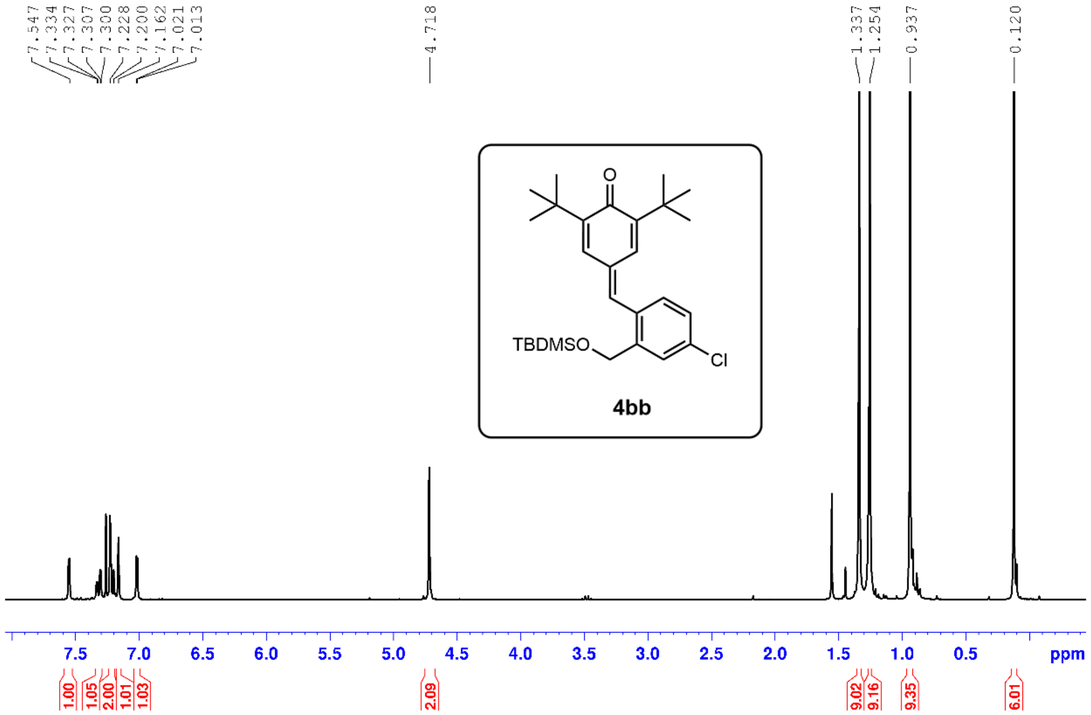

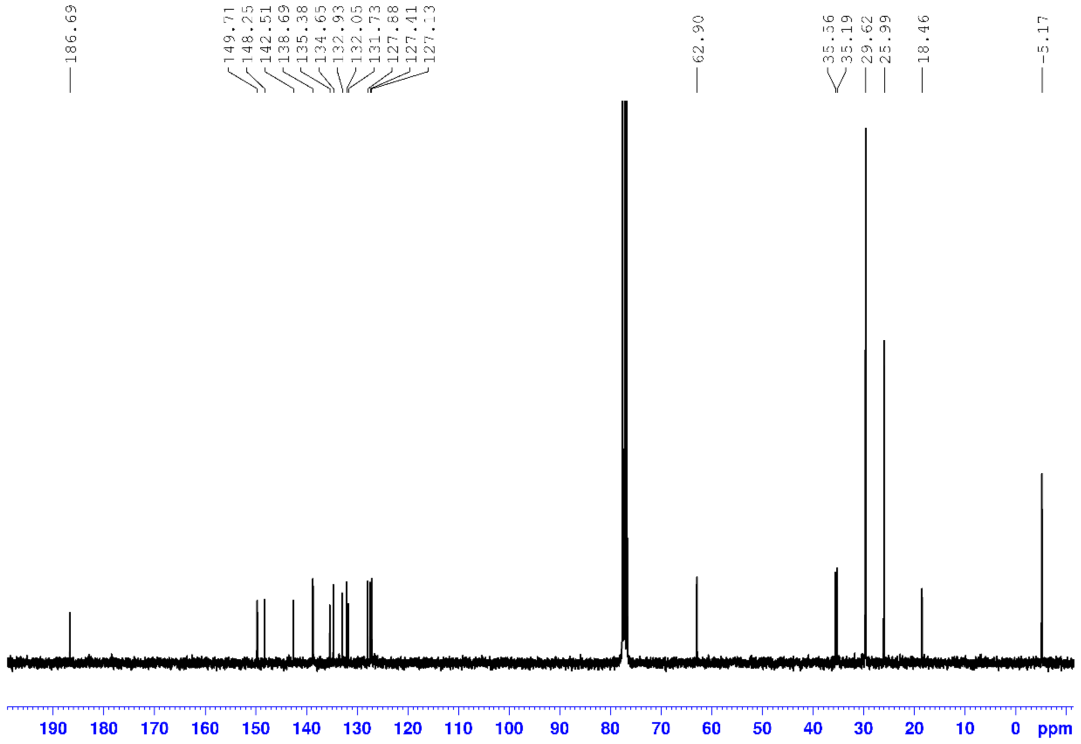

Naphthyl Quinone Methide (4da)

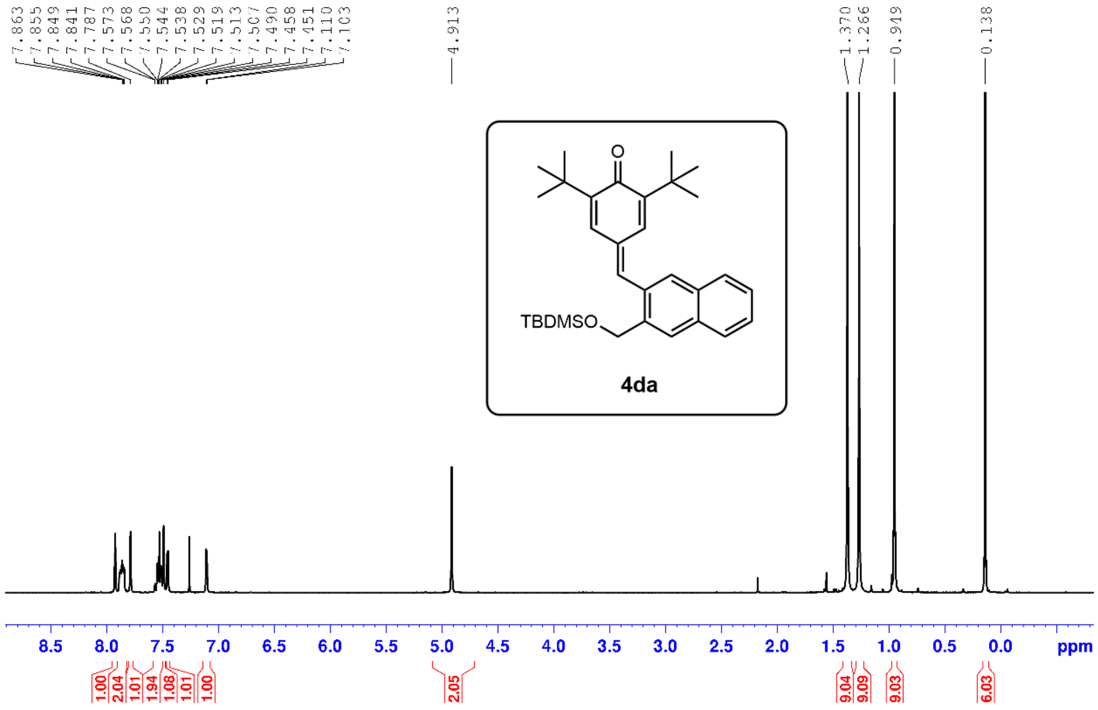

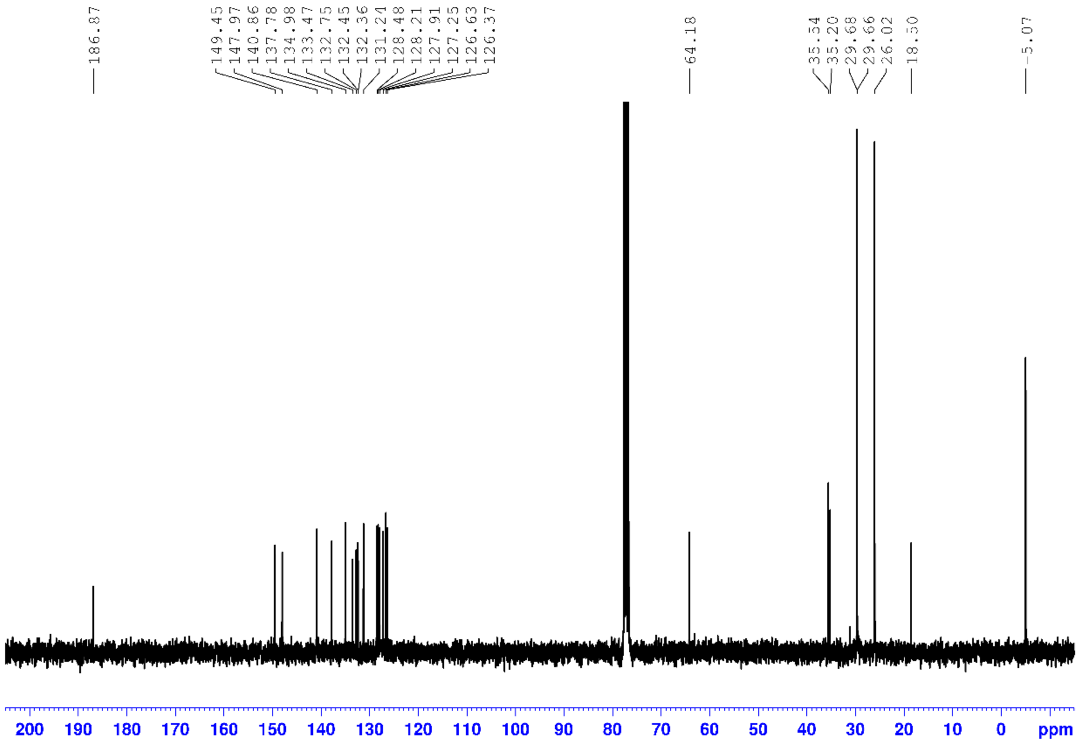

Unsymmetrically substituted Quinone Methide (4da) *(E/Z Mixture)*

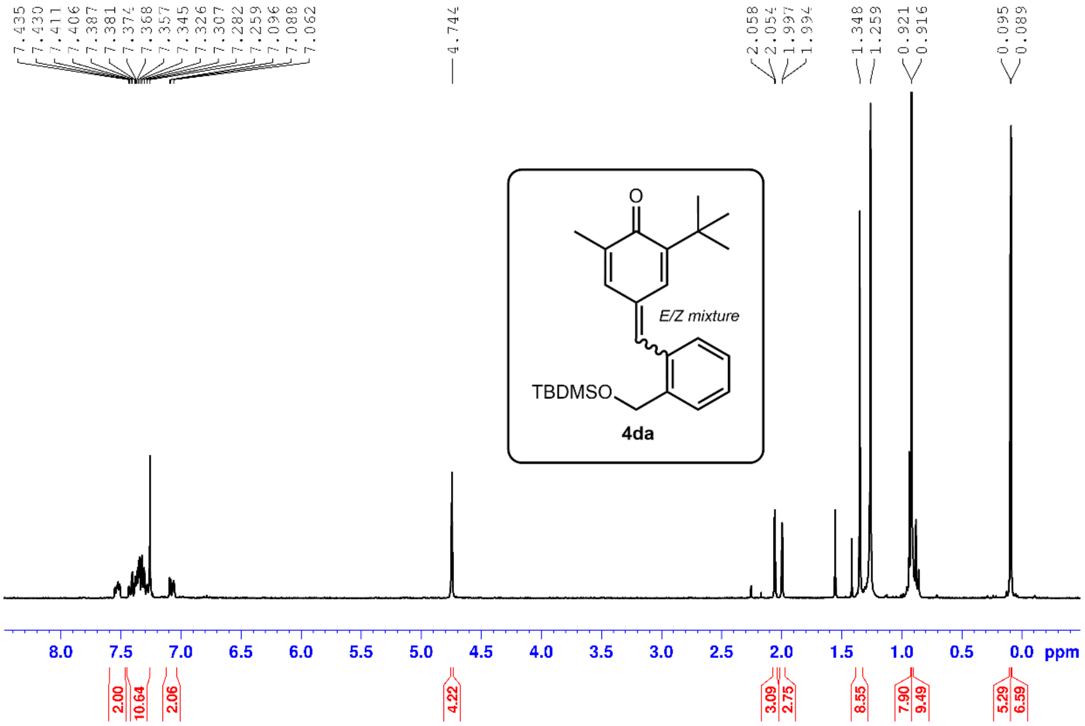

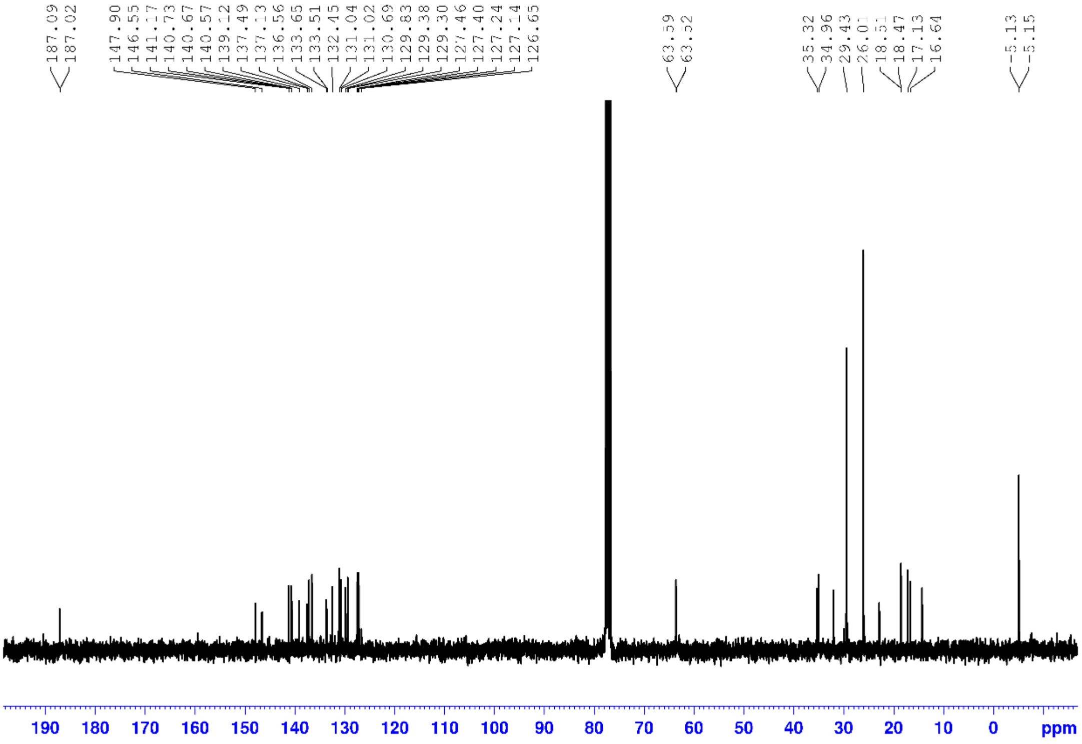

8.2 NMR Spectra for the Alkylation and Cyclization Products

Unsubstituted benzo[c]oxepinone derivative 6a

*Alkylation Product **5a***

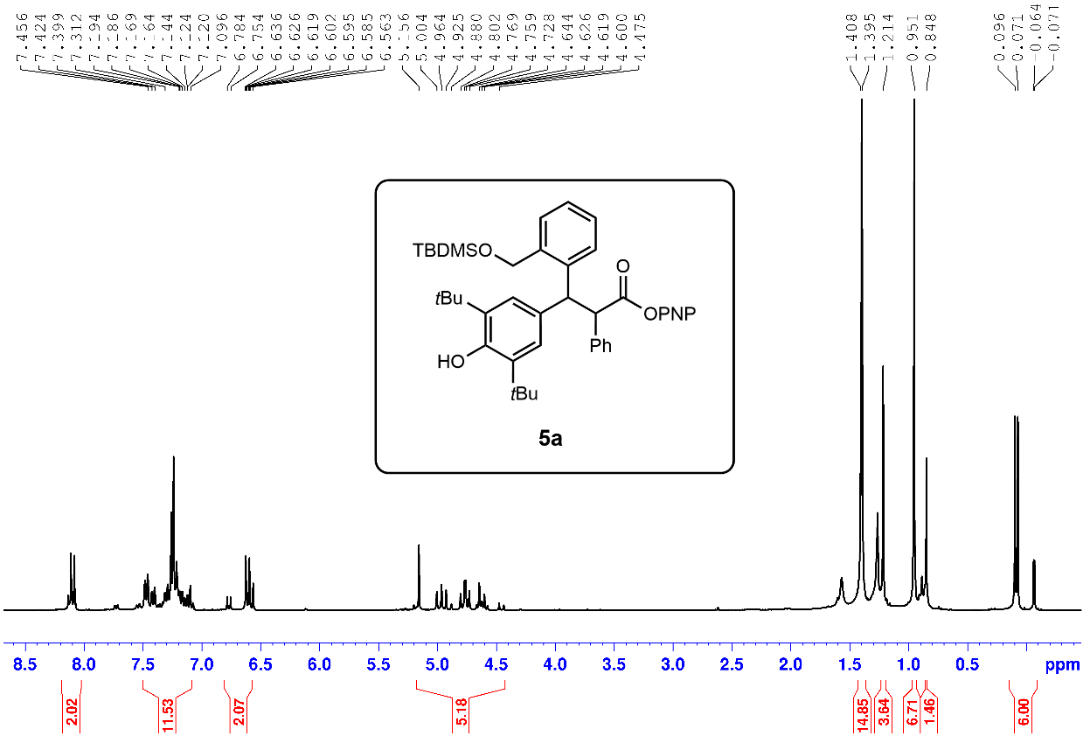

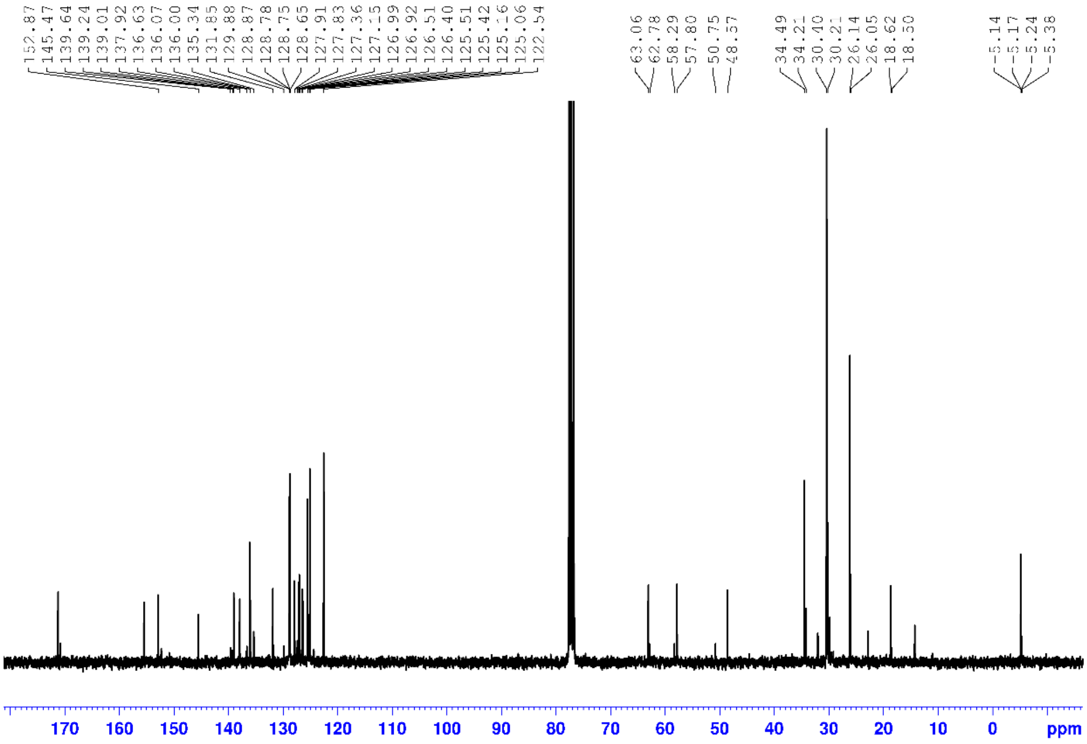

Cyclic cis product ***6a***$^{\mathbf{cis}}$

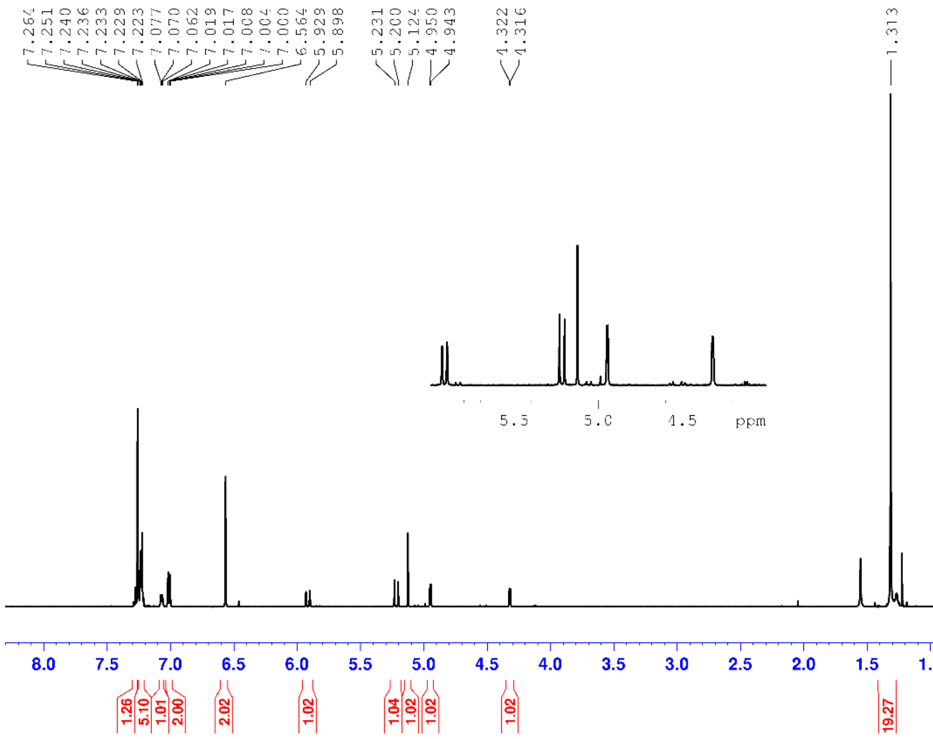

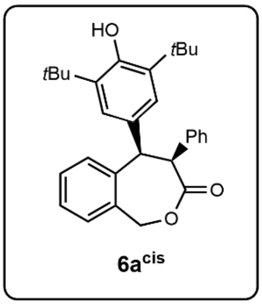

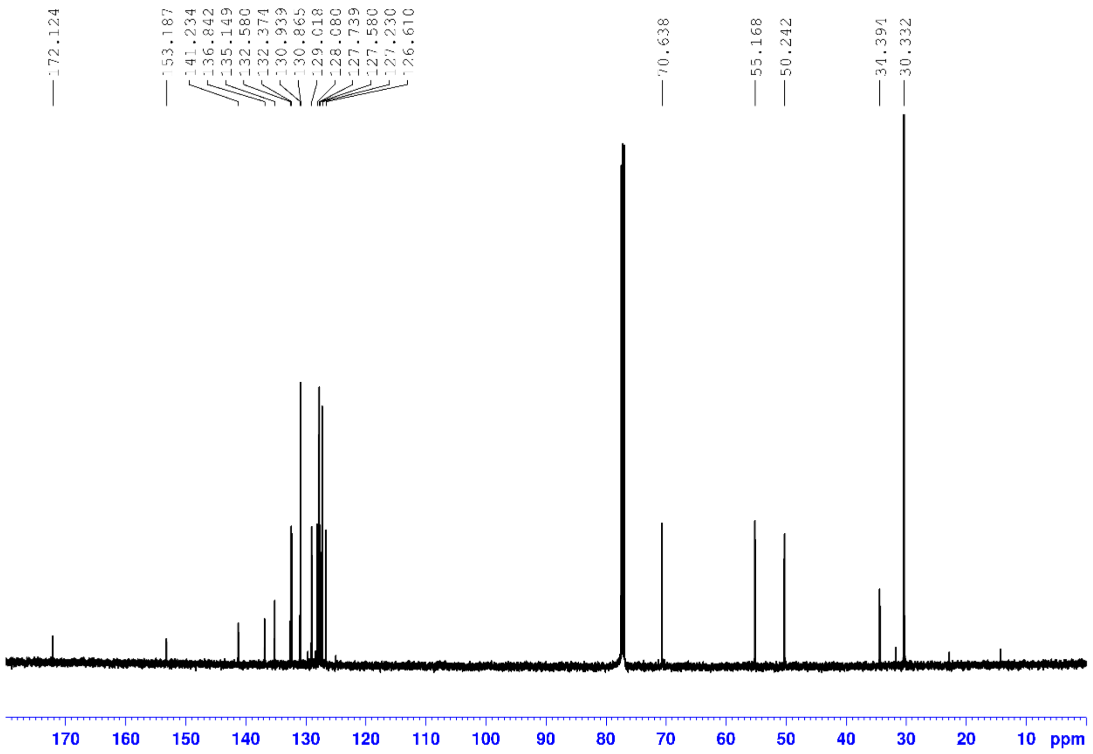

Cyclic trans product ***6a**^trans^*

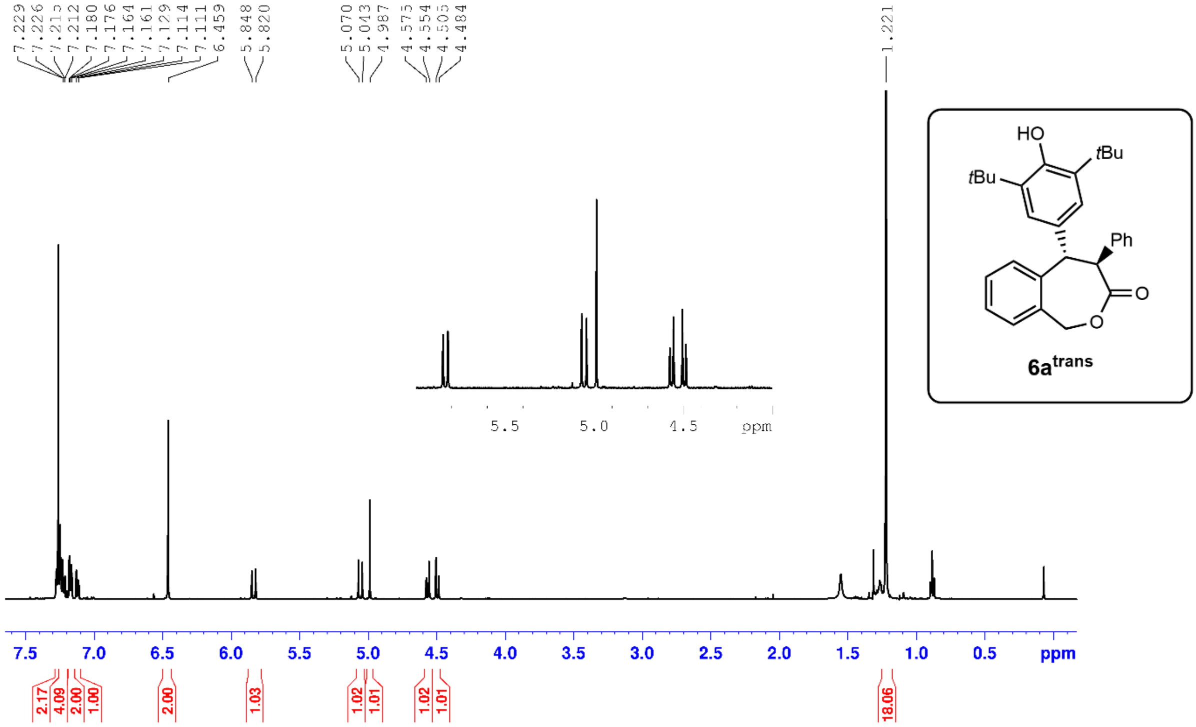

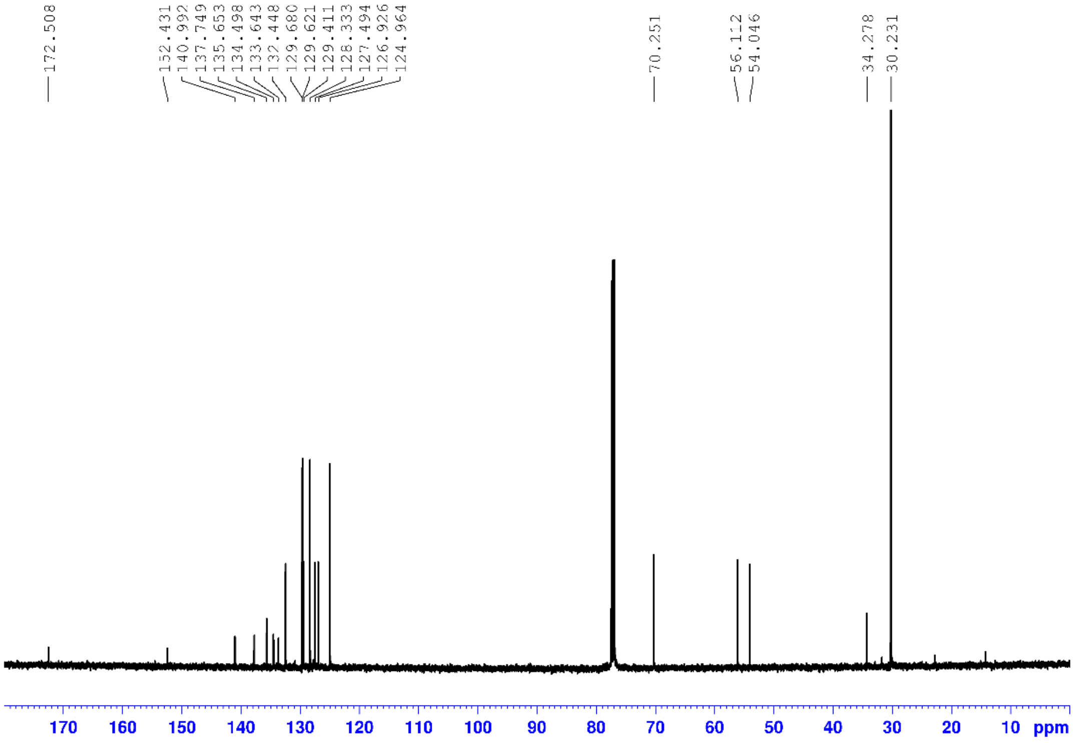

2-Fluorophenyl benzo[c]oxepinone derivative (6b)

*Alkylation Product **5b***

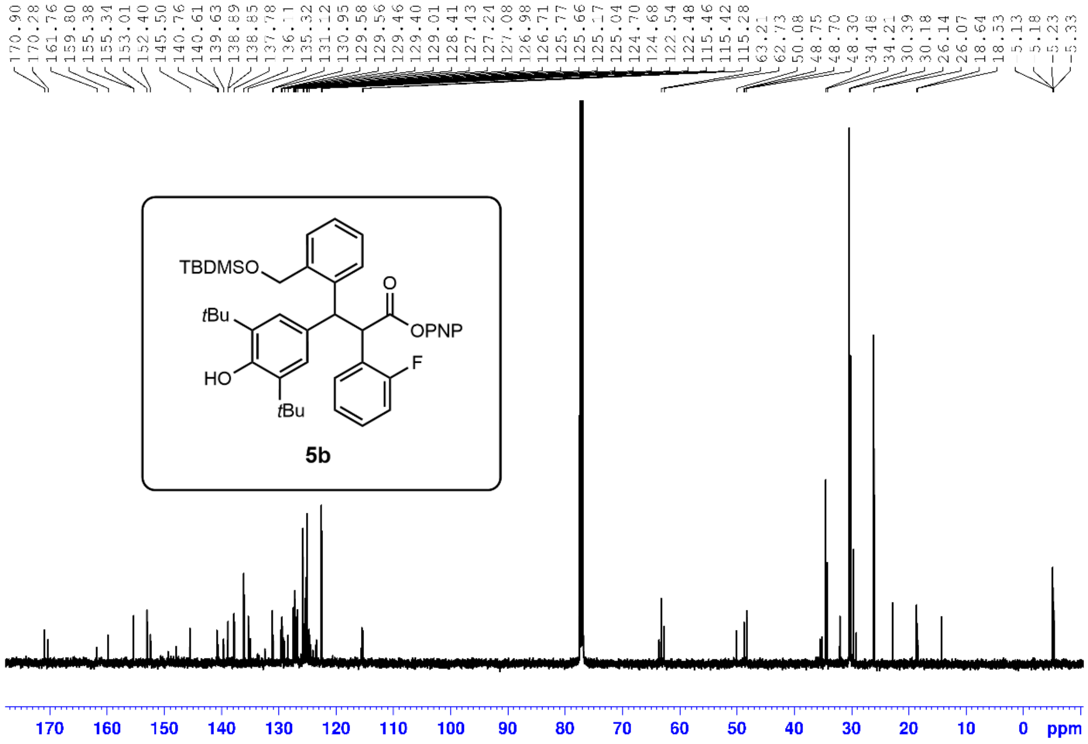

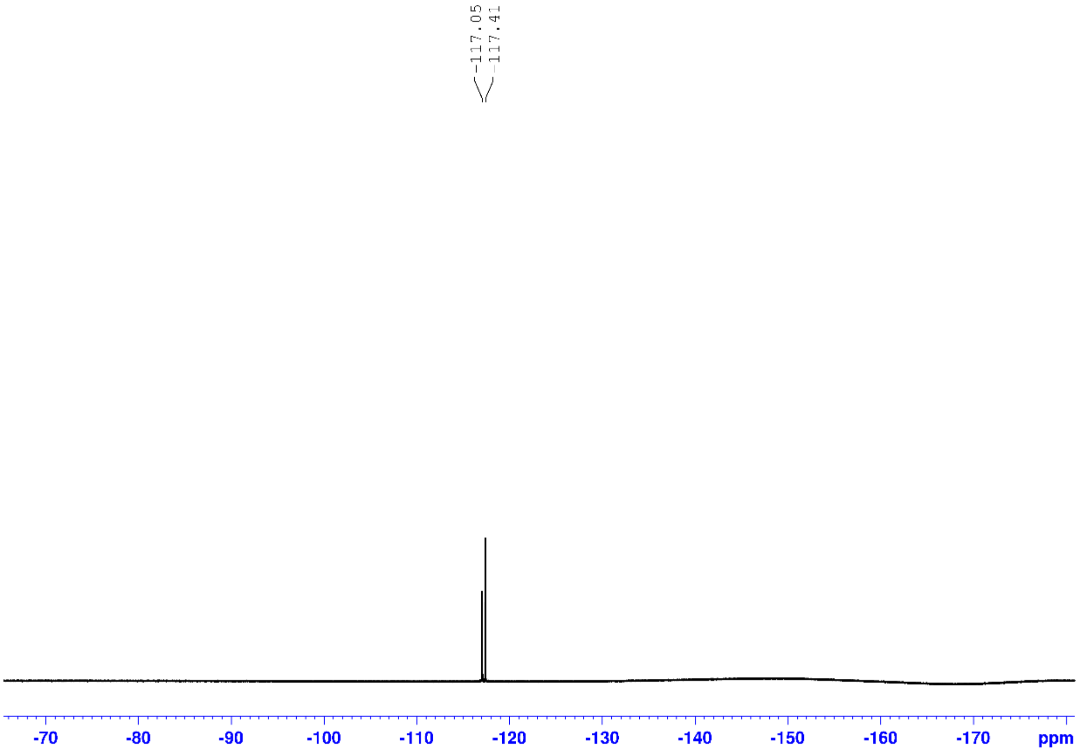

*Cyclic cis product **6b**^{cis}*

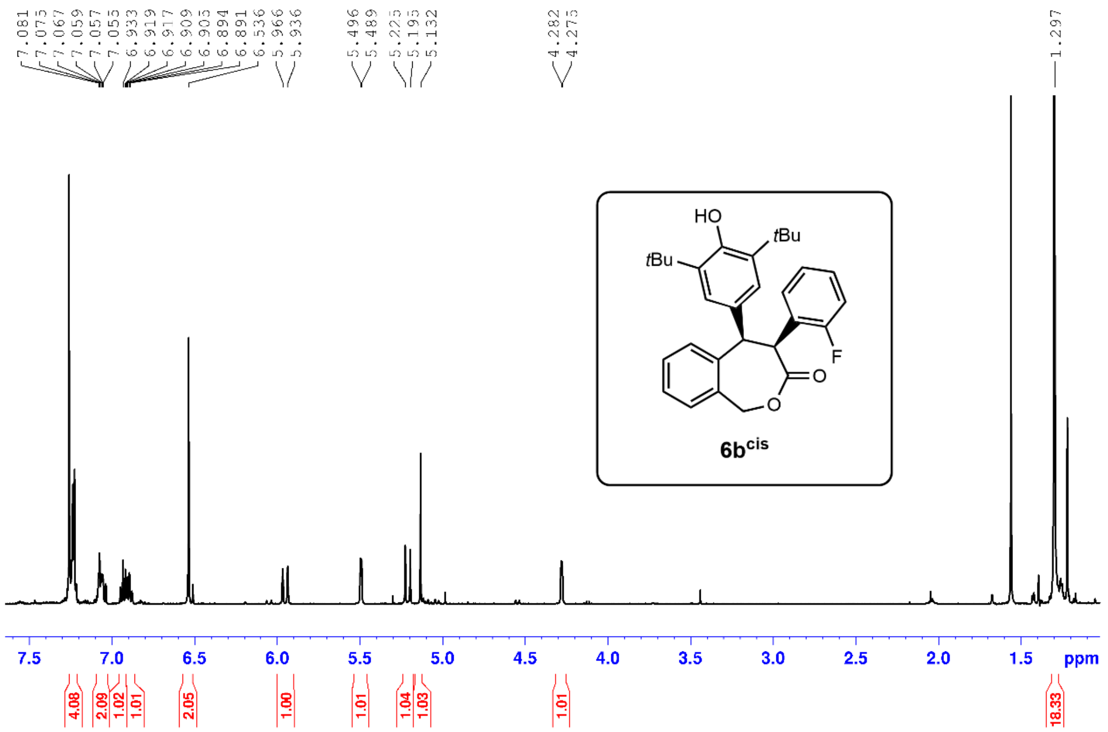

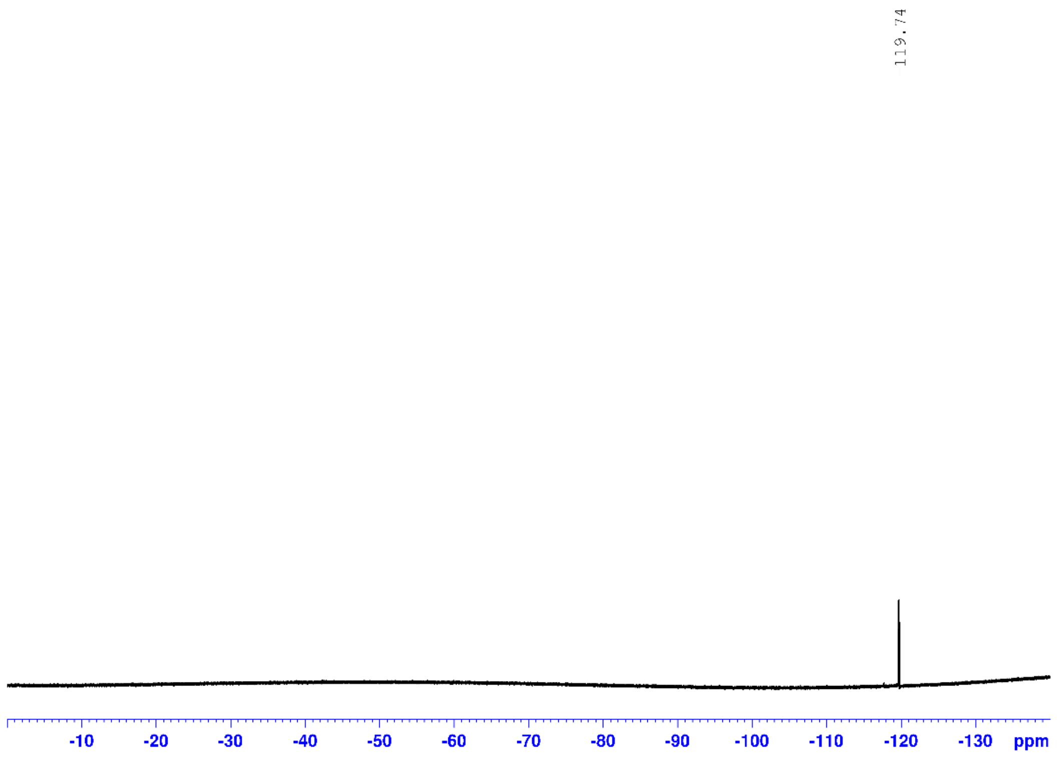

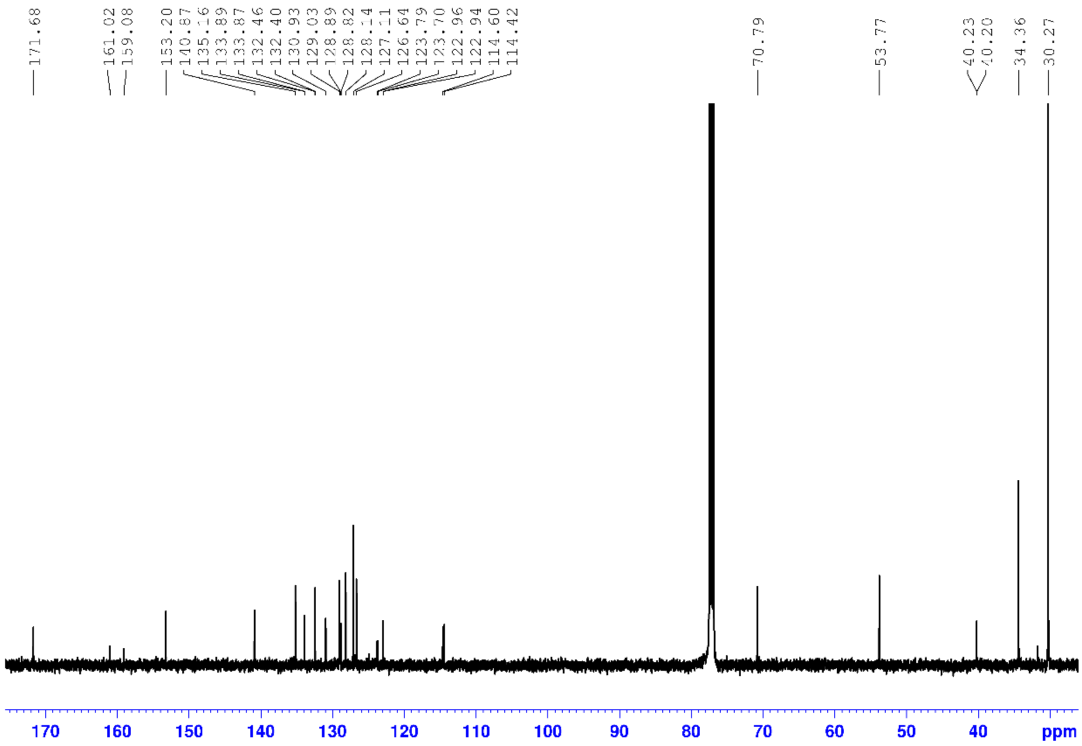

171.68
161.02
159.08
153.20
140.87
135.16
133.89
133.87
132.46
132.40
130.93
129.03
128.89
128.82
128.14
127.11
126.64
123.79
123.70
122.96
122.94
114.60
114.42
70.79
53.77
40.23
40.20
34.36
30.27
170
160
150
140
130
120
110
100
90
80
70
60
50
40
ppm

*Cyclic trans product **6a**^{trans}*

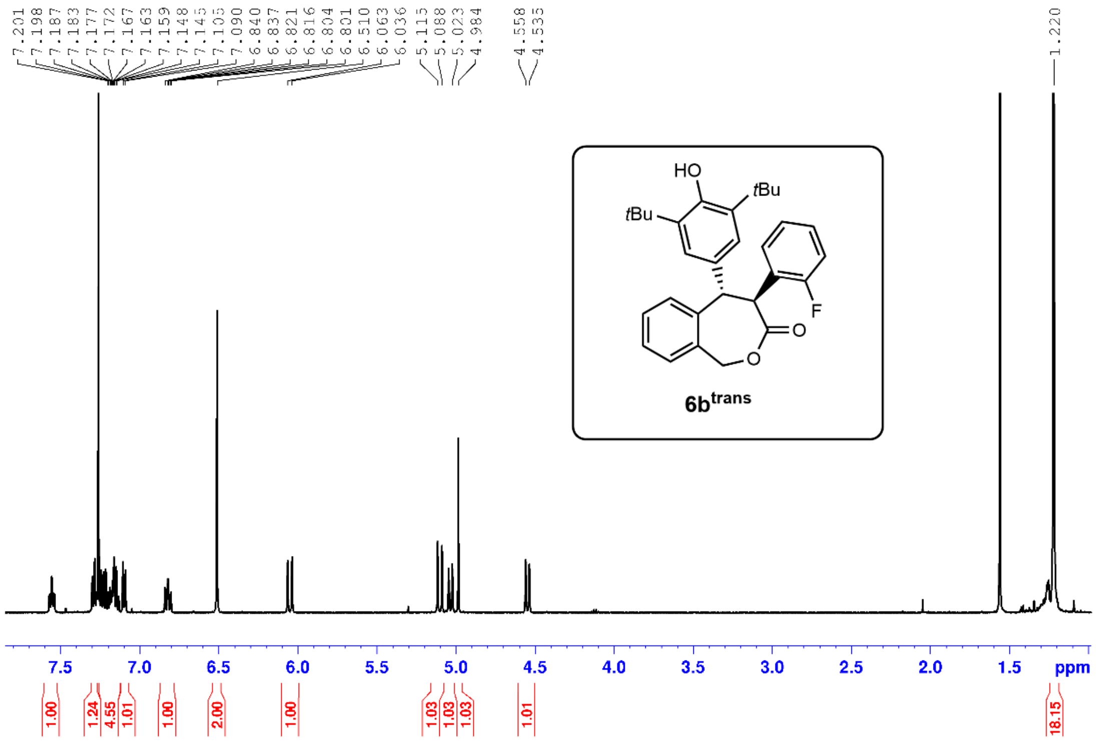

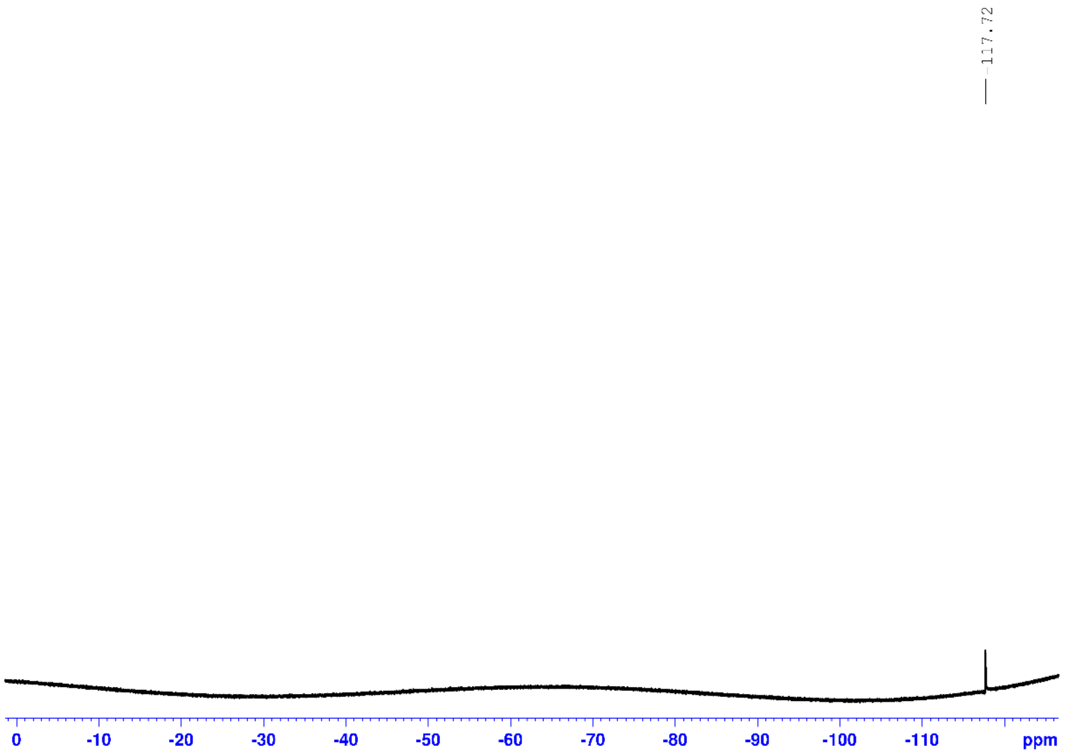

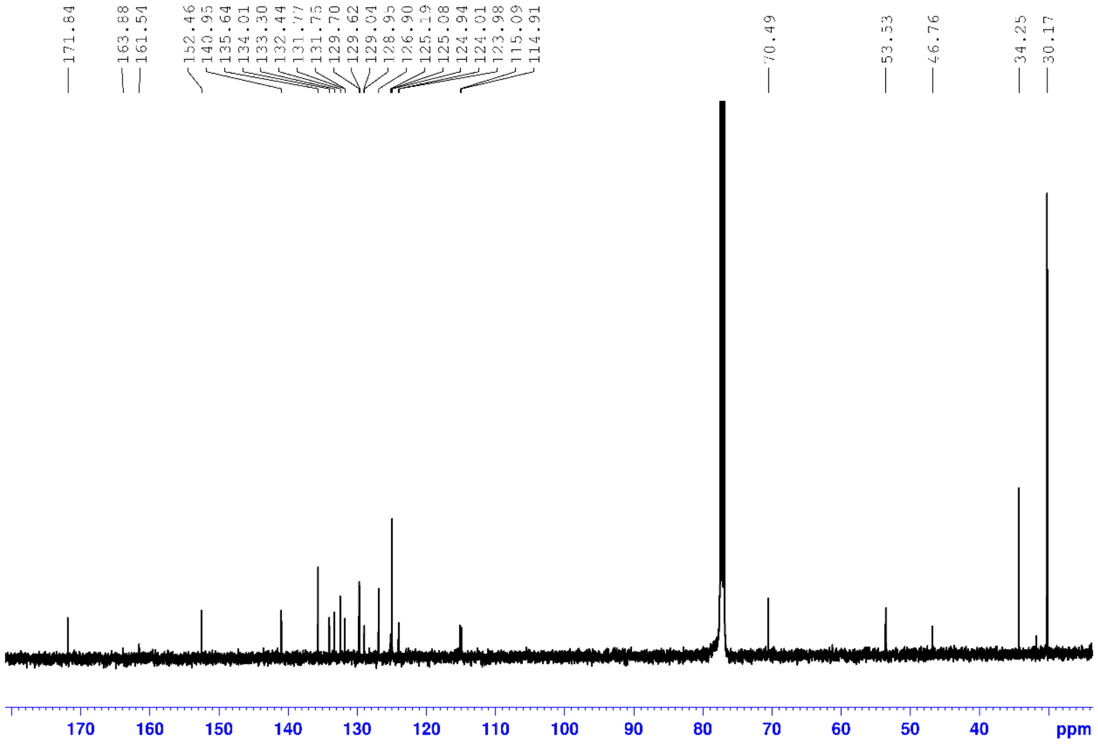
171.84
163.88
161.54
152.46
140.95
135.64
134.01
133.30
132.44
131.77
131.75
129.70
129.62
129.04
128.95
126.90
125.19
125.08
124.94
124.01
123.98
115.09
114.91
70.49
53.53
46.76
34.25
30.17
170
160
150
140
130
120
110
100
90
80
70
60
50
40
ppm

4-Fluorophenyl benzo[c]oxepinone derivative (6c)

Alkylation Product ***5c***

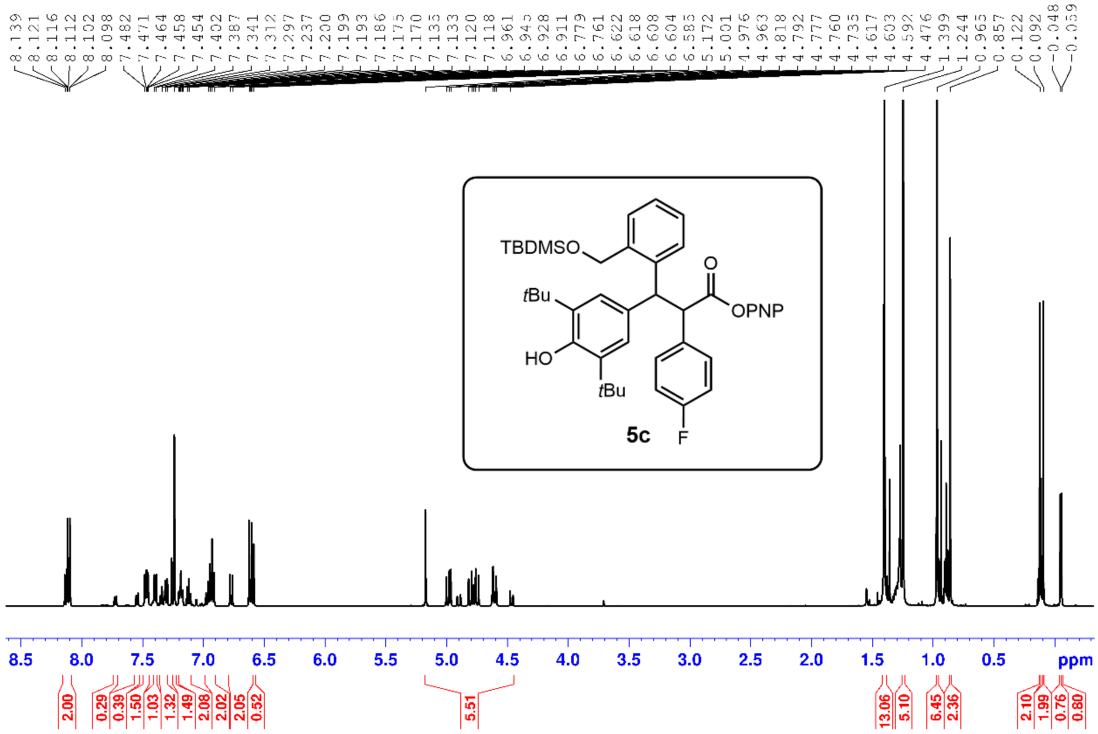

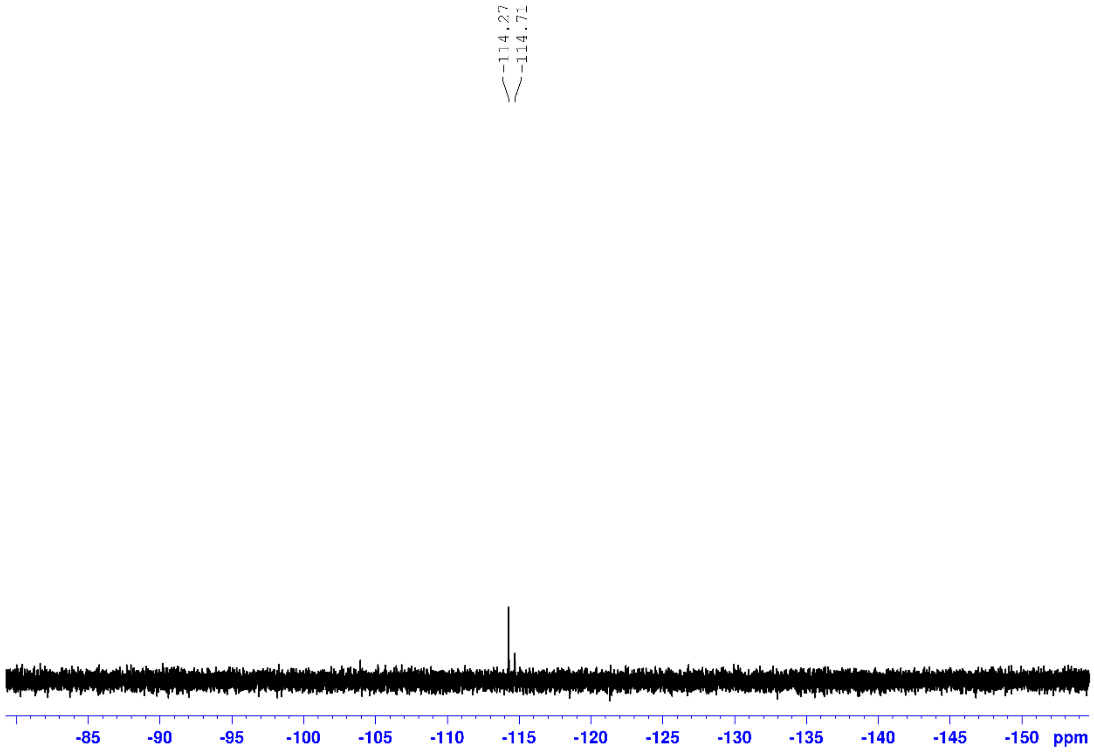

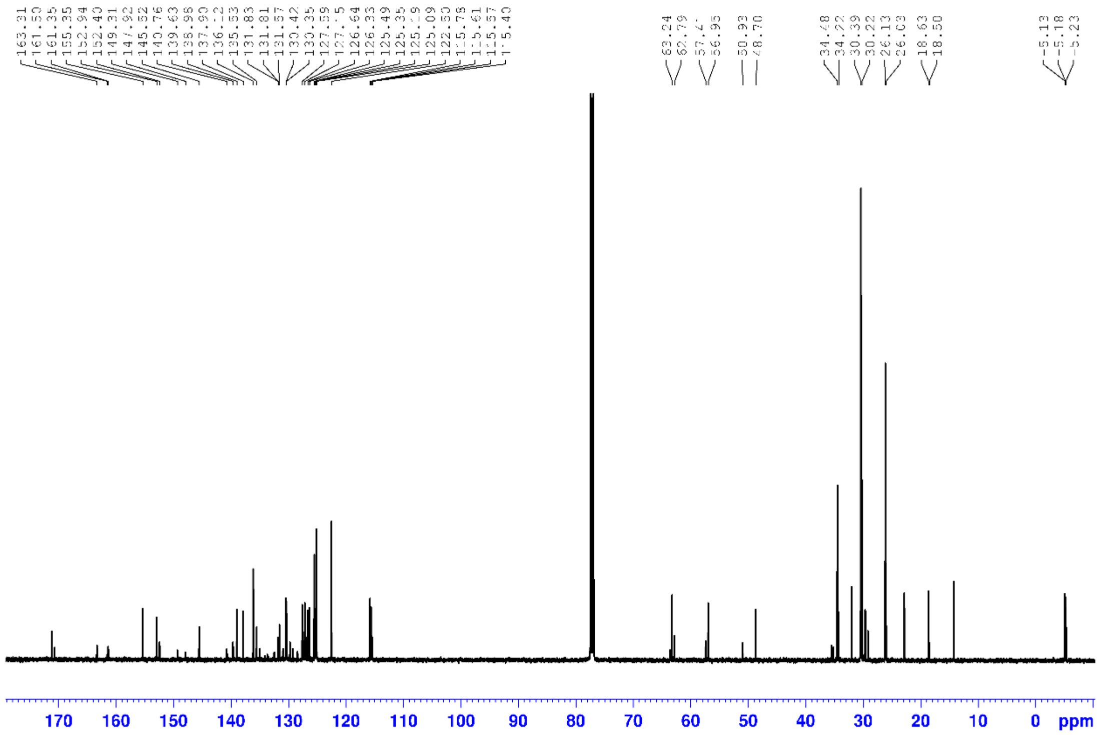
163.31
161.50
161.35
155.35
152.94
152.40
149.31
145.52
140.76
139.63
138.98
137.90
135.53
131.83
131.81
131.57
130.42
130.35
127.59
126.64
126.33
125.49
125.35
125.09
122.50
63.24
62.79
56.95
50.93
48.70
30.39
30.22
26.13
26.03
18.63
18.50
-5.13
-5.18
-5.23
170
160
150
140
130
120
110
100
90
80
70
60
50
40
30
20
10
0
ppm

*Cyclic cis product **6c**^{cis}*

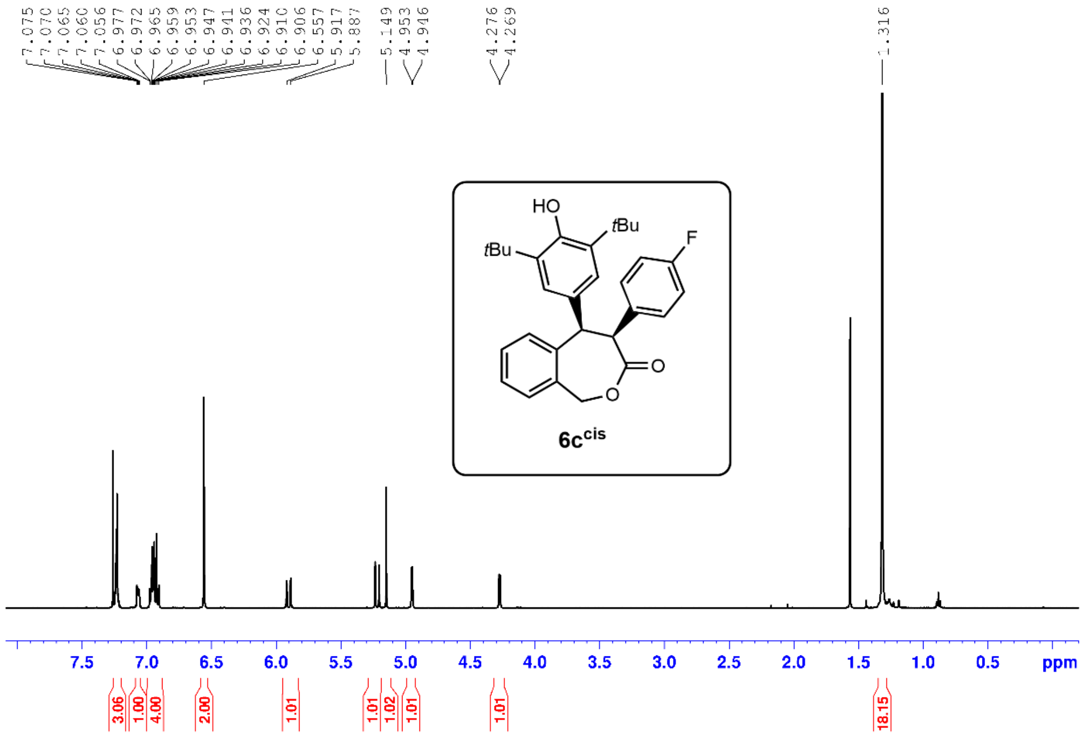

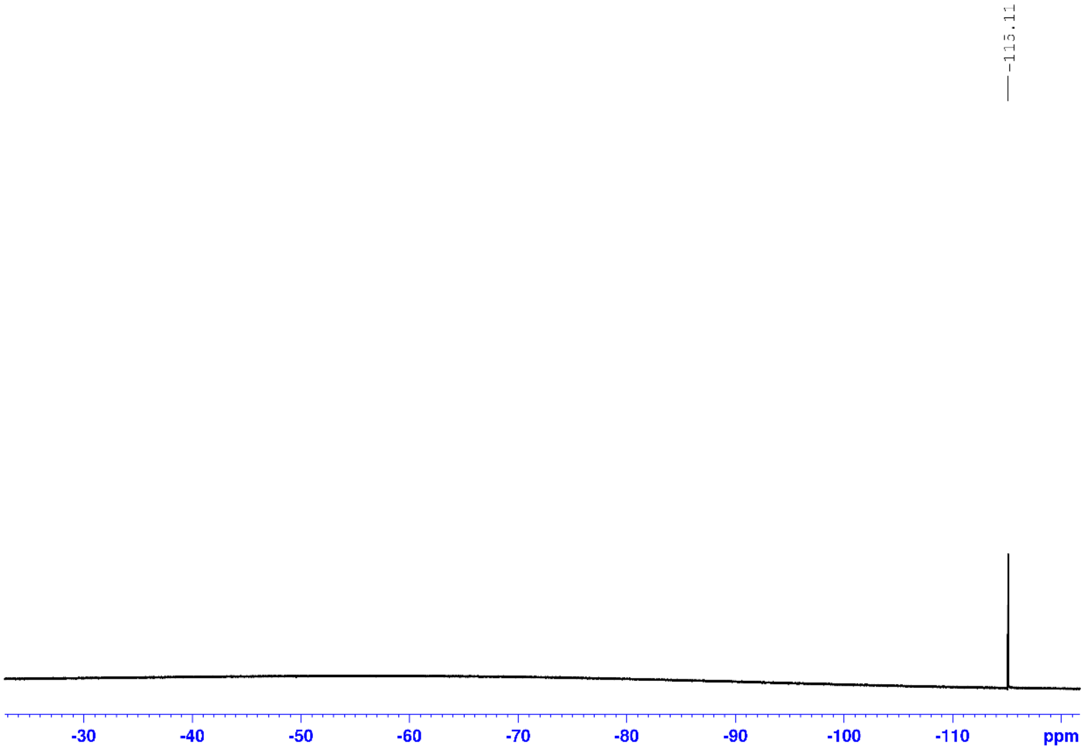

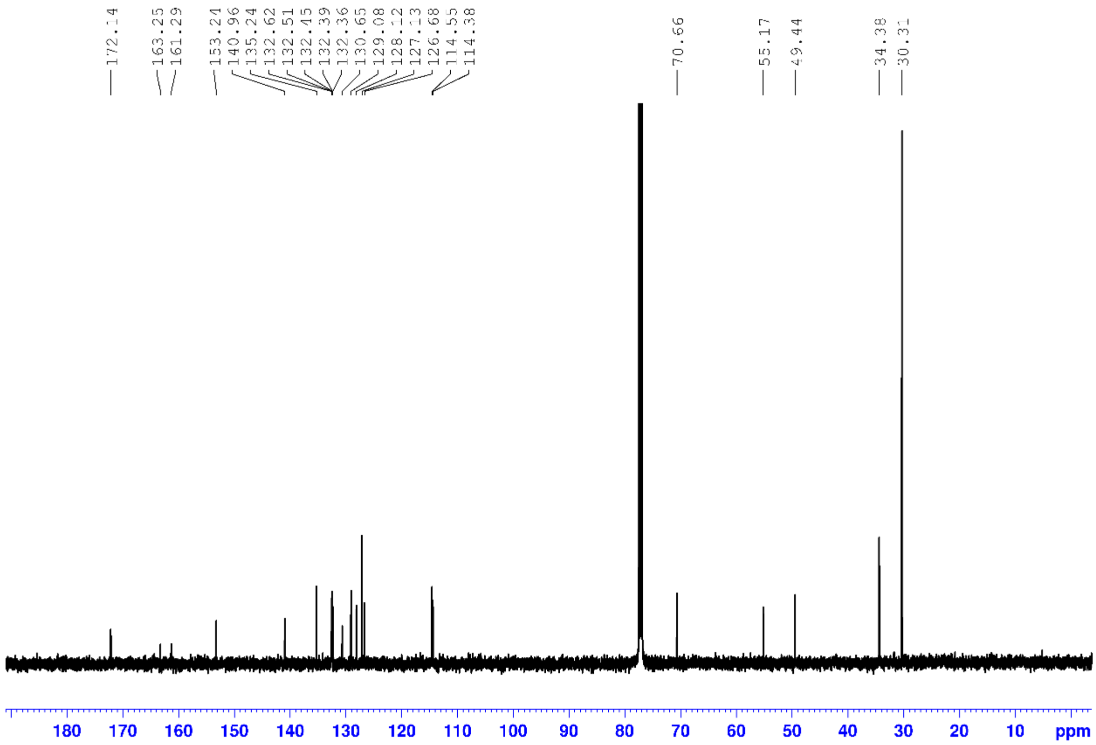
172.14
163.25
161.29
153.24
140.96
135.24
132.62
132.51
132.45
132.39
132.36
130.65
129.08
128.12
127.13
126.68
114.55
114.38
70.66
55.17
49.44
34.38
30.31
180
170
160
150
140
130
120
110
100
90
80
70
60
50
40
30
20
10
ppm

*Cyclic trans product **6c**^{trans}*

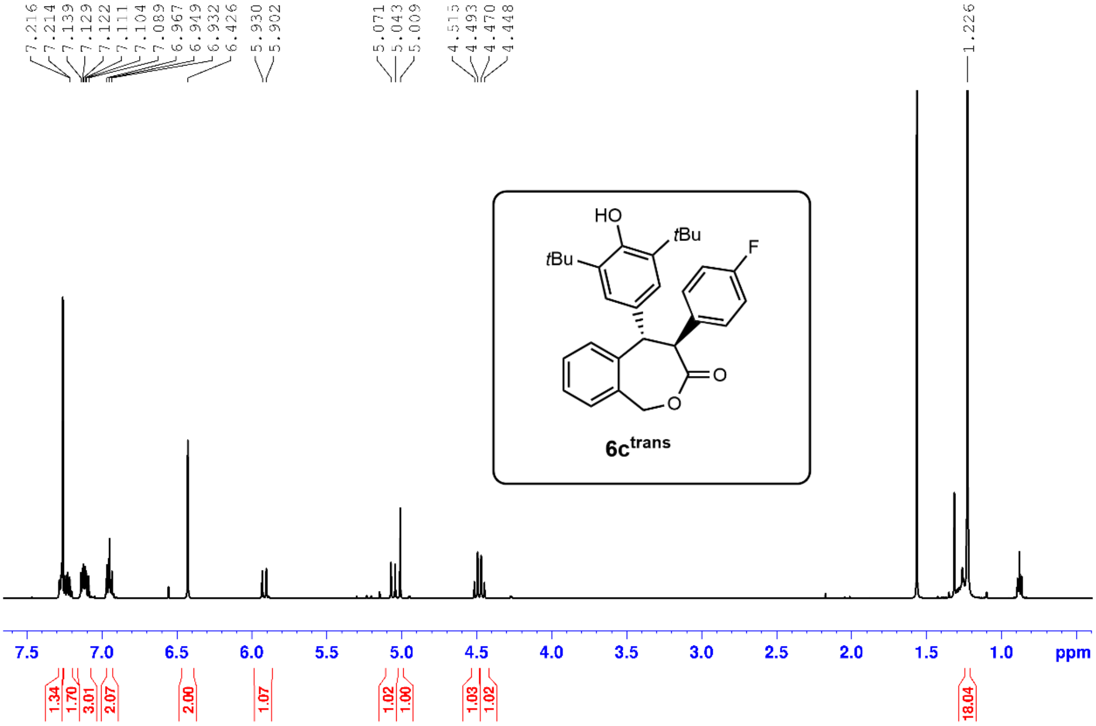

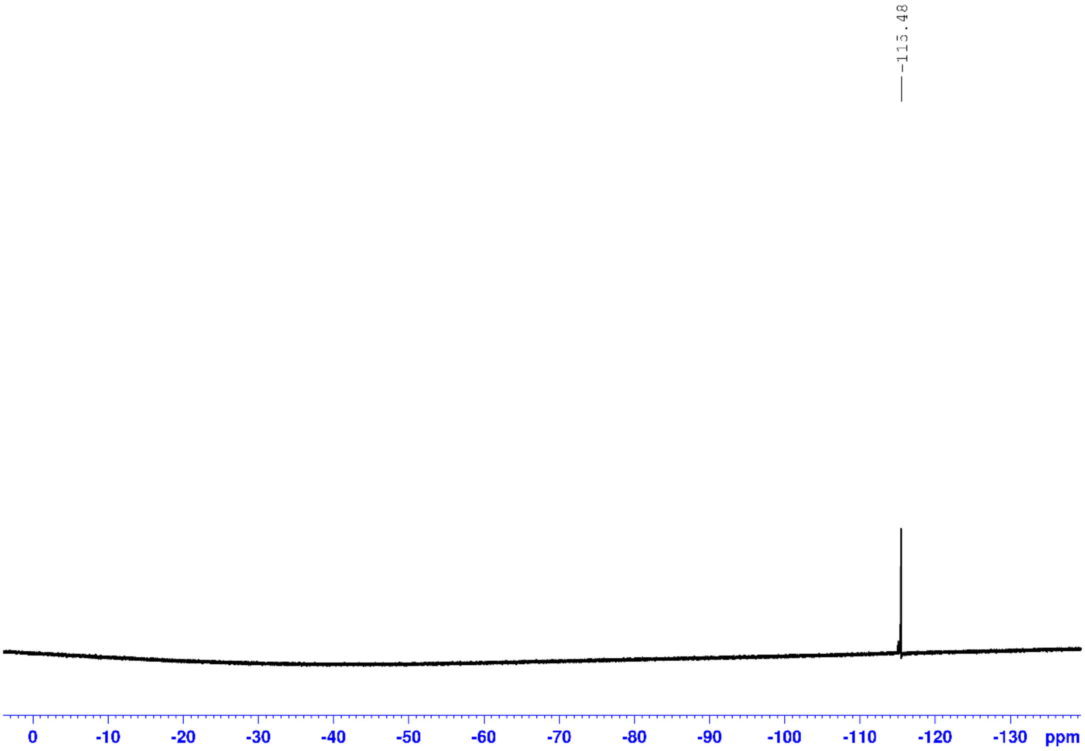

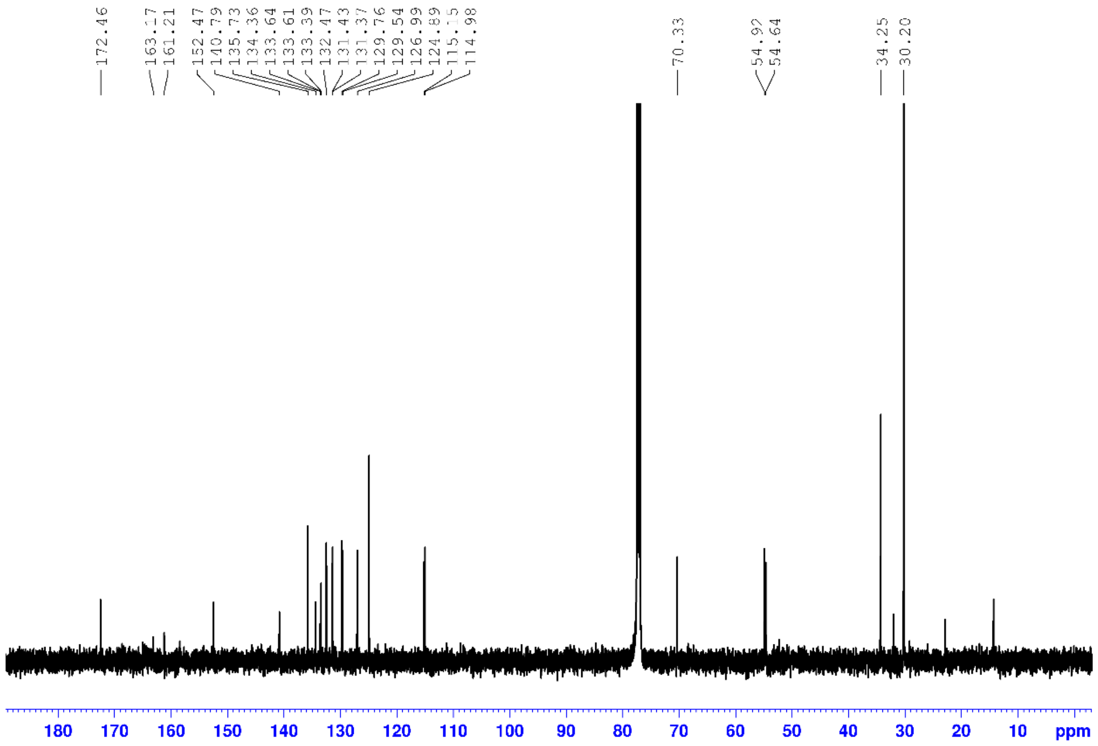

172.46
163.17
161.21
152.47
140.79
135.73
134.36
133.64
133.61
133.39
132.47
131.43
131.37
129.76
129.54
126.99
124.89
115.15
114.98
70.33
54.92
54.64
34.25
30.20
180
170
160
150
140
130
120
110
100
90
80
70
60
50
40
30
20
10
ppm

3-Chlorophenyl benzo[c]oxepinone derivative (6d)

*Alkylation Product **5d***

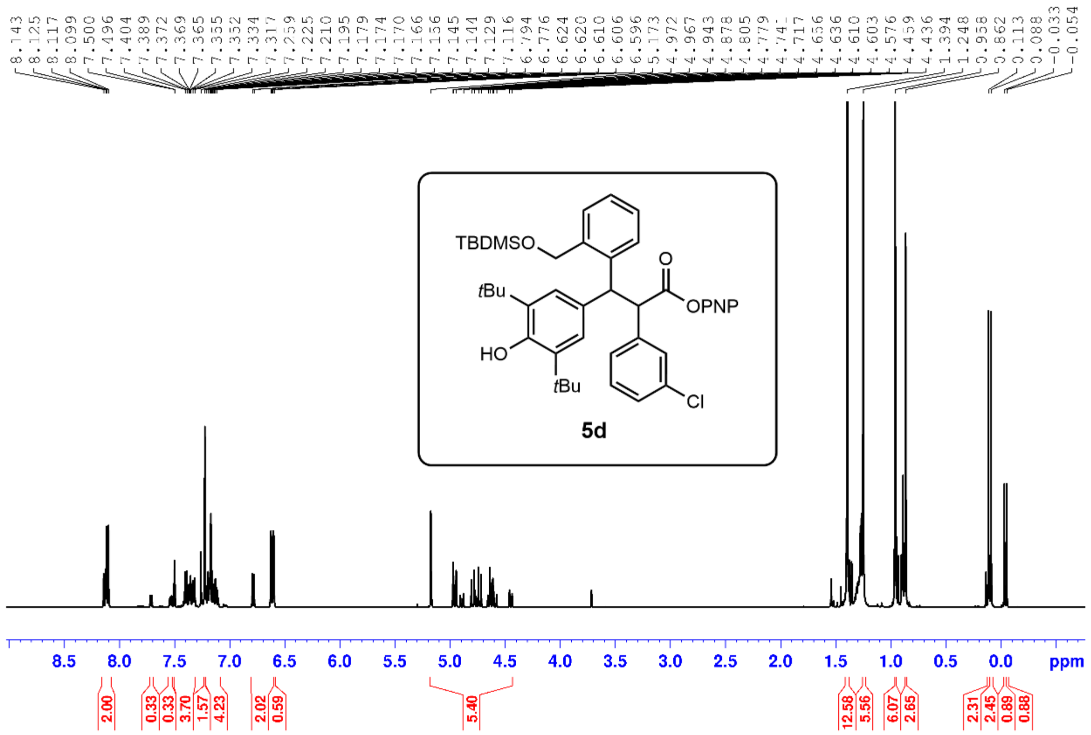

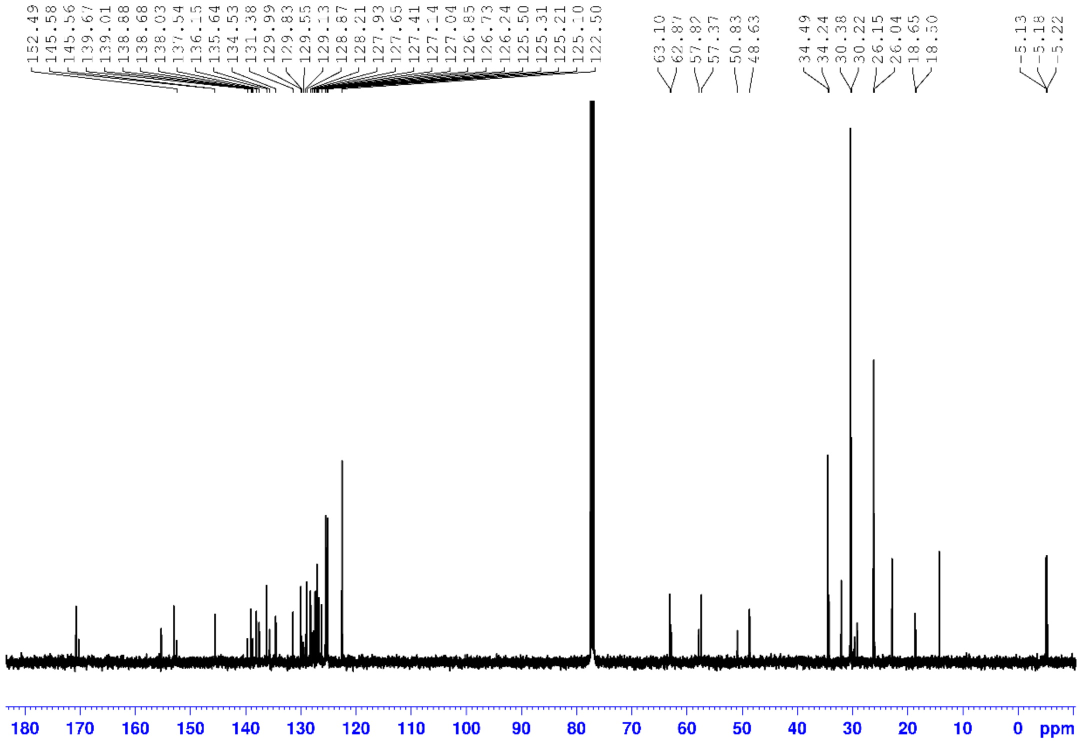

Cyclic cis product ***6d***$^{\textbf{\textit{cis}}}$

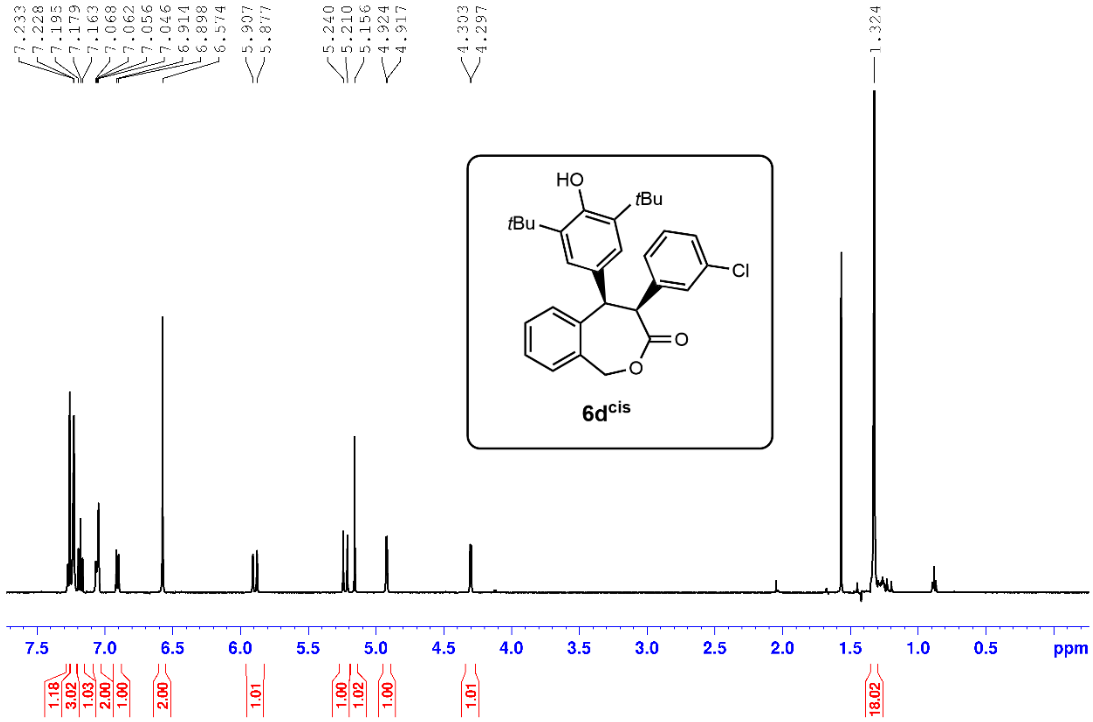

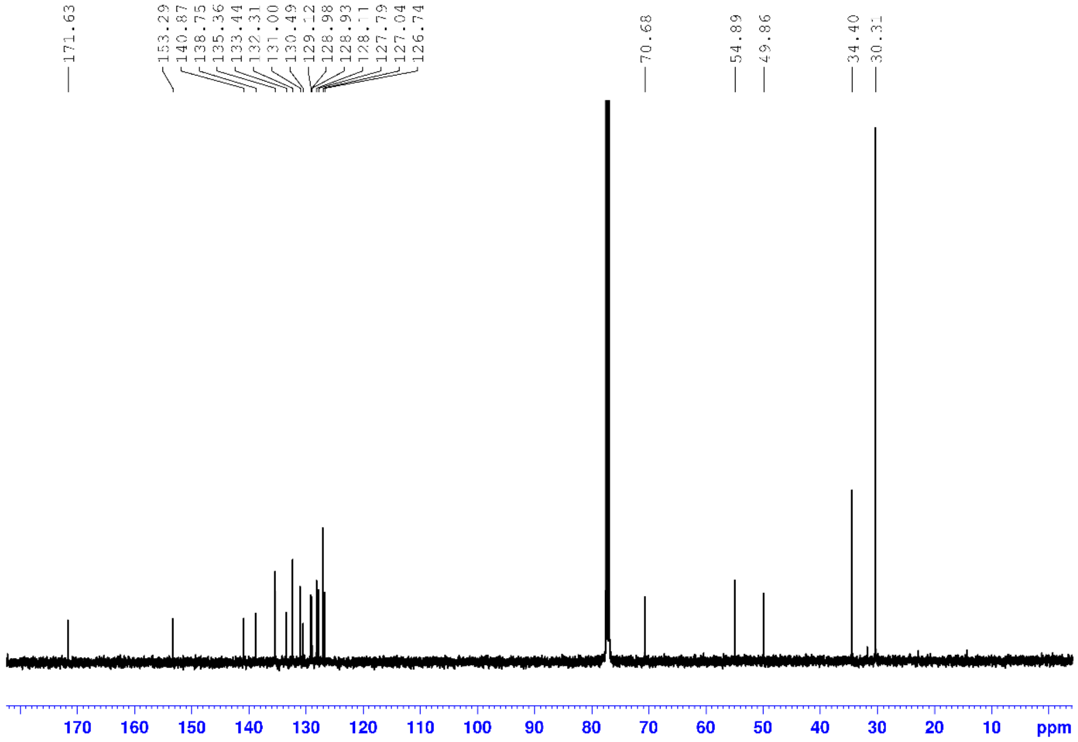

*Cyclic trans product **6d**^{trans}*

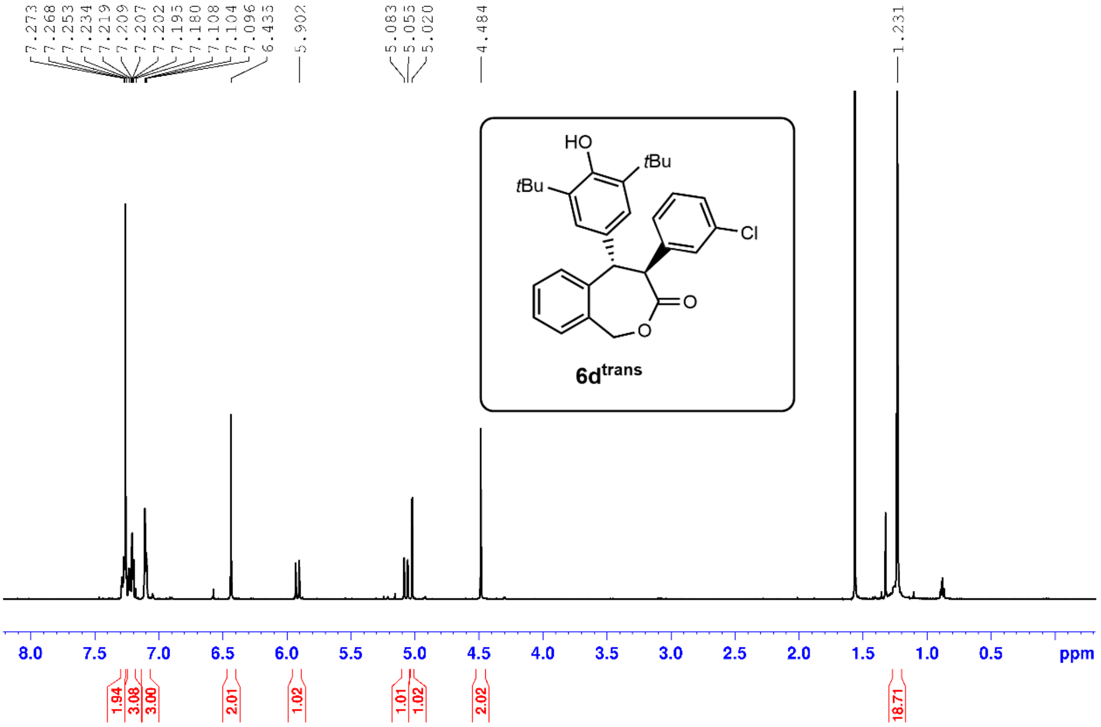

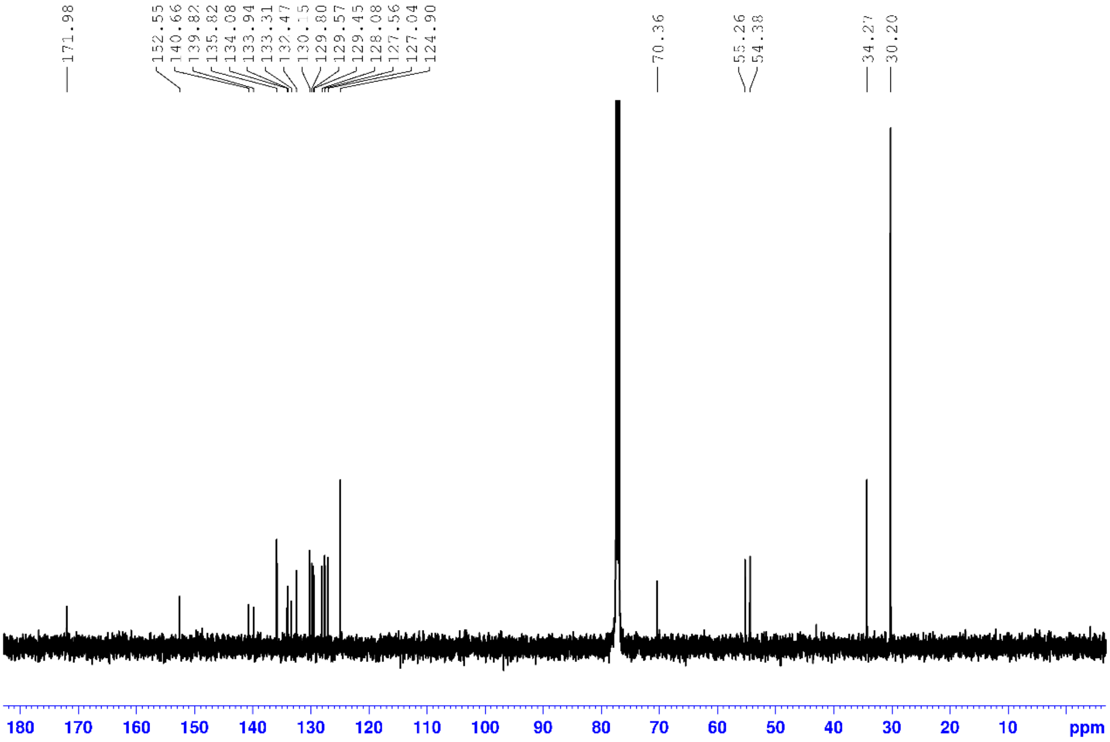

4-Chlorophenyl benzo[c]oxepinone derivative (6e)

Alkylation Product ***5e***

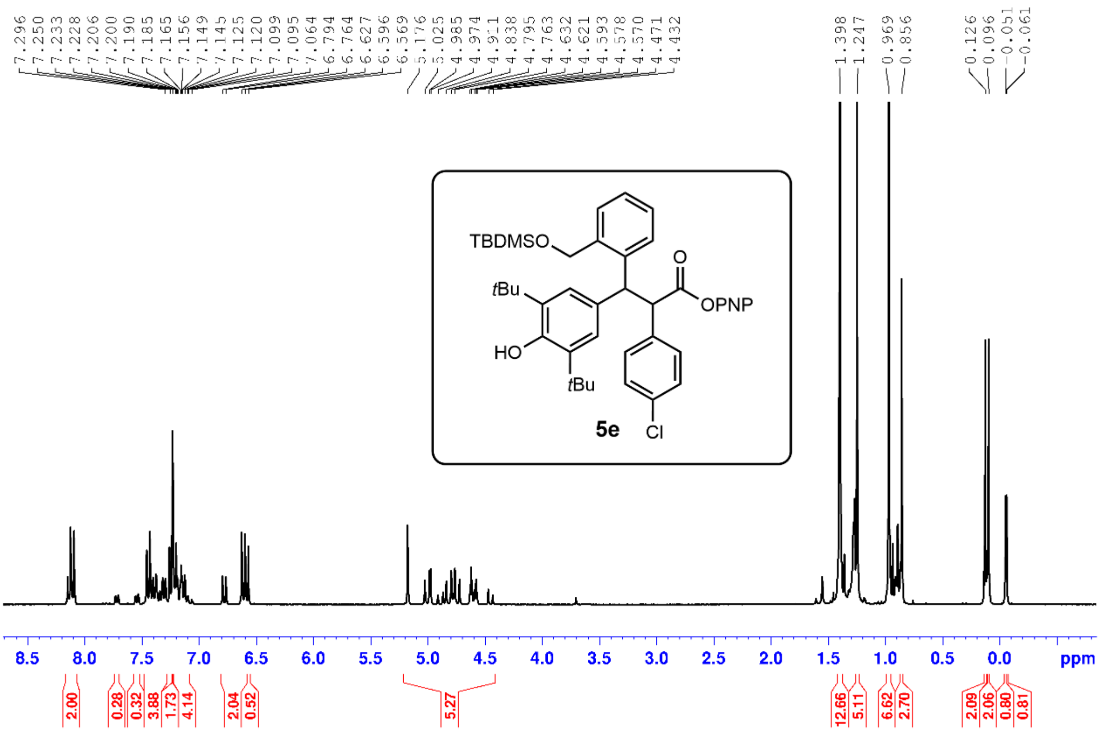

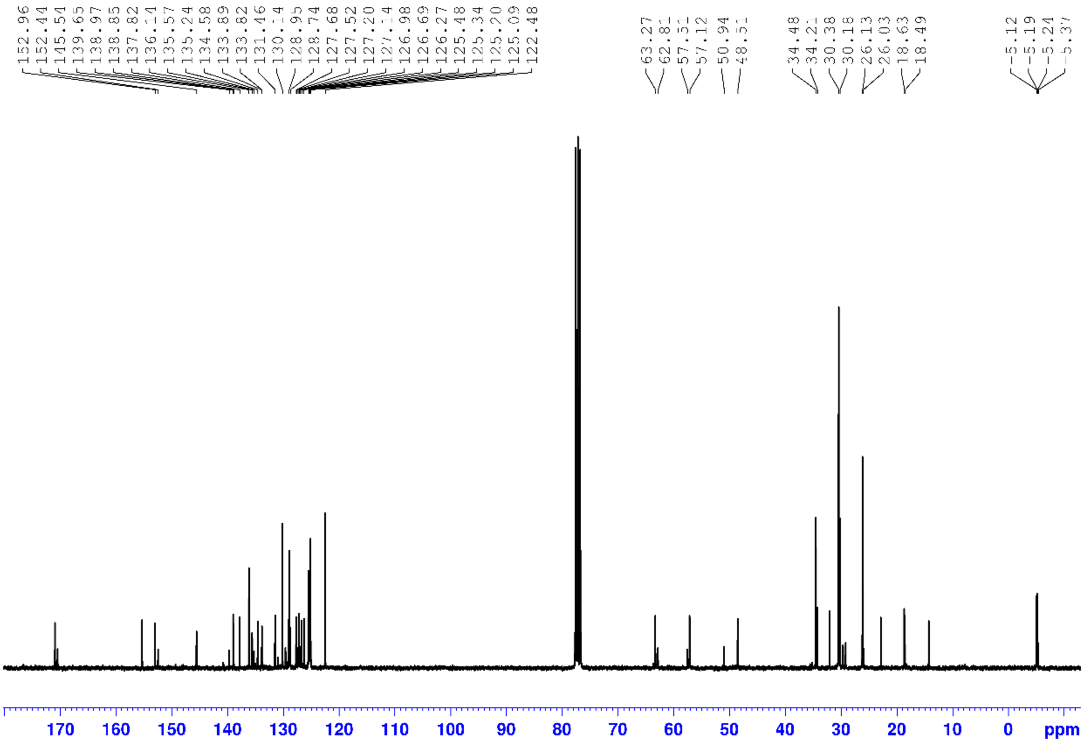

*Cyclic cis product **6e**^{cis}*

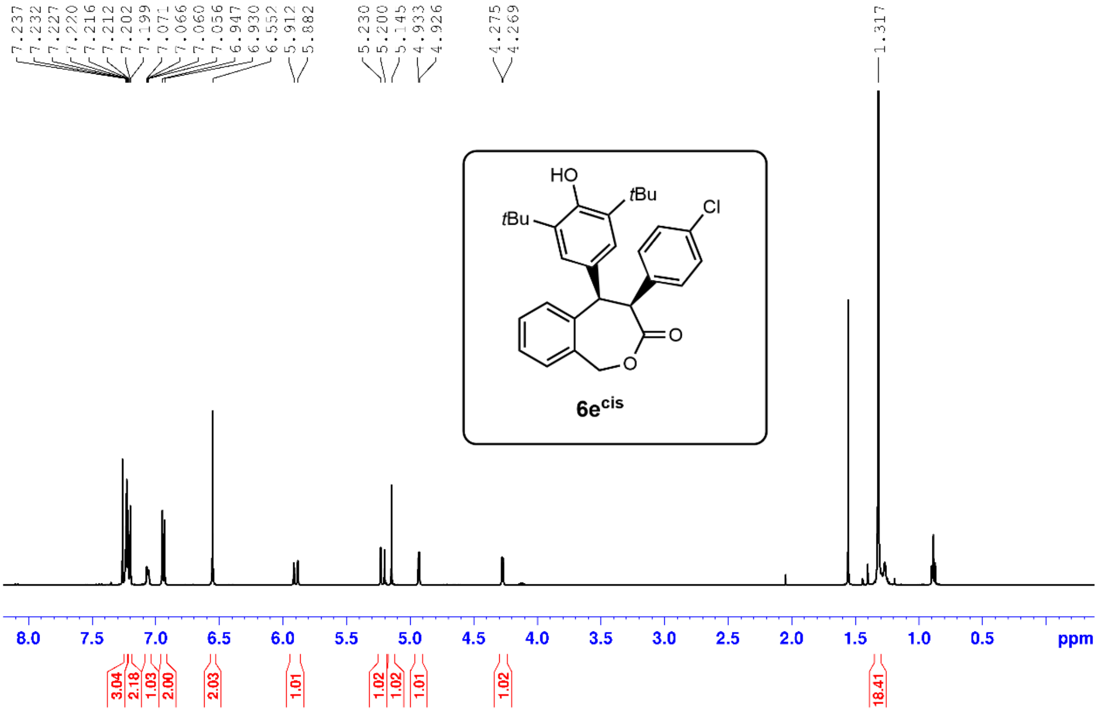

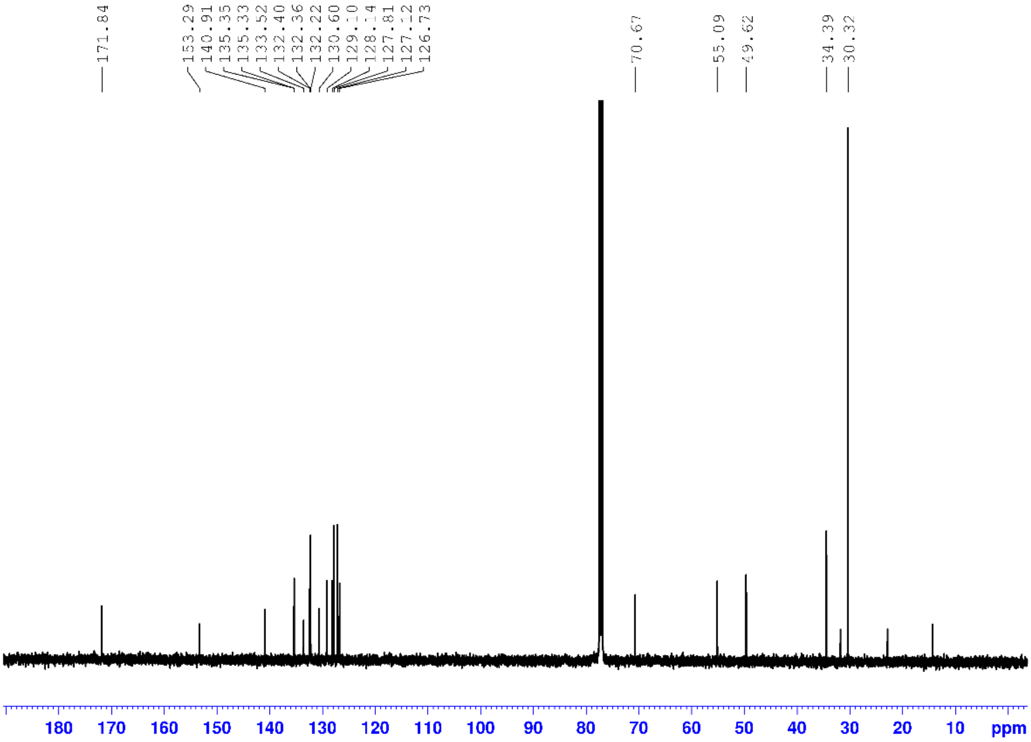

Cyclic trans product ***6e***^{trans}

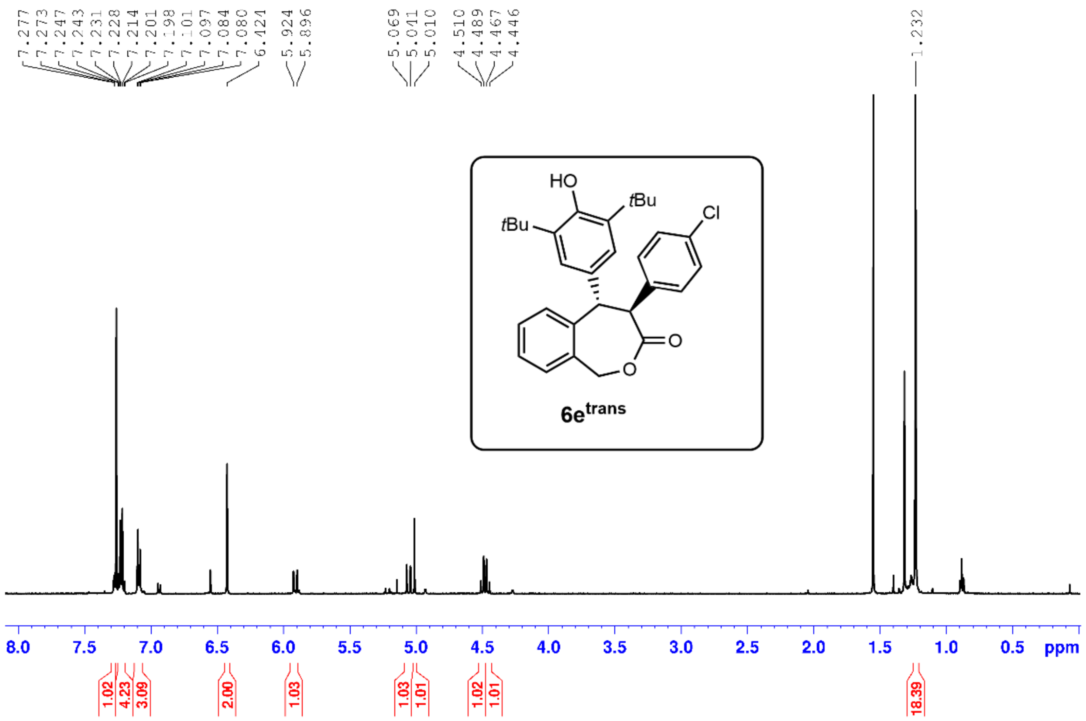

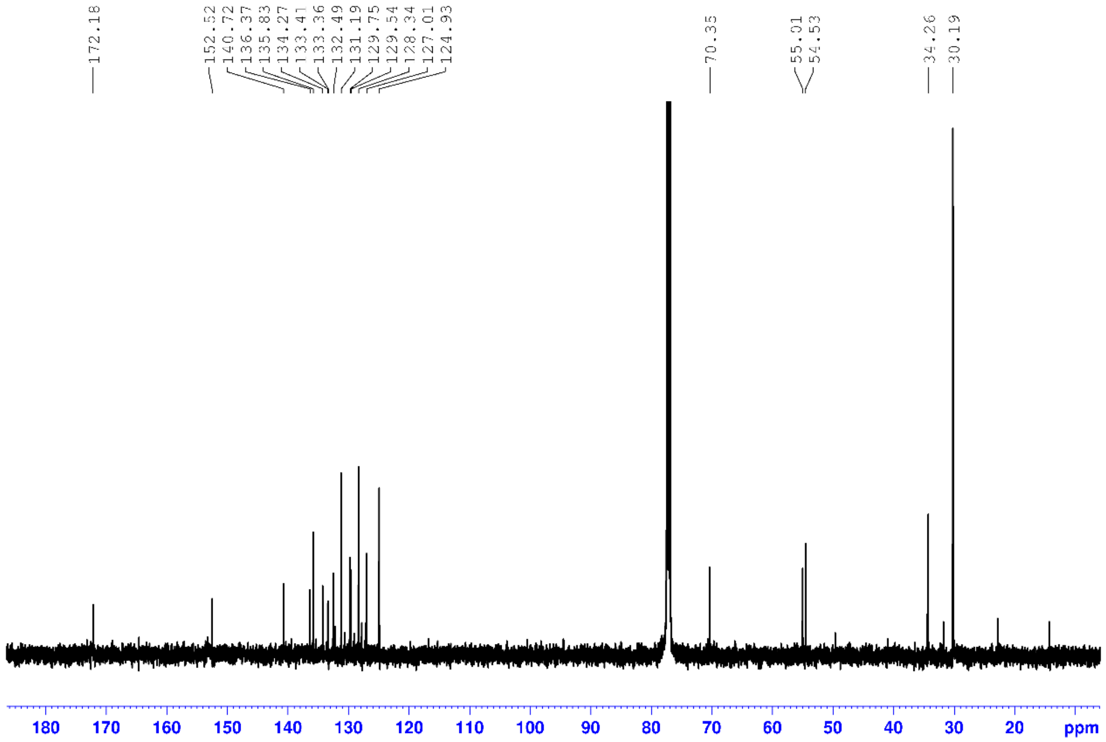

Dichlorophenyl benzo[c]oxepinone derivative (6f)

*Alkylation Product **5f***

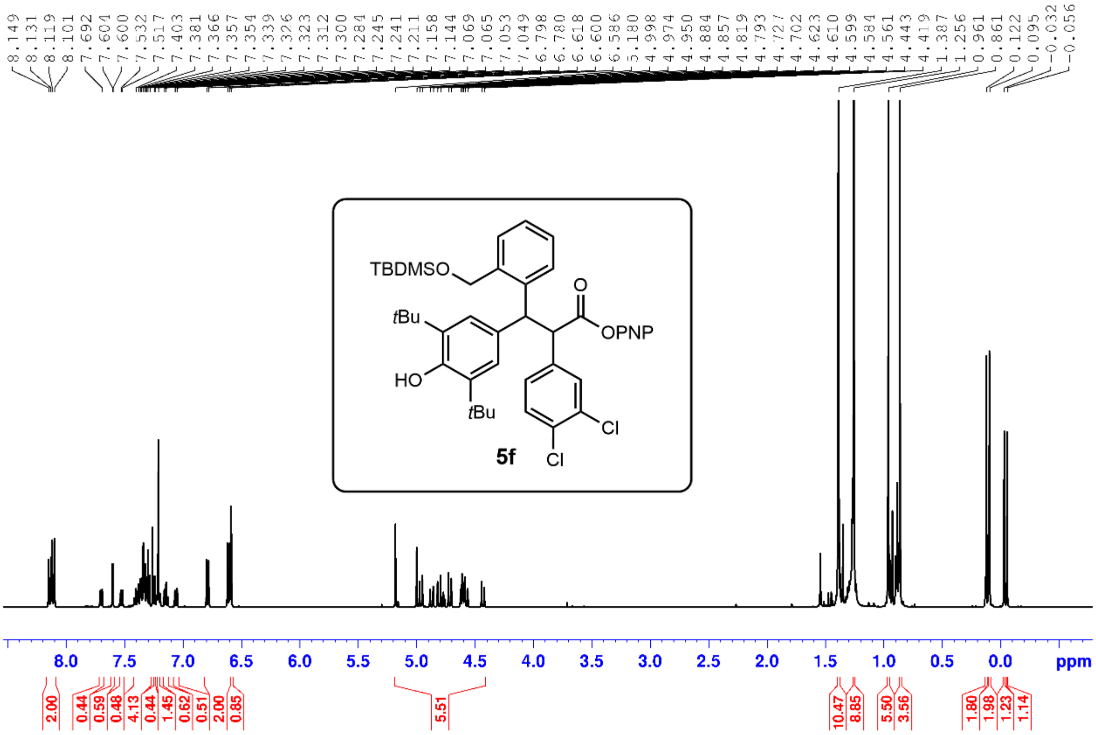

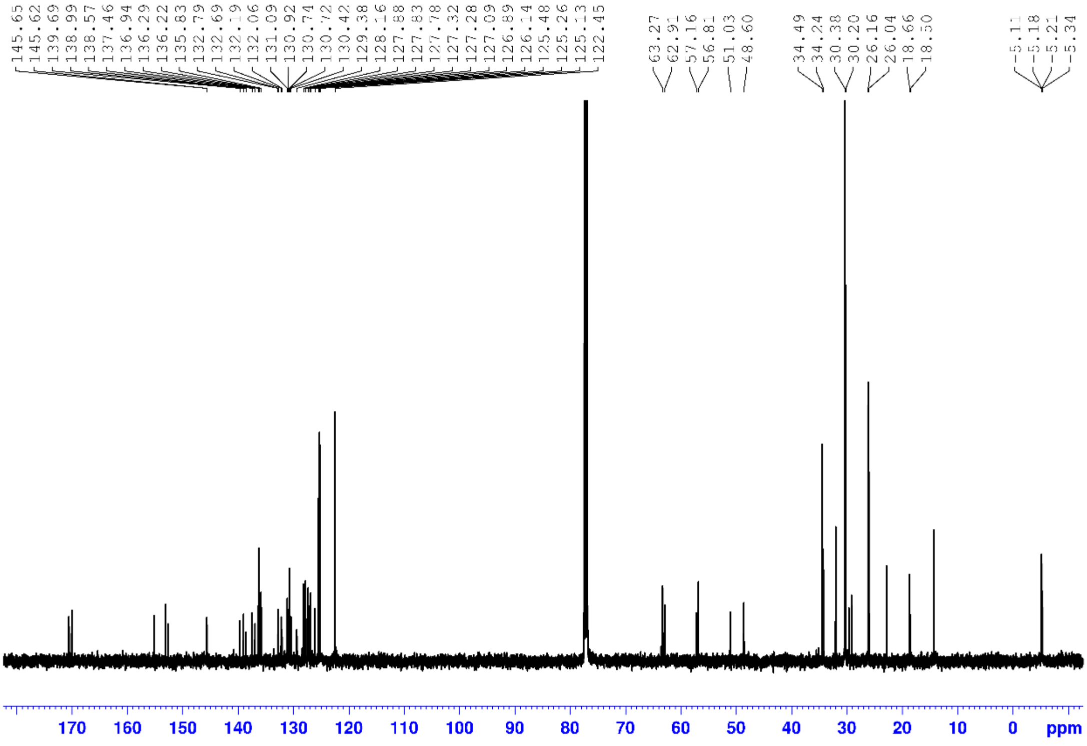

*Cyclic cis product **6f**^{cis}*

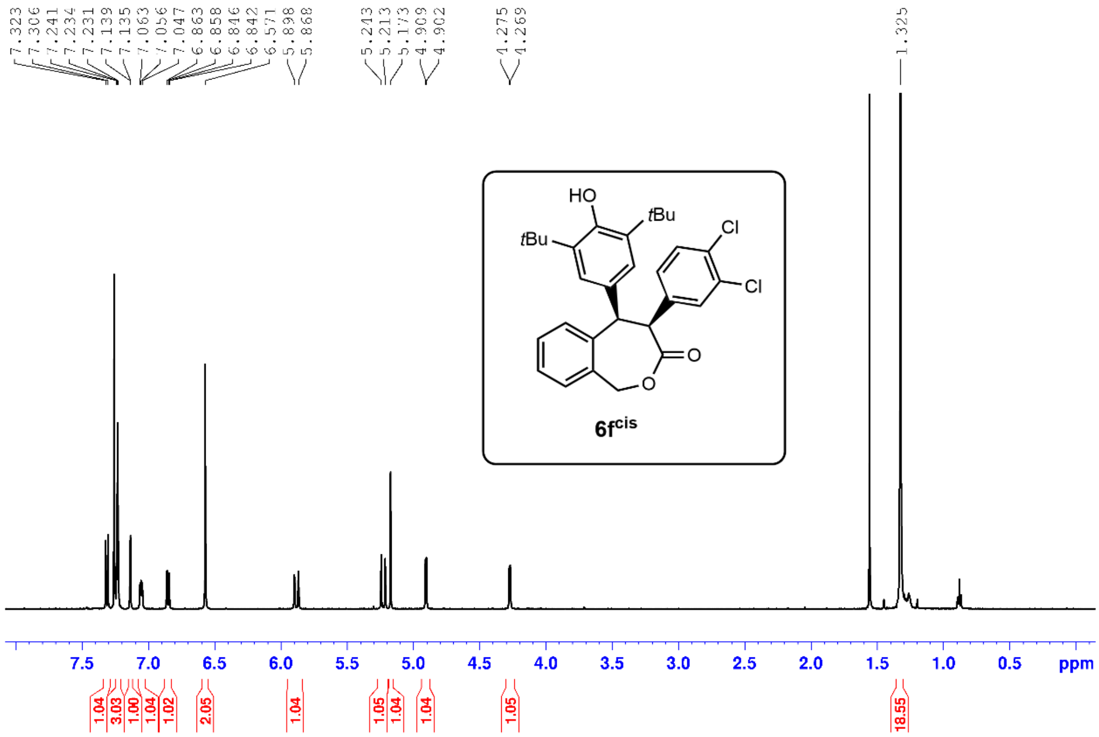

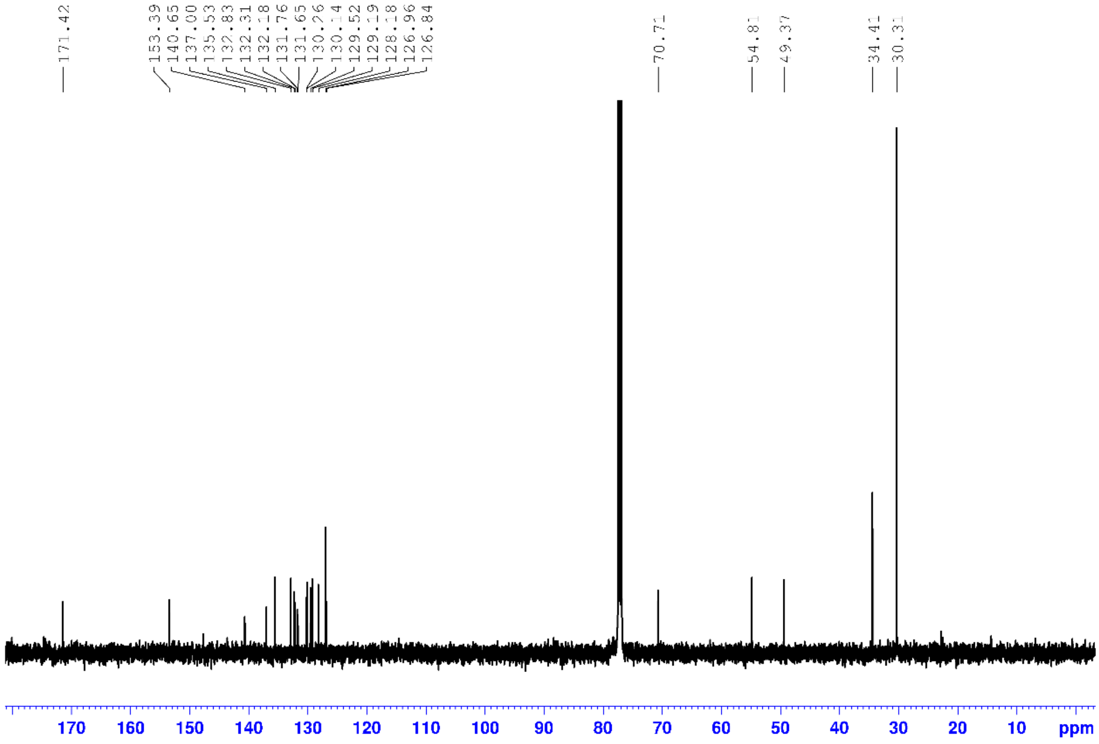

*Cyclic trans product **6f**^{trans}*

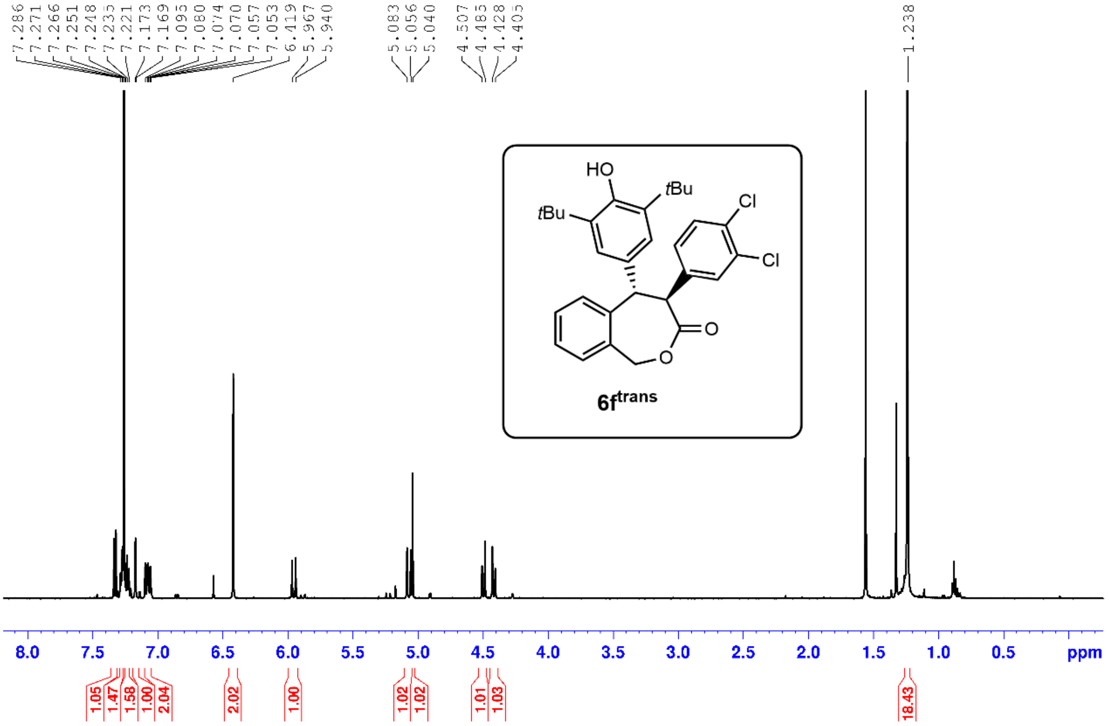

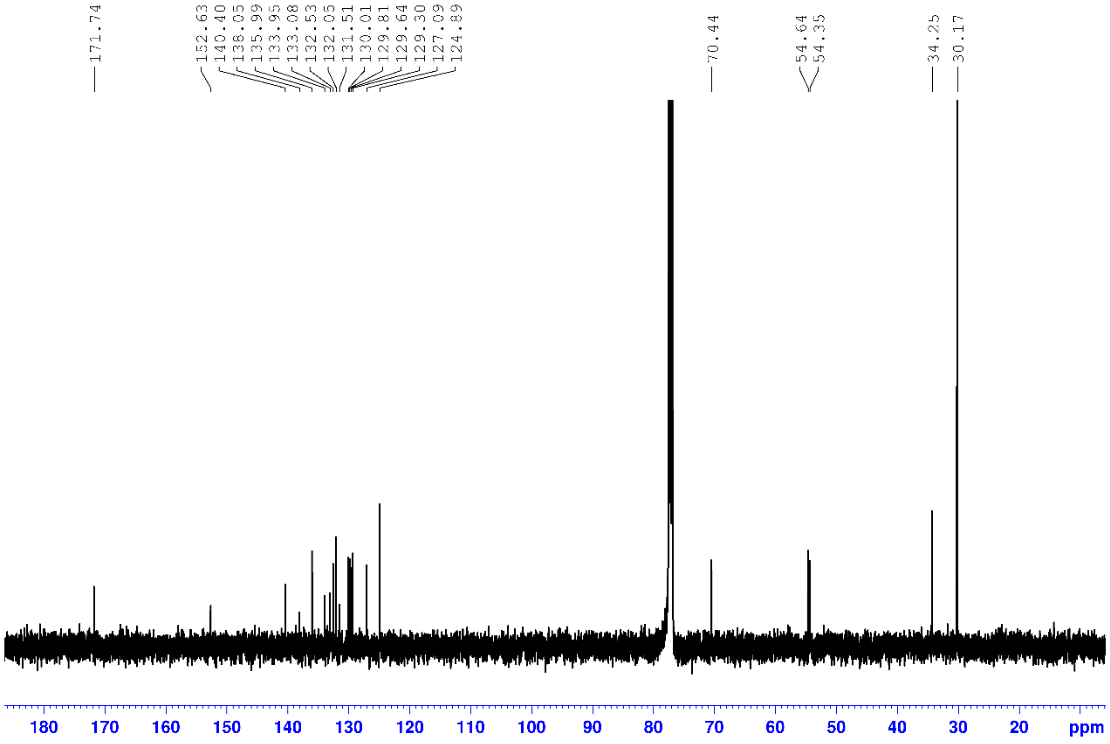

4-Bromophenyl benzo[c]oxepinone derivative (6g)

Alkylation Product ***5g***

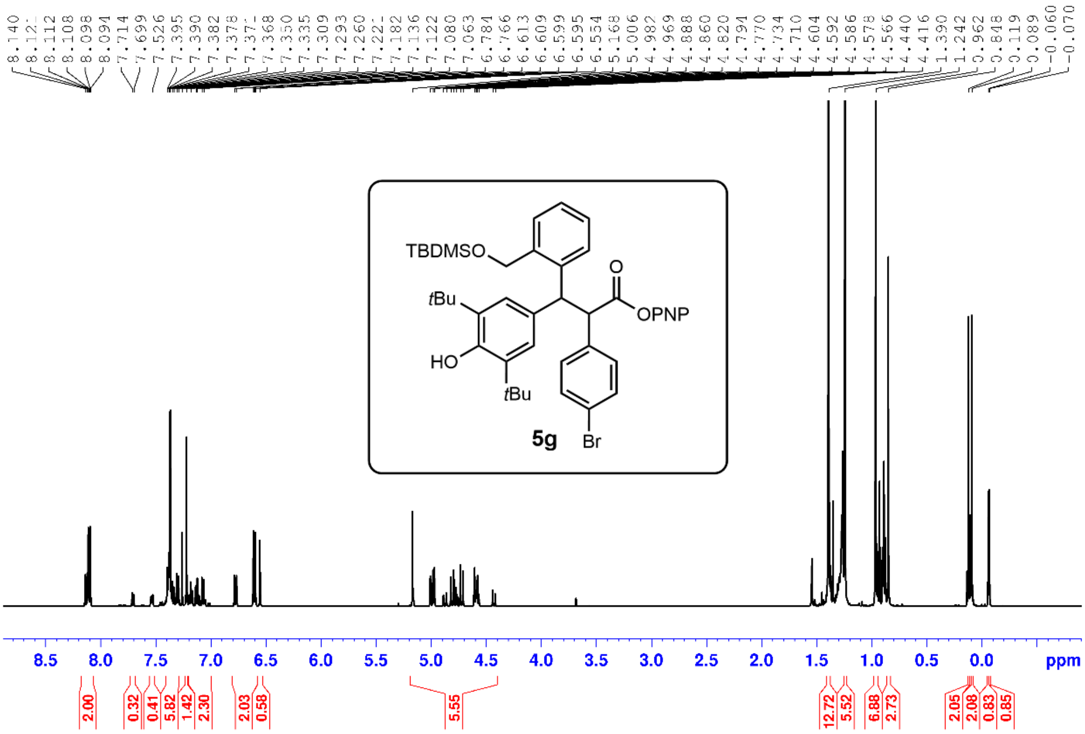

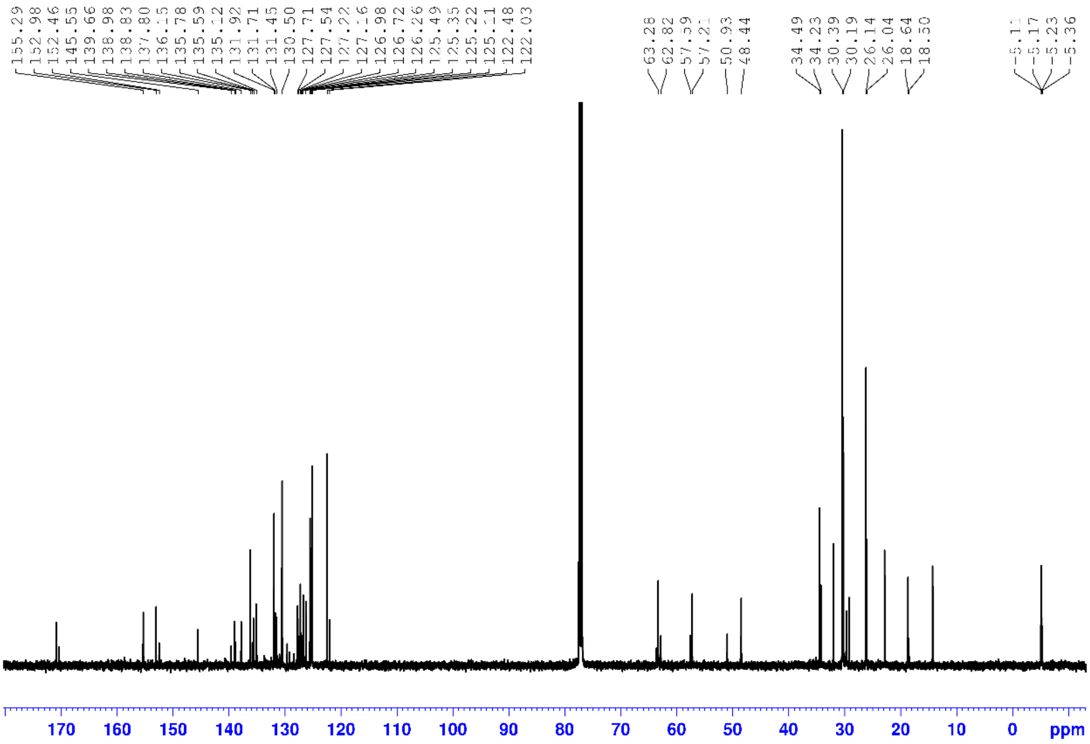

*Cyclic cis product **6g**^{cis}*

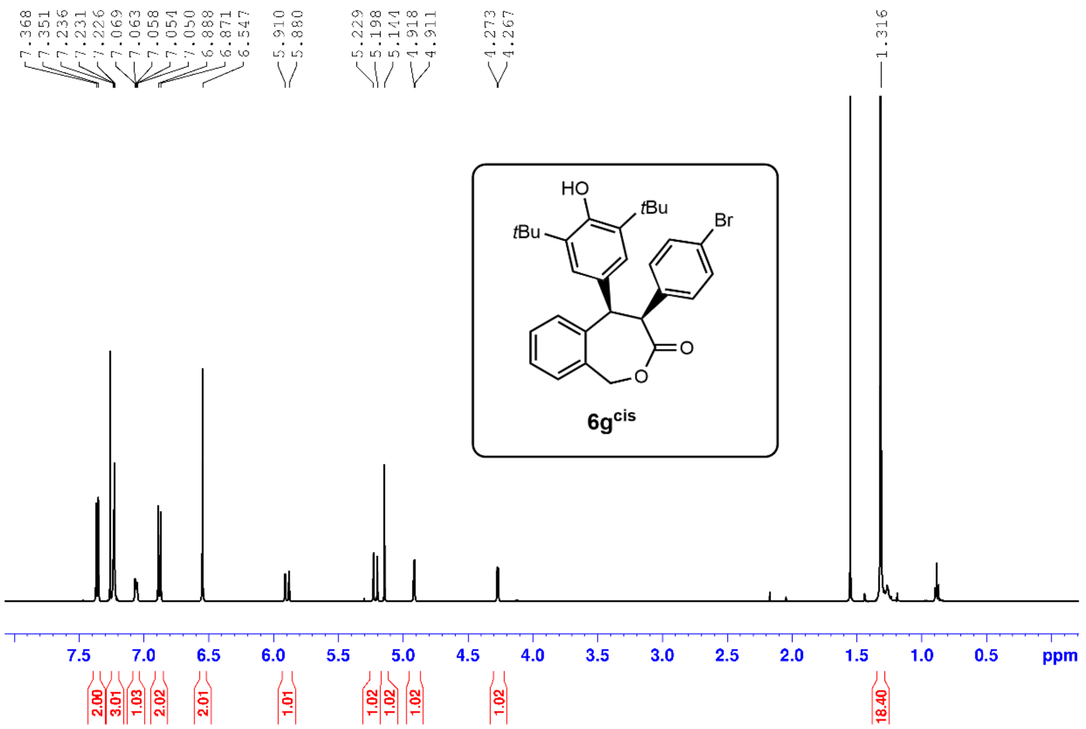

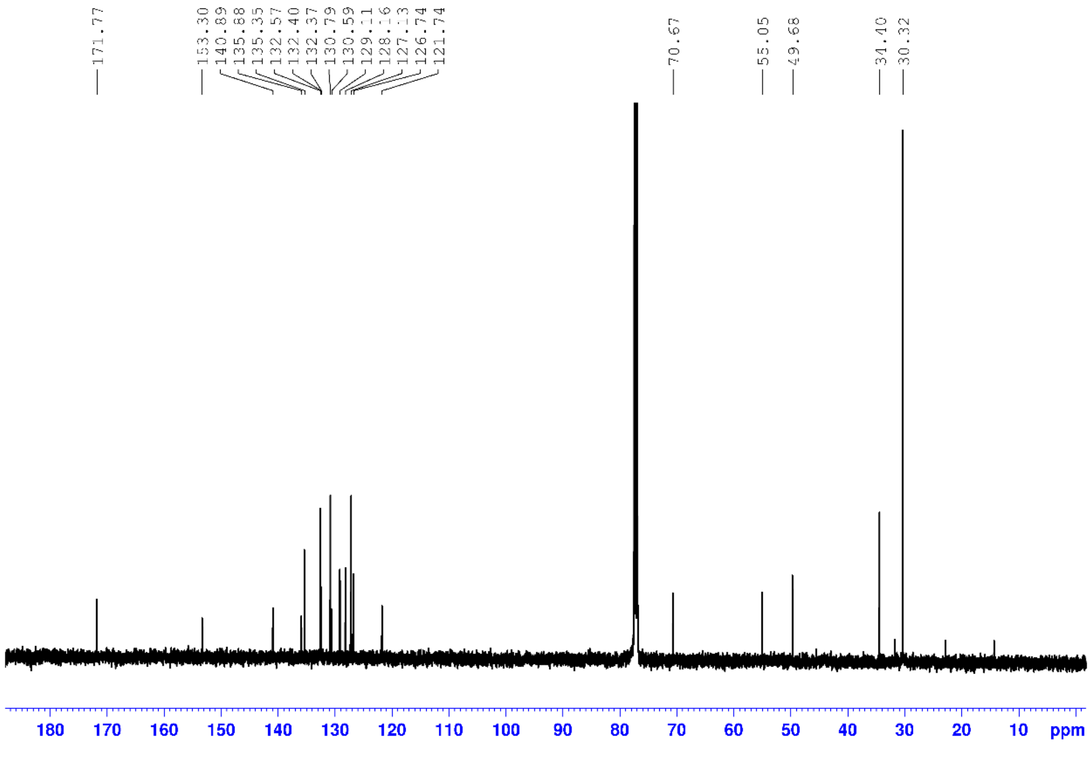

Cyclic trans product ***6g^{trans}***

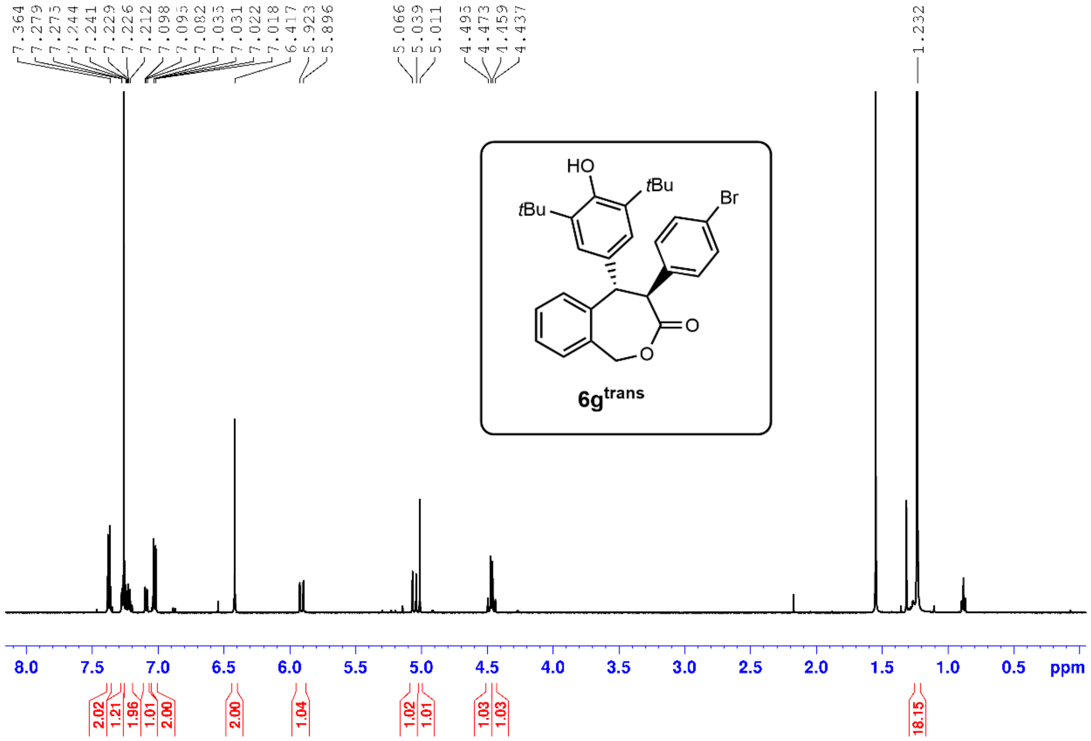

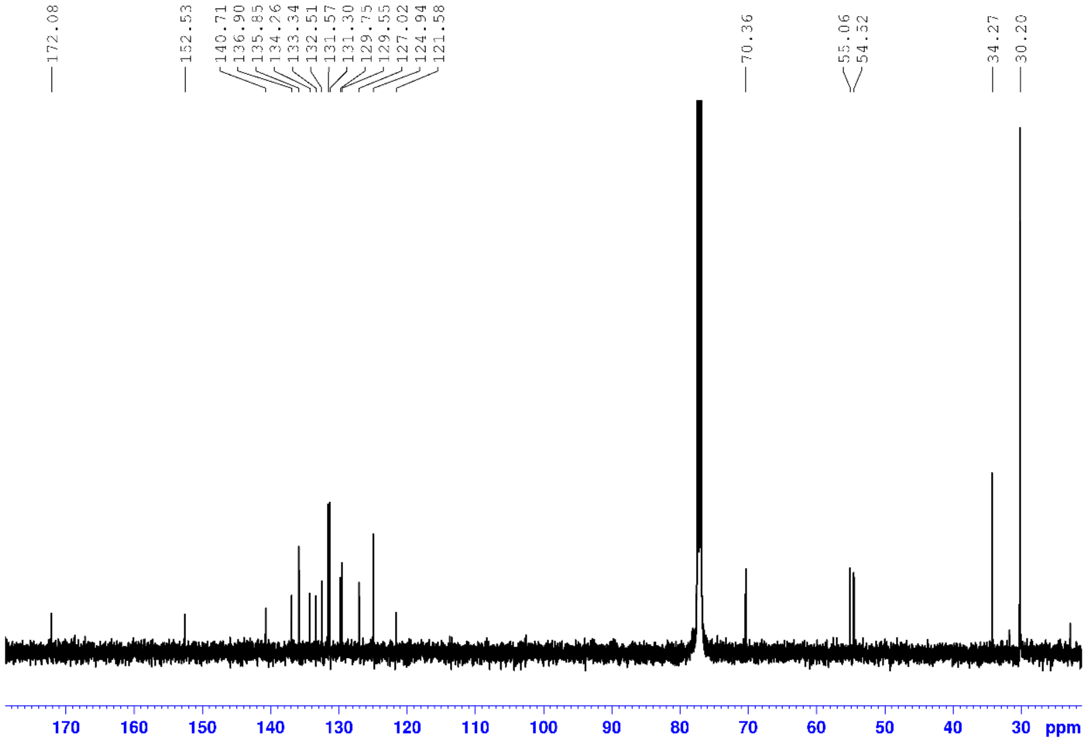

3-**Iodophenyl benzo[c]oxepinone derivative (6h)**

Alkylation product ***5h***

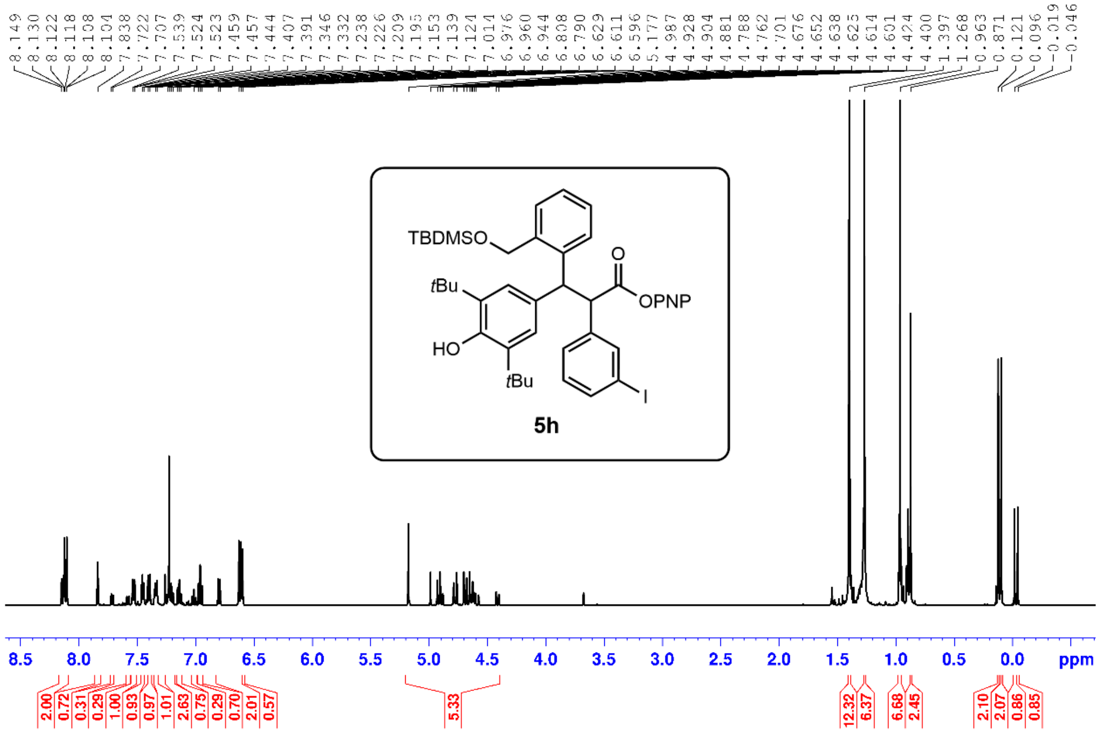

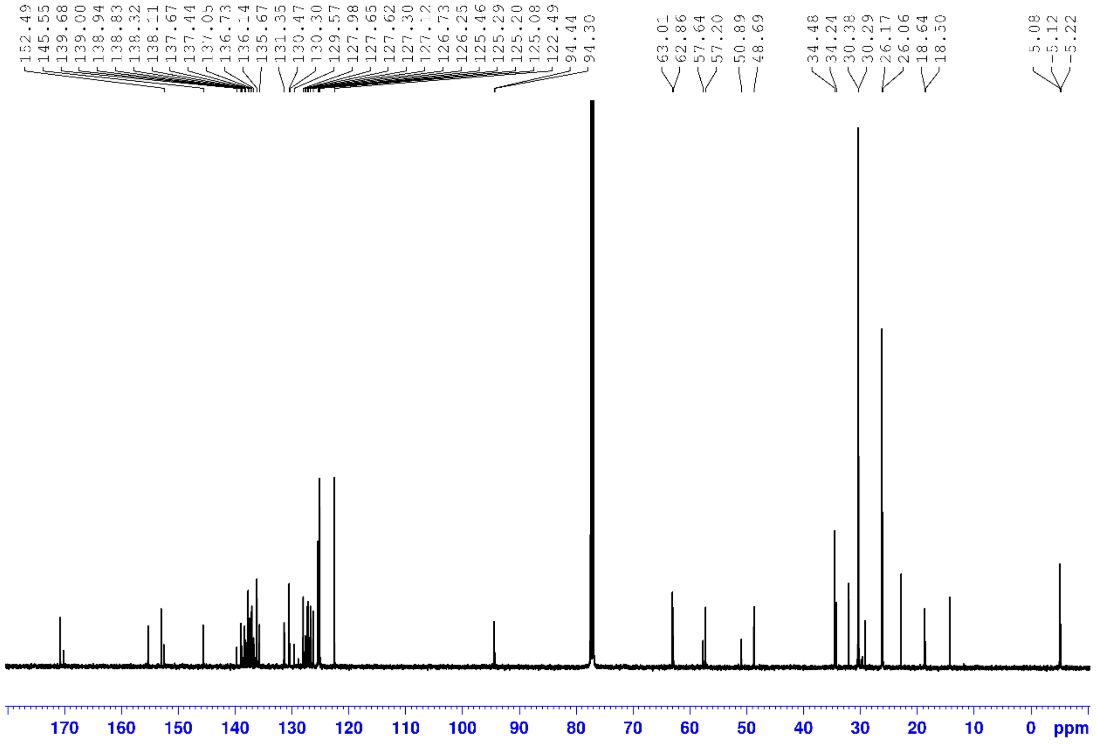

Cyclic cis product ***6h***^{cis}

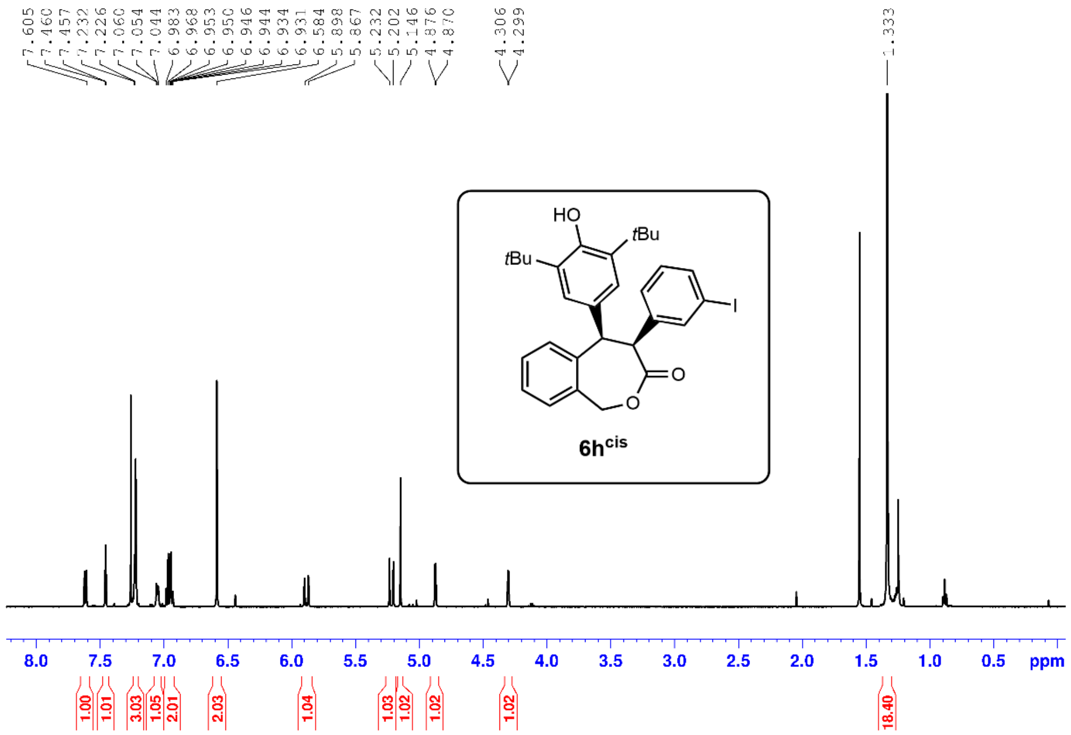

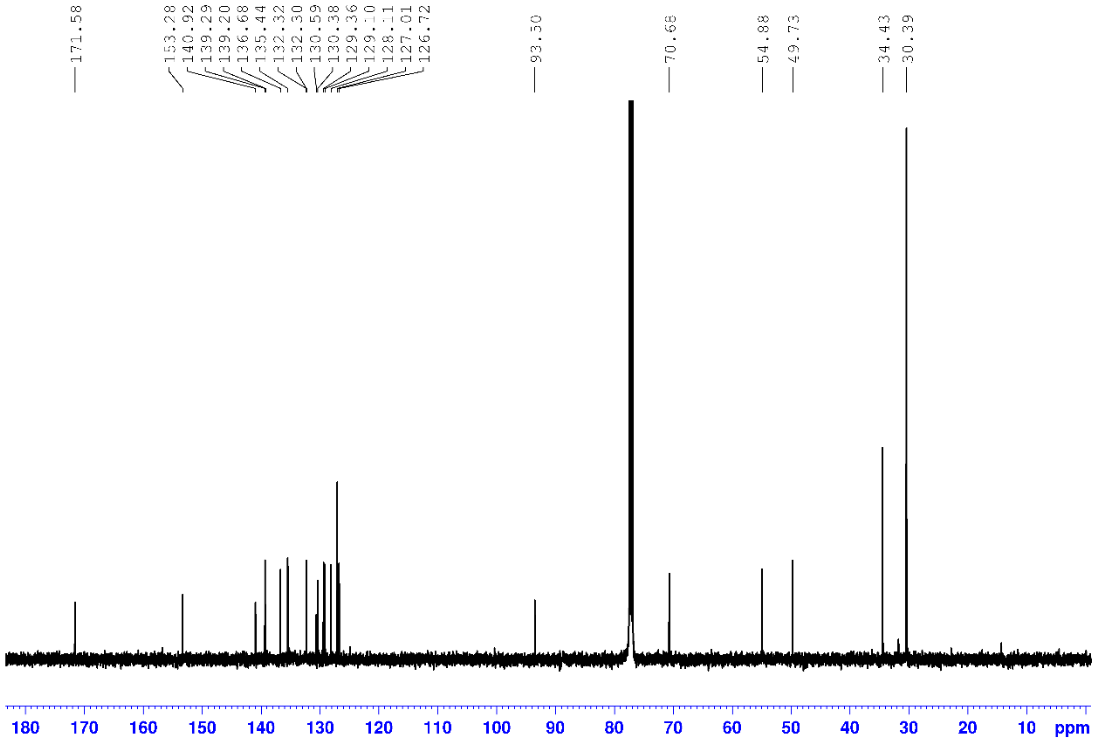

*Cyclic trans product **6h**^{trans}*

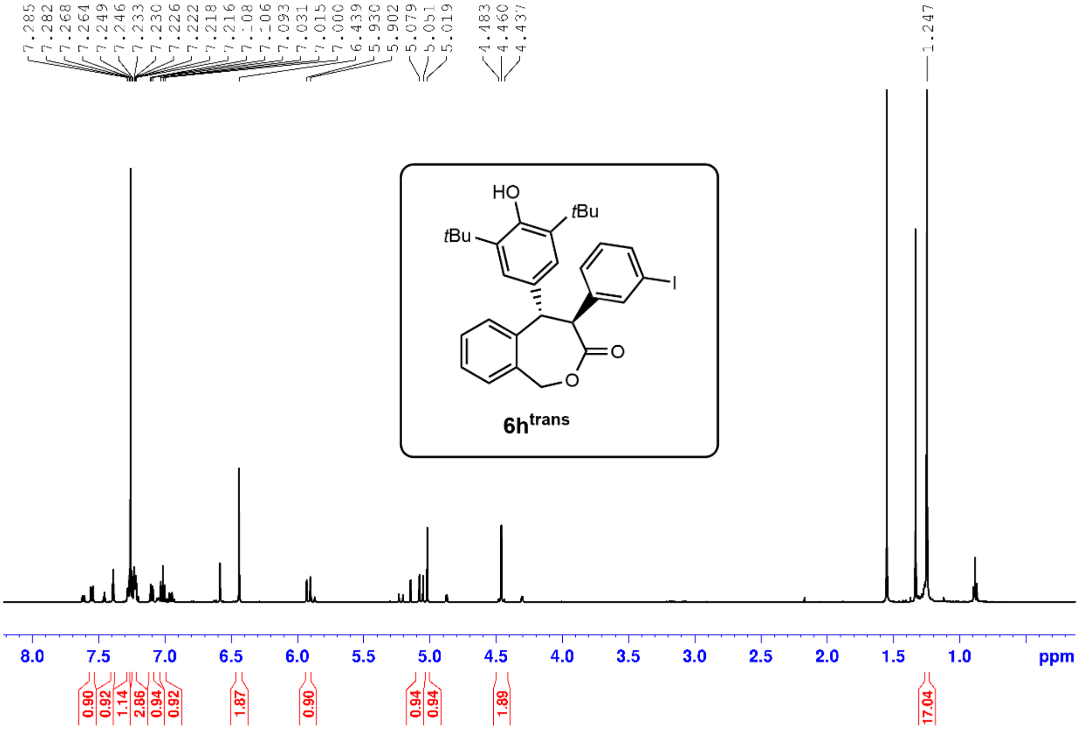

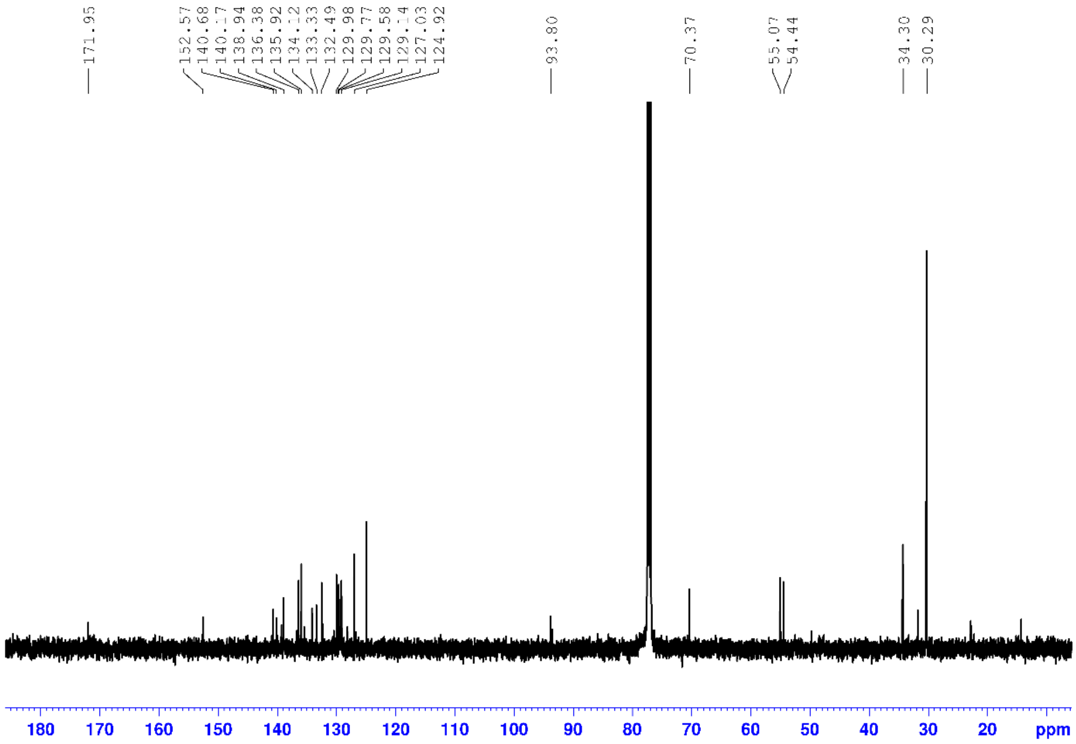

4-Methoxyphenyl benzo[c]oxepinone derivative (6i)

*Alkylation Product **5i***

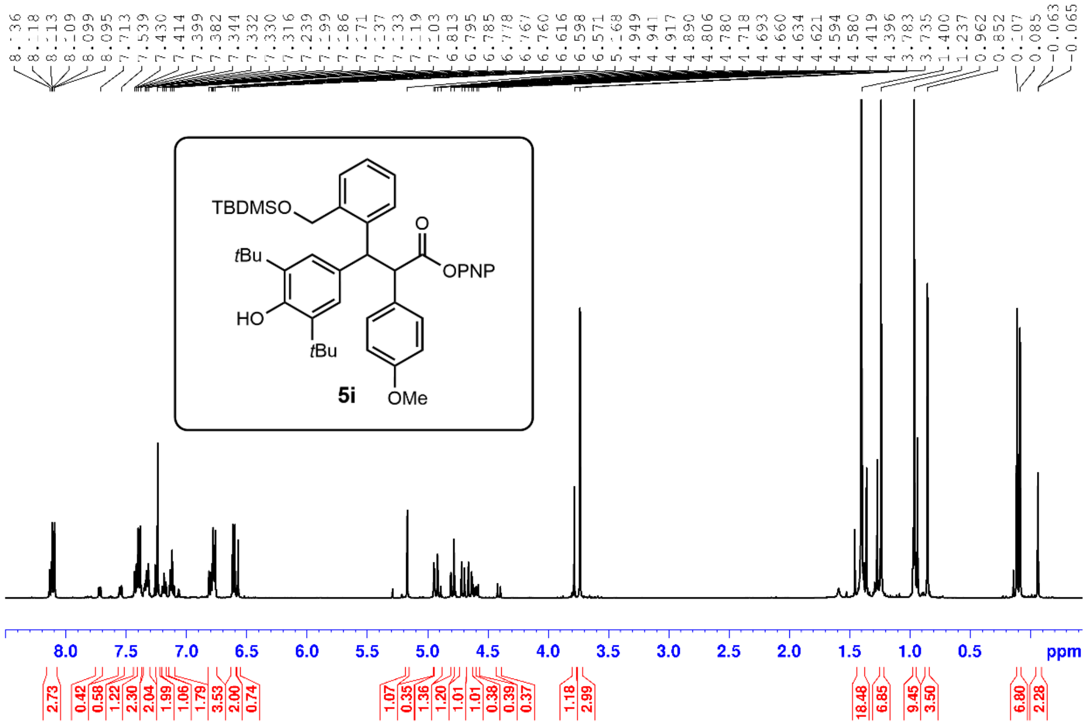

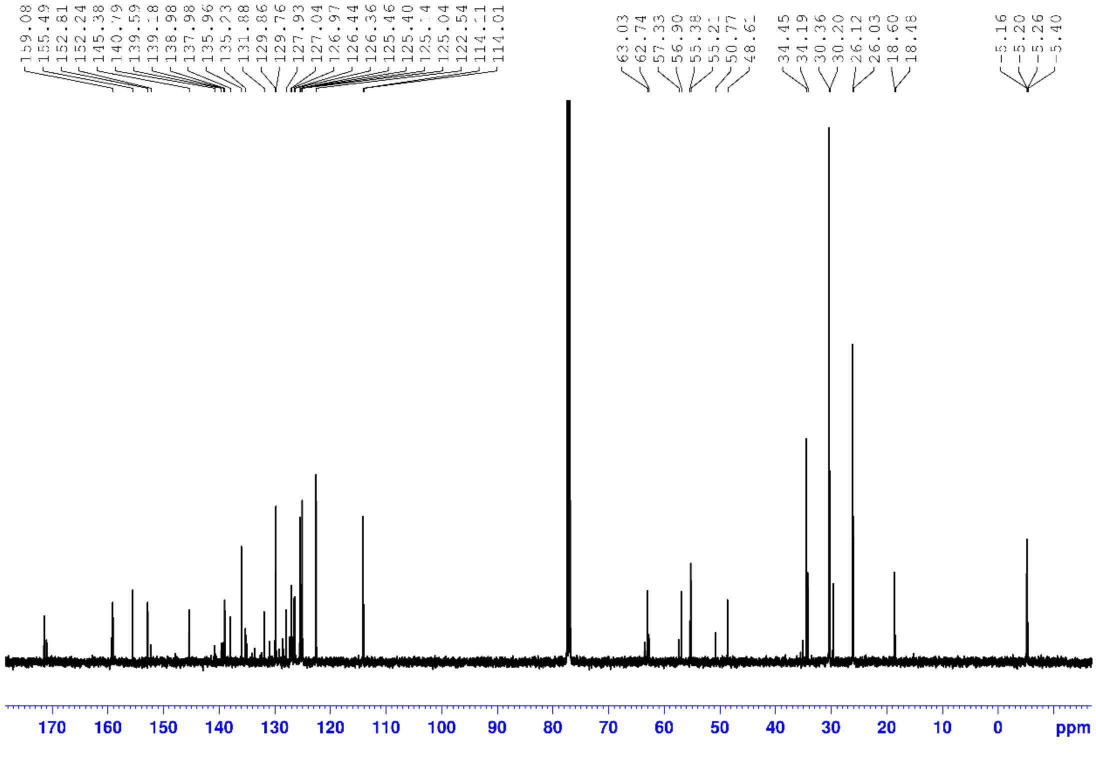

*Cyclic cis product **6i**cis*

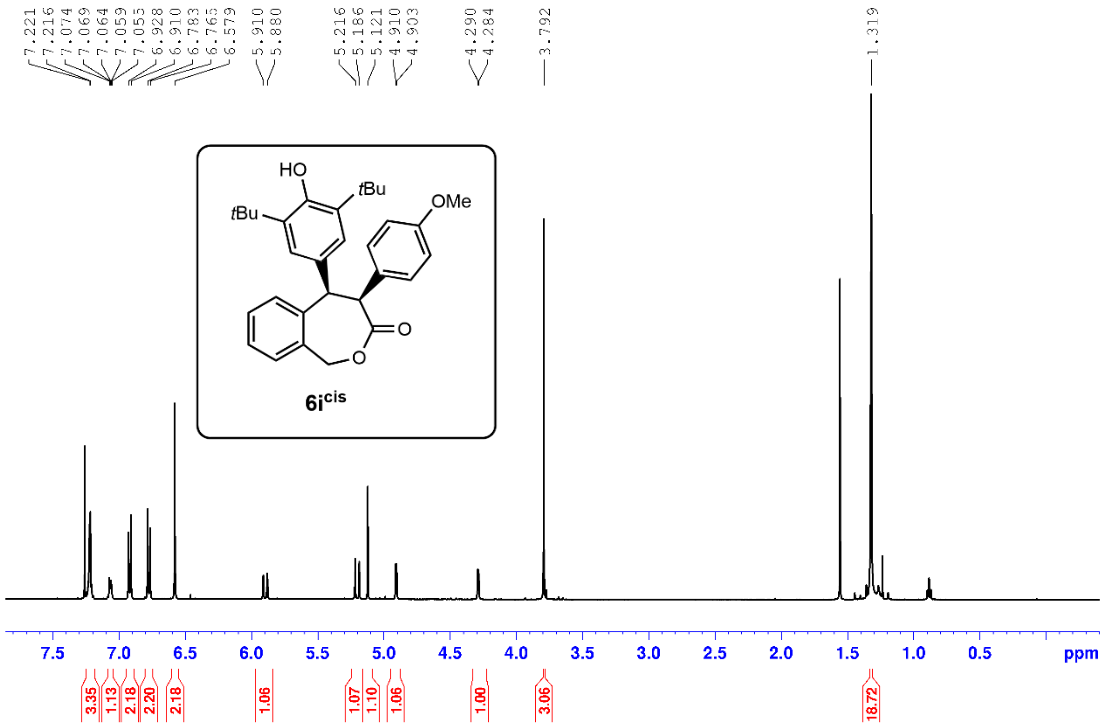

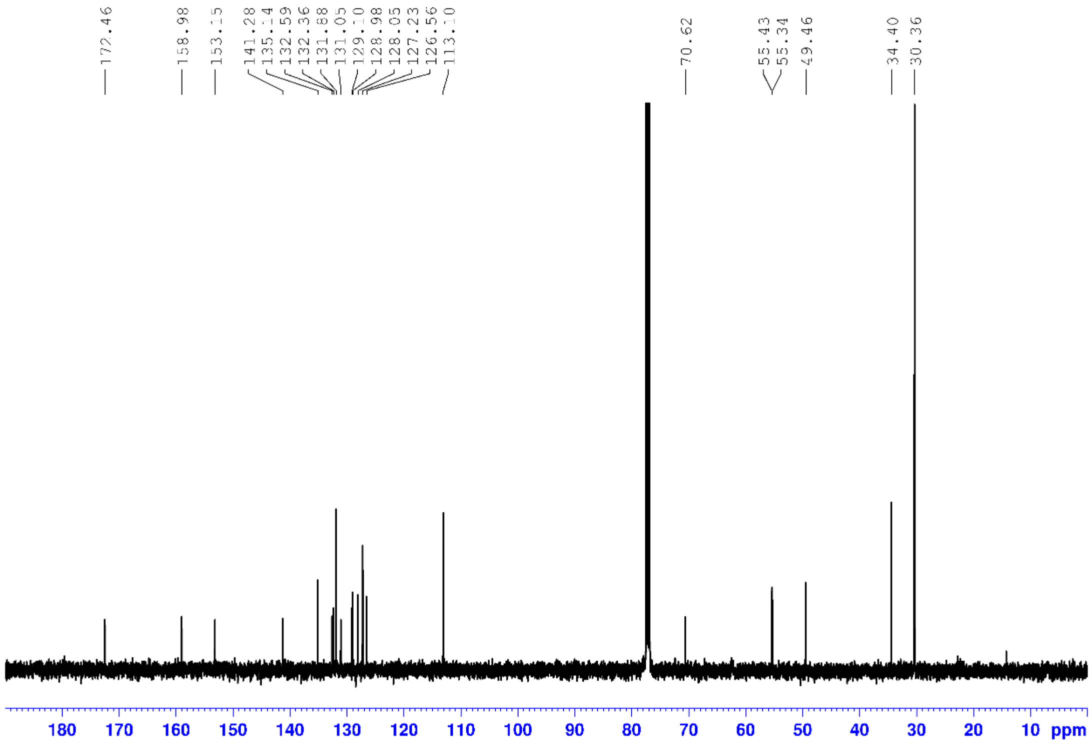

*Cyclic trans product **6i**^{trans}*

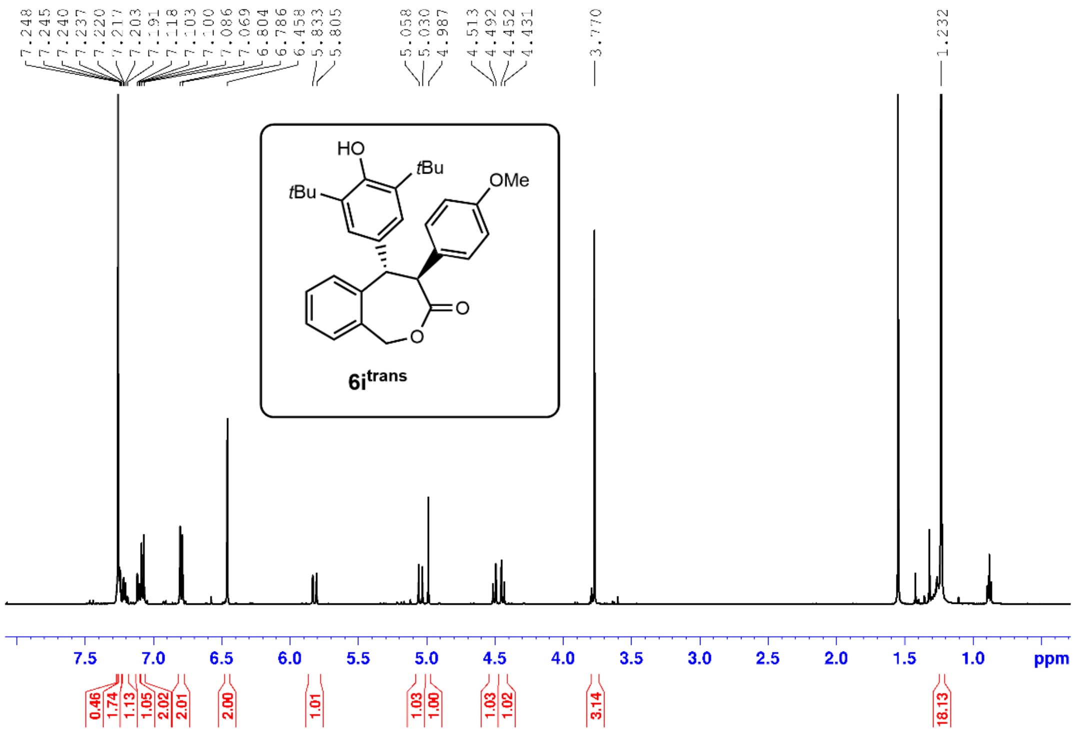

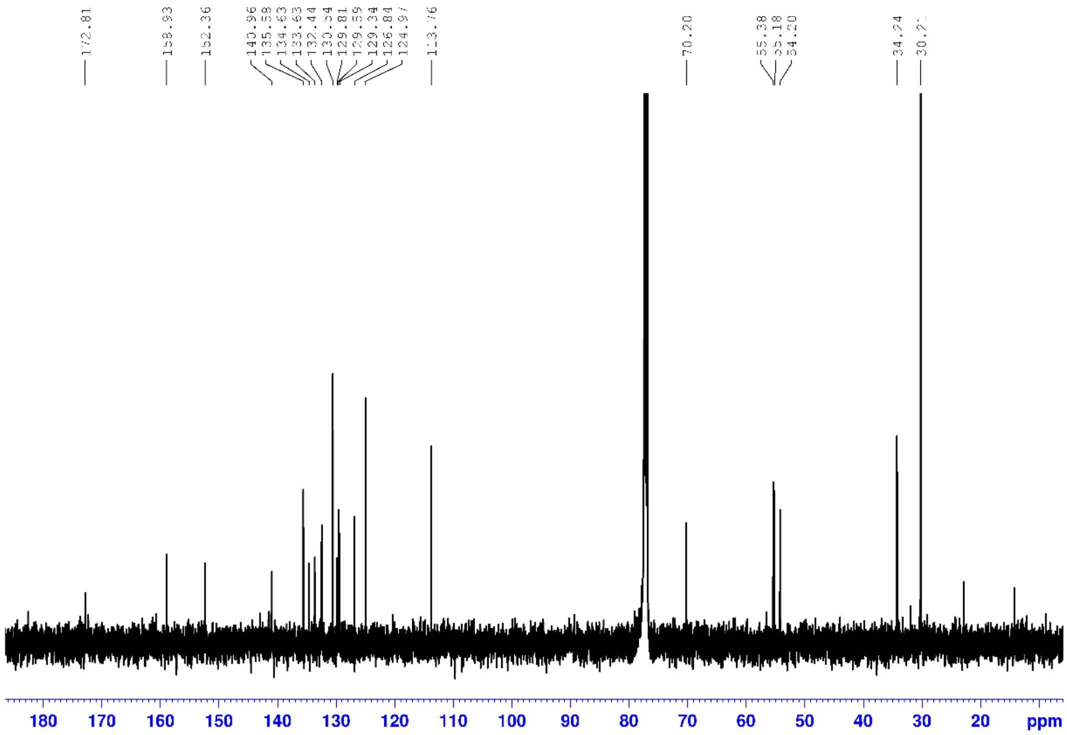

Dimethoxyphenyl benzo[c]oxepinone derivative (6j)

*Alkylation Product **5j***

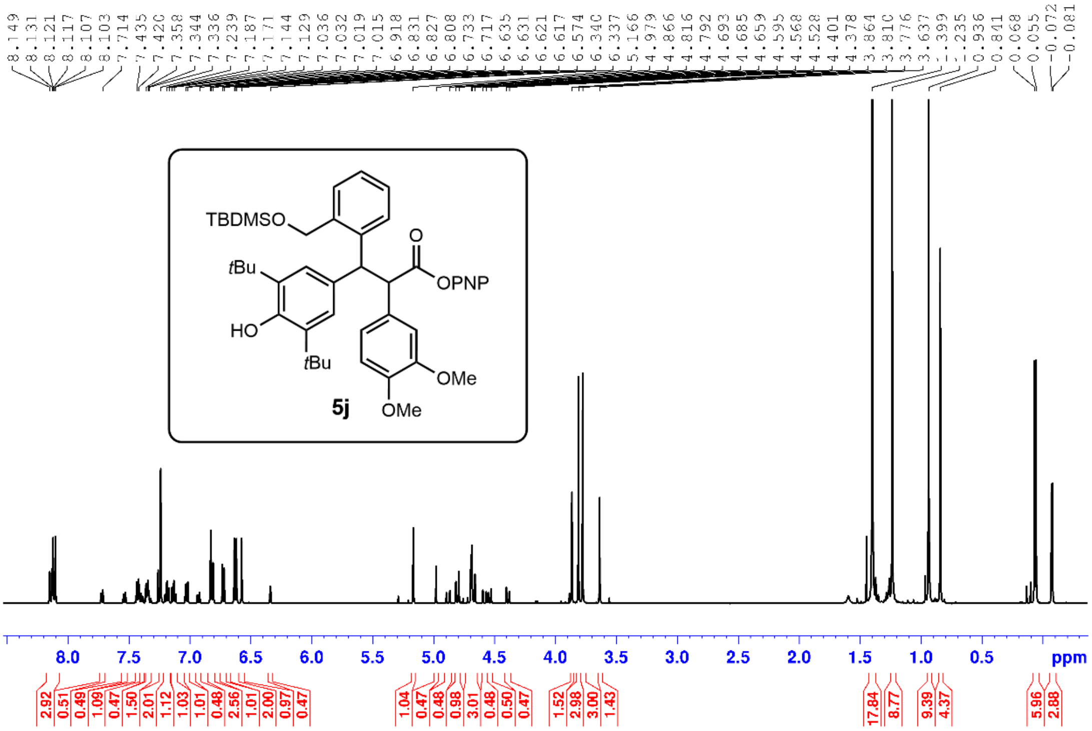

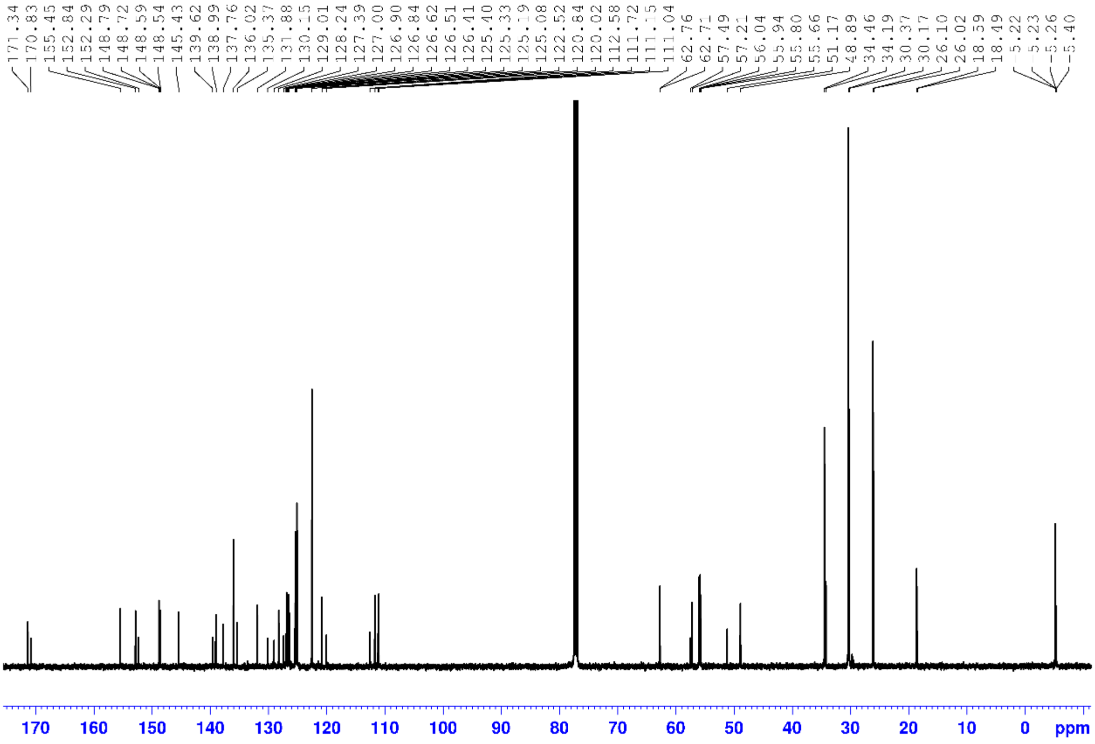

*Cyclic cis product **6j**cis*

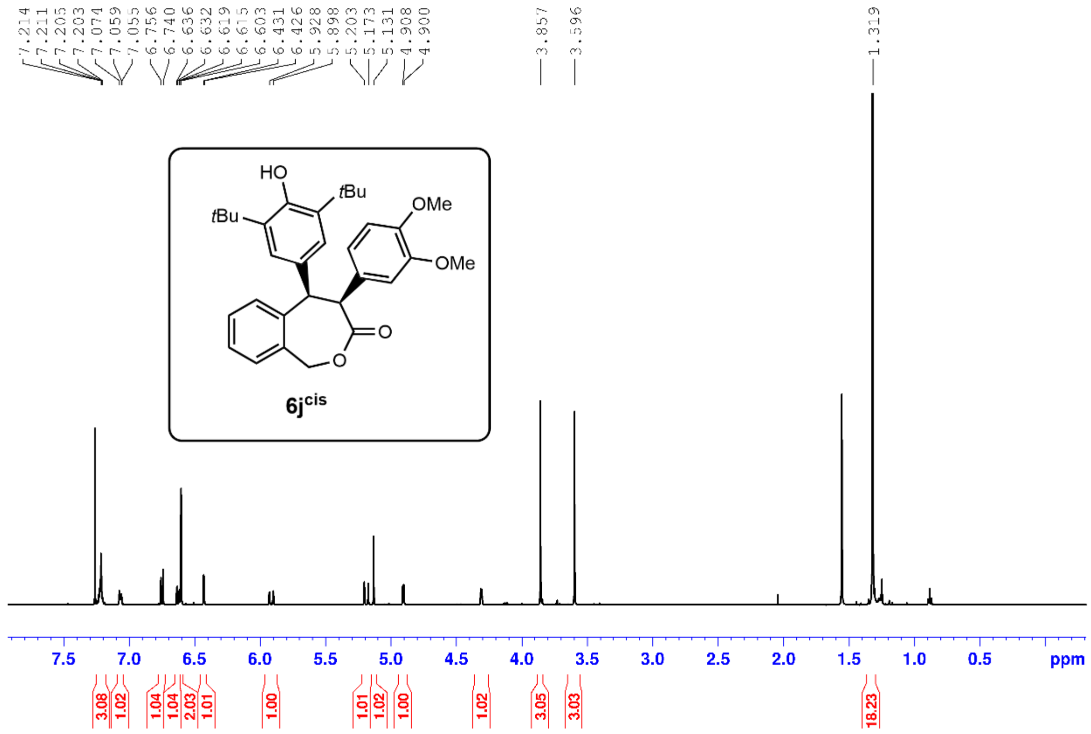

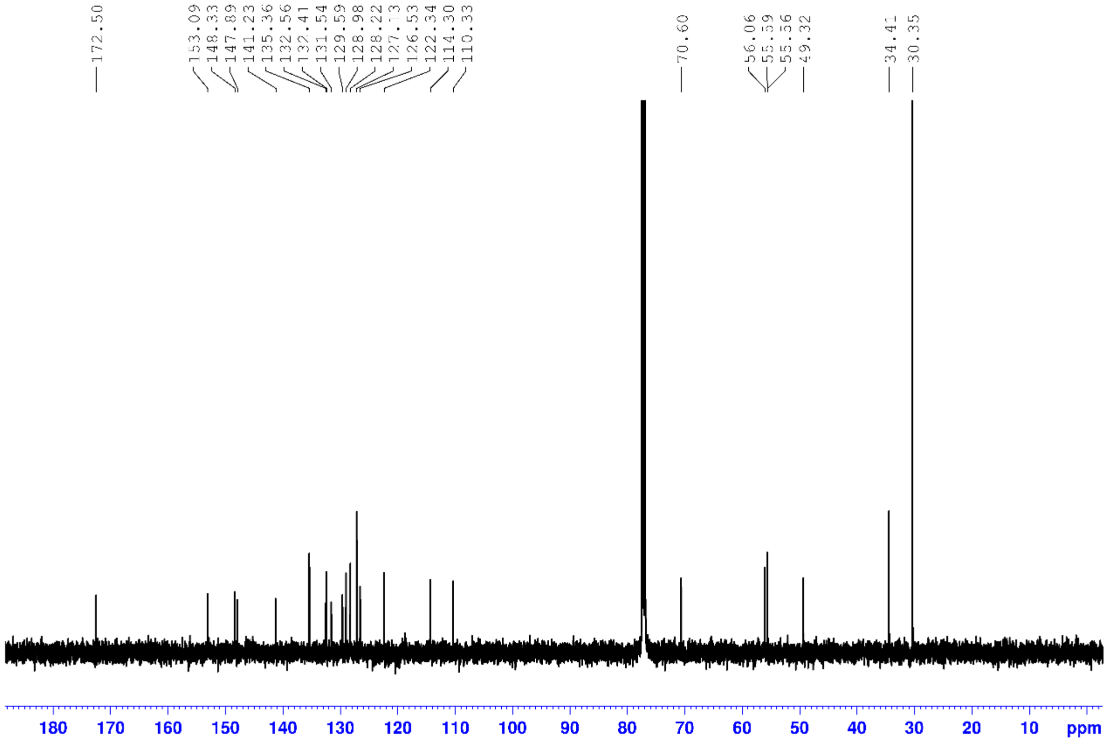

Cyclic trans product ***6j***$^{\textbf{\textit{trans}}}$

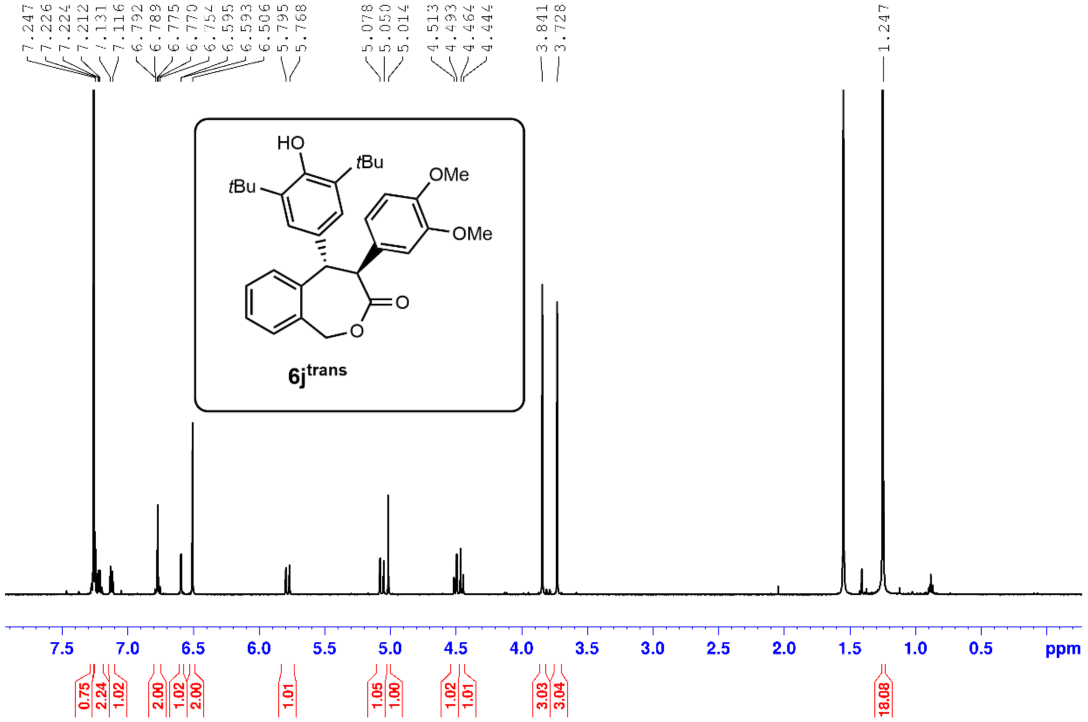

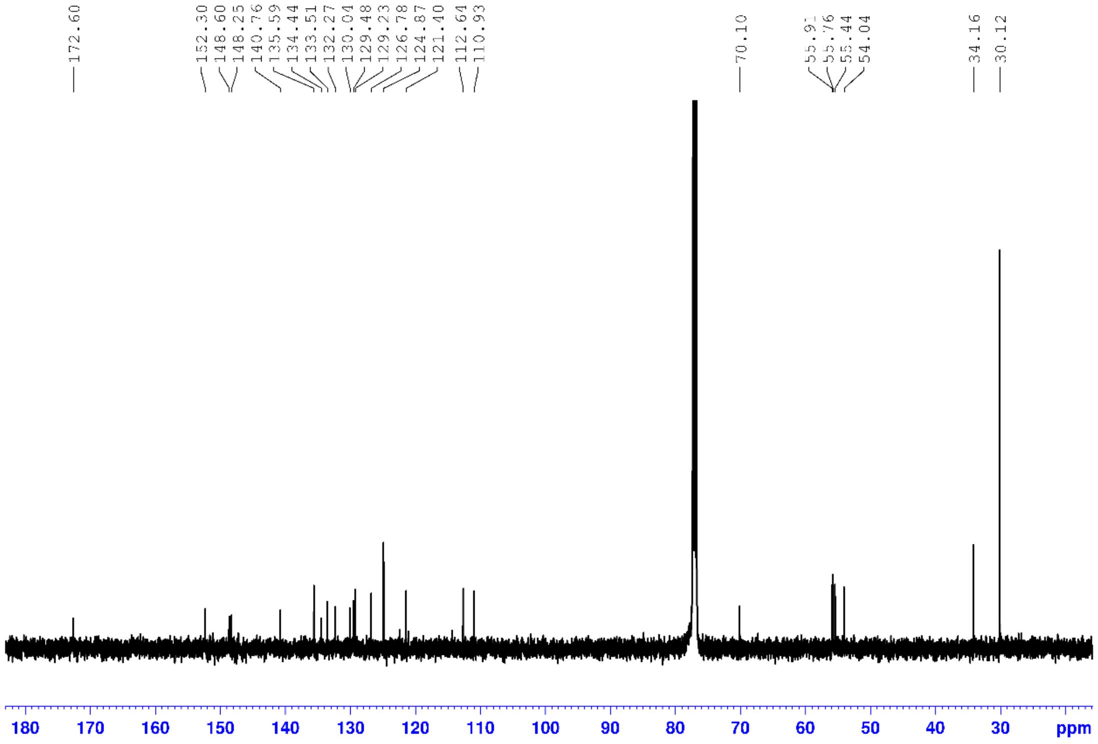

4-Methylthiophenyl benzo[c]oxepinone derivative (6k)

*Alkylation Product **5k***

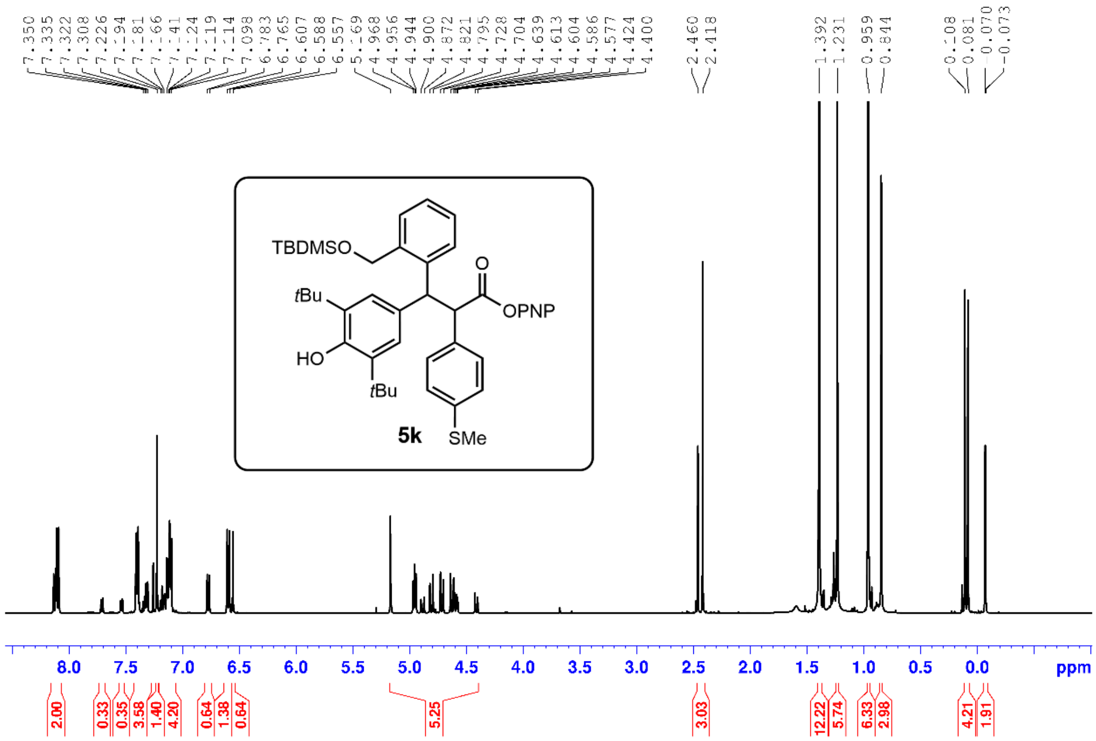

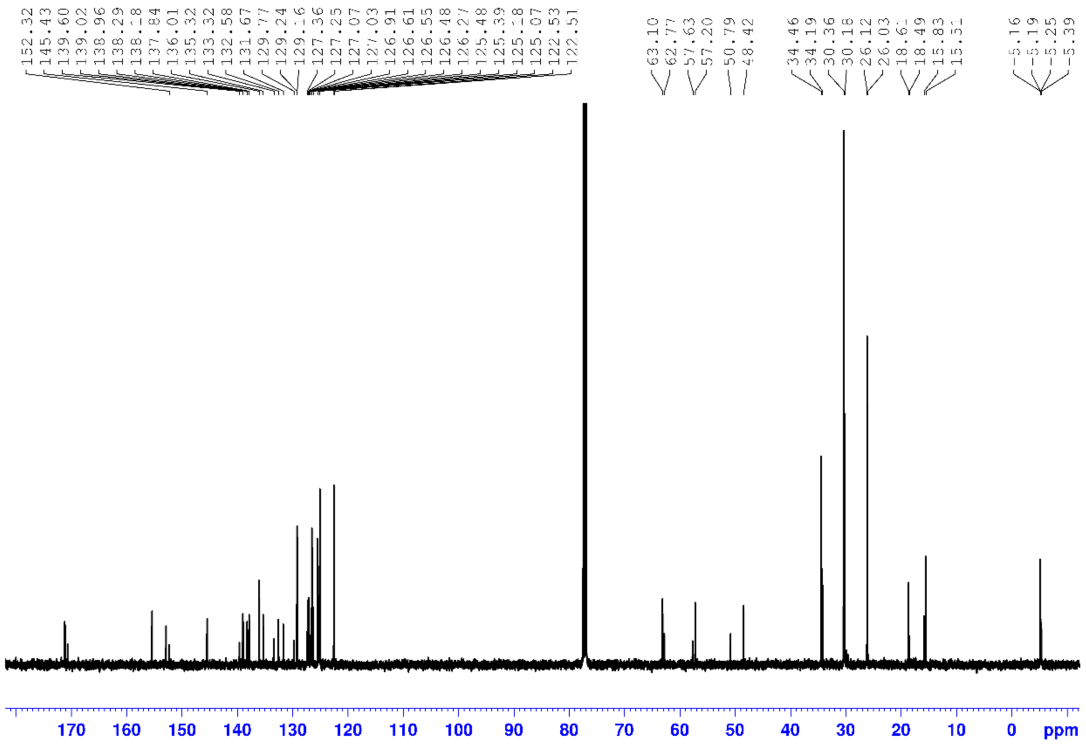

Cyclic cis product ***6k***^{cis}

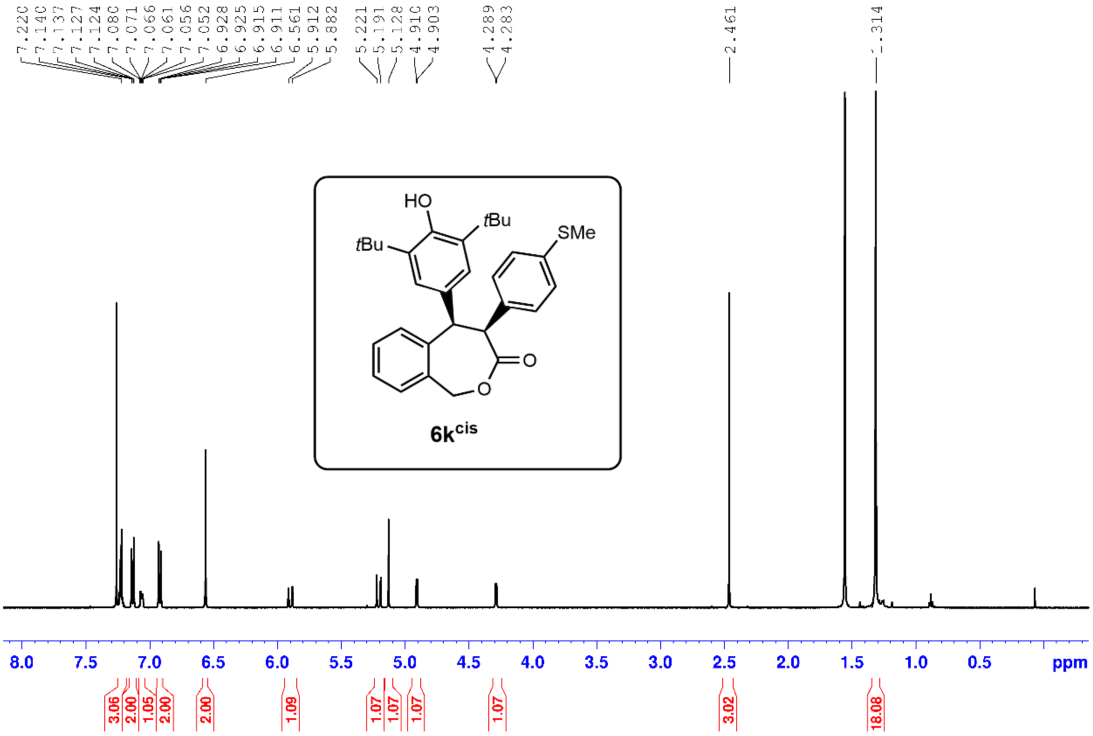

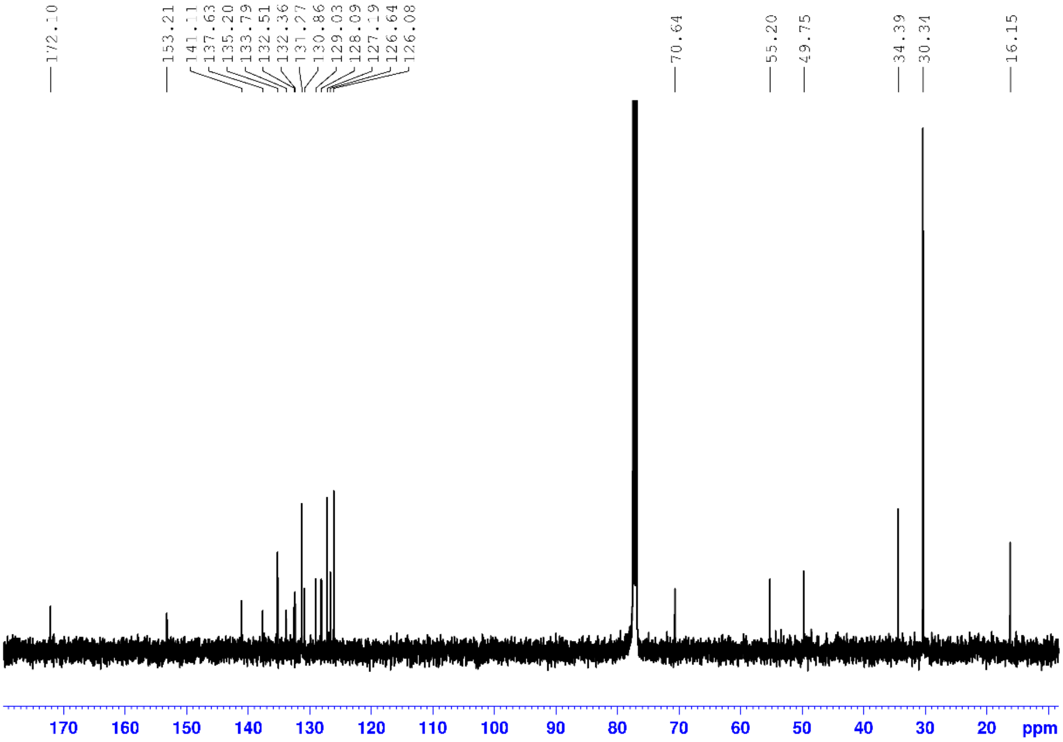

*Cyclic trans product **6k**[trans]*

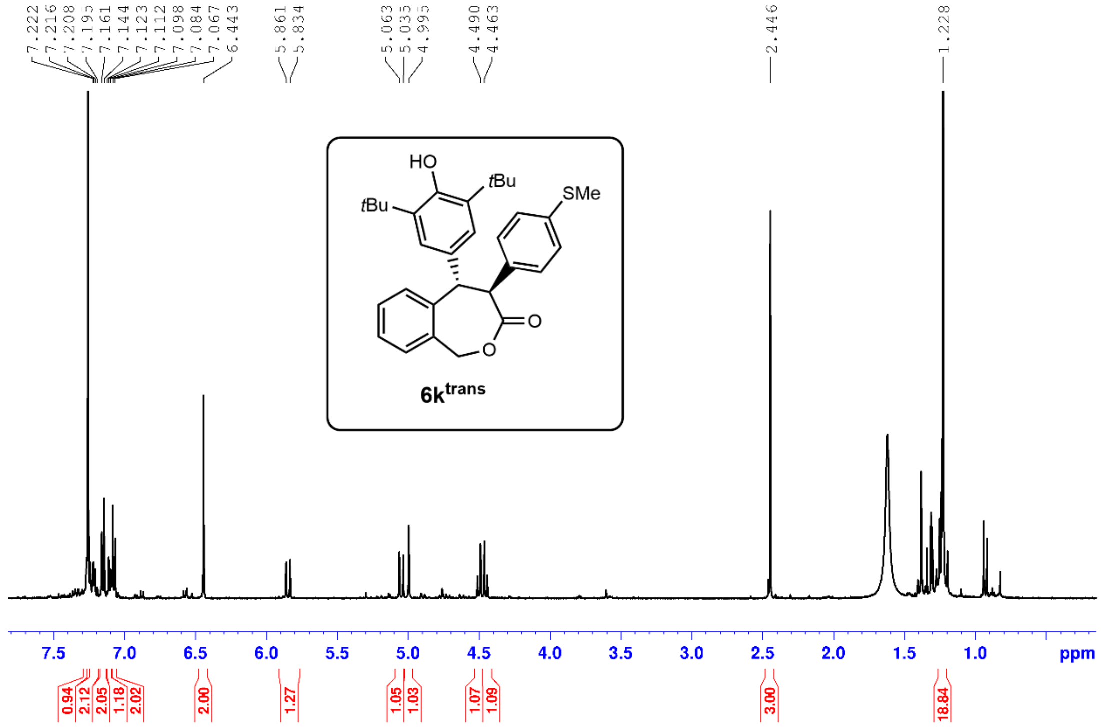

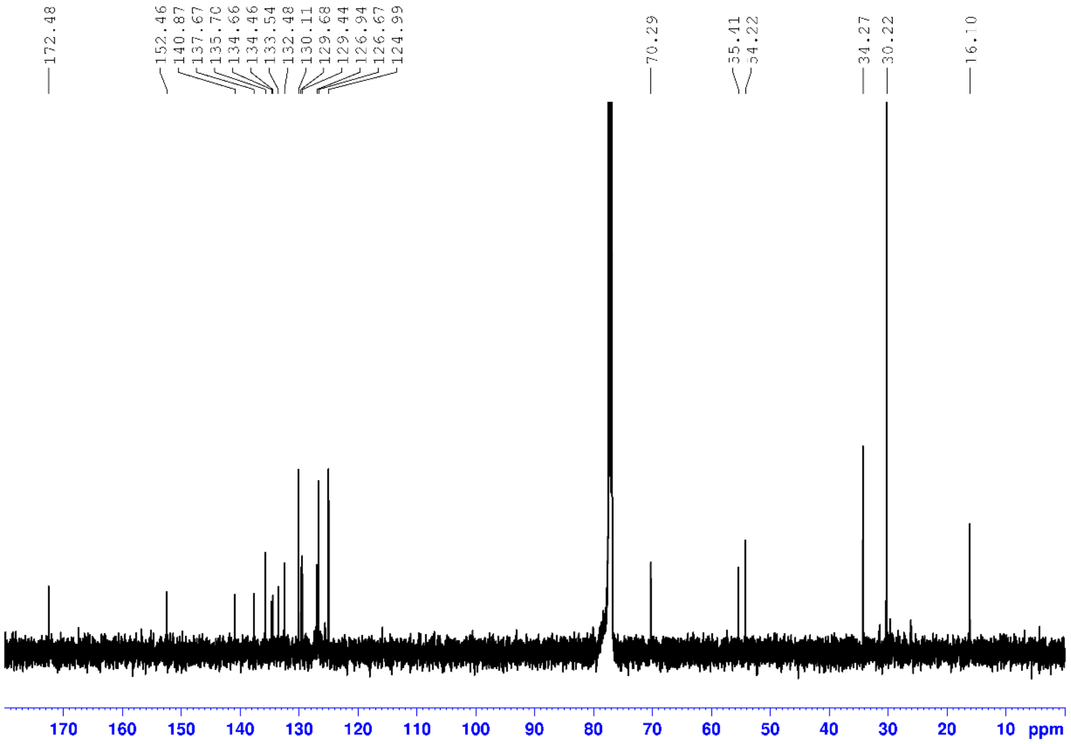

2-Methylphenyl benzo[c]oxepinone derivative (6l)

*Alkylation Product **5l***

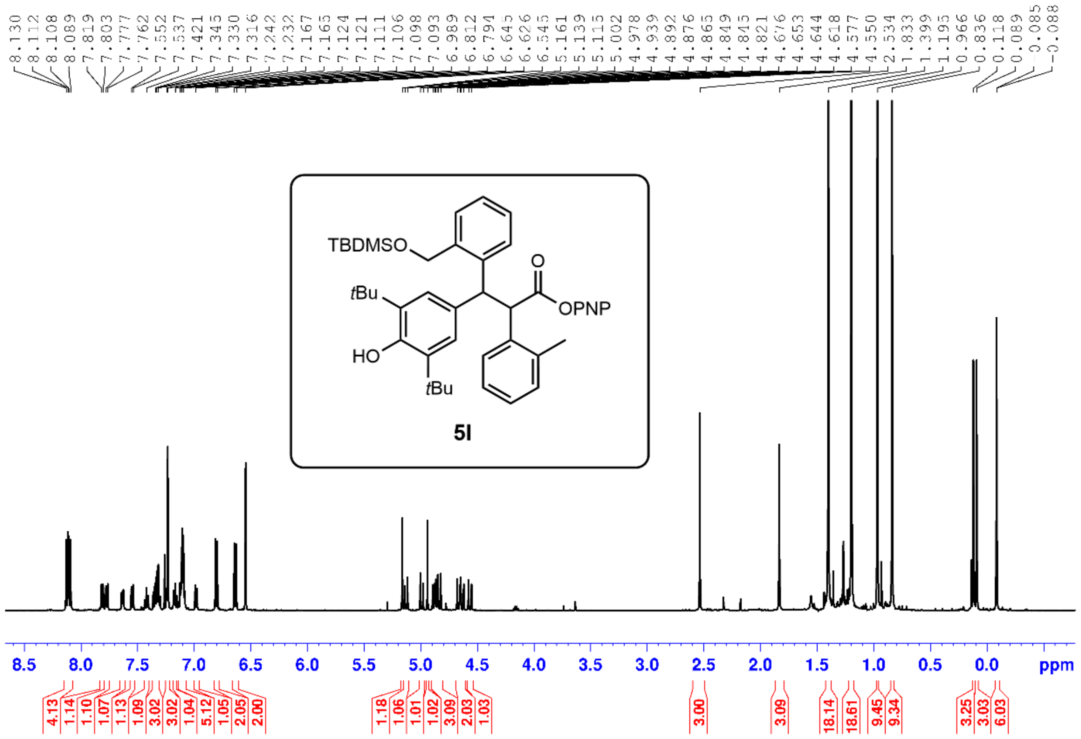

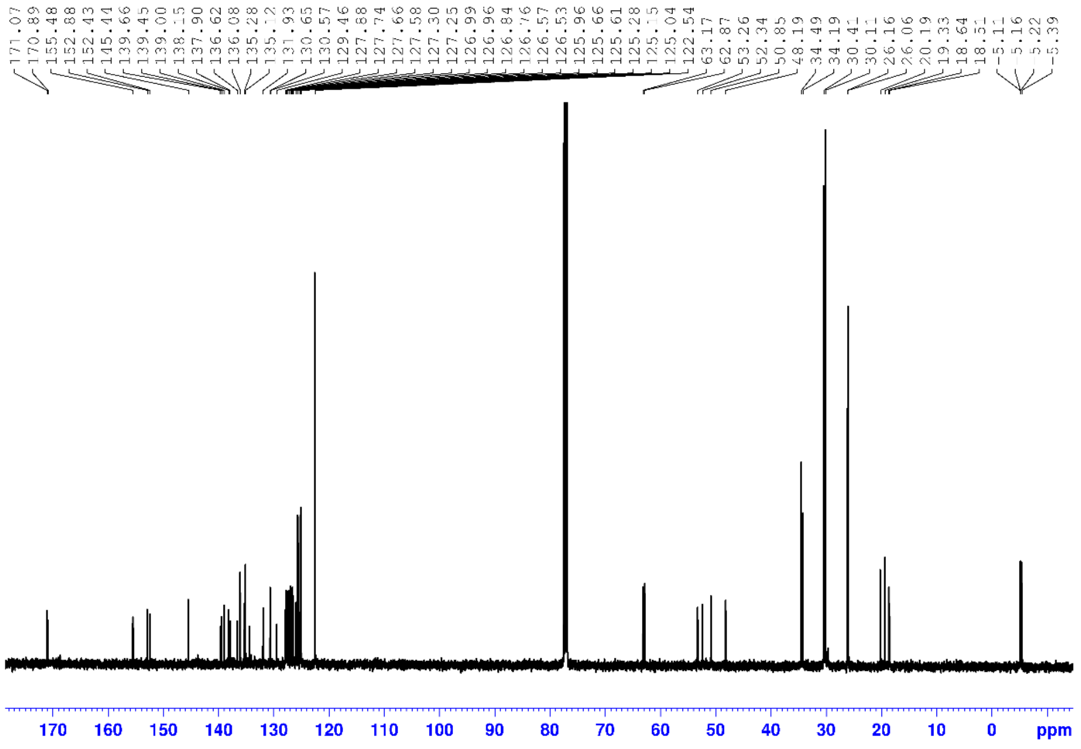

*Cyclic cis product **6l**^{cis}*

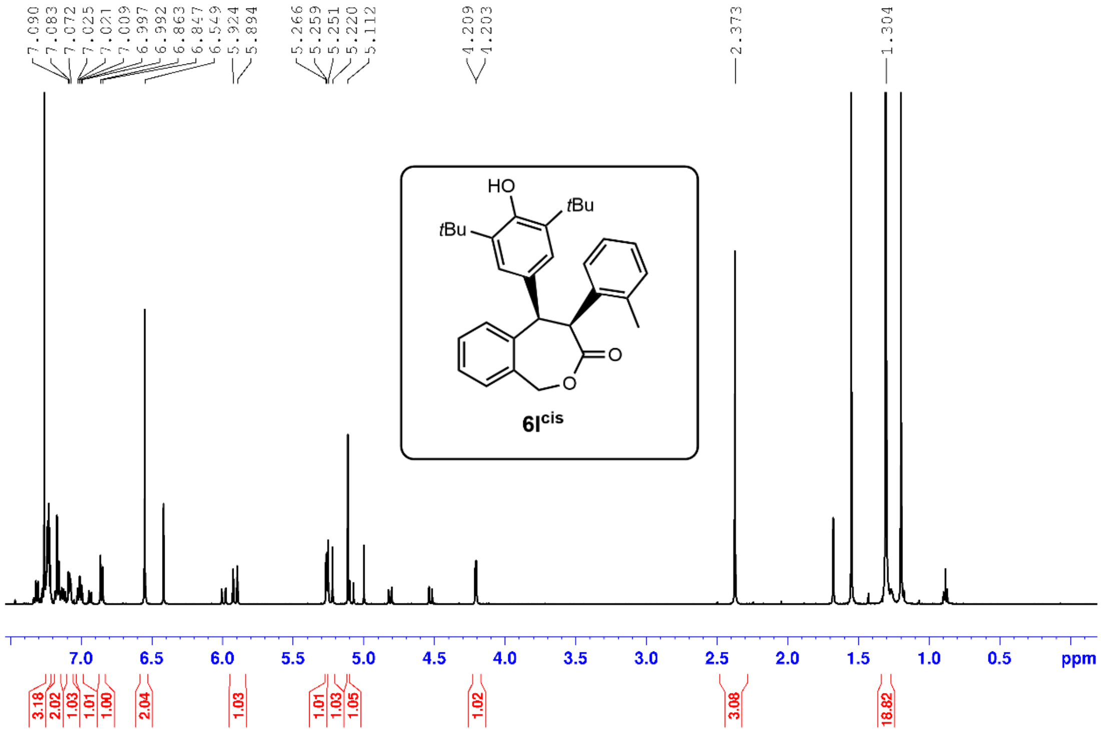

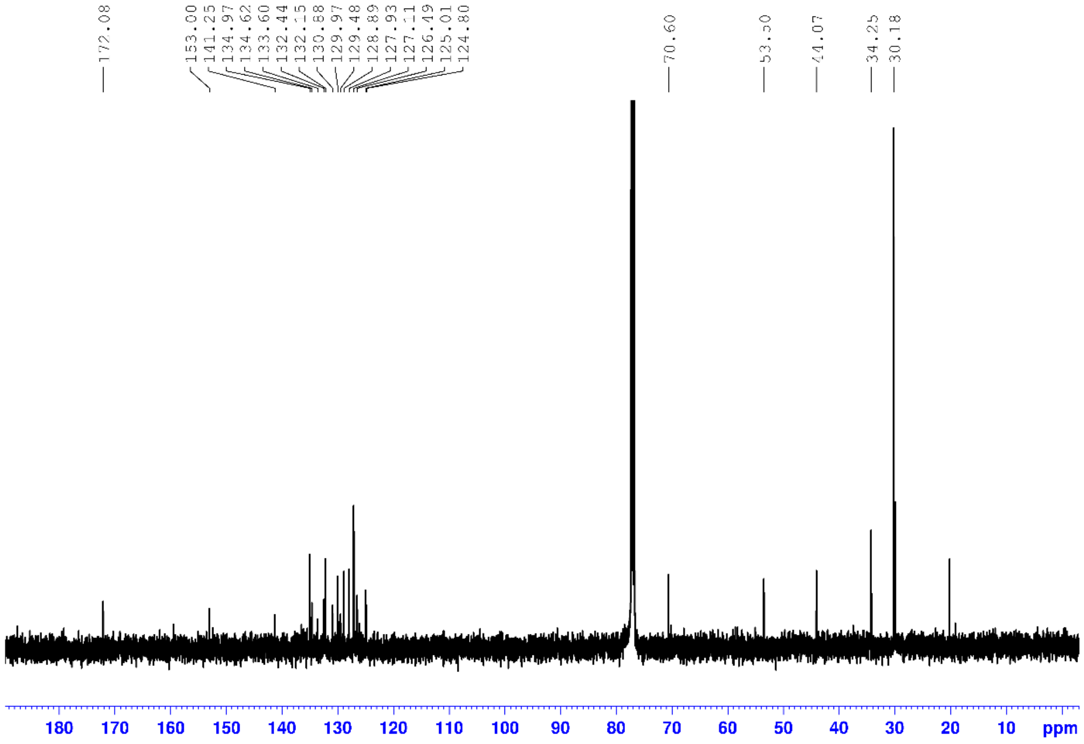

*Cyclic trans product **6k**[trans]*

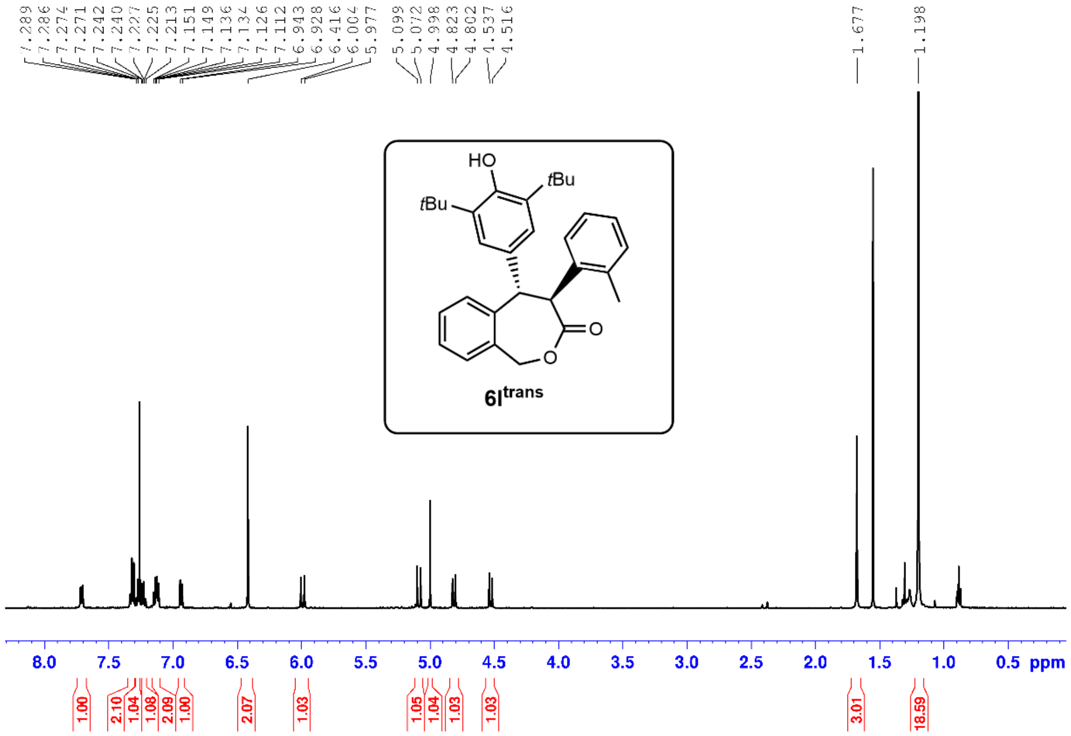

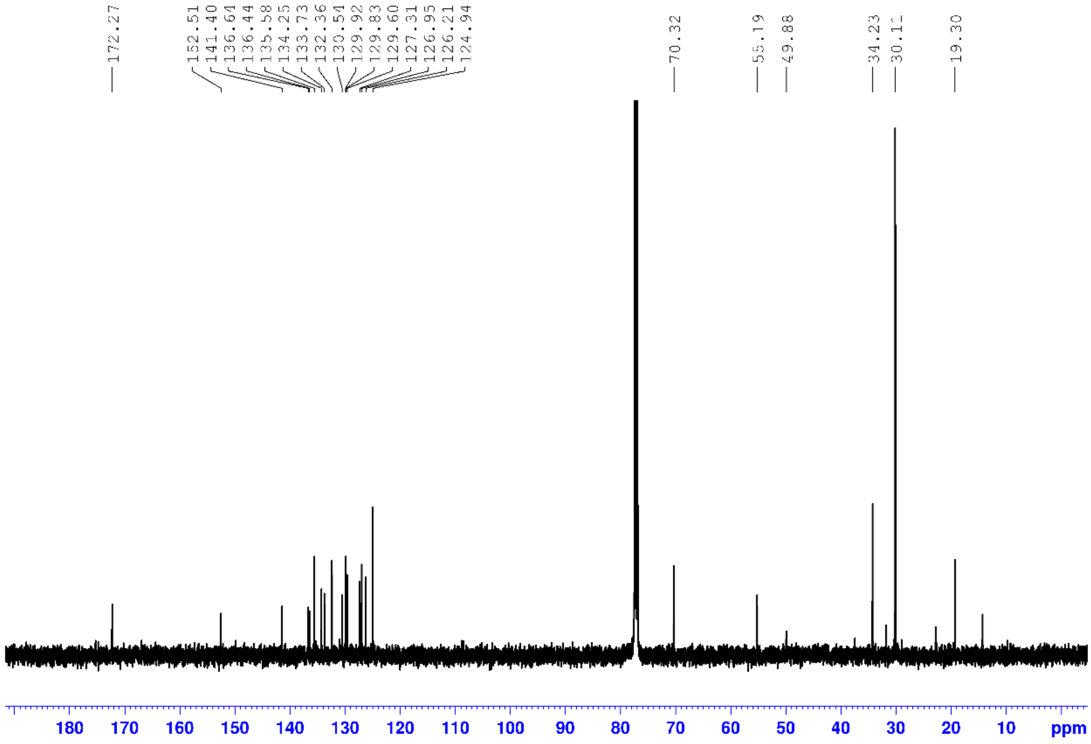

3-Methylphenyl benzo[c]oxepinone derivative (6m)

*Alkylation Product **5m***

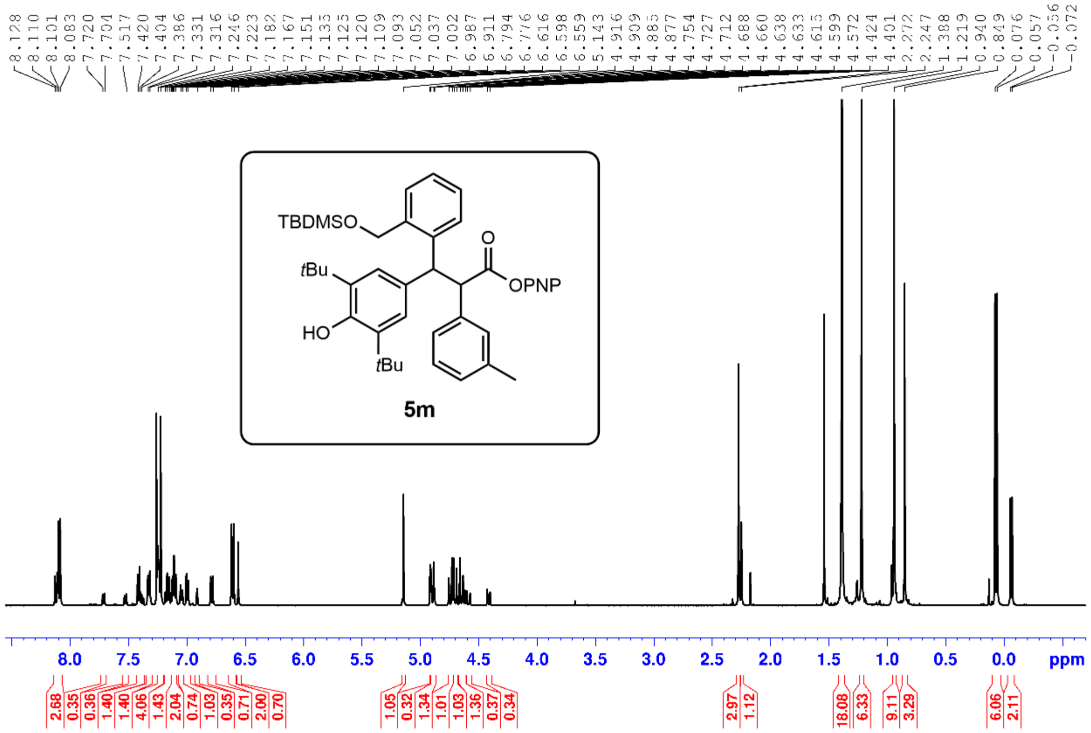

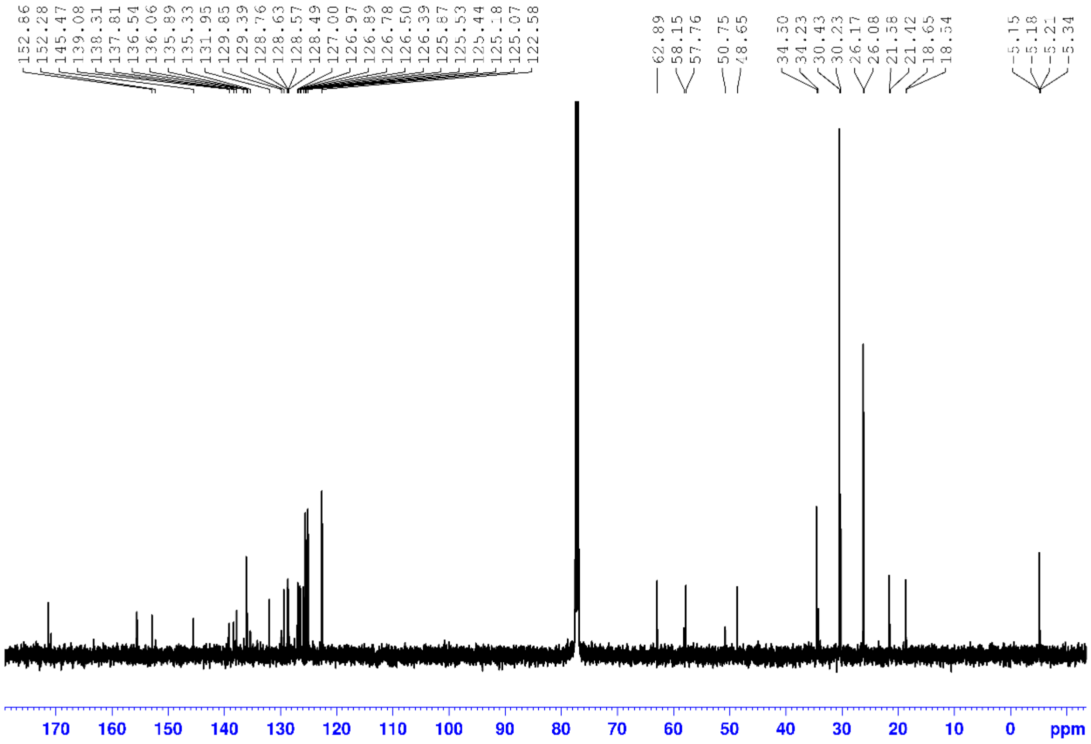

Cyclic cis product ***6m***^{cis}

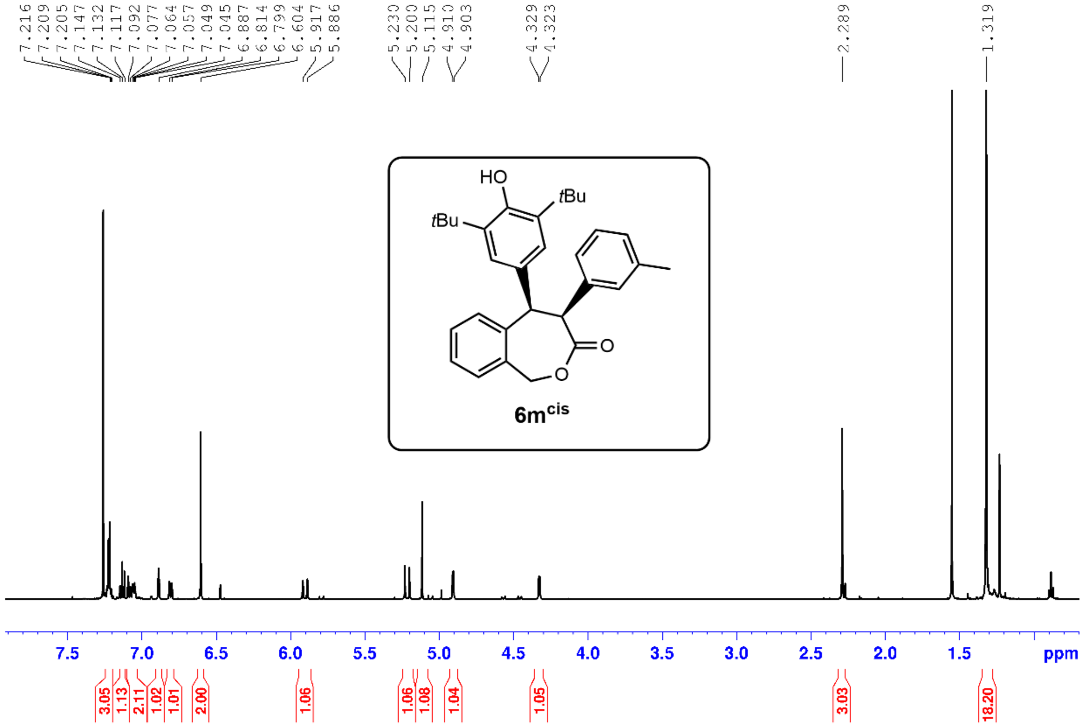

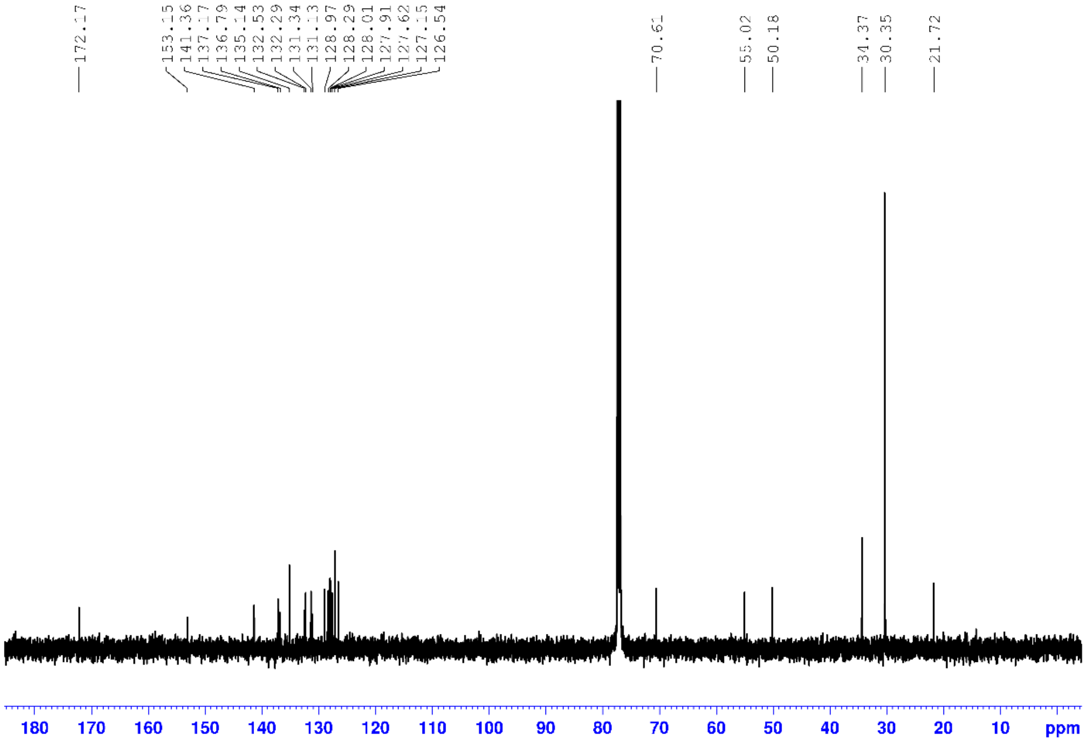

*Cyclic trans product **6m**^{trans}*

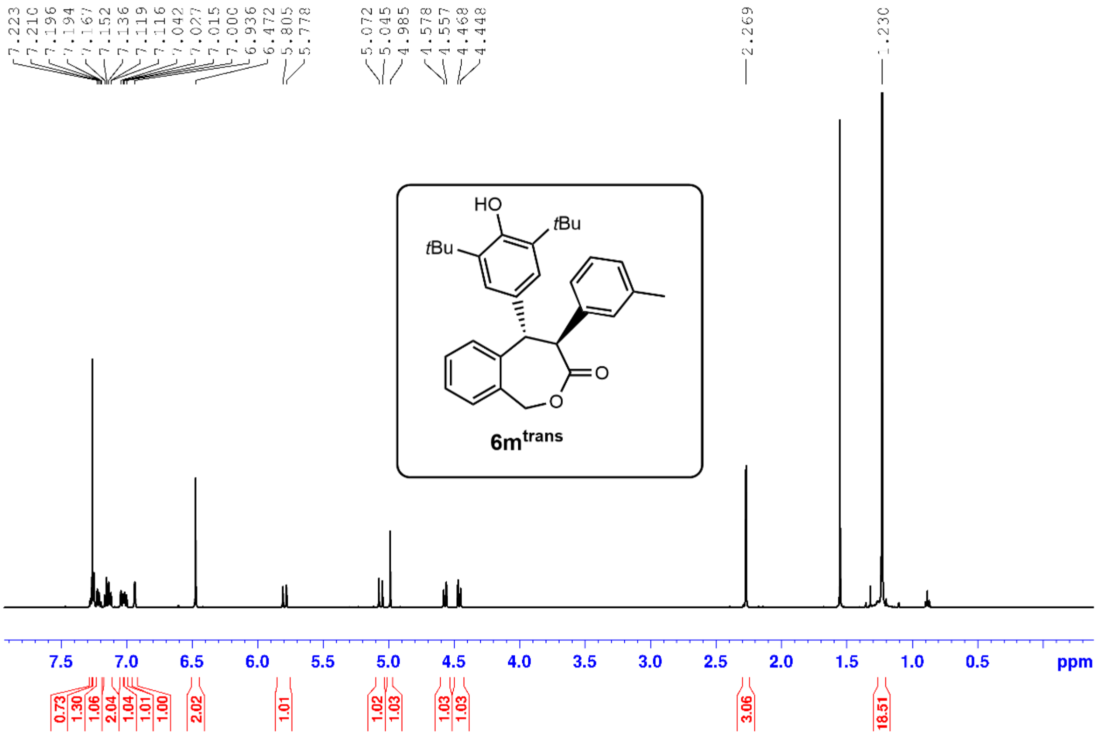

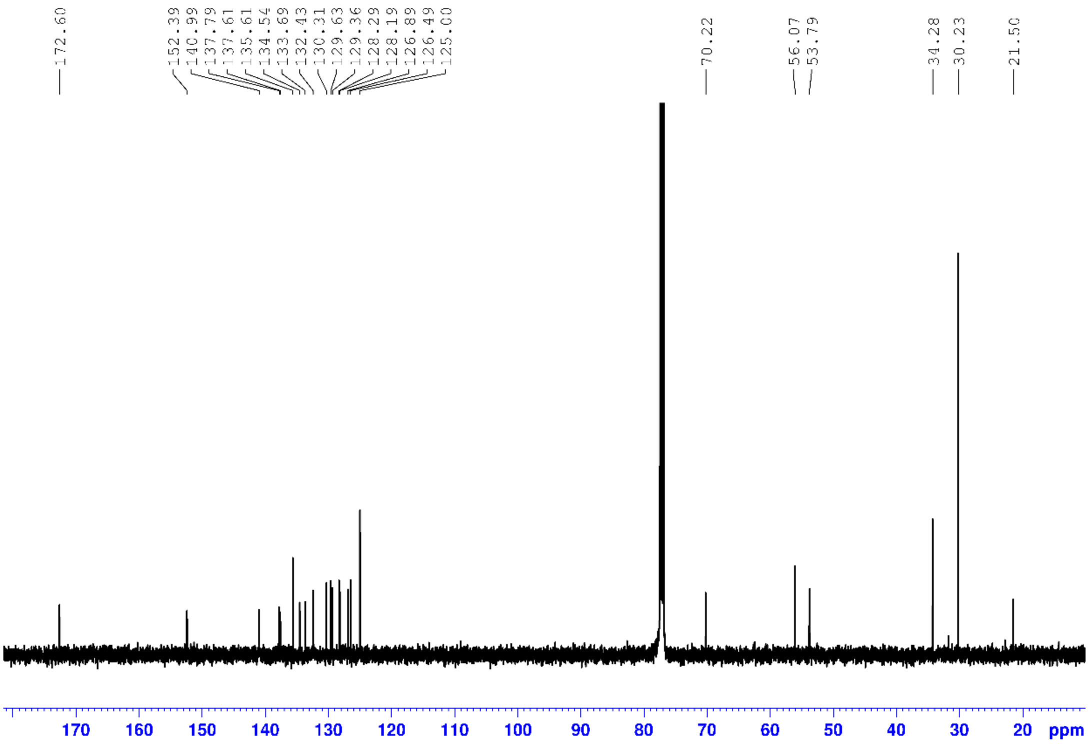

4-Methylphenyl benzo[c]oxepinone derivative (6n)

*Alkylation Product **5n***

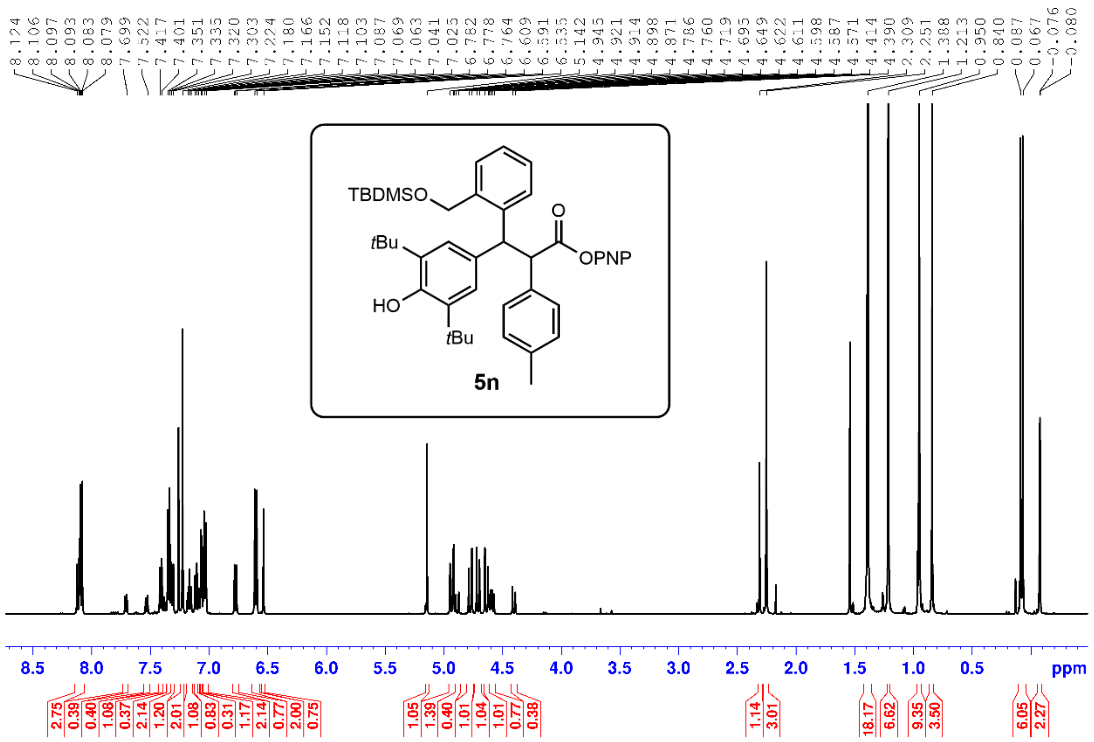

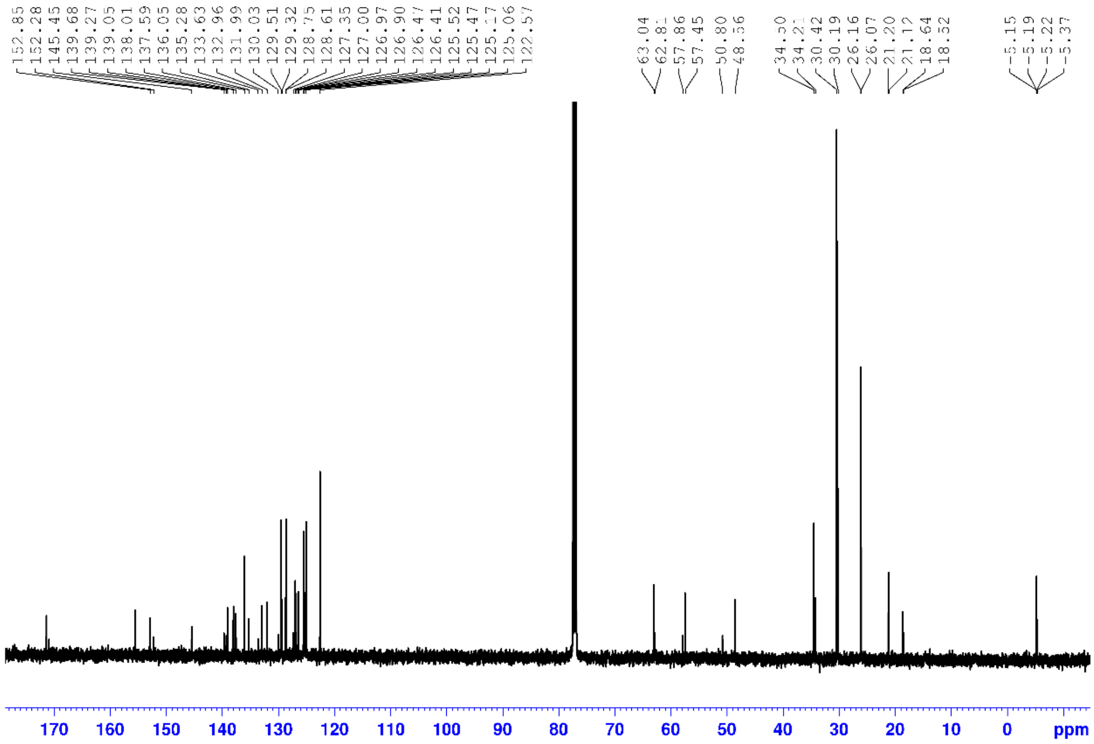

Cyclic cis product ***6n***^{cis}

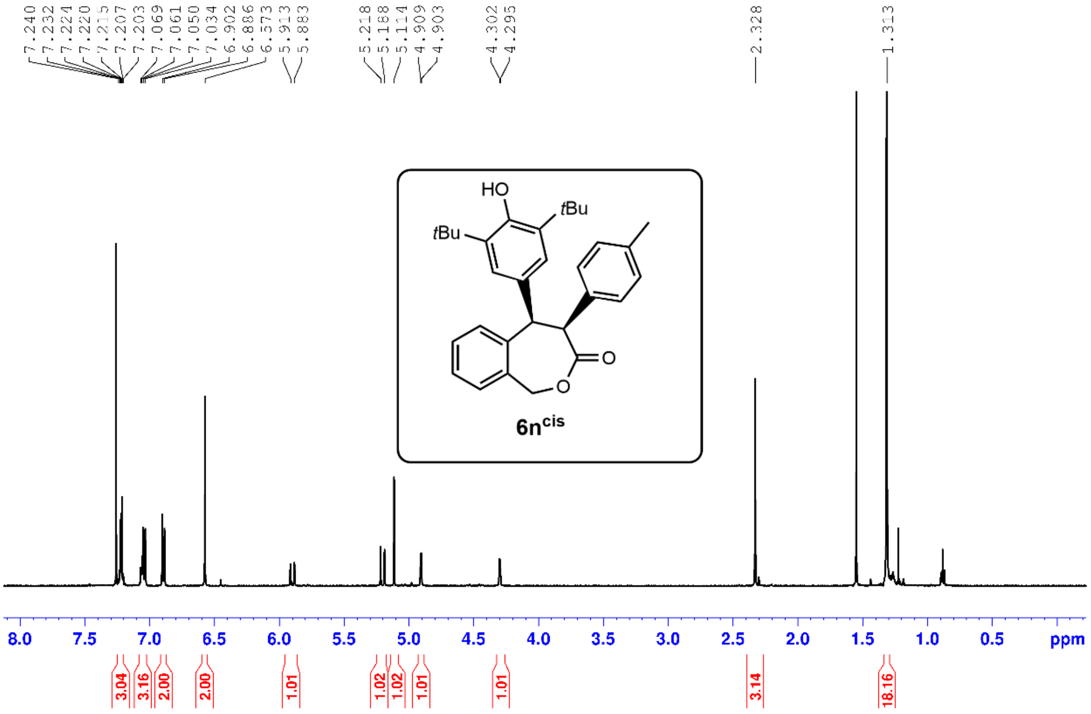

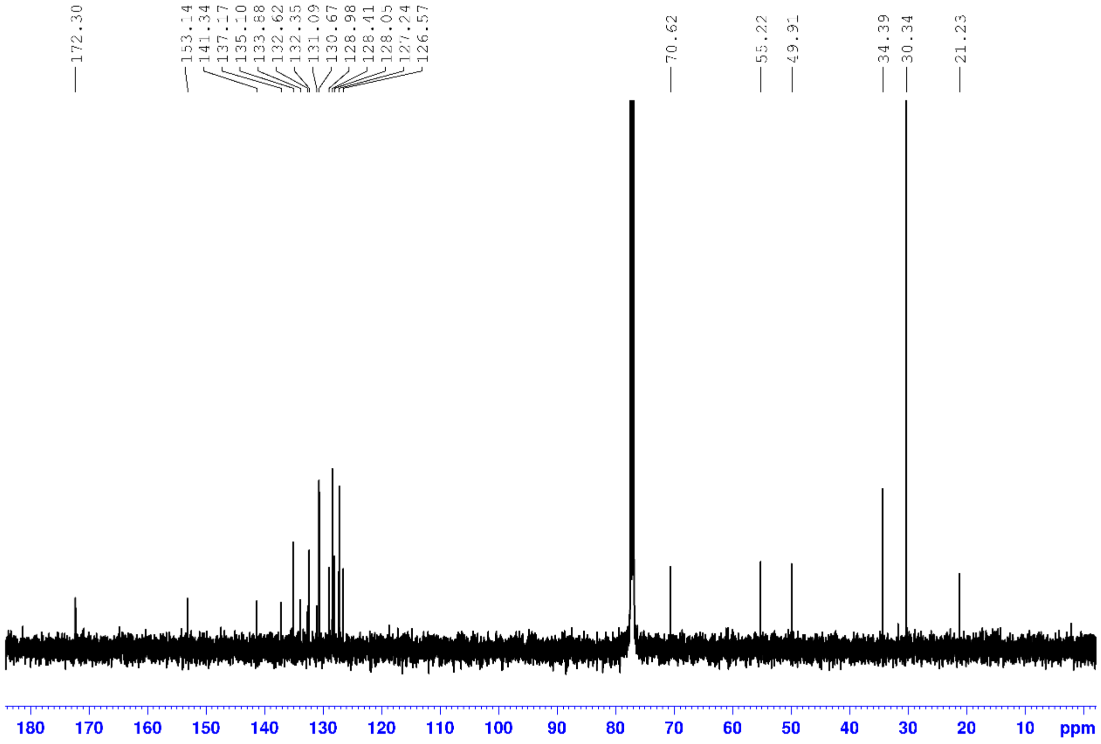

Cyclic trans product ***6n***^{trans}

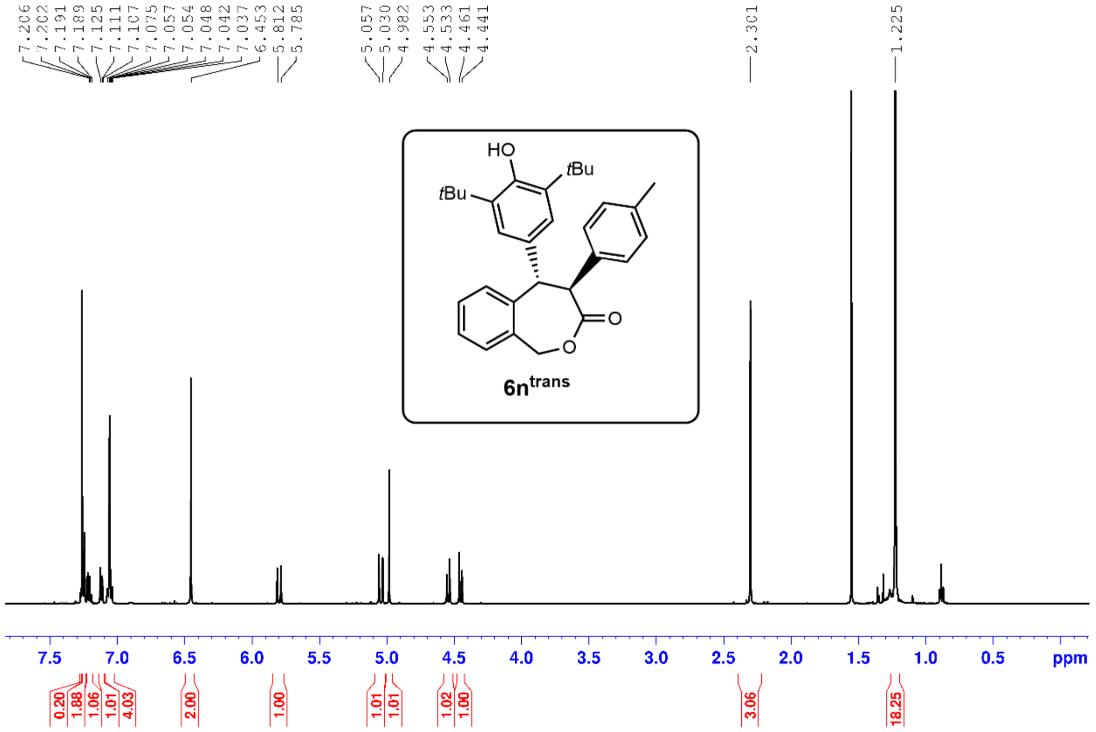

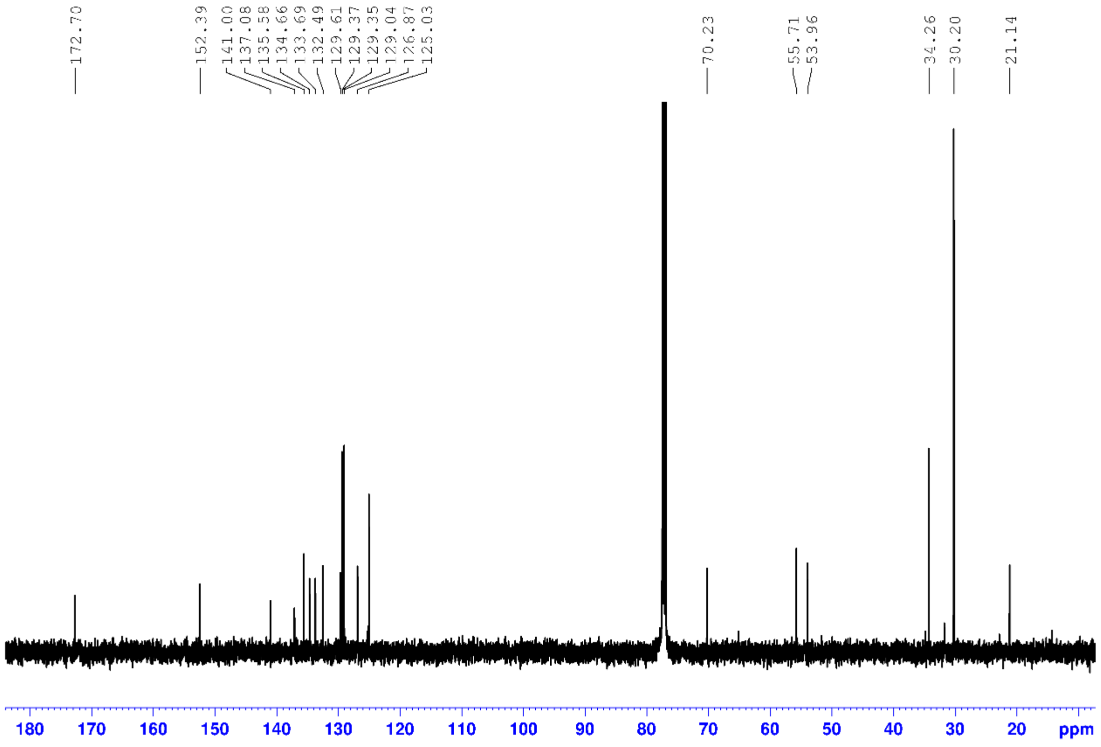

3,5-Dimethylphenyl benzo[c]oxepinone derivative (6o)

*Alkylation Product **5o***

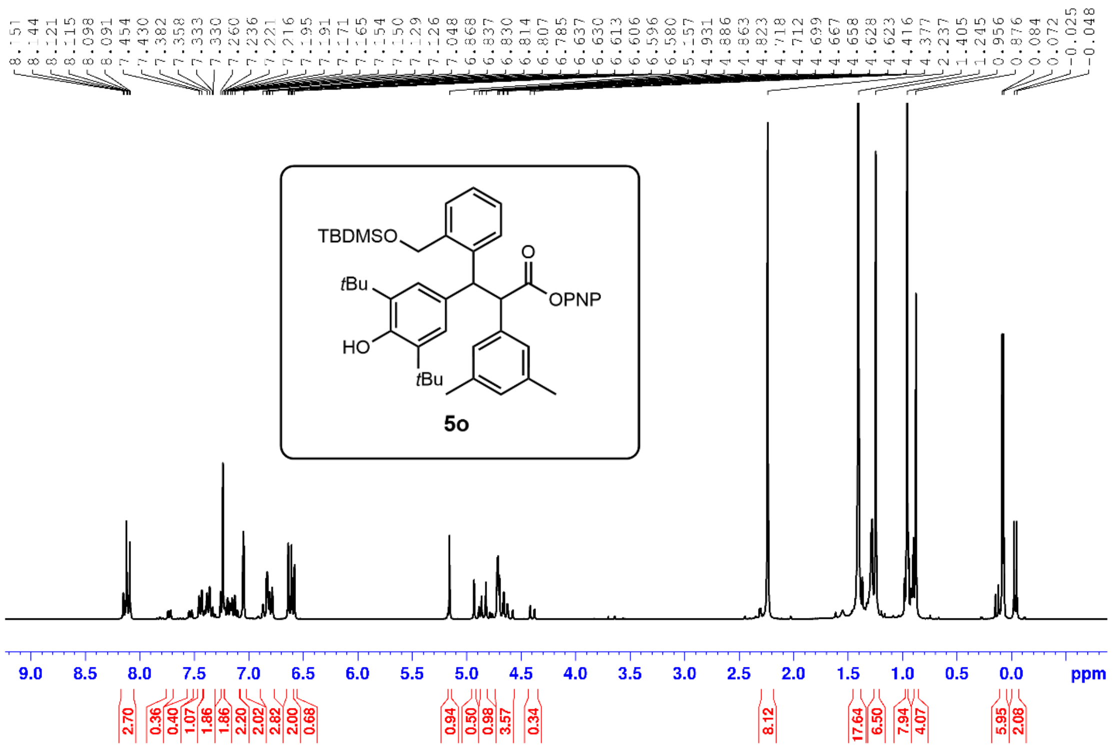

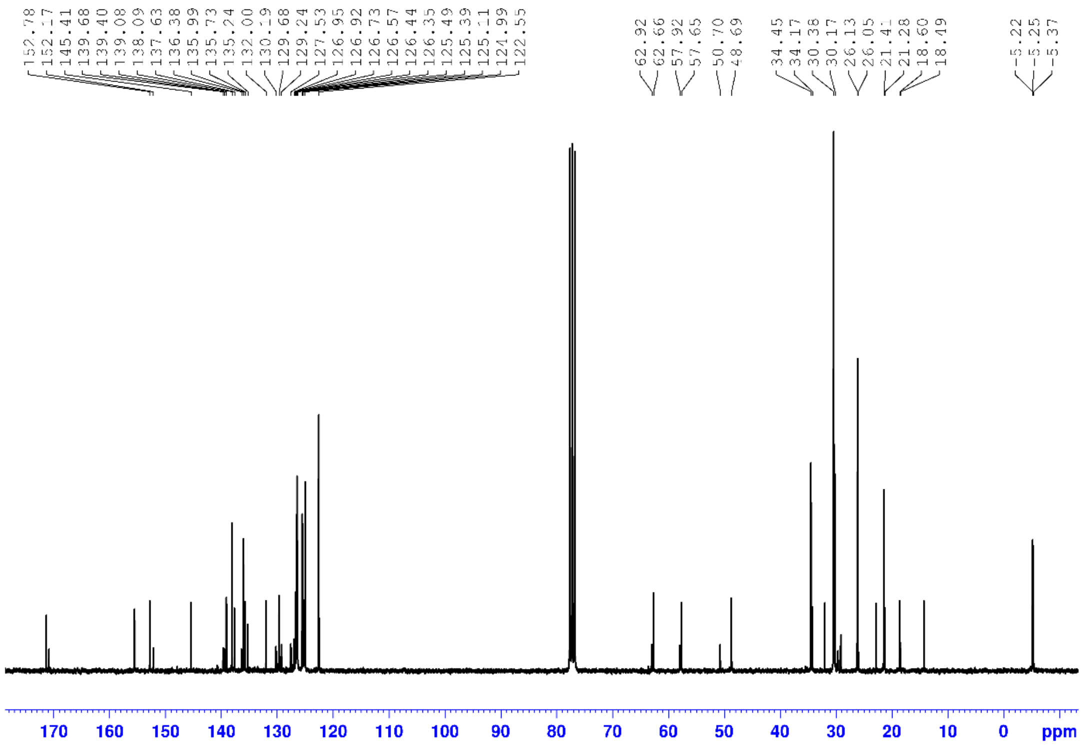

Cyclic cis product ***6o***^{cis}

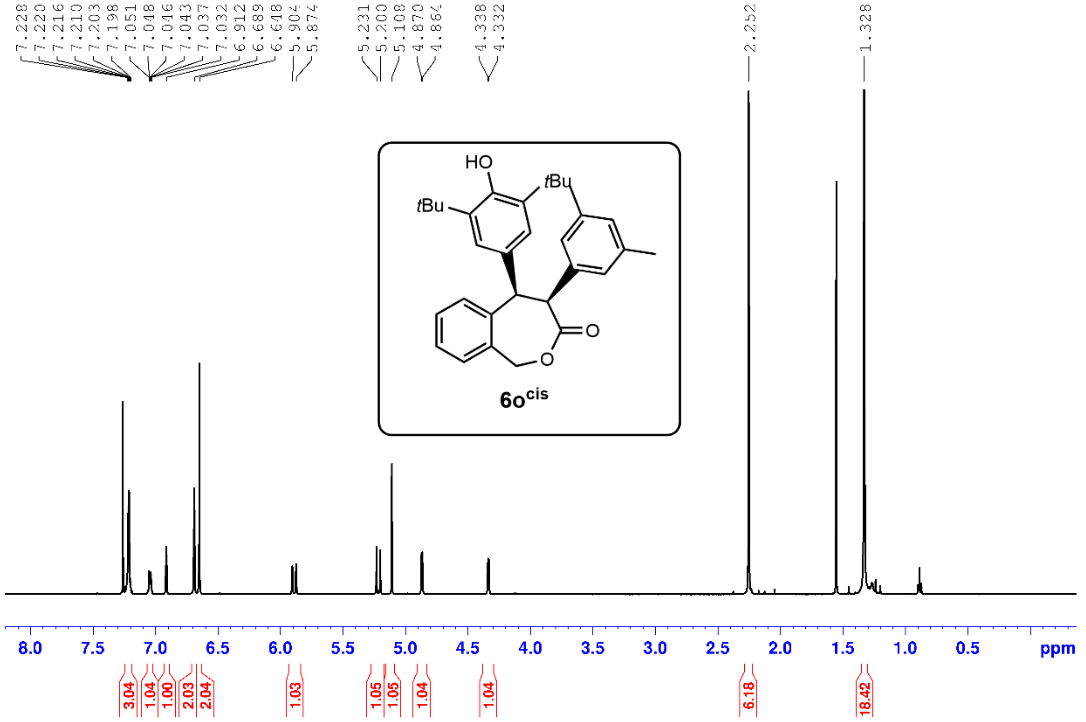

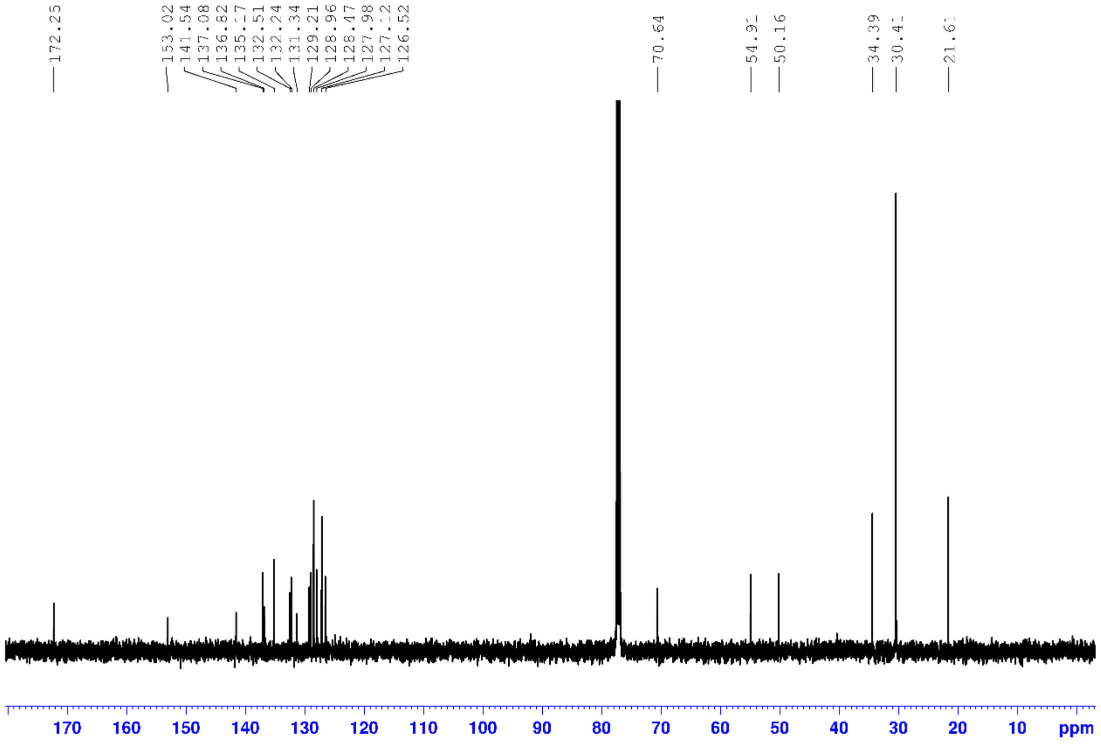

Cyclic trans product ***6o***^{trans}

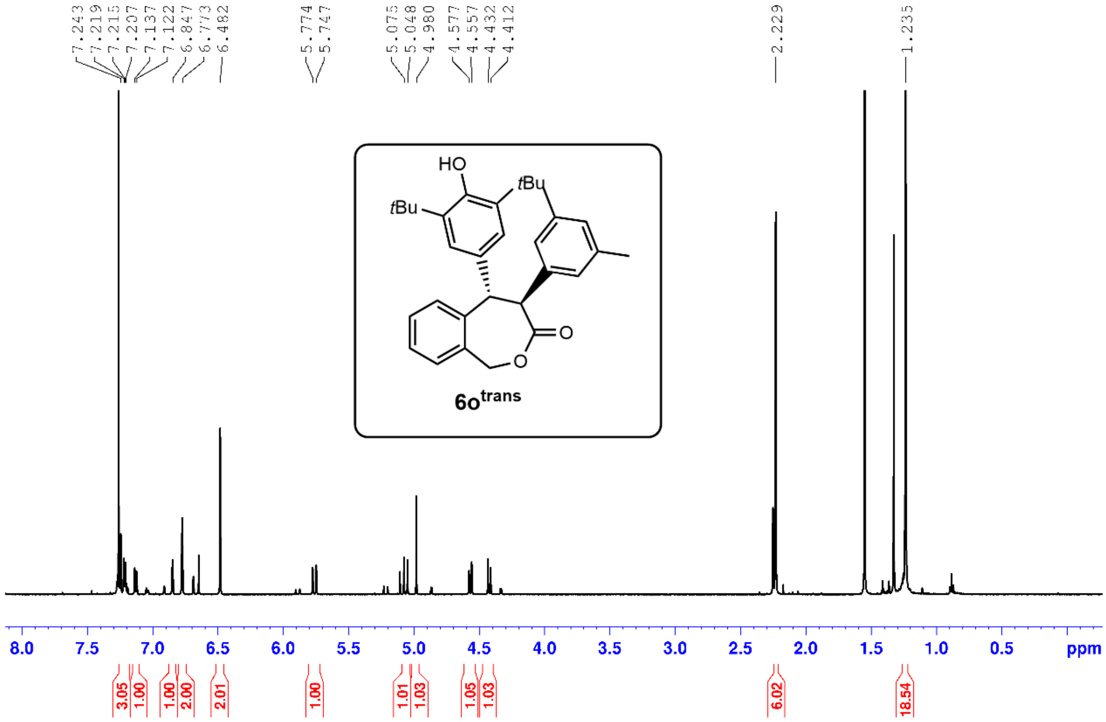

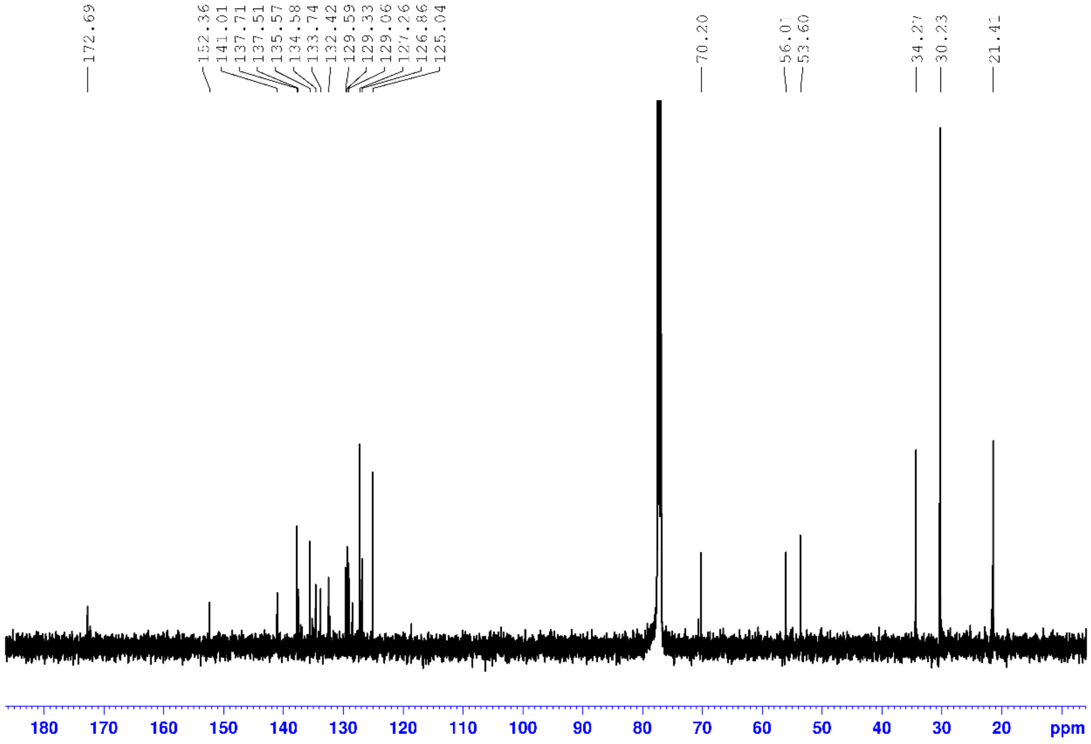

4-*t*-Butylphenyl benzo[c]oxepinone derivative (6p)

*Alkylation Product **5p***

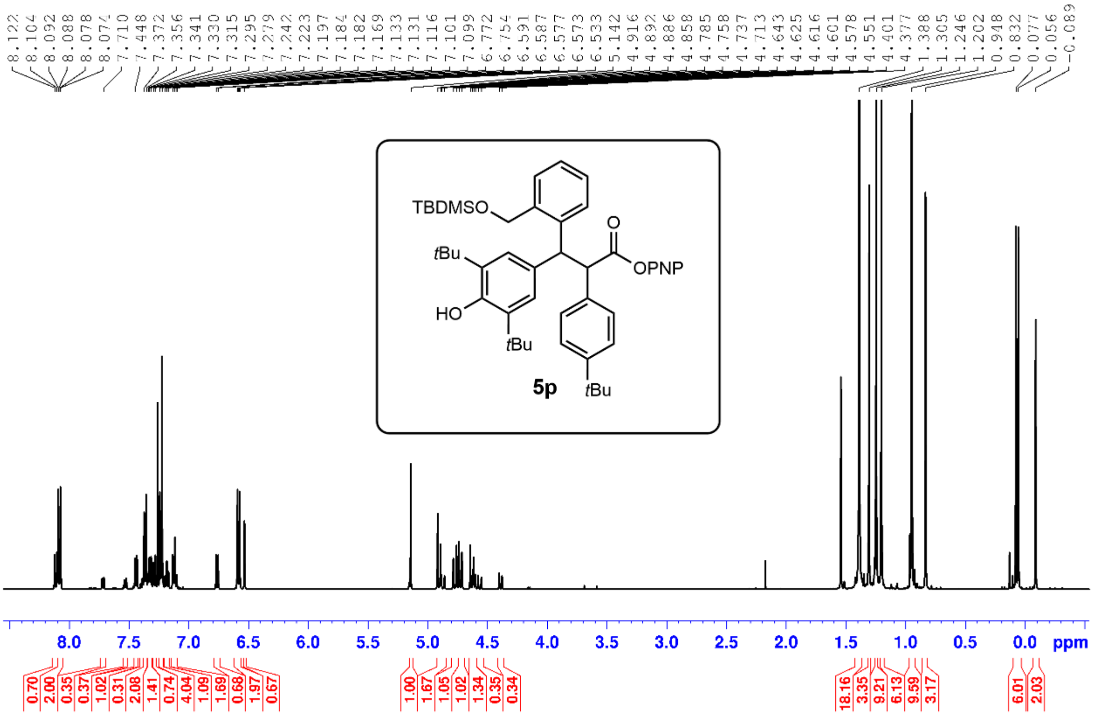

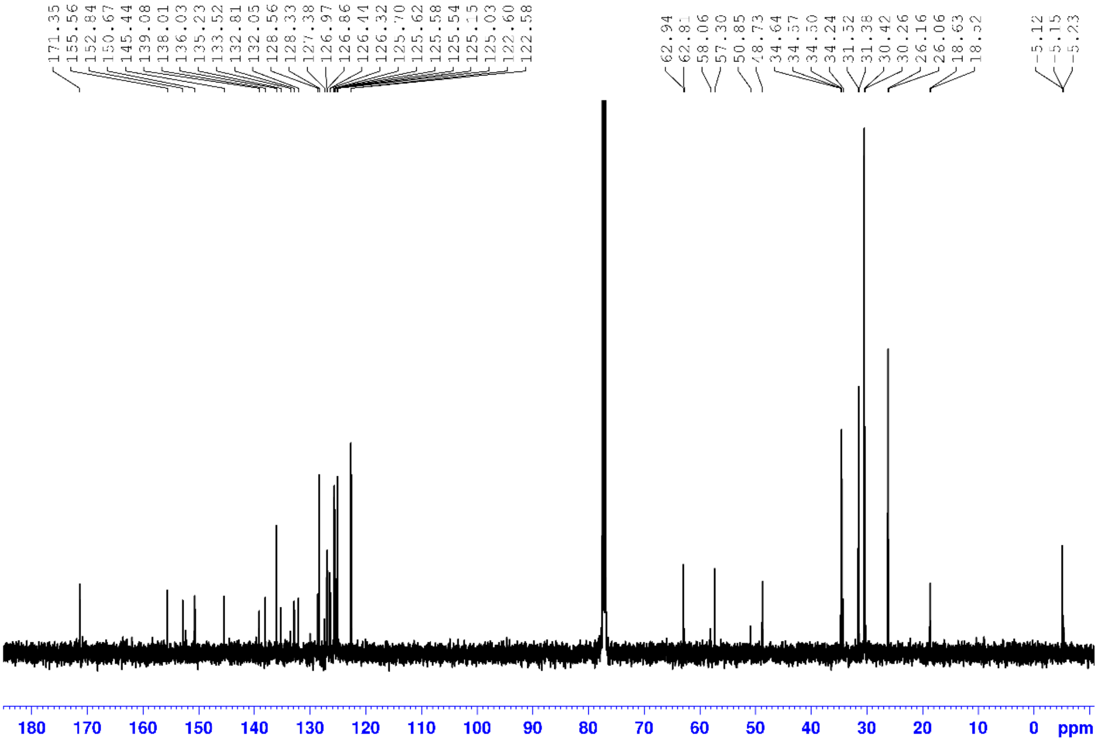

Cyclic products (mixture of diastereomers) ***6p**^{cis}* *and* ***6p**^{trans}*

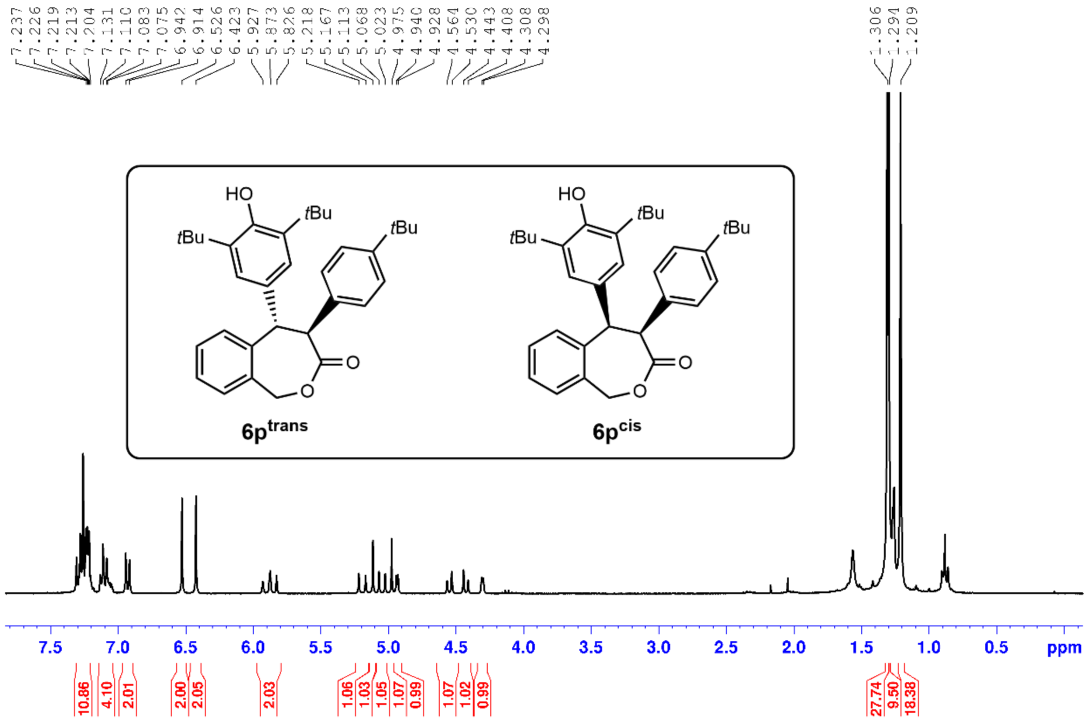

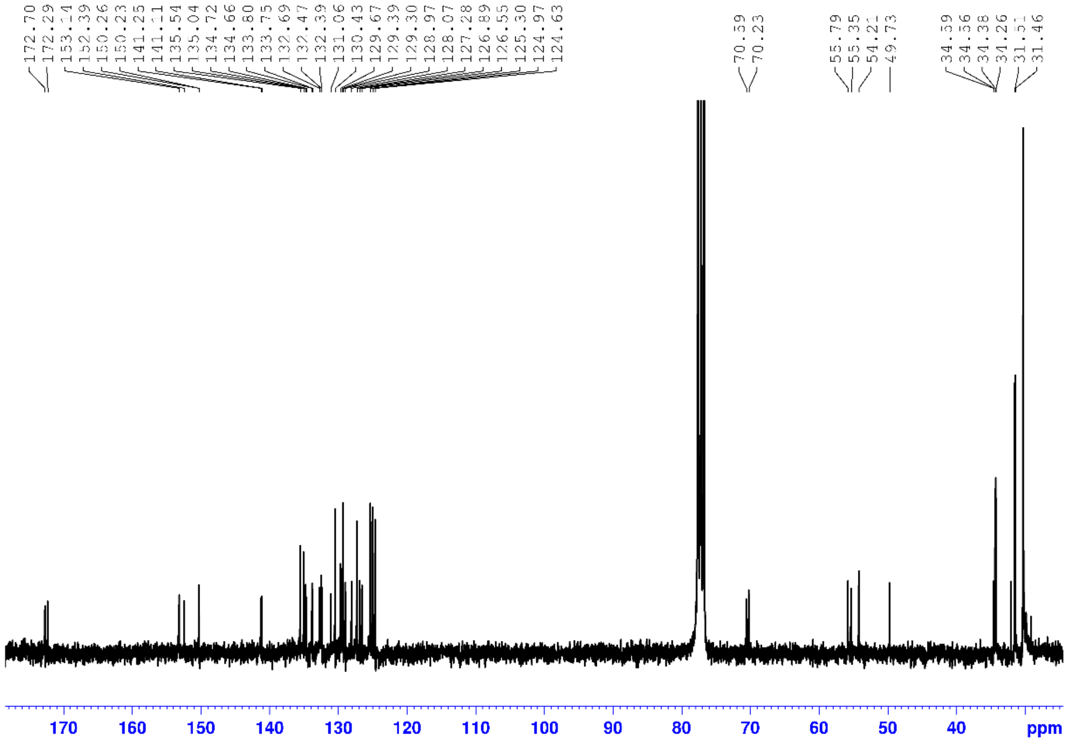

p-Phenyl benzo[c]oxepinone derivative (6q)

*Alkylation Product **5q***

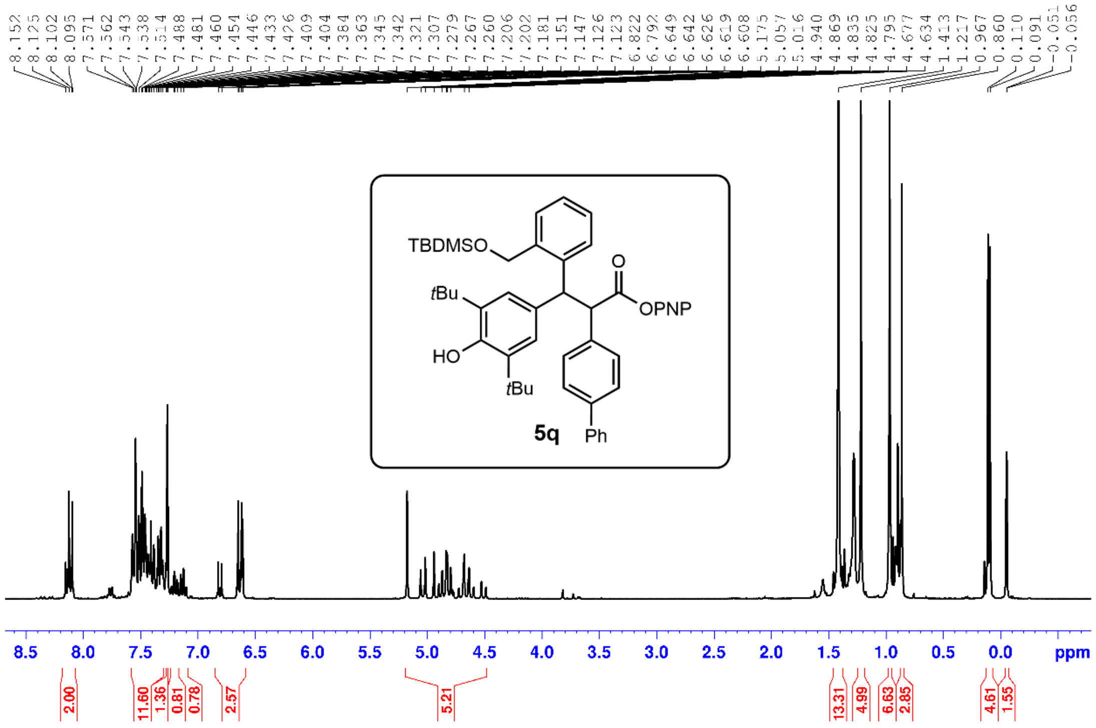

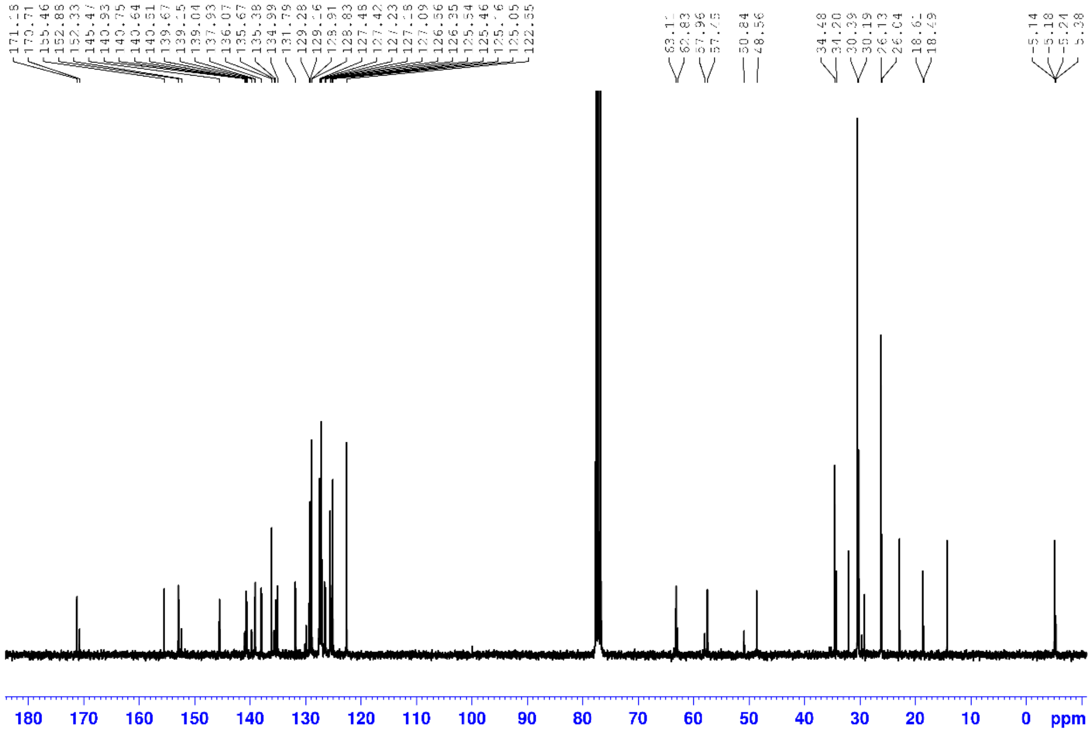

Cyclic cis product ***6q***^{cis}

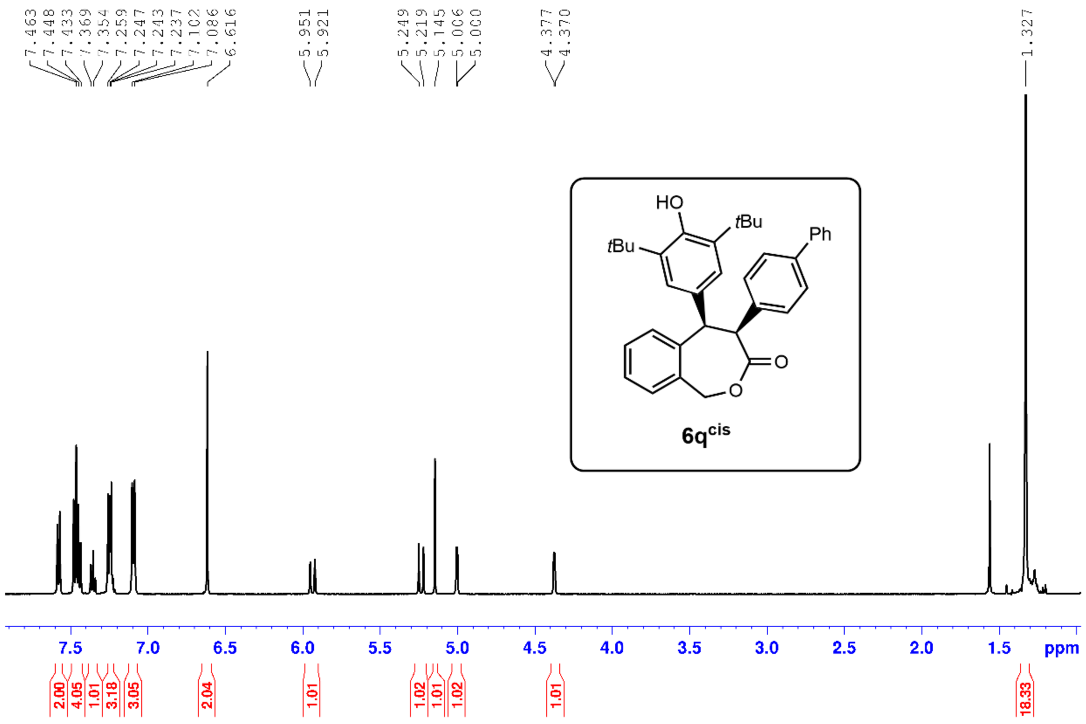

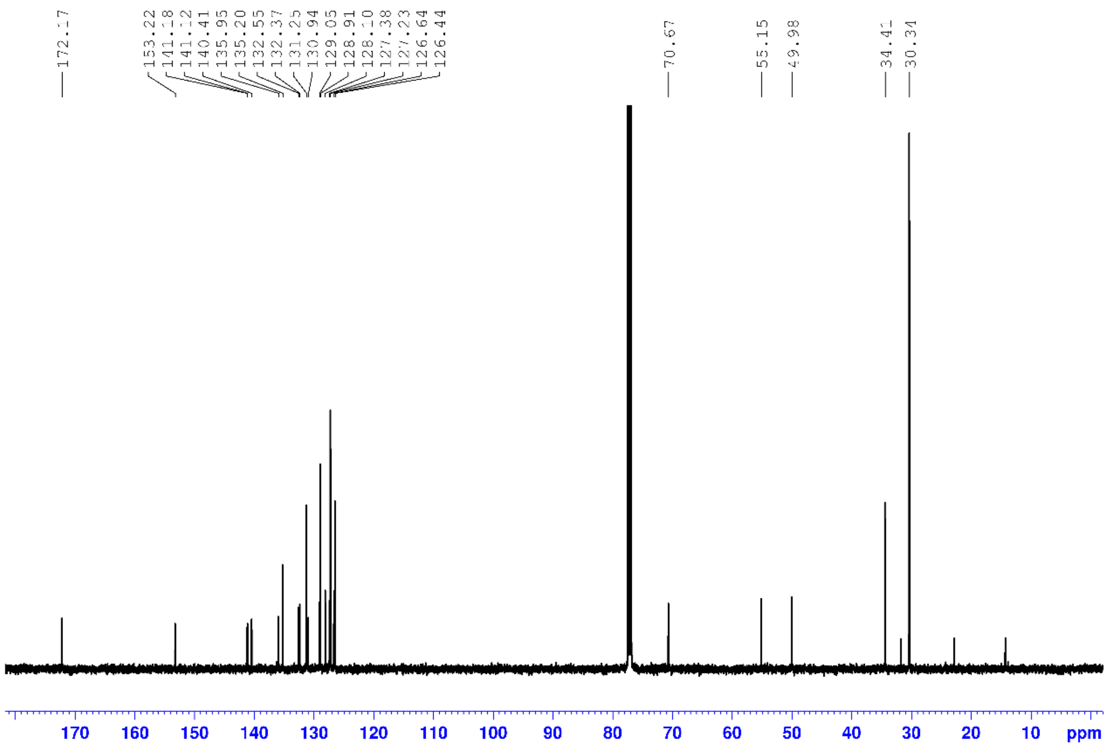

*Cyclic trans product **6q**^{trans}*

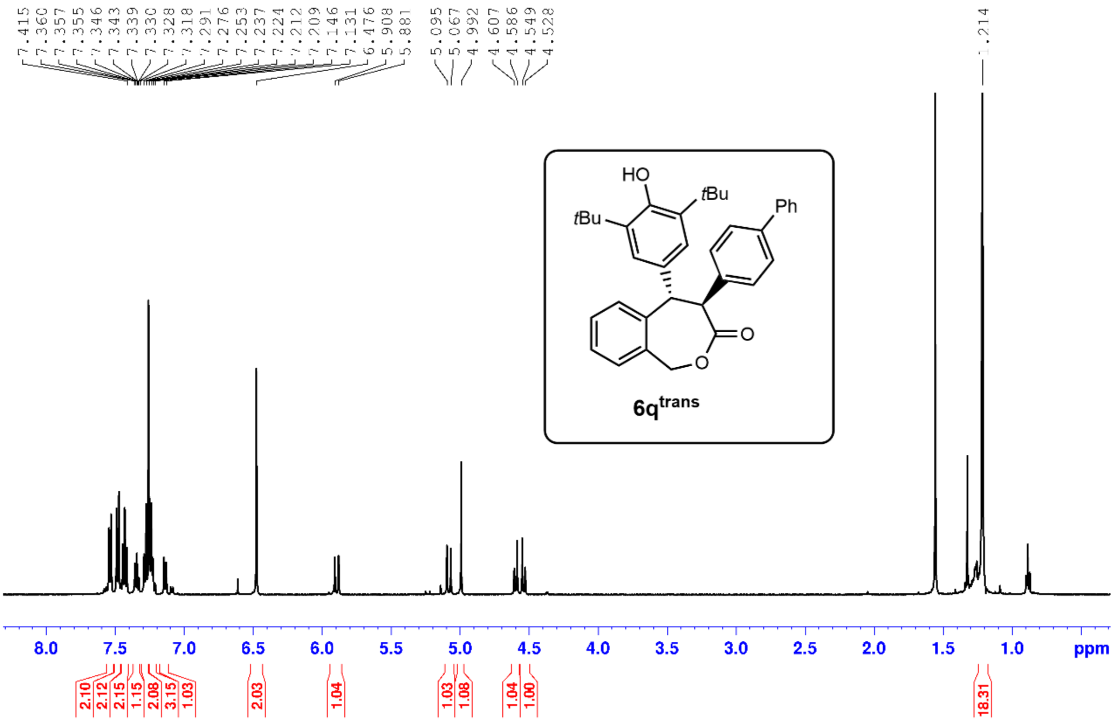

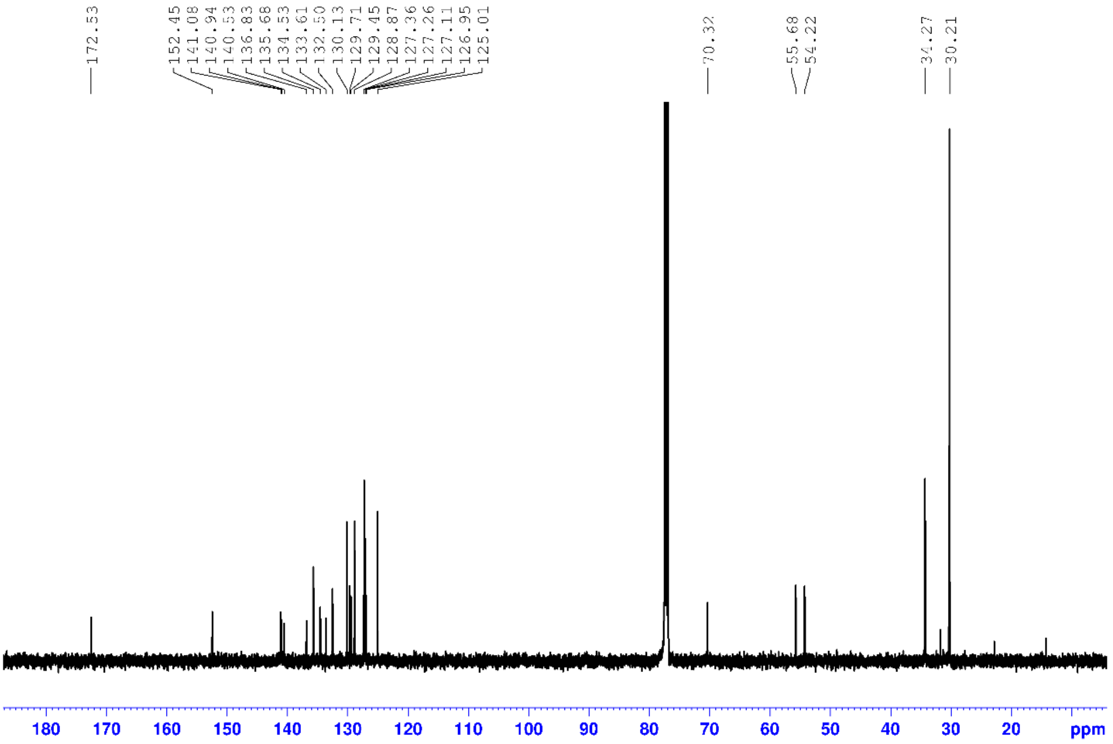

3-Trifluoromethylphenyl benzo[c]oxepinone derivative (6r)

*Alkylation Product **5r***

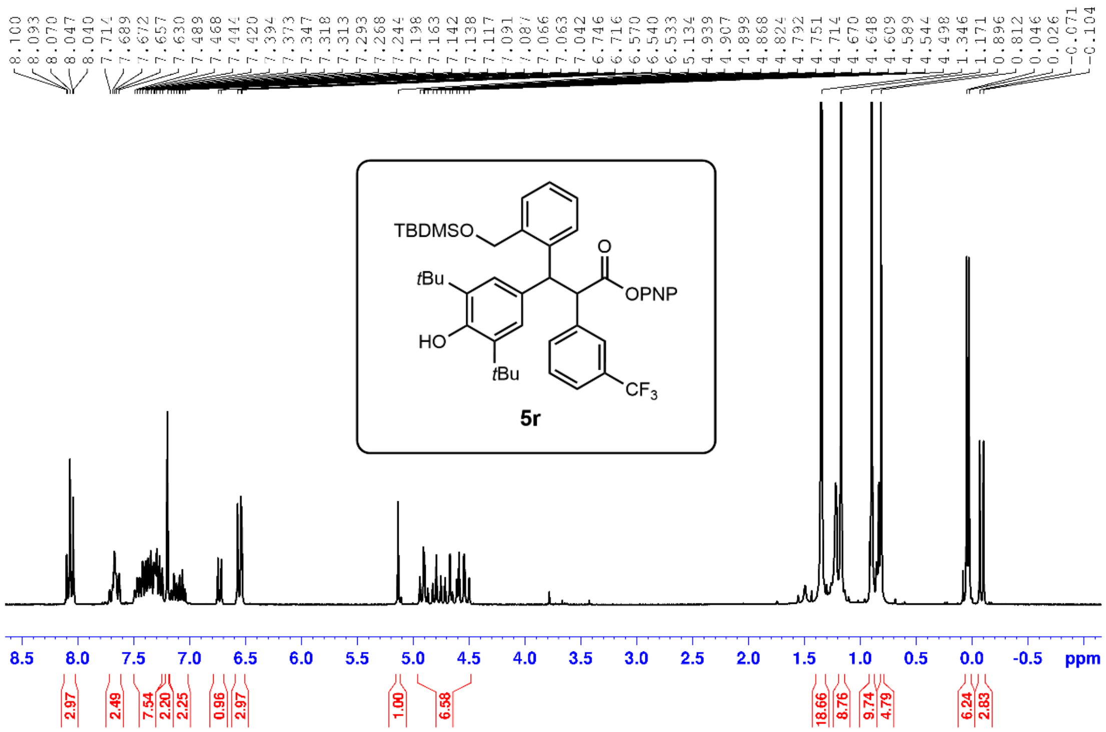

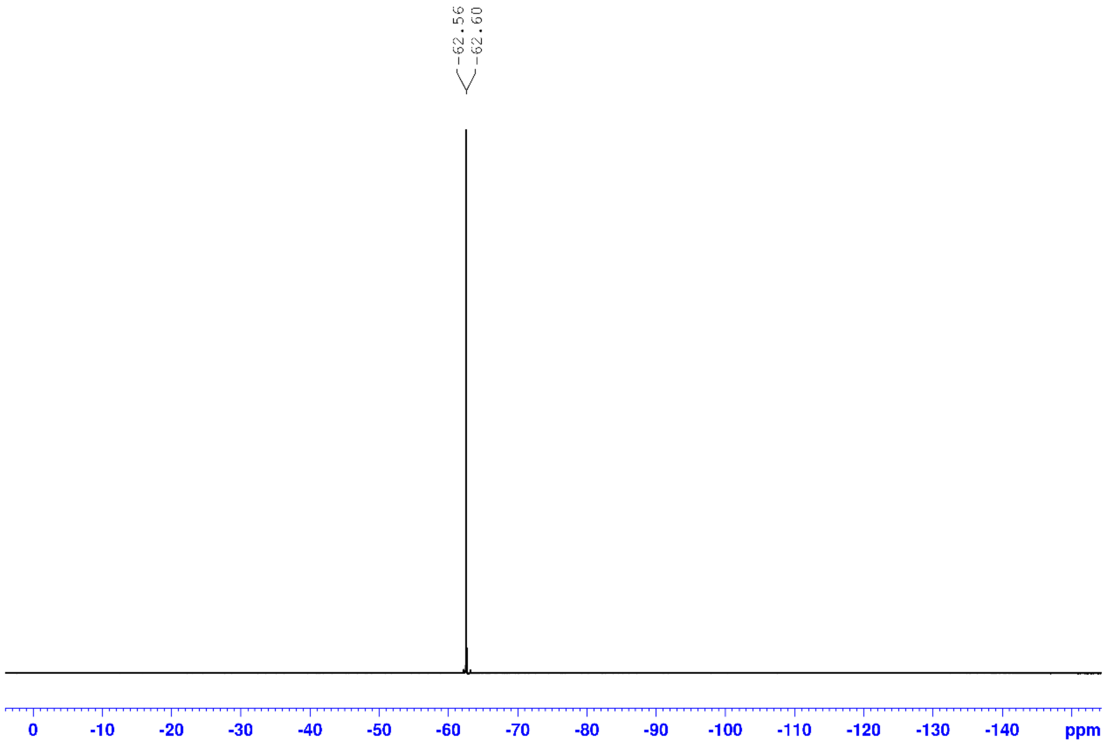

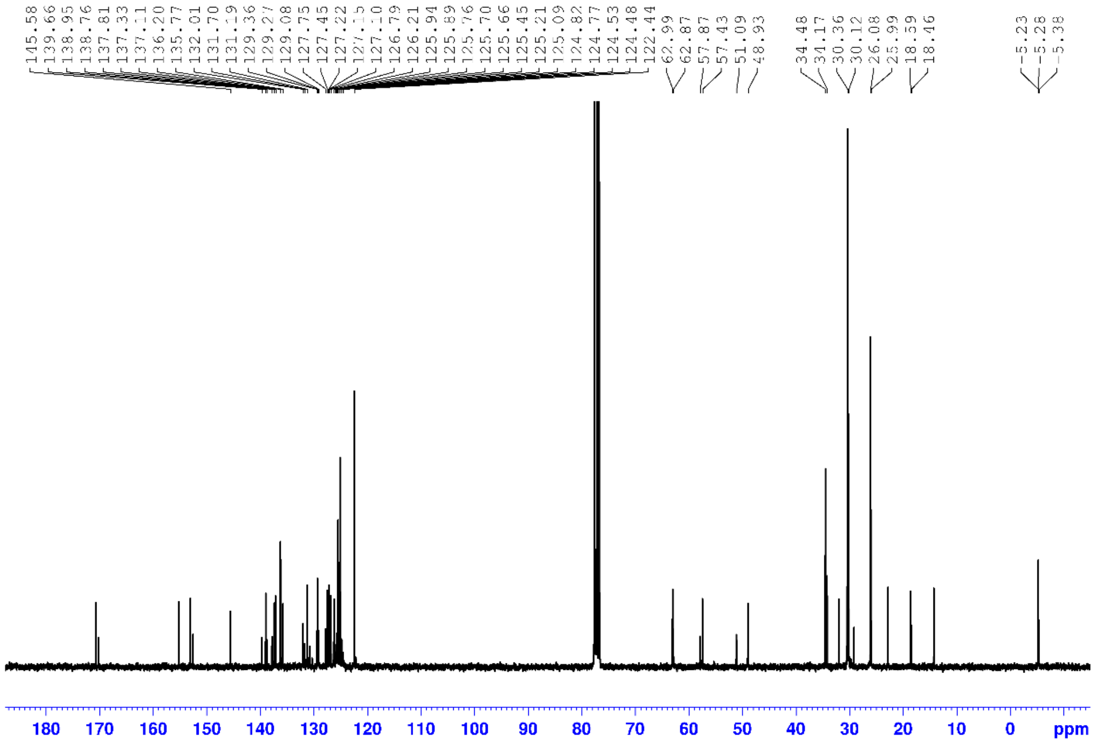
180
170
160
150
140
130
120
110
100
90
80
70
60
50
40
30
20
10
0
ppm

Cyclic products (mixture of diastereomers) ***6r**^{cis}* *and* ***6r**^{trans}*

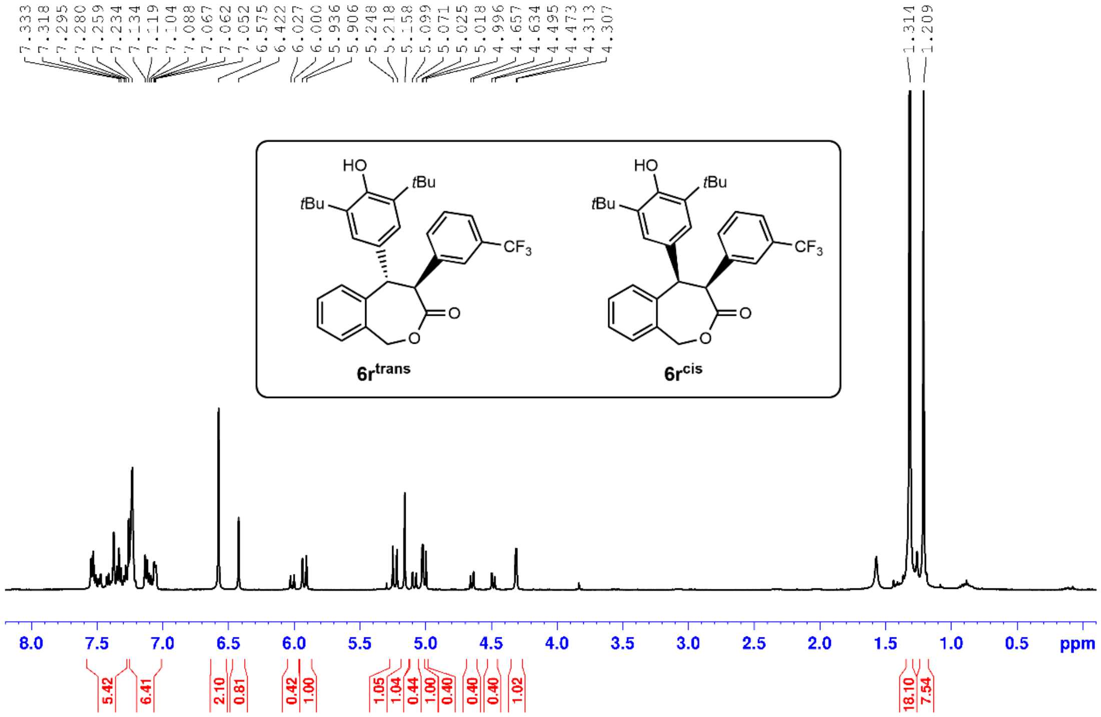

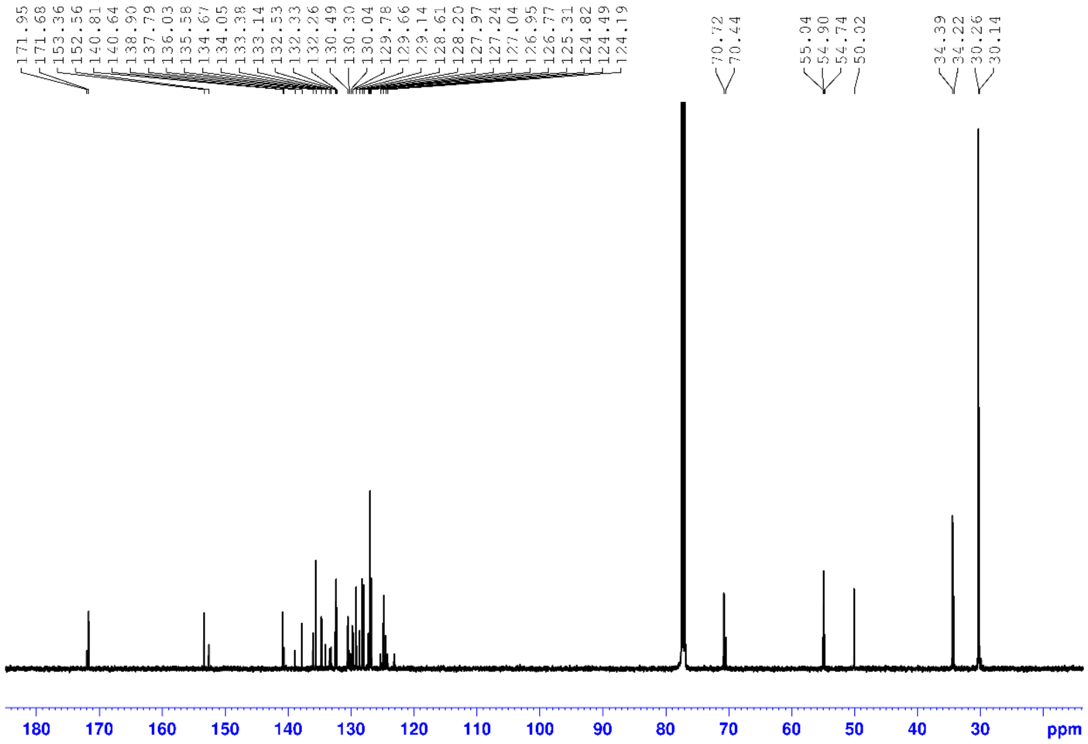

4-Trifluoromethylphenyl benzo[c]oxepinone derivative (6s)

Alkylation Product ***5s***

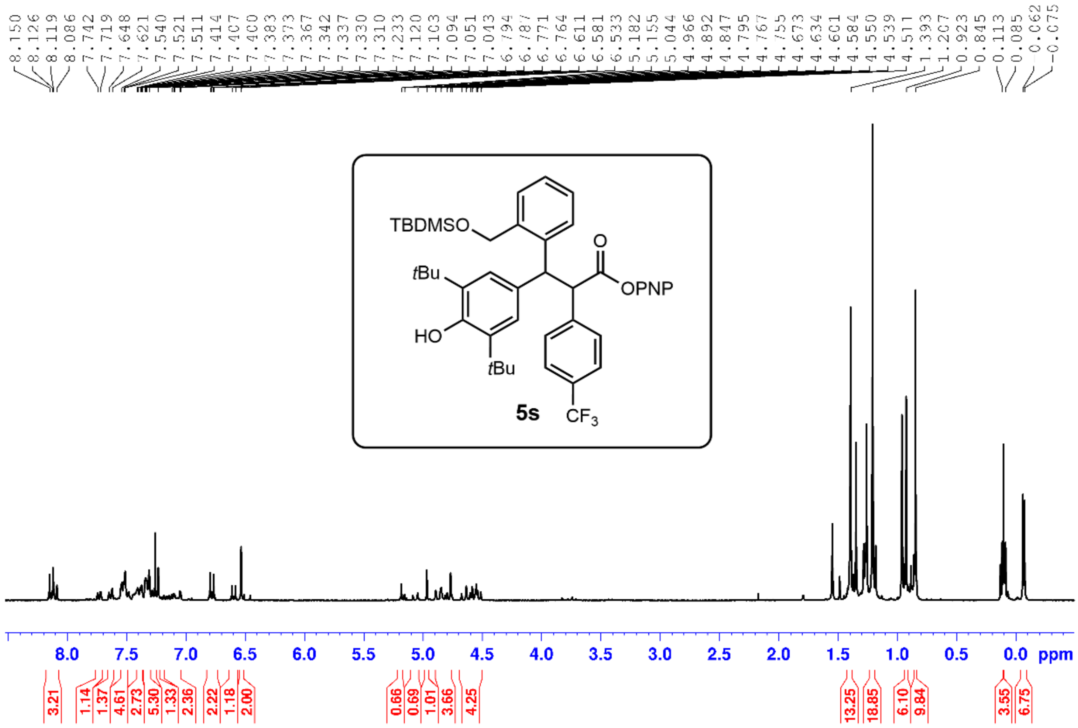

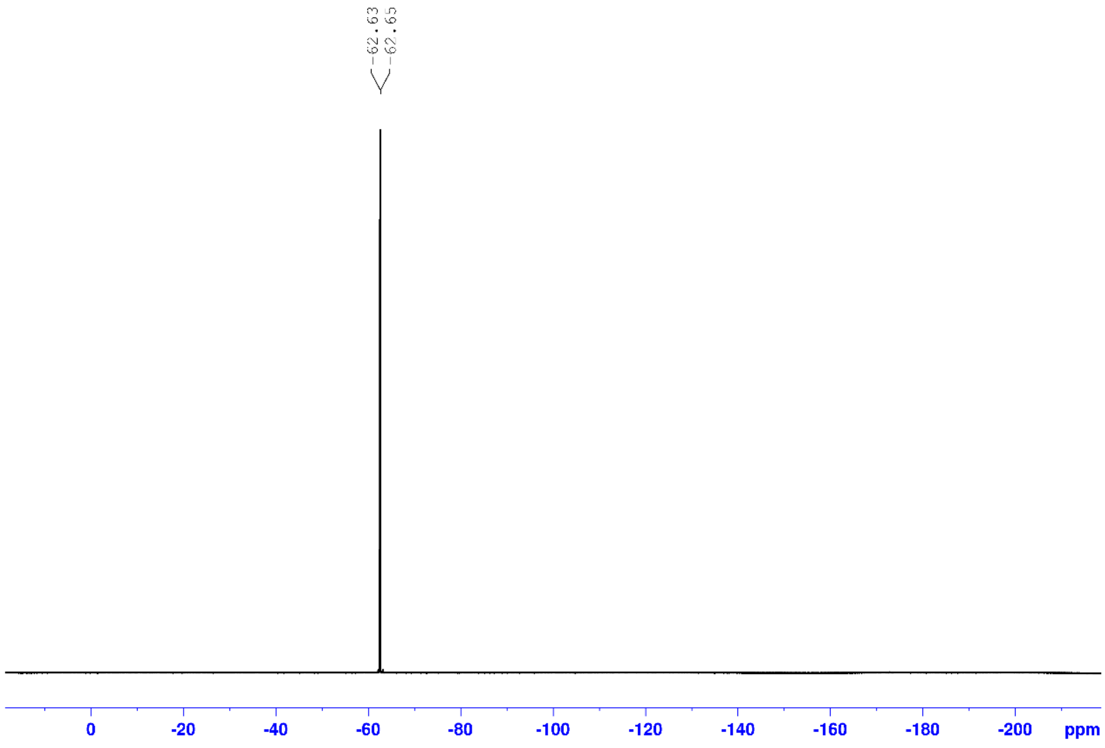

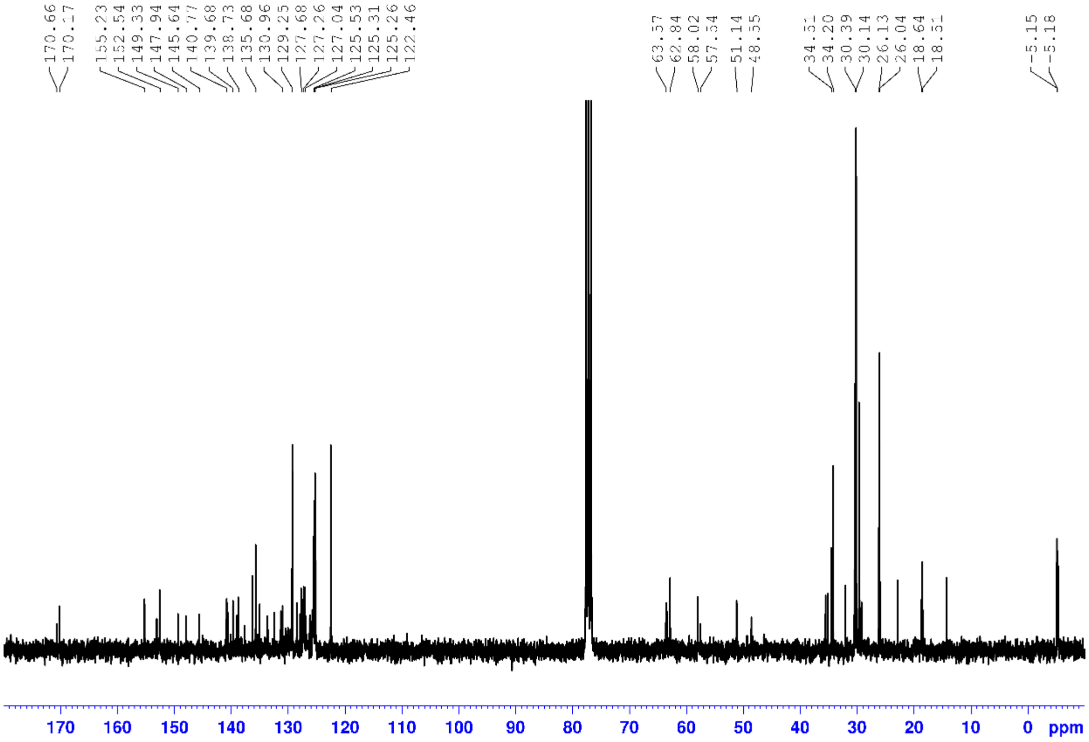
170.66
170.17
155.23
152.54
149.33
147.94
145.64
140.77
139.68
138.73
135.68
130.96
129.25
127.68
127.26
127.04
125.53
125.31
125.26
122.46
63.57
62.84
58.02
57.34
51.14
48.55
34.51
34.20
30.39
30.14
26.13
26.04
18.64
18.51
-5.15
-5.18
170
160
150
140
130
120
110
100
90
80
70
60
50
40
30
20
10
0
ppm

Cyclic cis product ***6s^{cis}***

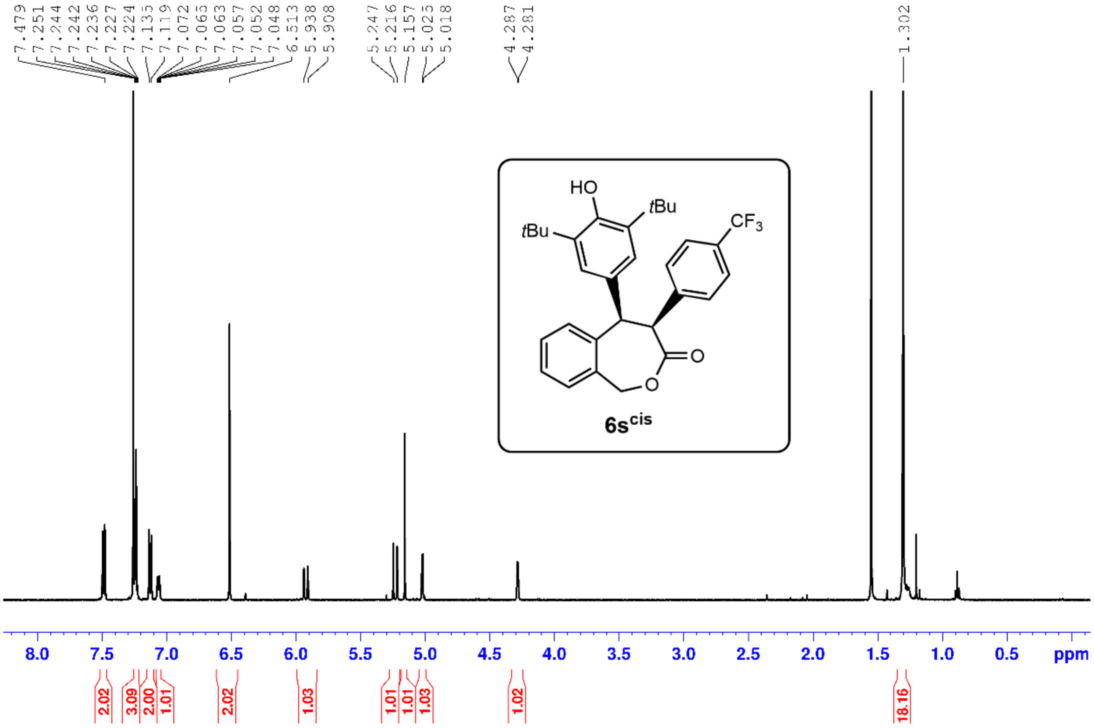

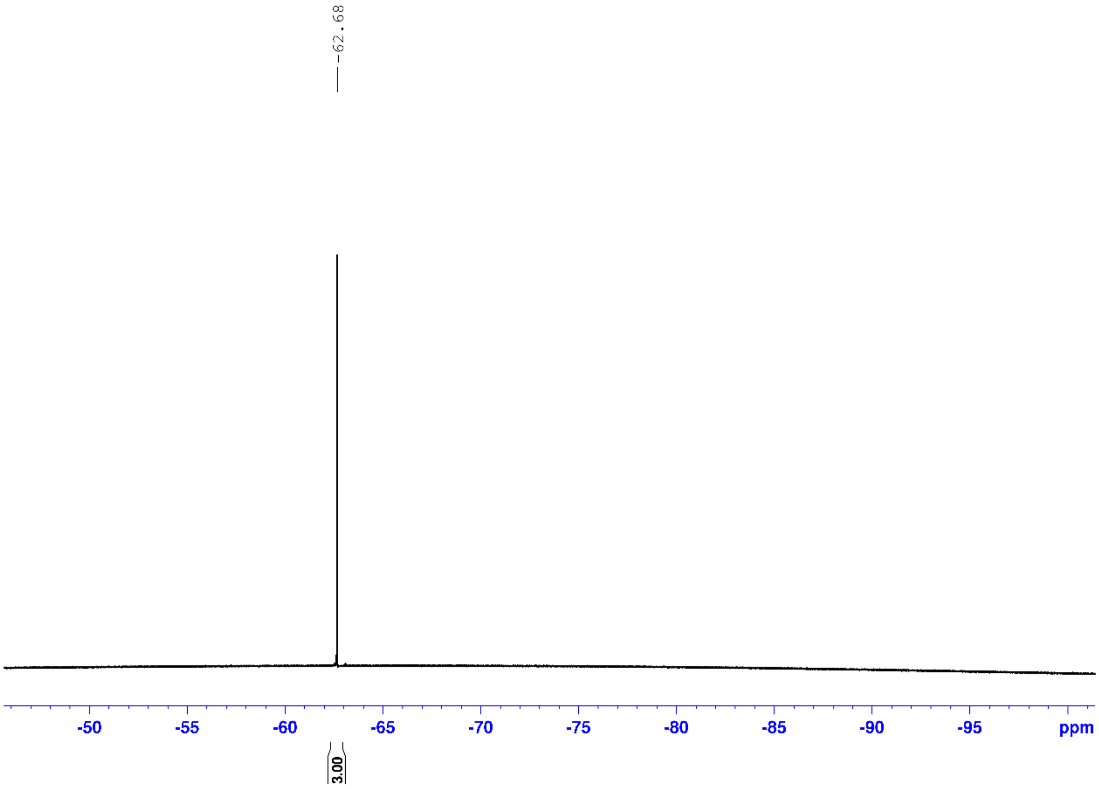

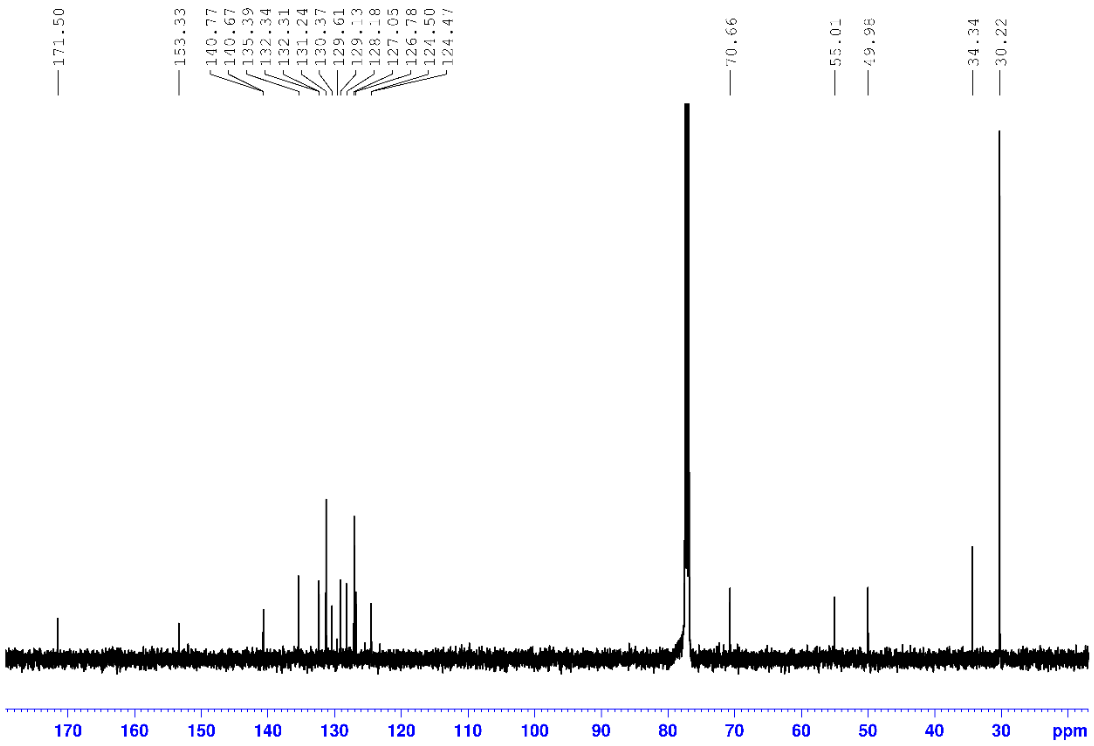
171.50
153.33
140.77
140.67
135.39
132.34
132.31
131.24
130.37
129.61
128.18
127.05
126.78
124.50
124.47
70.66
49.98
34.34
30.22
170
160
150
140
130
120
110
100
90
80
70
60
50
40
30
ppm

Cyclic trans product ***6s***^{trans}

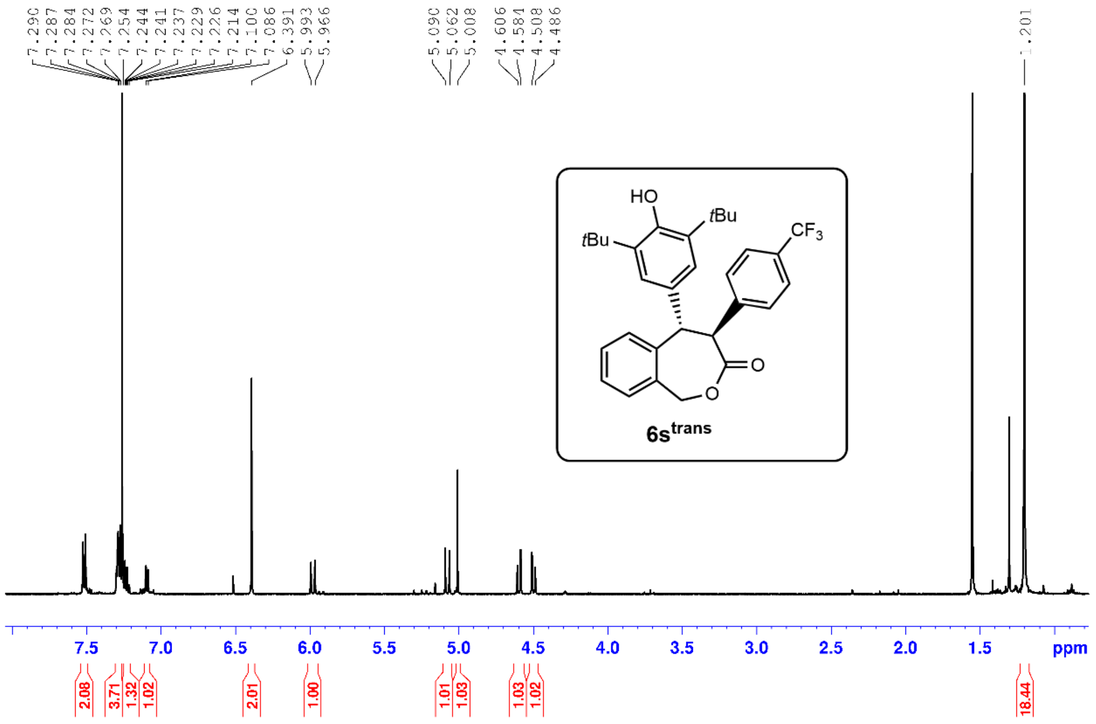

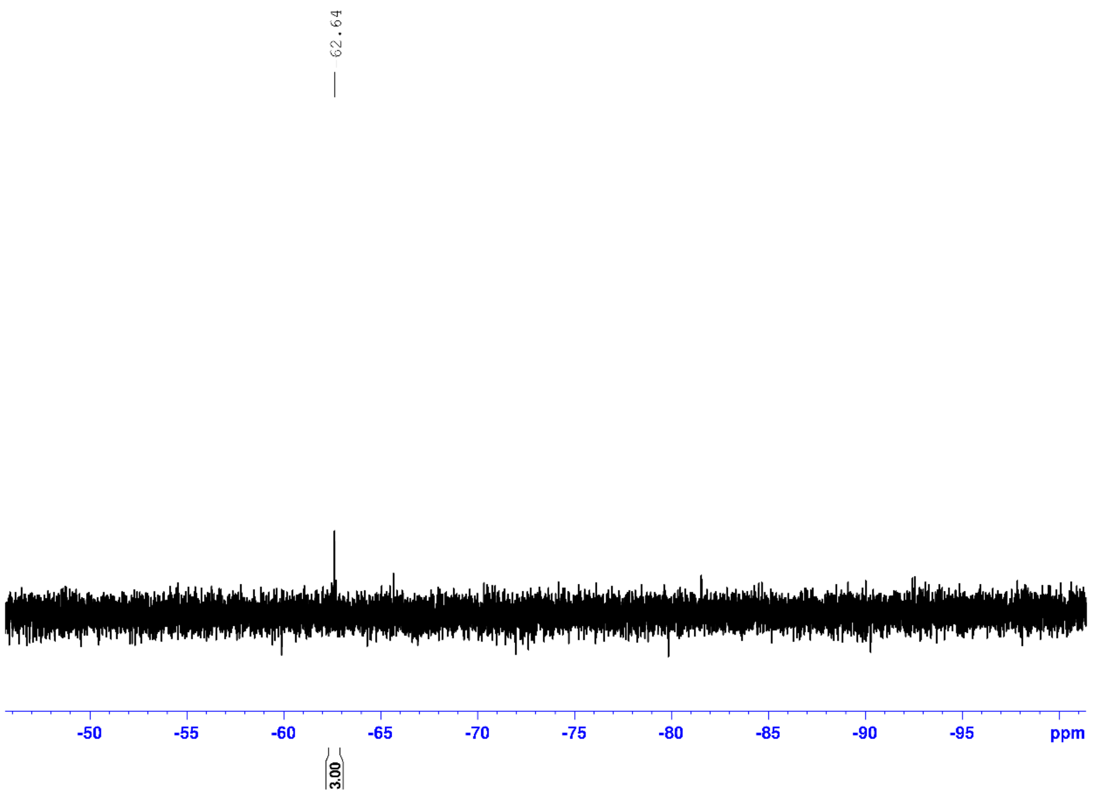

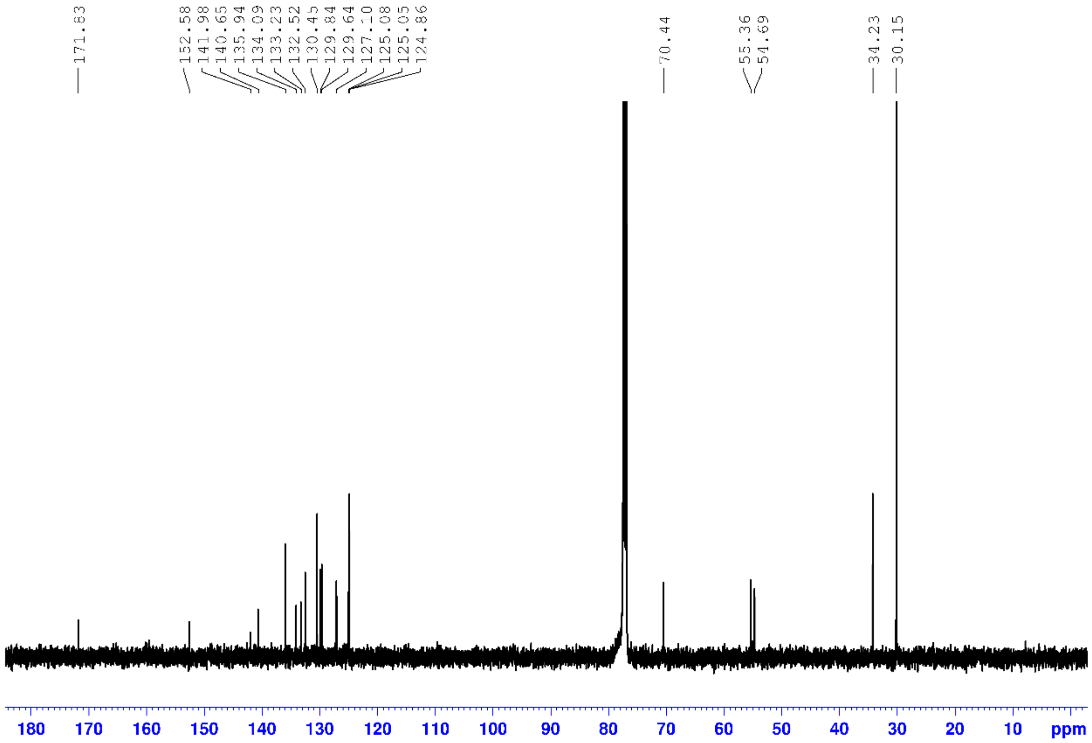
171.83
152.58
141.98
140.65
135.94
134.09
133.23
132.52
130.45
129.84
129.64
127.10
125.08
125.05
124.86
70.44
55.36
54.69
34.23
30.15
180 170 160 150 140 130 120 110 100 90 80 70 60 50 40 30 20 10 ppm

4-Nitrophenyl benzo[c]oxepinone derivative (6t)

*Alkylation Product **5t***

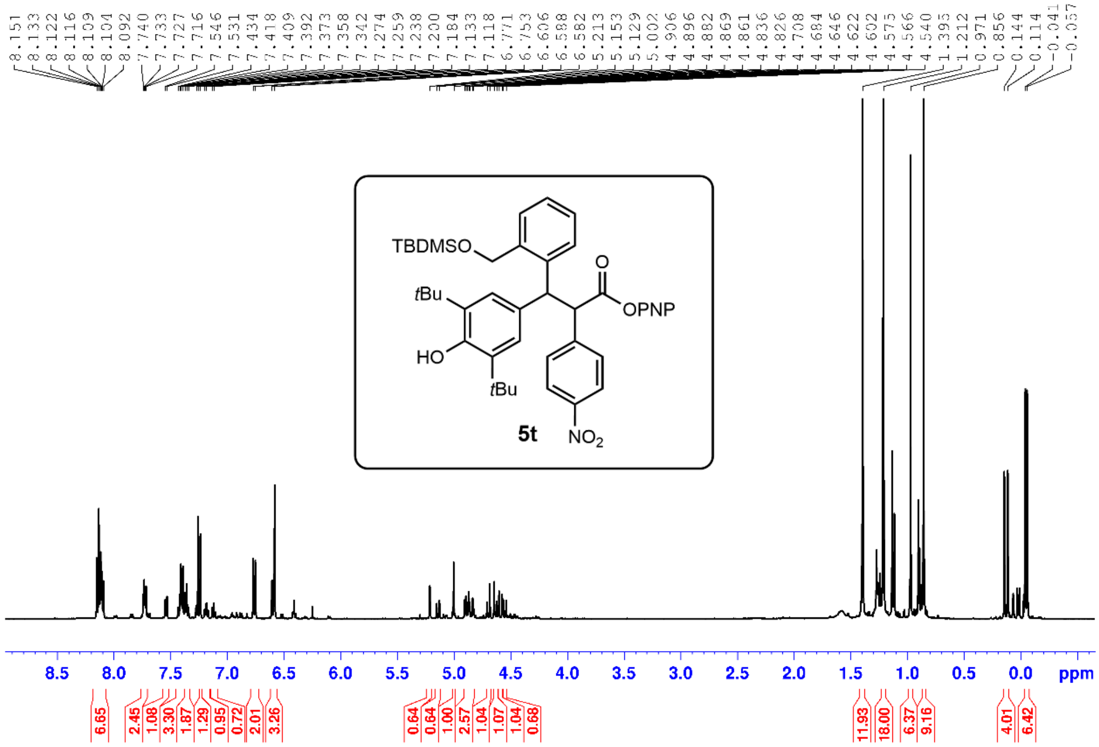

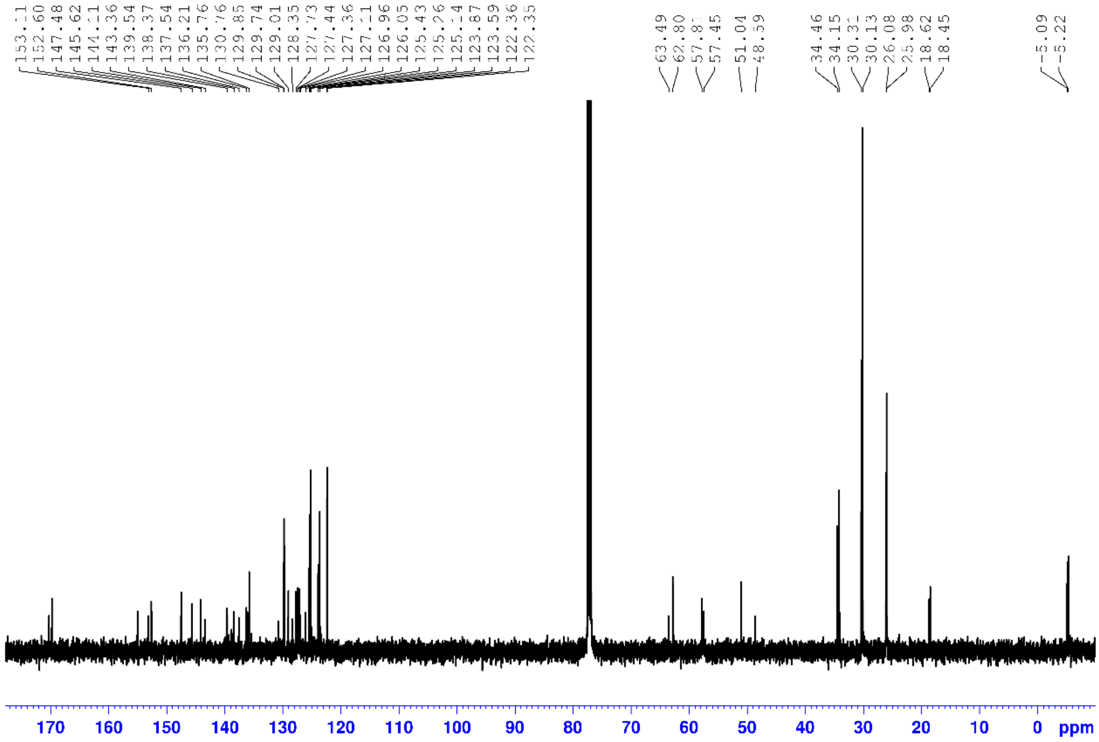

Cyclic cis product ***6t***^{cis}

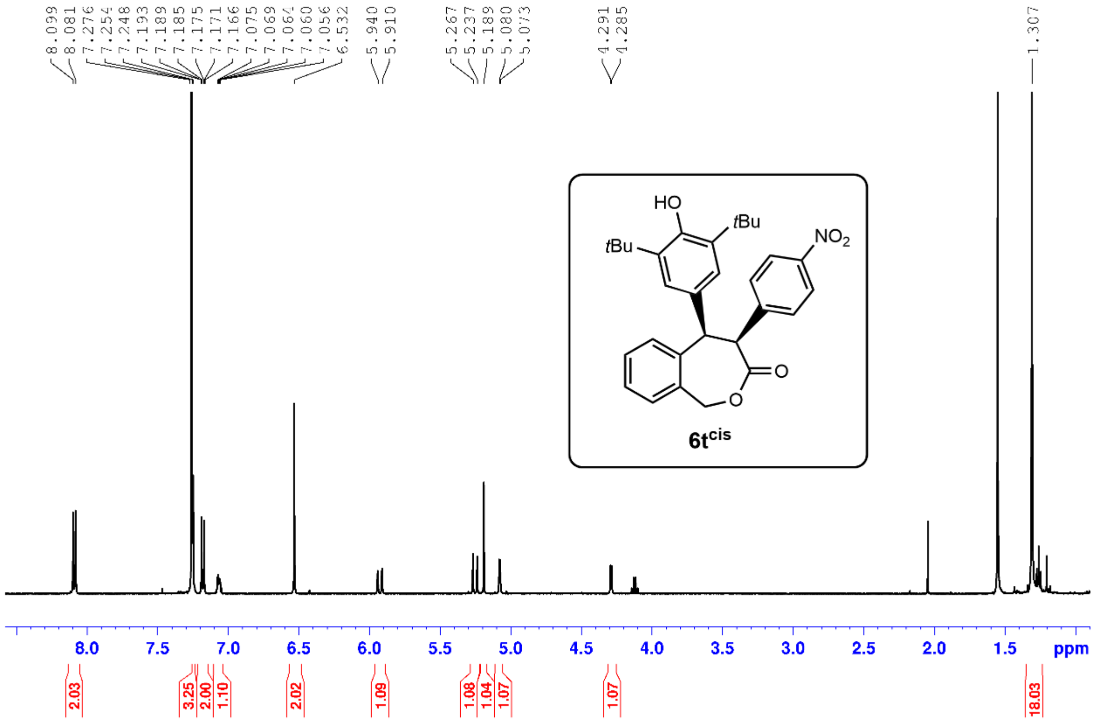

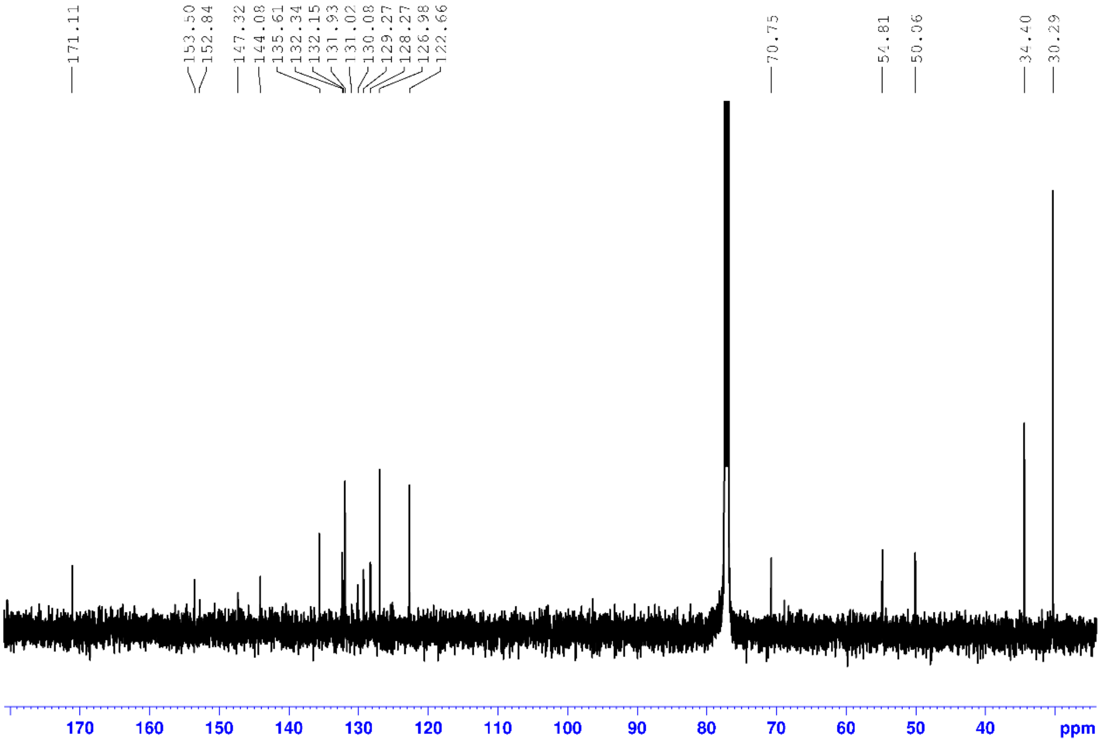

Cyclic trans product ***6t***$^{\textit{trans}}$

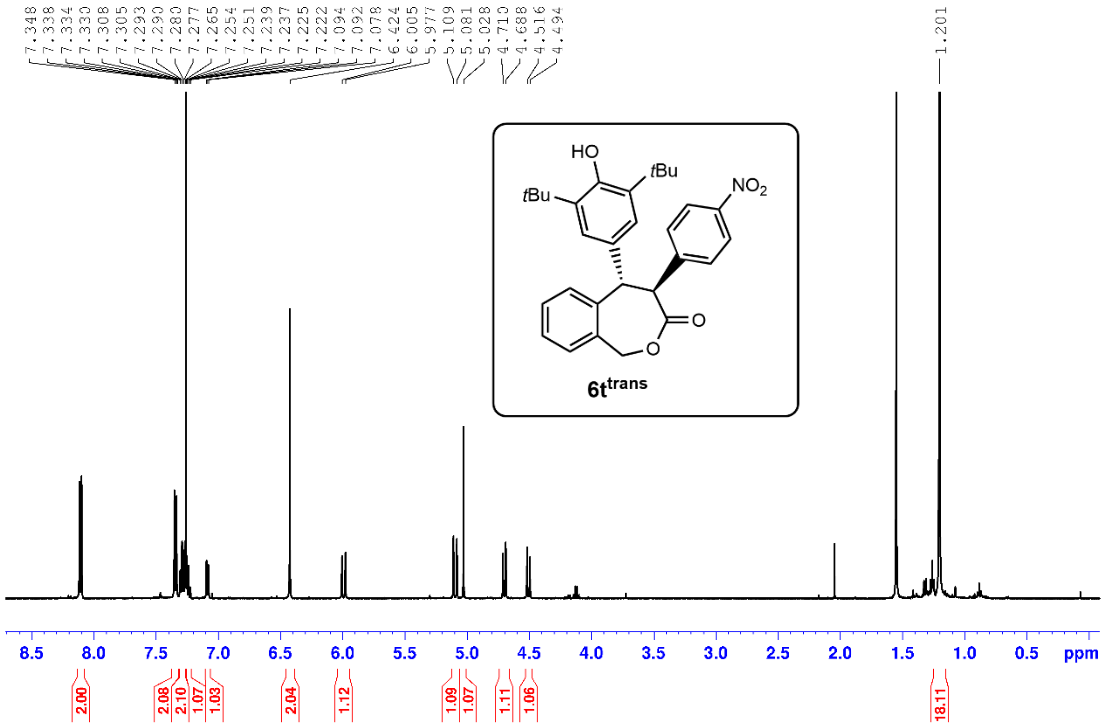

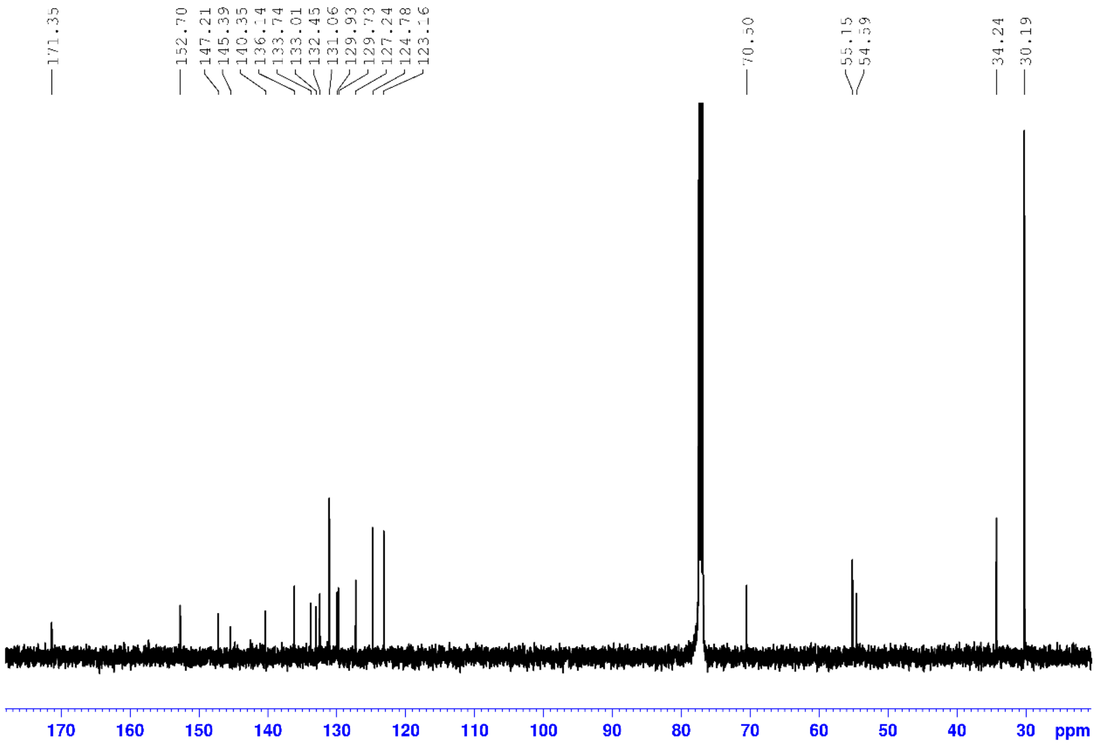

3-Cyanophenyl benzo[c]oxepinone derivative (6u)

Alkylation Product ***5u***

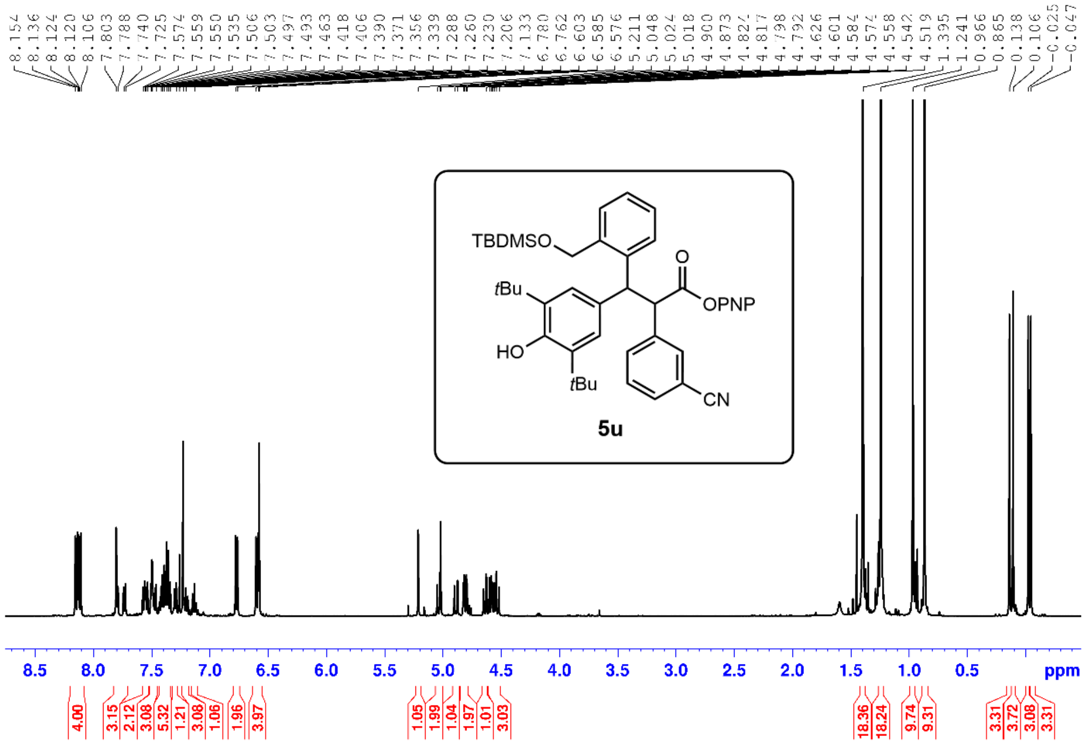

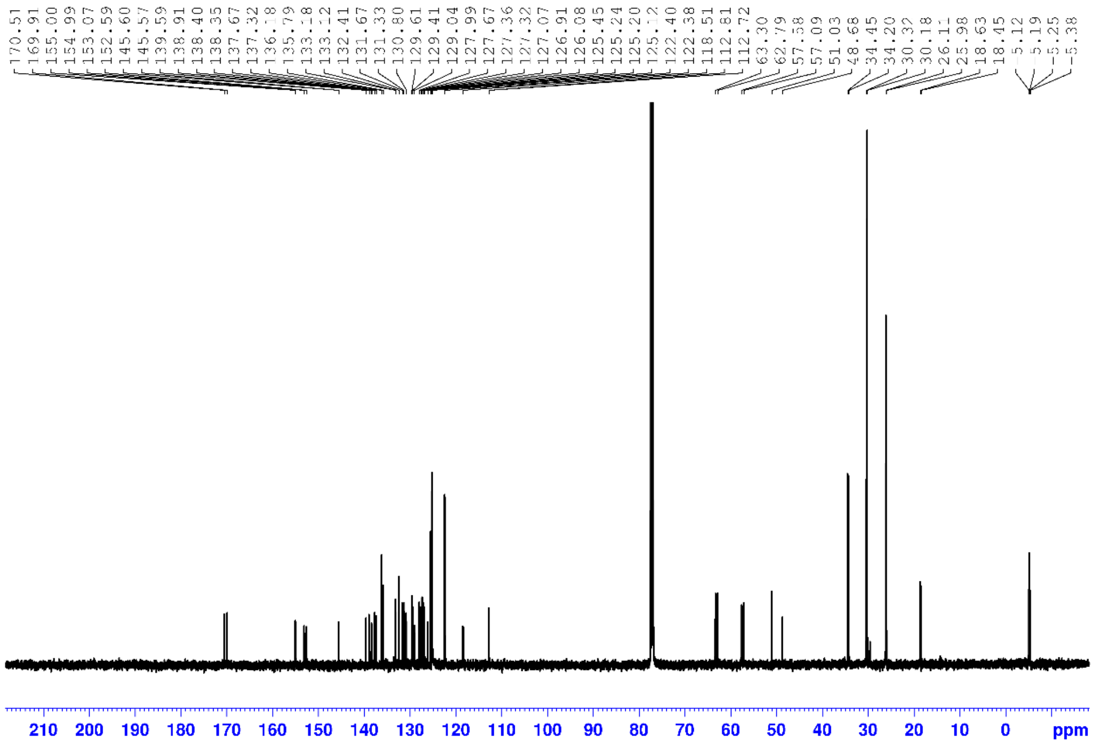

*Cyclic cis product **6u**^{cis}*

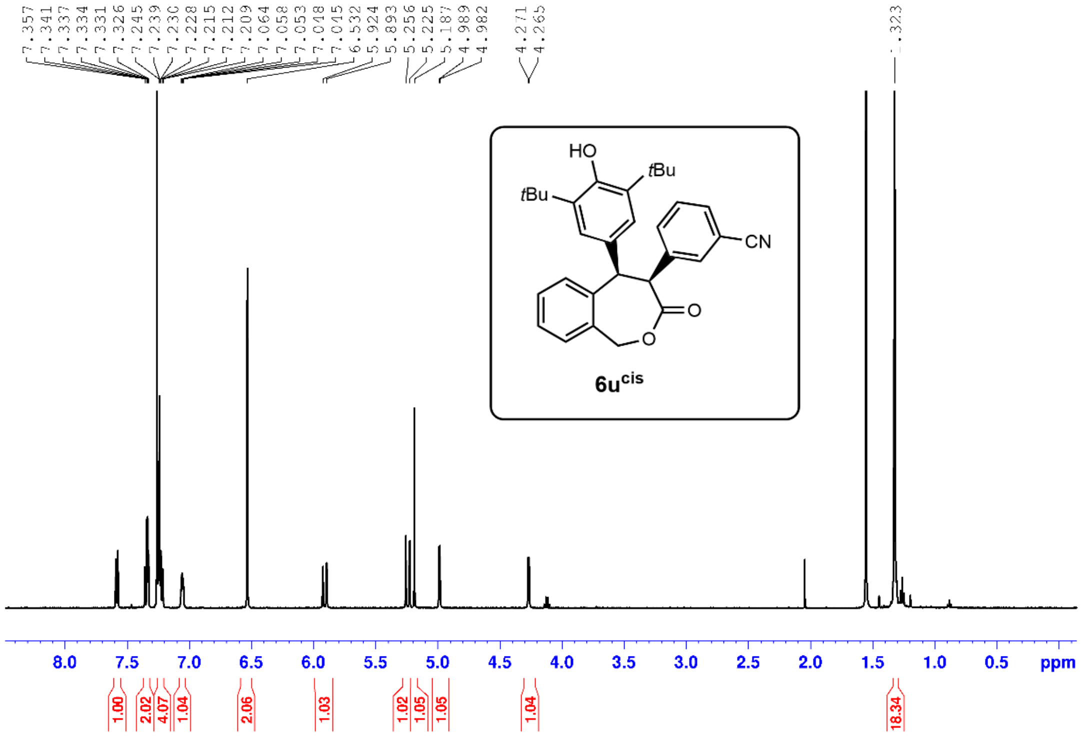

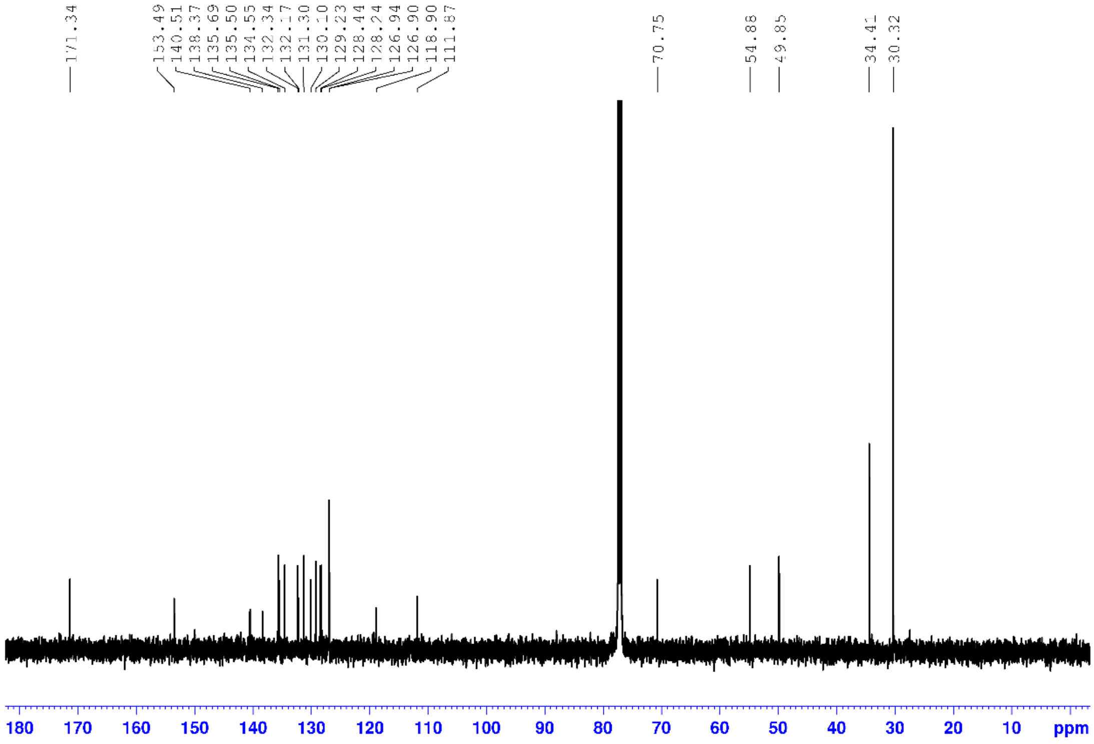

Cyclic trans product ***6u***$^{\textbf{\textit{trans}}}$

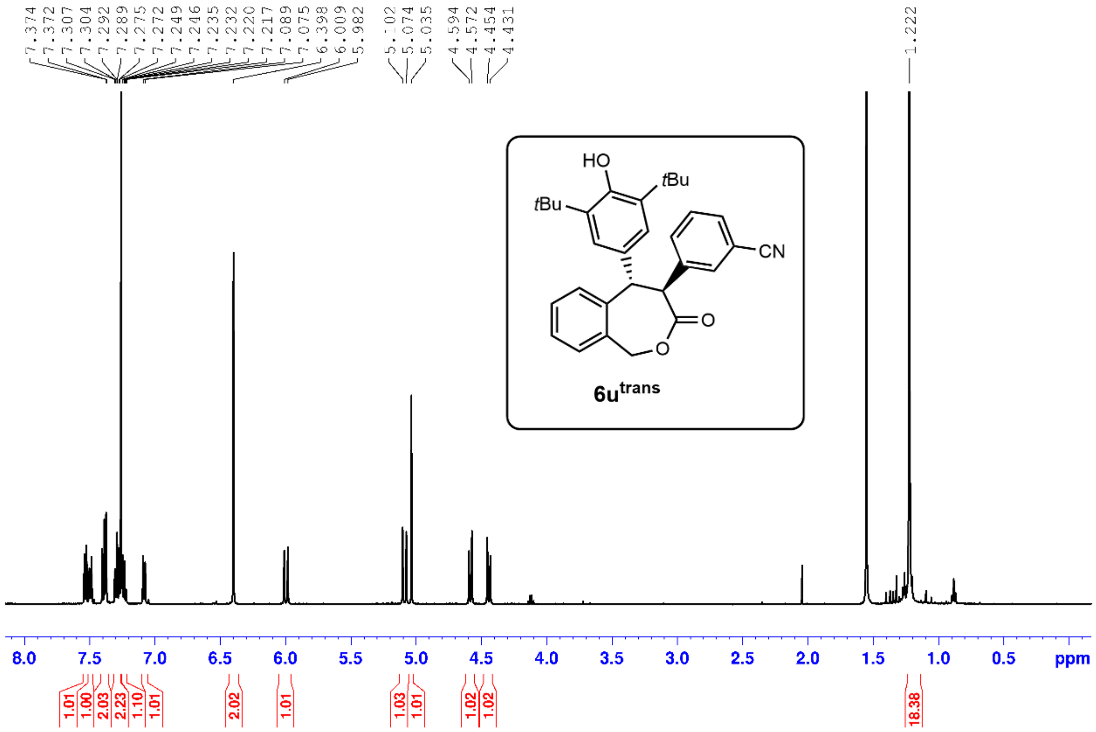

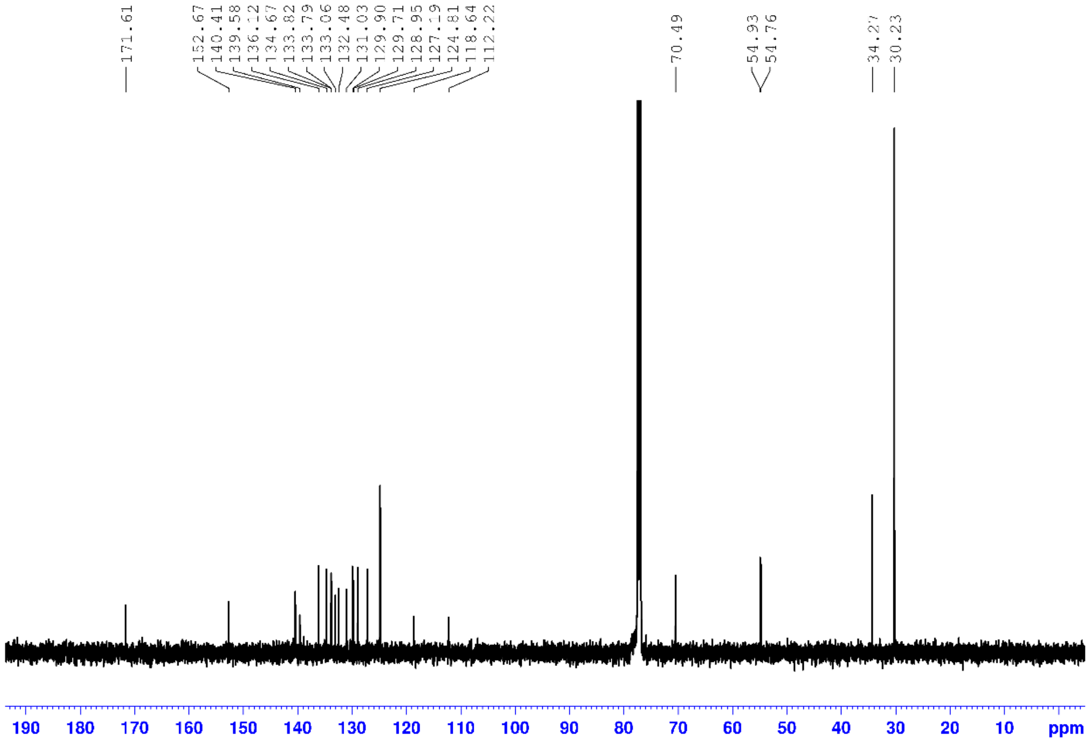

α-Naphthyl benzo[c]oxepinone derivative (6v)

*Alkylation Product **5v***

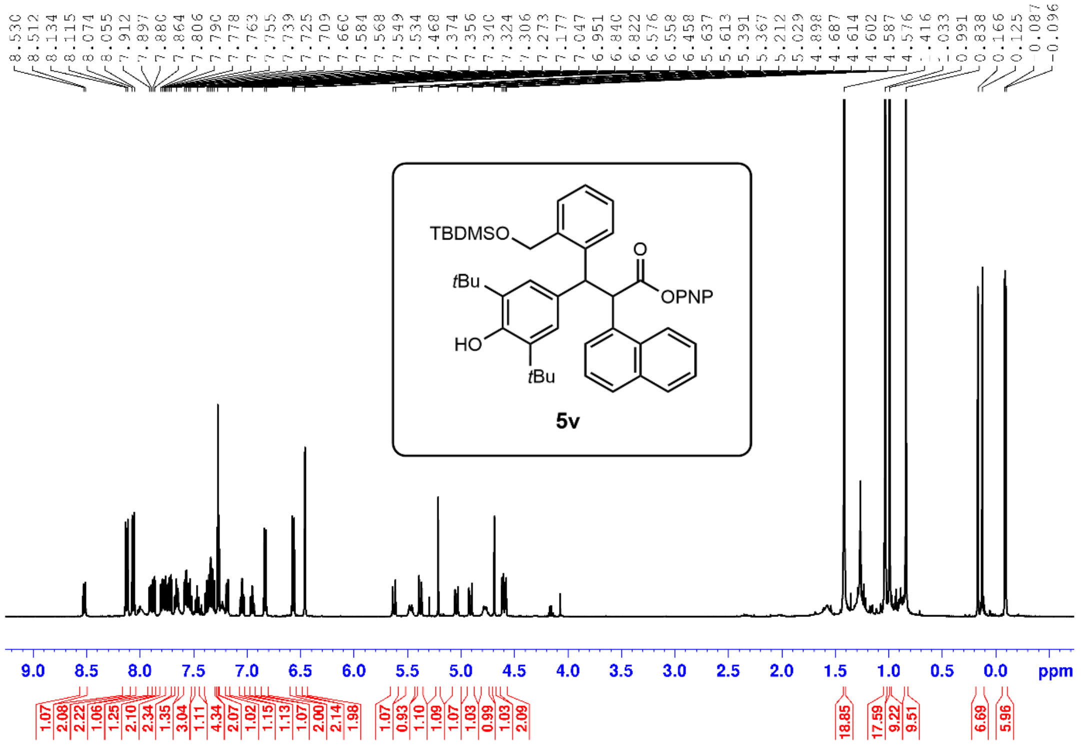

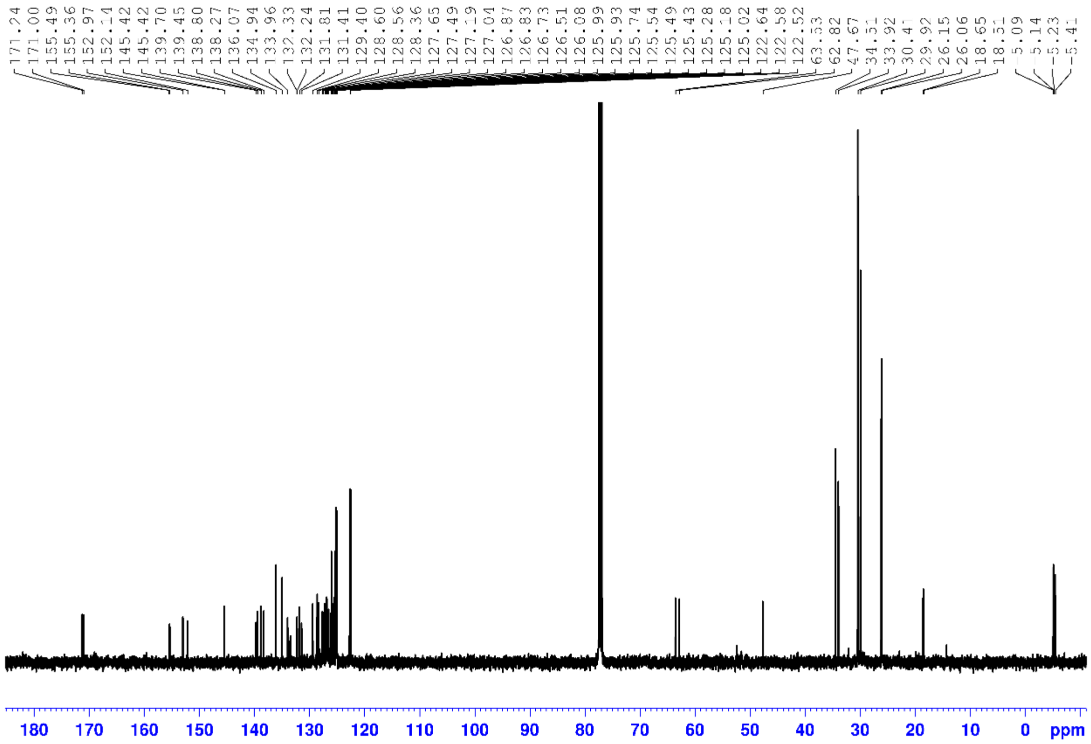

Cyclic products (mixture of diastereomers) ***6v**^{cis}* *and* ***6v**^{trans}*

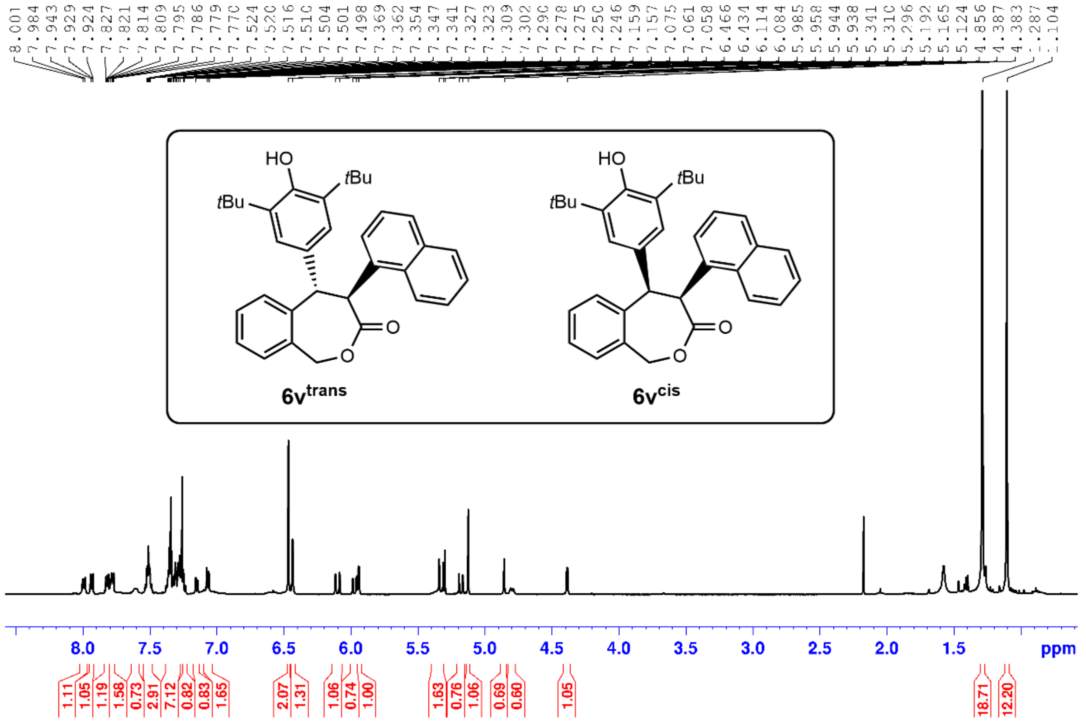

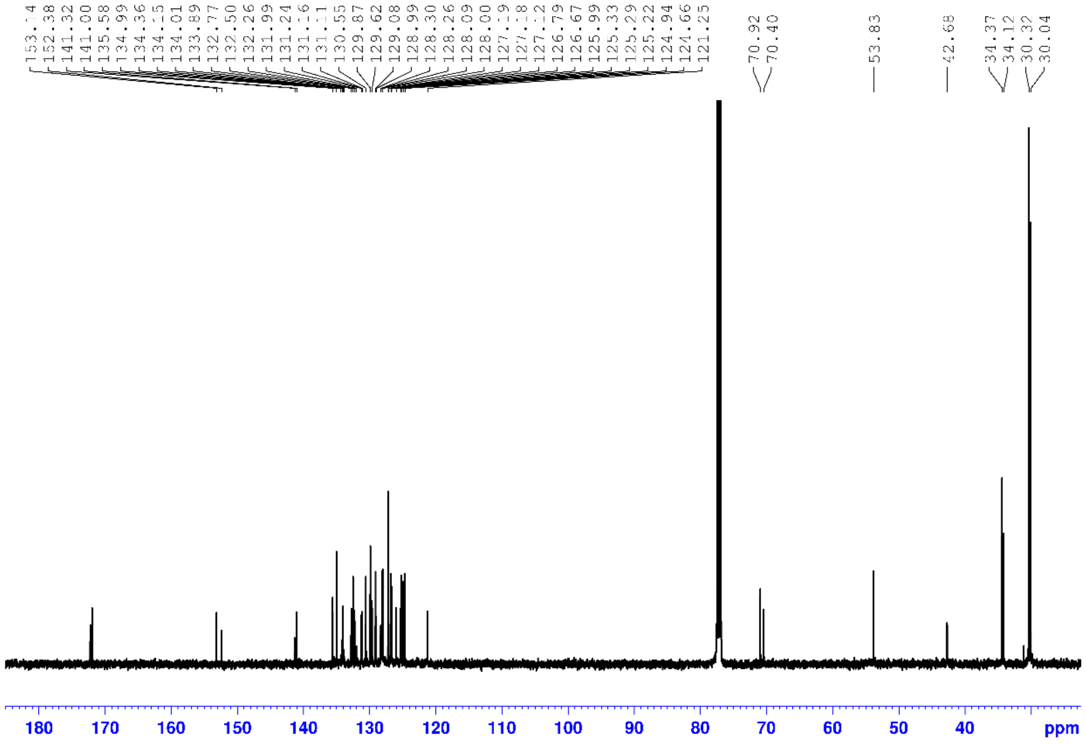

β-Naphthyl benzo[c]oxepinone derivative (6w)

Alkylation Product ***5w***

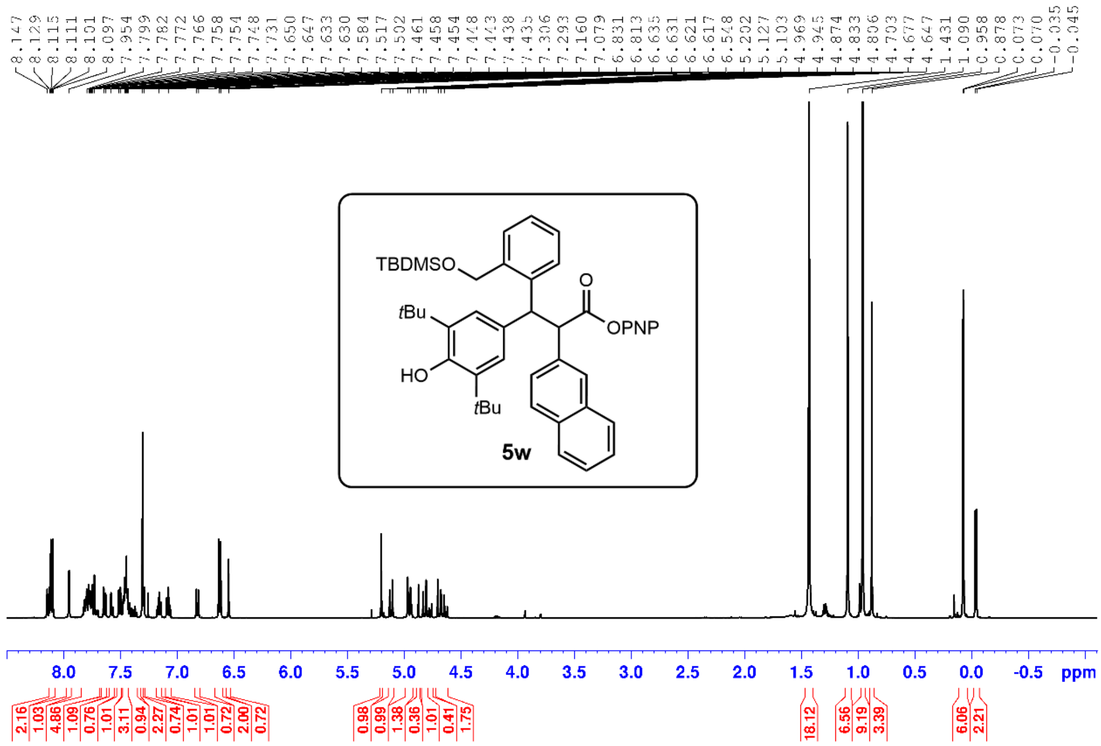

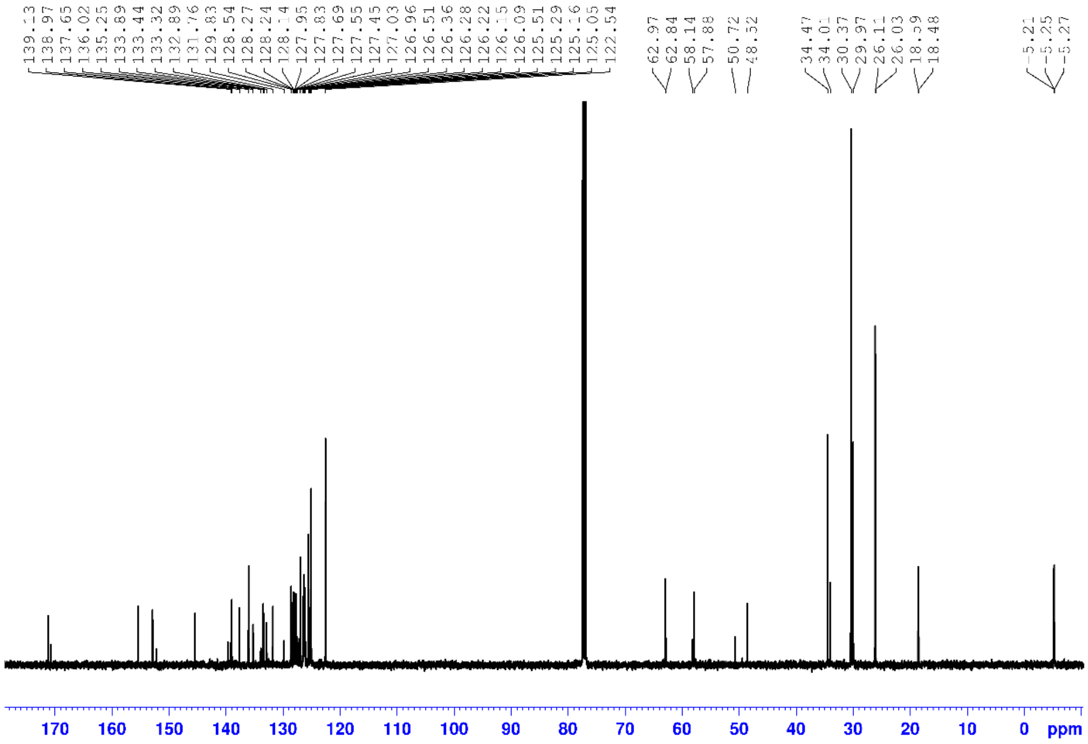

Cyclic cis product ***6w^{cis}***

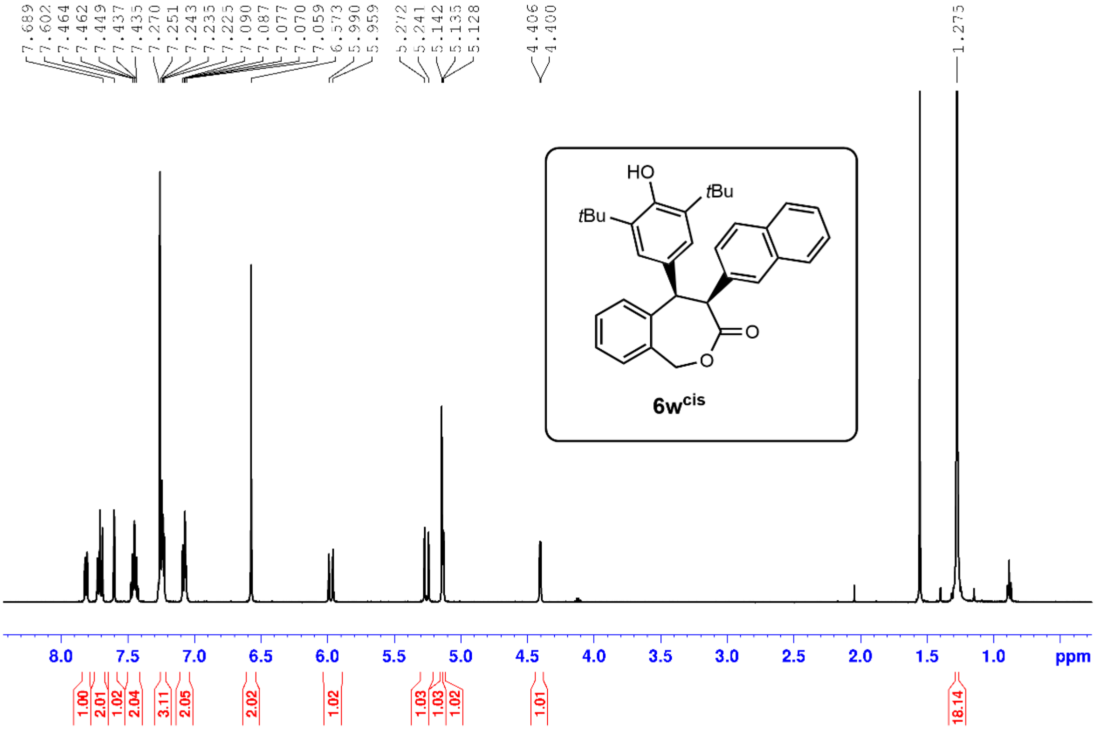

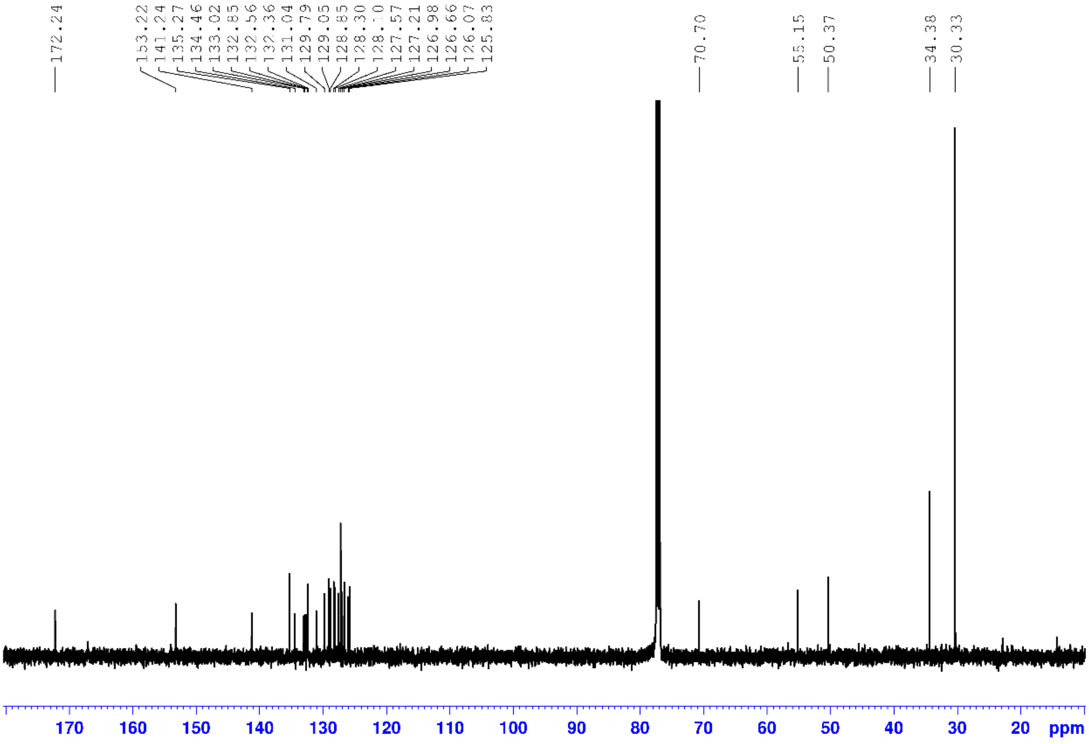

Cyclic trans product ***6w****trans*

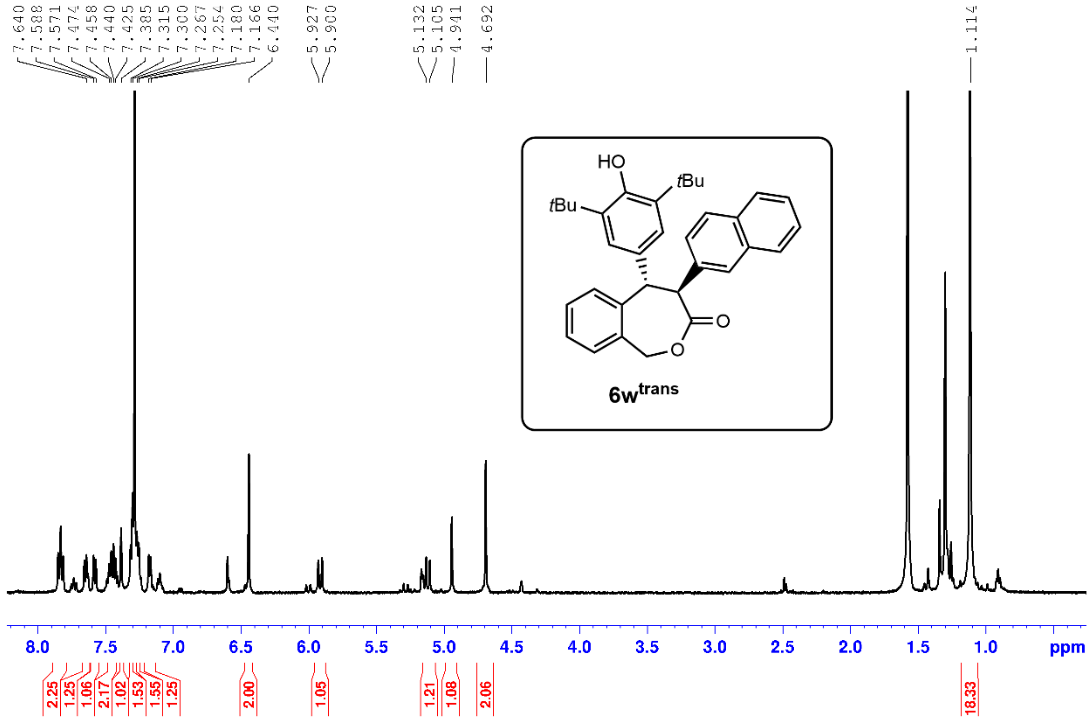

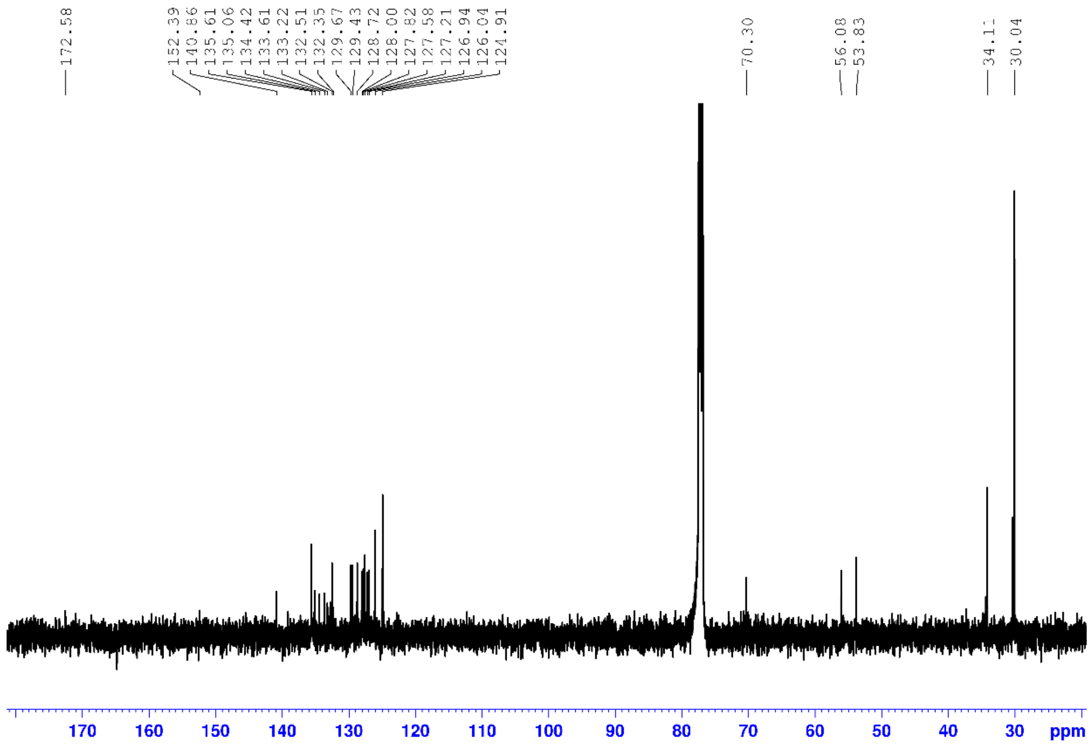

Thiophen-3-yl benzo[c]oxepinone derivative (6x)

Alkylation Product ***5x***

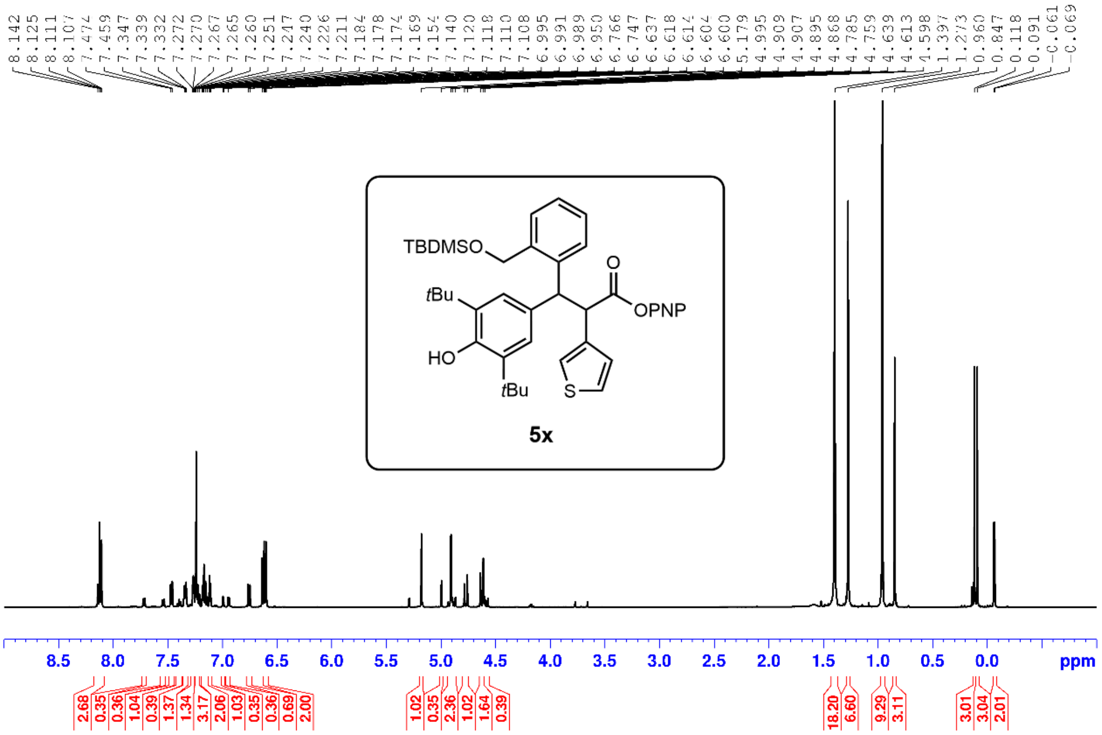

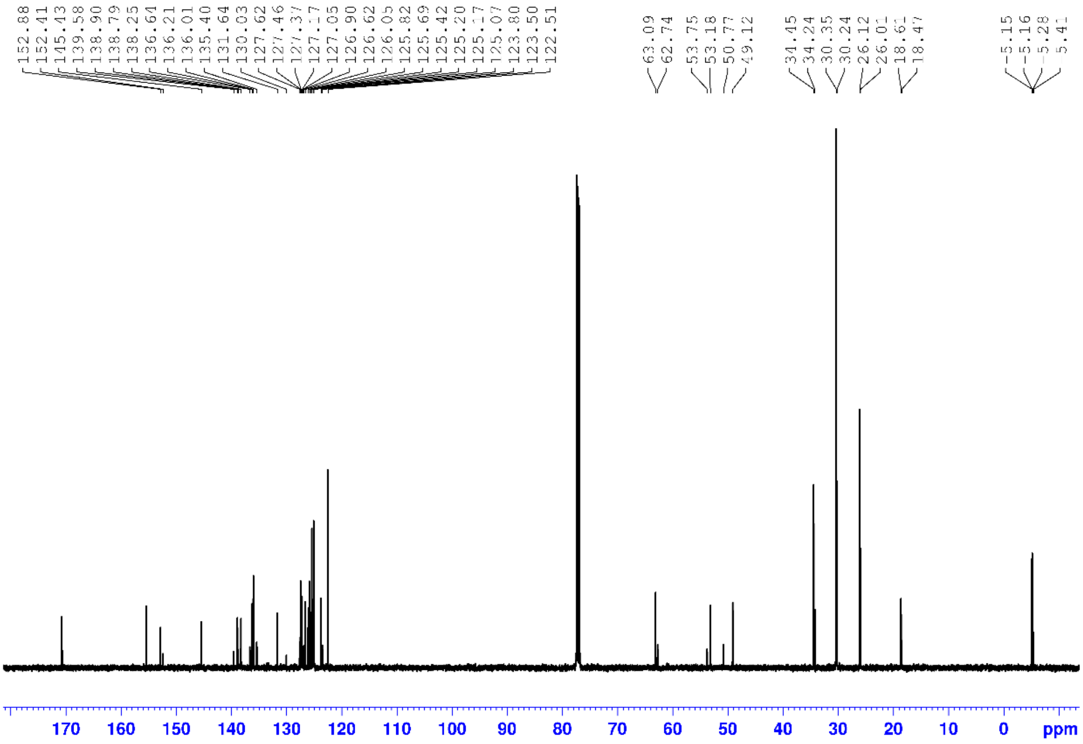

Cyclic cis product ***6x***$^{\textit{cis}}$

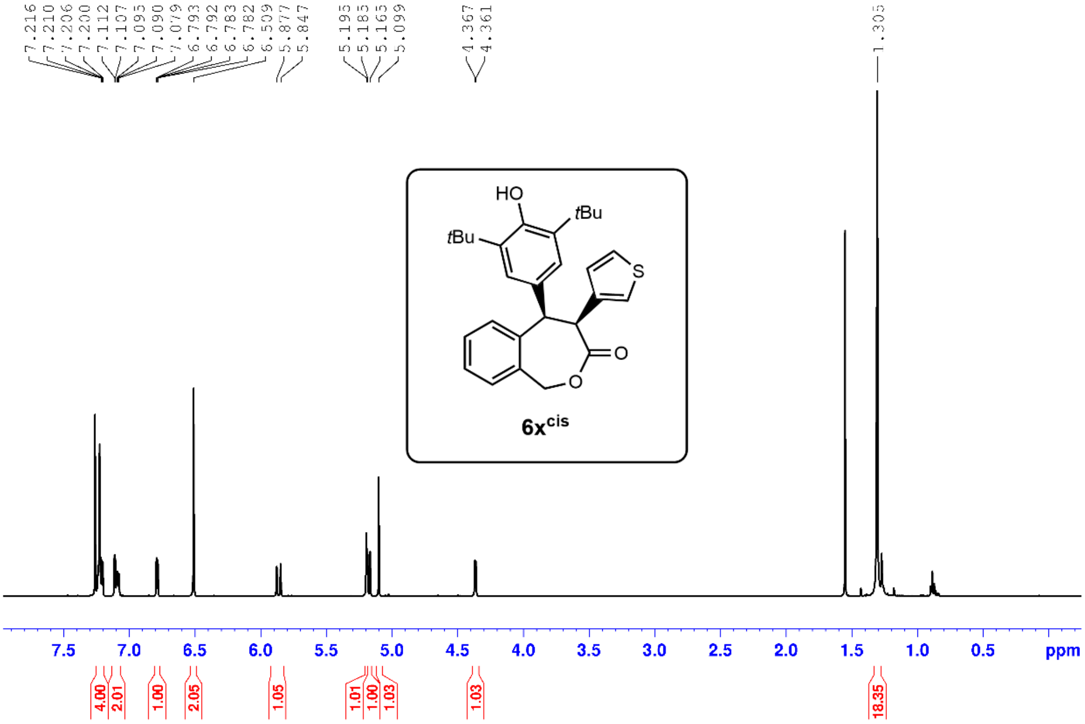

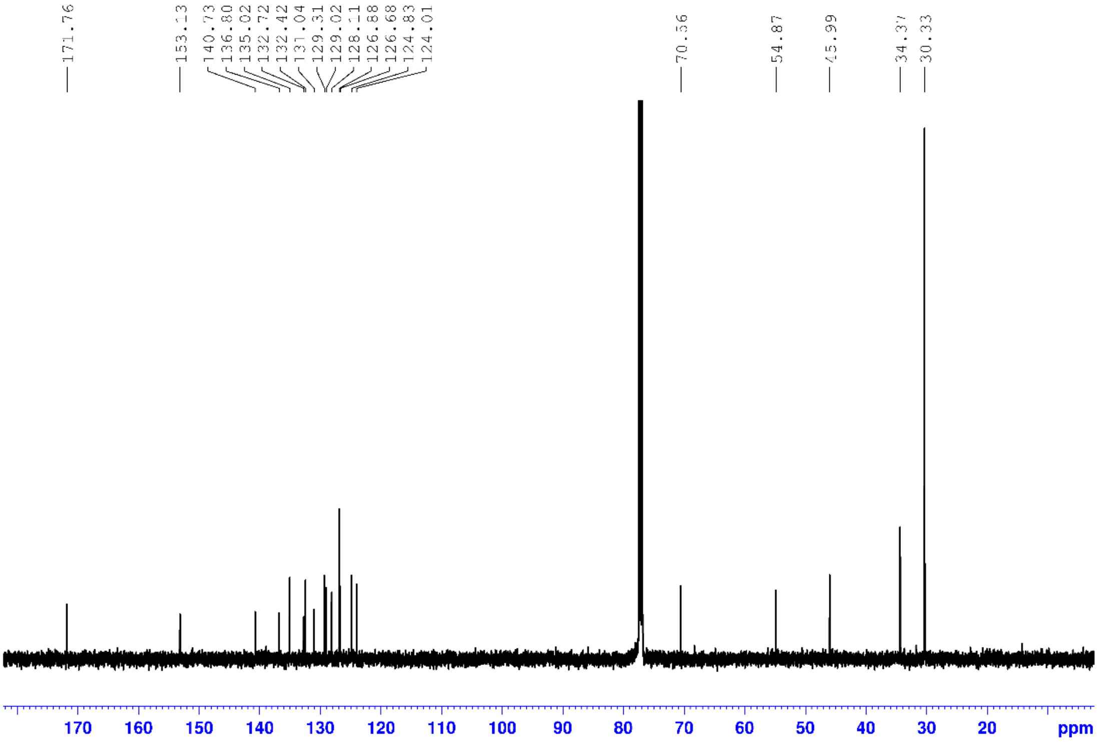

*Cyclic trans product **6x**^trans^*

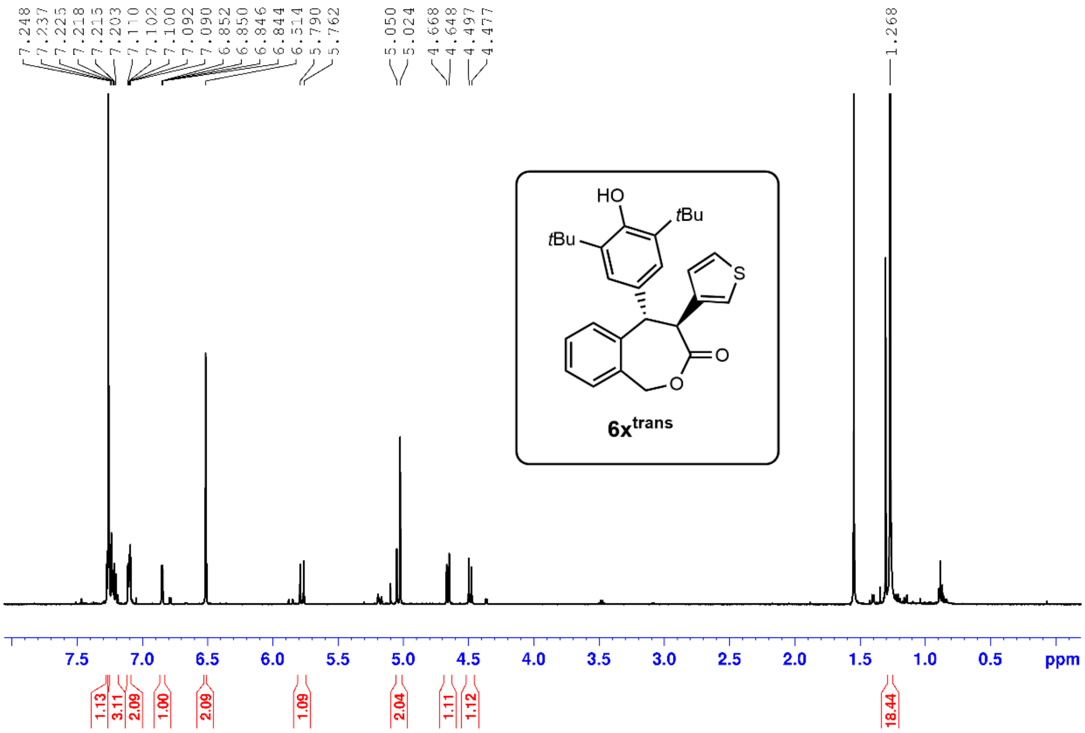

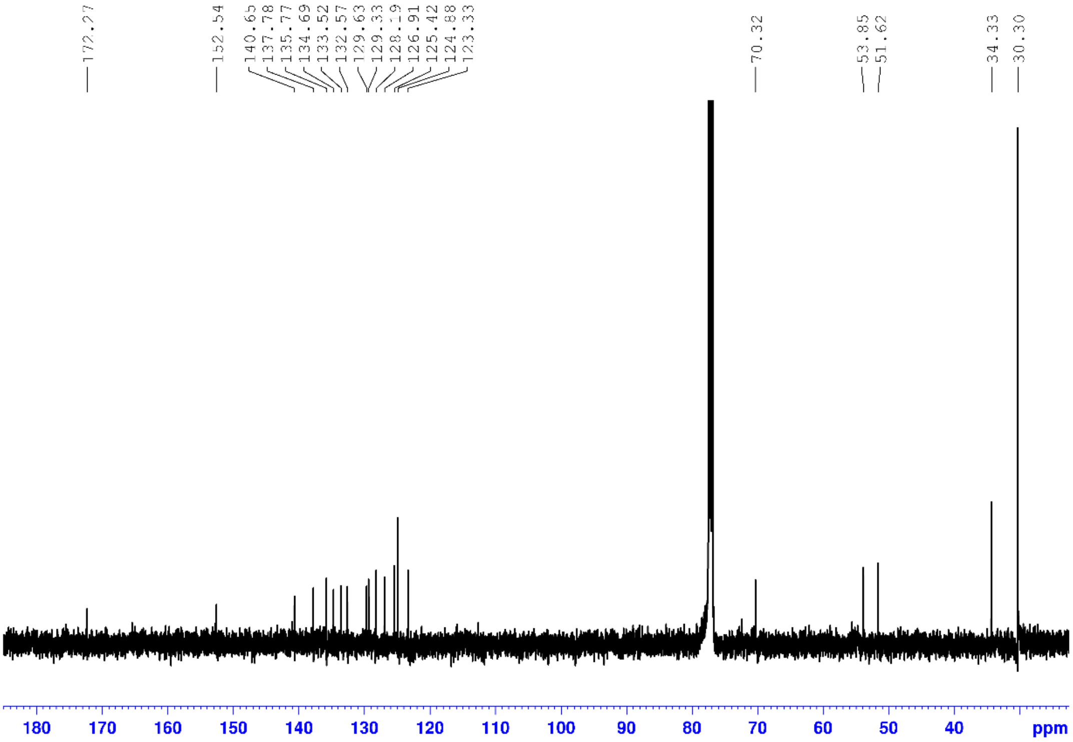

7-Fluoro benzo[c]oxepinone derivative (6aa)

*Alkylation Product **5aa***

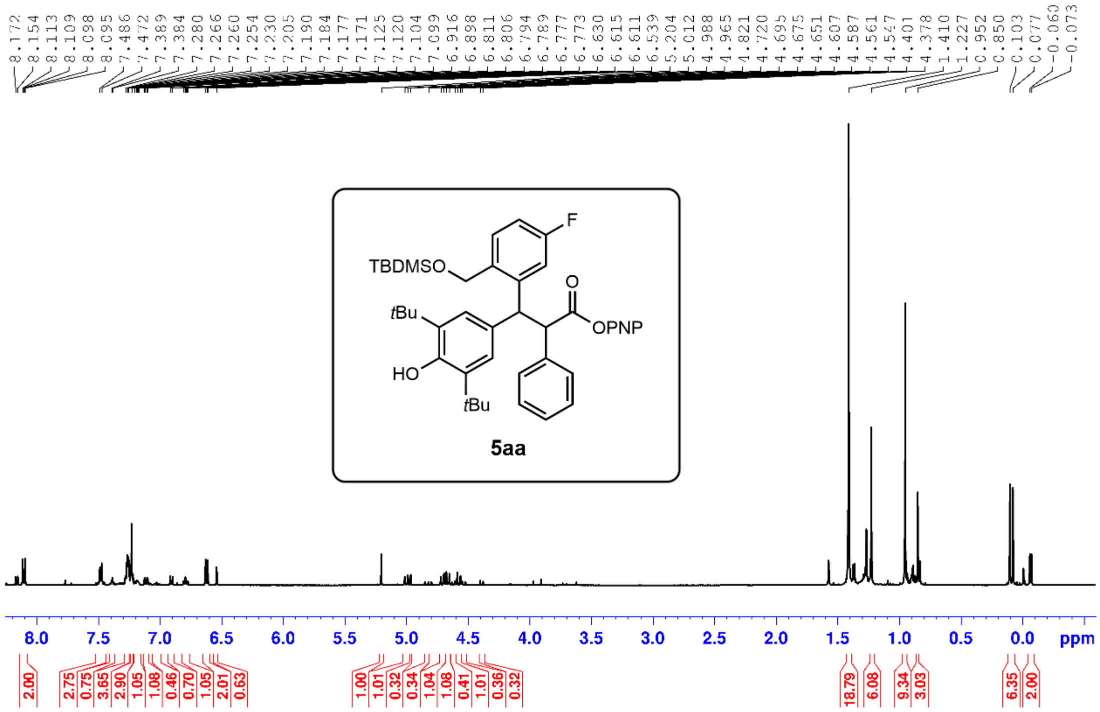

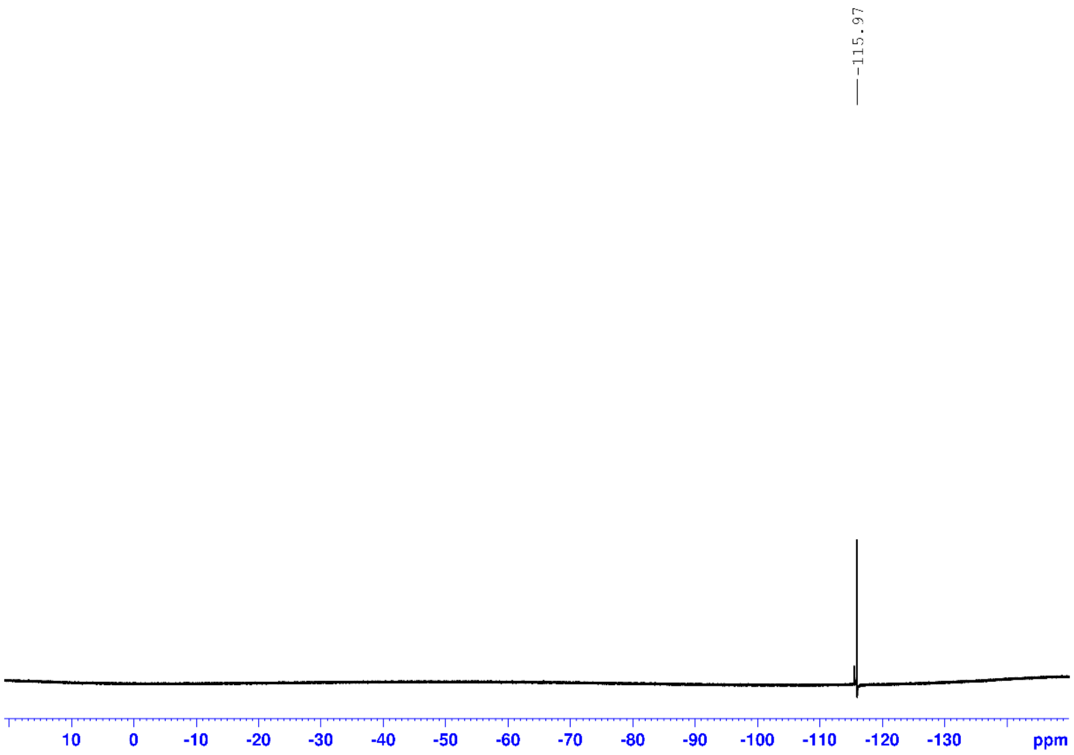

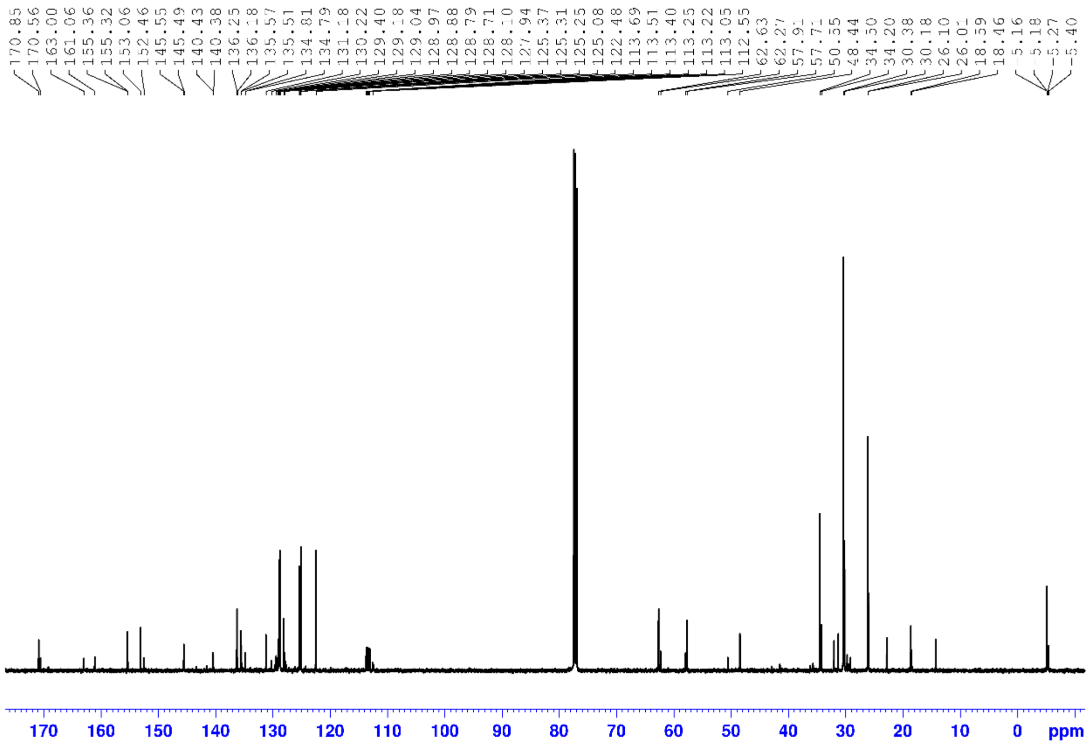
170 160 150 140 130 120 110 100 90 80 70 60 50 40 30 20 10 0 ppm

*Cyclic cis product **6aa**^{cis}*

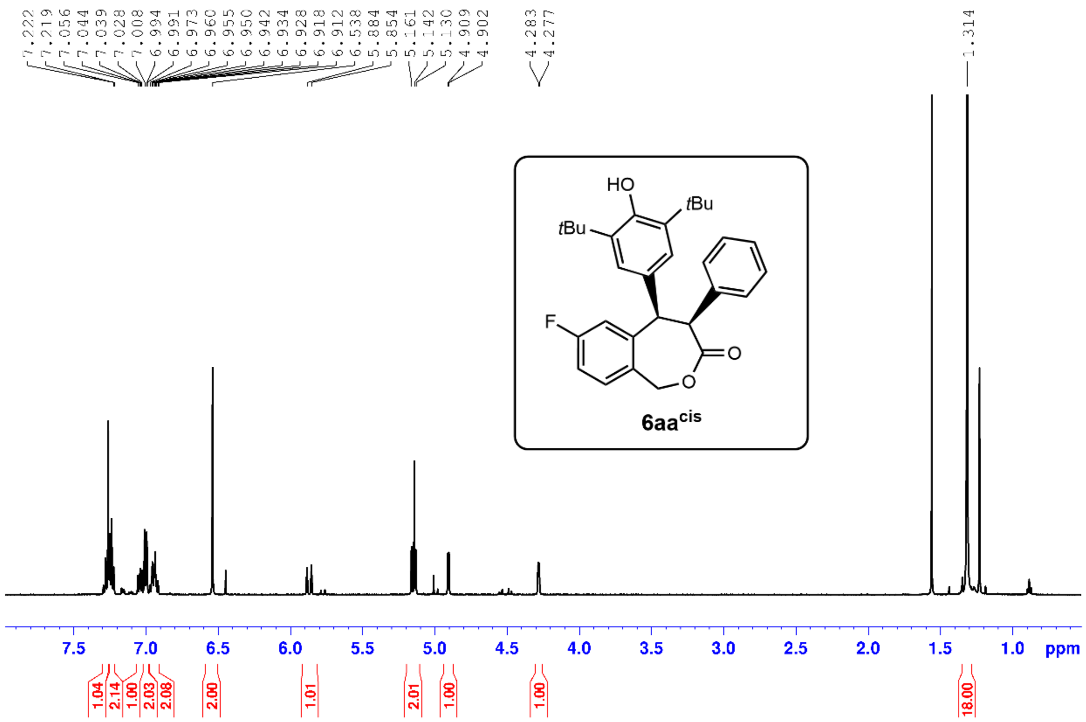

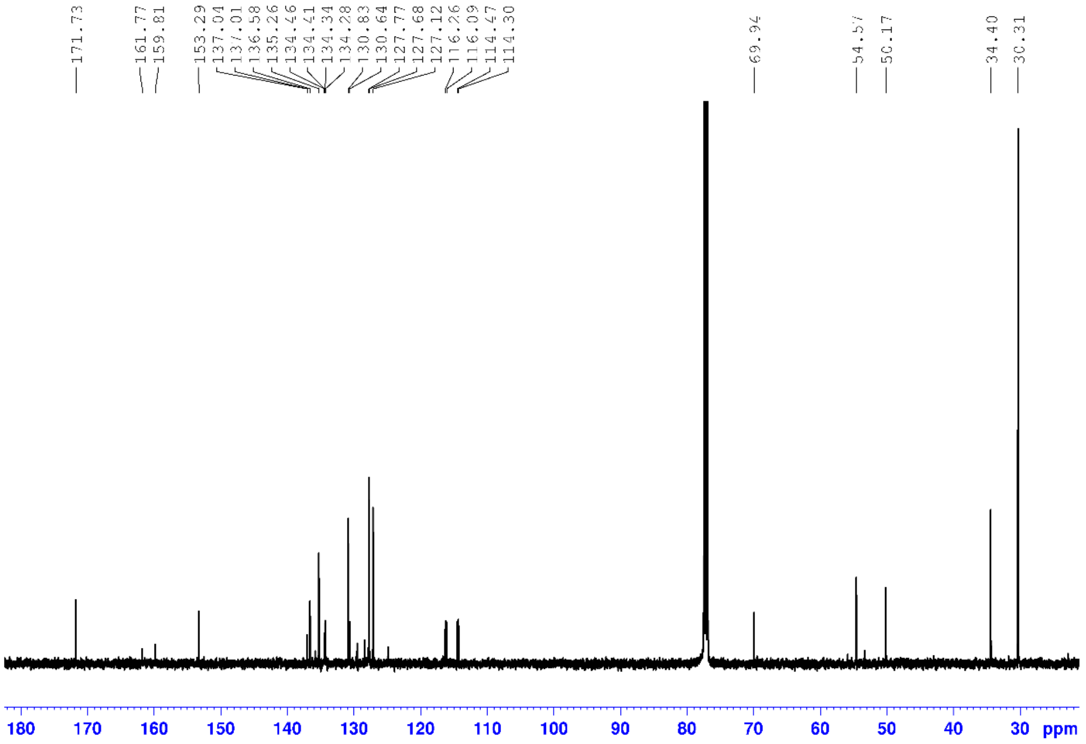

Cyclic trans productr ***6aa***$^{\textit{trans}}$ *(containing the cis as well -C13 only for aliphatic region)*

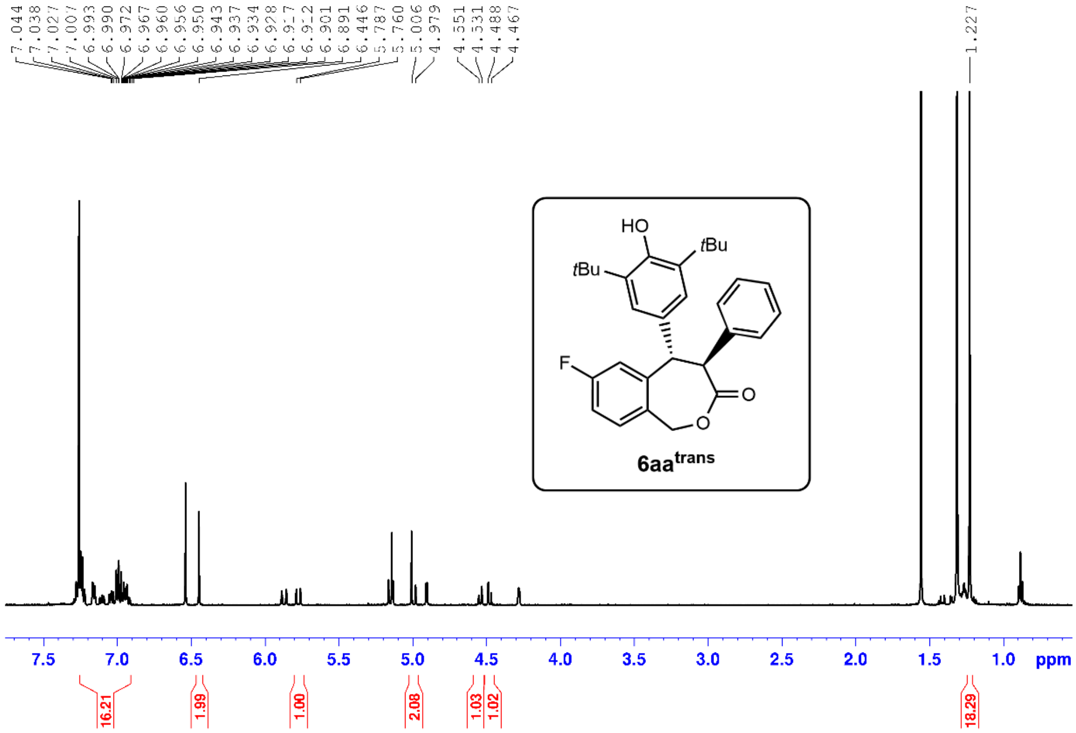

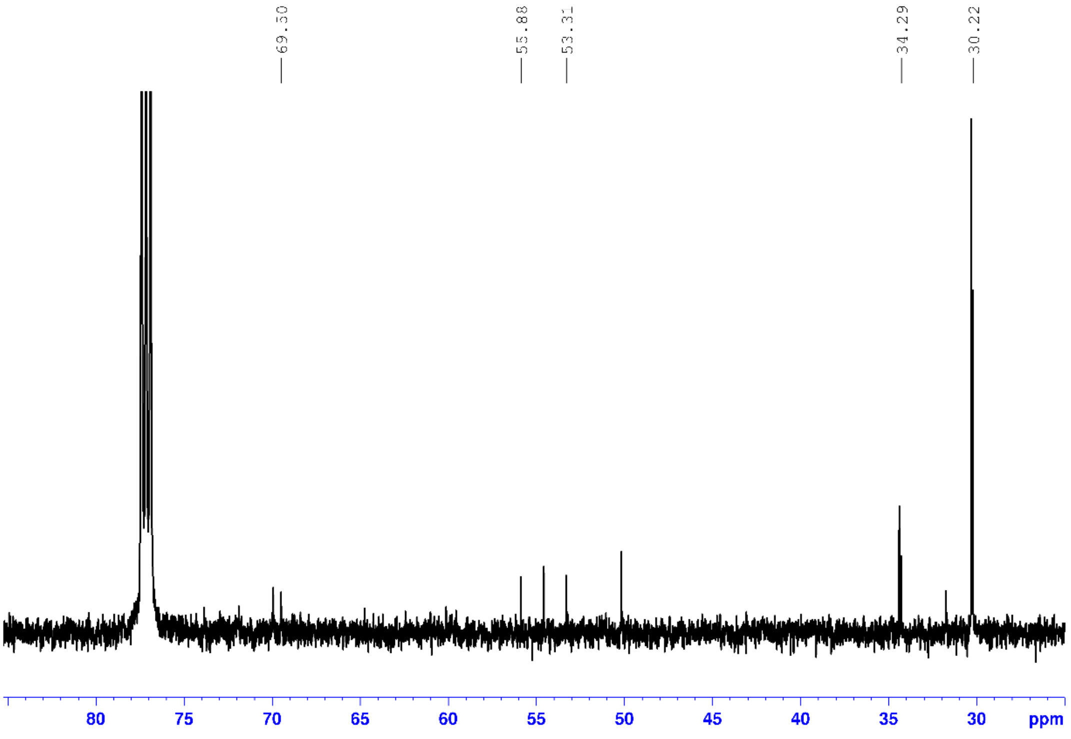

8-Fluoro benzo[c]oxepinone derivative (6ab)

*Alkylation Product **5ab***

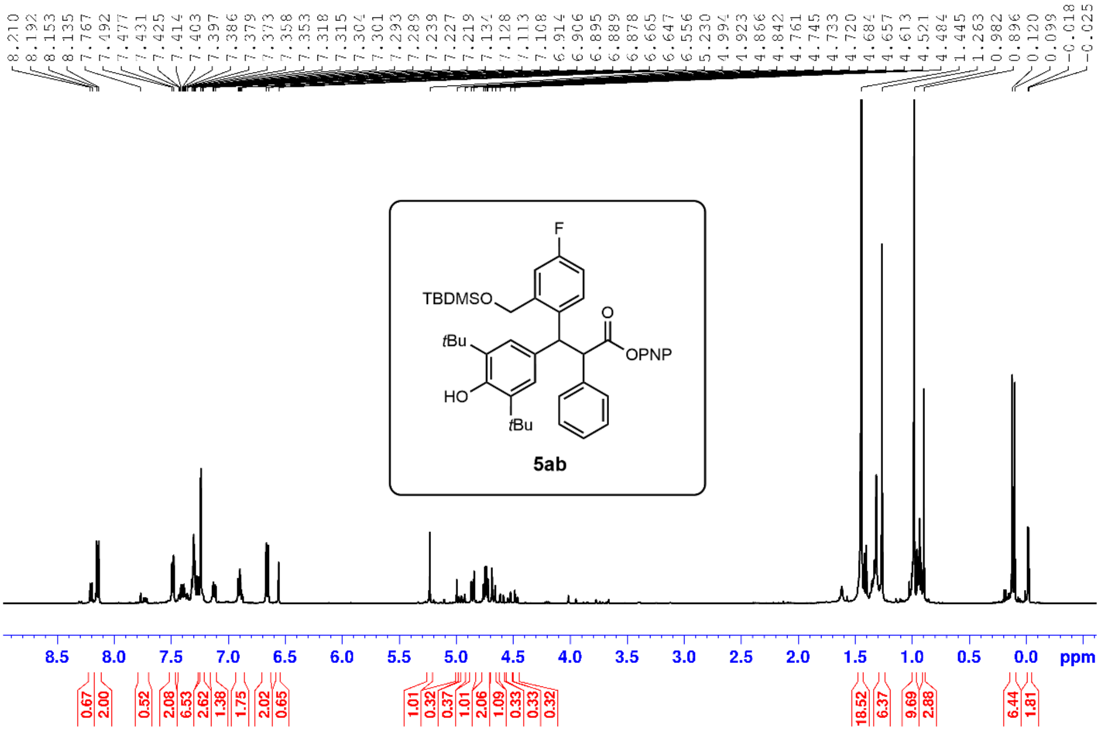

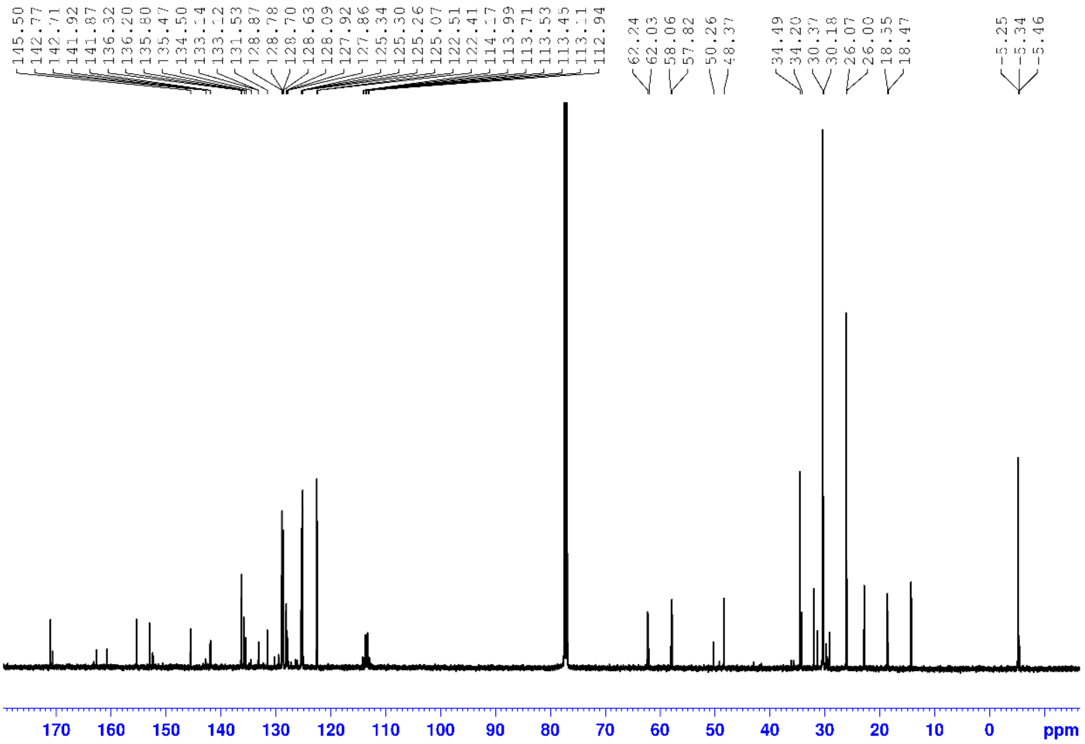

*Cyclic cis product **6ab^{cis}***

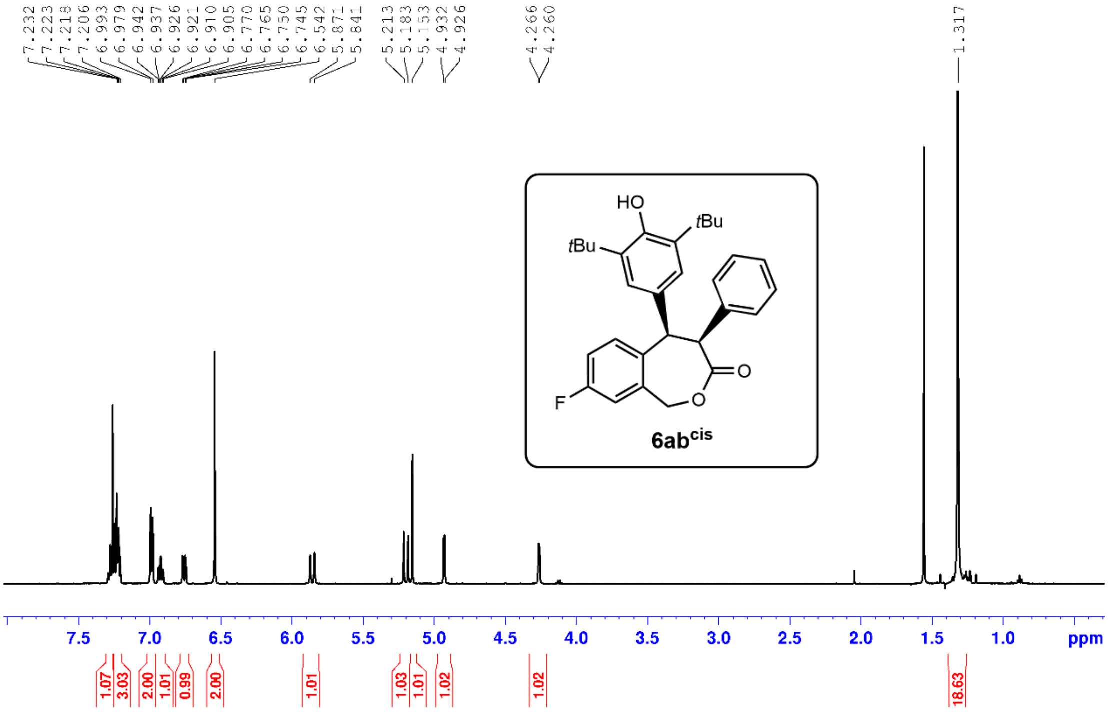

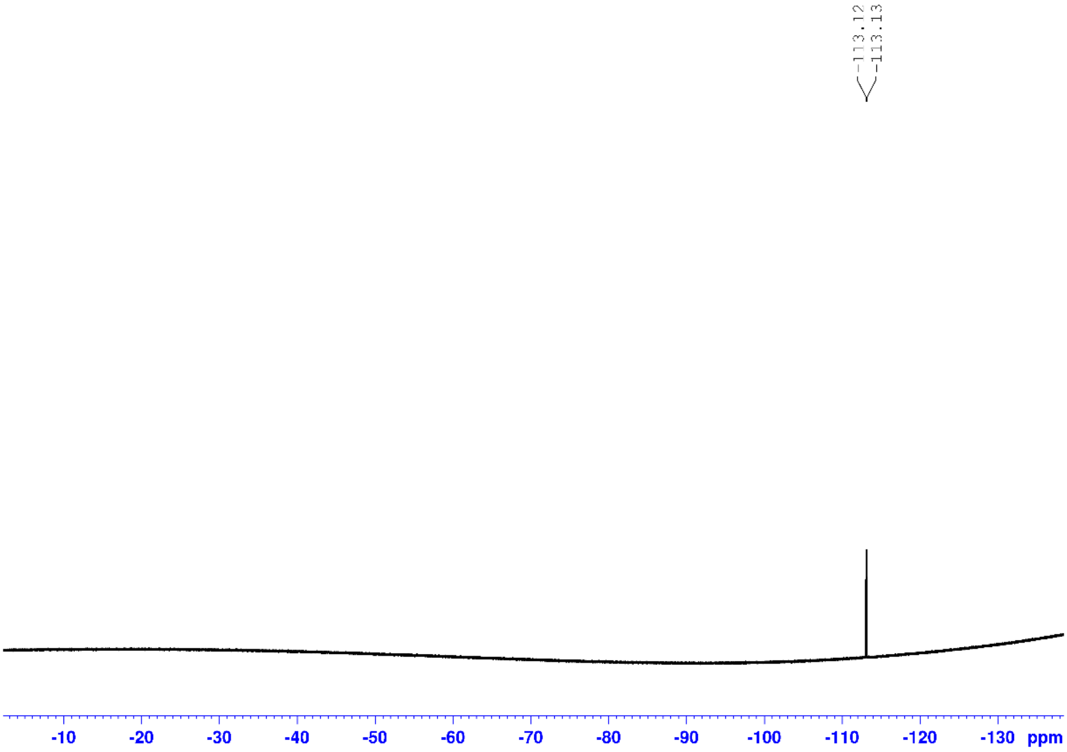

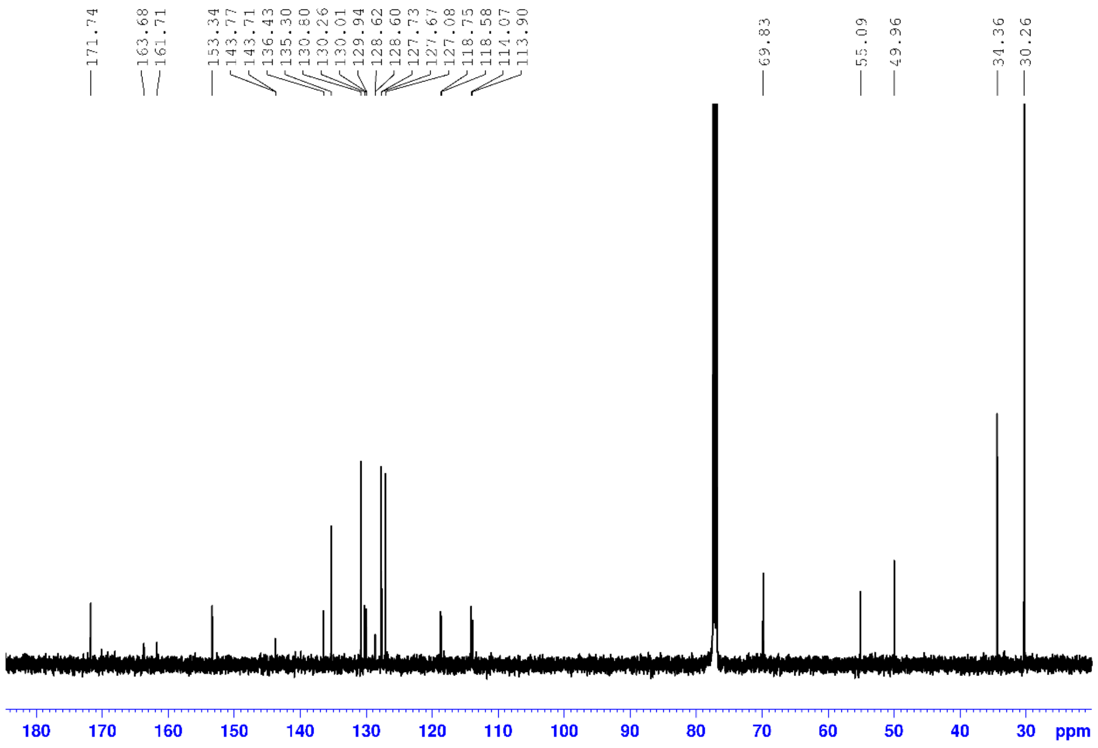
171.74
163.68
161.71
153.34
143.77
143.71
136.43
135.30
130.80
130.26
130.01
129.94
128.62
128.60
127.73
127.67
127.08
118.75
118.58
114.07
113.90
69.83
55.09
49.96
34.36
30.26
180
170
160
150
140
130
120
110
100
90
80
70
60
50
40
30
ppm

Cyclic trans product ***6ab***$^{\textbf{\textit{trans}}}$ *(contains the cis as well)*

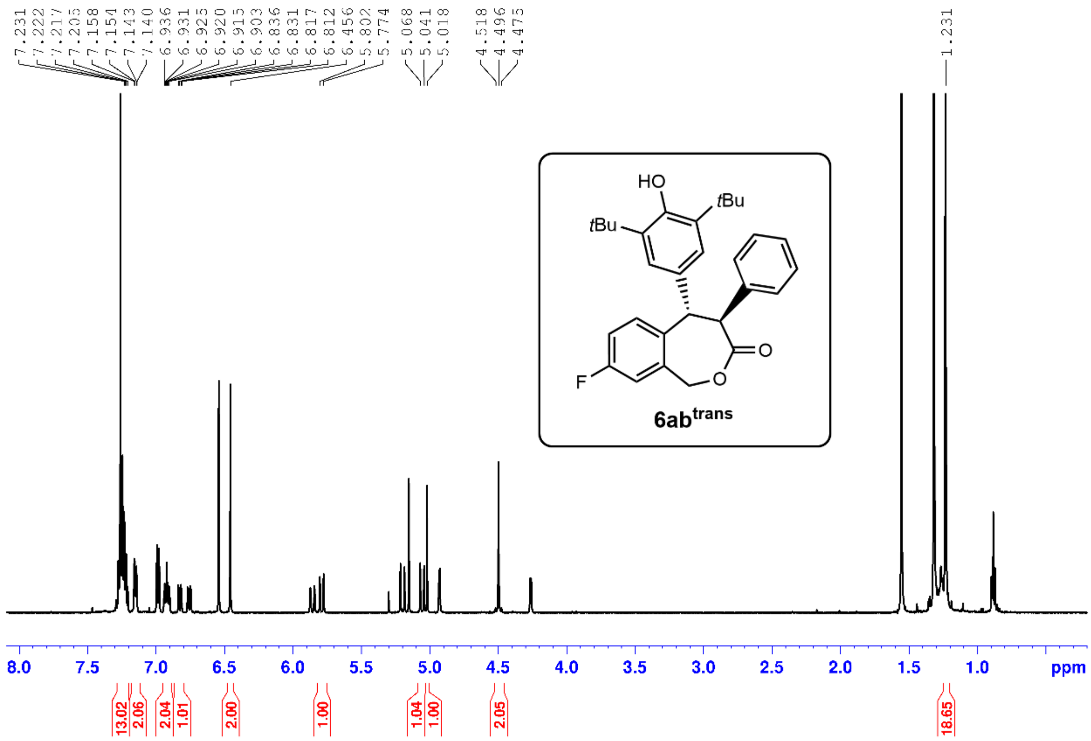

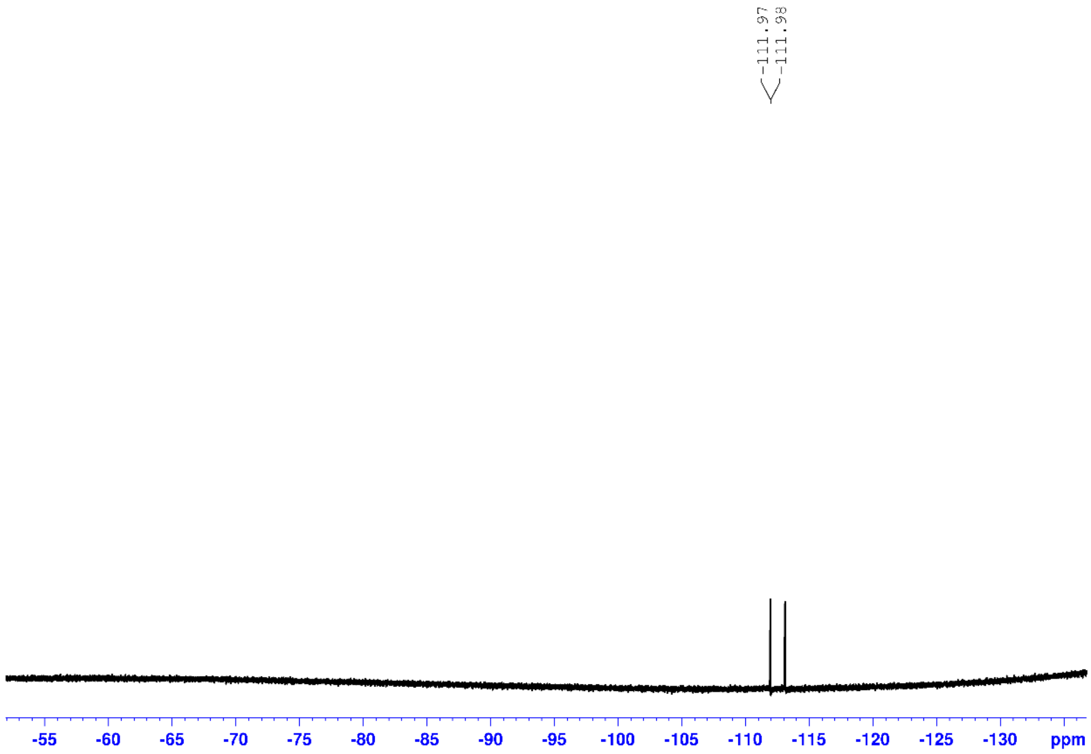

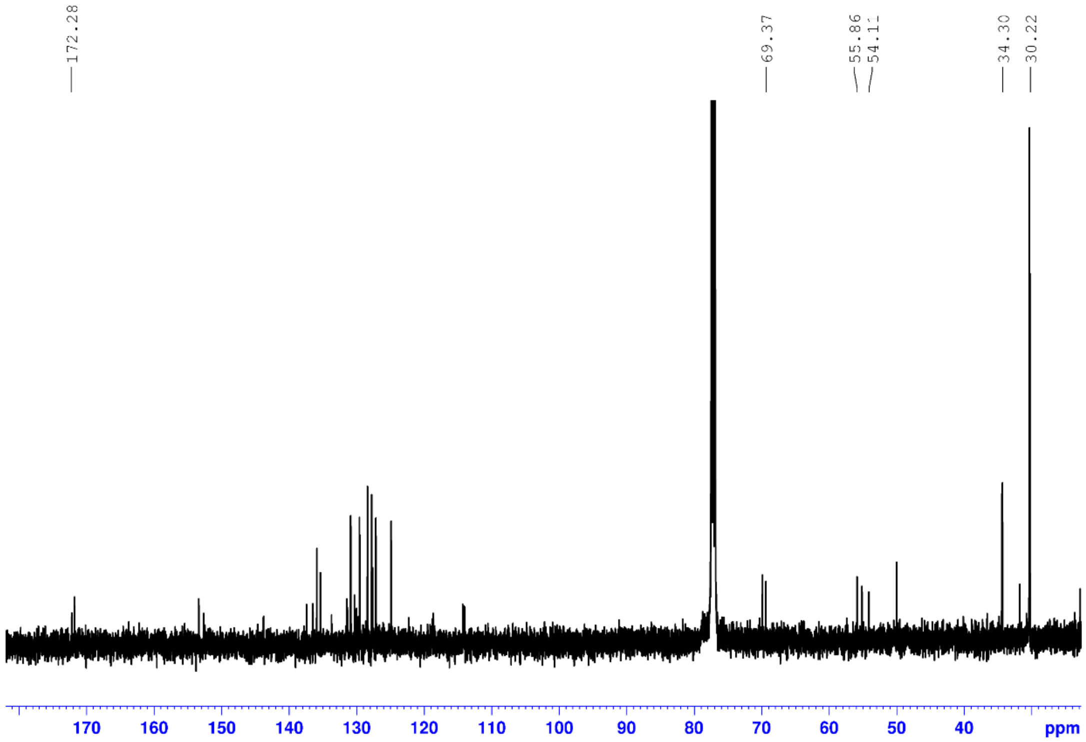

172.28
69.37
55.86
54.11
34.30
30.22
170
160
150
140
130
120
110
100
90
80
70
60
50
40
ppm

7-Chloro benzo[c]oxepinone derivative (6ba)

Alkylation Product ***5ba***

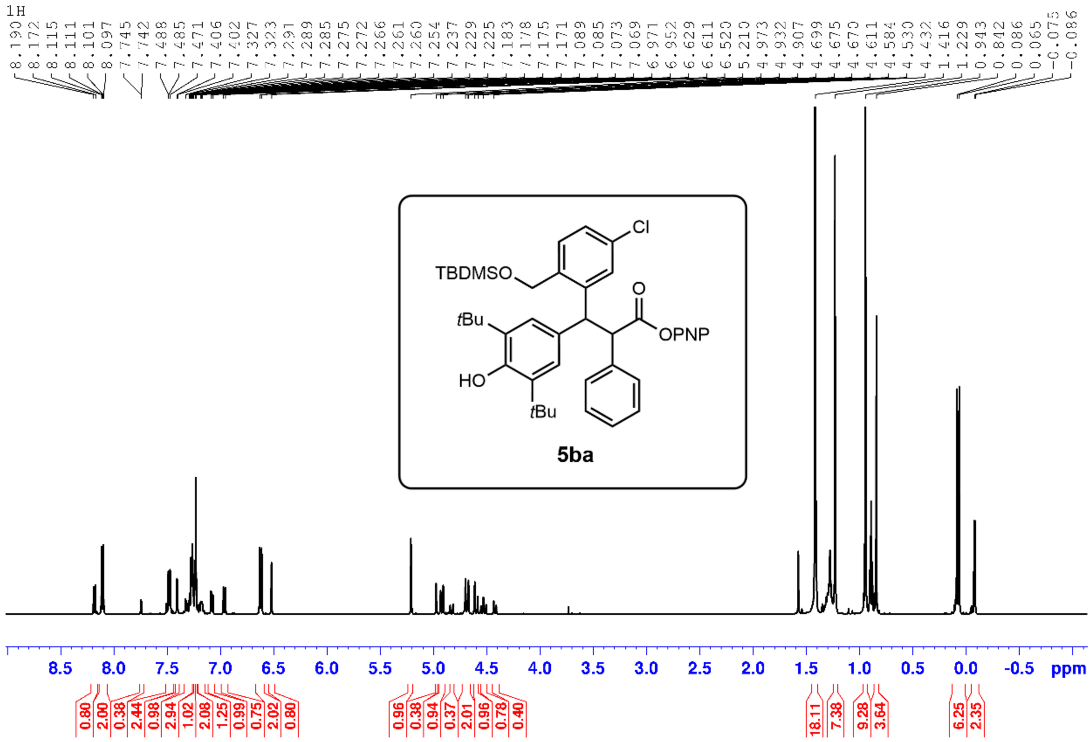

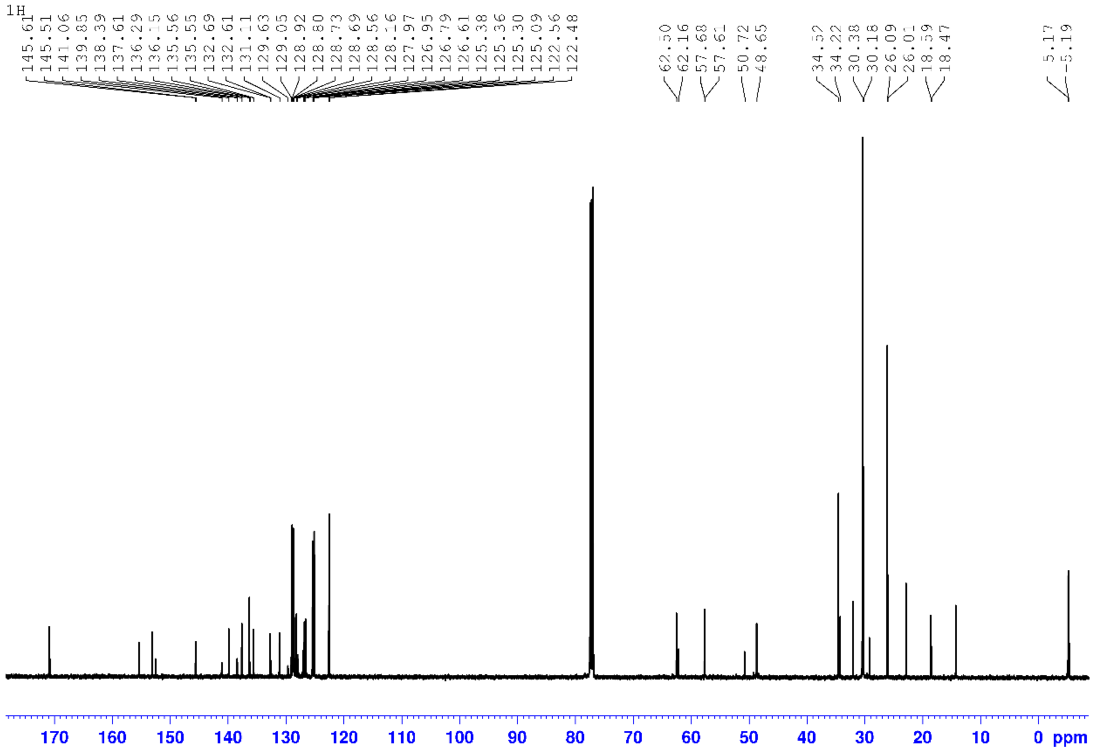

Cyclic cis product ***6ba***^{cis}

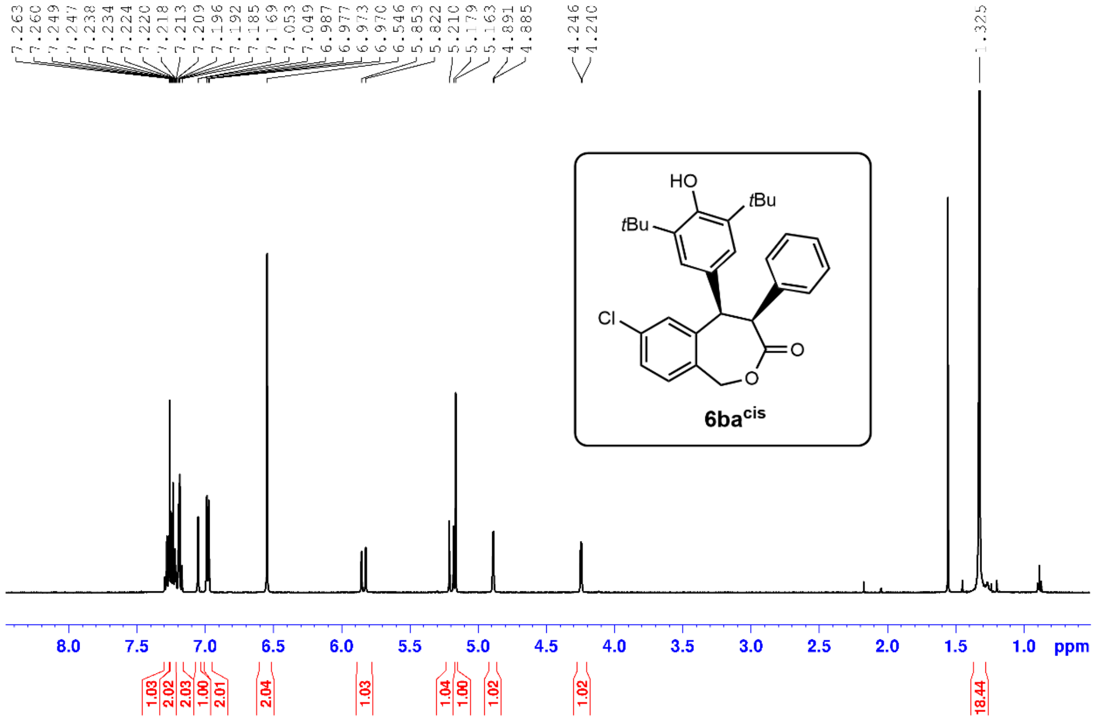

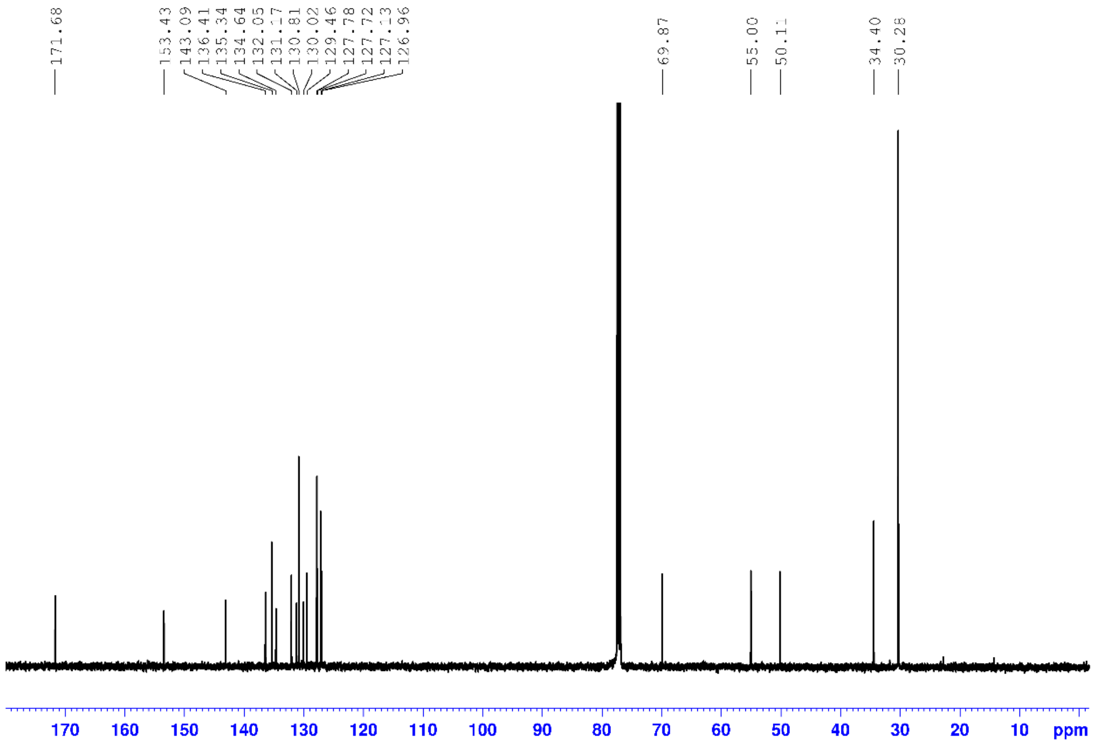

Cyclic trans product ***6ba***$^{\textbf{\textit{trans}}}$

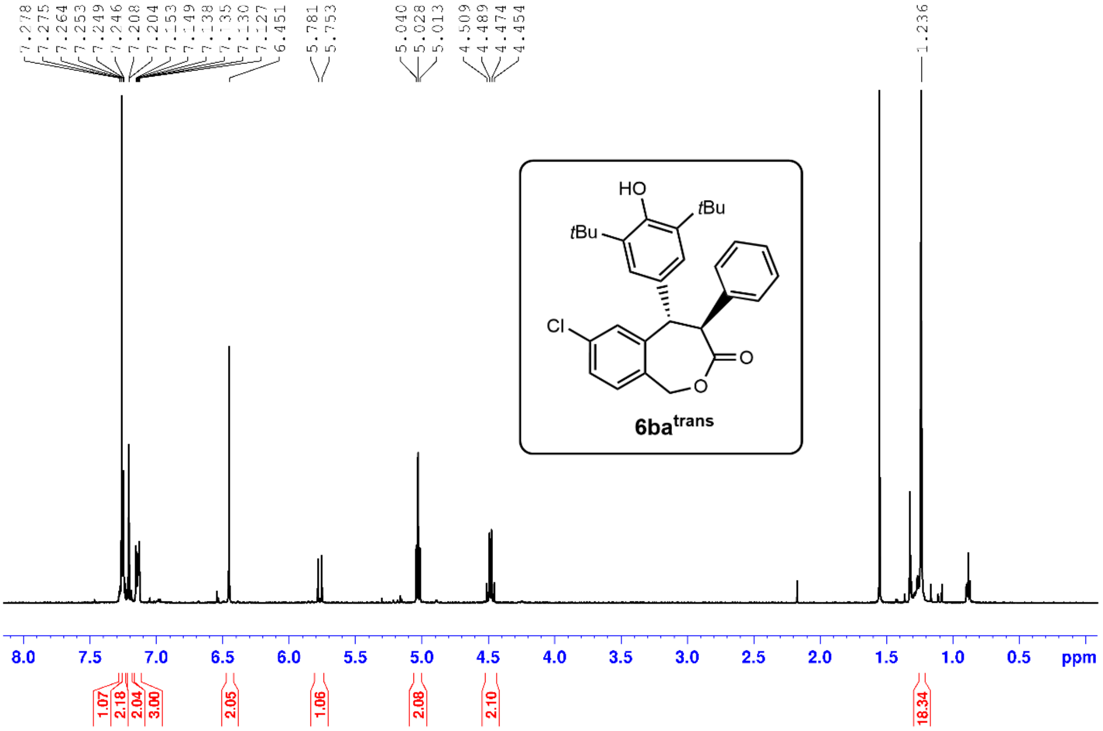

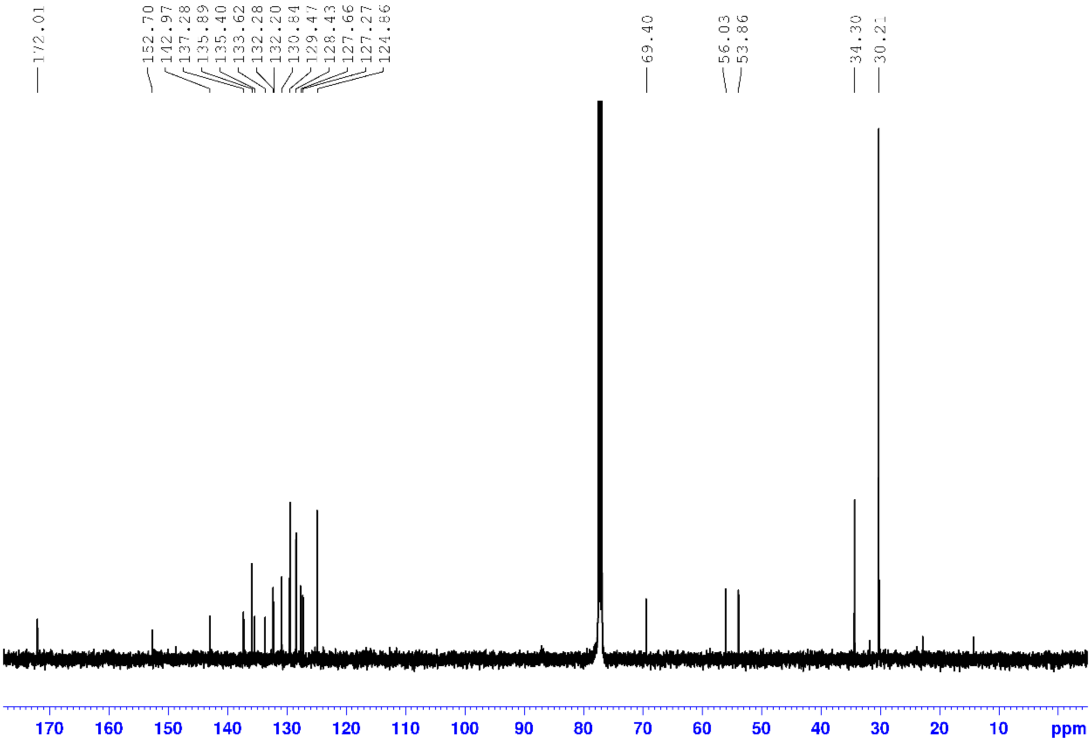

8-Chloro benzo[c]oxepinone derivative (6bb)

Alkylation Product ***5bb***

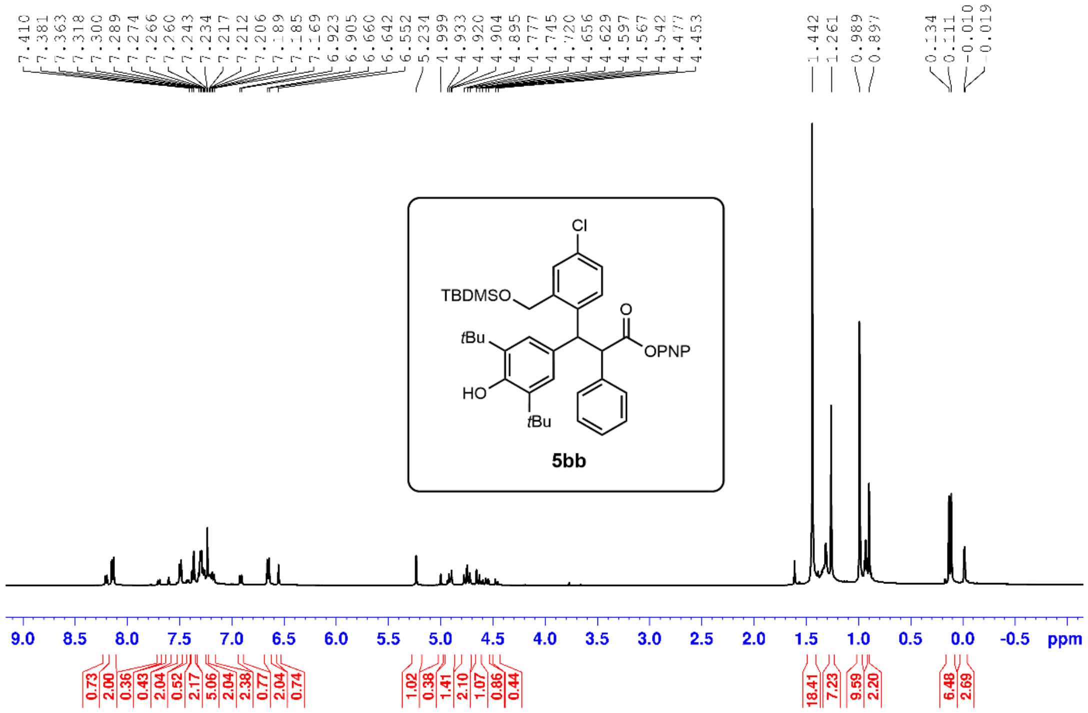

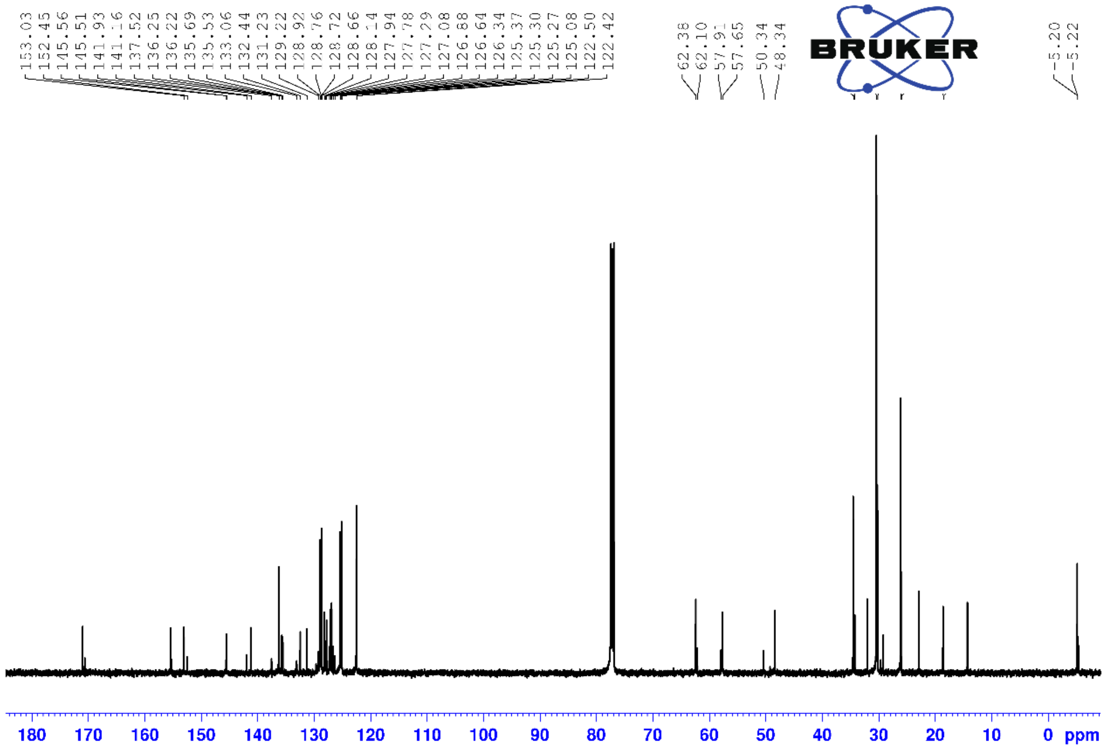

Cyclic products (mixture of diastereomers) ***6bb***^{cis} *and* ***6bb***^{trans}

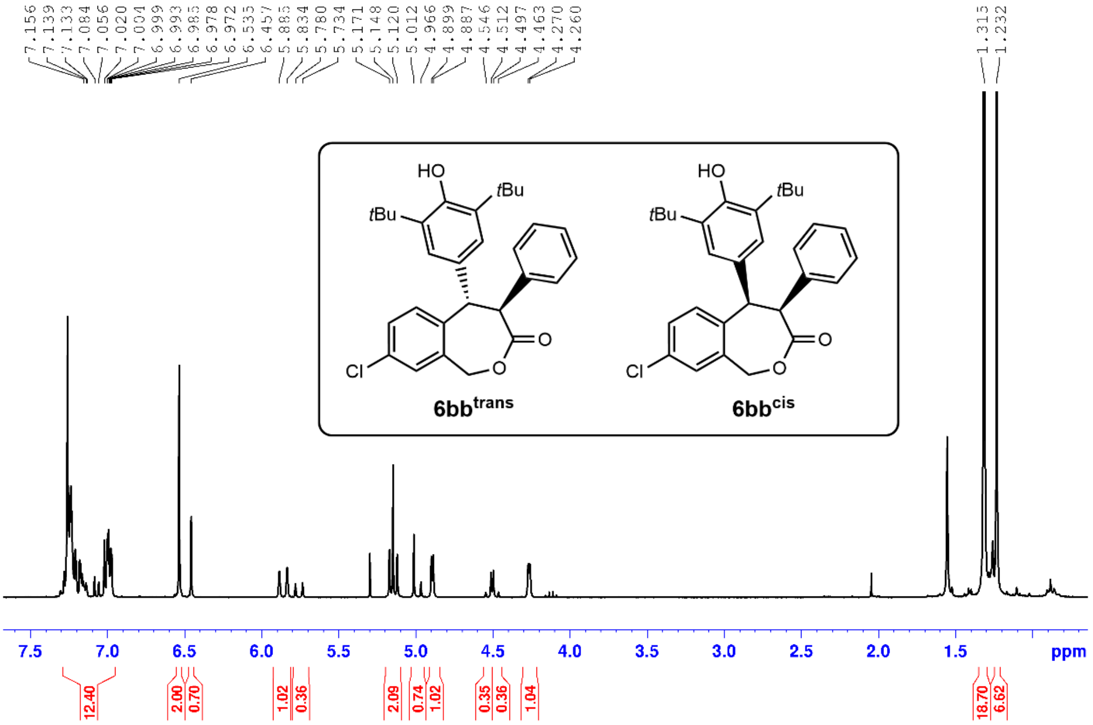

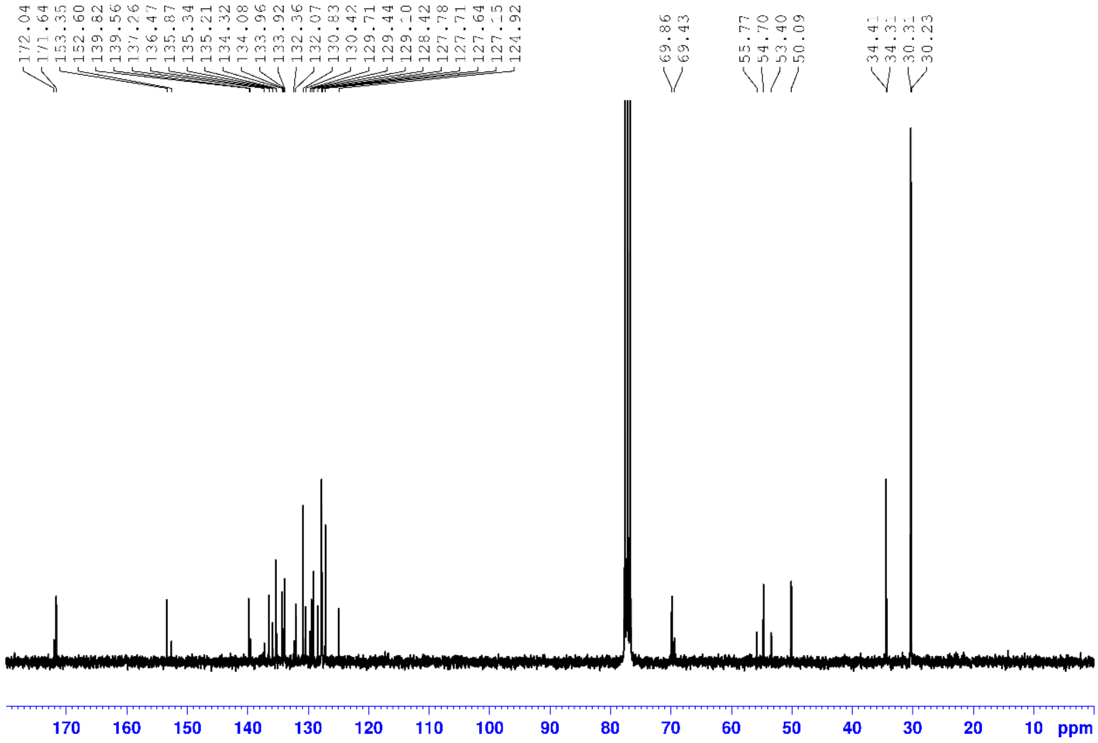

Naphtho[c]oxepinone derivative (6ca)

Alkylation Product ***5ca***

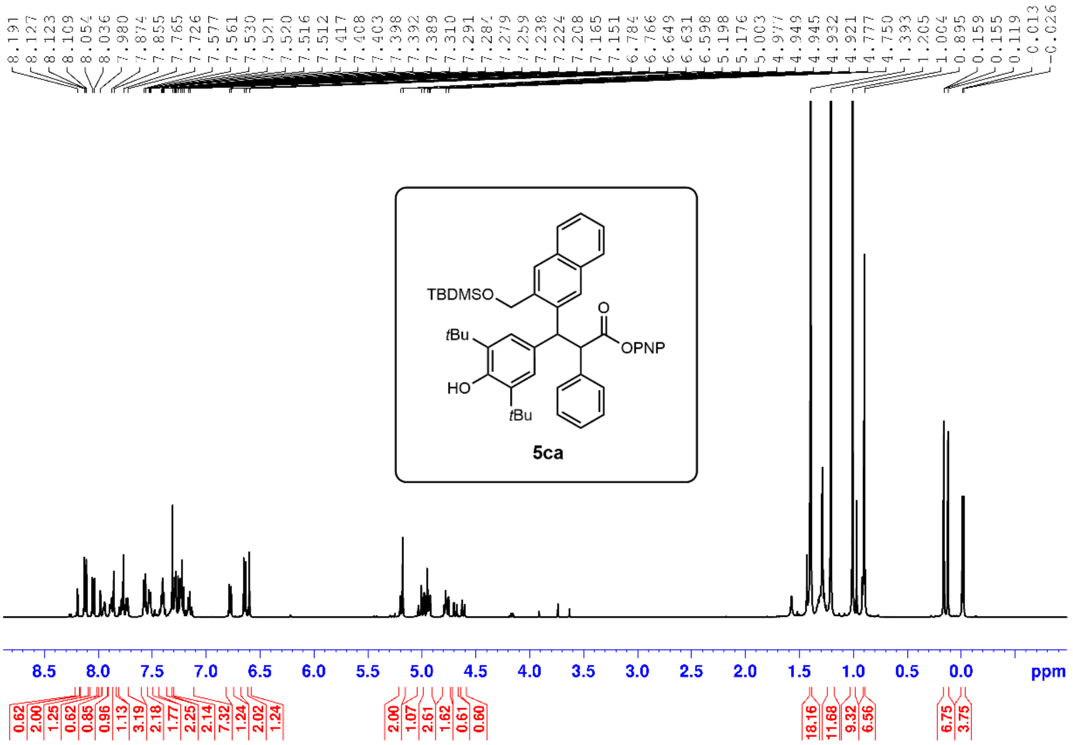

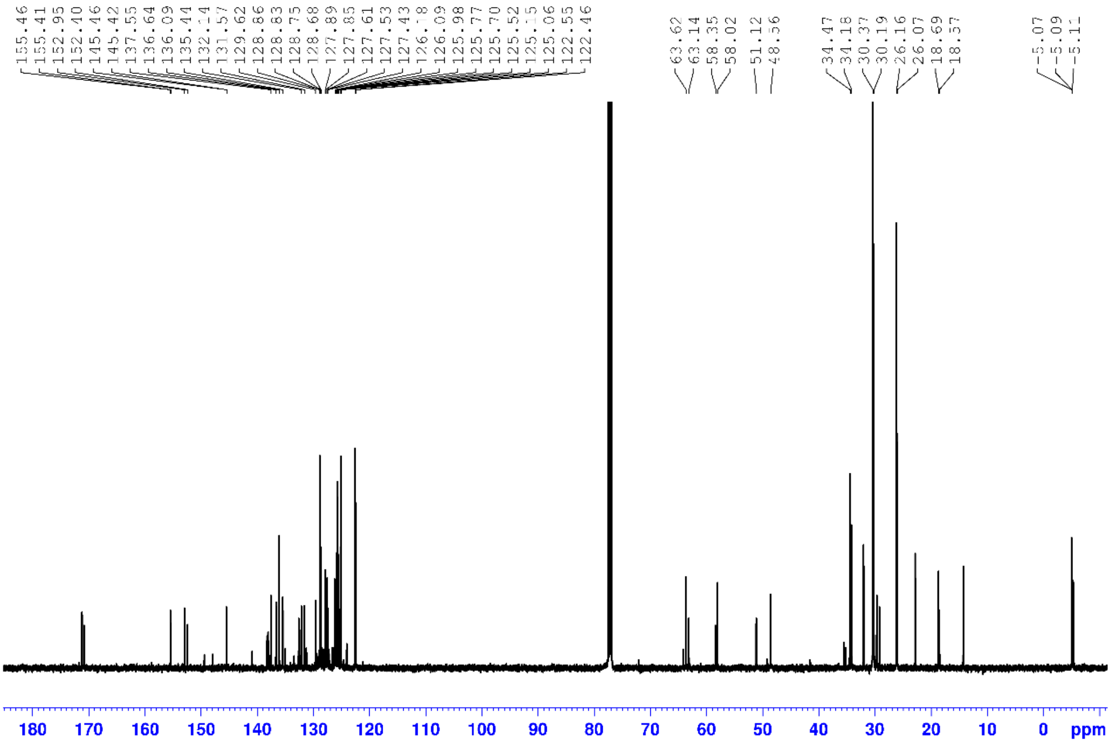

*Cyclic cis and trans products **6ca**^{cis} and **6ca**^{trans} (mixture of diastereomers)*

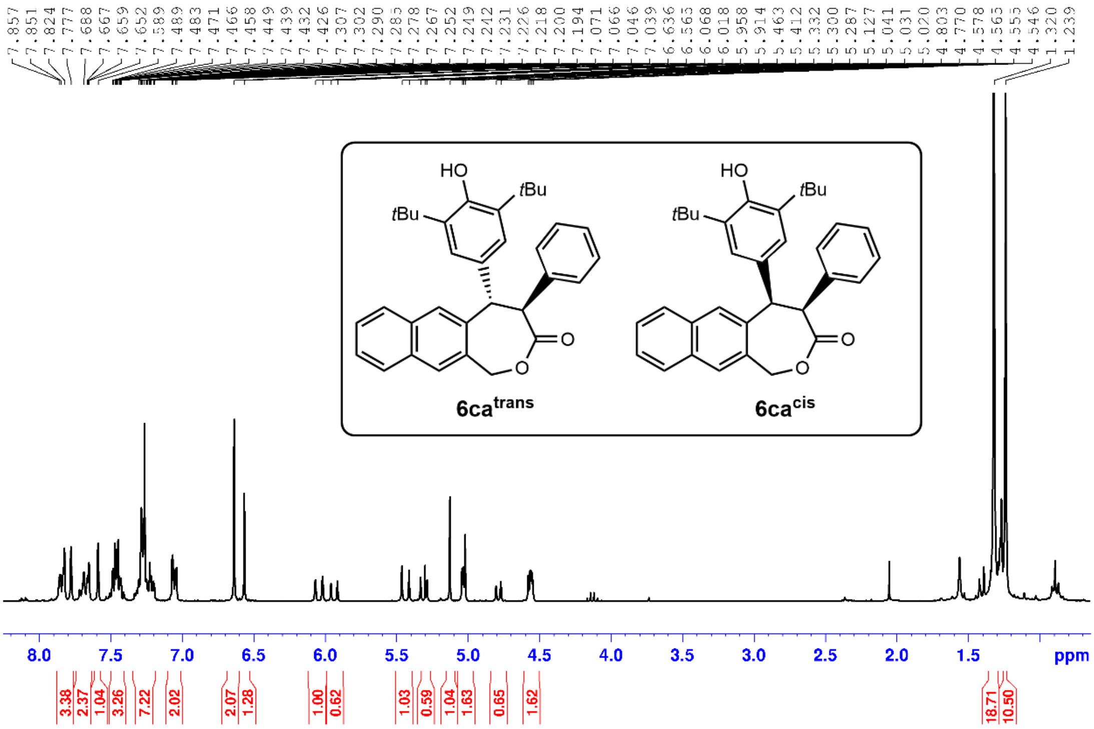

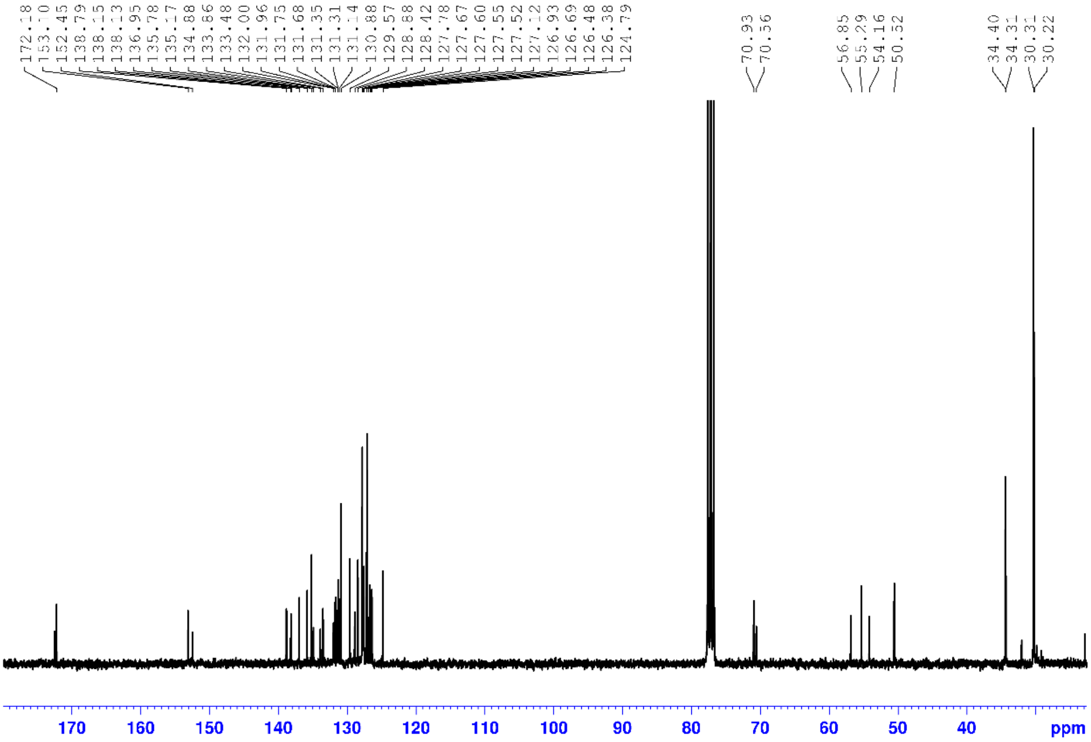

5-(3-(tert-butyl)-4-hydroxy-5-methylphenyl)-4-phenyl-4,5-dihydrobenzo[c]oxepin-3(1H)-one (6da)

Alkylation Product ***5da***

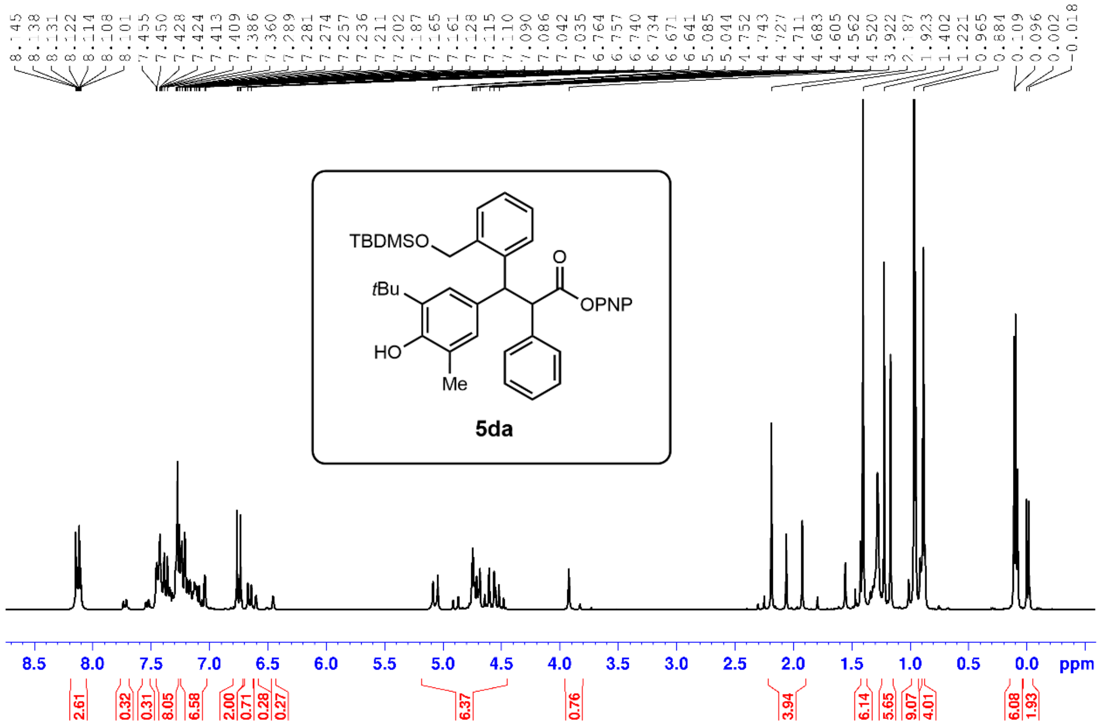

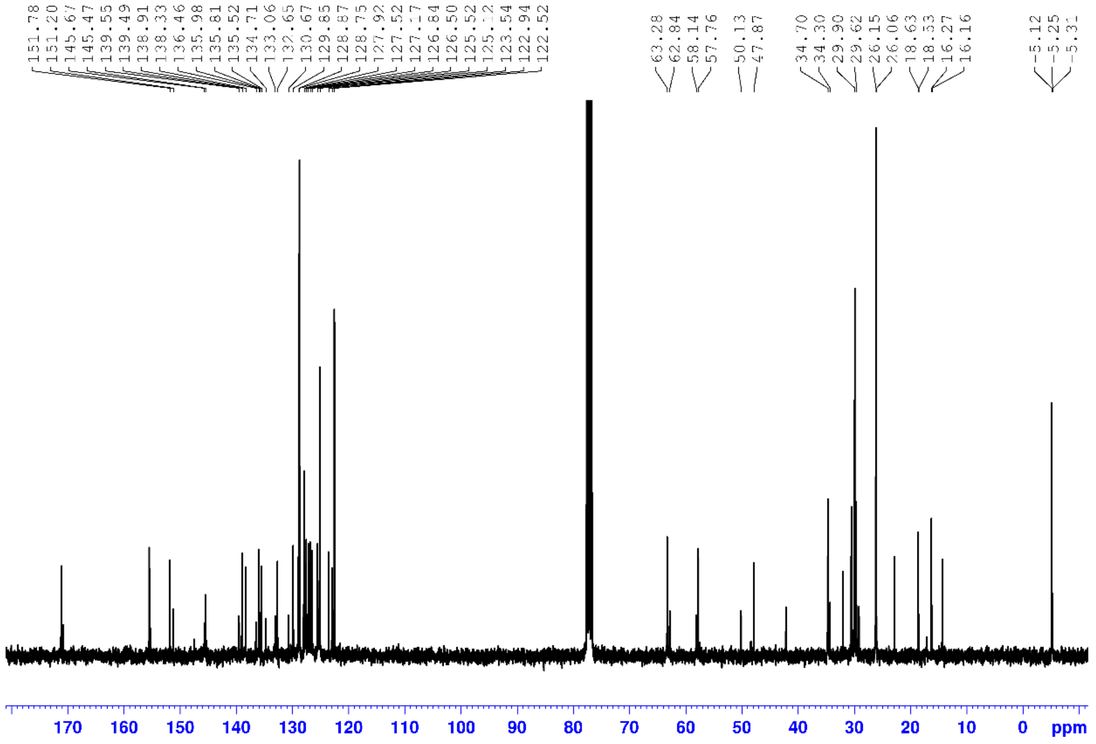

Cis Atropisomers (R_a^{cis} and S_a^{cis}; cis[1] and cis[2])

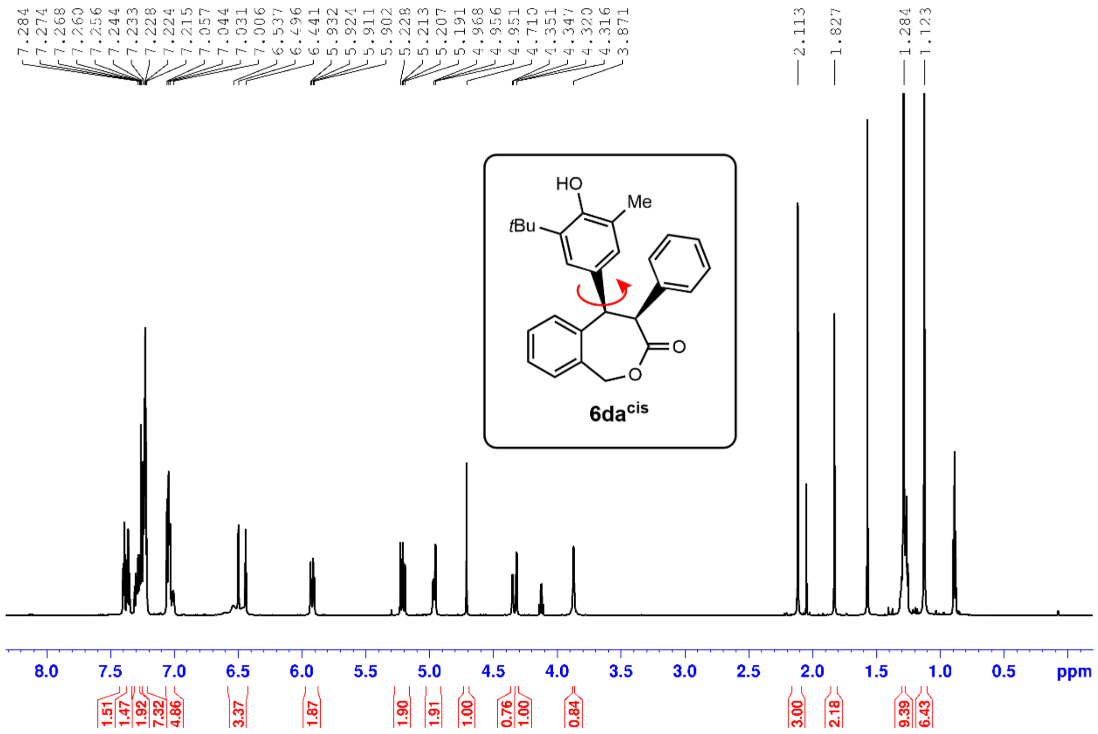

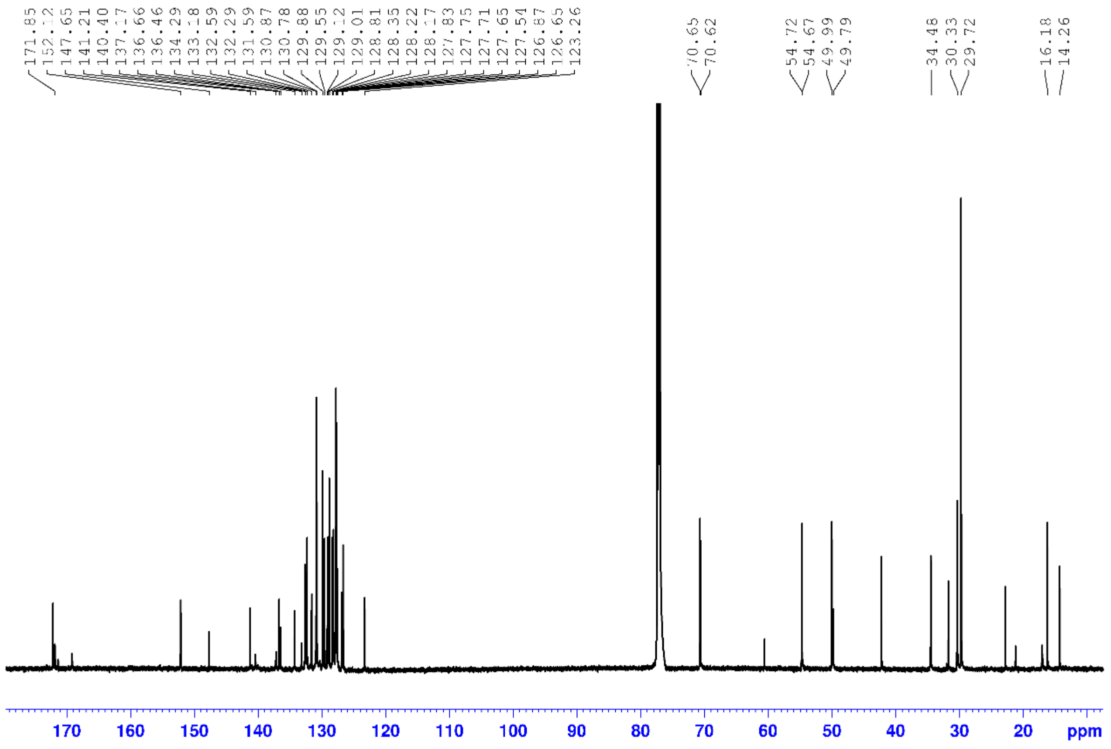

Trans Atropisomers (R_a^{trans} and S_a^{trans}; trans^1 and trans^2)

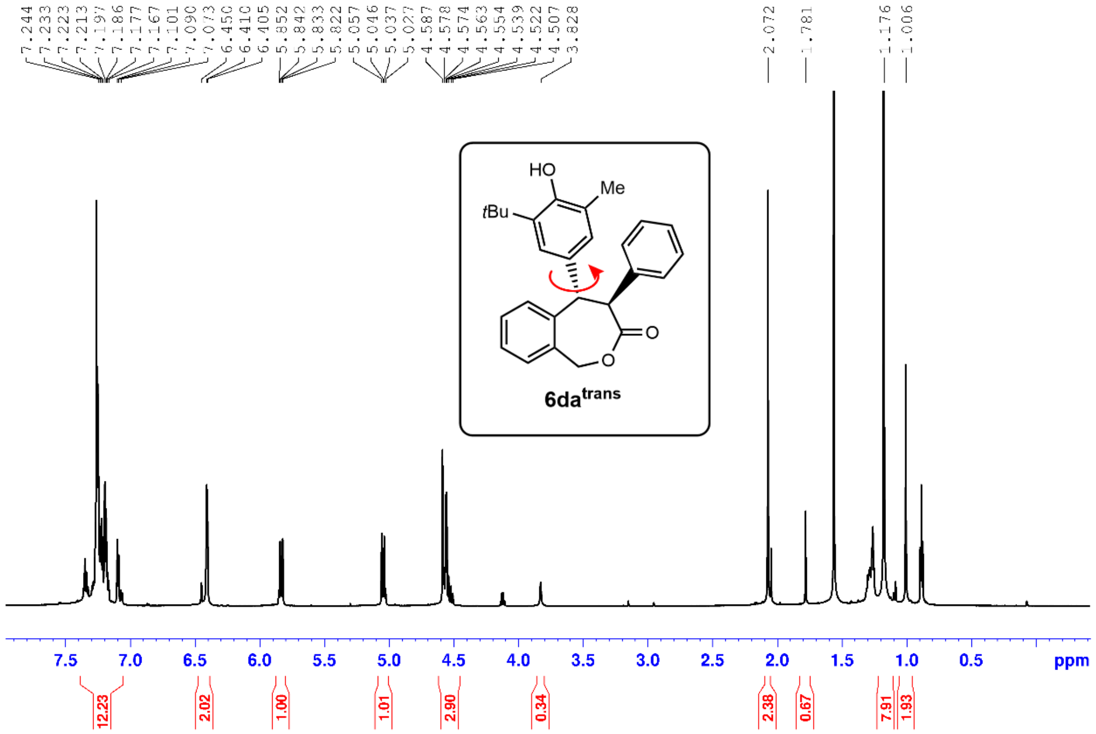

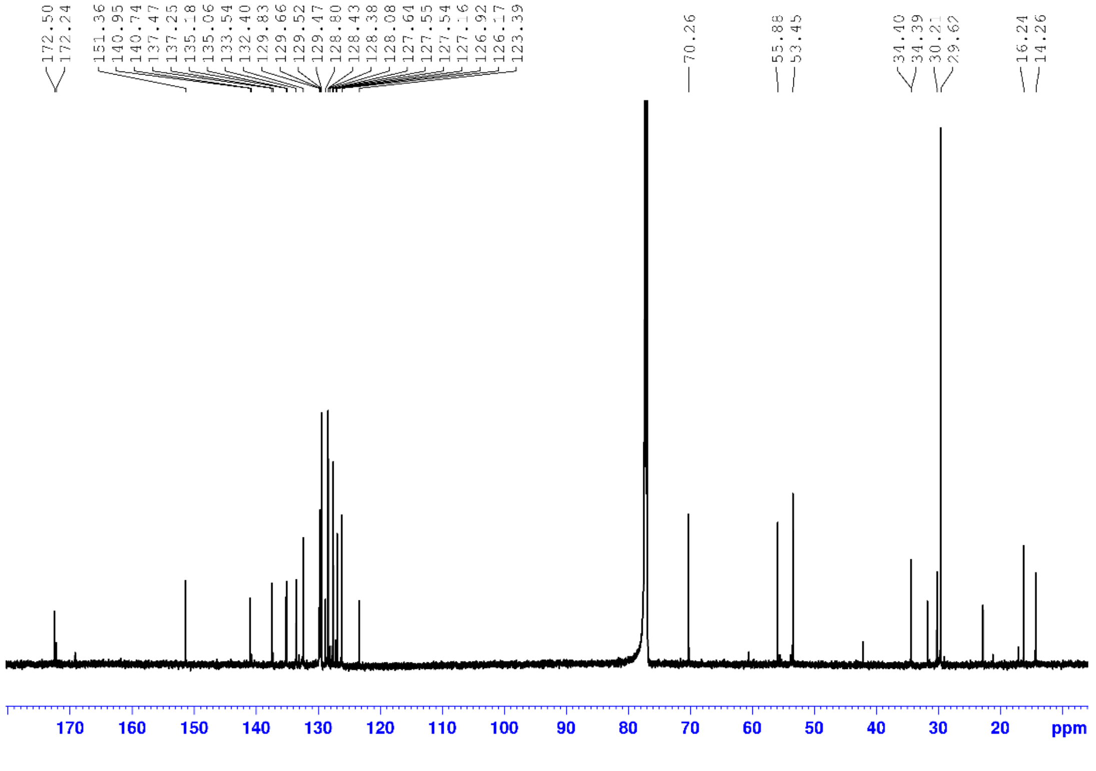

8.3 HPLC Chromatograms for the Cyclization Products

Unsubstituted benzo[c]oxepinone derivative 6a

Chromatogram

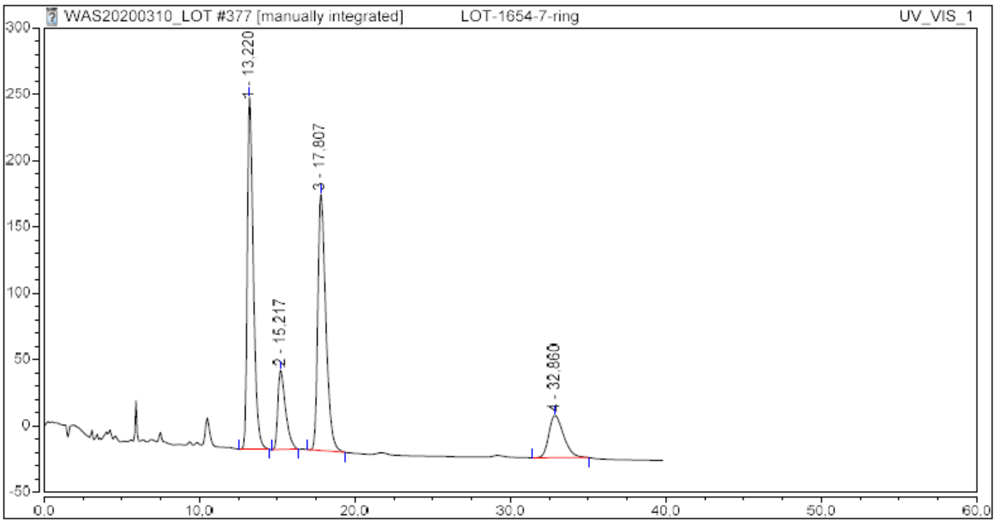

Integration Results

No.	Peak Name	Retention Time<br>min	Area<br>mAU*min	Height<br>mAU	Relative Area<br>%	Relative Height<br>%	Amount<br>n.a.
1		13,220	118,210	265,484	39,13	48,27	n.a.
2		15,217	34,079	59,483	11,28	10,82	n.a.
3		17,807	114,837	193,059	38,01	35,10	n.a.
4		32,860	34,964	31,930	11,57	5,81	n.a.
			**302,089**	**549,957**	**100,00**	**100,00**	

Chromatogram

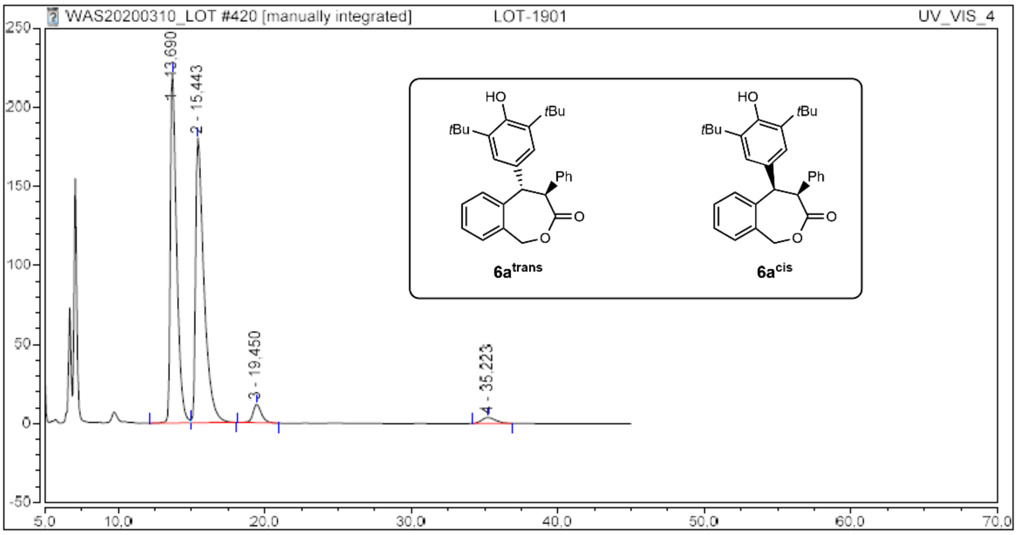

Integration Results

No.	Peak Name	Retention Time<br>min	Area<br>mAU*min	Height<br>mAU	Relative Area<br>%	Relative Height<br>%	Amount<br>n.a.
1		13,690	110,432	221,061	45,58	53,06	n.a.
2		15,443	119,923	180,175	49,50	43,24	n.a.
3		19,450	7,812	11,713	3,22	2,81	n.a.
4		35,223	4,089	3,701	1,69	0,89	n.a.
			**242,255**	**416,651**	**100,00**	**100,00**	

2-Fluorophenyl benzo[c]oxepinone derivative (6b)

Chromatogram

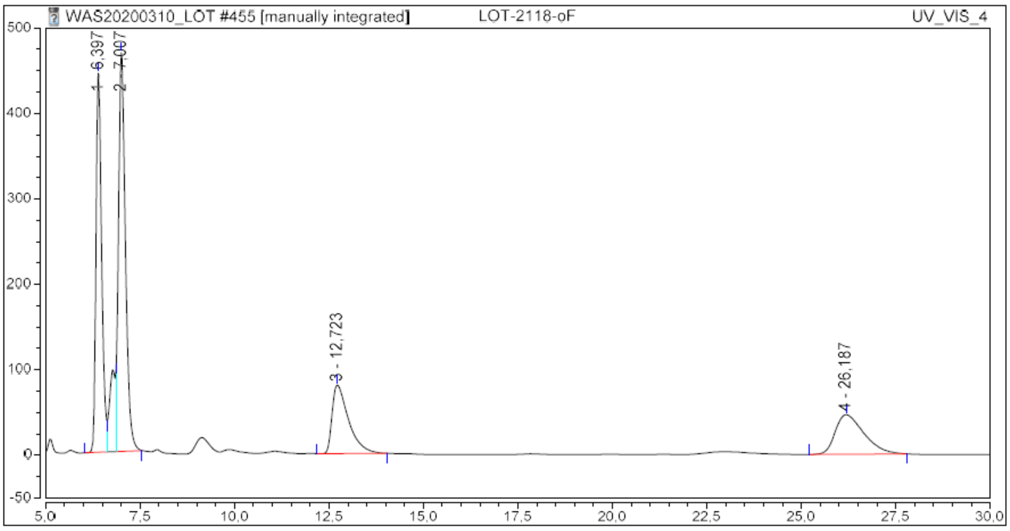

Integration Results

No.	Peak Name	Retention Time min	Area mAU*min	Height mAU	Relative Area %	Relative Height %	Amount n.a.
1		6,397	77,254	443,391	30,00	42,80	n.a.
2		7,007	99,179	466,224	38,51	45,00	n.a.
3		12,723	40,442	80,228	15,70	7,74	n.a.
4		26,187	40,668	46,201	15,79	4,46	n.a.
			**257,543**	**1036,043**	**100,00**	**100,00**	

Chromatogram

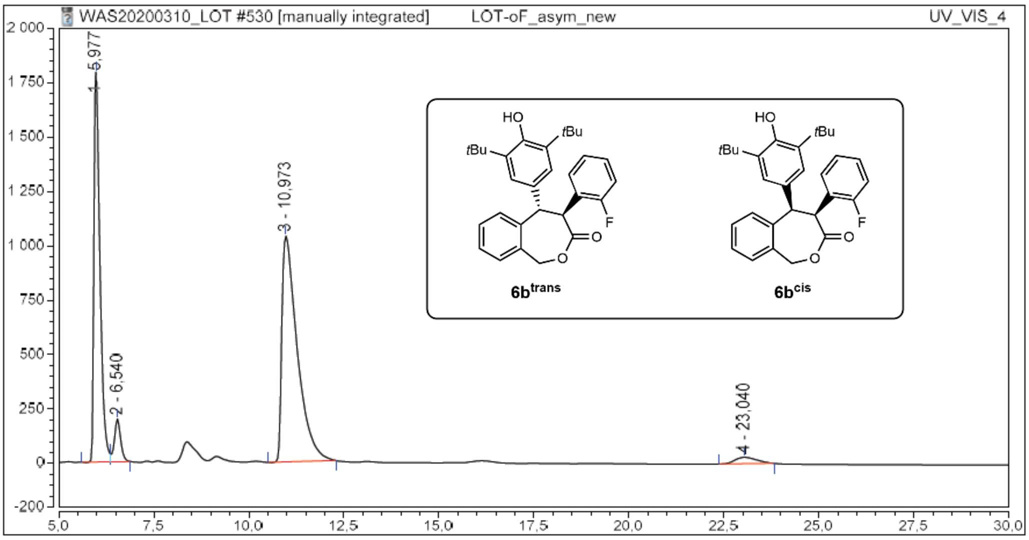

Integration Results

No.	Peak Name	Retention Time min	Area mAU*min	Height mAU	Relative Area %	Relative Height %	Amount n.a.
1		5,977	326,588	1790,590	37,48	58,61	n.a.
2		6,540	37,805	197,071	4,34	6,45	n.a.
3		10,973	487,240	1037,896	55,91	33,97	n.a.
4		23,040	19,831	29,485	2,28	0,97	n.a.
			**871,463**	**3055,042**	**100,00**	**100,00**	

4-Fluorophenyl benzo[c]oxepinone derivative (6c)

Chromatogram

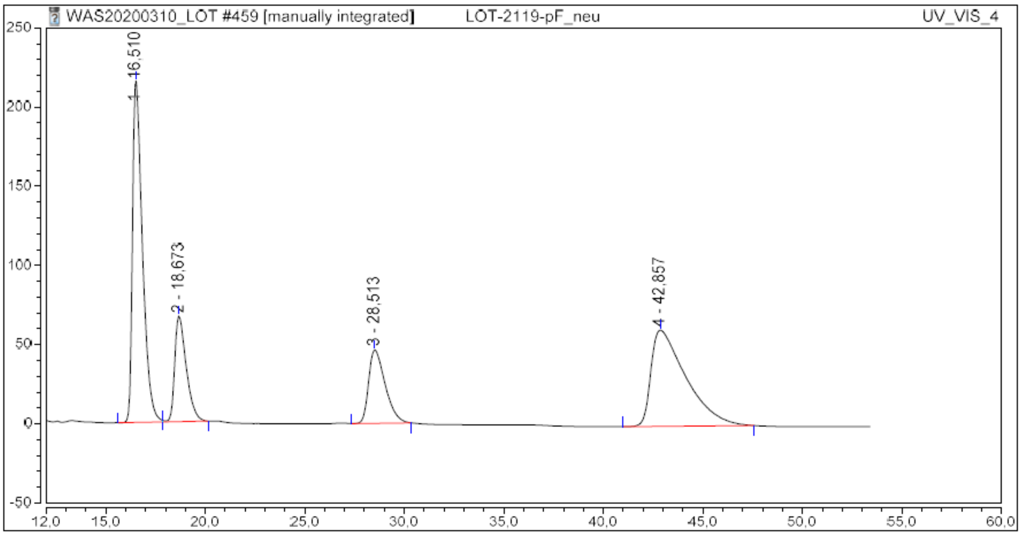

Integration Results

No.	Peak Name	Retention Time min	Area mAU*min	Height mAU	Relative Area %	Relative Height %	Amount n.a.
1		16,510	124,595	215,583	36,92	55,38	n.a.
2		18,673	44,857	66,714	13,29	17,14	n.a.
3		28,513	44,443	46,304	13,17	11,90	n.a.
4		42,857	123,588	60,669	36,62	15,59	n.a.
			**337,483**	**389,270**	**100,00**	**100,00**	

Chromatogram

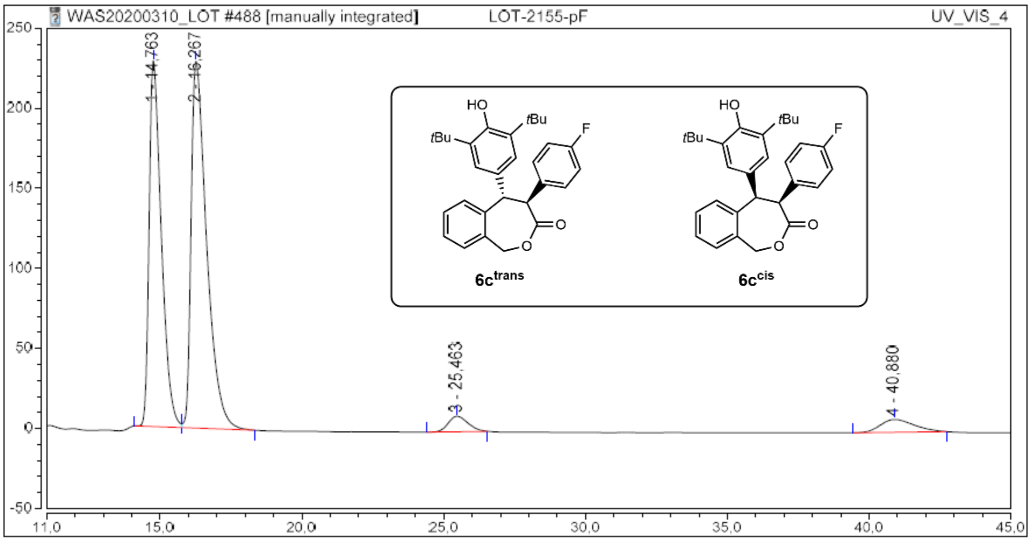

Integration Results

No.	Peak Name	Retention Time min	Area mAU*min	Height mAU	Relative Area %	Relative Height %	Amount n.a.
1		14,763	114,332	228,405	41,15	48,08	n.a.
2		16,267	144,760	229,010	52,11	48,21	n.a.
3		25,463	7,732	9,689	2,78	2,04	n.a.
4		40,880	10,999	7,949	3,96	1,67	n.a.
			**277,822**	**475,052**	**100,00**	**100,00**	

3-Chlorophenyl benzo[c]oxepinone derivative (3d)

Chromatogram

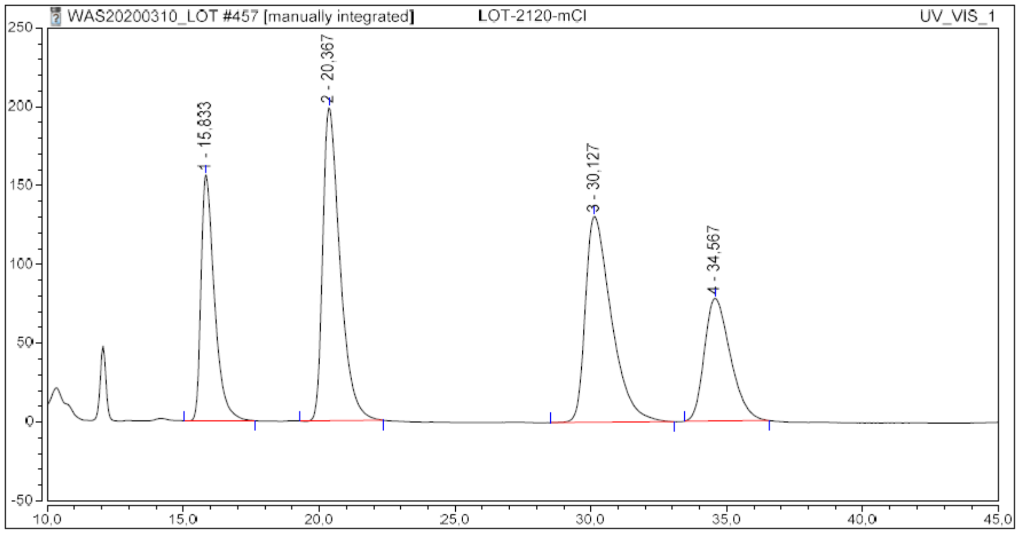

Integration Results

No.	Peak Name	Retention Time min	Area mAU*min	Height mAU	Relative Area %	Relative Height %	Amount n.a.
1		15,833	89,224	155,920	19,12	27,68	n.a.
2		20,367	146,074	198,750	31,30	35,29	n.a.
3		30,127	145,237	130,698	31,12	23,21	n.a.
4		34,567	86,170	77,833	18,46	13,82	n.a.
			**466,705**	**563,201**	**100,00**	**100,00**	

Chromatogram

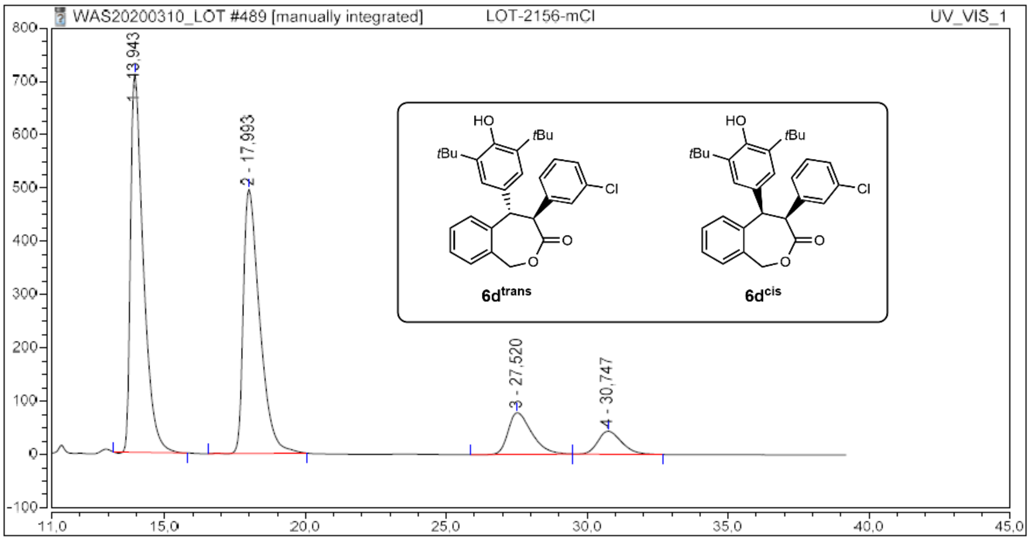

Integration Results

No.	Peak Name	Retention Time min	Area mAU*min	Height mAU	Relative Area %	Relative Height %	Amount n.a.
1		13,943	373,328	709,870	45,30	53,48	n.a.
2		17,993	333,057	496,029	40,41	37,37	n.a.
3		27,520	75,504	78,265	9,16	5,90	n.a.
4		30,747	42,230	43,146	5,12	3,25	n.a.
			**824,119**	**1327,310**	**100,00**	**100,00**	

4-Chlorophenyl benzo[c]oxepinone derivative (6e)

Chromatogram

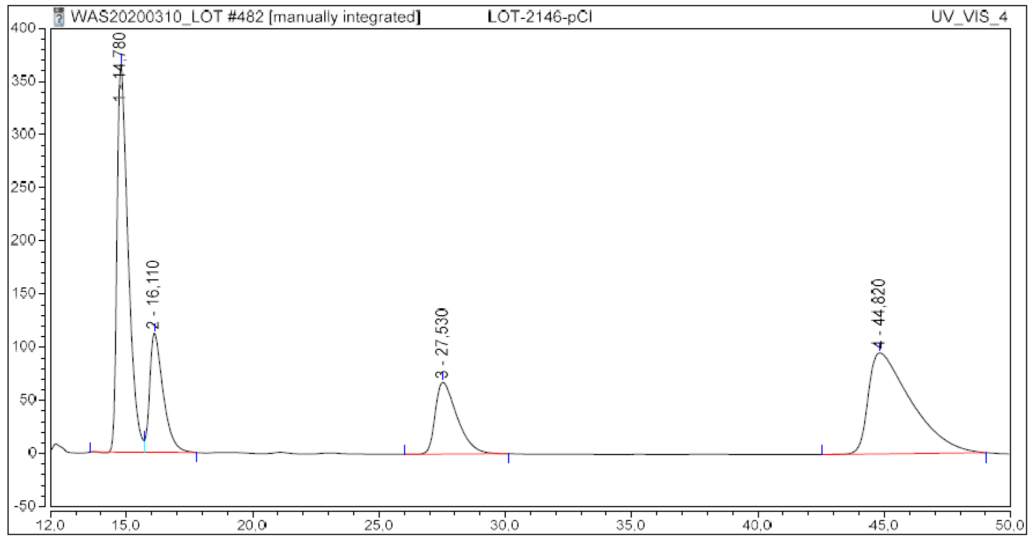

Integration Results

No.	Peak Name	Retention Time<br>min	Area<br>mAU*min	Height<br>mAU	Relative Area<br>%	Relative Height<br>%	Amount<br>n.a.
1		14,780	187,049	365,166	36,62	57,05	n.a.
2		16,110	69,231	112,276	13,55	17,54	n.a.
3		27,530	67,810	67,623	13,27	10,56	n.a.
4		44,820	186,732	95,046	36,56	14,85	n.a.
			**510,822**	**640,111**	**100,00**	**100,00**	

Chromatogram

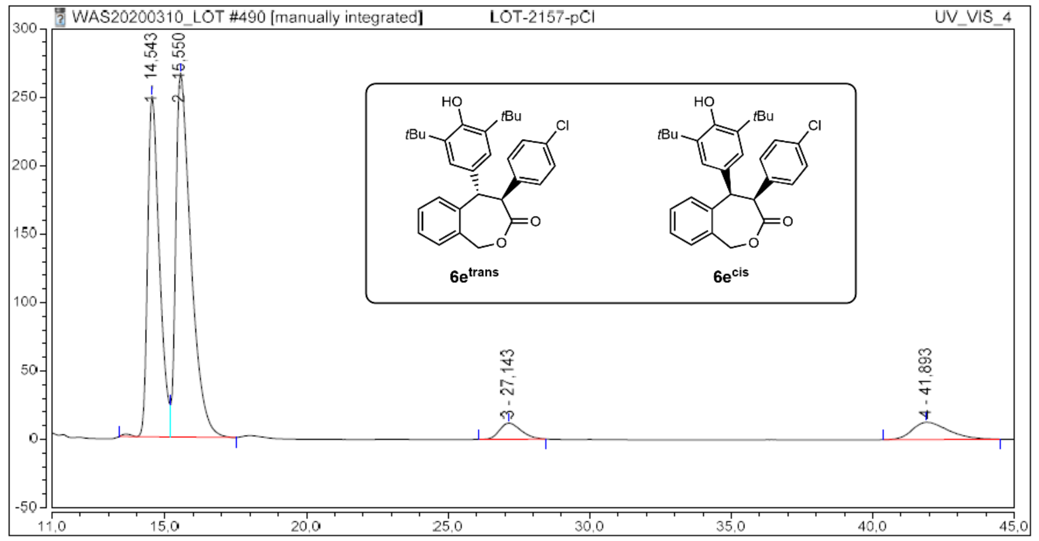

Integration Results

No.	Peak Name	Retention Time<br>min	Area<br>mAU*min	Height<br>mAU	Relative Area<br>%	Relative Height<br>%	Amount<br>n.a.
1		14,543	120,087	248,837	38,33	46,15	n.a.
2		15,550	164,067	265,956	52,37	49,32	n.a.
3		27,143	10,436	11,811	3,33	2,19	n.a.
4		41,893	18,722	12,621	5,98	2,34	n.a.
			**313,312**	**539,225**	**100,00**	**100,00**	

Dichlorophenyl benzo[c]oxepinone derivative (6f)

Chromatogram

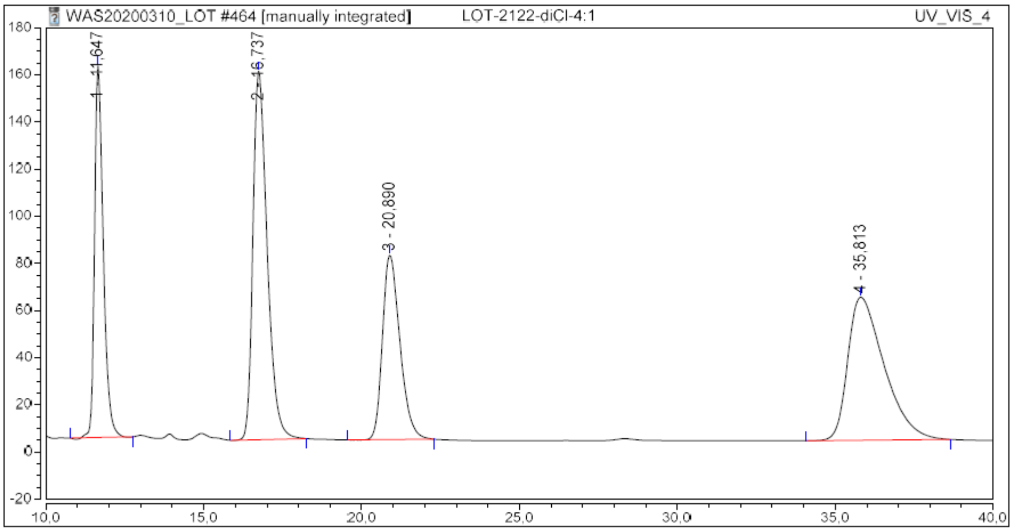

Integration Results

No.	Peak Name	Retention Time min	Area mAU*min	Height mAU	Relative Area %	Relative Height %	Amount n.a.
1		11,647	50,353	157,558	19,14	34,79	n.a.
2		16,737	81,617	156,347	31,02	34,52	n.a.
3		20,890	50,156	78,263	19,06	17,28	n.a.
4		35,813	80,963	60,714	30,77	13,41	n.a.
			**263,089**	**452,881**	**100,00**	**100,00**	

Chromatogram

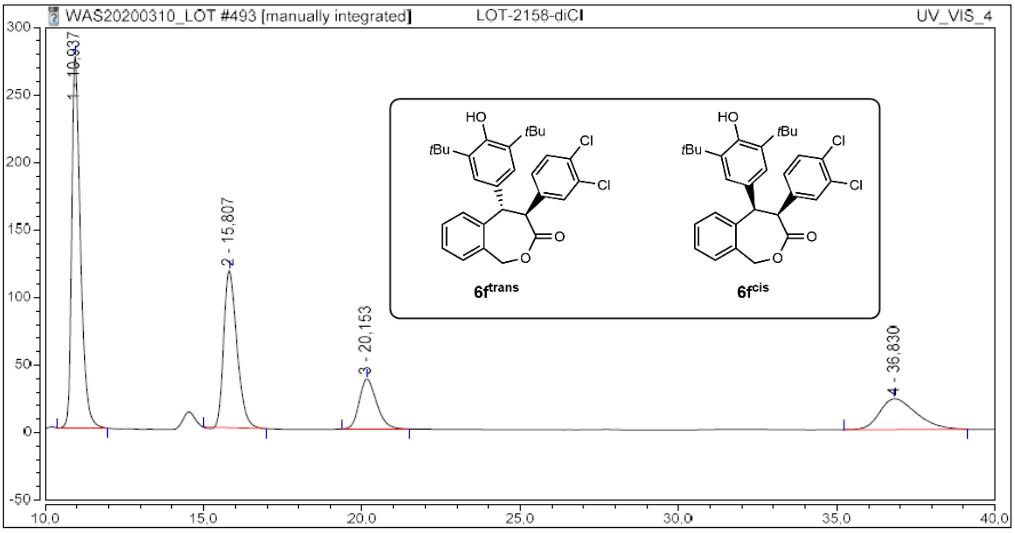

Integration Results

No.	Peak Name	Retention Time min	Area mAU*min	Height mAU	Relative Area %	Relative Height %	Amount n.a.
1		10,937	87,900	275,546	44,33	60,99	n.a.
2		15,807	56,389	116,589	28,44	25,80	n.a.
3		20,153	23,479	36,926	11,84	8,17	n.a.
4		36,830	30,533	22,764	15,40	5,04	n.a.
			**198,301**	**451,825**	**100,00**	**100,00**	

4-Bromophenyl benzo[c]oxepinone derivative (6g)

Chromatogram

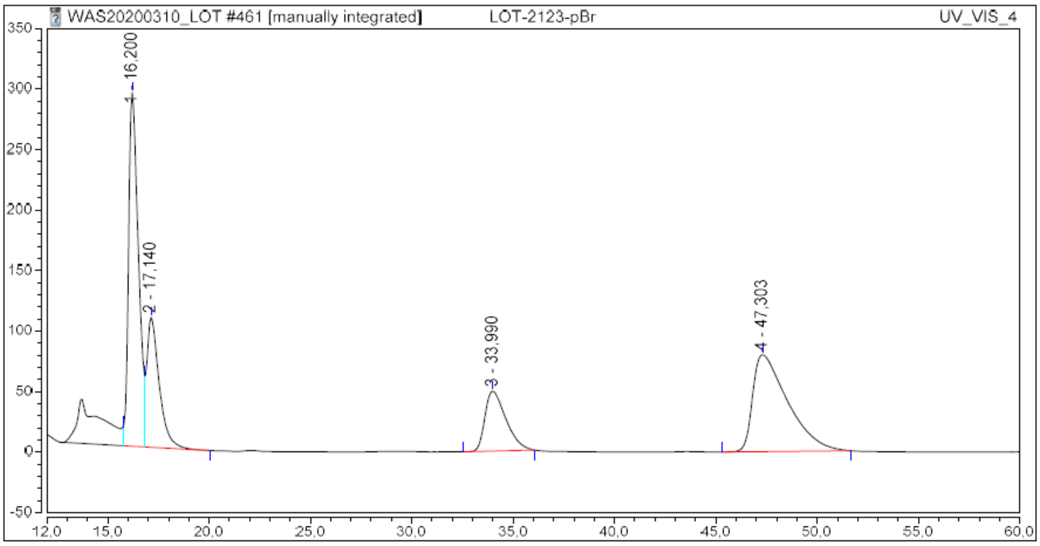

Integration Results

No.	Peak Name	Retention Time min	Area mAU*min	Height mAU	Relative Area %	Relative Height %	Amount n.a.
1		16,200	163,884	292,334	35,75	55,21	n.a.
2		17,140	75,471	107,526	16,46	20,31	n.a.
3		33,990	59,457	49,586	12,97	9,36	n.a.
4		47,303	159,663	80,043	34,82	15,12	n.a.
			**458,475**	**529,490**	**100,00**	**100,00**	

Chromatogram

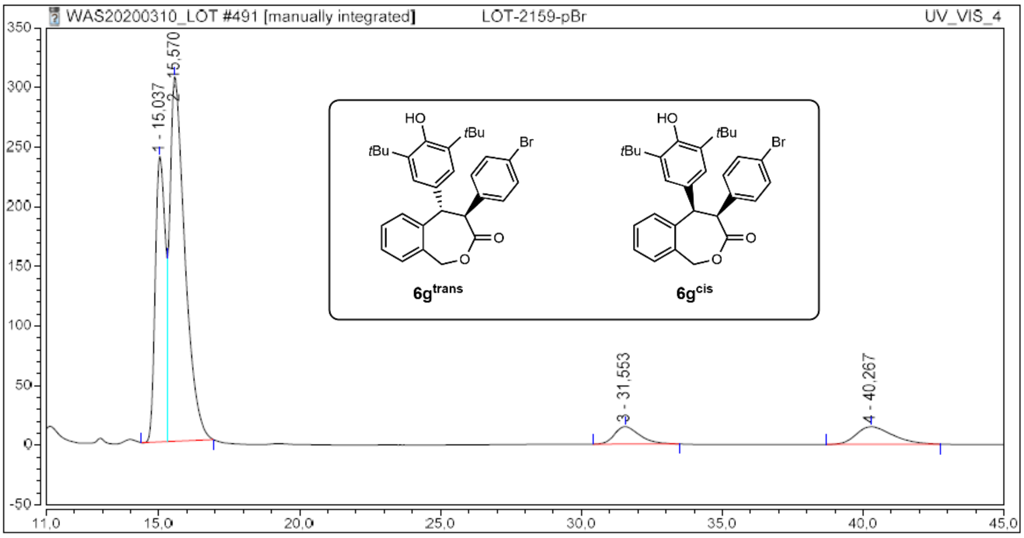

Integration Results

No.	Peak Name	Retention Time min	Area mAU*min	Height mAU	Relative Area %	Relative Height %	Amount n.a.
1		15,037	97,914	239,496	30,17	41,68	n.a.
2		15,570	189,431	305,831	58,37	53,23	n.a.
3		31,553	15,104	14,519	4,65	2,53	n.a.
4		40,267	22,103	14,728	6,81	2,56	n.a.
			**324,553**	**574,574**	**100,00**	**100,00**	

Due to overlap in the chromatogram, the diastereomers were separated and the measurement was repeated:

Chromatogram

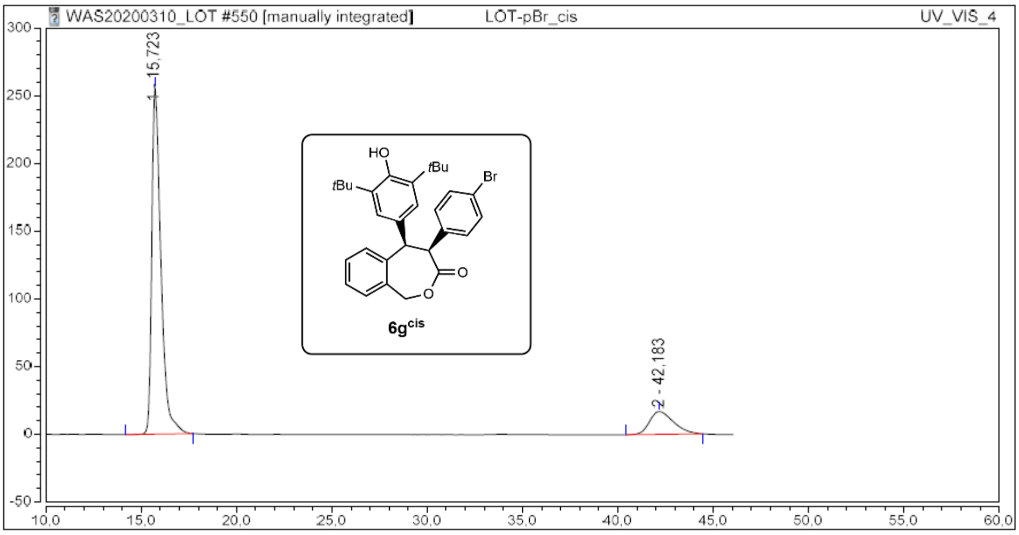

Integration Results

No.	Peak Name	Retention Time<br>min	Area<br>mAU*min	Height<br>mAU	Relative Area<br>%	Relative Height<br>%	Amount<br>n.a.
1		15,723	142,175	255,961	85,27	93,84	n.a.
2		42,183	24,566	16,811	14,73	6,16	n.a.
			**166,741**	**272,773**	**100,00**	**100,00**	

Chromatogram

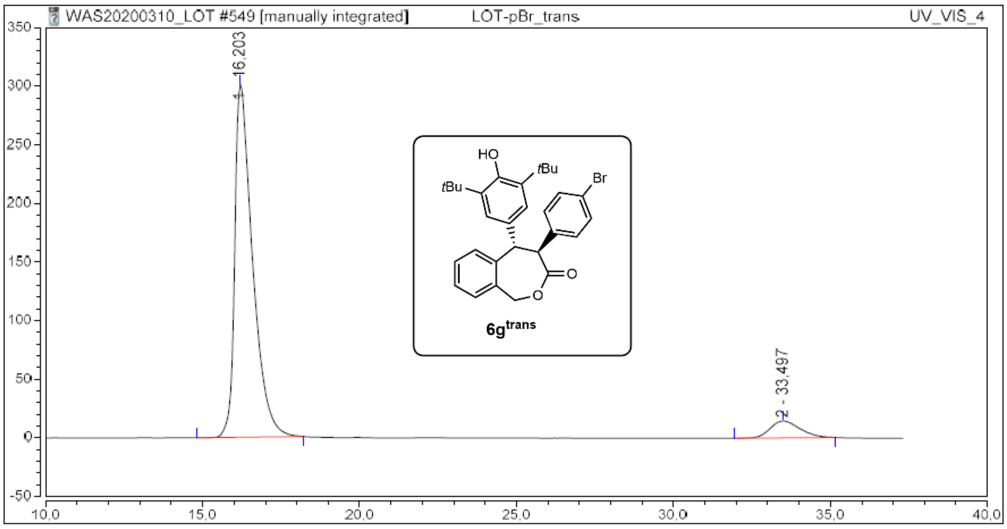

Integration Results

No.	Peak Name	Retention Time<br>min	Area<br>mAU*min	Height<br>mAU	Relative Area<br>%	Relative Height<br>%	Amount<br>n.a.
1		16,203	198,104	300,984	92,63	95,45	n.a.
2		33,497	15,754	14,343	7,37	4,55	n.a.
			**213,857**	**315,327**	**100,00**	**100,00**	

3-Iodophenyl benzo[c]oxepinone derivative (6h)

Chromatogram

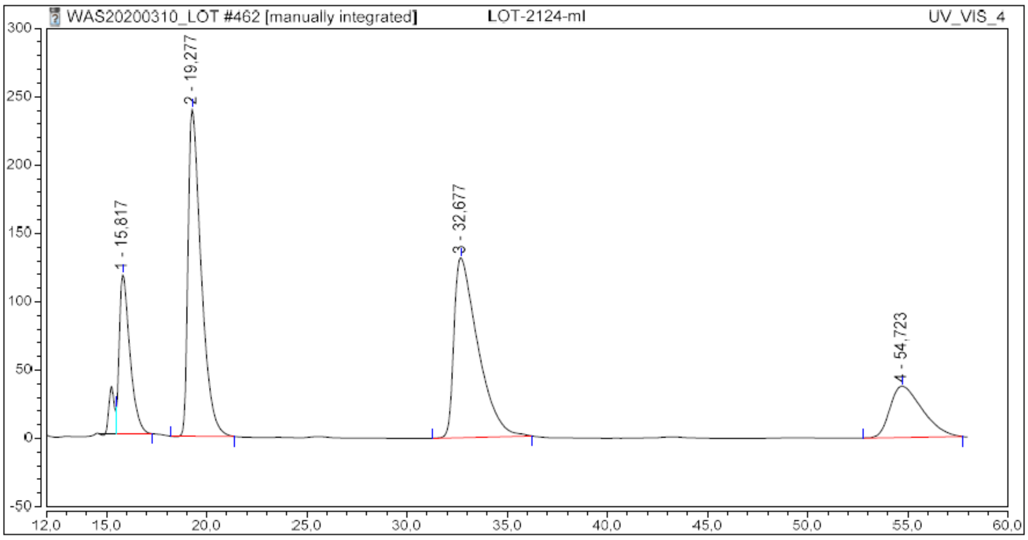

Integration Results

No.	Peak Name	Retention Time min	Area mAU*min	Height mAU	Relative Area %	Relative Height %	Amount n.a.
1		15,817	71,110	116,560	14,11	22,17	n.a.
2		19,277	181,236	239,851	35,95	45,63	n.a.
3		32,677	181,530	131,689	36,01	25,05	n.a.
4		54,723	70,241	37,590	13,93	7,15	n.a.
			**504,117**	**525,690**	**100,00**	**100,00**	

Chromatogram

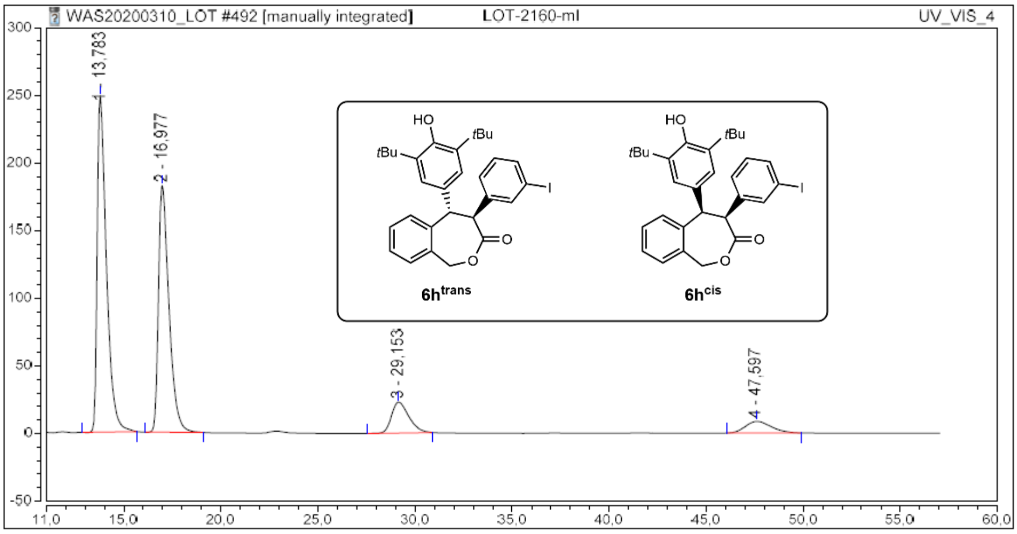

Integration Results

No.	Peak Name	Retention Time min	Area mAU*min	Height mAU	Relative Area %	Relative Height %	Amount n.a.
1		13,783	136,676	248,848	47,43	53,78	n.a.
2		16,977	114,593	182,434	39,77	39,42	n.a.
3		29,153	23,519	22,864	8,16	4,94	n.a.
4		47,597	13,377	8,602	4,64	1,86	n.a.
			**288,165**	**462,748**	**100,00**	**100,00**	

4-Methoxyphenyl benzo[c]oxepinone derivative (6i)

Chromatogram

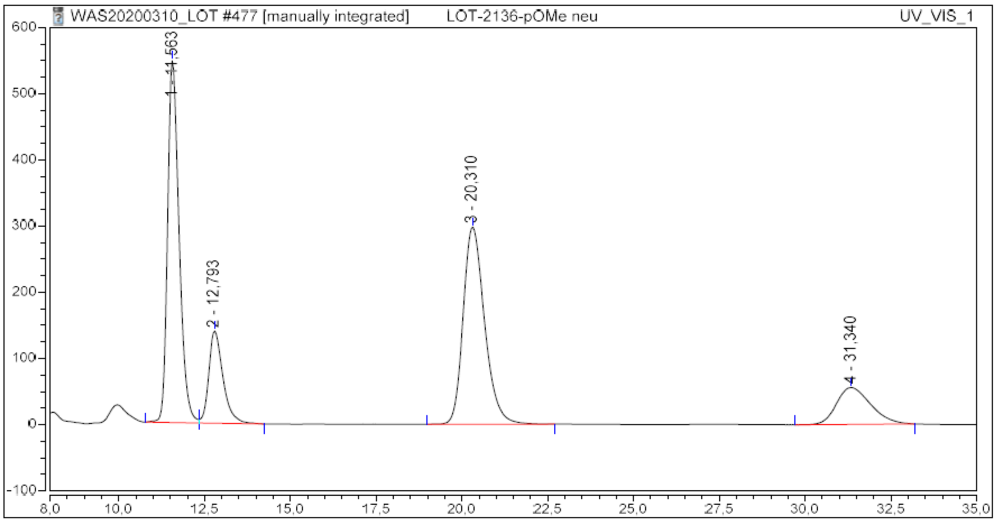

Integration Results

No.	Peak Name	Retention Time min	Area mAU*min	Height mAU	Relative Area %	Relative Height %	Amount n.a.
1		11,563	213,179	546,881	38,09	52,61	n.a.
2		12,793	66,831	139,130	11,94	13,38	n.a.
3		20,310	215,514	297,859	38,51	28,65	n.a.
4		31,340	64,148	55,647	11,46	5,35	n.a.
			**559,672**	**1039,517**	**100,00**	**100,00**	

Chromatogram

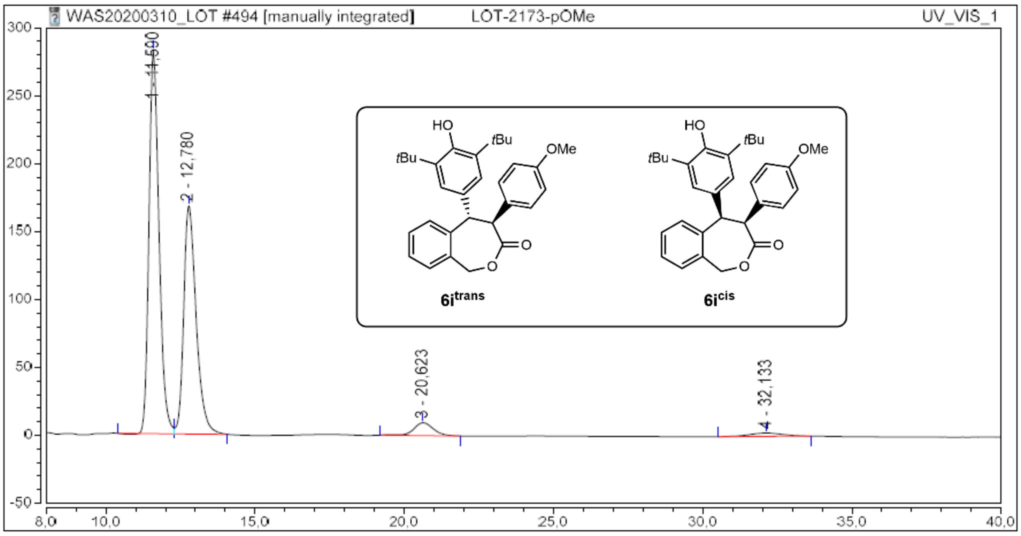

Integration Results

No.	Peak Name	Retention Time min	Area mAU*min	Height mAU	Relative Area %	Relative Height %	Amount n.a.
1		11,590	110,016	282,043	54,56	61,02	n.a.
2		12,780	81,122	168,099	40,23	36,37	n.a.
3		20,623	7,242	9,553	3,59	2,07	n.a.
4		32,133	3,252	2,530	1,61	0,55	n.a.
			**201,631**	**462,225**	**100,00**	**100,00**	

Dimethoxyphenyl benzo[c]oxepinone derivative (6j)

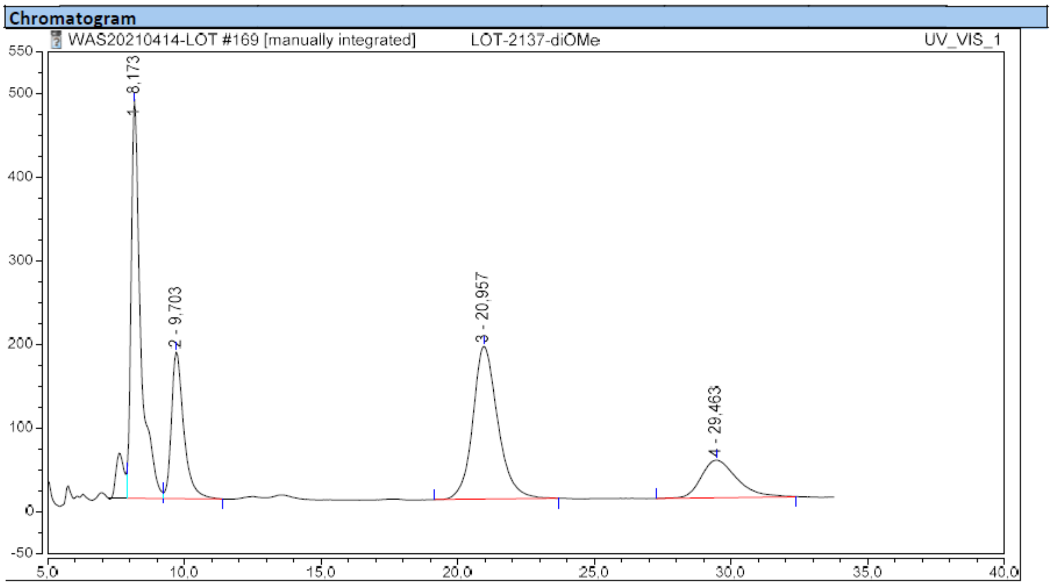

Integration Results

No.	Peak Name	Retention Time min	Area mAU*min	Height mAU	Relative Area %	Relative Height %	Amount n.a.
1		8,173	195,805	472,303	35,66	53,94	n.a.
2		9,703	91,928	175,730	16,74	20,07	n.a.
3		20,957	193,365	182,662	35,22	20,86	n.a.
4		29,463	67,920	44,901	12,37	5,13	n.a.
**Total:**			**549,018**	**875,595**	**100,00**	**100,00**	

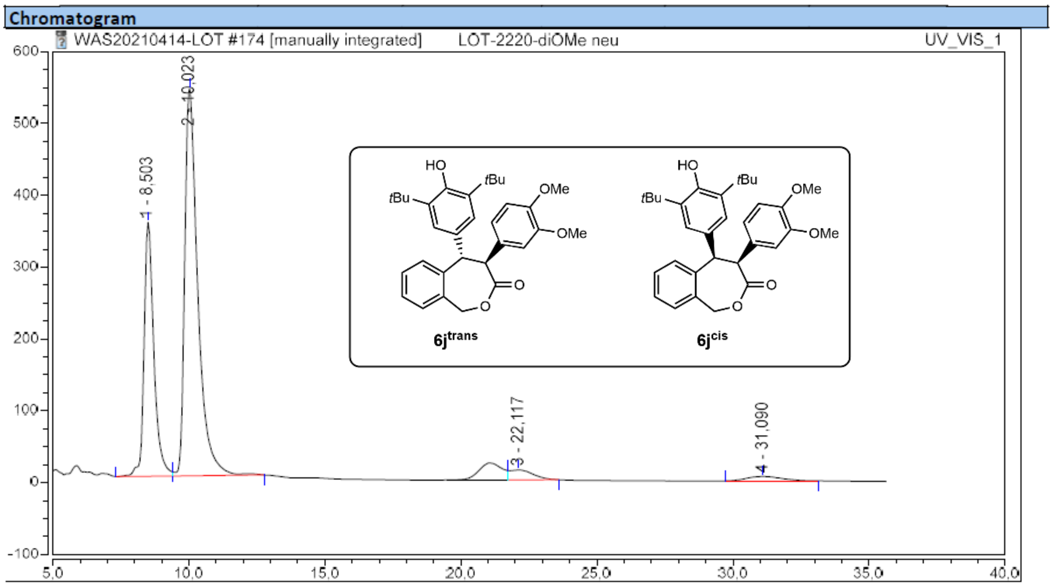

Integration Results

No.	Peak Name	Retention Time min	Area mAU*min	Height mAU	Relative Area %	Relative Height %	Amount n.a.
1		8,503	147,712	353,689	31,39	38,75	n.a.
2		10,023	299,756	539,094	63,69	59,06	n.a.
3		22,117	13,730	13,646	2,92	1,50	n.a.
4		31,090	9,432	6,307	2,00	0,69	n.a.
**Total:**			**470,630**	**912,736**	**100,00**	**100,00**	

Double Peak at ~22 min: The impurity peak (first peak) was always present, even after further purification. The seccond peak was assigned as product peak by compariosn of UV(vis spectra.

4-Methylthiophenyl benzo[c]oxepinone derivative (6k)

Chromatogram

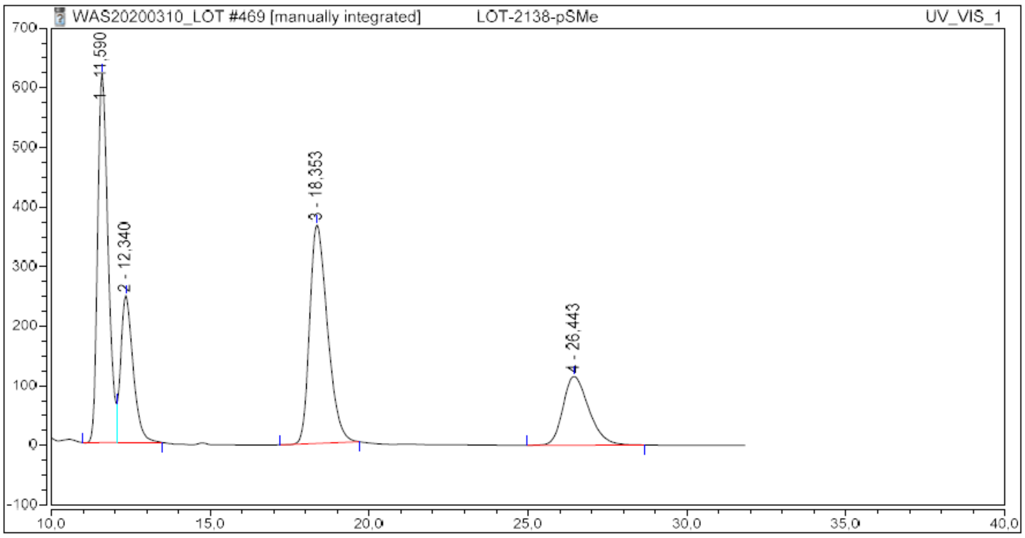

Integration Results

No.	Peak Name	Retention Time min	Area mAU*min	Height mAU	Relative Area %	Relative Height %	Amount n.a.
1		11,590	234,814	619,229	33,78	45,94	n.a.
2		12,340	112,679	246,705	16,21	18,30	n.a.
3		18,353	238,800	366,572	34,35	27,19	n.a.
4		26,443	108,812	115,460	15,65	8,57	n.a.
			**695,105**	**1347,966**	**100,00**	**100,00**	

Chromatogram

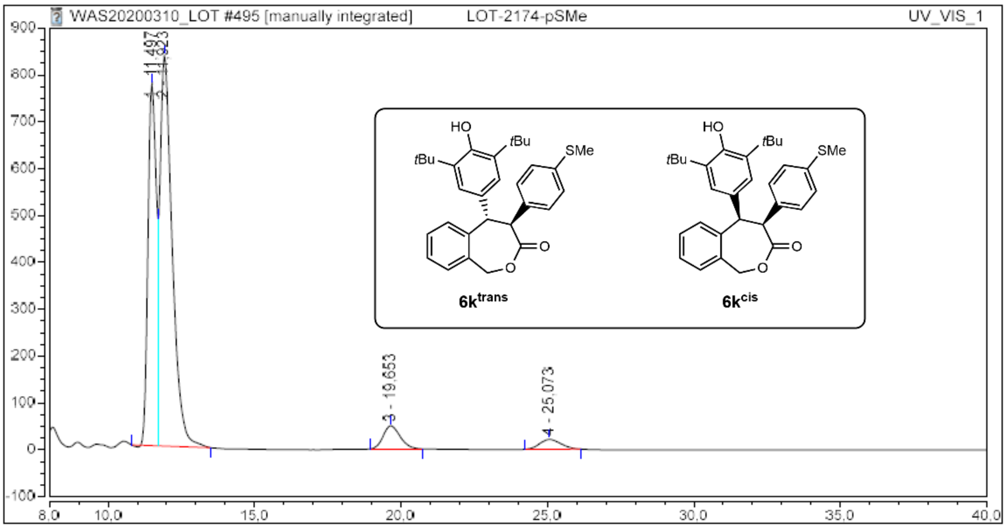

Integration Results

No.	Peak Name	Retention Time min	Area mAU*min	Height mAU	Relative Area %	Relative Height %	Amount n.a.
1		11,497	251,951	771,754	35,59	46,05	n.a.
2		11,923	405,439	834,000	57,26	49,77	n.a.
3		19,653	33,110	49,360	4,68	2,95	n.a.
4		25,073	17,508	20,758	2,47	1,24	n.a.
			**708,008**	**1675,872**	**100,00**	**100,00**	

Due to overlap in the chromatogram, the diastereomers were separated and the measurement was repeated:

Chromatogram

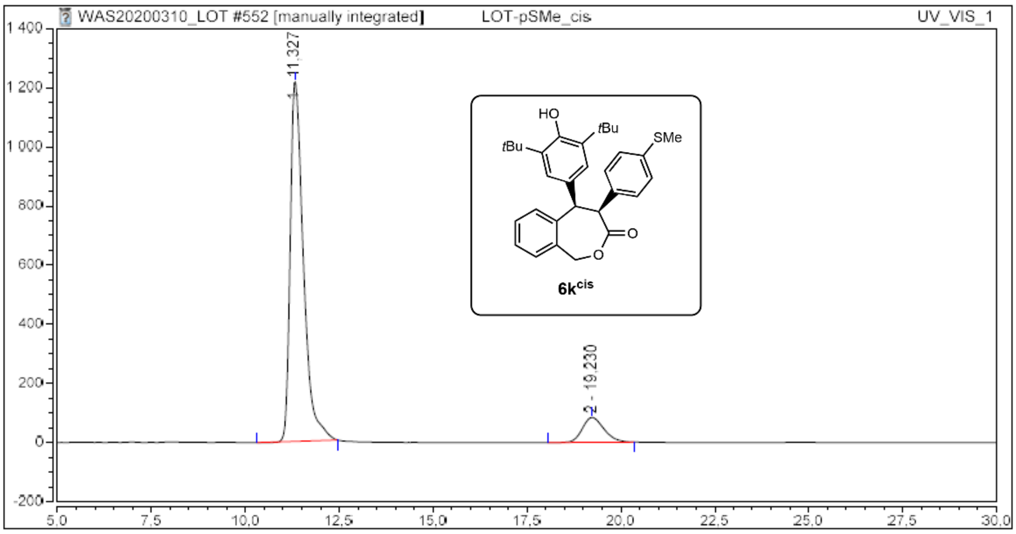

Integration Results

No.	Peak Name	Retention Time min	Area mAU*min	Height mAU	Relative Area %	Relative Height %	Amount n.a.
1		11,327	494,652	1215,788	89,83	93,53	n.a.
2		19,230	56,028	84,044	10,17	6,47	n.a.
			**550,681**	**1299,832**	**100,00**	**100,00**	

Chromatogram

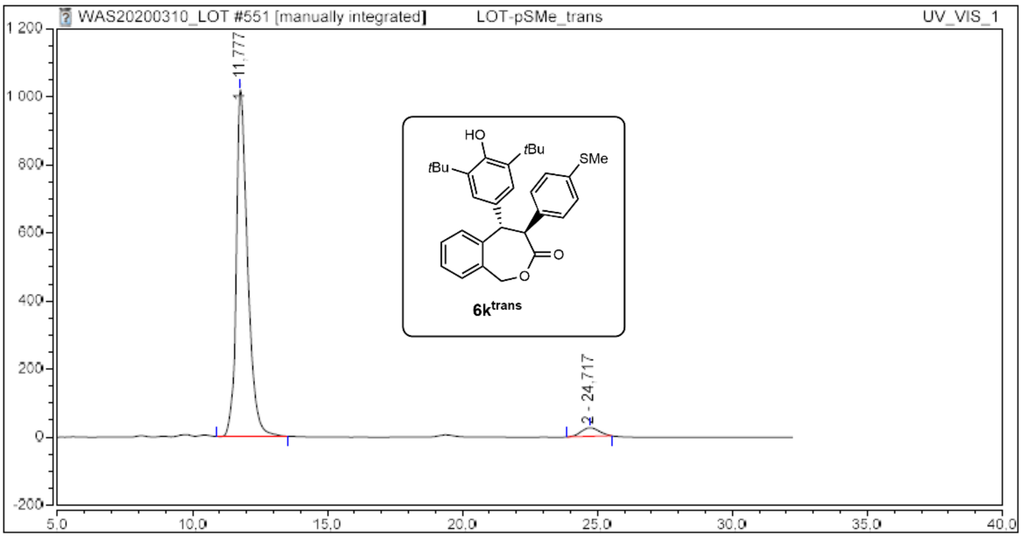

Integration Results

No.	Peak Name	Retention Time min	Area mAU*min	Height mAU	Relative Area %	Relative Height %	Amount n.a.
1		11,777	497,014	1018,190	96,19	97,57	n.a.
2		24,717	19,687	25,315	3,81	2,43	n.a.
			**516,701**	**1043,504**	**100,00**	**100,00**	

2-Methylphenyl benzo[c]oxepinone derivative (6l)

As the dr for the racemic product was 50:50, the diastereomers needed to be separated to assign cis/trans for the peaks in the chromatogram.

Chromatogram

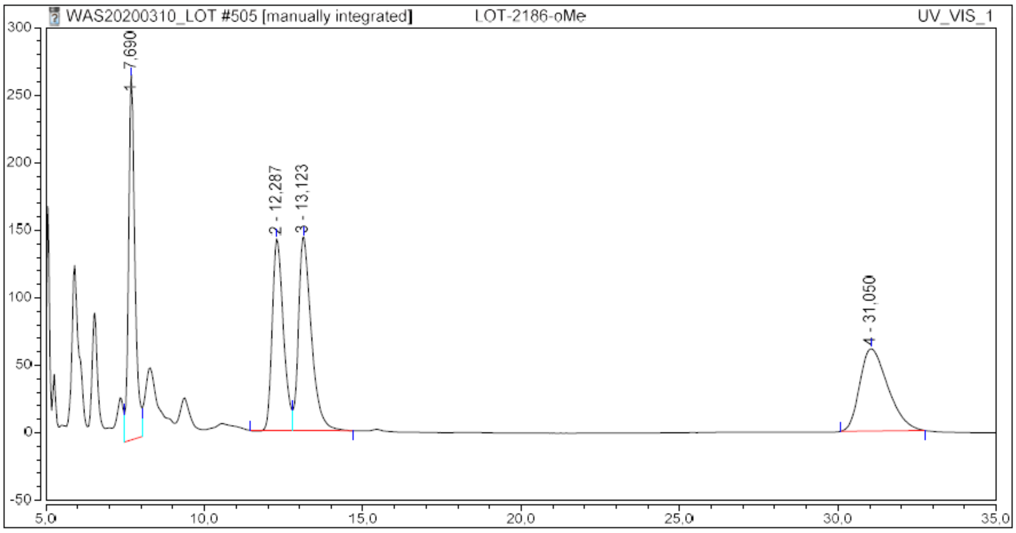

Integration Results

No.	Peak Name	Retention Time min	Area mAU*min	Height mAU	Relative Area %	Relative Height %	Amount n.a.
1		7,690	62,725	268,895	25,00	43,69	n.a.
2		12,287	57,739	142,137	23,01	23,09	n.a.
3		13,123	67,629	143,492	26,95	23,31	n.a.
4		31,050	62,829	60,976	25,04	9,91	n.a.
			**250,923**	**615,500**	**100,00**	**100,00**	

Cis diastereomer (Dirt peak still inside, even after semipreparative HPLC):

Chromatogram

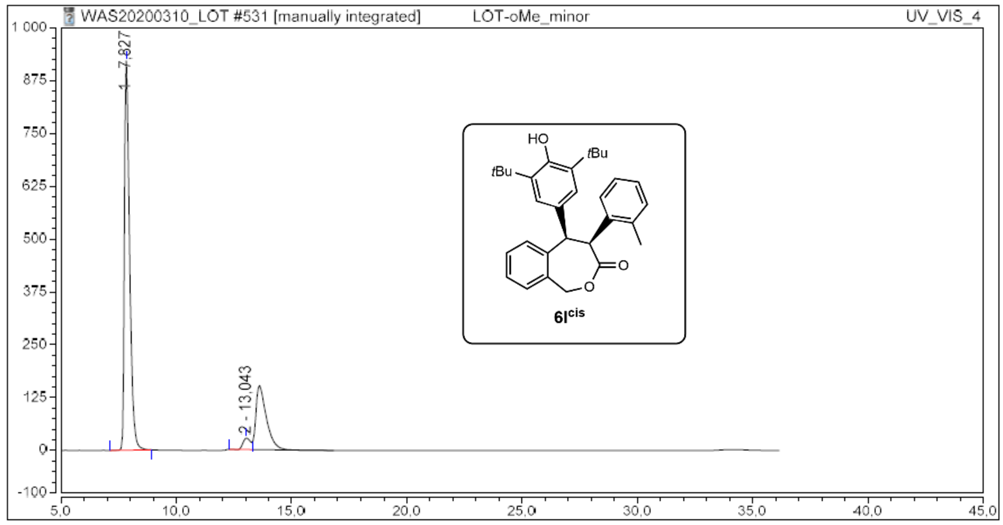

Integration Results

No.	Peak Name	Retention Time min	Area mAU*min	Height mAU	Relative Area %	Relative Height %	Amount n.a.
1		7,827	237,708	921,013	95,90	97,12	n.a.
2		13,043	10,172	27,357	4,10	2,88	n.a.
			**247,880**	**948,371**	**100,00**	**100,00**	

Trans diastereomer:

Chromatogram

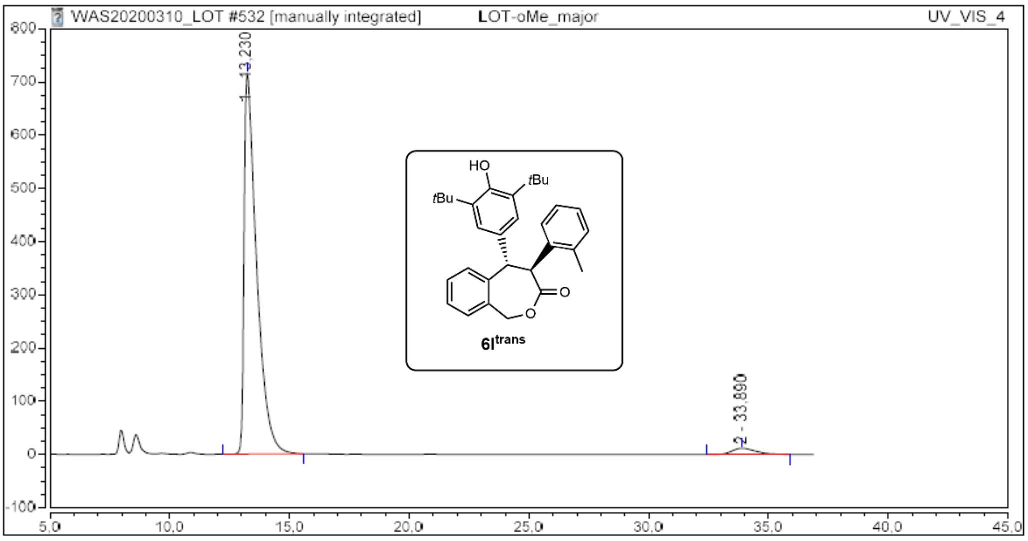

Integration Results

No.	Peak Name	Retention Time min	Area mAU*min	Height mAU	Relative Area %	Relative Height %	Amount n.a.
1		13,230	433,650	715,684	97,01	98,40	n.a.
2		33,890	13,343	11,633	2,99	1,60	n.a.
			**446,992**	**727,316**	**100,00**	**100,00**	

3-Methylphenyl benzo[c]oxepinone derivative (6m)

Chromatogram

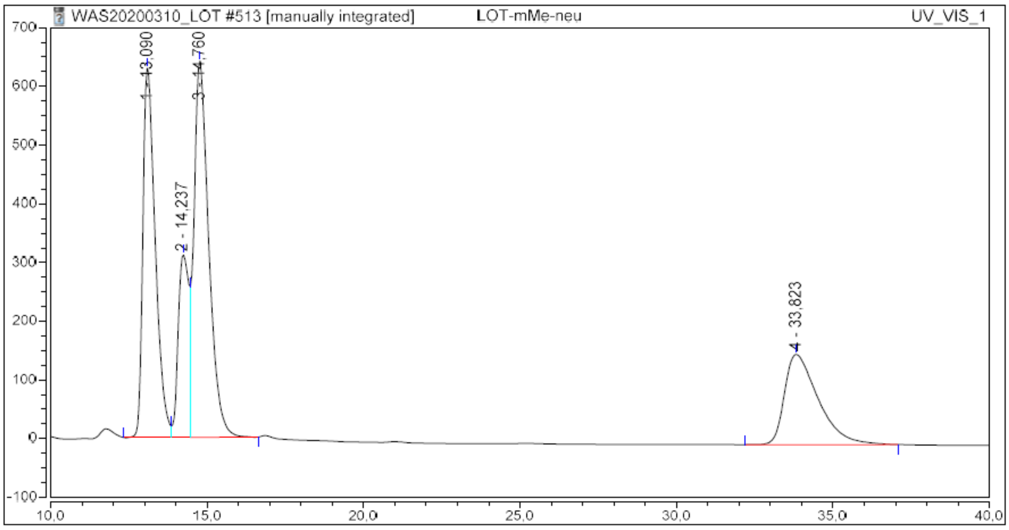

Integration Results

No.	Peak Name	Retention Time min	Area mAU*min	Height mAU	Relative Area %	Relative Height %	Amount n.a.
1		13,090	287,616	628,812	30,13	36,24	n.a.
2		14,237	120,045	310,997	12,58	17,93	n.a.
3		14,760	354,869	641,448	37,18	36,97	n.a.
4		33,823	192,014	153,682	20,12	8,86	n.a.
			**954,544**	**1734,938**	**100,00**	**100,00**	

Due to overlap in the rac-chromatogram, the diastereomers were separated before HPLC measurement.

Cis diastereomer:

Chromatogram

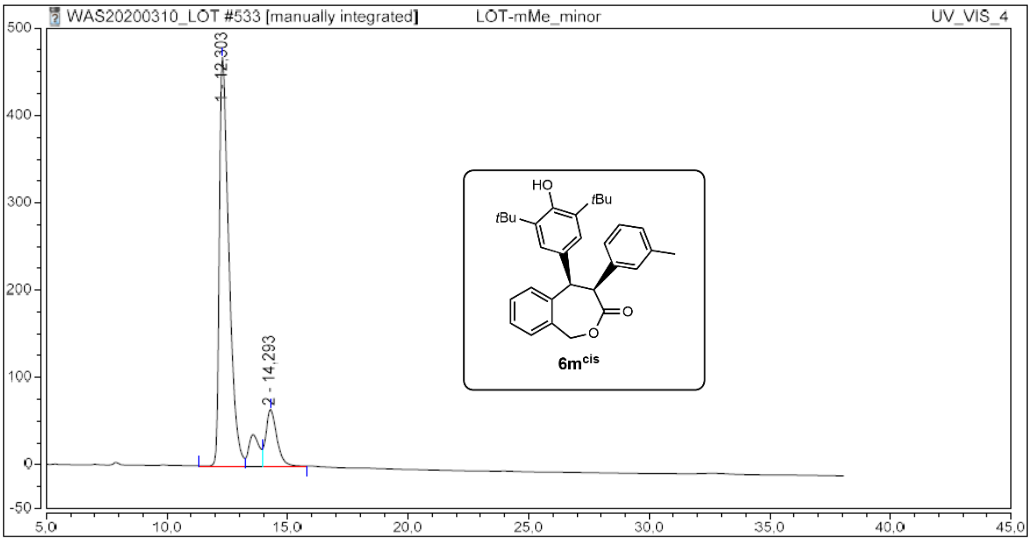

Integration Results

No.	Peak Name	Retention Time min	Area mAU*min	Height mAU	Relative Area %	Relative Height %	Amount n.a.
1		12,303	215,341	467,732	86,41	87,83	n.a.
2		14,293	33,874	64,828	13,59	12,17	n.a.
			**249,215**	**532,559**	**100,00**	**100,00**	

Trans diastereomer:

Chromatogram

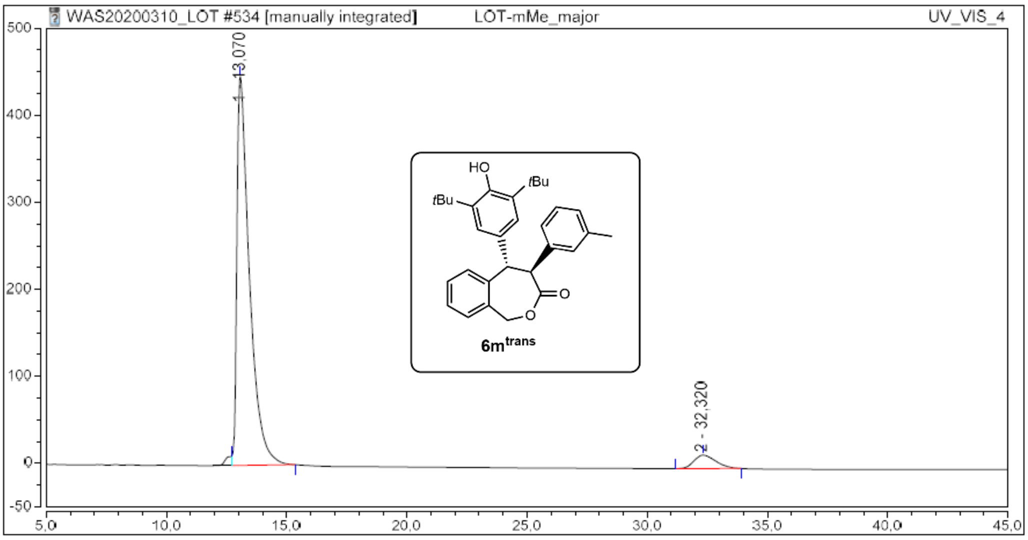

Integration Results

No.	Peak Name	Retention Time min	Area mAU*min	Height mAU	Relative Area %	Relative Height %	Amount n.a.
1		13,070	269,831	446,935	94,26	96,65	n.a.
2		32,320	16,435	15,476	5,74	3,35	n.a.
			**286,266**	**462,412**	**100,00**	**100,00**	

4-Methylphenyl benzo[c]oxepinone derivative (6n)

Chromatogram

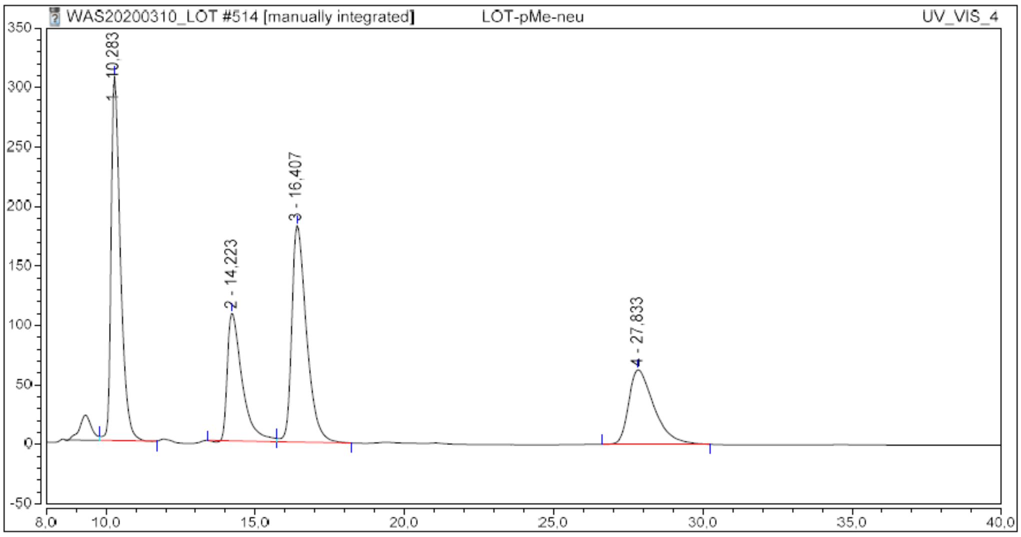

Integration Results

No.	Peak Name	Retention Time<br>min	Area<br>mAU*min	Height<br>mAU	Relative Area<br>%	Relative Height<br>%	Amount<br>n.a.
1		10,283	110,798	306,503	32,74	46,52	n.a.
2		14,223	61,346	107,528	18,13	16,32	n.a.
3		16,407	104,560	182,127	30,90	27,64	n.a.
4		27,833	61,674	62,660	18,23	9,51	n.a.
			**338,378**	**658,818**	**100,00**	**100,00**	

Cis diastereomer:

Chromatogram

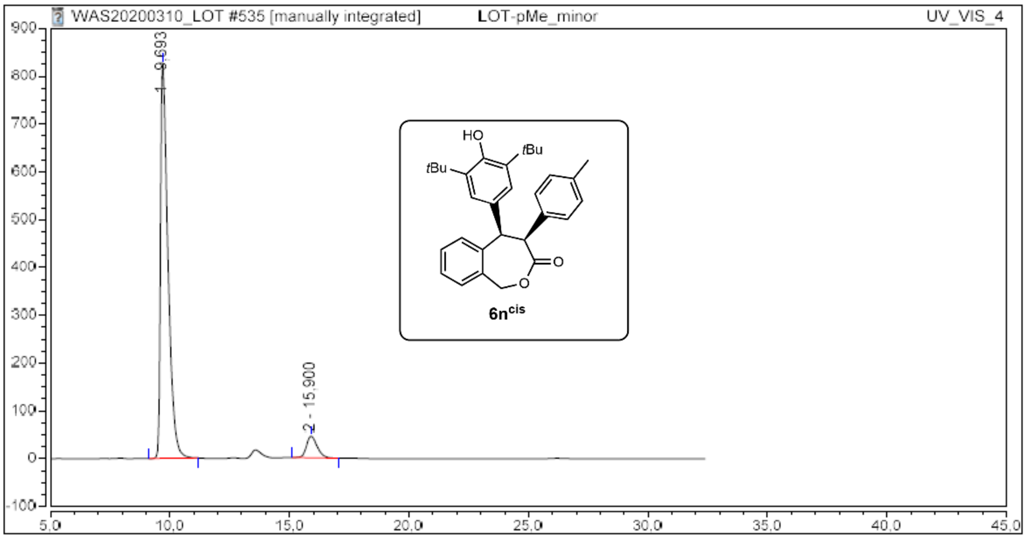

Integration Results

No.	Peak Name	Retention Time<br>min	Area<br>mAU*min	Height<br>mAU	Relative Area<br>%	Relative Height<br>%	Amount<br>n.a.
1		9,693	299,523	826,412	92,74	94,82	n.a.
2		15,900	23,462	45,136	7,26	5,18	n.a.
			**322,985**	**871,548**	**100,00**	**100,00**	

Trans diastereomer:

Chromatogram

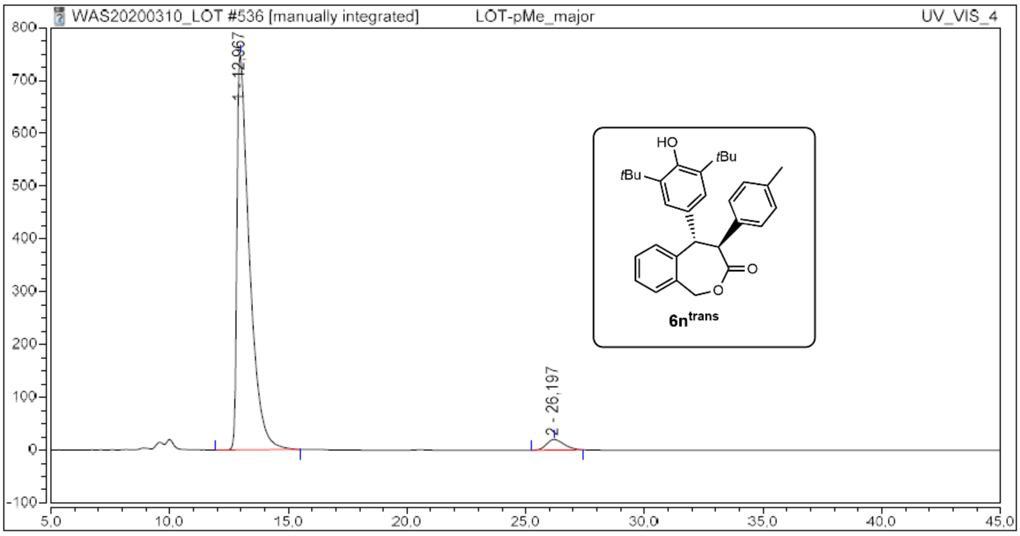

Integration Results

No.	Peak Name	Retention Time min	Area mAU*min	Height mAU	Relative Area %	Relative Height %	Amount n.a.
1		12,967	454,455	748,883	96,41	97,40	n.a.
2		26,197	16,912	19,958	3,59	2,60	n.a.
			**471,367**	**768,841**	**100,00**	**100,00**	

3,5-Dimethylphenyl benzo[c]oxepinone derivative (6o)

Chromatogram

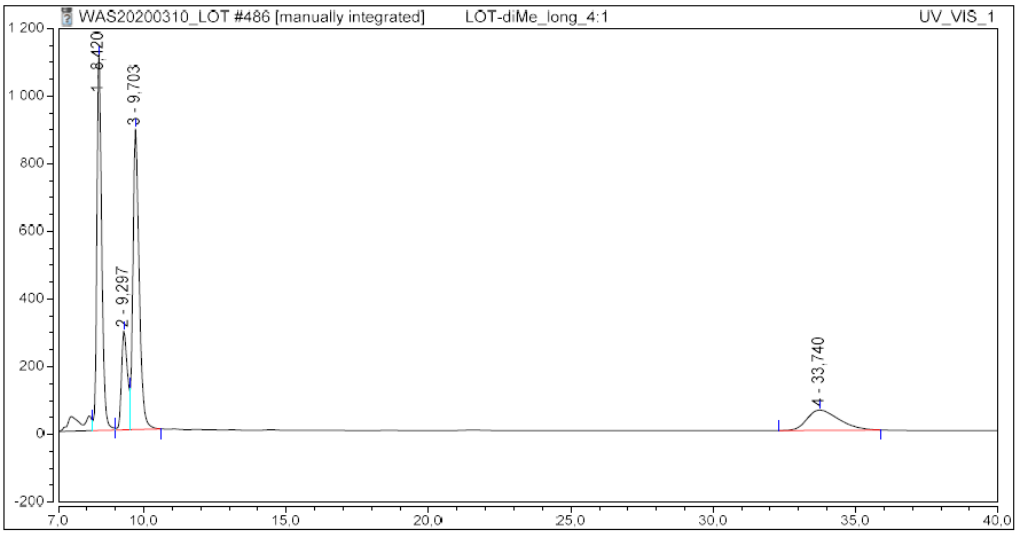

Integration Results

No.	Peak Name	Retention Time min	Area mAU*min	Height mAU	Relative Area %	Relative Height %	Amount n.a.
1		8,420	218,278	1107,296	36,79	47,17	n.a.
2		9,297	72,838	291,889	12,28	12,44	n.a.
3		9,703	224,643	888,373	37,86	37,85	n.a.
4		33,740	77,522	59,701	13,07	2,54	n.a.
			**593,281**	**2347,260**	**100,00**	**100,00**	

Chromatogram

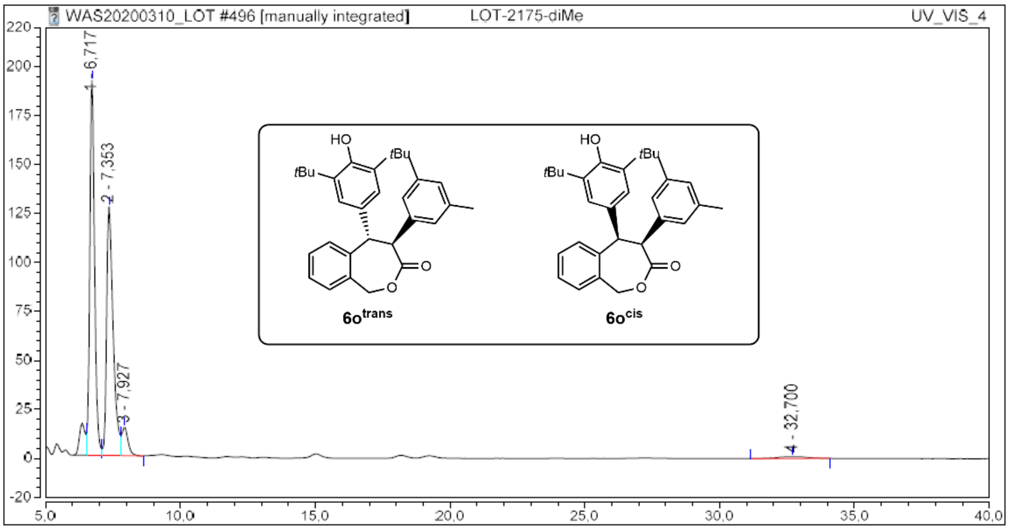

Integration Results

No.	Peak Name	Retention Time min	Area mAU*min	Height mAU	Relative Area %	Relative Height %	Amount n.a.
1		6,717	39,197	191,334	49,02	57,27	n.a.
2		7,353	35,570	127,086	44,48	38,04	n.a.
3		7,927	4,014	14,725	5,02	4,41	n.a.
4		32,700	1,181	0,964	1,48	0,29	n.a.
			**79,963**	**334,109**	**100,00**	**100,00**	

4-*t*-Butylphenyl benzo[c]oxepinone derivative (6p)

Chromatogram

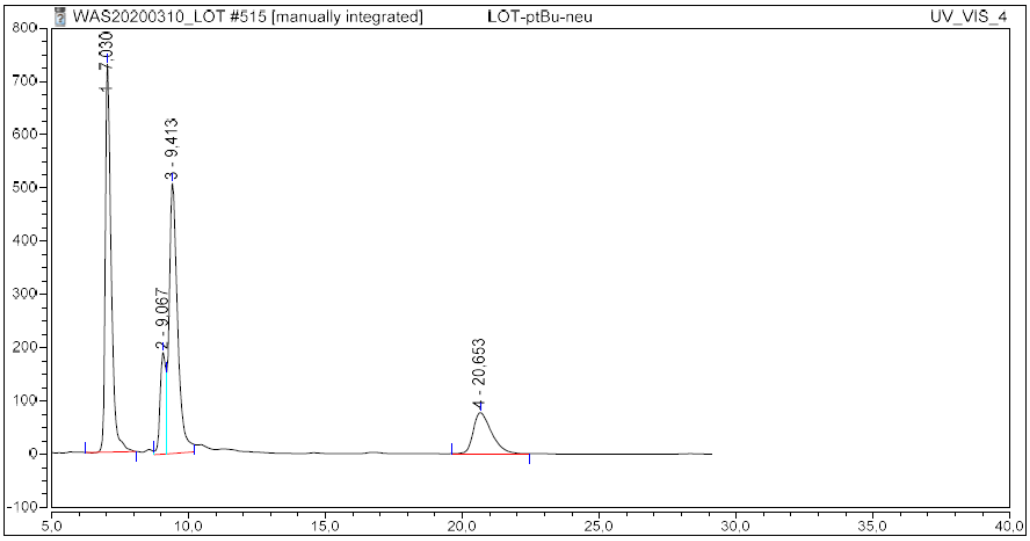

Integration Results

No.	Peak Name	Retention Time<br>min	Area<br>mAU*min	Height<br>mAU	Relative Area<br>%	Relative Height<br>%	Amount<br>n.a.
1		7,030	180,760	728,564	38,77	48,46	n.a.
2		9,067	46,617	190,536	10,00	12,67	n.a.
3		9,413	178,889	507,130	38,37	33,73	n.a.
4		20,653	59,941	77,264	12,86	5,14	n.a.
			**466,207**	**1503,493**	**100,00**	**100,00**	

Chromatogram

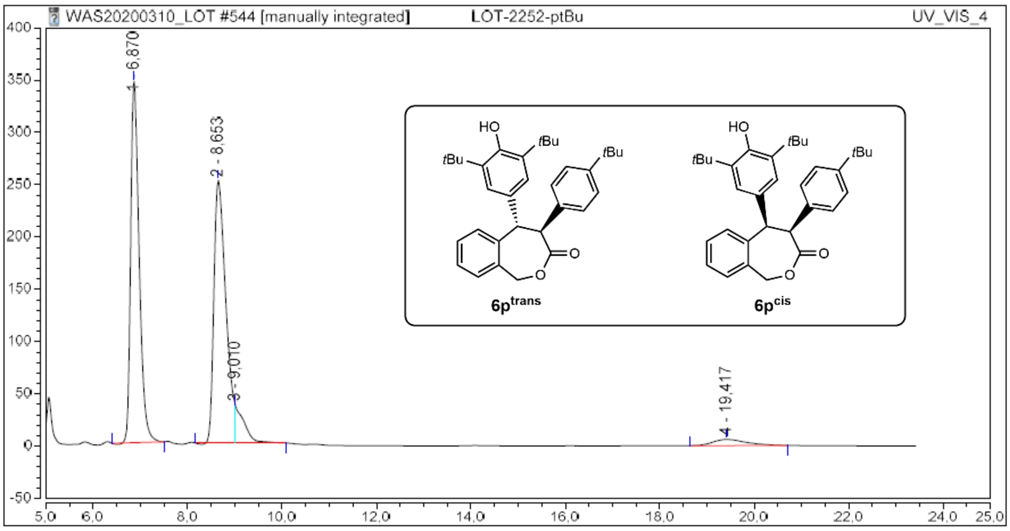

Integration Results

No.	Peak Name	Retention Time<br>min	Area<br>mAU*min	Height<br>mAU	Relative Area<br>%	Relative Height<br>%	Amount<br>n.a.
1		6,870	71,482	345,485	45,19	54,06	n.a.
2		8,653	73,686	251,293	46,58	39,32	n.a.
3		9,010	8,097	36,314	5,12	5,68	n.a.
4		19,417	4,922	5,954	3,11	0,93	n.a.
			**158,187**	**639,046**	**100,00**	**100,00**	

The measurement of the asymmetric sample was repeated with reduced flow rate (0.5 mL min^{-1} instead of 1 mL min^{-1}) to separate the 2nd and 3rd peak better: Using alternative columns did not help.

Chromatogram

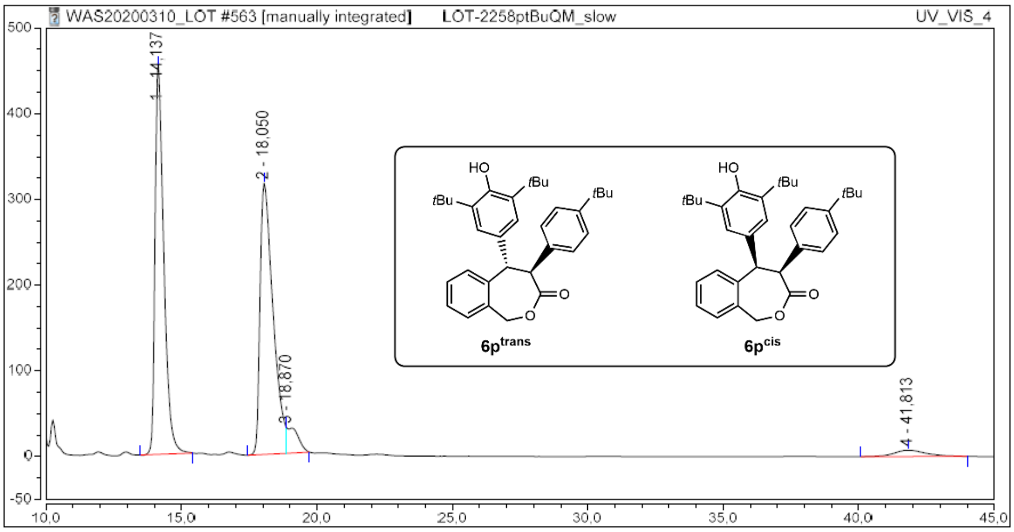

Integration Results

No.	Peak Name	Retention Time min	Area mAU*min	Height mAU	Relative Area %	Relative Height %	Amount n.a.
1		14,137	169,841	452,869	45,86	56,11	n.a.
2		18,050	175,940	316,537	47,50	39,22	n.a.
3		18,870	14,591	30,416	3,94	3,77	n.a.
4		41,813	10,005	7,349	2,70	0,91	n.a.
			**370,376**	**807,171**	**100,00**	**100,00**	

p-Phenyl benzo[c]oxepinone derivative (6q)

Chromatogram

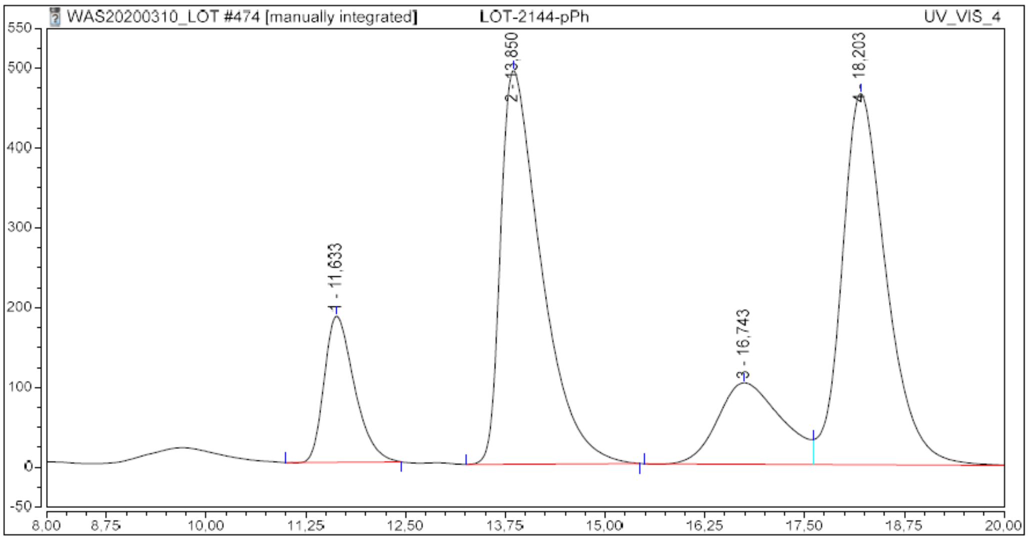

Integration Results

No.	Peak Name	Retention Time min	Area mAU*min	Height mAU	Relative Area %	Relative Height %	Amount n.a.
1		11,633	80,410	183,265	10,48	14,73	n.a.
2		13,850	291,951	493,594	38,07	39,66	n.a.
3		16,743	94,342	102,099	12,30	8,20	n.a.
4		18,203	300,230	465,478	39,15	37,40	n.a.
			**766,933**	**1244,436**	**100,00**	**100,00**	

Chromatogram

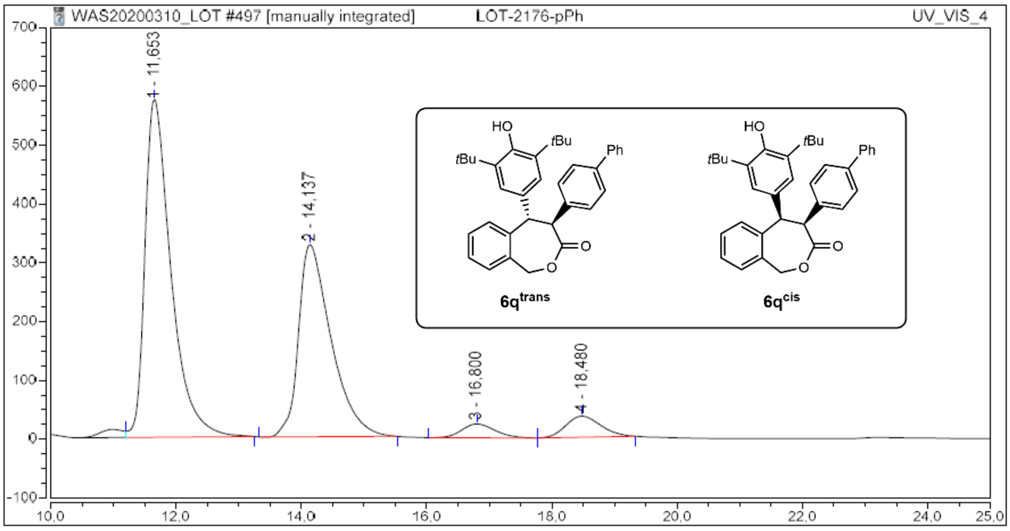

Integration Results

No.	Peak Name	Retention Time min	Area mAU*min	Height mAU	Relative Area %	Relative Height %	Amount n.a.
1		11,653	276,435	574,598	54,16	59,83	n.a.
2		14,137	196,871	326,667	38,57	34,02	n.a.
3		16,800	14,251	23,411	2,79	2,44	n.a.
4		18,480	22,886	35,642	4,48	3,71	n.a.
			**510,444**	**960,318**	**100,00**	**100,00**	

3-Trifluoromethylphenyl benzo[c]oxepinone derivative (6r)

Chromatogram

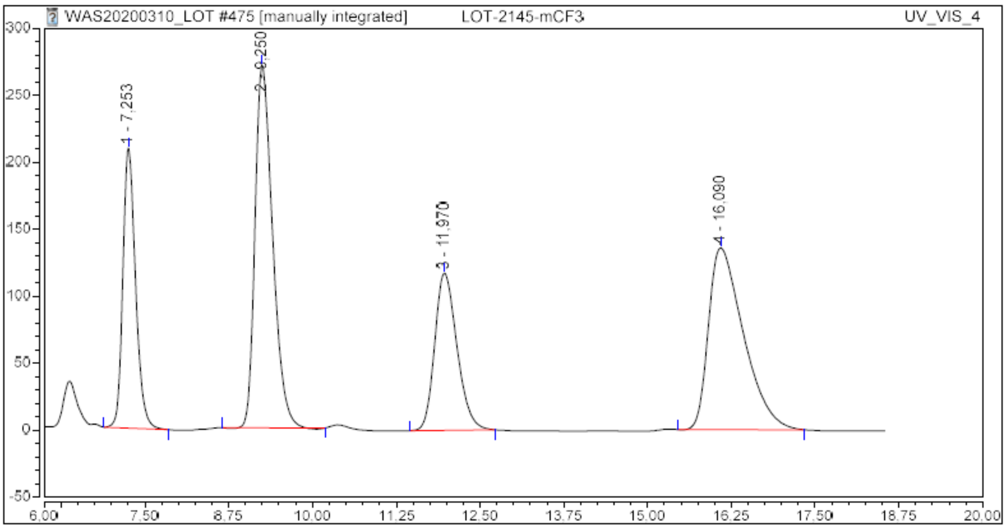

Integration Results

No.	Peak Name	Retention Time min	Area mAU*min	Height mAU	Relative Area %	Relative Height %	Amount n.a.
1		7,253	45,946	209,175	17,98	28,54	n.a.
2		9,250	82,258	270,660	32,20	36,92	n.a.
3		11,970	45,205	117,432	17,69	16,02	n.a.
4		16,090	82,082	135,747	32,13	18,52	n.a.
			**255,491**	**733,014**	**100,00**	**100,00**	

Chromatogram

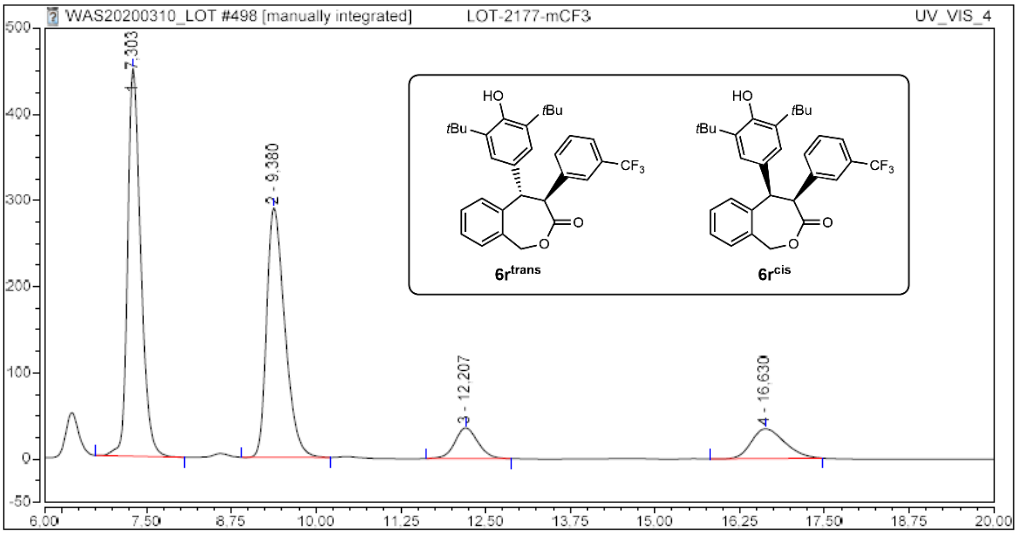

Integration Results

No.	Peak Name	Retention Time min	Area mAU*min	Height mAU	Relative Area %	Relative Height %	Amount n.a.
1		7,303	100,830	449,600	44,91	55,57	n.a.
2		9,380	89,966	289,381	40,07	35,77	n.a.
3		12,207	14,030	35,534	6,25	4,39	n.a.
4		16,630	19,694	34,575	8,77	4,27	n.a.
			**224,520**	**809,089**	**100,00**	**100,00**	

4-Trifluoromethylphenyl benzo[c]oxepinone derivative (6s)

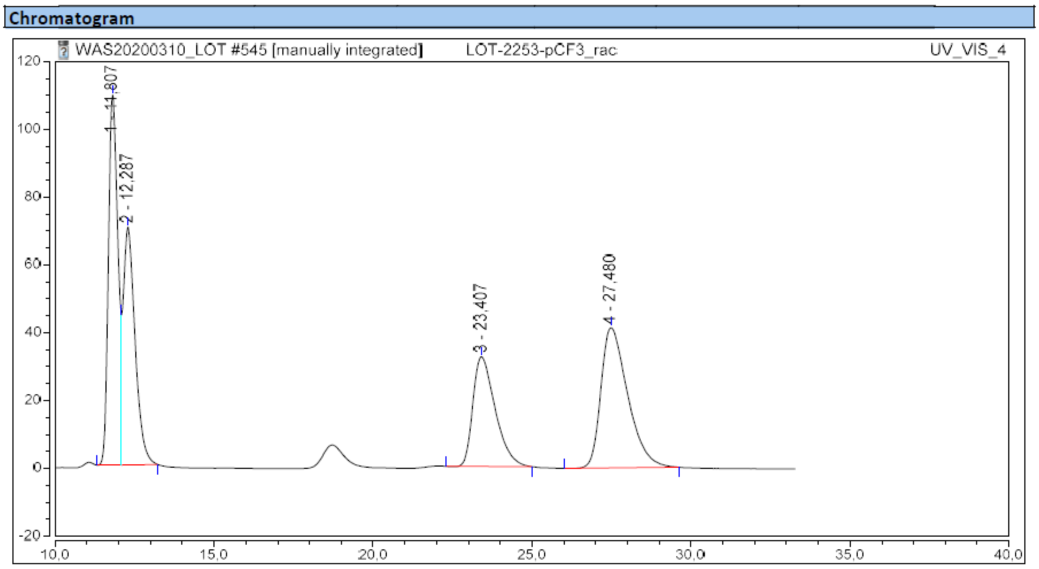

Integration Results							
No.	Peak Name	Retention Time min	Area mAU*min	Height mAU	Relative Area %	Relative Height %	Amount n.a.
1		11,807	37,314	109,141	27,74	43,13	n.a.
2		12,287	30,576	70,110	22,73	27,71	n.a.
3		23,407	26,247	32,384	19,52	12,80	n.a.
4		27,480	40,359	41,388	30,01	16,36	n.a.
			**134,496**	**253,024**	**100,00**	**100,00**	

Due to overlap in the chromatogram, the diastereomers were separated.

Cis Diastereomer:

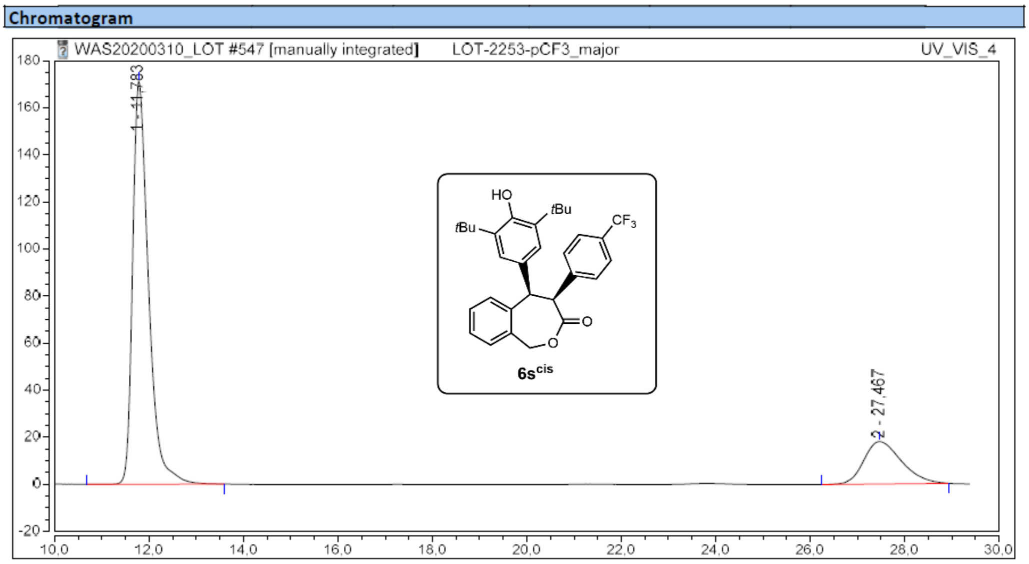

Integration Results							
No.	Peak Name	Retention Time min	Area mAU*min	Height mAU	Relative Area %	Relative Height %	Amount n.a.
1		11,783	66,266	171,181	79,93	90,48	n.a.
2		27,467	16,635	18,021	20,07	9,52	n.a.
			**82,901**	**189,201**	**100,00**	**100,00**	

Trans Diastereomer:

Chromatogram

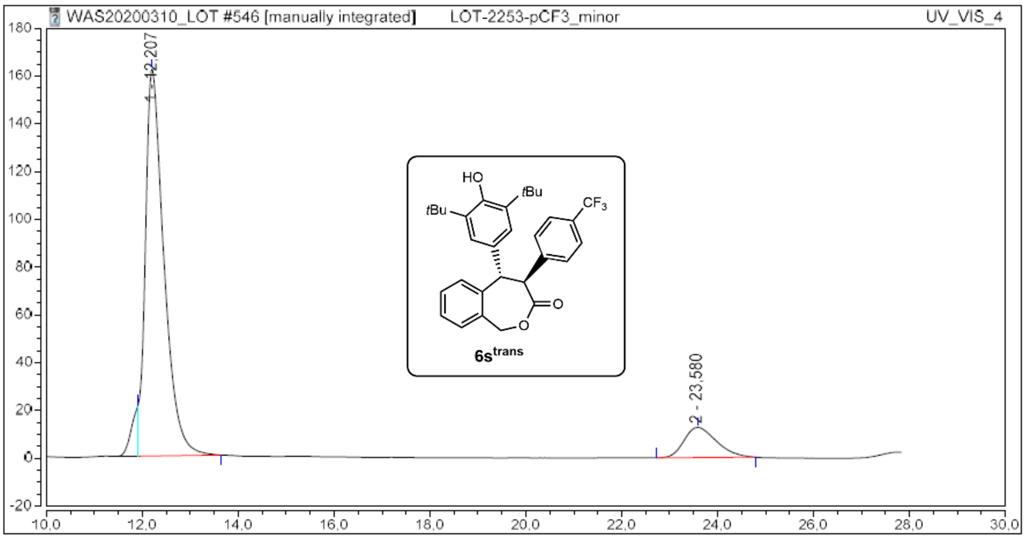

Integration Results

No.	Peak Name	Retention Time min	Area mAU*min	Height mAU	Relative Area %	Relative Height %	Amount n.a.
1		12,207	72,698	161,956	87,99	92,76	n.a.
2		23,580	9,927	12,635	12,01	7,24	n.a.
			**82,626**	**174,591**	**100,00**	**100,00**	

4-Nitrophenyl benzo[c]oxepinone derivative (6t)

Chromatogram

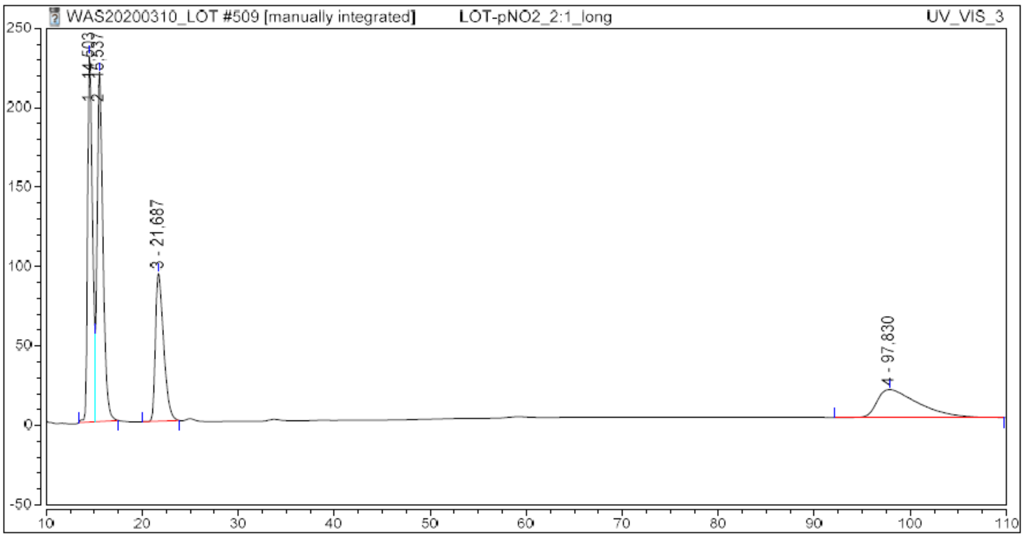

Integration Results

No.	Peak Name	Retention Time min	Area mAU*min	Height mAU	Relative Area %	Relative Height %	Amount n.a.
1		14,503	136,406	230,635	29,12	41,10	n.a.
2		15,537	152,404	219,888	32,54	39,19	n.a.
3		21,687	91,768	92,979	19,59	16,57	n.a.
4		97,830	87,822	17,637	18,75	3,14	n.a.
			**468,399**	**561,139**	**100,00**	**100,00**	

Chromatogram

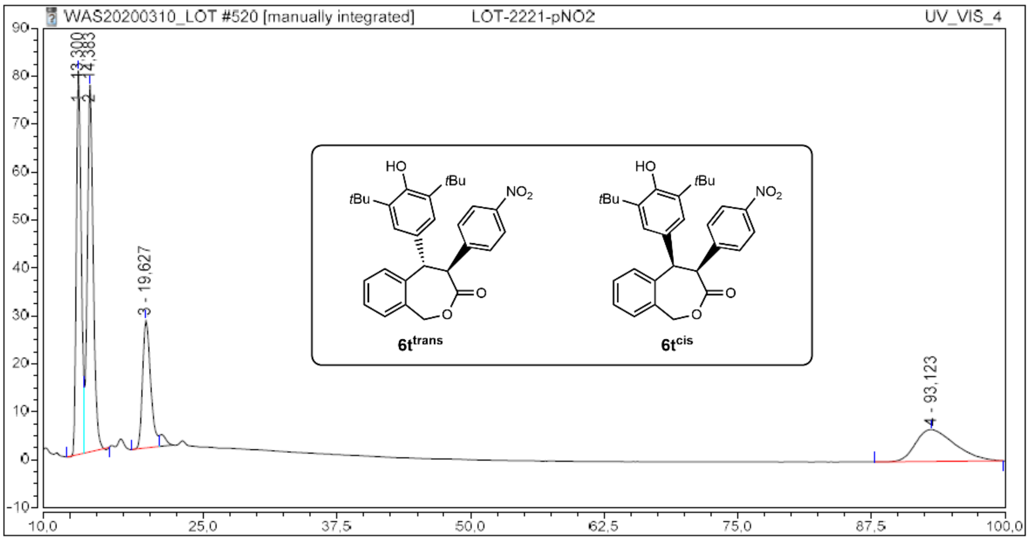

Integration Results

No.	Peak Name	Retention Time min	Area mAU*min	Height mAU	Relative Area %	Relative Height %	Amount n.a.
1		13,300	42,673	80,054	29,88	42,18	n.a.
2		14,383	48,461	76,373	33,94	40,24	n.a.
3		19,627	23,271	26,689	16,30	14,06	n.a.
4		93,123	28,394	6,655	19,88	3,51	n.a.
			**142,799**	**189,771**	**100,00**	**100,00**	

3-Cyanophenyl benzo[c]oxepinone derivative (6u)

Chromatogram

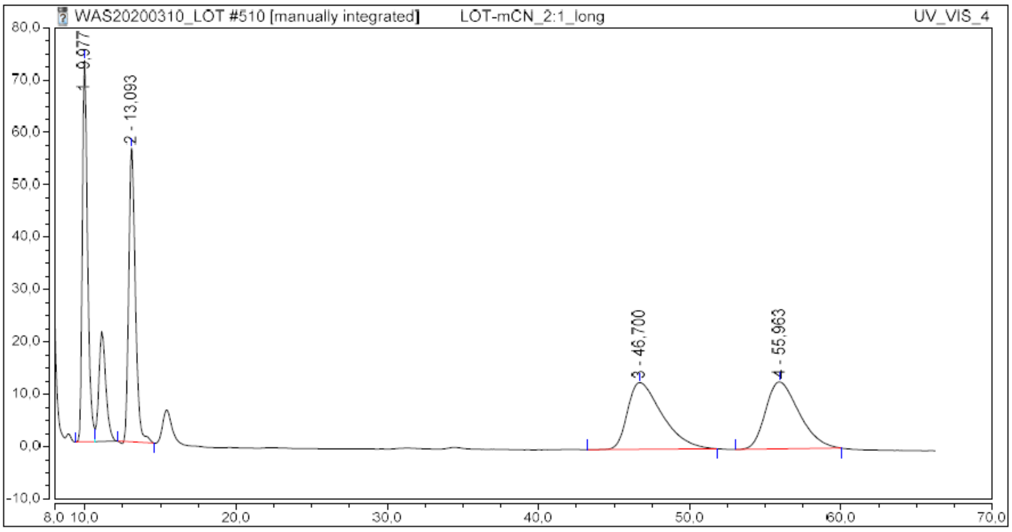

Integration Results

No.	Peak Name	Retention Time<br>min	Area<br>mAU*min	Height<br>mAU	Relative Area<br>%	Relative Height<br>%	Amount<br>n.a.
1		9,977	29,146	72,832	23,44	47,14	n.a.
2		13,093	28,575	56,109	22,99	36,32	n.a.
3		46,700	33,694	12,744	27,10	8,25	n.a.
4		55,963	32,901	12,800	26,47	8,29	n.a.
			**124,316**	**154,485**	**100,00**	**100,00**	

Chromatogram

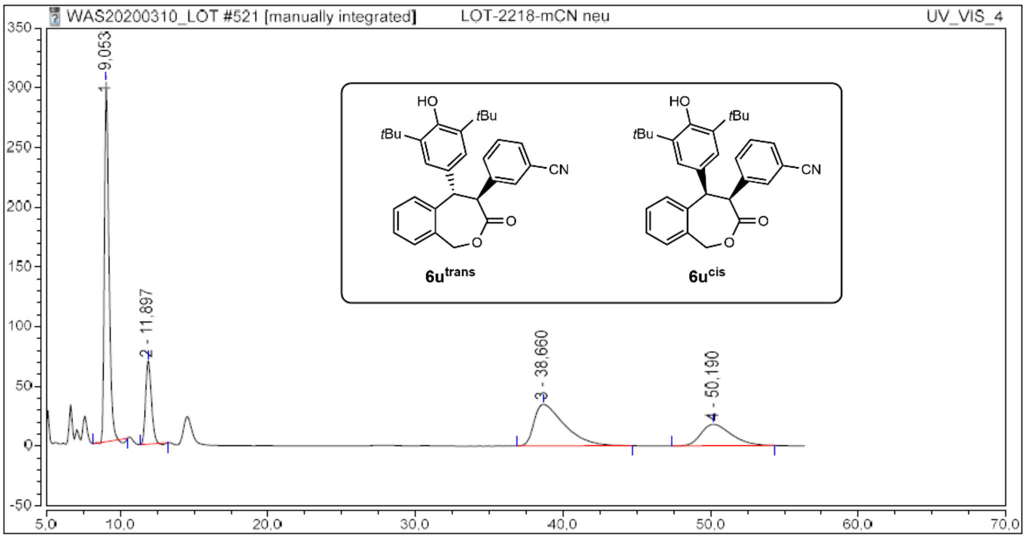

Integration Results

No.	Peak Name	Retention Time<br>min	Area<br>mAU*min	Height<br>mAU	Relative Area<br>%	Relative Height<br>%	Amount<br>n.a.
1		9,053	106,651	300,965	40,47	71,07	n.a.
2		11,897	31,237	69,775	11,85	16,48	n.a.
3		38,660	82,625	34,776	31,35	8,21	n.a.
4		50,190	43,021	17,990	16,32	4,25	n.a.
			**263,535**	**423,506**	**100,00**	**100,00**	

α-Naphthyl benzo[c]oxepinone derivative (6v)

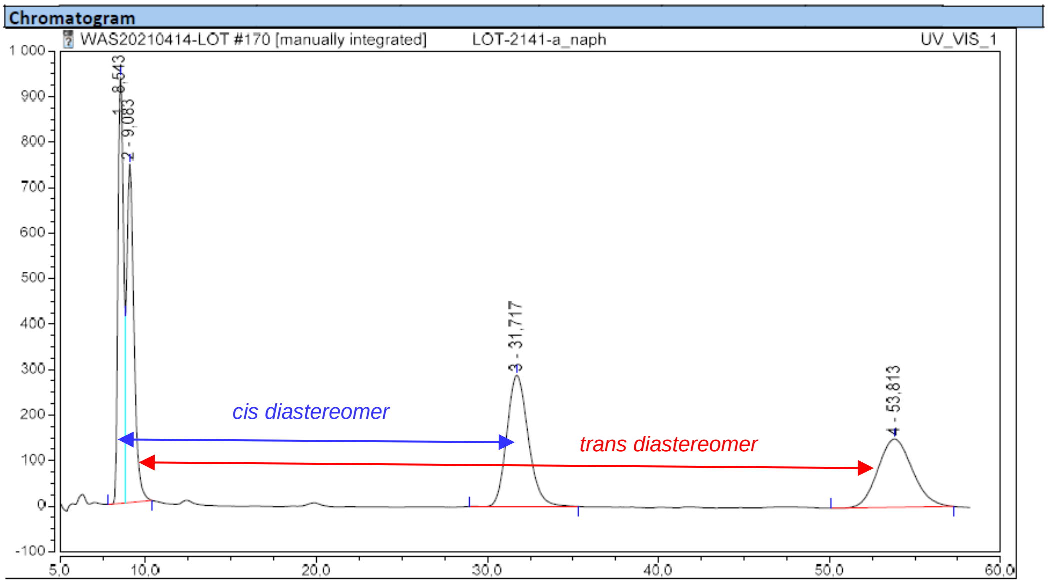

Integration Results							
No.	Peak Name	Retention Time min	Area mAU*min	Height mAU	Relative Area %	Relative Height %	Amount n.a.
1		8,543	354,557	937,660	23,80	44,20	n.a.
2		9,083	364,799	744,504	24,49	35,10	n.a.
3		31,717	419,724	288,948	28,17	13,62	n.a.
4		53,813	350,772	150,155	23,54	7,08	n.a.
**Total:**			**1489,853**	**2121,267**	**100,00**	**100,00**	

Due to a 50:50 ratio of diastereomers in the racemic sample and the diastereomers not being separable, the peaks were assigned by looking at similarities in UV/vis spectra. Also, in the asymmetric sample, the dr matched the dr from the NMR spectrum.

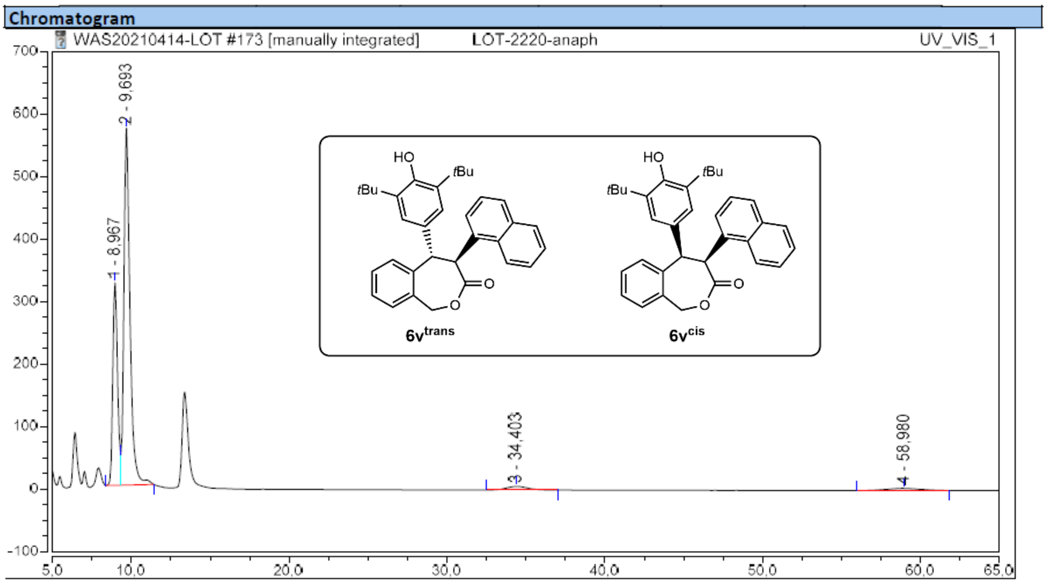

Integration Results							
No.	Peak Name	Retention Time min	Area mAU*min	Height mAU	Relative Area %	Relative Height %	Amount n.a.
1		8,967	112,794	324,151	29,31	35,86	n.a.
2		9,693	255,139	570,863	66,30	63,15	n.a.
3		34,403	8,780	5,744	2,28	0,64	n.a.
4		58,980	8,107	3,274	2,11	0,36	n.a.
**Total:**			**384,820**	**904,031**	**100,00**	**100,00**	

β-Naphthyl benzo[c]oxepinone derivative (6w)

Chromatogram

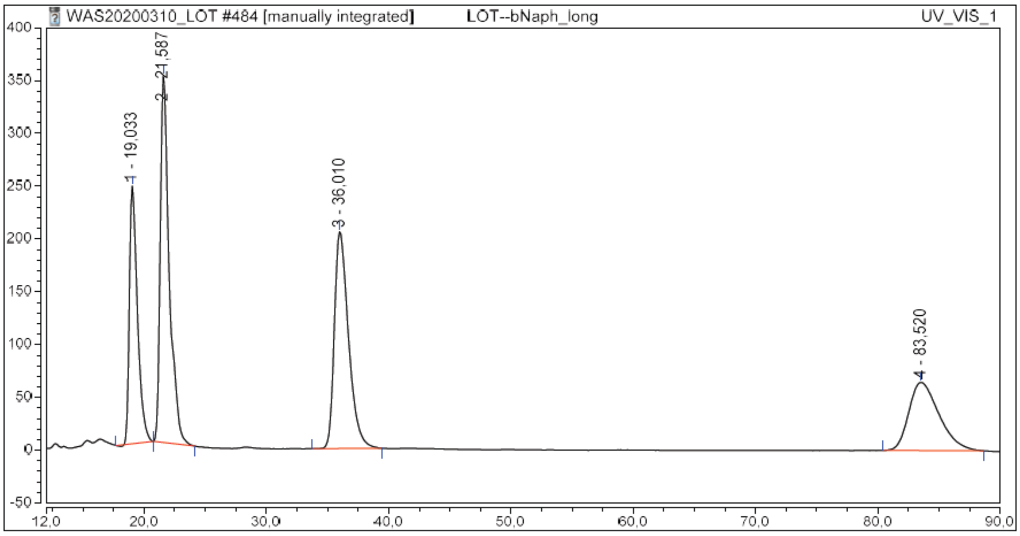

Integration Results

No.	Peak Name	Retention Time min	Area mAU*min	Height mAU	Relative Area %	Relative Height %	Amount n.a.
1		19,033	186,286	243,951	19,79	28,32	n.a.
2		21,587	296,319	347,328	31,47	40,32	n.a.
3		36,010	274,509	205,676	29,16	23,88	n.a.
4		83,520	184,407	64,475	19,59	7,48	n.a.
			**941,521**	**861,430**	**100,00**	**100,00**	

Chromatogram

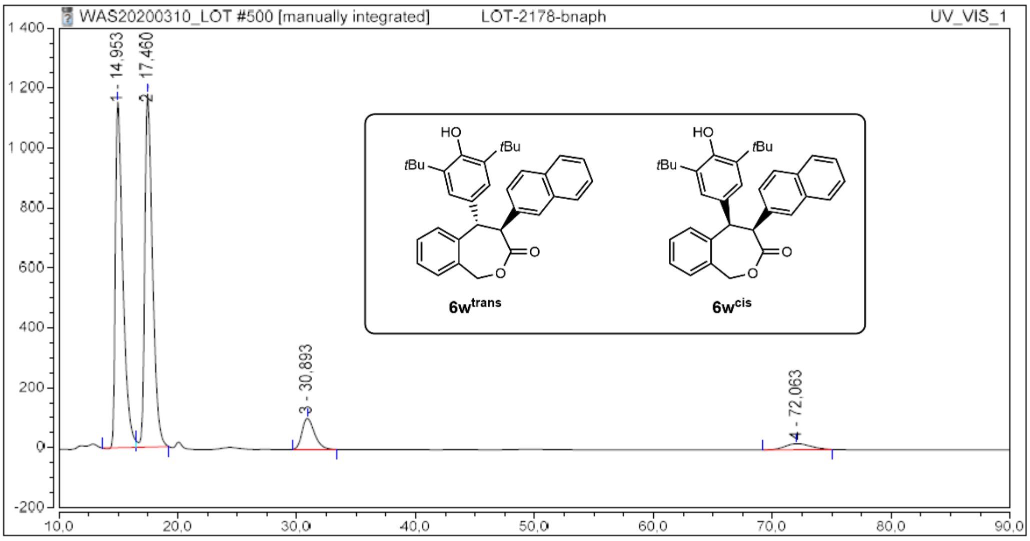

Integration Results

No.	Peak Name	Retention Time min	Area mAU*min	Height mAU	Relative Area %	Relative Height %	Amount n.a.
1		14,953	844,618	1153,342	44,20	46,95	n.a.
2		17,460	889,477	1178,518	46,55	47,98	n.a.
3		30,893	126,575	104,391	6,62	4,25	n.a.
4		72,063	50,124	20,227	2,62	0,82	n.a.
			**1910,795**	**2456,478**	**100,00**	**100,00**	

Thiophen-3-yl benzo[c]oxepinone derivative (6x)

Chromatogram

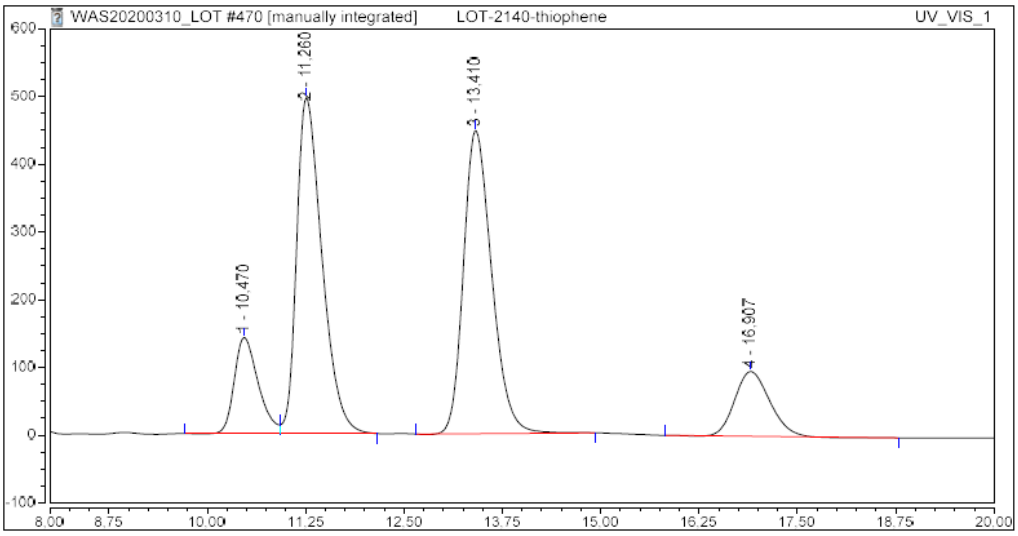

Integration Results

No.	Peak Name	Retention Time min	Area mAU*min	Height mAU	Relative Area %	Relative Height %	Amount n.a.
1		10,470	49,850	141,859	10,61	12,01	n.a.
2		11,260	185,354	495,827	39,44	41,99	n.a.
3		13,410	186,020	447,710	39,58	37,91	n.a.
4		16,907	48,760	95,459	10,37	8,08	n.a.
			**469,984**	**1180,855**	**100,00**	**100,00**	

Chromatogram

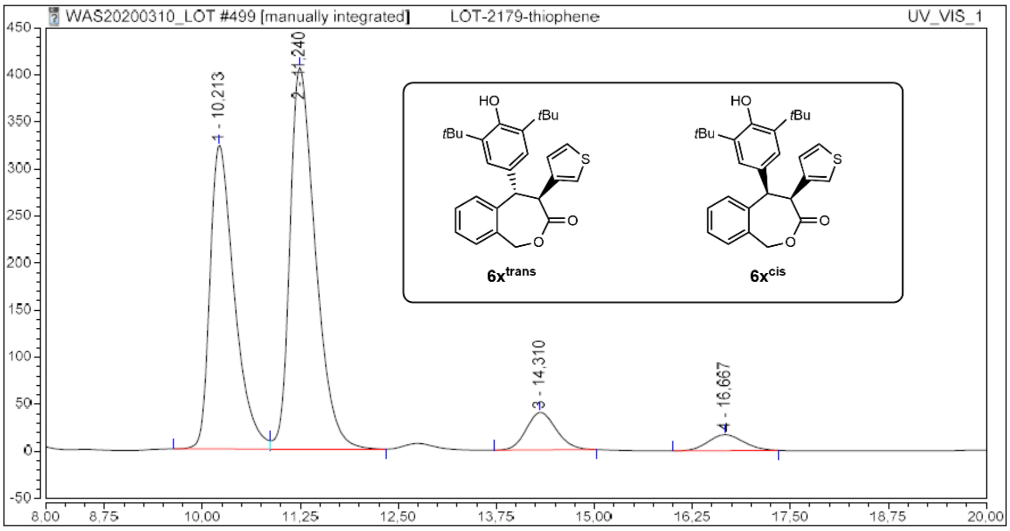

Integration Results

No.	Peak Name	Retention Time min	Area mAU*min	Height mAU	Relative Area %	Relative Height %	Amount n.a.
1		10,213	116,379	323,025	39,30	41,14	n.a.
2		11,240	153,226	405,448	51,75	51,64	n.a.
3		14,310	17,730	39,844	5,99	5,07	n.a.
4		16,667	8,776	16,845	2,96	2,15	n.a.
			**296,111**	**785,163**	**100,00**	**100,00**	

7-Fluoro benzo[c]oxepinone derivative (6aa)

Chromatogram

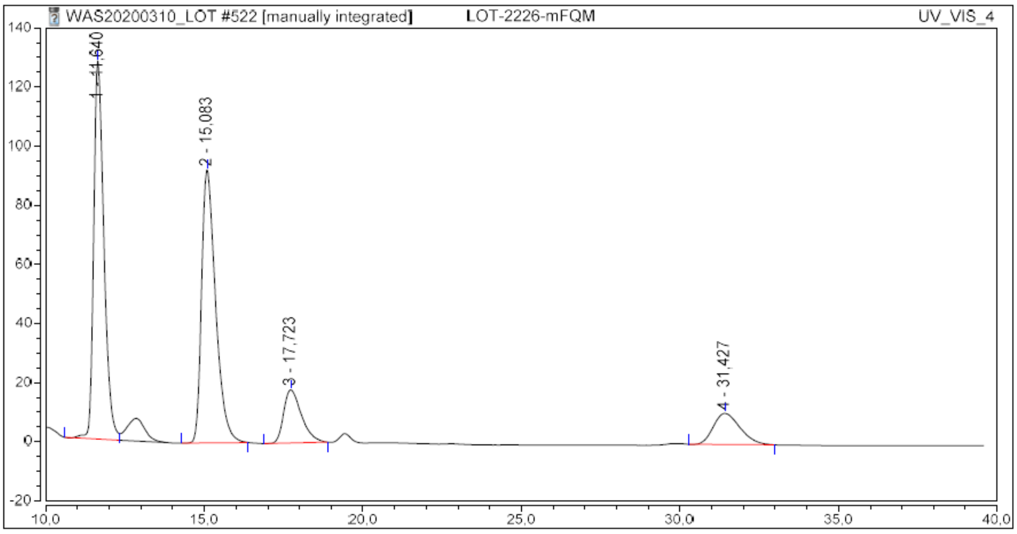

Integration Results

No.	Peak Name	Retention Time min	Area mAU*min	Height mAU	Relative Area %	Relative Height %	Amount n.a.
1		11,640	47,571	127,919	40,73	51,39	n.a.
2		15,083	47,235	92,431	40,45	37,14	n.a.
3		17,723	11,654	18,009	9,98	7,24	n.a.
4		31,427	10,324	10,541	8,84	4,24	n.a.
			**116,783**	**248,900**	**100,00**	**100,00**	

Chromatogram

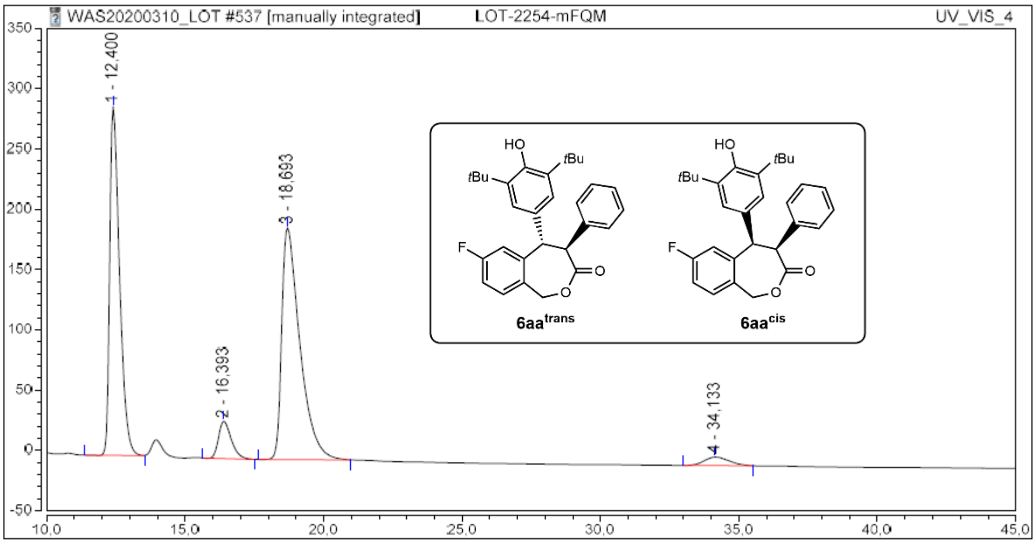

Integration Results

No.	Peak Name	Retention Time min	Area mAU*min	Height mAU	Relative Area %	Relative Height %	Amount n.a.
1		12,400	118,627	289,257	41,14	55,67	n.a.
2		16,393	16,477	30,965	5,71	5,96	n.a.
3		18,693	145,993	192,308	50,63	37,01	n.a.
4		34,133	7,258	7,017	2,52	1,35	n.a.
			**288,355**	**519,546**	**100,00**	**100,00**	

8-Fluoro benzo[c]oxepinone derivative (6ab)

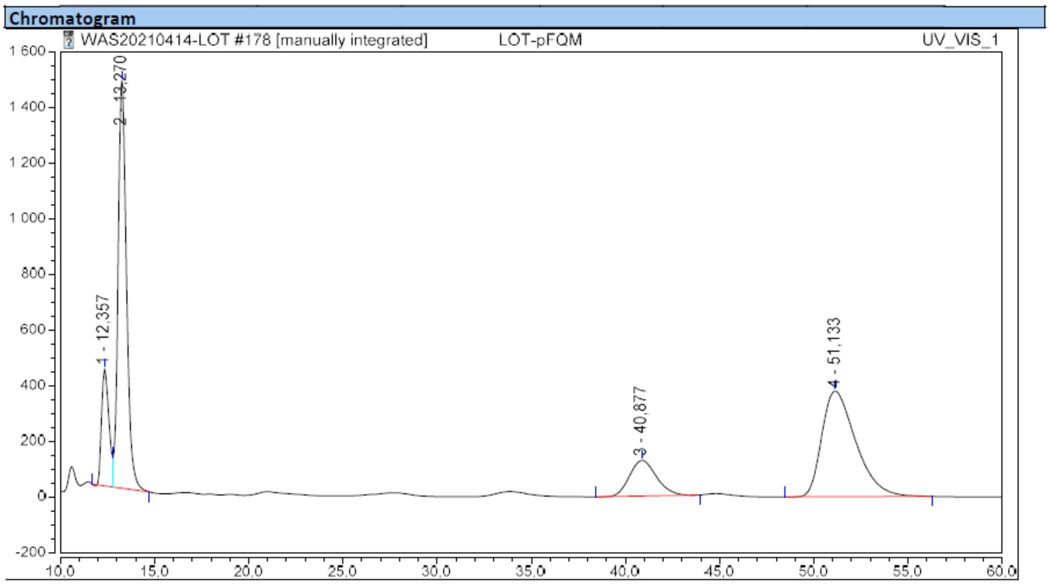

Integration Results

No.	Peak Name	Retention Time min	Area mAU*min	Height mAU	Relative Area %	Relative Height %	Amount n.a.
1		12,357	190,931	420,528	9,78	17,58	n.a.
2		13,270	753,543	1464,526	38,60	61,21	n.a.
3		40,877	211,201	127,886	10,82	5,35	n.a.
4		51,133	796,290	379,614	40,79	15,87	n.a.
**Total:**			**1951,965**	**2392,553**	**100,00**	**100,00**	

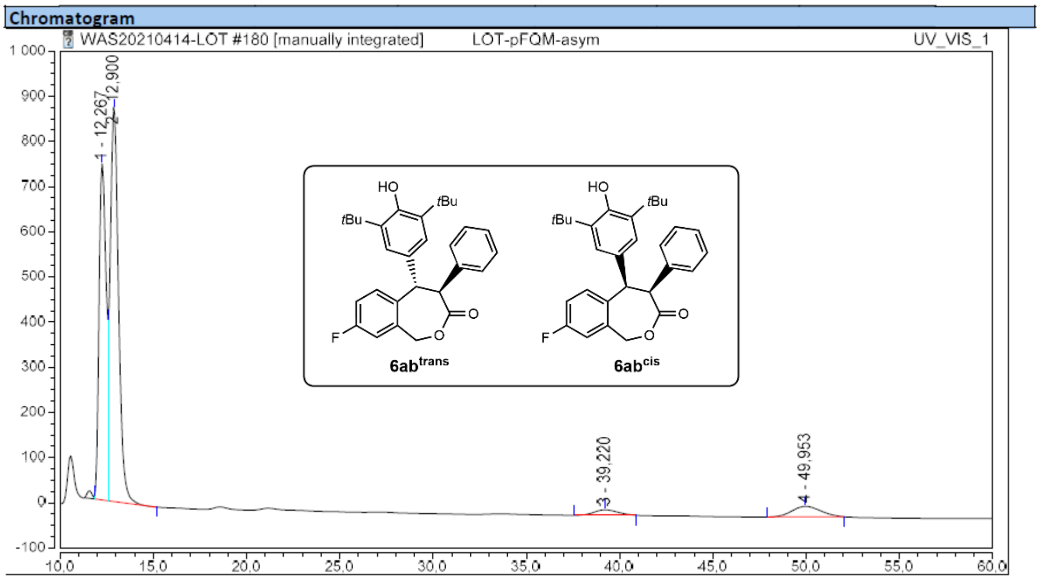

Integration Results

No.	Peak Name	Retention Time min	Area mAU*min	Height mAU	Relative Area %	Relative Height %	Amount n.a.
1		12,267	325,792	744,816	38,87	45,15	n.a.
2		12,900	453,570	869,756	54,11	52,72	n.a.
3		39,220	17,445	12,000	2,08	0,73	n.a.
4		49,953	41,381	23,143	4,94	1,40	n.a.
**Total:**			**838,187**	**1649,714**	**100,00**	**100,00**	

7-Chloro benzo[c]oxepinone derivative (6ba)

Chromatogram

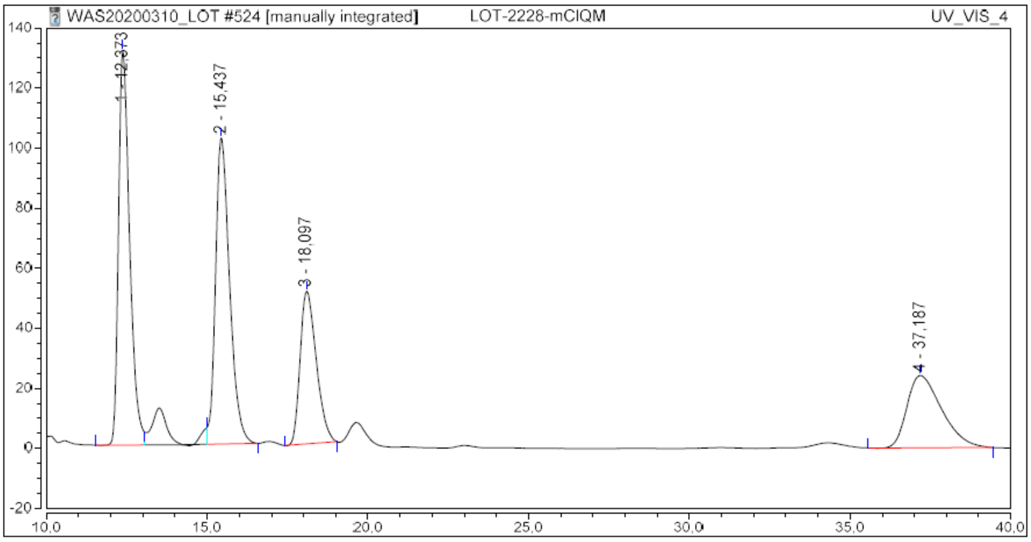

Integration Results

No.	Peak Name	Retention Time min	Area mAU*min	Height mAU	Relative Area %	Relative Height %	Amount n.a.
1		12,373	52,835	131,508	32,16	42,64	n.a.
2		15,437	51,806	101,938	31,54	33,05	n.a.
3		18,097	29,619	50,890	18,03	16,50	n.a.
4		37,187	30,014	24,079	18,27	7,81	n.a.
			**164,274**	**308,414**	**100,00**	**100,00**	

Chromatogram

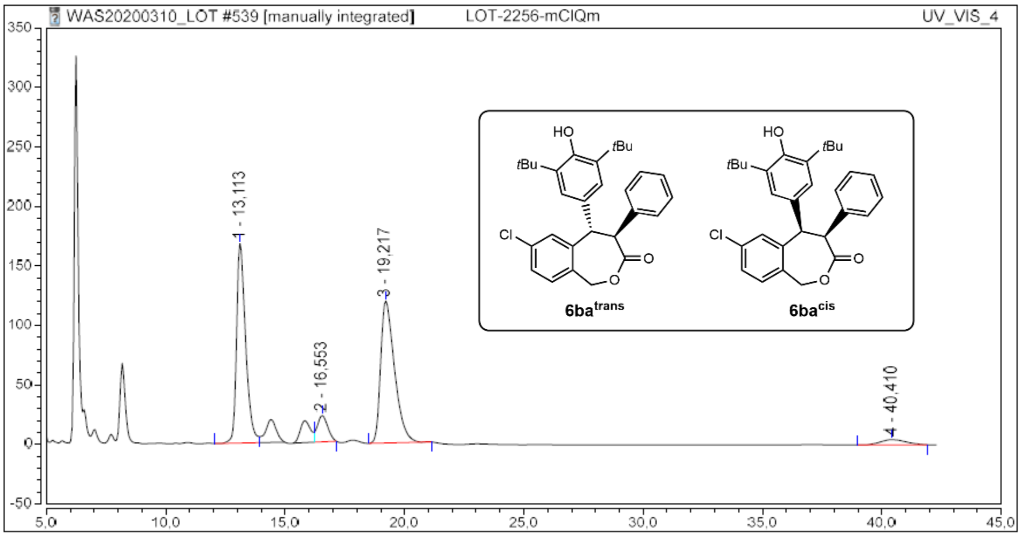

Integration Results

No.	Peak Name	Retention Time min	Area mAU*min	Height mAU	Relative Area %	Relative Height %	Amount n.a.
1		13,113	73,982	167,641	43,31	53,51	n.a.
2		16,553	11,172	22,110	6,54	7,06	n.a.
3		19,217	80,217	119,187	46,96	38,04	n.a.
4		40,410	5,434	4,353	3,18	1,39	n.a.
			**170,806**	**313,291**	**100,00**	**100,00**	

8-Chloro benzo[c]oxepinone derivative (6bb)

Chromatogram

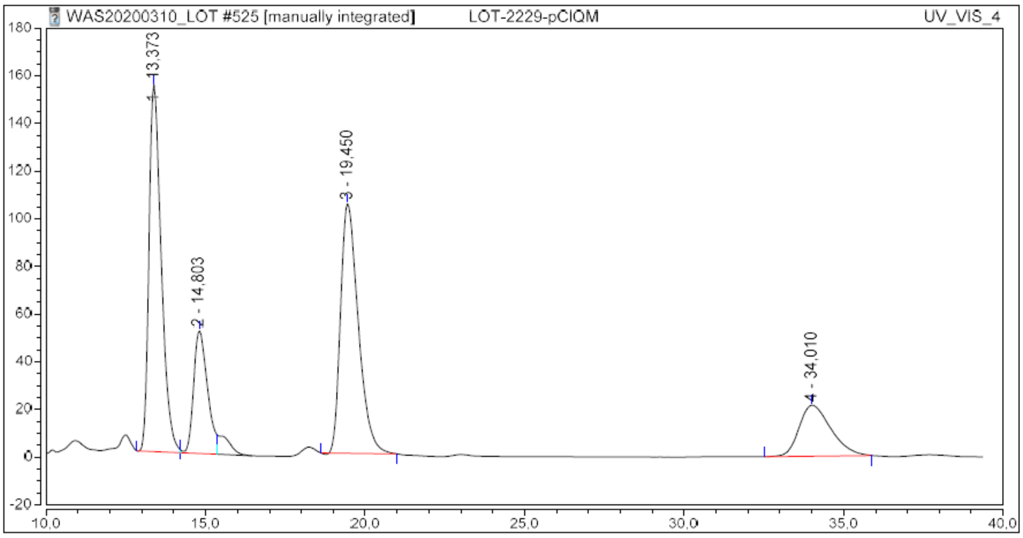

Integration Results

No.	Peak Name	Retention Time min	Area mAU*min	Height mAU	Relative Area %	Relative Height %	Amount n.a.
1		13,373	68,455	153,531	36,76	46,38	n.a.
2		14,803	25,641	51,458	13,77	15,54	n.a.
3		19,450	67,429	104,708	36,21	31,63	n.a.
4		34,010	24,676	21,354	13,25	6,45	n.a.
			**186,201**	**331,050**	**100,00**	**100,00**	

Chromatogram

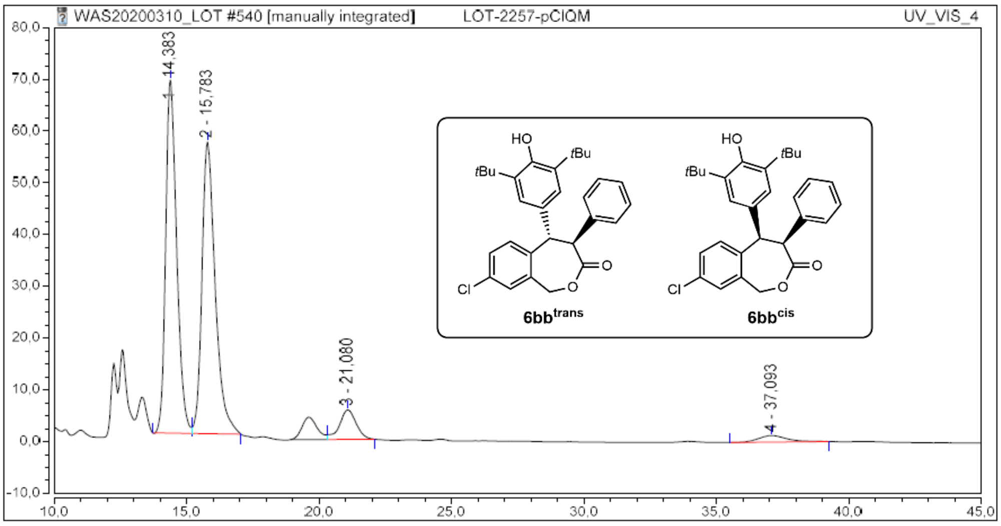

Integration Results

No.	Peak Name	Retention Time min	Area mAU*min	Height mAU	Relative Area %	Relative Height %	Amount n.a.
1		14,383	32,732	68,197	46,03	51,86	n.a.
2		15,783	32,673	56,355	45,95	42,86	n.a.
3		21,080	4,138	5,701	5,82	4,34	n.a.
4		37,093	1,560	1,241	2,19	0,94	n.a.
			**71,103**	**131,494**	**100,00**	**100,00**	

Naphtho[c]oxepinone derivative (6ca)

Chromatogram

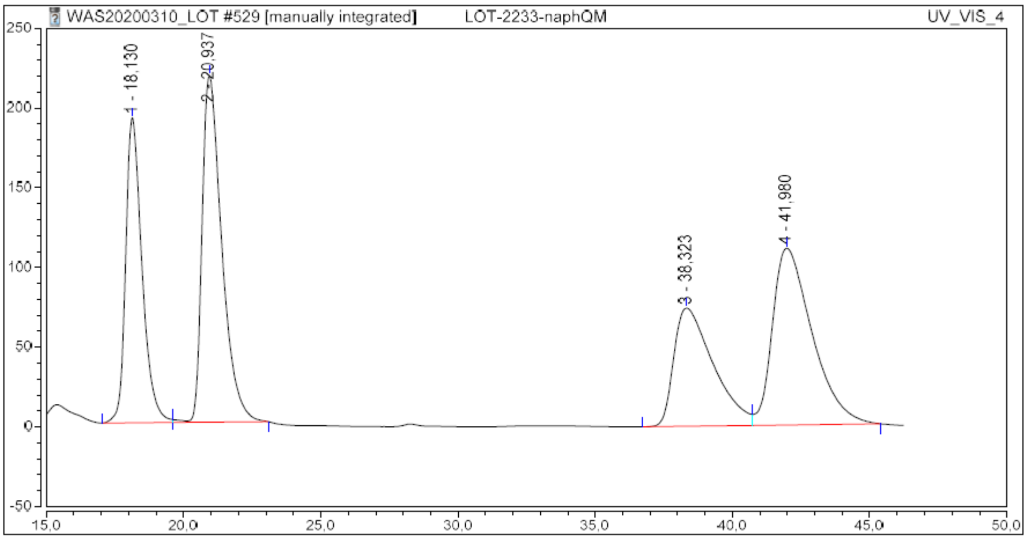

Integration Results

No.	Peak Name	Retention Time min	Area mAU*min	Height mAU	Relative Area %	Relative Height %	Amount n.a.
1		18,130	132,333	191,299	21,29	32,21	n.a.
2		20,937	180,550	217,544	29,05	36,63	n.a.
3		38,323	122,732	74,100	19,75	12,48	n.a.
4		41,980	185,829	110,911	29,90	18,68	n.a.
			**621,444**	**593,855**	**100,00**	**100,00**	

Chromatogram

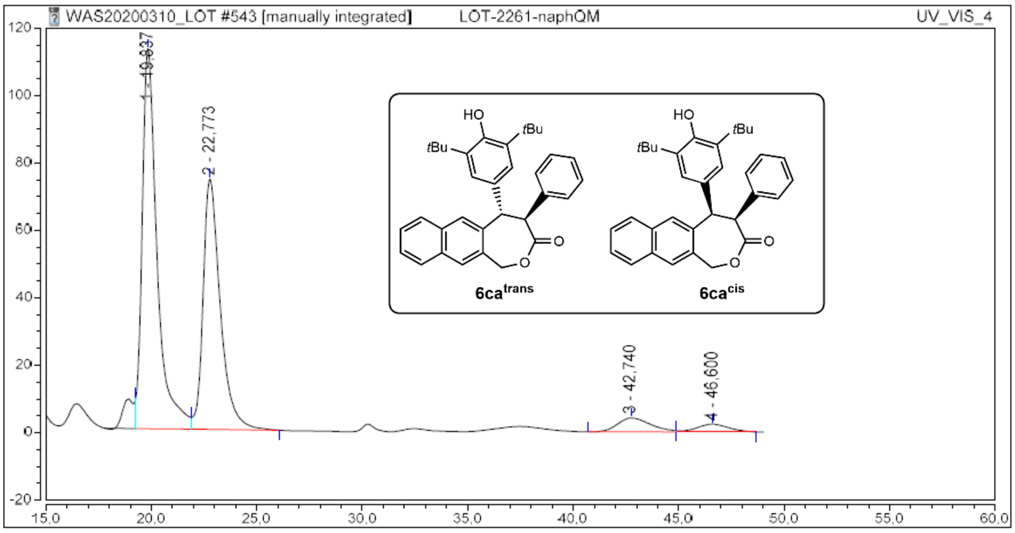

Integration Results

No.	Peak Name	Retention Time min	Area mAU*min	Height mAU	Relative Area %	Relative Height %	Amount n.a.
1		19,837	91,610	112,669	52,91	58,25	n.a.
2		22,773	70,226	74,374	40,56	38,45	n.a.
3		42,740	7,362	4,106	4,25	2,12	n.a.
4		46,600	3,942	2,276	2,28	1,18	n.a.
			**173,139**	**193,425**	**100,00**	**100,00**	

5-(3-(tert-butyl)-4-hydroxy-5-methylphenyl)-4-phenyl-4,5-dihydrobenzo[c]oxepin-3(1H)-one (6da)

Cis Atropisomers (R_a^{cis} and S_a^{cis})

Chromatogram

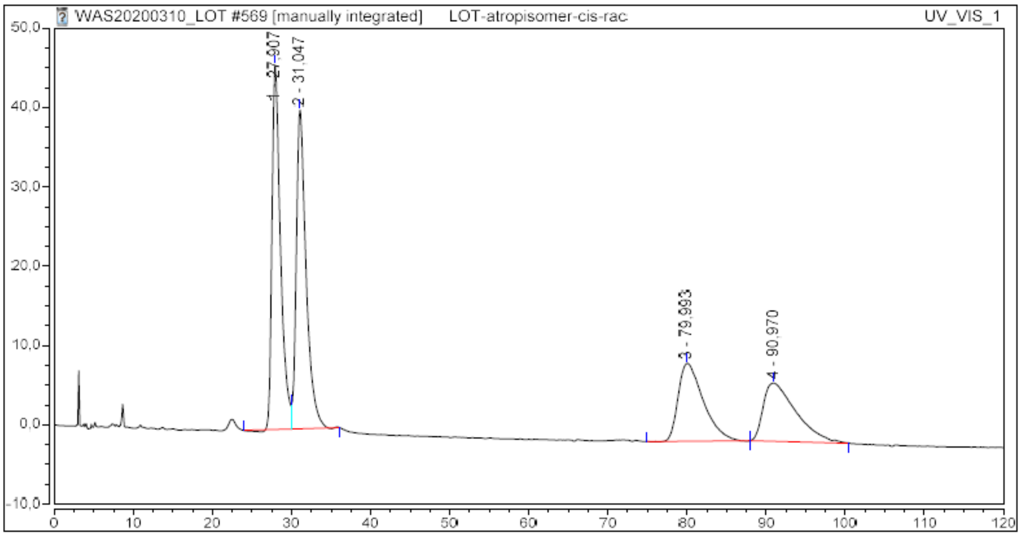

Integration Results

No.	Peak Name	Retention Time min	Area mAU*min	Height mAU	Relative Area %	Relative Height %	Amount n.a.
1		27,907	57,245	45,952	30,76	44,47	n.a.
2		31,047	57,548	40,199	30,93	38,90	n.a.
3		79,993	36,147	9,837	19,43	9,52	n.a.
4		90,970	35,142	7,353	18,89	7,12	n.a.
			**186,081**	**103,342**	**100,00**	**100,00**	

Chromatogram

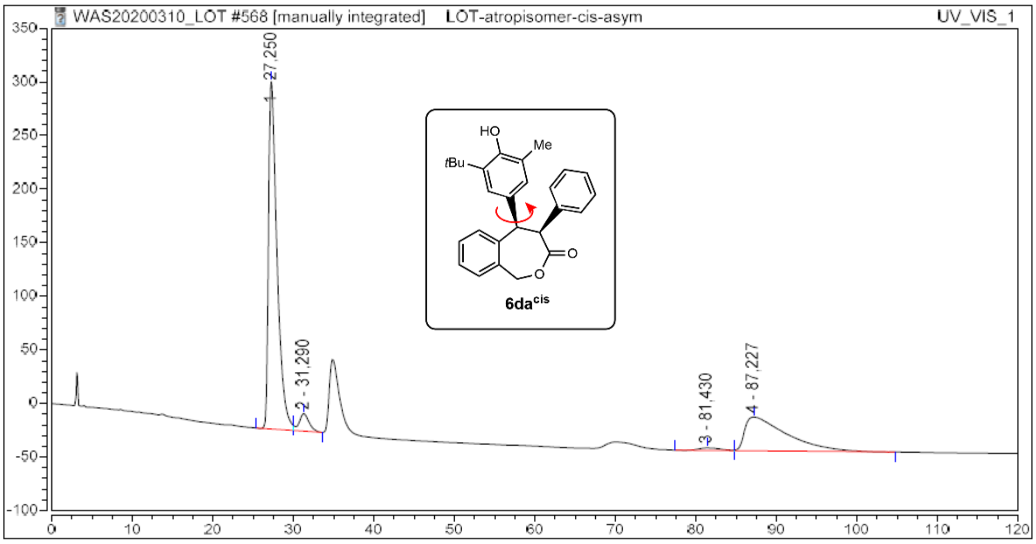

Integration Results

No.	Peak Name	Retention Time min	Area mAU*min	Height mAU	Relative Area %	Relative Height %	Amount n.a.
1		27,250	396,681	324,421	64,20	86,54	n.a.
2		31,290	23,449	16,527	3,80	4,41	n.a.
3		81,430	7,266	2,400	1,18	0,64	n.a.
4		87,227	190,459	31,530	30,83	8,41	n.a.
			**617,855**	**374,878**	**100,00**	**100,00**	

Trans Atropisomers (R_a^{trans} and S_a^{trans})

Chromatogram

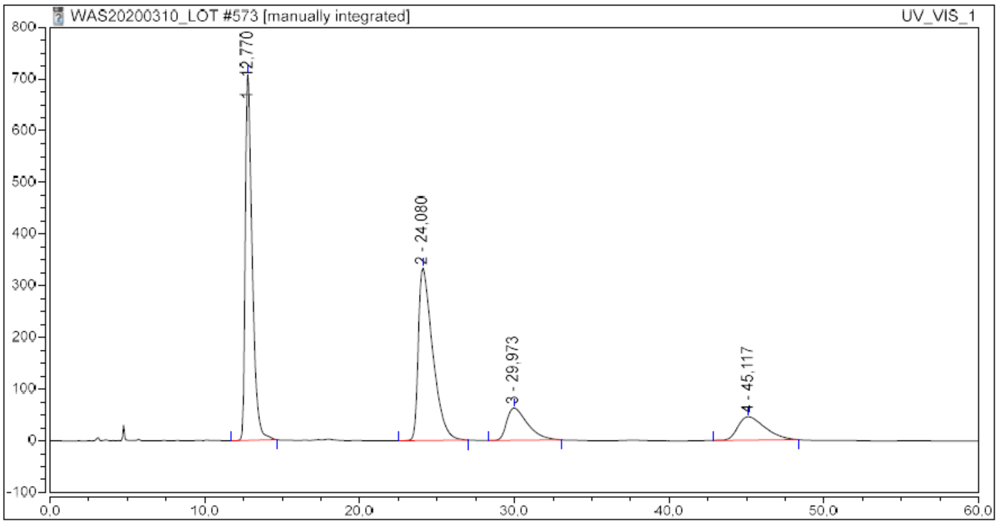

Integration Results

No.	Peak Name	Retention Time min	Area mAU*min	Height mAU	Relative Area %	Relative Height %	Amount n.a.
1		12,770	367,549	707,586	40,14	61,56	n.a.
2		24,080	359,451	333,911	39,26	29,05	n.a.
3		29,973	97,146	62,735	10,61	5,46	n.a.
4		45,117	91,531	45,260	10,00	3,94	n.a.
			**915,676**	**1149,493**	**100,00**	**100,00**	

Chromatogram

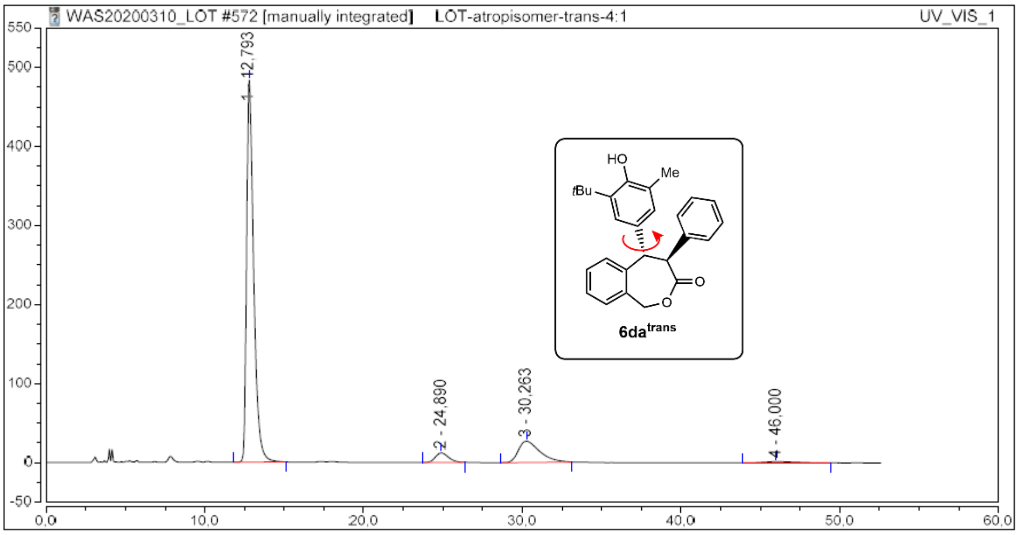

Integration Results

No.	Peak Name	Retention Time min	Area mAU*min	Height mAU	Relative Area %	Relative Height %	Amount n.a.
1		12,793	248,968	483,381	81,40	92,23	n.a.
2		24,890	11,699	12,193	3,82	2,33	n.a.
3		30,263	41,807	26,893	13,67	5,13	n.a.
4		46,000	3,397	1,610	1,11	0,31	n.a.
			**305,872**	**524,077**	**100,00**	**100,00**	
